# Intermolecular CDC amination of remote and proximal unactivated C_sp^3^_–H bonds through intrinsic substrate reactivity – expanding towards a traceless directing group†‡

**DOI:** 10.1039/d1sc04365j

**Published:** 2021-10-27

**Authors:** Suresh Rajamanickam, Mayank Saraswat, Sugumar Venkataramani, Bhisma K. Patel

**Affiliations:** Department of Chemistry, Indian Institute of Technology Guwahati North Guwahati Address Assam-781039 India patel@iitg.ac.in; Department of Chemical Sciences, Indian Institute of Science Education and Research (IISER) Mohali Sector 81, Knowledge City, Manauli SAS Nagar 140306 India sugumarv@iisermohali.ac.in

## Abstract

An intermolecular radical based distal selectivity in appended alkyl chains has been developed. The selectivity is maximum when the distal carbon is *γ* to the appended group and decreases by moving from *γ* → *δ* → *ε* positions. In –COO– linked alkyl chains, the same distal *γ*-selectivity is observed irrespective of its origin, either from the alkyl carboxy acid or alkyl alcohol. The appended groups include esters, N–H protected amines, phthaloyl, sulfone, sulfinimide, nitrile, phosphite, phosphate and borate esters. In borate esters, boron serves as a traceless directing group, which is hitherto unprecedented for any remote C_sp^3^_–H functionalization. The selectivity order follows the trend: 3° benzylic > 2° benzylic > 3° tertiary > *α* to keto > distal methylene (*γ* > *δ* > *ε*). Computations predicted the radical stability (thermodynamic factors) and the kinetic barriers as the factors responsible for such trends. Remarkably, this strategy eludes any designer catalysts, and the selectivity is due to the intrinsic substrate reactivity.

## Abstract 2

An intermolecular amination at the distal methylene carbon has been realized in an appended alkyl chain with electron withdrawing groups. Traceless remote C_sp^3^_–H functionalization has been accomplished using borate esters.

## Graphical abstract

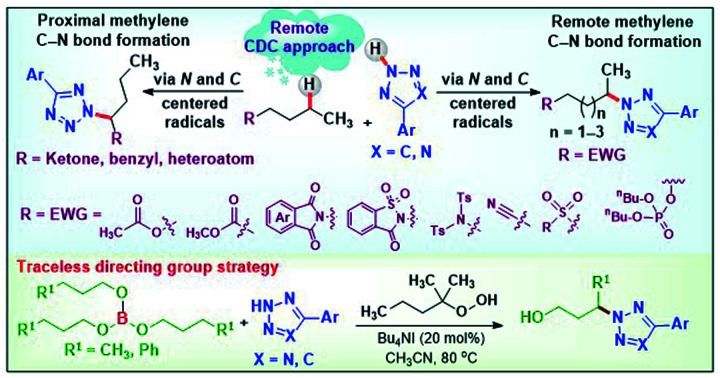

## Introduction

Functionalization of inert C_sp^3^_–H bonds with a high degree of selectivity is one of the most challenging yet desirable avenues in organic synthesis. In living systems, the enzyme cytochrome P_450_ uses an intricate binding pocket to achieve this transformation in appended alkyl chains with precise selectivity onto a particular substrate.^1^ Chemists have successfully functionalized C_sp^3^_–H bonds adjacent to π-systems,^2^ heteroatoms^2*b*,3^ or using directing groups.^4^ Lately, chemists have developed designer metal catalysts or molecular recognition units to functionalize C_sp^3^_–H bonds of the same type without the assistance of directing groups.^5^ The catalysts/oxidants achieve selectivity through electronic, steric and stereo-electronic factors inherited in the substrates; though it is quite often that the examined substrates are electronically biased.^2^

Several strategies have emerged for the non-directed remote C_sp^3^_–H functionalization of aliphatic compounds. For instance, the methine and methylene C–H bonds have been selectively oxidized using Fe(PDP)/H_2_O^6*a*–*c*^ and NO_2_[Fe–bTAML]/*m*-CPBA^6*d*^ in complex substrates. An electrochemical method demonstrates the oxyfunctionalization of electron-rich methylene carbon centers at remote positions.^7*a*^ Intermolecular remote C_sp^3^_–H bromination,^7*b*^ chlorination^7*c*^ and xanthylation^7*d*^ have been accomplished utilizing *N*-halo and *N*-xanthylamides under irradiation of visible light Zhdankin's azidoiodinane method. Indeed, it has been used in association with an Fe(ii)/i-Pr-PyBox-catalyst for the selective azidation of electron-rich, remote methine over methylene and methyl centers.^8^ Subsequently, azidation is achieved using a Mn-catalyst,^9*a*^ and radical trifluoromethylthiolation (SCF_3_) is mediated by AgSCF_3_ (ref. 9b and c). The site selectivity of substrates can be altered by fine-tuning the catalytic systems.^9*d*^ Synthetic chemists have made significant strides in the field of remote C_sp^3^_–H functionalization; however, this area remains as exciting as ever, and several reactions are yet to be discovered and generalized.

Compounds containing C–N bonds find extensive utility in diverse areas of chemistry, and several reaction methods have emerged. Historically, the Hofmann–Löffler–Freytag (HLF) reaction is employed for intramolecular amination, involving the C_sp^3^_–H bond.^10*a*,*b*^ The modern HLF reaction is achieved *via* electrochemical^11*a*^ or a combination of photo and electrochemical processes.^11*b*^ Currently, the direct conversion of the C–H bond to the C–N bond is demonstrated by nitrene chemistry,^12^ directing groups,^4*a*,*b*,13*a*,*b*^ and cross dehydrogenative coupling (CDC) strategies.^2*a*,*b*,*g*–*i*,14^ Performing intermolecular aminations *via* nitrene chemistry is a tedious task as the metallo-nitrene intermediates are unstable, and the scope is limited mainly to electronically biased benzylic or tertiary sites.^12*d*,*g*–*i*^ Strategies based on directing groups require two additional steps during the synthesis—pre-functionalization of the starting materials followed by deprotection. Moreover, the reductive elimination step in the catalytic cycle is hampered by the high binding affinity of the newly introduced amino moieties.^15^ The CDC predominantly works for activated C–H bonds that are adjacent to heteroatoms,^3*f*,*g*^ or π-systems.^2*b*,*c*,*g*–*i*^ Intermolecular amination *via* CDC remains hitherto undiscovered in substrates that can engage remote, unactivated methylene sites in the coupling process. Moreover, the question arises, whether the selectivity in a structurally diverse substrate is a result of steric, or stereo-electronic factors or the outcome of the catalyst and oxidant.

The tetrazole moiety is highly admired among N-heterocyclic compounds: it acts as a bioisostere of carboxylic acid surrogates due to similar p*K*_a_'s and renders high metabolic stability.^16^ For instance, biphenyl tetrazoles are key intermediates in the production of multibillion-dollar angiotensin II receptor antagonists, a class of drugs for treating high blood pressure known as sartan drugs; pemirolast—an antiallergic drug; and azosemide—a diuretic. Also, many of the cephalosphorine derivatives possess tetrazole units (Fig. S1, see ESI‡).^16^ The [Mn^III^(ClPc)]^12*d*^ and Rh_2_(esp)_2_ (ref. 12g–i) catalysts can efficiently differentiate large substrate variation which is applied to complex molecules, such as terpenes, steroids, alkaloids, peptides, and lactams.^12*d*,*g*–*i*^ In most cases, the functionalization is limited to a position in electronically biased substrates, such as the tertiary or benzylic.^12*d*,*g*–*i*,*n*^ Thus, it is challenging to achieve amination at undirected C–H bonds with predictable site-selectivity without a *de novo* approach. Can a remote and rational C_sp^3^_–H amination strategy be developed based on the intrinsic substrate reactivity that can be expanded to a traceless directing group? If so, what are the possible determining factors?

## Results and discussion

A nitrogen centered radical (NCR) is generated by reacting tetrazole with a combination of iodide and peroxide or *tert*-butyl hydroperoxide (TBHP)/tetrabutylammonium iodide (TBAI).^17*a*,*b*^ In the absence of any other radical coupling partners, the generated NCR abstracts a methyl (–CH_3_) group from the TBHP giving a N^2^-methylated tetrazole exclusively.^17*a*^ Taking cues from this and the non-directed remote C_sp^3^_–H bond functionalization strategies,^6–8,9*a*^ particularly Wu's and Tang's trifluorothiomethylation,^9*b*,*c*^ we envisaged the incorporation of N-heterocycles (tetrazoles) at an unactivated remote methylene C_sp^3^_–H position. With this idea in mind, we set up a reaction at 70 °C between 5-phenyl-2*H*-tetrazole a and *n*-butyl acetate 1 in the presence of Bu_4_NI and the oxidant, TBHP (5–6 M in decane). As anticipated, the site-selective *γ*-aminated product 1a was achieved in 21% yield. Since the oxidant, TBHP, is dissolved in *n*-decane, the *in situ* generated tetrazole radical reacted with it affording an indiscriminate mixture of aminated *n*-decane products, which is quite unsolicited. This preliminary result was quite encouraging, as the remote CDC of 1 with a evades any directing group or designer catalyst. Next, the optimization parameters were scrutinized by varying the reaction temperature, oxidant, catalyst, and its loading to improve the reaction efficacy. After extensive exploration (Table S1, see ESI‡), it was revealed that subjecting a (0.5 mmol) to two iterative additions of 10 mol% of Bu_4_NI, two equivalents of oxidant (aq TBHP), and 330 μl of 1 at the beginning and after an interval of 2.5 h at 80 °C afforded product 1a in 69% yield after 8 h (Table S1, see ESI‡).

**Scheme 1:**
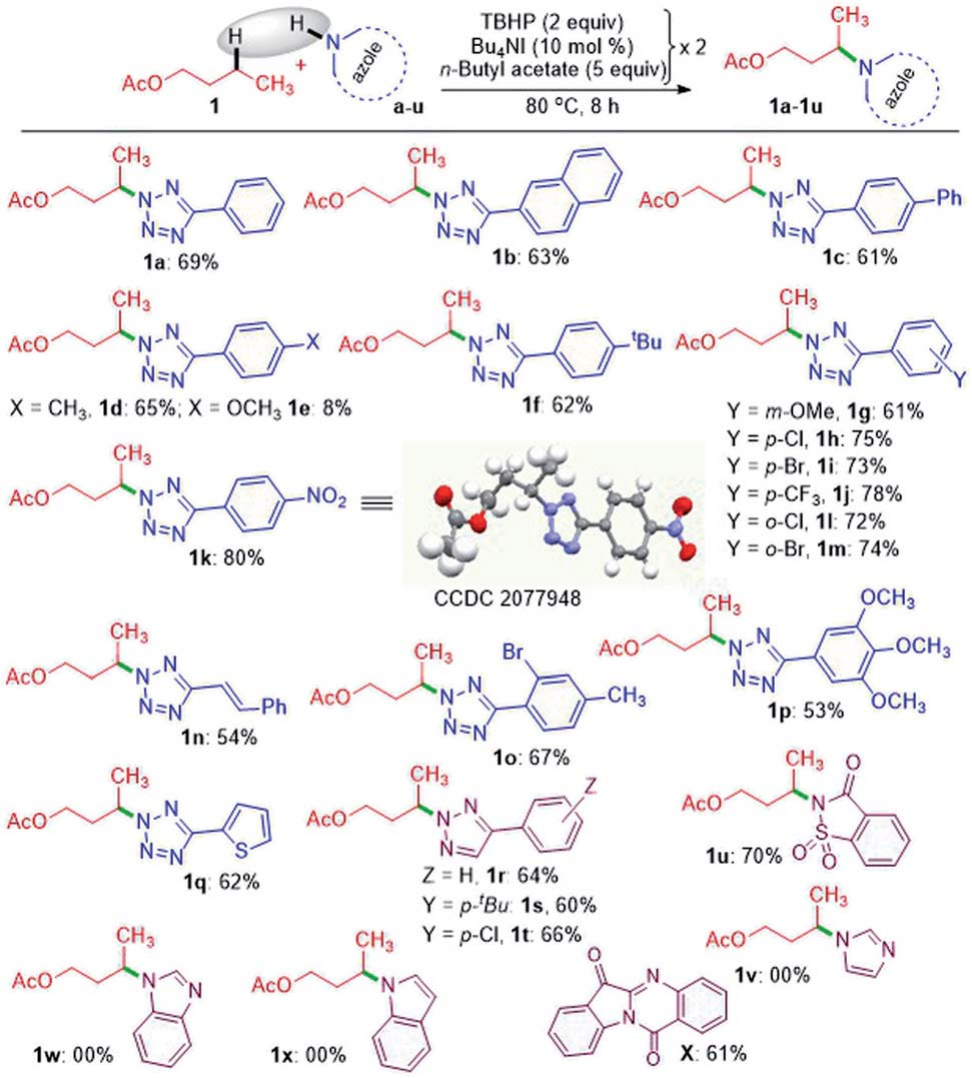
Substrate scope for the intermolecular amination of *n*-butyl acetate. ^*a*^ Reaction conditions: azole (0.5 mmol), *n*-butyl acetate (5 equiv. × 2), Bu_4_NI (10 mol% × 2) and aq TBHP (2 equiv. × 2) at 80 °C for 8 h in an inert atmosphere. ^*b*^ Isolated yields.

A range of substituted tetrazoles a–q were subjected to the optimized conditions, and the results are summarized in Scheme 1. Tetrazole a and π-conjugated tetrazoles b and c coupled with 1 giving products (1a–1c) in modest yields. The key feature here is the occurrence of exclusive amination at the *γ*-position of a, leading to a single regioisomer. The regioselectivity can be attributable to a slight kinetic preference in the ease of formation of the radical center in 1 at *γ* over its *α* position. {For further details, see the Computational studies section (Fig. 2)}. The phenyl ring of aryl tetrazole possesses either electron-donating d–g or electron-withdrawing h–m substituents smoothly coupled with 1 to afford their *γ*-tetrazolyl products 1d–1m in moderate to good yields (Scheme 1). When a π-conjugated tetrazole n reacted with 1, it gave *γ*-aminated product 1n. Furthermore, aryl tetrazoles o and p having multiple substituents afforded their respective products 1o, and 1p. A thiophenyl tetrazole q provided its product 1q in modest yield. The regioselectivity of the product was ascertained by X-ray crystallographic analysis, as shown in Scheme 1. Recently the Patel group achieved similar N^2^ alkylation using TBAI/TBHP combination in the presence of various organic peroxides.^17*a*^ Computations revealed the determining factors for exclusive N^2^ regioselectivity such as the high spin density at the N^2^ center, low transition state barriers, and better thermodynamic stability of the products.^17*a*^

Next, we sought the possibilities of remote amination of 1 with other azoles and N-heterocycles, such as triazoles r–t, saccharin u, imidazole v, benzimidazole w and indole x (Scheme 1), while aryltriazoles r–t and saccharin u reacted successfully giving *γ*-functionalized products 1r–t and 1u; imidazole v and benzimidazole w failed to react (Scheme 1). However, indole x provided tryptanthrine X in 61% yield, which is well documented.^18^ The failure to undergo similar amination for v and w is any one or a combination of the factors, namely (i) delocalization and stabilization of the radical with no or minimal radical character at the nitrogen center, (ii) significant activation energy, and (iii) unfavourable thermodynamic stability of the product.^17*a*^

**Scheme 2:**
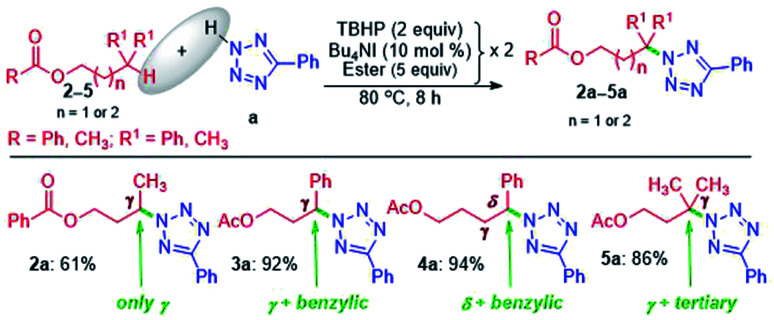
Substrate scope of amination at remote methylene, benzylic and tertiary-methine sites. ^*a*^ Reaction conditions: azole (0.5 mmol), ester (5 equiv. × 2), Bu_4_NI (10 mol% × 2) and aq TBHP (2 equiv. × 2) at 80 °C for 8 h. ^*b*^ Isolated yields.

The successful *γ*-amination of ester a encouraged us to test a similar strategy with an aromatic ester 2 which provided a *γ*-tetrazolyl product 2a in 61% yield (Scheme 2). This result suggests that both methyl and phenyl groups have identical or no influence when present on the carbonyl side of the ester, and the fate is dictated by the ester –COO– moiety. However, after mutually swapping the position of the phenyl and the methyl group as in 3, the *γ*-tetrazolyl product 3a was isolated in a very high yield (92%). The phenyl group, which is now present at the *γ*-position, is electronically biased as it is also benzylic; thus, there is a positive influence on the selectivity and the yield. Further, an exclusive *δ*-functionalization without a trace of *γ*-product in ester 4a confirmed the complete electronic biasness.^12*d*^ Further, an ester having a *γ*-methine hydrogen (3° C–H), as in 5, provided an exclusive *γ*-tetrazolyl product 5a. Thus, the current strategy evades designer catalysts, and the selectivity is due to the inherent substrate reactivity.

**Scheme 3:**
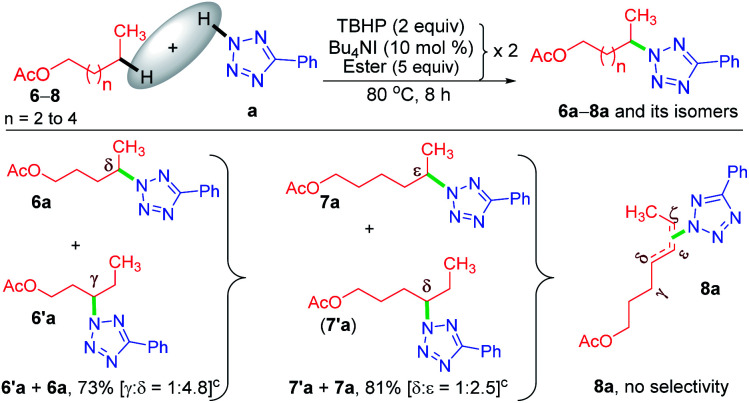
Substrate scope for the amination of alkyl acetates. ^*a*^ Reaction conditions: 5-phenyl-2*H*-tetrazole (0.5 mmol), ester (5 equiv. × 2), Bu_4_NI (10 mol% × 2) and aq TBHP (2 equiv. × 2) at 80 °C for 8 h. ^*b*^ Isolated yields. ^*c*^ Products obtained as an inseparable regioisomeric mixture and the ratio determined by ^1^HMR analysis.

Now, a further query arose: other than the benzylic position, does this protocol always provide *γ*-selectivity, or is this amination feasible beyond this position, particularly for substrates having longer alkyl chains? With this objective in mind, ester 6 possessing *γ* and *δ* positions was tested, which provided an inseparable mixture of *γ* and *δ* aminated products 6′a and 6a in a combined yield of 73% in the ratio of 1 : 4.8 (Scheme 3). The higher percentage of the *δ*-product 6a is due to the better stability of the distal *δ*-carbon center compared to the *γ*-centered radical. In product 1a, the selectivity is at the *γ*-center and in 6a it is at the *δ*-center, and both happen to be at the distal carbon (secondary). This raises further inquisitiveness: whether the selectivity is dictated by the distance from the ester moiety (*γ* or *δ* position) or only by the distal methylene carbon. When hexyl acetate 7 was subjected to identical reaction conditions, it provided an inseparable mixture of *δ* and *ε* aminated products, 7′a and 7a in the ratio of (1 : 2.5) in a combined yield of 81% (Scheme 3), suggesting a preferential distal selectivity. Undoubtedly, the *ε*-selective product 7a was the major one, but the degree of distal selectivity decreased considerably. No preferential selectivity was observed for *n*-heptyl acetate 8, as it provided a mixture of *δ*, *ε*, and ζ aminated products whose exact ratio could not be ascertained. The distal selectivity is governed by the electronic influence imparted by the –COO– group along the alkyl chain.^7*a*–*d*,9*b*,*c*^

In this regard, we computationally estimated the C–H bond dissociation energies (BDEs) for the compounds having more than four carbons in the alkyl chain (C-5 ester 6 and C-6 ester 7).^19^ Surprisingly, the values are nearly the same for the distal positions. The observed lower C–H BDEs and their difference (0.1–0.2 kcal mol^−1^) are not only consistent with the isolated mixture of products but also the difference in the stability [inferred from the comparison of C–H BDEs at all the centers] that can be accountable for the distal selectivity.^20^

**Scheme 4:**
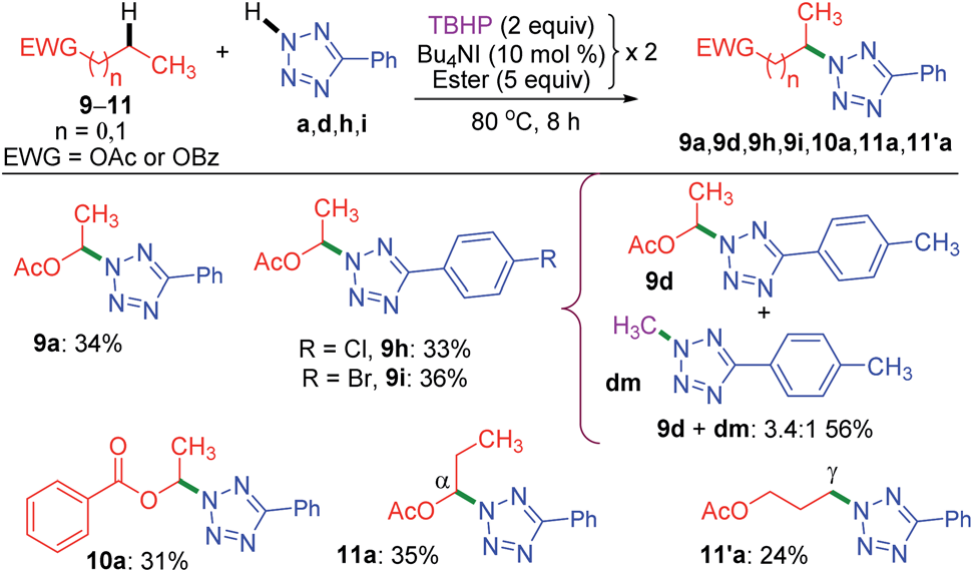
Substrate scope for intermolecular amination of esters.^*a*^ Reaction conditions: aryl tetrazole (0.5 mmol), ester (5 equiv. × 2), Bu_4_NI (10 mol% × 2) and aq TBHP (2 equiv. × 2) at 80 °C for 8 h. ^*a*^ Isolated yield.

Now, the query arises about the selectivity in short-chain esters, say C-2 esters 9 and 10 possessing no *γ*-carbon, and a C-3 ester having a terminal but a primary *γ*-carbon. With this objective in mind, we treated 9 and 10 with a. The amination took place only at the *α*-position to yield products 9a and 10a in lower yields with no traces of *β* products (Scheme 4). Despite the superior stability of the *α*-C radical, the reduced yields of products 9a and 10a are due to the competitive *N*-methylation of tetrazole.^17*a*^ For tetrazoles h and i, the products 9h and 9i were isolated in their pure form. However, tetrazole d provided an inseparable mixture of *α*-aminated product 9d and 2-methyl-5-(*p*-tolyl)-2*H*-tetrazole **dm**^17*a*^ in 3.4 : 1 ratio. The C-3 ester 11 provided two isomeric products, namely *α*-aminated (11a, 35%) and *γ*-aminated (11′a, 24%) products without giving any *β*-isomer. Both the *α*-, and *γ*-functionalized products 11a and 11′a might be forming *via* a radical pathway due to the inductive (–I) influence of the ester group. An alternative pathway for the *α*-functionalized product 11a originating *via* the formation of an oxocarbenium is less likely.^21*a*^

**Scheme 5:**
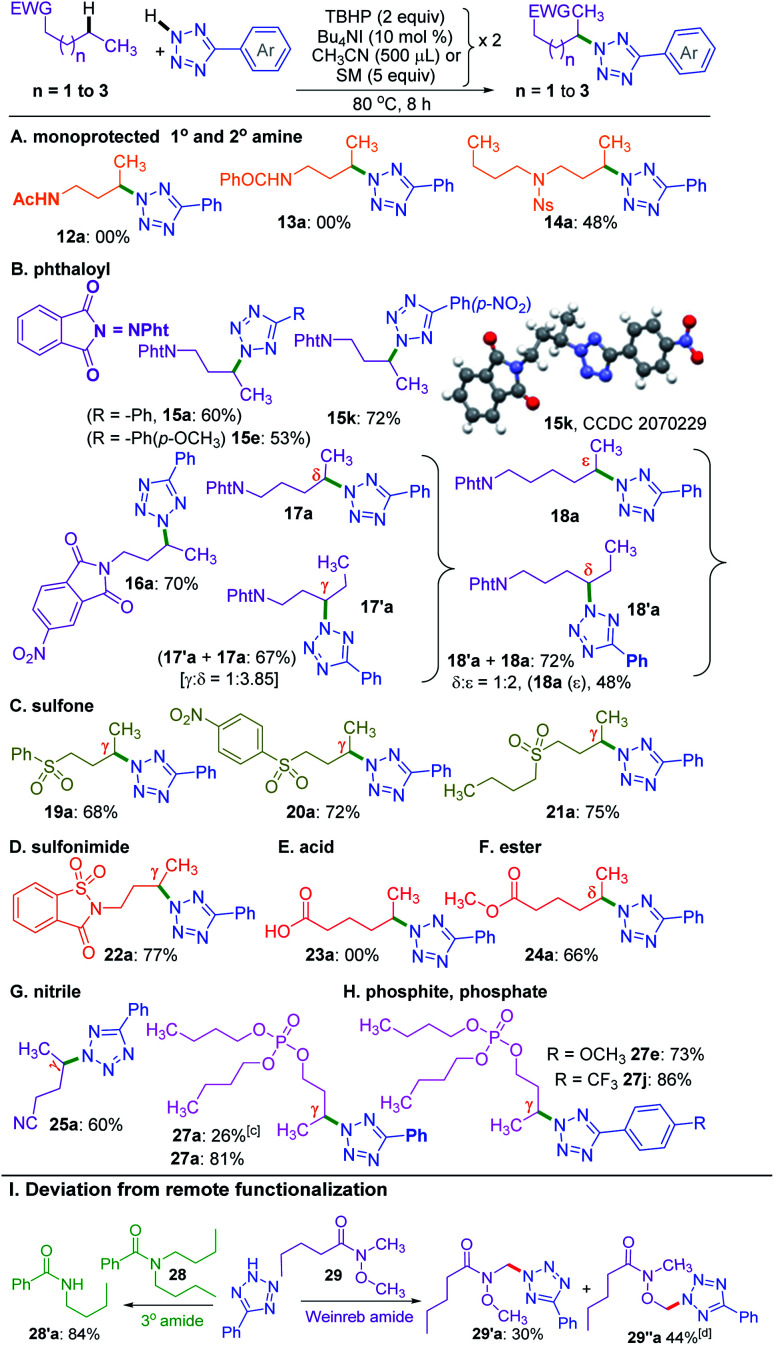
Substrate scope for the amination of various electron withdrawing groups. ^*a*^ Reaction conditions: (A–D), aryl tetrazole (0.5 mmol), substrates 12–22 (0.5 mmol), Bu_4_NI (10 mol% × 2), aq TBHP (2 equiv. × 2) and CH_3_CN (500 μl × 2) at 80 °C for 8 h. (E–H), azoles (0.5 mmol), substrates 23–27 (5 equiv. × 2), Bu_4_NI (10 mol% × 2) and aq TBHP (2 equiv. × 2) at 80 °C for 8 h. ^*b*^ Isolated yield. ^*c*^ Tributyl phosphite (26) used as the starting material. I, aryl tetrazole (0.5 mmol), substrates 28, 29 (0.5 mmol), Bu_4_NI (10 mol% × 2), aq TBHP (2 equiv. × 2) and CH_3_CN (500 μl × 2) at 80 °C for 8 h. ^*d*^ Isolated along with an inseparable mixture of uncharacterized impurities.

So far, the substrates examined are ester appended alkyl moieties that dictate the site-selective amination by intrinsic reactivity. Other electron-withdrawing groups, such as amide, phthaloyl, ketone, sulfone, cyano, phosphate, electron-deficient heterocycles, and sulfonimide, were tested to establish similar influences (Scheme 5). Primary alkyl amides 12 and 13 both failed to provide any product. Interestingly, a nosyl protected dibutyl amine 14 provided mono *γ*-tetrazolyl product 14a, which suggests the detrimental influence of the free N–H group. This fact was further confirmed when a phthaloyl protected *n*-butyl amine 15 coupled with a and provided the product 15a in 60% yield. Additionally, substrate 15 reacted with e and k, and delivered exclusive *γ*-tetrazolylated products 15e and 15k. The regio- and site-selectivity of the product was confirmed by X-ray crystallography (Scheme 5). In a similar vein, 5-nitrophthalic anhydride protected *n*-butyl amine 16 delivered product 16a.

To determine the supremacy of the distal methylene selectivity, the chain length was increased from C-4 to C-5. A phthaloyl protected *n*-pentyl amine 17 gave an inseparable mixture of *γ* : *δ* isomeric products 17′a and 17a in the ratio of 1 : 3.8. On the other hand, for an *n*-hexyl amine derivative 18, the *δ* : *ε* selectivity was found to be 1 : 2. These results reconfirmed the dominance of distal methylene selectivity. Thus, the presence of electron-withdrawing moieties such as esters and protected secondary amines is all capable of dictating the regioselective amination in an alkyl chain at the distal methylene carbon. Interestingly, aryl butyl sulfones 19 and 20 also provided exclusive *γ*-aminated products 19a and 20a, whereas a dibutyl sulfone 21 possessing two butyl chains yielded only a mono *γ*-aminated product 21a (Scheme 5). An *n*-butyl saccharin 22 possessing a strong electron-withdrawing sulfonimide group provided product 22a. Unprotected alkyl carboxylic acid, such as hexanoic acid 23, failed to deliver any product, suggesting the detrimental influence of the free carboxy group. However, when the acid was converted to methyl hexanoate 24, it afforded the *δ*-aminated product 24a. Thus, there is a distinct distal selectivity irrespective of the attached appended group in an alkyl chain (either from the alkyl carboxylic acid or alkyl alcohol). Similar distal methylene tetrazolylation was observed for valeronitrile 25 affording product 25a. As further testimony to the distal selectivity, a reaction between a tributyl phosphite 26 and a provided a mono *γ*-aminated product with concurrent oxidation of the phosphite to a phosphate derivative 27a in 26% yield. The lower yield obtained is due to the consumption of the oxidant in converting the phosphite to a phosphate. However, a fully oxidized species 27 provided mono *γ*-aminated product 27a in 81% yield. Again, high yields were obtained when phosphate 27 reacted with aryl tetrazoles e and j, giving products 27e and 27j, respectively (Scheme 5).

On the other hand, an *n*-butyl chain bearing a tertiary amide such as *N*,*N*-dibutylbenzamide 28 in the presence of 5-phenyl-2*H*-tetrazole a under the standard reaction conditions did not provide the *γ*-tetrazolylated product rather underwent dealkylation producing *N*-butylbenzamide 28′a in 84% yield (Scheme 5). Similar dealkylation using 5,10,15,20-tetraphenylporphyrinato iron(iii) chloride–Bu^*t*^OOH is reported.^21*b*^ The reaction between *N*-methoxy-*N*-methylpentanamide (Weinreb amide) (29) and tetrazole under the present conditions provided a multitude of products. The major CDC product occurs adjacent to oxygen and nitrogen heteroatoms. The CDC product adjacent to the nitrogen atom 29′a could be isolated in its pure form. But the product adjacent to oxygen 29′′a ended up along with other inseparable uncharacterized products.

**Scheme 6:**
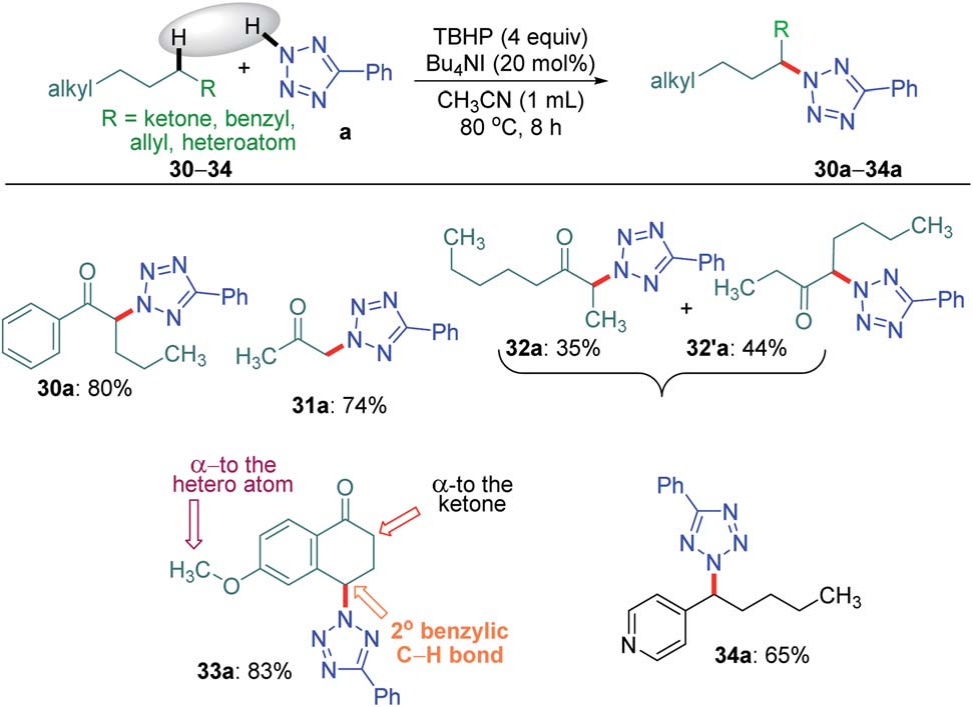
Substrate scope for alpha-site-selective amination. ^*a*^ Reaction conditions: 5-phenyl-2*H*-tetrazole (0.5 mmol), substrates 30–34 (0.5 mmol), Bu_4_NI (20 mol%), aq TBHP (4 equiv.) and CH_3_CN (1 mL) at 80 °C for 8 h. ^*b*^ Isolated yield.

Intriguingly, another electron-withdrawing group, a ketone, possessing a *γ*-methylene site, as in valerophenone 30 led to *α*-functionalized product 30a in 80% yield (Scheme 6). Acetone 31 having two symmetrical methyl groups *α-* to carbonyl reacted with a to yield product 31a. Unsymmetrical dialkyl ketone having two sets of alpha hydrogen as in 32 afforded a regioisomeric mixture of products 32a and 32′a (1 : 1.25) in 35% and 44% yields, respectively. There is a marginal preference towards the *α*-side of the longer alkyl chain. This observation is consistent with Zhang's oxidative imidation of ketone with saccharin.^22^ Computationally BDEs and the spin densities are estimated to be quite similar for both the regioisomeric radicals. However, there is a slight kinetic bias (0.2–0.3 kcal mol^−1^) for the formation of radicals at the alpha position to the longer alkyl chain, which accounts for a marginal preference for forming 32′a over 32a (see Fig. 4 in the Computational studies section).

We examined another keto containing substrate, 6-methoxy tetralone 33, having three potential amination sites namely a benzylic, a methoxy and an alpha C–H to the ketone to access the most preferential site. Substrate 33 provided an exclusive mono-aminated product 33a at its benzylic position, without affecting the other two sites (Scheme 6). This preferential amination at the benzylic position is further evident when an alkyl pyridine 34 gave its exclusive benzylic product 34a in 65%.

**Scheme 7:**
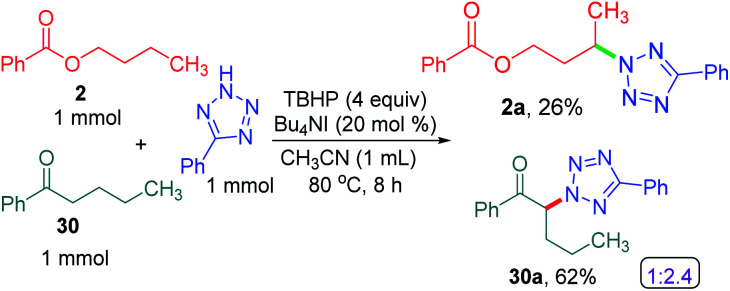
Intermolecular selectivity between *α*-carbon to ketone and distal carbon. ^*a*^ Reaction conditions: 5-phenyl-2*H*-tetrazole (1 mmol), substrates 2 and 30 (1 mmol), Bu_4_NI (20 mol%), aq TBHP (4 equiv.) and CH_3_CN (1 mL) at 80 °C for 8 h. ^*b*^ Isolated yields.

To determine the preferential selectivity order between an *α*-carbon to ketone and a distal methylene carbon in an ester, an intermolecular competitive reaction between 2 and 30 was performed (Scheme 7). Interestingly preferential amination took place at the *α* position of ketone in 30 over the distal methylene carbon in 2 in the ratio of 2.4 : 1 (Scheme 7).

**Scheme 8:**
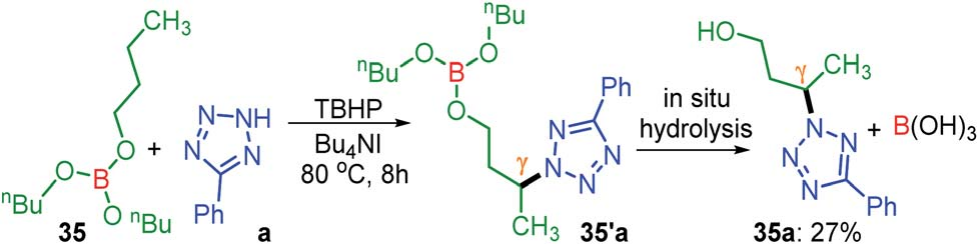
Site-selective amination of tributyl borate. ^*a*^ Reaction conditions: 5-phenyl-2*H*-tetrazole (0.5 mmol), tributyl borate (5 equiv.), Bu_4_NI (20 mol%), aq TBHP (5 equiv.) and CH_3_CN (1 mL) at 80 °C for 8 h. ^*b*^ Isolated yield. Intermediate 35′a was not isolated.

**Scheme 9:**
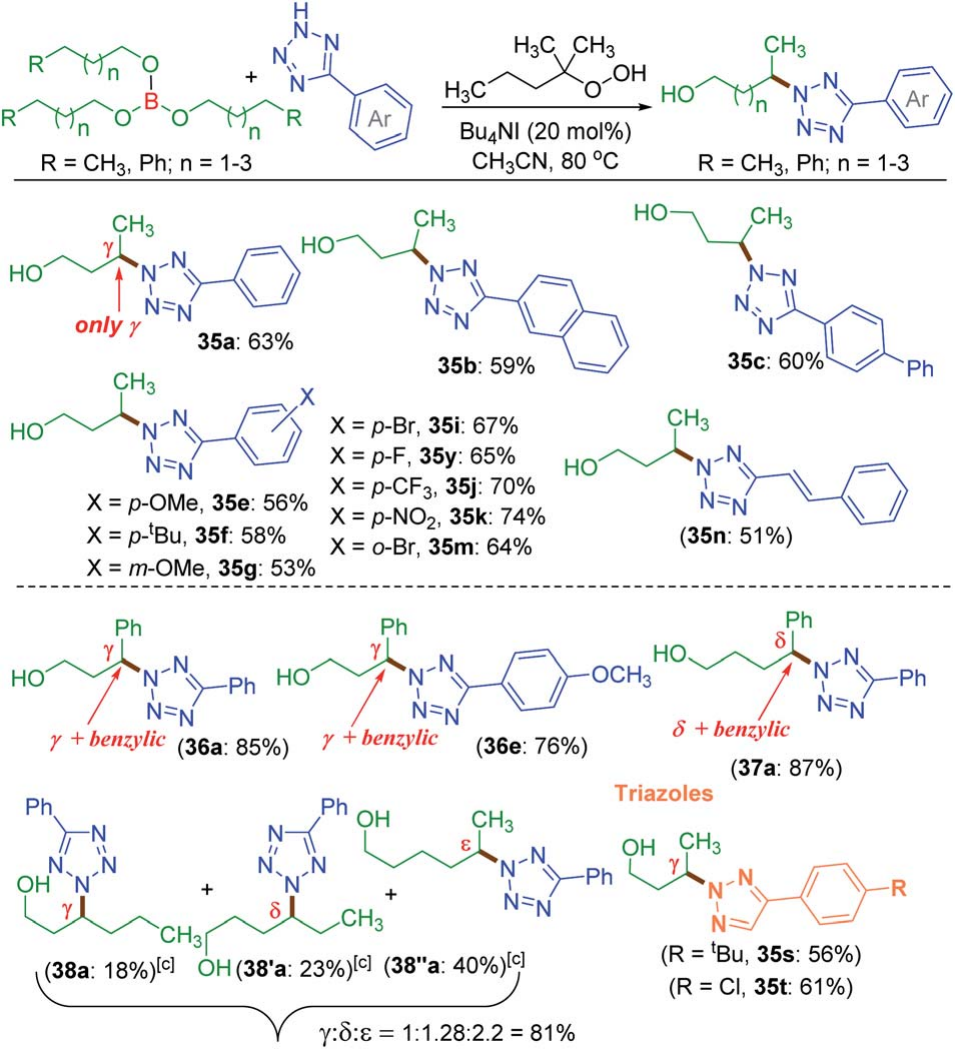
Traceless directing group strategy for amination using borate esters. ^*a*^ Reaction conditions: 5-phenyl-2*H*-tetrazole (0.5 mmol), borate ester 35–38 (0.5 mmol), Bu_4_NI (20 mol%), *tert*-hexyl hydroperoxide (5 equiv.) and CH_3_CN (1 mL) at 80 °C for 8 h. ^*b*^ Isolated yield. ^*c*^ Trihexyl borate (38) was used.

Unlike protected alcohol, amine, amide and carboxylic acid, this method is completely unsuccessful in its free forms. Boron is known to be electron deficient, so for an alkoxy borane, the following question arises: (i) whether the attached alkyl alcohol in the form of alkoxy borane can undergo similar substrate-induced remote amination; (ii) Can the borylated amino alcohols be *in situ* hydrolyzed to generate their amino alcohols and serve as a traceless directing group? With this objective, tributyl borate 35 was subjected to the reaction conditions where mono-amination took place at one of the distal methylene carbons giving 35a in 27% yield (Scheme 8). No doubt the reaction is induced by the central boron atom and proceeds *via* intermediate 35′a, but due to the usage of aqueous TBHP the B–O bond got hydrolyzed to free alcohol before completion of amination. Thus, neither the use of aqueous TBHP nor the decane solution of TBHP is suitable for alkyl borate. The peroxy reagent, *tert*-hexyl hydroperoxide (THHP), available in its pure form, was used instead of TBHP. Using the TBAI/THHP combination, the yield of 35a improved up to 63% (Scheme 9). Thus this is a unique illustration of boron serving as a traceless directing group in any remote functionalization and possesses great synthetic potential.

Employing the modified reaction conditions *i.e.* using *tert*-hexyl hydroperoxide (THHP), *γ*-amination of 35 was accomplished using an array of electronically diverse aryl tetrazoles (Scheme 9). The use of π-conjugated aromatic hydrocarbons bearing tetrazoles, such as naphthyl b and biphenyl c, provided good yields of their corresponding *γ*-aminated butanols 35b and 35c. The phenyl ring of tetrazoles substituted with electron-donating groups such as e–g and electron-withdrawing groups i, k and m all coupled efficiently with 35 to give their respective amino-alcohols 35i, 35k and 35m in yields ranging from 53–74% (Scheme 9). The efficacy of the amination was demonstrated with a cinnamyl tetrazolyl moiety n, which afforded product 35n. The synthetic utility of this transformation was extended to borate 36 and 37 possessing *γ* and *δ* phenyl groups respectively {(36a, 85%), (36e, 76%) and (37a, 87%)}. The high product yields obtained for substrates 36 and 37 at their *γ* and *δ* position are due to the benzylic nature of this traceless directing group assisted strategy. This strategy was then employed to a longer alkyl chain bearing borate 38 with a as the aminating partner. The reaction provided three isolable positional isomers namely *γ* (38a, 18%), *δ* (38′a, 23%) and *ε* (38′′a, 40%). This result clearly shows distal as well as distance selectivity in the order (*ε* > *δ* > *γ*). Unlike in the ester appended alkyl chain (8a, Scheme 3) here, the selectivity is much more distinct and the regioisomeric aminoalcohol could be isolated in their pure form. Finally, this traceless amination strategy was successfully applied to two triazoles s and t with tributylborate 35, and both provided triazolyl alcohols 35s and 35t demonstrating the power of this traceless strategy (Scheme 9).

**Scheme 10:**
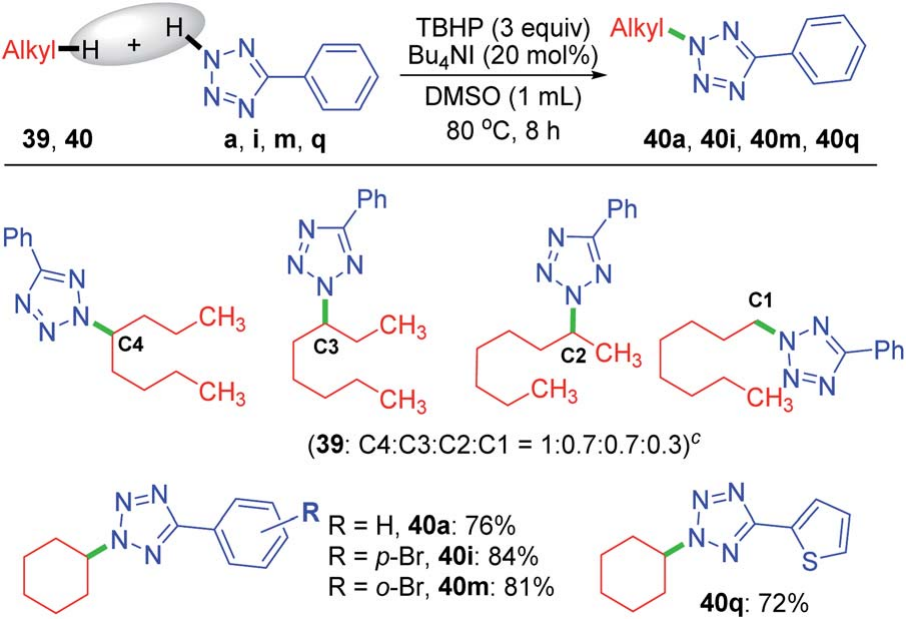
Substrate scope of *N*-cycloalkylation of aryl tetrazoles. ^*a*^ Reaction conditions: aryl tetrazole (0.5 mmol), hydrocarbon 39, 40 (1.0 mmol), Bu_4_NI (20 mol%), aq TBHP (4 equiv.) and DMSO (1 mL) at 80 °C for 8 h. ^*b*^ Isolated yield. ^*c*^ Isomer ratio determined by ^1^H NMR.

Despite the extreme inertness of linear alkane, amination takes place in the decane present in the decane solution of TBHP. This compelled us to use an aqueous solution of TBHP for subsequent investigations. To see if any selectivity can be achieved in a shorter linear chain hydrocarbon having no electronic bias such as *n*-octane 39, *n*-octane 39 was treated with tetrazole a in the presence of TBHP–Bu_4_NI combination in DMSO, resulting in a mixture of inseparable aminated products C4 : C3 : C2 : C1 with a 1 : 0.7 : 0.7 : 0.3 ratio as determined by ^1^H NMR. In contrast, a cyclic hydrocarbon such as cyclohexane 40 reacts with a diverse range of electronically substituted tetrazoles a, i, m and q to afford good yields of tetrazole-*N*-cycloalkylated products (Scheme 10).

**Scheme 11:**
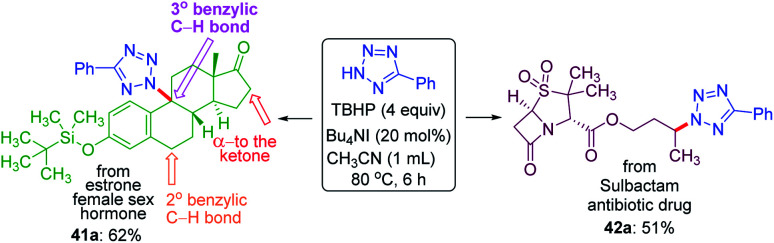
Late stage amination of biologically active molecules. ^*a*^ Reaction conditions: 5-phenyl-2*H*-tetrazole (0.25 mmol), substrates 41 (0.25 mmol), 42 (1 mmol), Bu_4_NI (20 mol%), aq TBHP (4 equiv.) and CH_3_CN (1 mL) at 80 °C for 6 h.

To demonstrate the site-selective intermolecular CDC amination strategy towards late-stage synthetic applications, we subjected estrone 41, a female sex hormone and sulbactam 42 an antibiotic to our present protocol (Scheme 11). For the estrone 41 having both 2° and 3° benzylic carbon and *α*-carbon to the ketone, the amination took place at the sterically hindered 3° benzylic position (41a, 62% yield) without affecting the other two sites suggesting the dominance of the electronic over the steric factor. The sulbactam 42 is a *β*-lactamase inhibitor used in combination with the antibiotic ampicillin for the treatment of bacterial infections resistant to *β*-lactam antibiotics. The butyl esters moiety of sulbactam 42 underwent site-selective intermolecular CDC amination giving product 42a in 51% isolated yield at the electron-rich remote methylene C(sp^3^)–H bond of the ester group.

Thus, the preferential order for site-selective amination obtained so far in an appended alkyl chain from the overall investigation follows 3° benzylic > 2° benzylic > *α* to keto > distal methylene (*γ* > *δ* > *ε*).

### Mechanistic studies

**Scheme 12:**
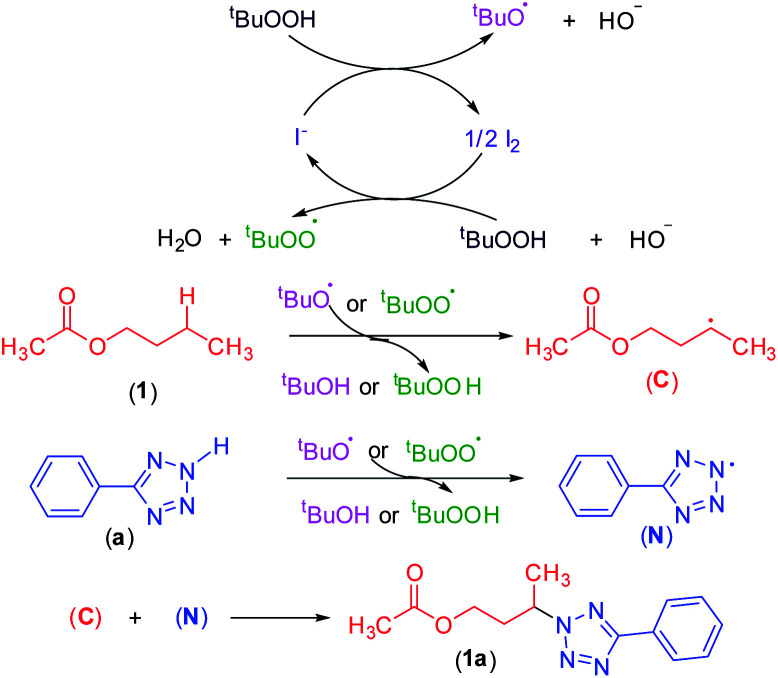
The putative mechanism for an oxidative C–N bond formation.

To shed light on the mechanistic pathway of this site-selective remote intermolecular amination, a series of control experiments were performed (Scheme S1, see ESI‡) from which the following conclusion was drawn: (i) the reaction is non-ionic; (ii) non-involvement of hypoiodite [Bu_4_N]^+^[IO]^−^; (iii) radical nature of the reaction and formation of a radical center on the ester moiety and involvement of the ^*t*^BuOO radical; (iv) formation of the N-centered tetrazolyl radical. Based on our findings and following the previous literature,^7–9,17,23^ a plausible mechanistic pathway is depicted in Scheme 12. Initially, oxidation of iodide by TBHP generates the *tert*-butoxyl radical, hydroxyl anion, and iodine. The *in situ* generated iodine reacts with another molecule of TBHP and a hydroxyl ion to afford a *tert*-butylperoxy radical and a molecule of water. Consequently, these radicals abstract a hydrogen atom from the ester and aryl tetrazole resulting in the formation of both carbon C and nitrogen-centered N radical intermediates. Finally, the coupling of carbon C and nitrogen centered radical N affords the desired intermolecular aminated product 1a (Scheme 12).

### Computational studies

To understand the reasons and also to gain additional insights on the selectivity, we have performed DFT computations at (U)M06-2X^24^/6-311G(d,p)^25^ and (U)wB97XD^26^/6-311G(d,p) levels of theory using Gaussian 09.^27^ For the majority of the substrates and their radical isomers, geometries have been optimized at both levels of theory. Furthermore, their relative stability has been compared using bond dissociation energies (BDEs) for deducing the most stable radical among the isomers. Bond dissociation energies and spin densities have been estimated to enumerate the kinetic and thermodynamic stability of the radicals. All these results have been compared, which showed excellent consistency with the experimentally observed selectivity and the yields. In the preceding section, the decisive factors in the selectivity for each type of substrate are discussed based on the computational studies:

#### (a) Unactivated alkanes

**Fig. 1:**
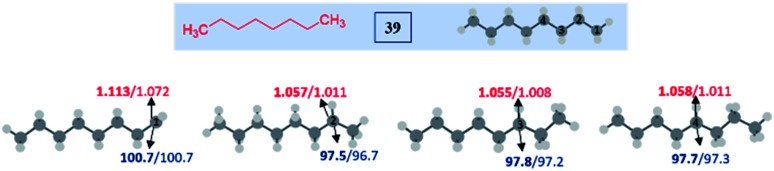
Computed data on the distal selectivity in unactivated alkane (*n*-octane) 39. For each case, spin densities are represented in red and the BDEs (in kcal mol^−1^) are mentioned in blue; bold – (U)M06-2X/6-311G(d,p) and normal font – (U)wB97XD/6-311G(d,p) levels of theory.

In the case of *n*-octane 39, four isomeric radicals are possible, among them, the 2° radicals, namely (2-, 3- and 4-dehydro *n*-octyl radicals) are more stable compared to the 1° radical. Among these, the 2° radicals (having radical centers at C2, C3 and C4) have nearly the same BDEs and spin densities, which makes them equally probable to react; in other words, the reaction did not lead to any selectivity (Fig. 1, and Table S4, see ESI‡).

#### (b) Esters

**Fig. 2:**
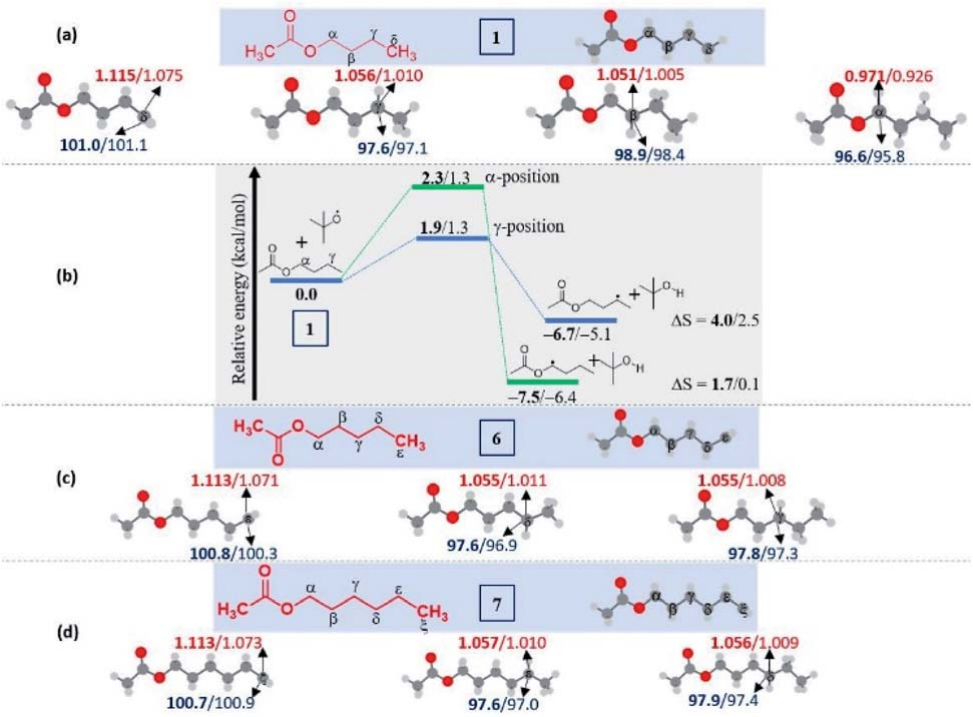
Computed data on the distal selectivity in esters. (a) *n*-butyl acetate 1; (b) energy profile depicting the kinetic favourability of the *γ*-radical over *α*-radical formation in 1 through hydrogen abstraction by the ^*t*^BuO radical (the corresponding transition states are indicated in Fig. S7 in the ESI‡); (c) *n*-pentyl acetate 6 and, (d**)***n*-hexyl acetate 7; (for each case, spin densities are represented in red and the BDEs (in kcal mol^−1^) are mentioned in blue; the energies (in kcal mol^−1^) relative to the reactants, and the thermodynamic entropy changes (in cal K^−1^ mol^−1^) accompanying the reactions are indicated; bold – (U)M06-2X/6-311G(d,p) and normal font – (U)wB97XD/6-311G(d,p) levels of theory).

In the case of *n*-butyl acetate, four carbon-centered radicals are possible in the butyl chain, however, the *α*-radical with the unpaired electron positioned alpha to the ester oxygen is found to be thermodynamically stable on account of the lower spin density and BDE (Fig. 2a), whereas the *δ*-radical is the least stable due to less hyperconjugation. Despite occupying the second in the series among those four isomeric radicals, the *γ*-radical formation is a kinetically preferred channel based on the results at both the levels of theory (Fig. 2b). This has been illustrated from the reaction between the ^*t*^BuO radical and *n*-butyl acetate. Interestingly, a higher positive entropy of the product is also favored by its formation over the *α*-radical. The distal selectivity has also been observed upon increasing the chain length of the ester. In the case of 6 and 7, product selectivity has been restricted to *γ-*/*δ-* and *δ-*/*ε-*, respectively (Fig. 2c and d). Once again, the BDEs and spin densities showed excellent consistency for the distal selectivity and, more importantly, the isolated yields of regioisomers.

**Fig. 3:**
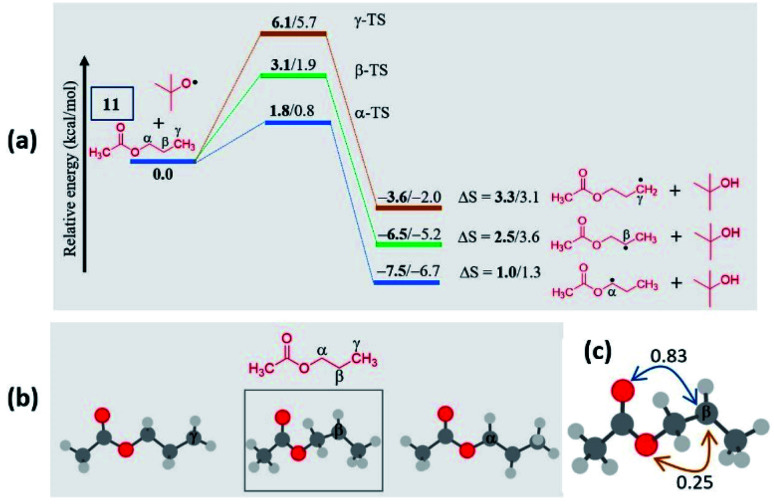
(a) Computed energy profiles depicting the kinetic and thermodynamic favorability of radical formation in 11; the energies relative to the 11 + ^*t*^BuO radical are indicated (in kcal mol^−1^), the thermodynamic entropy changes accompanying the reactions are indicated (in cal K^−1^ mol^−1^); (the corresponding transition states are indicated in Fig. S8 in the ESI‡); (bold – (U)M06-2X/6-311G(d,p) and normal font – (U)wB97XD/6-311G(d,p) levels of theory). (b) Optimized geometries of possible isomeric radical intermediates in *n*-propyl acetate (11) at the (U)M06-2X/6-311G(d,p) level of theory; (c) The second-order perturbation energies (in kcal mol^−1^) from the natural bond orbital (NBO) analysis of the *β*-radical of *n*-propyl acetate (11) at the (U)M06-2X/6-311G(d,p) level of theory.

In the case of *n*-propyl acetate 11 (Scheme 4), computations predict the *α*-radical as the thermodynamically most stable radical as well as kinetically more favored, whereas the *γ*-radical is the least stable among the isomers (Fig. 3a). Besides that, the *γ* radical has the least preference. However, the inspection of the optimized geometries of radicals indicates that both *α* and *γ* radicals attain a zigzag geometry with a planar radical center, whereas the *β* radical attained a twisted geometry upon optimization (Fig. 3b). The attempts to optimize the zigzag *β* radical structure led to a saddle point. To enumerate the reason, we have also performed NBO analysis,^28^ which showed a weak interaction between the radical center and the lone pairs of oxygen (Fig. 3c). Presumably, the twist in the alkyl chain containing the radical center at the *β*-position, along with the lone pairs of oxygen (at the anomeric position) prevents the approach of the tetrazolyl radical to form the N–C bond. On the other hand, if the chain length of the ester increases (for *e.g. n*-butyl acetate), the geometries did not show any twists. So, the distal product selectivity dominates (Fig. 2a). In the case of short-chain esters (ethyl acetate), the *α*-position is favored over the *β*-position, which can be accounted for their thermodynamic stability and the ease of formation of the corresponding radicals (Fig. S5–S7, see ESI‡).

#### (c) Ketones

**Fig. 4:**
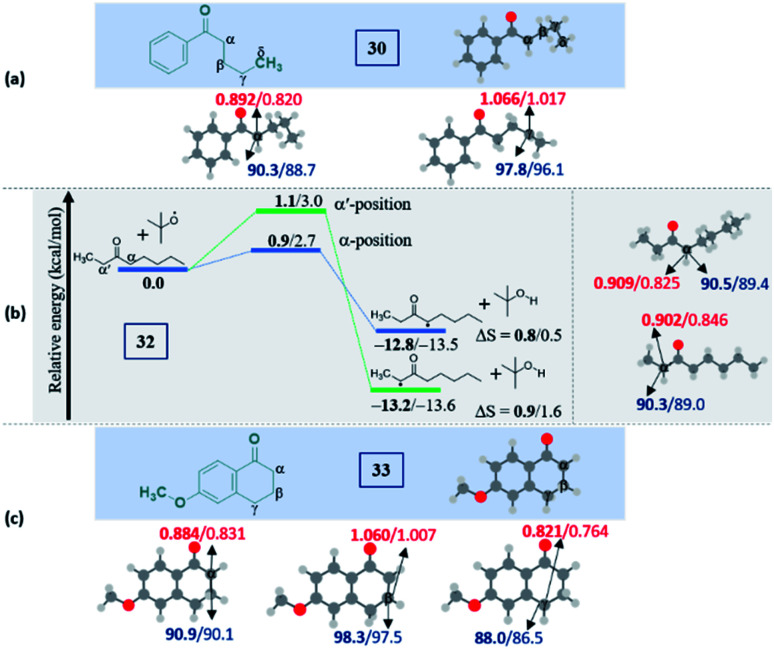
Computed data on the distal selectivity in ketones: (a) valerophenone 30; (b) energy profile depicting the formation of the radicals 32a and 32′a by hydrogen abstraction by the ^*t*^BuO radical from 32 (the corresponding transition states are indicated in Fig. S9 in the ESI‡); (c), 33 (for each case, spin densities are represented in red and the BDEs (in kcal mol^−1^) are mentioned in blue; the energies (in kcal mol^−1^) relative to the reactants, and the thermodynamic entropy changes (in cal K^−1^ mol^−1^) accompanying the reactions are indicated; bold – (U)M06-2X/6-311G(d,p) and normal font – (U)wB97XD/6-311G(d,p) levels of theory).

Unlike esters, the ketones prefer the *α*-selectivity in almost all cases. The preference mainly arises due to the delocalization of the radical character with the carbonyl group, which can be reflected in the lower spin density values at those centers. Apart from the thermodynamic stabilization of the *α*-radical, the lower BDE suggests that the ease of radical formation at the *α*-position over distal positions indicates a kinetic preference. Notably, this can be observed in the case of aryl alkyl ketone 30, where the major product is 30a, an alpha selective product (Fig. 4a). The situation is quite interesting in unsymmetrical ketones with two different alkyl chains as both the *α*-positions are equally favorable. However, the experimental results showed a mixture of products with a marginal preference for the C–N bond formation at the long alkyl chain over the short alkyl chain (Fig. 4b). Despite the spin densities and the BDEs being comparable, a slight kinetic preference (0.2–0.3 kcal mol^−1^) to form a radical at the alpha position to the longer alkyl chain has been predicted. Presumably, lower kinetic favorability led to a mixture of products and a marginal preference for forming 32′a over 32a (Fig. S9, see ESI‡). The importance of radical stability in the selectivity can be envisaged in 33, where the selectivity is not at the *α*-position to the carbonyl. Instead, the major product is found to be *γ*-selective (Fig. 4c). The domination of the resonance stabilization at the *γ*-position over the *α*-position is responsible for such a remarkably high selectivity. Indeed, this situation can be rationalized from the lower spin density and BDE at the *γ*-position than at the *α*-position.

It is noteworthy to mention that the delocalization of spin density and changes in the bond distances between the radical center and the carbonyl carbon (shortening) indicate the stabilization of the alpha radicals in both valerophenone (30) and methylcaproate (24) (Fig. S10, see ESI‡). However, valerophenone and methylcaproate react in a contrasting fashion exhibiting different product selectivities. We presume that the lone pairs of the ester oxygen disfavor the approach of the tetrazole radical in forming the C–N bond. Overall, the kinetic factors associated with the reactions control the selectivity in esters, whereas the thermodynamic stability of the radicals dictates the selectivity in ketones. On the other hand, the unactivated systems did not show any selectivity at all.

## Conclusions

We have developed and rationalized site-selective intermolecular amination *via* CDC at unactivated, non-acidic, remote methylene positions without the aid of any directing group or designer catalysts under metal-free conditions. This site-selective amination takes place with a remarkable level of selectivity with a range of electron-withdrawing groups possessing alkyl chains of various lengths. Unprotected functional groups such as alcohol, amines, amides, and carboxylic acid-containing ionizable hydrogens are unsuccessful substrates for this strategy. Alkyl borated substrates gave identical selectivity giving free amino alcohol where the appended boron atom serves as a traceless directing group which is unprecedented in any remote C_sp^3^_–H functionalization. In a di-alkyl ester, the selectivity is dictated by the –COO– group giving the same *γ* selectivity irrespective of its origin either from the alcohol or carboxy acid. In an appended alkyl chain, the preference is always for the distal carbon. It is best when the position is at the *γ* carbon to the directing group. The site-selectivity is favored at the methine C–H over methylene and methyl of the same length alkyl chain. The degree of distal selectivity decreases as the chain length increases, which is noticeable up to the *ε* position beyond which it is unnoticeable. The ketone and benzylic functionalities bearing longer alkyl chains provided *α*-aminated (proximal) products rather than remote-functionalization, and when the remote site is also benzylic, high yields of products are obtained. Substrates possessing multiple amenable sites undergo selective mono-amination, which allows late-stage functionalization. Linear hydrocarbons having no electronic bias are completely unselective, whereas cyclohexane provided mono aminated products. In this site-selective amination strategy, N-heterocyclic azoles (tetrazole and triazole) and saccharine served as the nitrogen centered radicals. From the control experiments carried out, a radical–radical cross-coupling between carbon and nitrogen center radicals has been proposed for this oxidative C–N bond formation. Based on the DFT computations, such high selectivity has been attributed to the thermodynamic stability (in ketones) or kinetic factors (in esters) associated with the radicals. Furthermore, the unactivated alkanes led to a mixture of products without any selectivity, which corroborates nearly the same stability of various distal radical isomers. Thus, we have rationalized site selectivity amination without the *de novo* approach and found it is solely dependent on the intrinsic reactivity of the substrate.

## Data availability

Optimization of reaction parameters, mechanistic studies, crystallographic description, all experimental procedures, characterisation data, computational details, and copies of ^1^H, ^13^C{^1^H}, ^19^F and, ^31^P NMR spectra for all compounds featured in this manuscript are provided in the ESI.†

## Author contributions

S. R. and B. K. P. conceived, designed and executed the project. S. R. conducted all the experiments and with B. K. P. analysed the data. M. S. and S. V. performed the computational calculations and interpreted the data. S. R., B. K. P., M. S. and S. V. all prepared the manuscript.

## Conflicts of interest

There are no conflicts to declare.

## Supplementary Material

SC-012-D1SC04365J-s001

SC-012-D1SC04365J-s002
