# Supplementary material for: Intermolecular CDC amination of remote and proximal unactivated Csp3–H bonds through intrinsic substrate reactivity – expanding towards a traceless directing group
Source: Chem Sci. 2021 Oct 27;12(46):15318–28. doi: 10.1039/d1sc04365j (PMC8635183; doi:10.1039/d1sc04365j)
Supplement: SC-012-D1SC04365J-s001 [file SC-012-D1SC04365J-s001.pdf]

Electronic Supplementary Material (ESI) for Chemical Science.
This journal is © The Royal Society of Chemistry 2021



# Supporting Information

## Intermolecular CDC Amination of Remote and Proximal Unactivated $C_{sp}^3$–H Bonds Through Intrinsic Substrate Reactivity – Expanding Towards a Traceless Directing Group

List of Contents


1. Figure S1. Biologically active tetrazoles 2
2. Optimization of reaction parameters 2-5
3. Mechanistic studies 5-6
4. Crystallographic description 7-10
5. General information 10-11
6. Experimental section 11-14
7. Procedure for free radical trapping experiment (**1-TEMPO**) 14-15
8. Spectral data 16-48
9. Computational details 48-51
10. References 52-52
11. NMR spectra of all compounds 53-246
12. Electronic and thermodynamic parameters 247-251
13. Cartesian coordinates 252-262

## 1. Figure S1. Biologically active tetrazoles

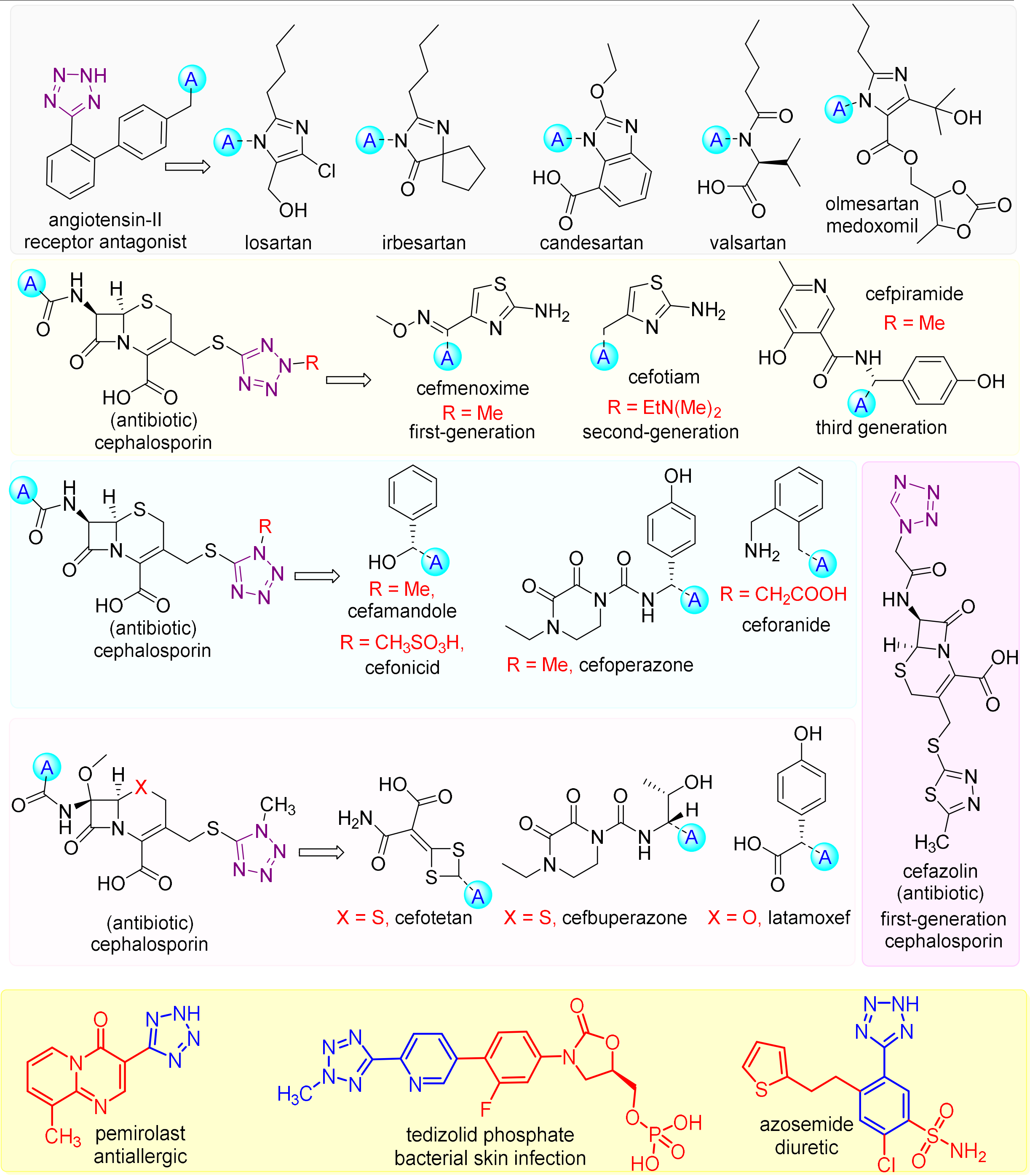


## 2. Optimization of reaction parameters

We set up a reaction at 70 °C between the test candidates, 5-phenyl-2*H*-tetrazole (**a**) and *n*-butyl acetate (**1**) in the presence of an iodide source, $Bu_4NI$ (20 mol %), and oxidant, TBHP (5−6 M in decane) (4 equiv). As anticipated, the site-selective γ-amination of *n*-butyl acetate was achieved, however, the desired product (**1a**) was isolated in a poor yield of 21%. Since the oxidant, TBHP is dissolved in *n*-decane (as solvent), the *in-situ* generated tetrazole radical

reacted with the solvent (*n*-decane), affording an indiscriminate mixture of aminated *n*-decane products in 60% yield, which is quite unsolicited. This preliminary result was quite encouraging, as the remote CDC of *n*-butyl acetate (**1**) with a tetrazole (**a**) evades the involvement of any directing group or designer catalyst. Next, the optimization parameters were scrutinized by varying the reaction temperature, oxidant, catalyst and its loading to improve the reaction efficacy. To our delight, the product yield was enhanced to 47% (Table S1, entry 2) when the above reaction was performed using an aqueous TBHP (70% in water) (4 equiv) instead of a decane solution of TBHP (5–6 M). Decreasing the amount of either TBHP from 4 to 3 equivalent, or $Bu_4NI$ from 20 to 10 mol % (keeping all other parameters constant), resulted in reduced product yield (40% and 41%, respectively) (Table S1, entries 3 and 4). Increasing the amount of $Bu_4NI$ from 20 to 30 mol %, and oxidant from 4 to 5 equivalents, did not lead to any significant enhancement in the product yield (Table S1, entries 5 and 6).

**Table S1** Development of $Bu_4NI$-catalyzed intermolecular remote methylene C–H amination

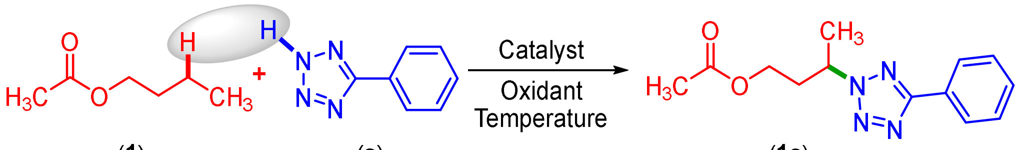


| Entry | Catalyst (mol %) | Oxidant (equiv) | Temp (°C) | Yield (%)$^{b,c}$ |
|---|---|---|---|---|
| 1 | $Bu_4NI$ (20) | dec TBHP (4) | 70 | 21 |
| 2 | $Bu_4NI$ (20) | aq TBHP (4) | 70 | 47 |
| 3 | $Bu_4NI$ (20) | aq TBHP (3) | 70 | 40 |
| 4 | $Bu_4NI$ (10) | aq TBHP (4) | 70 | 41 |
| 5 | $Bu_4NI$ (30) | aq TBHP (4) | 70 | 43 |
| 6 | $Bu_4NI$ (20) | aq TBHP (5) | 70 | 48 |
| 7 | $Bu_4NI$ (20) | aq TBHP (4) | 80 | 56 |
| 8 | $Bu_4NI$ (20) | aq TBHP (4) | 90 | 41 |
| 9$^{d}$ | $Bu_4NI$ (20) | aq TBHP (4) | 80 | 32 |
| 10$^{e}$ | $Bu_4NI$ (20) | aq TBHP (4) | 80 | 58 |
| **11$^{f}$** | **$Bu_4NI$ (10)** | aq **TBHP (2)** | **80** | **69** |
| 12$^{g}$ | $Bu_4NI$ (10) | aq TBHP (2) | 80 | 72 |
| 13 | $Bu_4NF$ (20) | aq TBHP (4) | 80 | n.d |
| 14 | $Bu_4NCl$ (20) | aq TBHP (4) | 80 | n.d |
| 15 | $Bu_4NBr$ (20) | aq TBHP (4) | 80 | 09 |
| 16 | $I_2$ (20) | aq TBHP (4) | 80 | n.d |
| 17 | KI (20) | aq TBHP (4) | 80 | 48 |
| 18 | NaI (20) | aq TBHP (4) | 80 | 40 |
| 19 | CuBr (20) | aq TBHP (4) | 80 | n.r |
| 20 | $CuBr_2$ (20) | aq TBHP (4) | 80 | n.r |

| | | | | |
|---|---|---|---|---|
| 21 | $Cu(OAc)_2$ (20) | aq TBHP (4) | 80 | n.r |
| 22 | $Cu(OTf)_2$ (20) | aq TBHP (4) | 80 | n.r |
| 23 | $Bu_4NI$ (20) | -- | 80 | n.r |
| 24 | -- | aq TBHP (3) | 80 | n.r |

[a]Reaction conditions: *n*-butyl acetate (**1**) (660 µl), phenyl tetrazole (**a**) (0.5 mmol), time 8 h. [b]Isolated yield. [c]All the reactions were carried out in an oxygen-free atmosphere. [d]Oxygen atmosphere. [e]Argon atmosphere. [f]Two iterative addition of oxidant (2 equiv), catalyst (10 mol %) and solvent (330 µl). [g]Three iterative addition of oxidant, catalyst and solvent. n.d = not detected. n.r = no reaction.

At higher temperatures, along with the formation of (**1a**), competitive methylation of tetrazole (**a**) took place giving another product 5-phenyl-2-methyl-tetrazole, where the methyl group is originating from TBHP an observation previously reported by Patel group.[57] The product yield drastically reduced (32%) when the reaction was performed in an oxygen atmosphere (Table S1, entry 9). On the contrary, the yield of the product (**1a**) improved slightly (58%) when the reaction was carried out in an atmosphere of argon (Table S1, entry 10). Interestingly, two iterative addition of oxidant (TBHP), catalyst ($Bu_4NI$), and *n*-butyl acetate at the beginning and after an interval of 2.5 h was found to be beneficial affording the product (**1a**) in 69% yield (Table S1, entry 11). However, three iterative additions (2.5 h intervals) was not so effective as only marginal improvement in the product yield was observed (Table S1, entry 12). *n*-Butyl acetate was used as the reacting partner cum solvent, and hence solvent optimization was not carried out. Choosing a suitable oxidant was crucial in the formation of this aminated product (**1a**): no product was obtained when other oxidants, such as aqueous $H_2O_2$, di-*tert*-butyl peroxide, *tert*-butyl peroxy benzoate, dicumyl peroxide, benzoquinone, Oxone®, $K_2S_2O_8$, and DDQ were used in place of TBHP or cumene peroxide (not shown in Table S1). Different quaternary ammonium salts, such as $Bu_4NF$, $Bu_4NCl$, $Bu_4NBr$ were screened. All the salts and iodine turned out to be quite ineffective, although, with the usage of $Bu_4NBr$, some product was formed (Table S1, entries 13−16). Other inorganic iodide sources, such as KI and NaI provided inferior results (Table S1, entries 17 and 18). Replacing TBAI with Cu-salts that is capable of single-electron transfer, such as CuBr, $CuBr_2$, $Cu(OAc)_2$, and $Cu(OTf)_2$, turned out to be futile (Table S1, entries 19–22). Control experiments performed by the omission of either oxidant (TBHP) or catalyst ($Bu_4NI$) gave no product (Table S1, entries 23 and 24) which suggests the cooperative participation of both these entities. Thus, after extensive exploration, it was revealed that subjecting 5-phenyl-2*H*-tetrazole (**a**) (0.5 mmol) to two iterative addition of 10 mol % of catalyst $Bu_4NI$, 2 equivalent of oxidant (aq TBHP), and

330 µl of *n*-butyl acetate (**1**) at the beginning and after an interval of 2.5 h at 80 °C afforded product (**1a**) in 69% yield after 8 h.

## 3. Mechanistic studies

**Scheme S1. Control experiments to resolve the mechanistic enigma**

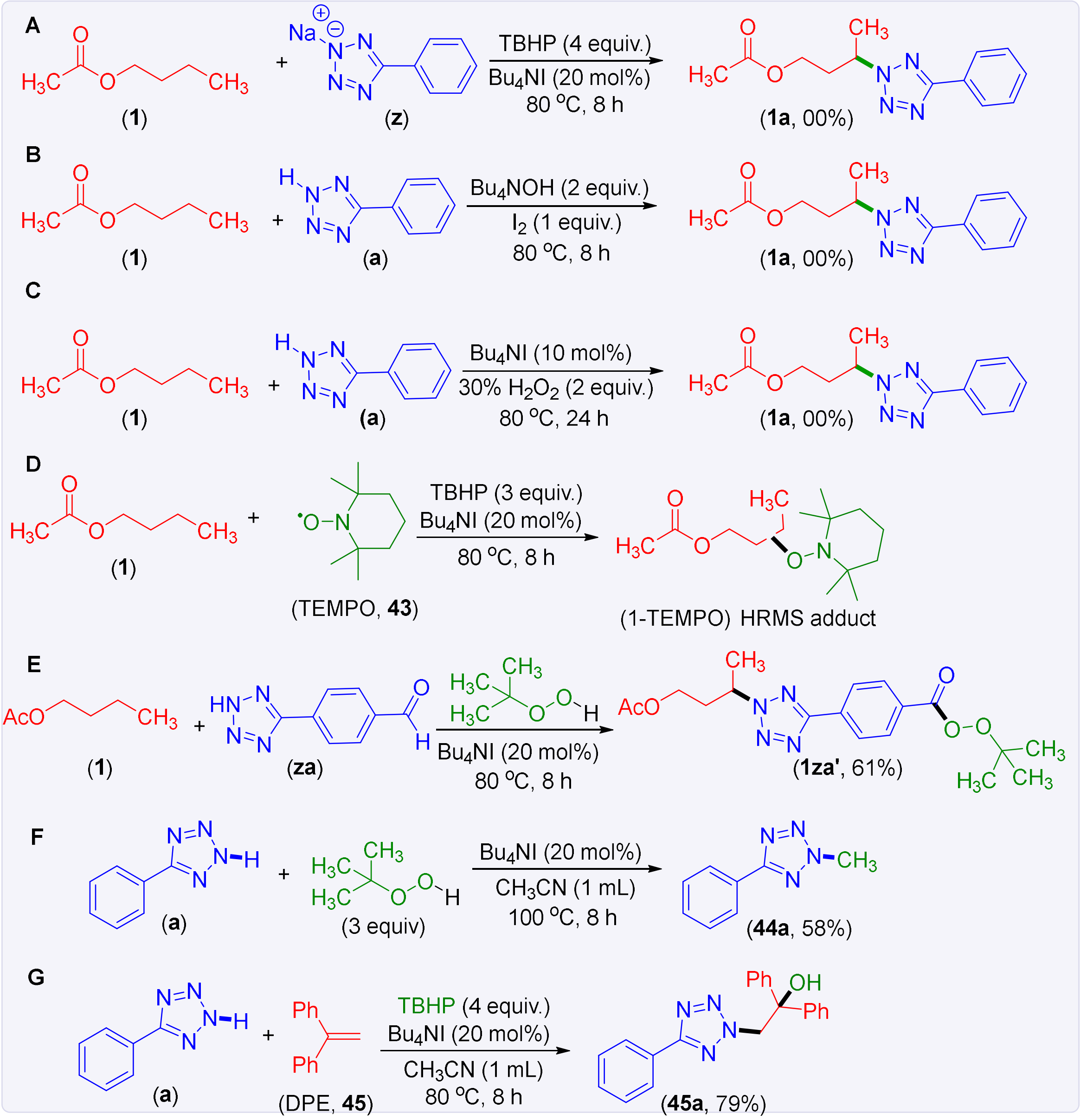


A, non-involvement of the ionic path. B and C, non-involvement of hypoiodate species. D, E, F and G, radical nature of the reaction.

To shed light on the mechanistic pathway of this splendid protocol on site-selective remote intermolecular amination, a series of control experiments were performed. The reaction of *n*-butyl acetate and the sodium salt of tetrazole [sodium 5-phenyltetrazol-2-ide, (**y**)] did not provide the desired product under the standard reaction condition (even in acetonitrile or

DMSO) (Scheme S1A), thereby ruling out any ionic path. The combination of an equivalent of $I_2$ with a stoichiometric amount of base ($Bu_4NOH$) halted the product formation, suggesting non-involvement the of *in-situ* generated tetrabutylammonium hypoiodite $[Bu_4N]^+[IO]^-$, I(I) and its disproportionate products $[Bu_4N]^+[IO_2]^-$, I(III) in the catalytic path. (Scheme S1B).[1,2] Similarly, another hypoiodite species generator from $H_2O_2$ and $Bu_4NI$, also failed to provide the coupled product thereby ruling out any participation of hypoiodite species in this transformation (Scheme S1C).[3] The reaction between *n*-butyl acetate (**1**) and aryl tetrazole (**a**) was considerably hampered in the presence of a radical hunter TEMPO (**43**), giving only 12% yield suggesting the radical nature of the coupling reaction. During the reaction (after 3 hours), *n*-butyl acetate-TEMPO adduct was detected by HRMS analysis of the reaction aliquot. This result confirmed the formation of a radical centre on the ester moiety (Scheme S1D). An aryl tetrazole bearing an aldehydic functionality (**za**) yielded site-selective CDC product (**1za′**) with concurrent per-esterification of aldehyde functionality in a 61% yield (Scheme S1E) possibly *via* a radical-induced path. This result confirmed the involvement of $^t$BuOO radical during the catalytic cycle which is reminiscent of Wan's per-esterification.[4] In yet another control experiment replacement of *n*-butyl acetate (**1**), (which serve the dual role of solvent cum coupling partner) with acetonitrile, 5-phenyl-2*H*-tetrazole (**a**) provided 58% of 2-methyl-5-phenyl-2*H*-tetrazole (**44a**). Here, TBHP serves as a radical methylating agent on to a tetrazolyl radical a fact well documented (Scheme S1F).[5] Again, the radical nature of the tetrazole was further reconfirmed by carrying out the reaction with another radical scavenger diphenyl ethylene (DPE) (**45**), which yielded 79% of DPE-tetrazole coupled adduct (**45a**) (Scheme S1G). A hydroxyl and an *N*-centered radical (NCR) originating respectively from TBHP (**a**) and aryl tetrazole gets incorporated into DPE (**45**) *via* a homolytic cleavage of respective tetrazole N–H and TBHP (Scheme S1G). These observations support the radical-mediated coupling as well as non-involvement of hypoiodite species.

# 4. Crystallographic description

## (i) Crystallographic description of 3-(5-(4-Nitrophenyl)-2*H*-tetrazol-2-yl)butyl acetate (1k).

The compound (**1k**) was crystallized from a supersaturated solution of chloroform by the slow evaporation method. A specimen (**1k**) of $C_{13}H_{15}N_5O_4$, approximate dimensions 0.210 mm x 0.290 mm x 0.350 mm, $M_r$ = 305.30 was used for the X-ray crystallographic analysis. The X-ray intensity data were measured (λ = 0.71073 Å). The integration of the data using a triclinic unit cell yielded a total of 5389 reflections to a maximum θ angle of 29.00° (0.73 Å resolution), of which 3417 were independent (average redundancy 1.577, completeness = 84.8%, $R_{int}$ = 2.54%, $R_{sig}$ = 5.74%) and 1910 (55.90%) were greater than $2\sigma(F^2)$. The final cell constants of a = 7.8987(6) Å, b = 10.2849(12) Å, c = 10.7179(14) Å, α = 116.598(13)°, β = 101.905(9)°, γ = 90.188(8)°, volume = 757.24(17) Å3, are based upon the refinement of the XYZ-centroids of reflections above 20 σ(I). The calculated minimum and maximum transmission coefficients (based on crystal size) are 0.9800 and 0.9850. The structure was solved and refined using the Bruker SHELXTL Software Package, using the space group P -1, with Z = 2 for the formula unit, $C_{13}H_{15}N_5O_4$. The final anisotropic full-matrix least-squares refinement on $F^2$ with 201 variables converged at R1 = 7.43%, for the observed data and wR2 = 25.88% for all data. The goodness-of-fit was 1.024. The largest peak in the final difference electron density synthesis was 0.715 $e^-/Å^3$ and the largest hole was -0.358 $e^-/Å^3$ with an RMS deviation of 0.065 $e^-/Å^3$. On the basis of the final model, the calculated density was 1.339 g/cm$^3$ and F(000), 320 $e^-$. CCDC-2077948 for 3-(5-(4-Nitrophenyl)-2*H*-tetrazol-2-yl)butyl acetate (**1k**) contains the supplementary crystallographic data for this paper. These data can be obtained free of charge from The Cambridge Crystallographic Data Centre *via* www.ccdc.cam.ac.uk/data_request/cif.

**Table S2. Sample and crystal data for (1k)**

| | | |
|---|---|---|
| Identification code | RSHBuOAcNO$_2$ | |
| CCDC | 2077948 | |
| Chemical formula | $C_{13}H_{15}N_5O_4$ | |
| Formula weight | 305.30 g/mol | |
| Temperature | 293(2) K | |
| Wavelength | 0.71073 Å | |
| Crystal size | 0.210 x 0.290 x 0.350 mm | |
| Crystal system | triclinic | |
| Space group | P -1 | |
| Unit cell dimensions | a = 7.8987(6) Å | α = 116.598(13)° |
| | b = 10.2849(12) Å | β = 101.905(9)° |

| | | |
|---|---|---|
| | c = 10.7179(14) Å | γ = 90.188(8)° |
| Volume | 757.24(17) $Å^3$ | |
| Z | 2 | |
| Density (calculated) | 1.339 $g/cm^3$ | |
| Absorption coefficient | 0.102 $mm^{-1}$ | |
| Reflections collected | 5389 | |
| Independent reflections | 3417 [R(int) = 0.0254] | |
| Goodness-of-fit on $F^2$ | 1.024 | |

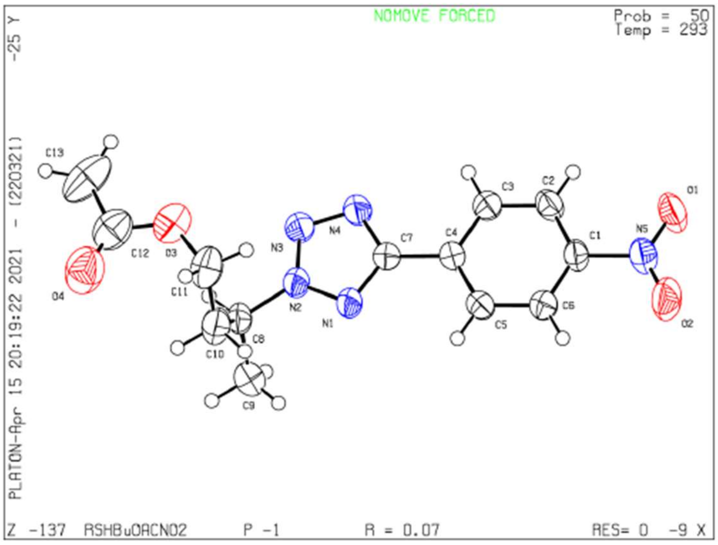


**Figure S2.** ORTEP view of 3-(5-(4-Nitrophenyl)-2*H*-tetrazol-2-yl)butyl acetate (**1k**) with ellipsoids at 50% probability.

**(ii) Crystallographic description of 2-(3-(5-(4-Nitrophenyl)-2*H*-tetrazol-2-yl)butyl)isoindoline-1,3-dione** (**15k**)

The compound (**15k**) was crystallized by vapor diffusion crystallizations method using methanol as inner vial solvent and di-ethyl-ether as outer vial solvent. A specimen of $C_{19}H_{16}N_6O_4$, approximate dimensions 0.210 mm x 0.220 mm x 0.310 mm, $M_r$ = 392.38, was used for the X-ray crystallographic analysis. The X-ray intensity data were measured ($\lambda$ = 0.71073 Å). The integration of the data using an orthorhombic unit cell yielded a total of 20256 reflections to a maximum θ angle of 28.42 (0.75 Å resolution), of which 4435 were independent (average redundancy 4.567, completeness = 98.0%, $R_{int}$ = 5.54%, $R_{sig}$ = 7.85%) and 2574 (58.04%) were greater than $2\sigma(F^2)$. The final cell constants of a = 7.6825(8) Å, b = 13.3272(13) Å, c = 18.012(2) Å, volume = 1844.2(3) $Å^3$, are based upon the refinement of the XYZ-centroids of reflections above 20 σ(I). The calculated minimum and maximum transmission coefficients (based on crystal size) are 0.9730 and 0.9790. The structure was

solved and refined using the Bruker SHELXTL Software Package, using the space group P n a 21, with Z = 4 for the formula unit, $C_{19}H_{16}N_6O_4$. The final anisotropic full-matrix least-squares refinement on $F^2$ with 263 variables converged at R1 = 4.55%, for the observed data and wR2 = 10.88% for all data. The goodness-of-fit was 1.024. The largest peak in the final difference electron density synthesis was 0.106 $e^-/Å^3$ and the largest hole was -0.138 $e^-/Å^3$ with an RMS deviation of 0.030 $e^-/Å^3$. On the basis of the final model, the calculated density was 1.413 g/cm$^3$ and F(000), 816 $e^-$. CCDC-2070229 for 2-(3-(5-(4-Nitrophenyl)-2*H*-tetrazol-2-yl)butyl)isoindoline-1,3-dione (**15k**) contains the supplementary crystallographic data for this paper. The data can be obtained free of charge from The Cambridge Crystallographic Data Centre *via* www.ccdc.cam.ac.uk/data_request/cif.

**Table S3. Sample and crystal data for (15k)**

| | | |
|---|---|---|
| Identification code | RSH_30012021 | |
| CCDC | 2070229 | |
| Chemical formula | $C_{19}H_{16}N_6O_4$ | |
| Formula weight | 392.38 g/mol | |
| Temperature | 293(2) K | |
| Wavelength | 0.71073 Å | |
| Crystal size | 0.210 x 0.220 x 0.310 mm | |
| Crystal system | orthorhombic | |
| Space group | P n a 21 | |
| Unit cell dimensions | a = 7.6825(8)Å | α = 90° |
| | b = 13.3272(13) Å | β = 90° |
| | c = 18.012(2) Å | γ = 90° |
| Volume | 1844.2(3) Å$^3$ | |
| Z | 4 | |
| Density (calculated) | 1.413g/cm$^3$ | |
| Absorption coefficient | 0.103 mm$^{-1}$ | |
| Reflections collected | 20256 | |
| Independent reflections | 4435 [R(int) = 0.0554] | |
| Goodness-of-fit on $F^2$ | 1.024 | |

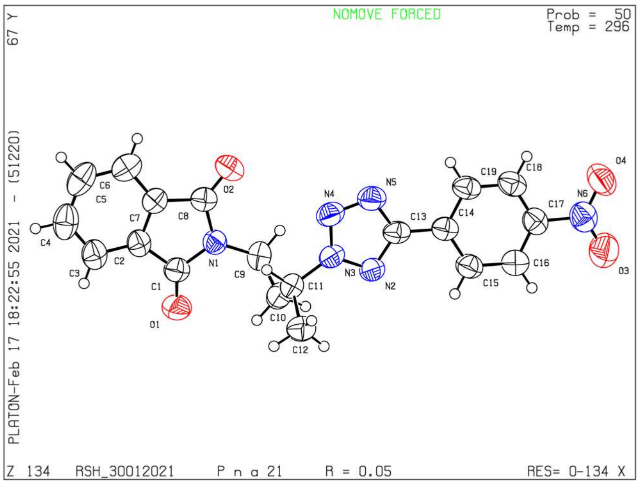


**Figure S3.** ORTEP view of 2-(3-(5-(4-Nitrophenyl)-2*H*-tetrazol-2-yl)butyl)isoindoline-1,3-dione (**15k**) with ellipsoids at 50% probability.

## 5. General information

All the reactions were carried out in scrupulously clean and oven-dried glassware carefully protected from exposure to atmospheric moisture oxygen under a degassed atmosphere unless otherwise stated. Highest-commercial-quality reagents were purchased and used without further purification unless otherwise stated. Reactions were monitored by thin-layer chromatography (TLC) carried out on a 0.25 mm silica gel plates ($60F_{254}$) visualized under UV illumination at 254 nm. Further visualization was achieved by iodine vapor adsorbed on silica gel, basic solution of potassium permanganate ($KMnO_4$) and heat, an acidic solution of vanillin and heat, ninhydrin solution and heat, PMA and heat as developing agents depending on the product type. Organic extracts were dried over anhydrous sodium sulfate. Solvents were removed using a rotary evaporator under reduced pressure. Yields refer to chromatographically homogeneous material unless otherwise stated. Column chromatography was performed on silica gel 60−120 or 100−200 mesh using a mixture of hexane and ethyl acetate as eluent. Isolated compounds were characterized by $^{1}H$ and $^{13}C$ {$^{1}H$} NMR, $^{19}F$ NMR, $^{31}P$ NMR and IR spectroscopic, HRMS-spectrometric techniques.

NMR spectra for all the samples were measured in deuterochloroform ($CDCl_3$). NMR spectra were recorded at ambient temperature in either 400 or 600 MHz for $^{1}H$ NMR and 101 or 151 MHz for $^{13}C$ {$^{1}H$} NMR, 565 or 377 MHz for $^{19}F$ NMR and 162 MHz for $^{31}P$ NMR. All the spectra were calibrated using tetramethylsilane or residual undeuterated solvent for $^{1}H$ NMR, deuterochloroform for $^{13}C$ NMR as an internal reference {$Si(CH_3)_4$ : 0.00 ppm or $CHCl_3$:

7.23 ± 0.03 ppm for $^{1}$H NMR and 77.16 ± 0.06 ppm for $^{13}$C NMR}. $^{19}$F NMR and $^{31}$P NMR were calibrated using hexafluorobenzene and phosphoric acid as an internal standard. The chemical shifts are quoted in *δ* units, parts per million (ppm). $^{1}$H NMR data is represented as follows: Chemical shift (from downfield to upfield, towards the signal of internal standard TMS), multiplicity (s = singlet, d = doublet, t = triplet, q = quartet, m = multiplet, br = broad, p = pentet, h = hexcept, dd = doublet of doublet, dt = doublet of triplet, dq = doublet of quartet, td = triplet of doublet, tq = triplet of quartet, tt = triplet of triplet, qd = quartet of doublet, qt = quartet of triplet, ddd = doublet of doublet of doublet, ddt = doublet of doublet of triplet), coupling constant(s) *J* in Hertz (Hz) and integration. High-resolution mass spectra (HRMS) were recorded on a mass spectrometer using electrospray ionization-time of flight (ESI-TOF) reflectron experiments or atmospheric pressure chemical ionization (APCI). FT-IR spectra were recorded in KBr or neat and reported in the frequency of absorption (cm$^{-1}$). Solvents ($CH_3CN$, DMSO) were purchased in HPLC grade passed through activated alumina column, degassed by purging thoroughly with argon.

## 6. Experimental section

**General procedure for the synthesis of aryl tetrazole** (**a–q**). These aryl tetrazoles can be prepared following the literature procedure.[6] However, we have adopted the following modified procedure:In a 50 mL round bottom flask, sodium azide (4.87 g, 75 mmol, 1.5 equiv) was added to a magnetically stirred solution of benzonitrile (5.16 g, 50 mmol, 1 equiv) in anhydrous DMF (3 mL). Then, portion-wise, ceric ammonium nitrate (CAN), (2.74 g, 5 mmol, 0.1 equiv) was added to the above reaction mixture. [***CAUTION***: *Addition of CAN in the reaction medium is highly exothermic*]. The reaction mixture was allowed to reflux for 24 h at 110 °C with vigorous stirring. After the completion of reaction as judge by TLC, the reaction mixture was brought to room temperature and dissolved in ethyl acetate (2 X 100 mL) and the solution was washed with 4 M HCl solution (2 X 100 mL). The organic layer was separated, dried over anhydrous $Na_2SO_4$, filtered and the solvent was removed under reduced pressure to obtain 5-phenyl-2*H*-tetrazole (**a**) (6.58 g, yield of 90%). The same procedure adopted for preparation of other aryl tetrazole derivative also.

**Method A: General procedure for the synthesis of remote intermolecular amination products (Scheme 1-5).** To a 10 mL double-neck round bottom flask was charged with a stirring bar, azole (5-phenyl-2*H*-tetrazole) (73 mg, 0.5 mmol, 1 equiv) and tetrabutylammonium iodide ($Bu_4NI$) (19 mg, 0.05 mol, 0.1 equiv).[a] One neck of the round bottom flask was capped with a rubber septum and another neck equipped with a reflux condenser. The neck of the reflux condenser was attached with a two-way stopcock. One way was fitted with an argon balloon and the other way was attached to a vacuum pump. The reaction setup was flushed with argon gas and evacuated using a vacuum pump through an appropriately fitted needle (three repeats). In this reaction container 5 equivalent of appropriate degassed solvent[b,c] (*n*-butyl acetate, 330 µl) and 2 equivalent of 70% aqueous TBHP (136 µl, 1 mmol, 2 equiv) were injected *via* the rubber septum. The reaction setup was transferred to an oil bath and the temperature was raised to 80 °C from room temperature by increasing the temperature at the rate of 5 °C/ minute and maintained for 2.5 h. After 2.5 h of stirring at 80 °C, the reaction mixture was lifted from the oil bath. The reaction container was degassed gently using a two-way stopcock and a vacuum pump. Separately in a dram borosilicate vial (2 mL) 10 mol % of tetrabutylammonium iodide ($Bu_4NI$) (19 mg, 0.05 mol, 0.1 equiv) and TBHP (136 µl, 1 mmol, 2 equiv) were added. The vial was capped with rubber septum degassed and flushed with argon. To this vial, 230 µl of degassed solvent[b,c] (*n*-butyl acetate) was added, the whole catalyst, oxidant and starting material (solvent) combination were slowly transferred to the reaction flask *via* a cannula. The catalyst vial was rinsed with 100 µl of solvent (*n*-butyl acetate) and transferred to the reaction flask *via* a cannula. The combined reaction mass was again heated at 80 °C for 6 hours. The reaction mixture was cooled to room temperature. Stopcock, condenser and septum were dismantled. The reaction mass was diluted with ethyl acetate (30 mL) and washed with 10% aqueous solution of sodium thiosulfate (30 mL). The organic layer was dried over sodium sulfate, filtered and concentrated under reduced pressure. The crude reaction mass was purified over a silica gel column chromatography using ethyl acetate and hexane as the eluents to provide 69% (90 mg) of the intermolecular aminated product (**1a**).

***Note:*** *[a]0.5 mmol of staring material* ***14*** *(solid) and substrates* ***12, 13, 15–23*** *and* ***26*** *(liquid) which are incapable to solubilize the azole) were added along with tetrabutylammonium iodide. [b]Starting materials such as esters* ***(1–11, 24)****, nitrile* ***(25)*** *and phosphate* ***(27)*** *were used as the starting material cum solvent. [c]For starting materials* ***12–23, 26, 28*** *and* ***29*** *acetonitrile (1 mL) was used as the solvent.*

**Method B: General procedure for the synthesis of intermolecular α–amination products and late-stage amination (Scheme 6, 11).** To an oven-dried 10 mL double-neck round bottom flask, magnetic stirring bar, 5-phenyl-2*H*-tetrazole (73 mg, 0.5 mmol, 1 equiv), tetrabutylammonium iodide ($Bu_4NI$) (37 mg, 10 mol %, 0.2 equiv) and 1 equivalent of appropriate starting material (**30, 32–34, 41 and 42**) were added.[a-c] One neck of the round bottom flask was capped with a rubber septum and another neck equipped with a reflux condenser. The neck of the reflux condenser was attached with a two-way stopcock. One way was fitted with an argon balloon and the other way with a vacuum pump. The reaction setup was flushed with argon gas and degassed (three repeats). Then, in 4 equivalents of aqueous TBHP (272 μl, 2 mmol) 1 mL of degassed acetonitrile were injected. The reaction flask was transferred into an oil bath and the temperature was raised to 80 °C from room temperature by increasing the temperature at the rate of 5 °C/ minute and the reaction content was allowed to stir for 6 hours at 80 ºC. Then the reaction mixture was cooled to room temperature and acetonitrile was removed under reduced pressure. The crude reaction mass was diluted with ethyl acetate (30 mL) and work up with a 10% aqueous solution of sodium thiosulphate (30 mL). The organic layer was collected and dried over anhydrous sodium sulfate, filtered and concentrated under vacuum pressure. The crude reaction mixture was purified over a silica column chromatography to provide 80% (123 mg) a spectroscopically pure product. ([a]Note: For **31a**, the reaction was performed in a pressure tube, acetone (**31**) was used as starting material cum solvent. [b]Estrone derivative (**41**) functionalization was carried out in 0.25 mmol scale. [c]Sulbactam (**42**) derivative functionalization was carried out in 1 mmol scale.

**Method C: general procedure for the synthesis of intermolecular amination of borate esters products (Scheme 9).** To an oven-dried 10 mL double-neck round bottom flask, a magnetic stirring bar, aryl tetrazole (5-phenyl-*2H*-tetrazole) (73 mg, 0.5 mmol, 1 equiv) and $Bu_4NI$ (37 mg, 10 mol %, 0.2 equiv) were added. One neck of the round bottom flask was capped with a rubber septum and another neck equipped with a reflux condenser. The neck of the reflux condenser was attached with a two-way stopcock. One way was fitted with an argon balloon and the other way was attached to a vacuum pump. The reaction setup was flushed with argon gas and degassed (three repeats). Then, tributyl borate (**35**) (0.5 mmol, 1 equiv) and 5 equivalents of THHP (2.5 mmol) were mixed in 1 mL of degassed $CH_3CN$, and injected into the reaction mass. The reaction container was transferred into an oil bath and the temperature

was raised to 80 °C from room temperature by increasing the temperature at the rate of 5 °C/ minute and the reaction content was allowed to stir for 8 hours at 80 ºC. The reaction mixture was cooled to room temperature. Acetonitrile was removed under a reduced pressure, then the resulting reaction mass was admixed with ethyl acetate (30 mL) and work up with 10% aqueous solution of sodium thiosulphate (30 mL) and then with a brine solution (30 mL). The organic layer was dried over anhydrous $Na_2SO_4$, filtered and concentrated under reduced pressure. The crude reaction mass was purified over a silica gel column using ethyl acetate and hexane as the eluents to provide 63% (69 mg) aminated alcohol product (3-(5-Phenyl-2*H*-tetrazol-2-yl)butan-1-ol) (**35a**).

**Method D: General procedure for the synthesis of intermolecular amination of cyclohexane products (Scheme 10).** To an oven-dried 10 mL double-neck round bottom flask, a magnetic stirring bar, aryl tetrazole (5-phenyl-2*H*-tetrazole) (73 mg, 0.5 mmol, 1 equiv) and $Bu_4NI$ (37 mg, 10 mol %, 0.2 equiv) were added. One neck of the round bottom flask was capped with a rubber septum and another neck equipped with a reflux condenser. The neck of the reflux condenser was attached with a two-way stopcock. One way was fitted with an argon balloon and the other way was attached to a vacuum pump. The reaction setup was flushed with argon gas and degassed (three repeats). Then, 1 mL of degassed DMSO, afterwards, *n*-octane or cyclohexane (1 mmol, 2 equiv) and 4 equivalents of aqueous TBHP (272 µl, 2 mmol) were injected. The reaction container was transferred into an oil bath and the temperature was raised to 80 °C from room temperature by increasing the temperature at the rate of 5 °C/ minute and the reaction content was allowed to stir for 6 hours at 80 ºC. The reaction mixture was cooled to room temperature. Then, the reaction mixture was admixed with ethyl acetate (30 mL) and work up with 10% aqueous solution of sodium thiosulphate (30 mL) and then with a brine solution (30 mL). The organic layer was dried over anhydrous $Na_2SO_4$, filtered and concentrated under reduced pressure. The crude reaction mass was purified over a silica gel column using ethyl acetate and hexane as the eluents to provide 76% (87 mg) tetrazole-*N*-alkylated product (2-cyclohexyl-5-phenyl-2*H*-tetrazole) (**40a**).

## 7. Procedure for free radical trapping experiment (1-TEMPO)

Identical to general experimental procedure on remote intermolecular amination protocol provided in the main manuscript (Method A), 5-phenyl-2*H*-tetrazole (73 mg, 0.5 mmol, 1 equiv) and tetrabutylammonium iodide ($Bu_4NI$) (37 mg, 10 mol %, 0.2 equiv), 1 mL of *n*-butyl acetate and 4 equiv of 70% aqueous TBHP (272 µL, 2 mmol) but the reaction was

carried out in the presence of 2 equivalent of TEMPO. After 60 minutes of stirring at 80 ºC an aliquot (50 µL) of reaction mixture was taken out *via* septum using syringe, the aliquot was diluted with HPLC grade acetonitrile and filtered using syringe filter (20 micron). The filtrate was directly injected into HRMS probe. The TEMPO-butyl acetate adduct (**1-TEMPO**) was detected in HRMS probe.

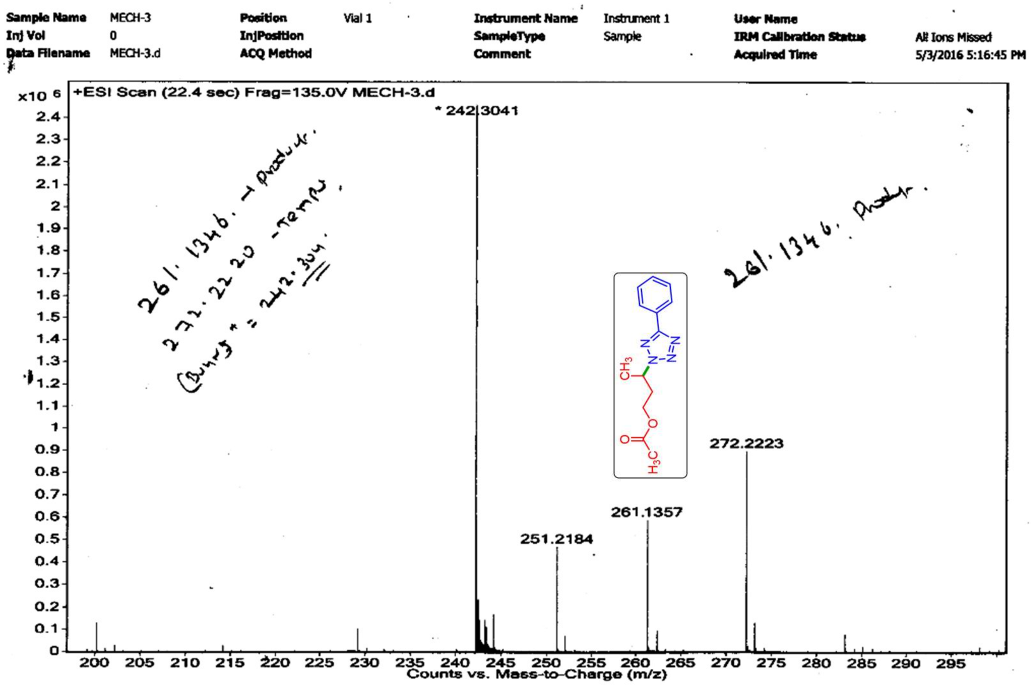


**Figure S4.** *Detection of radical intermediate (**1-TEMPO**) in HRMS probe*

## 8. Spectral data

### 3-(5-Phenyl-2*H*-tetrazol-2-yl)butyl acetate (1a)

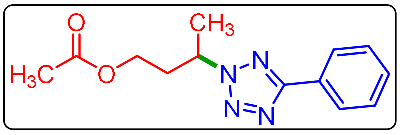



The title compound was obtained according to the general procedure (Method A). Gummy (90 mg, 69% yield); $R_f$ = 0.45 (2:8 EtOAc:hexane, silica gel); $^1$H NMR (600 MHz, $CDCl_3$): $\delta$ 8.14 (dd, $J$ = 7.8, 1.5 Hz, 2H), 7.49–7.44 (m, 3H), 5.19–5.13 (m, 1H), 4.15–4.10 (m, 1H), 3.97 (ddd, $J$ = 12.0, 7.7, 4.8 Hz, 1H), 2.53–2.45 (m, 1H), 2.26 (ddt, $J$ = 14.7, 7.7, 5.1 Hz, 1H), 1.98 (s, 3H), 1.71 (d, $J$ = 6.8 Hz, 3H) ppm; $^{13}$C {$^1$H} NMR (151 MHz, $CDCl_3$): $\delta$ 170.8, 165.1, 130.4, 129.0, 127.6, 126.9, 60.8, 58.0, 35.1, 20.9, 20.8 ppm; IR (KBr): 3075, 3000, 2964, 2925, 2848, 1742, 1529, 1467, 1450, 1368, 1233, 1184, 1086, 1042, 1023, 1007, 941, 852, 789, 733, 693, 595 cm$^{-1}$; HRMS (ESI-TOF) *m/z:* [M + H]$^+$ calcd for $C_{13}H_{17}N_4O_2$, 261.1346; found, 261.1359.

### 3-(5-(Naphthalen-2-yl)-2*H*-tetrazol-2-yl)butyl acetate (1b)

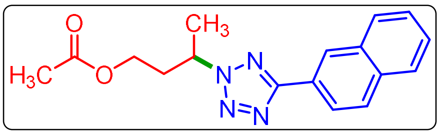



The title compound was obtained according to the general procedure (Method A). Gummy (98 mg, 63% yield); $R_f$ = 0.48 (2:8 EtOAc:hexane, silica gel); $^1$H NMR (600 MHz, $CDCl_3$): $\delta$ 8.69 (s, 1H), 8.22 (dd, $J$ = 8.5, 1.5 Hz, 1H), 7.95 (d, $J$ = 8.0 Hz, 2H), 7.90–7.85 (m, 1H), 7.57–7.51 (m, 2H), 5.23–5.17 (m, 1H), 4.16 (ddd, $J$ = 11.6, 6.5, 5.2 Hz, 1H), 4.02 (ddd, $J$ = 11.9, 7.6, 4.8 Hz, 1H), 2.57–2.50 (m, 1H), 2.29 (ddt, $J$ = 14.7, 7.6, 5.1 Hz, 1H), 1.98 (s, 3H), 1.75 (d, $J$ = 6.8 Hz, 3H) ppm; $^{13}$C {$^1$H} NMR (151 MHz, $CDCl_3$): $\delta$ 170.8, 165.2, 134.3, 133.3, 128.83, 128.77, 127.9, 127.2, 126.77, 126.71, 124.9, 124.0, 60.8, 58.1, 35.1, 20.9, 20.8 ppm; IR (KBr): 3057, 2985, 2936, 1745, 1603, 1522, 1500, 1450, 1437, 1368, 1232, 1138, 1037, 944, 863, 824, 772, 632, 604 cm$^{-1}$; HRMS (ESI-TOF) *m/z:* [M + H]$^+$ calcd for $C_{17}H_{19}N_4O_2$, 311.1503; found, 311.1494.

### 3-(5-([1,1'-Biphenyl]-4-yl)-2*H*-tetrazol-2-yl)butyl acetate (1c)

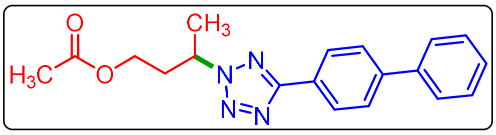



The title compound was obtained according to the general procedure (Method A). Gummy (103 mg, 61% yield); $R_f$ = 0.55 (2:8 EtOAc:hexane, silica gel); $^1$H NMR (600 MHz, $CDCl_3$): $\delta$ 8.22 (d, $J$ = 8.4 Hz, 2H), 7.73 (d, $J$ = 8.4 Hz, 2H), 7.67–7.64 (m, 2H), 7.47 (t, $J$ = 7.7 Hz, 2H), 7.38 (t, $J$ = 7.4 Hz, 1H), 5.23–5.14 (m, 1H), 4.15 (ddd, $J$ = 11.7, 6.5, 5.2 Hz, 1H), 4.00 (ddd, $J$ = 12.1, 7.6, 4.8 Hz, 1H), 2.54–2.48 (m, 1H), 2.28 (ddt, $J$ = 14.7, 7.6, 5.1 Hz, 1H), 1.99 (s, 3H), 1.73 (d, $J$ =

6.9 Hz, 3H) ppm; $^{13}C$ {$^{1}H$} NMR (151 MHz, $CDCl_3$): $\delta$ 170.9, 164.9, 143.1, 140.4, 129.0, 127.9, 127.7, 127.4, 127.2, 126.5, 60.8, 58.1, 35.1, 20.9, 20.8 ppm; IR (KBr): 3059, 3032, 2983, 2968, 2934, 2850, 1748, 1617, 1599, 1463, 1450, 1418, 1367, 1232, 1138, 1038, 850, 751, 723, 698, 605 $cm^{-1}$; HRMS (ESI-TOF) *m/z:* $[M + H]^+$ calcd for $C_{19}H_{21}N_4O_2$, 337.1659; found, 337.1661.

**3-(5-(4-Methoxyphenyl)-2*H*-tetrazol-2-yl)butyl acetate (1e)**

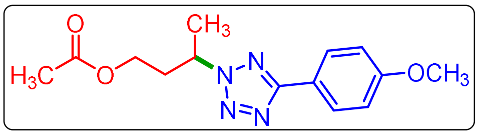


The title compound was obtained according to the general procedure (Method A). Gummy (85 mg, 58% yield); $R_f$ = 0.29 (2:8 EtOAc:hexane, silica gel); $^{1}H$ NMR (600 MHz, $CDCl_3$): $\delta$ 8.04 (d, $J$ = 8.3 Hz, 2H), 6.97 (d, $J$ = 8.4 Hz, 2H), 5.11 (dq, $J$ = 13.5, 6.7 Hz, 1H), 4.09 (dt, $J$ = 11.5, 5.7 Hz, 1H), 3.93 (ddd, $J$ = 11.9, 7.4, 5.0 Hz, 1H), 3.83 (s, 3H), 2.48–2.41 (m, 1H), 2.24–2.18 (m, 1H), 1.95 (s, 3H), 1.67 (d, $J$ = 6.8 Hz, 3H) ppm; $^{13}C$ {$^{1}H$} NMR (151 MHz, $CDCl_3$): $\delta$ 170.9, 165.0, 161.3, 128.5, 120.2, 114.4, 60.8, 57.9, 55.5, 35.1, 20.9, 20.8 ppm; IR (KBr): 2989, 2956, 2928, 2839, 1741, 1615, 1590, 1538, 1464, 1368, 1321, 1252, 1173, 1105, 1029, 841, 794, 699, 606, 530 $cm^{-1}$; HRMS (ESI-TOF) *m/z:* $[M + H]^+$ calcd for $C_{14}H_{19}N_4O_3$, 291.1452; found, 291.1444.

**3-(5-(*p*-Tolyl)-2*H*-tetrazol-2-yl)butyl acetate (1d)**

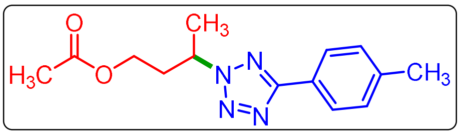


The title compound was obtained according to the general procedure (Method A). Gummy (90 mg, 65% yield); $R_f$ = 0.54 (2:8 EtOAc:hexane, silica gel); $^{1}H$ NMR (600 MHz, $CDCl_3$): $\delta$ 8.00 (d, $J$ = 7.8 Hz, 2H), 7.26 (d, $J$ = 7.8 Hz, 2H), 5.13 (h, $J$ = 6.7 Hz, 1H), 4.10 (dt, $J$ = 11.5, 5.7 Hz, 1H), 3.94 (ddd, $J$ = 11.8, 7.4, 5.1 Hz, 1H), 2.49–2.42 (m, 1H), 2.38 (s, 3H), 2.25–2.19 (m, 1H), 1.95 (s, 3H), 1.68 (d, $J$ = 6.8 Hz, 3H) ppm; $^{13}C$ {$^{1}H$} NMR (151 MHz, $CDCl_3$): $\delta$ 170.9, 165.2, 140.6, 129.7, 126.9, 124.8, 60.8, 57.9, 35.1, 21.6, 20.9, 20.8 ppm; IR (KBr): 2984, 2956, 2920, 2848, 1742, 1618, 1546, 1461, 1368, 1335, 1232, 1179, 1107, 1037, 1021, 829, 757, 677, 605, 517 $cm^{-1}$; HRMS (ESI-TOF) *m/z:* $[M + H]^+$ calcd for $C_{14}H_{19}N_4O_2$, 275.1503; found, 275.1500.

**3-(5-(4-(*tert*-Butyl)phenyl)-2*H*-tetrazol-2-yl)butyl acetate (1f)**

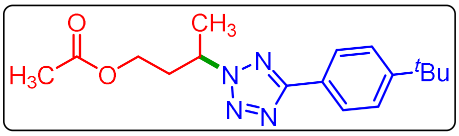


The title compound was obtained according to the general procedure (Method A). Gummy (98 mg, 62% yield); $R_f$ = 0.66 (2:8 EtOAc:hexane, silica gel); $^{1}H$

NMR (600 MHz, $CDCl_3$): $\delta$ 8.03 (d, $J$ = 8.1 Hz, 2H), 7.47 (d, $J$ = 8.1 Hz, 2H), 5.13 (dq, $J$ = 14.5, 7.1 Hz, 1H), 4.09 (dt, $J$ = 11.8, 5.9 Hz, 1H), 3.96–3.89 (m, 1H), 2.48–2.42 (m, 1H), 2.24–2.19 (m, 1H), 1.95 (s, 3H), 1.67 (d, $J$ = 7.0 Hz, 3H), 1.32 (s, 9H) ppm; $^{13}C$ {$^{1}H$} NMR (151 MHz, $CDCl_3$): $\delta$ 170.9, 165.1, 153.7, 126.7, 125.9, 124.8, 60.8, 57.9, 35.1, 34.9, 31.3, 20.9, 20.8 ppm; IR (KBr): 2963, 2870, 1743, 1620, 1542, 1463, 1422, 1366, 1231, 1142, 1037, , 845, 767, 640, 603, 563 $cm^{-1}$; HRMS (ESI-TOF) *m/z:* $[M + H]^+$ calcd for $C_{17}H_{25}N_4O_2$, 317.1972; found, 317.1977.

**3-(5-(3-Methoxyphenyl)-2*H*-tetrazol-2-yl)butyl acetate (1g)**

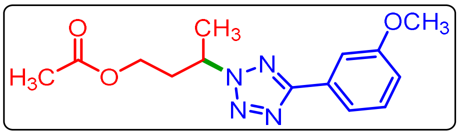


The title compound was obtained according to the general procedure (Method A). Gummy (89 mg, 61% yield); $R_f$ = 0.34 (2:8 EtOAc:hexane, silica gel); $^{1}H$ NMR (600 MHz, $CDCl_3$): $\delta$ 7.72 (d, $J$ = 7.7 Hz, 1H), 7.67 (s, 1H), 7.38 (t, $J$ = 8.1 Hz, 1H), 7.00 (d, $J$ = 9.6 Hz, 1H), 5.16 (dq, $J$ = 13.5, 6.8 Hz, 1H), 4.11 (dt, $J$ = 11.6, 5.8 Hz, 1H), 3.96 (ddd, $J$ = 12.0, 7.2, 4.8 Hz, 1H), 3.88 (s, 3H), 2.51–2.44 (m, 1H), 2.29–2.20 (m, 1H), 1.97 (s, 3H), 1.70 (d, $J$ = 6.9 Hz, 3H) ppm; $^{13}C$ {$^{1}H$} NMR (151 MHz, $CDCl_3$): $\delta$ 170.8, 165.0, 160.0, 130.1, 128.8, 119.3, 116.8, 111.6, 60.7, 58.0, 55.5, 35.1, 20.9, 20.8 ppm; IR (KBr): 2939, 2842, 1738, 1589, 1523, 1472, 1367, 1236, 1124, 1037, 860, 796, 690, 605 $cm^{-1}$; HRMS (ESI-TOF) *m/z:* $[M + H]^+$ calcd for $C_{14}H_{19}N_4O_3$, 291.1452; found, 291.1450.

**3-(5-(4-Chlorophenyl)-2*H*-tetrazol-2-yl)butyl acetate (1h)**

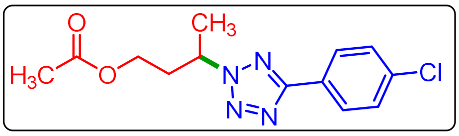


The title compound was obtained according to the general procedure (Method A). Gummy (110 mg, 75% yield); $R_f$ = 0.41 (2:8 EtOAc:hexane, silica gel); $^{1}H$ NMR (600 MHz, $CDCl_3$): $\delta$ 8.09 (d, $J$ = 8.5 Hz, 2H), 7.46 (d, $J$ = 8.5 Hz, 2H), 5.17 (dq, $J$ = 13.7, 6.8 Hz, 1H), 4.12 (dt, $J$ = 11.6, 5.7 Hz, 1H), 3.97 (ddd, $J$ = 11.9, 7.7, 4.8 Hz, 1H), 2.52–2.44 (m, 1H), 2.29–2.23 (m, 1H), 1.98 (s, 3H), 1.71 (d, $J$ = 6.8 Hz, 3H) ppm; $^{13}C$ {$^{1}H$} NMR (151 MHz, $CDCl_3$): $\delta$ 170.9, 164.3, 136.5, 129.3, 128.3, 126.1, 60.7, 58.1, 35.1, 20.9, 20.8 ppm; IR (KBr): 3079, 2984, 2957, 2929, 2854, 1742, 1608, 1456, 1420, 1367, 1231, 1137, 1091, 1037, 1016, 840, 759, 636, 605 $cm^{-1}$; HRMS (ESI-TOF) *m/z:* $[M + H]^+$ calcd for $C_{13}H_{16}{}^{35}ClN_4O_2$, 295.0956; found, 295.0945.

**3-(5-(4-Bromophenyl)-2*H*-tetrazol-2-yl)butyl acetate (1i)**

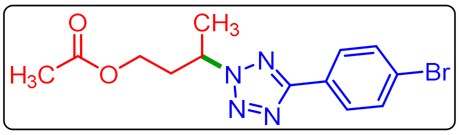

The title compound was obtained according to the general procedure (Method A). Colorless oil (124 mg, 73% yield); $R_f$ = 0.46 (2:8 EtOAc:hexane, silica gel); $^1$H NMR (400 MHz, $CDCl_3$): $\delta$ 7.97 (d, $J$ = 8.5 Hz, 2H), 7.57 (d, $J$ = 8.5 Hz, 2H), 5.17–5.06 (m, 1H), 4.07 (ddd, $J$ = 11.6, 6.4, 5.2 Hz, 1H), 3.91 (ddd, $J$ = 11.9, 7.7, 4.8 Hz, 1H), 2.48–2.37 (m, 1H), 2.21 (ddt, $J$ = 14.7, 7.7, 5.1 Hz, 1H), 1.92 (s, 3H), 1.66 (d, $J$ = 6.8 Hz, 3H) ppm; $^{13}$C {$^1$H} NMR (151 MHz, $CDCl_3$): $\delta$ 170.8, 164.2, 132.2, 128.4, 126.5, 124.7, 60.7, 58.1, 35.0, 20.8, 20.7 ppm; IR (KBr): 3078, 2953, 2931, 1733, 1605, 1468, 1456, 1366, 1251, 1233, 1069, 1045, 1003, 844, 759, 605, 523 cm$^{-1}$; HRMS (ESI-TOF) *m/z:* $[M + H]^+$ calcd for $C_{13}H_{16}{}^{79}BrN_4O_2$, 339.0451; found, 339.0445.

**3-(5-(4-(Trifluoromethyl)phenyl)-2*H*-tetrazol-2-yl)butyl acetate (1j)**

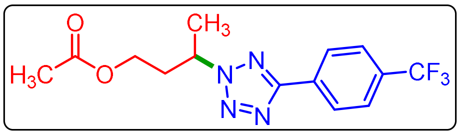

The title compound was obtained according to the general procedure (Method A). Colourless oil (128 mg, 78% yield); $R_f$ = 0.44 (2:8 EtOAc:hexane, silica gel); $^1$H NMR (600 MHz, $CDCl_3$): $\delta$ 8.24 (d, $J$ = 8.1 Hz, 2H), 7.71 (d, $J$ = 8.1 Hz, 2H), 5.16 (h, $J$ = 6.8 Hz, 1H), 4.10 (dt, $J$ = 11.8, 5.9 Hz, 1H), 3.97–3.92 (m, 1H), 2.49–2.43 (m, 1H), 2.27–2.21 (m, 1H), 1.95 (s, 3H), 1.70 (d, $J$ = 6.6 Hz, 3H) ppm; $^{13}$C {$^1$H} NMR (151 MHz, $CDCl_3$): $\delta$ 170.9, 163.9, 132.2 (q, $J$ = 32.47 MHz), 131.0, 127.2, 126.0 (d, $J$ = 3.47 MHz), 124.0 (q, $J$ = 272.4 MHz), 60.7, 58.3, 35.1, 20.88, 20.80 ppm; $^{19}$F NMR (565 MHz, $CDCl_3$): −62.8 ppm; IR (KBr): 2992, 2964, 2848, 2634, 1744, 1624, 1540, 1468, 1428, 1363, 1325, 1234, 1168, 1127, 1067, 1037, 1019, 955, 853, 766, 729, 635, 601 cm$^{-1}$; HRMS (ESI-TOF) *m/z:* $[M + H]^+$ calcd for $C_{14}H_{16}F_3N_4O_2$, 329.1220; found, 329.1220.

**3-(5-(4-Nitrophenyl)-2*H*-tetrazol-2-yl)butyl acetate (1k)**

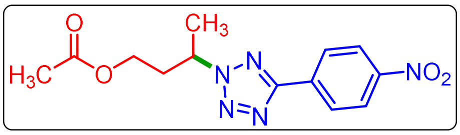

The title compound was obtained according to the general procedure (Method A). White solid (122 mg, 80% yield); $R_f$ = 0.36 (2:8 EtOAc:hexane, silica gel); $^1$H NMR (600 MHz, $CDCl_3$): $\delta$ 8.33 (s, 4H), 5.20 (dq, $J$ = 13.6, 6.7 Hz, 1H), 4.13 (dt, $J$ = 11.6, 5.8 Hz, 1H), 4.00–3.94 (m, 1H), 2.52–2.46 (m, 1H), 2.31–2.25 (m, 1H), 1.98 (s, 3H), 1.73 (d, $J$ = 6.8 Hz, 3H) ppm; $^{13}$C {$^1$H} NMR (151 MHz, $CDCl_3$): $\delta$ 170.7, 163.3, 149.0, 133.5, 127.8, 124.3, 60.6, 58.5, 35.1, 20.84, 20.8 ppm; IR (KBr): 3097, 2995, 2939, 1951, 1734, 1601, 1520,

1457, 1370, 1354, 1248, 1105, 1058, 1037, 866, 736, 691 $cm^{-1}$; HRMS (ESI-TOF) *m/z:* $[M + H]^+$ calcd for $C_{13}H_{16}N_5O_4$, 306.1197; found, 306.1199.

**3-(5-(2-Chlorophenyl)-2*H*-tetrazol-2-yl)butyl acetate (1l)**

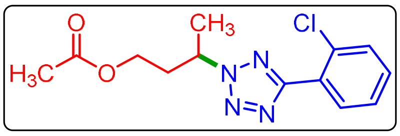

The title compound was obtained according to the general procedure (Method A). Colourless oil (106 mg, 72% yield); $R_f$ = 0.36 (2:8 EtOAc:hexane, silica gel); $^1H$ NMR (600 MHz, $CDCl_3$): $\delta$ 7.90 (d, $J$ = 7.3 Hz, 1H), 7.50 (d, $J$ = 7.6 Hz, 1H), 7.39–7.34 (m, 2H), 5.18 (dq, $J$ = 13.4, 6.7 Hz, 1H), 4.11 (dt, $J$ = 11.6, 5.8 Hz, 1H), 3.97–3.93 (m, 1H), 2.50–2.44 (m, 1H), 2.27–2.21 (m, 1H), 1.96 (s, 3H), 1.70 (d, $J$ = 6.8 Hz, 3H) ppm; $^{13}C$ {$^1H$} NMR (151 MHz, $CDCl_3$): $\delta$ 170.9, 163.3, 133.2, 131.4, 131.2, 130.9, 127.0, 126.7, 60.7, 58.2, 35.1, 20.9, 20.8 ppm; IR (KBr): 2993, 2960, 2931, 2845, 1742, 1601, 1571, 1515, 1461, 1368, 1340, 1231, 1177, 1127, 1032, 1004, 952, 803, 751, 667, 605, 513 $cm^{-1}$; HRMS (ESI-TOF) *m/z:* $[M + H]^+$ calcd for $C_{13}H_{16}{}^{35}ClN_4O_2$, 295.0956; found, 295.0950.

**3-(5-(2-Bromophenyl)-2*H*-tetrazol-2-yl)butyl acetate (1m)**

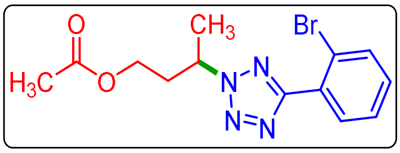

The title compound was obtained according to the general procedure (Method A). Colourless oil (126 mg, 74% yield); $R_f$ = 0.37 (2:8 EtOAc:hexane, silica gel); $^1H$ NMR (600 MHz, $CDCl_3$): $\delta$ 7.80 (d, $J$ = 7.7 Hz, 1H), 7.68 (d, $J$ = 8.0 Hz, 1H), 7.38 (t, $J$ = 7.5 Hz, 1H), 7.28 (t, $J$ = 7.7 Hz, 1H), 5.21–5.14 (m, 1H), 4.12–4.08 (m, 1H), 3.93 (ddd, $J$ = 11.9, 7.8, 4.8 Hz, 1H), 2.49–2.42 (m, 1H), 2.26–2.20 (m, 1H), 1.96 (s, 3H), 1.69 (d, $J$ = 6.8 Hz, 3H) ppm; $^{13}C$ {$^1H$} NMR (151 MHz, $CDCl_3$): $\delta$ 170.9, 164.0, 134.1, 131.7, 131.3, 128.7, 127.6, 122.2, 60.7, 58.1, 35.1, 20.9, 20.8 ppm; IR (KBr): 2983, 2940, 1738, 1604, 1451, 1367, 1229, 1136, 1037, 1008, 837, 756, 642, 606, 512, 451 $cm^{-1}$; HRMS (ESI-TOF) *m/z:* $[M + H]^+$ calcd for $C_{13}H_{16}{}^{79}BrN_4O_2$, 339.0451; found, 339.0457.

**(*E*)-3-(5-Styryl-2*H*-tetrazol-2-yl)butyl acetate (1n)**

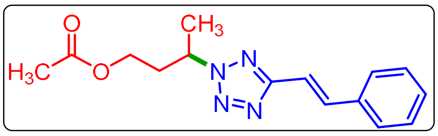

The title compound was obtained according to the general procedure (Method A). Gummy (77 mg, 54% yield); $R_f$ = 0.59 (2:8 EtOAc:hexane, silica gel); $^1H$ NMR (600 MHz, $CDCl_3$): $\delta$ 7.74 (d, $J$ = 16.5 Hz, 1H), 7.56 (d, $J$ = 7.3 Hz, 2H), 7.39 (t, $J$ = 7.5 Hz, 2H), 7.33 (t, $J$ = 7.3 Hz, 1H), 7.16 (d, $J$ = 16.5 Hz, 1H), 5.17–5.09 (m, 1H), 4.12 (ddd, $J$ = 11.6, 6.5, 5.2 Hz, 1H), 3.96 (ddd, $J$ = 11.9, 7.7, 4.8 Hz,

1H), 2.49–2.41 (m, 1H), 2.26–2.20 (m, 1H), 1.99 (s, 3H), 1.69 (d, *J* = 6.8 Hz, 3H) ppm; $^{13}$C {$^{1}$H} NMR (151 MHz, $CDCl_3$): *δ* 170.8, 164.2, 136.4, 135.8, 129.1, 128.9, 127.2, 113.6, 60.7, 57.9, 35.1, 20.9, 20.7 ppm; IR (KBr): 3060, 3026, 2986, 2940, 1742, 1650, 1578, 1501, 1474, 1444, 1368, 1237, 1048, 1022, 972, 850, 767, 733, 692, 606 cm$^{-1}$; HRMS (ESI-TOF) *m/z:* [M + H]$^+$ calcd for $C_{15}H_{19}N_4O_2$, 287.1503; found, 287.1504.

**3-(5-(2-Bromo-4-methylphenyl)-2*H*-tetrazol-2-yl)butyl acetate (1o)**

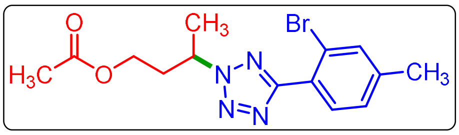


The title compound was obtained according to the general procedure (Method A). Gummy (118 mg, 67% yield); $R_f$ = 0.46 (2:8 EtOAc:hexane, silica gel); $^{1}$H NMR (600 MHz, $CDCl_3$): *δ* 7.71 (d, *J* = 7.7 Hz, 1H), 7.53 (s, 1H), 7.20 (d, *J* = 8.1 Hz, 1H), 5.17 (h, *J* = 7.2 Hz, 1H), 4.12 (dt, *J* = 11.8, 6.1 Hz, 1H), 3.96 (dt, *J* = 12.1, 6.3 Hz, 1H), 2.51–2.44 (m, 1H), 2.36 (s, 3H), 2.27–2.21 (m, 1H), 1.97 (s, 3H), 1.70 (d, *J* = 6.9 Hz, 3H) ppm; $^{13}$C {$^{1}$H} NMR (151 MHz, $CDCl_3$): *δ* 170.9, 164.1, 141.9, 134.7, 131.4, 128.5, 125.9, 121.9, 60.8, 58.1, 35.2, 21.1, 20.9, 20.8 ppm; IR (KBr): 2984, 2953, 2853, 1741, 1611, 1557, 1535, 1458, 1386, 1335, 1232, 1141, 1048, 1030, 949, 850, 827, 766, 672, 606, 560 cm$^{-1}$; HRMS (ESI-TOF) *m/z:* [M + H]$^+$ calcd for $C_{14}H_{18}{}^{79}BrN_4O_2$, 353.0608; found, 353.0612.

**3-(5-(3,4,5-Trimethoxyphenyl)-2*H*-tetrazol-2-yl)butyl acetate (1p)**

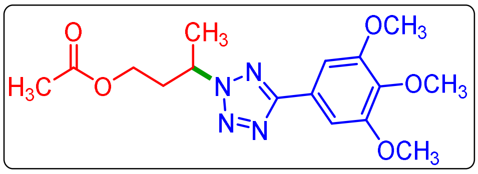


The title compound was obtained according to the general procedure (Method A). Gummy (93 mg, 53% yield); $R_f$ = 0.24 (2:8 EtOAc:hexane, silica gel); $^{1}$H NMR (600 MHz, $CDCl_3$): *δ* 7.35 (s, 2H), 5.11 (dt, *J* = 13.8, 6.8 Hz, 1H), 4.11–4.06 (m, 1H), 3.96 – 3.93 (m, 1H), 3.92 (s, 6H), 3.86 (s, 3H), 2.46 (dt, *J* = 14.1, 6.5 Hz, 1H), 2.23 (dt, *J* = 13.0, 5.2 Hz, 1H), 1.95 (s, 3H), 1.68 (d, *J* = 6.8 Hz, 3H) ppm; $^{13}$C {$^{1}$H} NMR (151 MHz, $CDCl_3$): *δ* 170.9, 165.0, 153.8, 139.9, 122.9, 104.0, 61.1, 60.8, 58.0, 56.4, 35.1, 20.9, 20.8 ppm; IR (KBr): 2964, 2938, 2846, 1738, 1590, 1481, 1424, 1395, 1368, 1234, 1185, 1127, 1047, 1005, 874, 850, 760 cm$^{-1}$; HRMS (ESI-TOF) *m/z:* [M + H]$^+$ calcd for $C_{16}H_{23}N_4O_5$, 351.1663; found, 351.1665.

**3-(5-(Thiophen-2-yl)-2*H*-tetrazol-2-yl)butyl acetate (1q)**

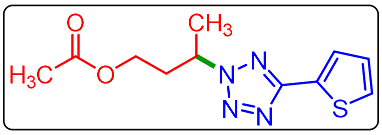

The title compound was obtained according to the general procedure (Method A). Gummy (83 mg, 62% yield); $R_f$ = 0.41 (2:8 EtOAc:hexane, silica gel); $^1$H NMR (600 MHz, $CDCl_3$): $\delta$ 7.84–7.79 (m, 1H), 7.49–7.44 (m, 1H), 7.17 (dd, $J$ = 4.9, 3.7 Hz, 1H), 5.20–5.13 (m, 1H), 4.15 (ddd, $J$ = 11.7, 6.4, 5.2 Hz, 1H), 3.99 (ddd, $J$ = 13.6, 9.2, 5.4 Hz, 1H), 2.53–2.47 (m, 1H), 2.27 (ddt, $J$ = 14.7, 7.7, 5.2 Hz, 1H), 2.01 (s, 3H), 1.73 (d, $J$ = 6.8 Hz, 3H) ppm; $^{13}$C {$^1$H} NMR (151 MHz, $CDCl_3$): $\delta$ 170.9, 161.2, 129.3, 128.1, 127.97, 127.91, 60.7, 58.1, 35.1, 20.9, 20.8 ppm; IR (KBr): 3106, 2958, 2924, 2853, 1737, 1653, 1572, 1478, 1386, 1367, 1229, 1104, 1045, 1027, 969, 852, 796, 708, 605 cm$^{-1}$; HRMS (ESI-TOF) *m/z:* $[M + H]^+$ calcd for $C_{11}H_{15}N_4O_2S$, 267.0910; found, 267.0904.

**3-(4-Phenyl-2*H*-1,2,3-triazol-2-yl)butyl acetate (1r)**

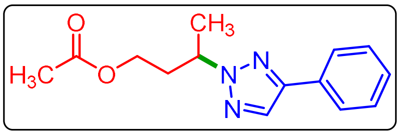

The title compound was obtained according to the general procedure (Method A). Gummy (83 mg, 64% yield); $R_f$ = 0.62 (2:8 EtOAc:hexane, silica gel); $^1$H NMR (600 MHz, $CDCl_3$): $\delta$ 7.82 (s, 1H), 7.77 (d, $J$ = 7.2 Hz, 2H), 7.42 (t, $J$ = 7.6 Hz, 2H), 7.34 (t, $J$ = 7.4 Hz, 1H), 4.92–4.85 (m, 1H), 4.08 (dt, $J$ = 11.5, 5.8 Hz, 1H), 3.94 (ddd, $J$ = 11.5, 7.9, 5.3 Hz, 1H), 2.47–2.40 (m, 1H), 2.20–2.14 (m, 1H), 2.01 (s, 3H), 1.63 (d, $J$ = 6.8 Hz, 3H) ppm; $^{13}$C {$^1$H} NMR (151 MHz, $CDCl_3$): $\delta$ 171.0, 147.6, 130.7, 130.7, 129.0, 128.5, 126.0, 61.3, 58.8, 35.4, 29.8, 21.0 ppm; IR (KBr): 3036, 2962, 2920, 2850, 1747, 1605, 1560, 1474, 1460, 1368, 1236, 1046, 977, 920, 842, 800, 769, 695, 605 cm$^{-1}$; HRMS (ESI-TOF) *m/z:* $[M + H]^+$ calcd for $C_{14}H_{18}N_3O_2$, 260.1394; found, 260.1387.

**3-(4-(4-(*tert*-Butyl)phenyl)-2*H*-1,2,3-triazol-2-yl)butyl acetate (1s)**

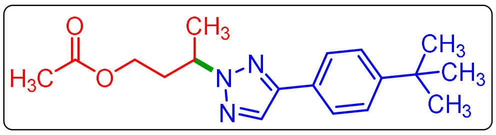

The title compound was obtained according to the general procedure (Method A). Gummy (95 mg, 60% yield); $R_f$ = 0.31 (1:9 EtOAc:hexane, silica gel); $^1$H NMR (600 MHz, $CDCl_3$): $\delta$ 7.63 (s, 1H), 7.53 (d, $J$ = 8.2 Hz, 2H), 7.28 (d, $J$ = 8.2 Hz, 2H), 4.74–4.69 (m, 1H), 3.91 (dd, $J$ = 11.4, 5.7 Hz, 1H), 3.76 (ddd, $J$ = 11.8, 7.8, 5.3 Hz, 1H), 2.27 (ddd, $J$ = 14.7, 10.2, 5.6 Hz, 1H), 2.00 (ddd, $J$ = 14.2, 8.0, 3.8 Hz, 1H), 1.84 (s, 3H), 1.45 (d, $J$ = 6.8 Hz, 3H), 1.17 (s, 9H) ppm; $^{13}$C {$^1$H} NMR (151 MHz, $CDCl_3$): $\delta$ 171.0, 151.6, 147.6, 130.6, 127.8, 125.9, 125.8, 61.2, 58.7, 35.4, 34.8, 31.4, 21.0, 20.9 ppm; IR (KBr): 3032,

2963, 2906, 2870, 1744, 1556, 1484, 1462, 1367, 1318, 1269, 1236, 1113, 1047, 980, 835, 739, 605 cm$^{-1}$; HRMS (ESI-TOF) *m/z:* $[M + H]^+$ calcd for $C_{18}H_{26}N_3O_2$, 316.2020; found, 316.2025.

**3-(4-(4-Chlorophenyl)-2*H*-1,2,3-triazol-2-yl)butyl acetate (1t)**

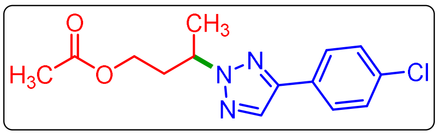


The title compound was obtained according to the general procedure (Method A). Gummy (97 mg, 66% yield); $R_f$ = 0.52 (2:8 EtOAc:hexane, silica gel); $^1H$ NMR (600 MHz, $CDCl_3$): $\delta$ 7.80 (s, 1H), 7.70 (d, $J$ = 8.5 Hz, 2H), 7.38 (d, $J$ = 8.5 Hz, 2H), 4.91–4.84 (m, 1H), 4.07 (dt, $J$ = 11.5, 5.8 Hz, 1H), 3.92 (ddd, $J$ = 11.5, 7.9, 5.2 Hz, 1H), 2.42 (ddt, $J$ = 11.1, 9.4, 5.6 Hz, 1H), 2.16 (ddt, $J$ = 10.6, 7.9, 5.4 Hz, 1H), 2.00 (s, 3H), 1.62 (d, $J$ = 6.8 Hz, 3H) ppm; $^{13}C$ {$^1H$} NMR (151 MHz, $CDCl_3$) $\delta$ 171.1, 146.5, 134.2, 130.7, 129.2, 129.1, 127.2, 61.2, 58.8, 35.4, 21.0 ppm; IR (KBr): 3127, 2984, 2940, 1740, 1603, 1529, 1475, 1429, 1369, 1317, 1237, 1092, 1047, 1015, 994, 978, 830, 722, 653, 602 cm$^{-1}$; HRMS (ESI-TOF) *m/z:* $[M + H]^+$ calcd for $C_{14}H_{17}{}^{35}ClN_3O_2$, 294.1004; found, 294.1007.

**3-(1,1-Dioxido-3-oxobenzo[d]isothiazol-2(3H)-yl)butyl acetate (1u)**

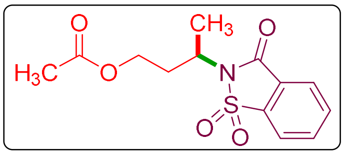


The title compound was obtained according to the general procedure (Method A). Gummy (104 mg, 70% yield); Rf = 0.36 (3:7 EtOAc:hexane, silica gel); 1H NMR (500 MHz, $CDCl_3$): δ 8.00–7.99 (m, 1H), 7.85–7.83 (m, 1H), 7.82–7.76 (m, 2H), 4.47–4.39 (m, 1H), 4.15–4.04 (m, 2H), 2.57–2.50 (m, 1H), 2.15–2.05 (m, 1H), 1.96 (s, 3H), 1.59 (d, J = 7.0 Hz, 3H) ppm; 13C {1H} NMR (101 MHz, CDCl3): δ 171.1, 159.0, 137.7, 134.8, 134.4, 127.4, 125.1, 120.9, 61.3, 47.8, 32.6, 20.9, 18.9 ppm; IR (KBr): 3091, 2978, 2944, 2870, 1744, 1460, 1357, 1249, 1172, 1051, 979, 890, 786, 677, 586 cm-1; HRMS (ESI-TOF) m/z: [M + H]+ calcd for C13H16NO5S, 298.0744; found, 298.0753.

**Indolo[*2,1-b*]quinazoline-6,12-dione (X)**[7]

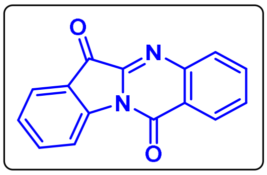


The title compound was obtained according to the general procedure (Method A). Yellow solid (76 mg, 61% yield); $R_f$ = 0.49 (3:7 EtOAc:hexane, silica gel); $^1H$ NMR (600 MHz, $CDCl_3$): $\delta$ 8.62 (d, $J$ = 8.1 Hz, 1H), 8.44 (d, $J$ = 7.5 Hz, 1H), 8.03 (d, $J$ = 8.5 Hz, 1H), 7.91 (d, $J$ = 8.2 Hz, 1H), 7.85 (t, $J$ = 8.0 Hz, 1H), 7.79 (t, $J$ = 8.0 Hz, 1H), 7.68 (t, $J$ = 7.3 Hz, 1H), 7.43 (t, $J$ = 7.5 Hz, 1H) ppm; $^{13}C$ {$^1H$} NMR (151 MHz, $CDCl_3$) $\delta$ 182.7, 158.2, 146.7, 146.4, 144.4, 138.4, 135.3, 130.8, 130.4, 127.6, 127.3, 125.5, 123.8, 122.0, 118.1 ppm; IR

(KBr): 1731, 1690, 1455, 1351, 1321, 1111, 1039, 932, 751, 677 cm$^{-1}$; HRMS (ESI-TOF) *m/z:* [M + H]$^+$ calcd for $C_{15}H_9N_2O_2$, 249.0659; found, 249.0667.

**3-(5-Phenyl-2*H*-tetrazol-2-yl)butyl benzoate (2a)**

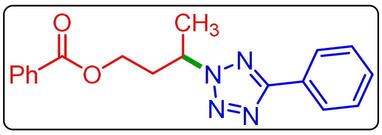


The title compound was obtained according to the general procedure (Method A). Gummy (98 mg, 61% yield); $R_f$ = 0.55 (2:8 EtOAc:hexane, silica gel); $^1$H NMR (400 MHz, $CDCl_3$): $\delta$ 8.15–8.05 (m, 2H), 7.95–7.87 (m, 2H), 7.51–7.42 (m, 4H), 7.34 (t, $J$ = 7.7 Hz, 2H), 5.30–5.18 (m, 1H), 4.40 (ddd, $J$ = 11.6, 6.6, 5.0 Hz, 1H), 4.21 (ddd, $J$ = 11.9, 7.7, 4.7 Hz, 1H), 2.66–2.57 (m, 1H), 2.39 (ddt, $J$ = 14.8, 7.6, 5.0 Hz, 1H), 1.73 (d, $J$ = 6.8 Hz, 3H) ppm; $^{13}$C {$^1$H} NMR (151 MHz, $CDCl_3$): $\delta$ 166.4, 165.2, 133.2, 130.4, 129.8, 129.7, 128.9, 128.5, 127.6, 127.0, 61.4, 58.2, 35.3, 20.9 ppm; IR (KBr): 3061, 2978, 2959, 2920, 2850, 1720, 1601, 1579, 1526, 1466, 1450, 1379, 1313, 1346, 1273, 1174, 1112, 1070, 1026, 785, 733, 712, 694 cm$^{-1}$; HRMS (ESI-TOF): *m/z:* [M + H]$^+$ calcd for $C_{18}H_{19}N_4O_2$, 323.1503; found, 323.1534.

**3-Phenyl-3-(5-phenyl-2*H*-tetrazol-2-yl)propyl acetate (3a)**

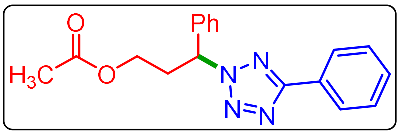


The title compound was obtained according to the general procedure (Method A). Gummy (148 mg, 92% yield); $R_f$ = 0.44 (2:8 EtOAc:hexane, silica gel); $^1$H NMR (600 MHz, $CDCl_3$): $\delta$ 8.12 (d, $J$ = 7.4 Hz, 2H), 7.46–7.41 (m, 5H), 7.36–7.30 (m, 3H), 6.08 (t, $J$ = 7.8 Hz, 1H), 4.04 (t, $J$ = 6.9 Hz, 2H), 2.95 (td, $J$ = 14.8, 5.9 Hz, 1H), 2.67 (td, $J$ = 12.9, 6.3 Hz, 1H), 2.00 (s, 3H) ppm; $^{13}$C {$^1$H} NMR (151 MHz, $CDCl_3$): $\delta$ 170.9, 165.2, 136.9, 130.4, 130.2, 129.2, 128.9, 128.5, 127.3, 126.9, 65.3, 60.7, 34.3, 20.9 ppm; IR (KBr): 3067, 3034, 2961, 2662, 1743, 1592, 1529, 1495, 1451, 1367, 1235, 1041, 789, 734, 604 cm$^{-1}$; HRMS (ESI-TOF) *m/z:* [M + H]$^+$ calcd for $C_{18}H_{19}N_4O_2$, 323.1503; found, 323.1509.

**4-Phenyl-4-(5-phenyl-2*H*-tetrazol-2-yl)butyl acetate (4a)**

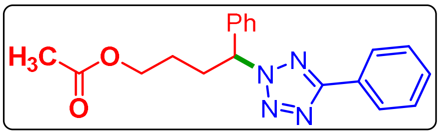


The title compound was obtained according to the general procedure (Method A). Gummy (158 mg, 94% yield); $R_f$ = 0.47 (2:8 EtOAc:hexane, silica gel); $^1$H NMR (600 MHz, $CDCl_3$): $\delta$ 8.18–8.12 (m, 2H), 7.50–7.44 (m, 5H), 7.38 (t, $J$ = 7.3 Hz, 2H), 7.34 (t, $J$ = 7.2 Hz, 1H), 5.95 (dd, $J$ = 9.0, 6.6 Hz, 1H), 4.18–4.04 (m, 2H), 2.74–2.68 (m, 1H), 2.46–2.40 (m, 1H), 2.05 (s, 3H), 1.67 (ddd, $J$ = 16.5, 10.0, 6.6 Hz, 1H), 1.60 (ddd, $J$ = 19.7, 10.0, 6.2 Hz, 1H) ppm; $^{13}$C {$^1$H} NMR (151 MHz,

$CDCl_3$): δ 171.1, 165.2, 137.5, 130.4, 129.1, 128.99, 128.92, 127.5, 127.3, 126.9, 68.1, 63.5, 32.1, 25.6, 21.0 ppm; IR (KBr): 3060, 3033, 2956, 2867, 1735, 1540, 1483, 1449, 1378, 1033, 755, 728, 619 $cm^{-1}$; HRMS (APCI-TOF) *m/z:* $[M + H]^+$ calcd for $C_{19}H_{21}N_4O_2$, 337.1659; found, 337.1659.

**3-Methyl-3-(5-phenyl-2*H*-tetrazol-2-yl)butyl acetate (5a)**

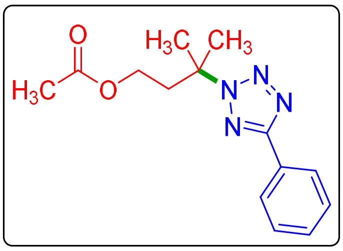

The title compound was obtained according to the general procedure (Method A). Gummy (118 mg, 86% yield); $R_f$ = 0.43 (2:8 EtOAc:hexane, silica gel); $^1H$ NMR (600 MHz, $CDCl_3$): δ 8.12 (dd, *J* = 8.0, 1.6 Hz, 2H), 7.50–7.39 (m, 3H), 4.07 (t, *J* = 6.5 Hz, 2H), 2.40 (t, *J* = 6.5 Hz, 2H), 1.83 (s, 3H), 1.82 (s, 6H) ppm; $^{13}C$ {$^1H$} NMR (151 MHz, $CDCl_3$): δ 170.9, 164.7, 130.3, 129.0, 127.8, 126.9, 65.0, 60.2, 40.5, 27.6, 20.9; IR (KBr): 3057, 3030, 2965, 2668, 1742, 1582, 1499, 1438, 1333, 1142, 770, 623 $cm^{-1}$; HRMS (ESI-TOF) *m/z:* $[M + H]^+$ calcd for $C_{14}H_{19}N_4O_2$, 275.1503; found, 275.1514.

**4-(5-Phenyl-2*H*-tetrazol-2-yl)pentyl acetate (6a) + 3-(5-Phenyl-2*H*-tetrazol-2-yl)pentyl acetate (6'a)**

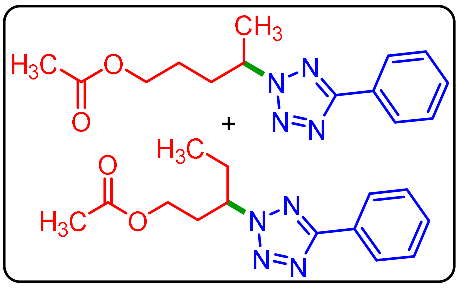

The title compound was obtained according to the general procedure (Method A). Gummy (100 mg, 73% yield); $R_f$ = 0.55 (2:8 EtOAc:hexane, silica gel); $^1H$ NMR (600 MHz, $CDCl_3$): δ 8.15 (d, *J* = 6.9 Hz, 2.38H), 7.51–7.43 (m, 3.61H), 5.02 (dq, *J* = 13.4, 6.7 Hz, 1H), 4.96–4.91 (m, 0.21H), 4.10–4.01 (m, 2.42H), 3.91–3.86 (m, 0.21H), 2.50–2.43 (m, 0.22H), 2.30–2.24 (m, 0.21H), 2.22–2.14 (m, 1H), 2.03 (s, 3H), 2.02–1.96 (m, 1.25H), 1.69 (d, *J* = 6.8 Hz, 3H), 1.63 (ddt, *J* = 16.8, 12.3, 6.3 Hz, 1.23H), 1.49 (tq, *J* = 11.5, 6.1 Hz, 1.21H) ppm; $^{13}C$ {$^1H$} NMR (151 MHz, $CDCl_3$): δ 171.1, 165.0, 130.3, 129.0, 127.7, 126.9, (63.99), 63.6, (60.85), 60.5, 33.0, (28.25), 25.2, 21.0, 20.8 ppm; IR (KBr): 3066, 3010, 2953, 2931, 2841, 1744, 1549, 1473, 1392, 1187, 1068, 1033, 1001, 920, 789, 713, 693 $cm^{-1}$; HRMS (ESI-TOF) *m/z:* $[M + H]^+$ calcd for $C_{14}H_{19}N_4O_2$, 275.1503; found, 275.1493.

**5-(5-Phenyl-2*H*-tetrazol-2-yl)hexyl acetate (7a) + 4-(5-Phenyl-2*H*-tetrazol-2-yl)hexyl acetate (7'a)**

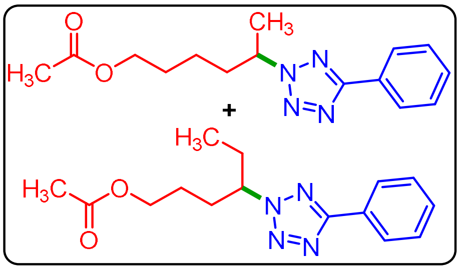

The title compound was obtained according to the general procedure (Method A). Gummy (117 mg, 81% yield); $R_f$ = 0.61 (2:8 EtOAc:hexane, silica gel); $^1$H NMR (600 MHz, $CDCl_3$): $\delta$ 8.16–8.11 (m, 2.45H), 7.49–7.43 (m, 3.85H), 4.99–4.93 (m, 1H), 4.79–4.74 (m, 0.4H), 4.06–3.95 (m, 2.8H), 2.19–2.09 (m, 1.5H), 2.00 (s, 1.2H), 1.97 (s, 3H), 1.96–1.84 (m, 1.4H), 1.65 (d, $J$ = 6.7 Hz, 3H), 1.64–1.58 (m, 2H), 1.44–1.37 (m, 0.4H), 1.36–1.30 (m, 1H), 1.25–1.16 (m, 1.6H), 0.82 (t, $J$ = 7.2 Hz, 1.2H); $^{13}$C {$^1$H} NMR (151 MHz, $CDCl_3$) $\delta$ 171.3, 165.0, 130.4, 129.0, 127.8, 126.9, 66.69, 64.1, 63.64, 60.8, 36.0, 28.1, 22.5, 21.1, 20.8; IR (KBr): 3071, 305, 2943, 2911, 2838, 1749, 1550, 1433, 1382, 1335, 1251, 1180, 1062, 1011, 919, 767, 739, 691 cm$^{-1}$; HRMS (APCI-TOF) *m/z:* [M + H]$^+$ calcd for $C_{15}H_{21}N_4O_2$, 289.1659; found, 289.1651.

**1-(5-Phenyl-2*H*-tetrazol-2-yl)ethyl acetate (9a)**

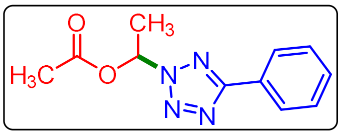

The title compound was obtained according to the general procedure (Method A). Gummy (40 mg, 34% yield); $R_f$ = 0.37 (1:9 EtOAc:hexane, silica gel); $^1$H NMR (600 MHz, $CDCl_3$): $\delta$ 8.18 (dd, $J$ = 7.2, 2.3 Hz, 2H), 7.51–7.47 (m, 3H), 7.37 (q, $J$ = 6.2 Hz, 1H), 2.14 (s, 3H), 2.02 (d, $J$ = 6.3 Hz, 3H) ppm; $^{13}$C {$^1$H} NMR (151 MHz, $CDCl_3$): $\delta$ 168.8, 165.4, 130.7, 129.0, 127.2, 126.9, 80.1, 20.8, 19.5 ppm; IR (KBr): 3071, 2964, 2859, 1764, 1609, 1530, 1467, 1450, 1372, 1341, 1216, 1184, 1086, 1042, 1023, 941, 852, 789, 733, 693 cm$^{-1}$; HRMS (ESI-TOF): *m/z* calcd. for $C_{11}H_{13}N_4O_2$ [M + H]$^+$ 233.1033; found 233.1038.

**1-(5-(4-Chlorophenyl)-2*H*-tetrazol-2-yl)ethyl acetate (9h)**

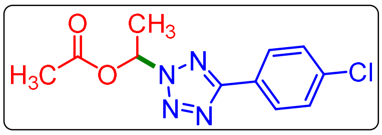

The title compound was obtained according to the general procedure (Method A). Gummy (44 mg, 33% yield); $R_f$ = 0.33 (1:9 EtOAc:hexane, silica gel); $^1$H NMR (600 MHz, $CDCl_3$): $\delta$ 8.12 (d, $J$ = 8.6 Hz, 2H), 7.47 (d, $J$ = 8.6 Hz, 2H), 7.36 (q, $J$ = 6.3 Hz, 1H), 2.14 (s, 3H), 2.02 (d, $J$ = 6.3 Hz, 3H) ppm; $^{13}$C {$^1$H} NMR (151 MHz, $CDCl_3$): $\delta$ 168.8, 164.6, 136.8, 129.4, 128.5, 125.7, 80.1, 20.8, 19.5 ppm; IR (KBr): 2999, 2923, 2852, 1763, 1606, 1458, 1418, 1370, 1213, 1183, 1088, 1033, 1007, 940, 839, 758, 729, 600 cm$^{-1}$; HRMS (ESI-TOF) *m/z:* [M + H]$^+$ calcd for $C_{11}H_{12}{}^{35}ClN_4O_2$, 267.0643; found, 267.0651.

**1-(5-(4-Bromophenyl)-2*H*-tetrazol-2-yl)ethyl acetate (9i)**

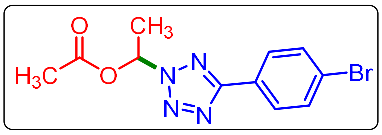


The title compound was obtained according to the general procedure (Method A). Gummy (56 mg, 36% yield); $R_f$ = 0.31 (1:9 EtOAc:hexane, silica gel); $^1$H NMR (600 MHz, $CDCl_3$): $\delta$ 8.05 (d, $J$ = 8.5 Hz, 2H), 7.63 (d, $J$ = 8.5 Hz, 2H), 7.36 (q, $J$ = 6.3 Hz, 1H), 2.14 (s, 3H), 2.01 (d, $J$ = 6.3 Hz, 3H) ppm; $^{13}$C {$^1$H} NMR (151 MHz, $CDCl_3$): $\delta$ 168.8, 164.7, 132.3, 128.7, 126.1, 125.2, 80.1, 20.8, 19.5 ppm; IR (KBr): 3008, 2930, 2848, 1756, 1610, 1451, 1376, 1173, 1072, 1001, 925, 763, 735, 611 cm$^{-1}$; HRMS (ESI-TOF) *m/z:* $[M + H]^+$ calcd for $C_{11}H_{12}{}^{79}BrN_4O_2$, 311.0138; found, 311.0144.

**1-(5-(*p*-Tolyl)-2*H*-tetrazol-2-yl)ethyl acetate (9d) + 2-Methyl-5-(*p*-tolyl)-2*H*-tetrazole (dm)**

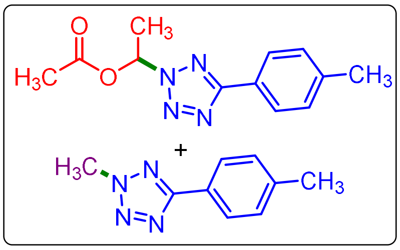


The title compound was obtained according to the general procedure (Method A). Gummy (110 mg, 56% yield); $R_f$ = 0.35 (1:9 EtOAc:hexane, silica gel); $^1$H NMR (600 MHz, $CDCl_3$): $\delta$ 8.03 (d, $J$ = 8.2 Hz, 2H), 7.99 (d, $J$ = 8.2 Hz, 0.9 H), 7.33 (q, $J$ = 6.3 Hz, 1H), 7.27 (d, $J$ = 8.2 Hz, 2.58H), 4.36 (s, 0.88H), 2.39 (s, 3.93H), 2.11 (s, 3H), 1.98 (d, $J$ = 6.3 Hz, 3H) ppm; $^{13}$C {$^1$H} NMR (151 MHz, $CDCl_3$): $\delta$ 168.88, 165.53, 165.50, 140.96, 140.63, 129.74, 127.09, 126.83, 124.64, 124.30, 80.02, 39.59, 21.67, 21.63, 20.87, 19.51 ppm; IR (KBr): 3005, 2961, 2849, 1754, 1613, 1515, 1444, 1362, 1278, 1190, 1033, 959, 842, 737, 691 cm$^{-1}$; HRMS (ESI-TOF) *m/z:* $[M + H]^+$ calcd for $C_{12}H_{15}N_4O_2$, 247.1190; found, 247.1191.

**1-(5-Phenyl-2*H*-tetrazol-2-yl)ethyl benzoate (10a)**

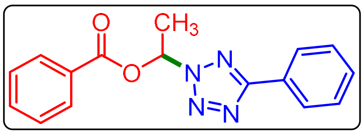


The title compound was obtained according to the general procedure (Method A). Gummy (46 mg, 31% yield); $R_f$ = 0.52 (1:9 EtOAc:hexane, silica gel); $^1$H NMR (600 MHz, $CDCl_3$): $\delta$ 8.16 (dd, $J$ = 7.4, 2.3 Hz, 2H), 8.03 (d, $J$ = 7.2 Hz, 2H), 7.60 (q, $J$ = 6.2 Hz, 1H), 7.56 (t, $J$ = 7.5 Hz, 1H), 7.47–7.44 (m, 3H), 7.42 (t, $J$ = 7.8 Hz, 2H), 2.13 (d, $J$ = 6.3 Hz, 3H) ppm; $^{13}$C {$^1$H} NMR (151 MHz, $CDCl_3$): $\delta$ 165.5, 164.5, 134.1, 130.7, 130.3, 129.0, 128.7, 128.6, 127.2, 127.2, 80.6, 19.7 ppm; IR (KBr): 3071, 2926, 2856, 1734, 1601, 1530, 1451, 1341, 1259, 1181, 1088, 1018, 889, 747, 704, 512 cm$^{-1}$; HRMS (ESI-TOF) *m/z:* $[M + H]^+$ calcd for $C_{16}H_{15}N_4O_2$, 295.1190; found, 295.1197.

**1-(5-Phenyl-2*H*-tetrazol-2-yl)propyl acetate (11a)**

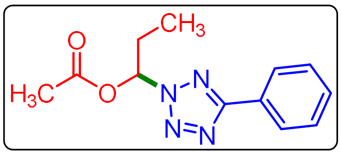


The title compound was obtained according to the general procedure (Method A). Gummy (43 mg, 35% yield); $R_f = 0.39$ (1:9 EtOAc:hexane, silica gel); $^1$H NMR (600 MHz, $CDCl_3$): $\delta$ 8.15 (dd, $J = 7.5$, 2.2 Hz, 2H), 7.48–7.44 (m, 3H), 7.14 (dd, $J = 7.5$, 6.4 Hz, 1H), 2.44–2.36 (m, 1H), 2.36–2.28 (m, 1H), 2.11 (s, 3H), 0.94 (t, $J = 7.5$ Hz, 3H) ppm; $^{13}$C {$^1$H} NMR (151 MHz, $CDCl_3$): $\delta$ 169.0, 165.4, 130.7, 129.0, 127.2, 127.1, 84.1, 26.8, 20.8, 8.7 ppm; IR (KBr): 3059, 2978, 2937, 1762, 1529, 1453, 1364, 1210, 1046, 1016, 921, 827, 734, 693, 529 cm$^{-1}$; HRMS (ESI-TOF) *m/z:* $[M + H]^+$ calcd for $C_{12}H_{15}N_4O_2$, 247.1190; found, 247.1191.

**3-(5-Phenyl-2*H*-tetrazol-2-yl)propyl acetate (11'a)[8]**

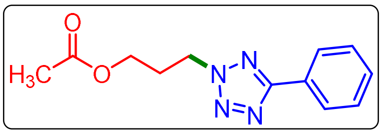


The title compound was obtained according to the general procedure (Method A). Gummy (30 mg, 24% yield); $R_f = 0.35$ (1:9 EtOAc:hexane, silica gel); $^1$H NMR (600 MHz, $CDCl_3$): $\delta$ 8.11 (d, $J = 8.0$ Hz, 2H), 7.49–7.44 (m, 3H), 4.75 (t, $J = 6.9$ Hz, 2H), 4.16 (t, $J = 6.0$ Hz, 2H), 2.39 (p, $J = 6.6$ Hz, 2H), 2.02 (s, 3H) ppm; $^{13}$C {$^1$H} NMR (151 MHz, $CDCl_3$): $\delta$ 170.9, 165.4, 130.5, 129.1, 127.5, 127.0, 61.1, 50.3, 28.6, 20.9 ppm; IR (KBr): 3073, 2976, 2930, 1740, 1603, 1452, 1371, 1218, 1038, 918, 753, 602, 460 cm$^{-1}$; HRMS (ESI-TOF) *m/z:* $[M + H]^+$ calcd for $C_{12}H_{15}N_4O_2$, 247.1190; found, 247.1190.

***N*-Butyl-4-nitro-*N*-(3-(5-phenyl-2*H*-tetrazol-2-yl)butyl)benzenesulfonamide (14a)**

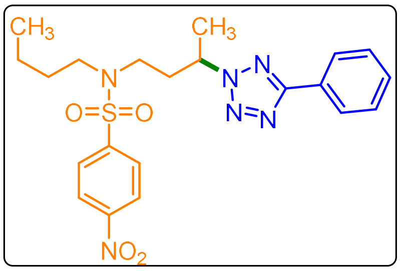


The title compound was obtained according to the general procedure, (Method A), in acetonitrile solvent. Gummy (110 mg, 48% yield); $R_f = 0.46$ (2:8 EtOAc:hexane, silica gel); $^1$H NMR (600 MHz, $CDCl_3$): $\delta$ 8.31 (d, $J = 8.8$ Hz, 2H), 8.14 (dd, $J = 7.6$, 1.9 Hz, 2H), 7.92 (d, $J = 8.8$ Hz, 2H), 7.52–7.46 (m, 3H), 5.09–5.03 (m, 1H), 3.25–3.20 (m, 1H), 3.19–3.14 (m, 1H), 3.10–3.05 (m, 1H), 2.91–2.86 (m, 1H), 2.53–2.46 (m, 1H), 2.33–2.27 (m, 1H), 1.72 (d, $J = 6.8$ Hz, 3H), 1.42 (dt, $J = 15.6$, 7.5 Hz, 2H), 1.27–1.23 (m, 2H), 0.85 (t, $J = 7.4$ Hz, 3H) ppm; $^{13}$C {$^1$H} NMR (151 MHz, $CDCl_3$): $\delta$ 165.3, 150.1, 145.2, 130.6, 129.1, 128.4, 127.4, 126.9, 124.6, 58.5, 49.1, 45.2, 35.8, 30.6, 21.1, 19.9, 13.7 ppm; IR (KBr): 2923, 2855, 1531, 1457, 1348, 1162, 1087, 856, 752, 602 cm$^{-1}$; HRMS (ESI-TOF) *m/z:* $[M + H]^+$ calcd for $C_{21}H_{27}N_6O_4S$, 459.1809; found, 459.1803.

**2-(3-(5-Phenyl-2*H*-tetrazol-2-yl)butyl)isoindoline-1,3-dione (15a)**

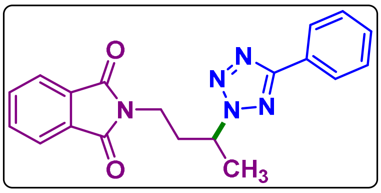


The title compound was obtained according to the general procedure, (Method A), in acetonitrile solvent. Gummy (104 mg, 60% yield); $R_f$ = 0.41 (2:8 EtOAc:hexane, silica gel); $^1$H NMR (600 MHz, $CDCl_3$): $\delta$ 8.09–8.03 (m, 2H), 7.75 (dd, $J$ = 5.4, 3.1 Hz, 2H), 7.63 (dd, $J$ = 5.5, 3.0 Hz, 2H), 7.45–7.39 (m, 3H), 5.08–5.00 (m, 1H), 3.79–3.69 (m, 2H), 2.65–2.56 (m, 1H), 2.36–2.26 (m, 1H), 1.71 (d, $J$ = 6.8 Hz, 3H) ppm; $^{13}$C {$^1$H} NMR (151 MHz, $CDCl_3$): $\delta$ 168.2, 165.1, 134.2, 132.0, 130.3, 128.9, 127.6, 126.9, 123.5, 58.8, 34.9, 34.5, 21.0 ppm; IR (KBr): 3051, 2930, 2849, 1763, 1717, 1610, 1435, 1374, 1177, 979, 835, 784, 720, 618 cm$^{-1}$; HRMS (APCI-TOF) *m/z:* [M + H]$^+$ calcd for $C_{19}H_{18}N_5O_2$, 348.1455; found, 348.1451.

**2-(3-(5-(4-Methoxyphenyl)-2*H*-tetrazol-2-yl)butyl)isoindoline-1,3-dione (15e)**

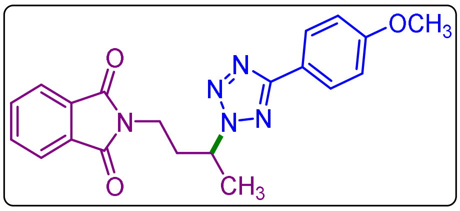


The title compound was obtained according to the general procedure, (Method A), in acetonitrile solvent. Colorless solid (100 mg, 53% yield); $R_f$ = 0.35 (2:8 EtOAc:hexane, silica gel); $^1$H NMR (600 MHz, $CDCl_3$): $\delta$ 7.99 (d, $J$ = 8.8 Hz, 2H), 7.76 (dd, $J$ = 5.4, 3.1 Hz, 2H), 7.64 (dd, $J$ = 5.5, 3.0 Hz, 2H), 6.94 (d, $J$ = 8.8 Hz, 2H), 5.01 (dq, $J$ = 13.4, 6.7 Hz, 1H), 3.83 (s, 3H), 3.73 (dq, $J$ = 21.4, 7.0 Hz, 2H), 2.59 (dq, $J$ = 15.2, 7.4 Hz, 1H), 2.30 (dq, $J$ = 13.1, 6.1 Hz, 1H), 1.69 (d, $J$ = 6.8 Hz, 3H) ppm; $^{13}$C {$^1$H} NMR (151 MHz, $CDCl_3$): $\delta$ 168.1, 164.8, 161.2, 134.0, 131.9, 128.4, 123.3, 120.1, 114.2, 58.5, 55.4, 34.8, 34.4, 20.8 ppm; IR (KBr): 3062, 2934, 2845, 1771, 1706, 1614, 1462, 1396, 1251, 1177, 1027, 961, 841, 764, 718, 613, 530 cm$^{-1}$; HRMS (ESI-TOF) *m/z:* [M + H]$^+$ calcd for $C_{20}H_{20}N_5O_3$, 378.1561; found, 378.1560.

**2-(3-(5-(4-Nitrophenyl)-2*H*-tetrazol-2-yl)butyl)isoindoline-1,3-dione (15k)**

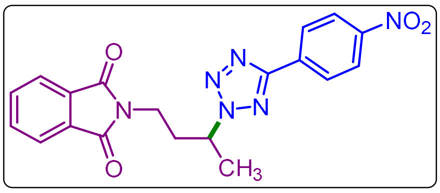


The title compound was obtained according to the general procedure, (Method A), in acetonitrile solvent. White solid (141 mg, 72% yield); $R_f$ = 0.45 (2:8 EtOAc:hexane, silica gel); $^1$H NMR (600 MHz, $CDCl_3$): $\delta$ 8.30–8.25 (m, 4H), 7.76 (dd, $J$ = 5.4, 3.0 Hz, 2H), 7.64 (dd, $J$ = 5.5, 3.0 Hz, 2H), 5.11–5.05 (m, 1H), 3.78–3.68 (m, 2H), 2.63–2.59 (m, 1H), 2.39–2.32 (m, 1H), 1.72 (d, $J$ = 6.8 Hz, 3H) ppm; $^{13}$C {$^1$H} NMR (151 MHz, $CDCl_3$): $\delta$ 168.2,

163.2, 148.9, 134.3, 133.5, 131.9, 127.8, 124.3, 123.5, 59.2, 34.8, 34.5, 21.0 ppm; IR (KBr): 3071, 2931, 2850, 1772, 1709, 1606, 1523, 1453, 1343, 1183, 1047, 962, 861, 724, 529 cm$^{-1}$; HRMS (ESI-TOF) *m/z:* $[M + H]^+$ calcd for $C_{19}H_{17}N_6O_4$, 393.1306; found, 393.1299.

**5-Nitro-2-(3-(5-phenyl-2*H*-tetrazol-2-yl)butyl)benzo[*d*]isothiazol-3(2*H*)-one 1,1-dioxide (16a)**

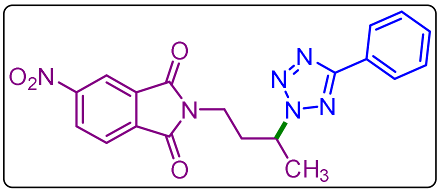


The title compound was obtained according to the general procedure, (Method A), in acetonitrile solvent. Colorless solid (137 mg, 70% yield); $R_f$ = 0.43 (2:8 EtOAc:hexane, silica gel); $^1H$ NMR (600 MHz, $CDCl_3$): $\delta$ 8.44 (d, $J$ = 1.6 Hz, 1H), 8.39 (dd, $J$ = 8.1, 2.0 Hz, 1H), 7.96–7.92 (m, 2H), 7.85 (d, $J$ = 8.4 Hz, 1H), 7.37 (dd, $J$ = 5.1, 1.9 Hz, 3H), 5.11–5.05 (m, 1H), 3.83 (ddd, $J$ = 8.0, 6.4, 2.4 Hz, 2H), 2.76–2.69 (m, 1H), 2.37–2.31 (m, 1H), 1.70 (d, $J$ = 6.8 Hz, 3H) ppm; $^{13}C$ {$^1H$} NMR (151 MHz, $CDCl_3$): $\delta$ 165.8, 165.6, 164.9, 151.6, 136.1, 133.1, 131.5, 130.5, 129.4, 129.3, 128.9, 127.1, 126.7, 124.5, 118.8, 59.1, 35.8, 33.7, 21.3 ppm; IR (KBr): 3109, 2931, 2866, 1778, 1716, 1623, 1538, 1448, 1395, 1344, 1186, 1070, 965, 836, 720, 603, 515 cm$^{-1}$; HRMS (ESI-TOF) *m/z:* $[M + H]^+$ calcd for $C_{19}H_{17}N_6O_4$, 393.1306; found, 393.1309.

**2-(4-(5-Phenyl-2*H*-tetrazol-2-yl)pentyl)isoindoline-1,3-dione (17a) + 2-(3-(5-Phenyl-2*H*-tetrazol-2-yl)pentyl)isoindoline-1,3-dione (17'a)**

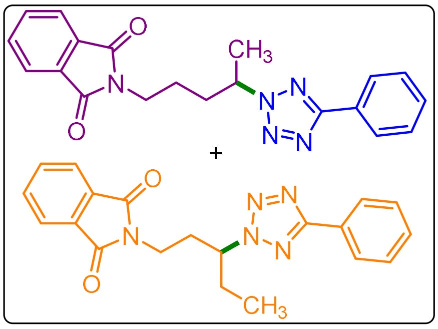


The title compound was obtained according to the general procedure, (Method A), in acetonitrile solvent. Gummy (121 mg, 67% yield); $R_f$ = 0.36 (2:8 EtOAc:hexane, silica gel); $^1H$ NMR (600 MHz, $CDCl_3$): $\delta$ 8.11–8.06 (m, 2H), 8.06–8.04 (m, 0.56H), 7.80–7.77 (m, 2H), 7.74–7.71 (m, 0.54H), 7.68–7.65 (m, 2H), 7.62–7.59 (m, 0.5H), 7.46–7.38 (m, 3.83H), 5.02 (m, 1H), 4.85–4.81 (m, 0.26H), 3.67 (m, 2.56H), 2.62–2.56 (m, 0.26H), 2.36–2.31 (m, 0.26H), 2.19–2.11 (m, 1H), 2.10–1.99 (m, 0.55H), 1.94–1.90 (m, 1H), 1.69–1.65 (m, 1H), 1.64 (d, $J$ = 6.8 Hz, 3H), 1.57–1.50 (m, 1H), 0.80 (t, $J$ = 7.4 Hz, 0.8H) ppm; $^{13}C$ {$^1H$} NMR (151 MHz, $CDCl_3$) δ 168.5, 168.1, 165.1, 165.0, 134.12, 134.10, 132.1, 131.9, 130.3, 129.1, 128.92, 128.87, 127.7, 127.6, 126.9, 123.40, 123.38, 64.7, 60.3, 37.2, 34.97, 33.5, 32.7, 28.4, 25.1, 20.7, 10.3 ppm; IR (KBr): 3047, 2936, 2844, 1761, 1723, 1602, 1430, 1365, 1152, 983, 827,

779, 713, 605 $cm^{-1}$; HRMS (ESI-TOF) *m/z:* $[M + H]^+$ calcd for $C_{20}H_{20}N_5O_2$, 362.1612; found, 362.1608.

**2-(5-(5-Phenyl-2*H*-tetrazol-2-yl)hexyl)isoindoline-1,3-dione (18a)**

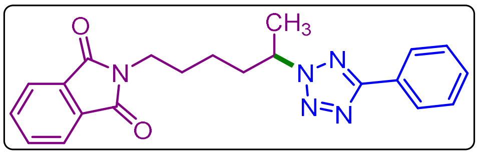


The title compound was obtained according to the general procedure, (Method A), in acetonitrile solvent. Gummy (90 mg, 48% yield); $R_f$ = 0.39 (2:8 EtOAc:hexane, silica gel); $^1H$ NMR (600 MHz, $CDCl_3$): $\delta$ 8.12–8.08 (m, 2H), 7.77 (dd, $J$ = 5.4, 3.0 Hz, 2H), 7.66 (dd, $J$ = 5.5, 3.0 Hz, 2H), 7.46–7.41 (m, 3H), 4.99–4.89 (m, 1H), 3.62 (t, $J$ = 7.2 Hz, 2H), 2.14 (m, 1H), 1.95 (m, 1H), 1.74–1.65 (m, 2H), 1.64 (d, $J$ = 6.8 Hz, 3H), 1.36–1.29 (m, 1H), 1.21–1.15 (m, 1H) ppm; $^{13}C$ {$^1H$} NMR (151 MHz, $CDCl_3$): $\delta$ 168.5, 165.0, 134.1, 132.2, 130.3, 128.9, 127.8, 127.0, 123.4, 60.8, 37.6, 35.8, 28.1, 23.2, 20.8 ppm; IR (KBr): 3053, 2932, 2823, 1756, 1711, 1613, 1418, 1354, 1143, 976, 819, 732, 600 $cm^{-1}$; HRMS (ESI-TOF) *m/z:* $[M + H]^+$ calcd for $C_{21}H_{22}N_5O_2$, 376.1768; found, 376.1769.

**5-Phenyl-2-(4-(phenylsulfonyl)butan-2-yl)-2*H*-tetrazole (19a)**

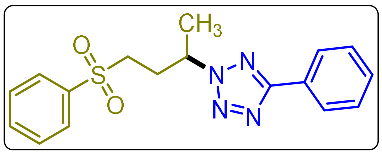


The title compound was obtained according to the general procedure, (Method A), in acetonitrile solvent. Gummy (117 mg, 68% yield); $R_f$ = 0.24 (2:8 EtOAc:hexane, silica gel); $^1H$ NMR (600 MHz, $CDCl_3$): $\delta$ 8.06–8.00 (m, 2H), 7.83–7.77 (m, 2H), 7.58 (t, $J$ = 7.4 Hz, 1H), 7.48 (t, $J$ = 7.8 Hz, 2H), 7.44–7.39 (m, 3H), 5.09–5.03 (m, 1H), 3.07 (ddd, $J$ = 14.2, 10.0, 5.6 Hz, 1H), 2.91 (ddd, $J$ = 14.1, 10.1, 5.4 Hz, 1H), 2.46 (ddd, $J$ = 14.7, 10.1, 5.4 Hz, 1H), 2.39 (ddd, $J$ = 14.7, 10.1, 5.2 Hz, 1H), 1.65 (d, $J$ = 6.8 Hz, 3H) ppm; $^{13}C$ {$^1H$} NMR (151 MHz, $CDCl_3$): $\delta$ 165.3, 138.8, 134.2, 130.6, 129.6, 129.0, 128.1, 127.3, 127.0, 59.2, 52.7, 29.2, 20.7 ppm; IR (KBr): 3063, 2984, 2932, 1529, 1448, 1306, 1145, 1084, 1022, 923, 792, 734, 692, 593, 534 $cm^{-1}$; HRMS (ESI-TOF) *m/z:* $[M + H]^+$ calcd for $C_{17}H_{19}N_4O_2S$, 343.1223; found, 343.1222.

**2-(4-((4-Nitrophenyl)sulfonyl)butan-2-yl)-5-phenyl-2*H*-tetrazole (20a)**

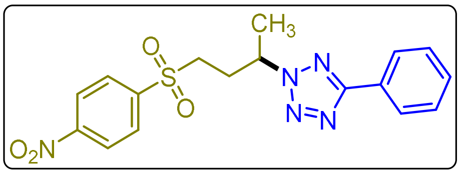


The title compound was obtained according to the general procedure, (Method A), in acetonitrile solvent. Gummy (140 mg, 72% yield); $R_f$ = 0.20 (2:8 EtOAc:hexane, silica gel); $^1H$ NMR (600 MHz, $CDCl_3$): $\delta$ 8.34 (d, $J$ = 8.8 Hz, 2H), 8.05–8.01 (m, 4H), 7.47–7.43 (m, 3H), 5.15–5.07 (m, 1H),

3.19–3.14 (m, 1H), 3.01–2.92 (m, 1H), 2.55–2.42 (m, 2H), 1.70 (d, $J$ = 6.8 Hz, 3H) ppm; $^{13}C$ {$^{1}H$} NMR (151 MHz, $CDCl_3$): $\delta$ 165.4, 151.1, 144.2, 130.8, 129.7, 129.1, 127.1, 126.9, 124.8, 59.0, 52.5, 28.9, 20.8 ppm; IR (KBr): 2959, 2962, 2837, 1533, 1449, 1309, 1272, 1176, 1128, 1019, 817, 724, 683, 527 cm$^{-1}$; HRMS (ESI-TOF) *m/z:* $[M + H]^+$ calcd for $C_{17}H_{18}N_5O_4S$, 388.1074; found, 388.1080.

**2-(4-(Butylsulfonyl)butan-2-yl)-5-phenyl-2*H*-tetrazole (21a)**

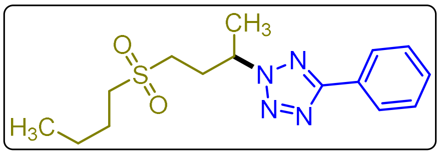


The title compound was obtained according to the general procedure, (Method A), in acetonitrile solvent. Gummy (121 mg, 75% yield); $R_f$ = 0.42 (3:7 EtOAc:hexane, silica gel); $^{1}H$ NMR (600 MHz, $CDCl_3$): $\delta$ 8.12 (dd, $J$ = 7.6, 2.0 Hz, 2H), 7.51–7.41 (m, 3H), 5.18–5.12 (m, 1H), 2.97–2.87 (m, 3H), 2.82–2.77 (m, 1H), 2.64–2.57 (m, 1H), 2.55–2.48 (m, 1H), 1.80–1.66 (m, 5H), 1.43–1.37 (m, 2H), 0.89 (t, $J$ = 7.4 Hz, 3H) ppm; $^{13}C$ {$^{1}H$} NMR (151 MHz, $CDCl_3$): $\delta$ 165.5, 130.7, 129.1, 127.3, 126.9, 59.4, 53.2, 48.9, 28.3, 24.0, 21.8, 20.9, 13.6 ppm; IR (KBr): 2961, 2935, 2874, 1529, 1453, 1313, 1280, 1187, 1132, 1023, 915, 736, 697, 510 cm$^{-1}$; HRMS (ESI-TOF) *m/z:* $[M + H]^+$ calcd for $C_{15}H_{23}N_4O_2S$, 323.1536; found, 323.1535.

**2-(3-(5-Phenyl-2*H*-tetrazol-2-yl)butyl)benzo[*d*]isothiazol-3(2*H*)-one 1,1-dioxide (22a)**

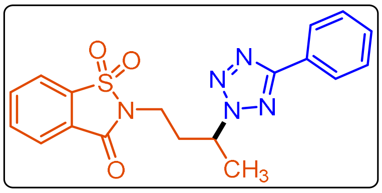


The title compound was obtained according to the general procedure, (Method A), in acetonitrile solvent. Colorless solid (148 mg, 77% yield); $R_f$ = 0.46 (3:7 EtOAc:hexane, silica gel); $^{1}H$ NMR (600 MHz, $CDCl_3$): $\delta$ 8.13 (dd, $J$ = 8.0, 1.6 Hz, 2H), 8.02 (d, $J$ = 7.7, Hz, 1H), 7.89 (d, $J$ = 7.4 Hz, 1H), 7.84 (td, $J$ = 7.5, 1.2 Hz, 1H), 7.80 (td, $J$ = 7.5, 1.2 Hz, 1H), 7.47–7.42 (m, 3H), 5.18–5.11 (m, 1H), 3.87 (dt, $J$ = 14.7, 7.3 Hz, 1H), 3.78–3.72 (m, 1H), 2.75–2.67 (m, 1H), 2.51–2.44 (m, 1H), 1.72 (d, $J$ = 6.8 Hz, 3H) ppm; $^{13}C$ {$^{1}H$} NMR (151 MHz, $CDCl_3$): $\delta$ 165.2, 159.0, 137.7, 135.1, 134.6, 130.4, 129.0, 127.6, 127.3, 127.1, 125.5, 121.1, 58.3, 36.0, 34.5, 20.9 ppm; IR (KBr): 2953, 2904, 2852, 1519, 1424, 1309, 1266, 1173, 1091, 953, 749, 543 cm$^{-1}$; HRMS (ESI-TOF) *m/z:* $[M + H]^+$ calcd for $C_{18}H_{18}N_5O_3S$, 384.1125; found, 384.1127.

**Methyl 5-(5-phenyl-2*H*-tetrazol-2-yl)hexanoate (24a)**

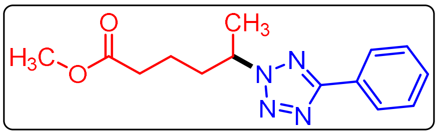


The title compound was obtained according to the general procedure, (Method A). Gummy (91 mg, 66% yield); $R_f$ = 0.48 (1:9 EtOAc:hexane, silica gel); $^1H$ NMR (600 MHz, $CDCl_3$): $\delta$ 8.12 (d, $J$ = 8.1 Hz, 2H), 7.48–7.42 (m, 3H), 5.00–4.93 (m, 1H), 3.62 (s, 3H), 2.30 (t, $J$ = 7.4 Hz, 2H), 2.17–2.09 (m, 1H), 1.98–1.91 (m, 1H), 1.69 (d, $J$ = 6.8 Hz, 3H), 1.63–1.58 (m, 1H), 1.50–1.42 (m, 1H) ppm; $^{13}C$ {$^1H$} NMR (151 MHz, $CDCl_3$): $\delta$ 173.5, 165.0, 130.3, 129.0, 127.7, 127.0, 60.6, 51.8, 35.7, 33.4, 21.4, 20.8 ppm; IR (KBr): 3073, 3037, 2950, 2919, 2849, 1738, 1529, 1467, 1450, 1379, 1339, 1255, 1175, 1072, 1024, 926, 789, 734, 695 $cm^{-1}$; HRMS (ESI-TOF) *m/z:* $[M + H]^+$ calcd for $C_{14}H_{19}N_4O_2$, 275.1503; found, 275.1497.

**4-(5-Phenyl-2*H*-tetrazol-2-yl)pentanenitrile (25a)**

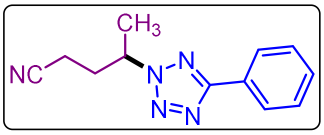


The title compound was obtained according to the general procedure, (Method A). Gummy (68 mg, 60% yield); $R_f$ = 0.30 (2:8 EtOAc:hexane, silica gel); $^1H$ NMR (600 MHz, $CDCl_3$): $\delta$ 8.12 (dd, $J$ = 7.3, 2.4 Hz, 2H), 7.50–7.43 (m, 3H), 5.17–5.05 (m, 1H), 2.54–2.45 (m, 1H), 2.35–2.24 (m, 3H), 1.72 (d, $J$ = 6.8 Hz, 3H) ppm; $^{13}C$ {$^1H$} NMR (151 MHz, $CDCl_3$): $\delta$ 165.5, 130.6, 129.0, 127.3, 126.9, 118.3, 59.3, 31.9, 20.6, 14.4 ppm; IR (KBr): 3070, 2986, 2918, 2849, 2247, 1603, 1529, 1467, 1450, 1348, 1279, 1175, 1071, 1024, 925, 789, 733, 694 $cm^{-1}$; HRMS (ESI-TOF) *m/z:* $[M + H]^+$ calcd for $C_{12}H_{14}N_5$, 228.1244; found, 228.1244.

**Dibutyl (3-(5-phenyl-2*H*-tetrazol-2-yl)butyl) phosphate (27a)**

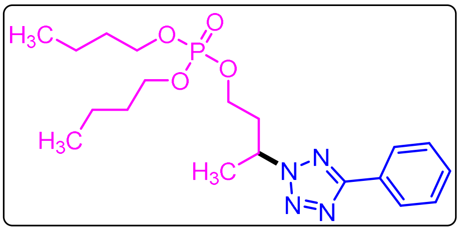


The title compound was obtained according to the general procedure, (Method A). Colorless oil (166 mg, 81% yield); $R_f$ = 0.27 (3:7 EtOAc:hexane, silica gel); $^1H$ NMR (600 MHz, $CDCl_3$): $\delta$ 8.11 (dd, $J$ = 7.9, 1.7 Hz, 2H), 7.47–7.42 (m, 3H), 5.24–5.17 (m, 1H), 4.09–4.04 (m, 1H), 4.01–3.94 (m, 4H), 3.90–3.85 (m, 1H), 2.52–2.46 (m, 1H), 2.32–2.25 (m, 1H), 1.68 (d, $J$ = 6.8 Hz, 3H), 1.62–1.55 (m, 4H), 1.37–1.30 (m, 4H), 0.89–0.85 (m, 6H) ppm; $^{13}C$ {$^1H$} NMR (151 MHz, $CDCl_3$): $\delta$ 165.1, 130.4, 129.0, 127.6, 126.9, 67.8 (dd, $J$ = 6.0, 1.8 Hz), 63.6 (d, $J$ = 5.8 Hz), 57.3, 36.6 (d, $J$ = 7.2 Hz), 32.4 (dd, $J$ = 6.8, 2.7 Hz), 20.8, 18.7 (d, $J$ = 2.3 Hz), 13.7 (d, $J$ = 2.1 Hz) ppm; $^{31}P$ {$^1H$} NMR (162 MHz, $CDCl_3$): $\delta$ –0.98 ppm; $^{31}P$ NMR (162 MHz, $CDCl_3$): $\delta$ –0.99 (p, $J$ = 7.1 Hz) ppm;

IR (KBr): 2960, 2930, 2871, 1452, 1384, 1270, 1025, 916, 805, 733, 696, 544 cm$^{-1}$; HRMS (ESI-TOF) *m/z:* $[M + H]^+$ calcd for $C_{19}H_{32}N_4O_4P$, 411.2156; found, 411.2153.

**Dibutyl (3-(5-(4-methoxyphenyl)-2*H*-tetrazol-2-yl)butyl) phosphate (27e)**

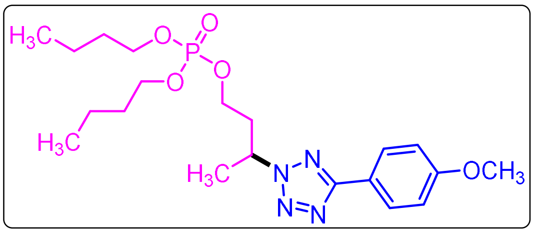


The title compound was obtained according to the general procedure, (Method A). Colorless oil (161 mg, 73% yield); $R_f$ = 0.20 (3:7 EtOAc:hexane, silica gel); $^1H$ NMR (600 MHz, $CDCl_3$): $\delta$ 8.04 (d, $J$ = 8.9 Hz, 2H), 6.96 (d, $J$ = 8.9 Hz, 2H), 5.21–5.13 (m, 1H), 4.08–4.03 (m, 1H), 4.02–3.94 (m, 4H), 3.89–3.84 (m, 1H), 3.83 (s, 3H), 2.52–2.44 (m, 1H), 2.30–2.24 (m, 1H), 1.66 (d, $J$ = 6.8 Hz, 3H), 1.62–1.55 (m, 4H), 1.36–1.29 (m, 4H), 0.89–0.85 (m, 6H), ppm; $^{13}C$ {$^1H$} NMR (151 MHz, $CDCl_3$): $\delta$ 165.0, 161.3, 128.4, 120.2, 114.4, 67.8 (dd, $J$ = 6.1 Hz), 63.6 (d, $J$ = 5.7 Hz), 57.2, 55.5, 36.6 (d, $J$ = 7.3 Hz), 32.4 (dd, $J$ = 6.8, 2.4 Hz), 20.8, 18.6 (d, $J$ = 1.9 Hz), 13.7 (d, $J$ = 1.7 Hz) ppm; $^{31}P$ {$^1H$} NMR (162 MHz, $CDCl_3$): $\delta$ –0.999 ppm; $^{31}P$ NMR (162 MHz, $CDCl_3$): $\delta$ –0.99 (p, $J$ = 6.9 Hz) ppm; IR (KBr): 2960, 2933, 2873, 1615, 1464, 1254, 1177, 1027, 913, 842, 763, 536 cm$^{-1}$; HRMS (ESI-TOF) *m/z:* $[M + H]^+$ calcd for $C_{20}H_{34}N_4O_5P$, 441.2261; found, 441.2269.

**Dibutyl (3-(5-(4-(trifluoromethyl)phenyl)-2*H*-tetrazol-2-yl)butyl) phosphate (27j)**

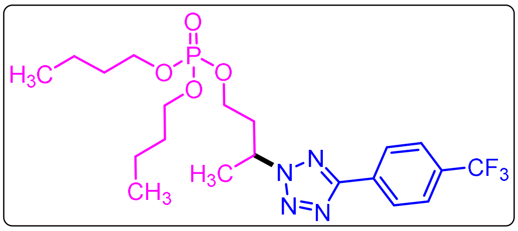


The title compound was obtained according to the general procedure, (Method A). Colorless oil (206 mg, 86% yield); $R_f$ = 0.29 (3:7 EtOAc:hexane, silica gel); $^1H$ NMR (600 MHz, $CDCl_3$): $\delta$ 8.24 (d, $J$ = 8.1 Hz, 2H), 7.72 (d, $J$ = 8.2 Hz, 2H), 5.27–5.20 (m, 1H), 4.08 (dq, $J$ = 11.3, 6.0 Hz, 1H), 3.99 (ddd, $J$ = 13.3, 6.7, 3.1 Hz, 4H), 3.92–3.86 (m, 1H), 2.53–2.46 (m, 1H), 2.34–2.26 (m, 1H), 1.70 (d, $J$ = 6.8 Hz, 3H), 1.62–1.56 (m, 4H), 1.37–1.31 (m, 4H), 0.87 (q, $J$ = 7.5 Hz, 6H) ppm; $^{13}C$ {$^1H$} NMR (151 MHz, $CDCl_3$): $\delta$ 163.9, 132.2 (q, $J$ = 32.62 Hz), 130.9, 127.3, 126.0 (q, $J$ = 3.7 Hz), 124.0 (q, $J$ = 272.41 Hz), 67.8 (dd, $J$ = 6.1, 1.7 Hz), 63.5 (d, $J$ = 5.7 Hz), 57.6, 36.6 (d, $J$ = 7.2 Hz), 32.4 (dd, $J$ = 6.9, 2.1 Hz), 20.8, 18.8 (d, $J$ = 1.8 Hz), 13.7 (d, $J$ = 1.8 Hz) ppm; $^{31}P$ {$^1H$} NMR (162 MHz, $CDCl_3$): $\delta$ –0.97 ppm; $^{19}F$ {$^1H$} NMR (377 MHz, $CDCl_3$): $\delta$ –62.88 ppm; IR (KBr): 2963, 2932, 2874, 1547, 1466, 1429, 1322, 1167, 1126, 1066, 1016, 852, 763, 599 cm$^{-1}$; HRMS (ESI-TOF) *m/z:* $[M + H]^+$ calcd for $C_{20}H_{31}F_3N_4O_4P$, 479.2030; found, 479.2025.

***N*-Butylbenzamide (28'a)**[9]

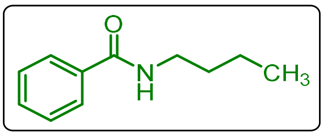


The title compound was obtained according to the general procedure, (Method A). White solid (75 mg, 84% yield); $R_f$ = 0.45 (2:8 EtOAc:hexane, silica gel); $^1$H NMR (400 MHz, $CDCl_3$): $\delta$ 7.75–7.69 (m, 2H), 7.43–7.37 (m, 1H), 7.33 (t, $J$ = 7.4 Hz, 2H), 6.60 (s, 1H), 3.36 (td, $J$ = 7.2, 5.7 Hz, 2H), 1.60–1.46 (m, 2H), 1.32 (dq, $J$ = 14.5, 7.3 Hz, 2H), 0.87 (t, $J$ = 7.3 Hz, 3H) ppm; $^{13}$C NMR (101 MHz, $CDCl_3$): $\delta$ 167.8, 134.9, 131.4, 128.6, 127.0, 39.9, 31.9, 20.3, 13.9 ppm; IR (KBr): 3325, 1640, 1548, 1401, 1329, 1034, 805, 710, 648 cm$^{-1}$; HRMS (ESI-TOF) *m/z:* $[M + H]^+$ calcd for $C_{11}H_{16}NO$, 178.1226; found, 178.1232.

***N*-Methoxy-*N*-((5-phenyl-2*H*-tetrazol-2-yl)methyl)pentanamide (29'a)**

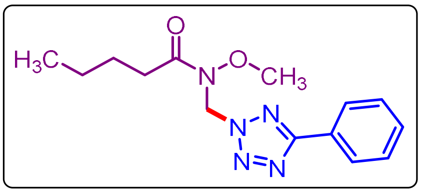


The title compound was obtained according to the general procedure, (Method A). Gummy (44 mg, 30% yield); $R_f$ = 0.56 (2:8 EtOAc:hexane, silica gel); $^1$H NMR (400 MHz, $CDCl_3$): $\delta$ 8.16–8.10 (m, 2H), 7.47-7.42 (m, 3H), 6.30 (s, 2H), 3.72 (s, 3H), 2.50 (t, $J$ = 7.5 Hz, 2H), 1.67–1.60 (m, 2H), 1.40–1.31 (m, 2H), 0.89 (t, $J$ = 7.4 Hz, 3H) ppm; $^{13}$C NMR (101 MHz, $CDCl_3$): $\delta$ 176.2, 165.8, 130.7, 129.0, 128.9, 127.2, 77.4, 63.4, 32.0, 29.8, 26.3, 22.5, 13.9ppm; IR (KBr): 3006, 2921, 2838, 1660, 1611, 1530, 1447, 1416, 1390, 1241, 1034, 910, 738 cm$^{-1}$; HRMS (ESI-TOF) *m/z:* $[M + H]^+$ calcd for $C_{14}H_{20}N_5O_2$, 290.1612; found, 290.1618.

***N*-Methyl-*N*-((5-phenyl-2*H*-tetrazol-2-yl)methoxy)pentanamide (29''a) + Uncharacterised inseparable impurity**

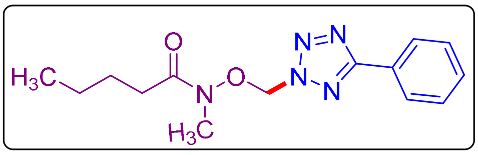


The title compound was obtained according to the general procedure, (Method A). Gummy (64 mg, 44% yield); $R_f$ = 0.42 (2:8 EtOAc:hexane, silica gel); $^1$H NMR (400 MHz, $CDCl_3$): $\delta$ 7.69–7.65 (m, 2H), 7.54–7.47 (m, 3H), 6.09 (s, 2H), 3.52 (s, 3H), 2.35 (t, $J$ = 7.6 Hz, 2H), 1.52–1.43 (m, 2H), 1.28-1.20 (m, 2H), 0.83–0.80 (m, 3H) ppm; $^{13}$C NMR (101 MHz, $CDCl_3$): $\delta$ 173.5, 165.5, 131.7, 129.4, 129.2, 127.0, 77.4, 36.1, 31.9, 26.2, 22.4, 13.9 ppm; IR (KBr): 3018, 2929, 2814, 1699, 1503, 1449, 1377, 1286, 1042, 960, 823, 754 cm$^{-1}$; cm$^{-1}$; HRMS (ESI-TOF) *m/z:* $[M + H]^+$ calcd for $C_{14}H_{20}N_5O_2$, 290.1612; found, 290.1621.

**1-Phenyl-2-(5-phenyl-2*H*-tetrazol-2-yl)pentan-1-one (30a)**

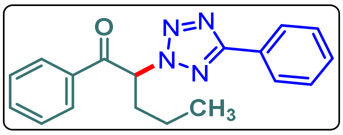

The title compound was obtained according to the general procedure, (Method B). Gummy (123 mg, 80% yield); $R_f$ = 0.60 (1:9 EtOAc:hexane, silica gel); $^1$H NMR (400 MHz, $CDCl_3$): $\delta$ 8.16–8.08 (m, 2H), 7.95 (dd, $J$ = 8.4, 1.4 Hz, 2H), 7.57 (t, $J$ = 7.4 Hz, 1H), 7.48–7.40 (m, 5H), 6.42 (dd, $J$ = 10.2, 4.4 Hz, 1H), 2.60–2.46 (m, 1H), 2.36–2.27 (m, 1H), 1.47–1.03 (m, 2H), 0.98 (t, $J$ = 7.4 Hz, 3H) ppm; $^{13}$C NMR (101 MHz, $CDCl_3$): $\delta$ 192.1, 165.4, 134.4, 134.3, 130.5, 129.2, 128.9, 128.8, 127.4, 127.1, 67.4, 32.9, 19.5, 13.6 ppm; IR (KBr): 3066, 3030, 2950, 2893, 1690, 1699, 1601, 1463,1423, 1386, 1377, 1289, 1258, 1176, 1019, 973, 746, 683, 559 cm$^{-1}$; HRMS (ESI-TOF) *m/z:* $[M + H]^+$ calcd for $C_{18}H_{19}N_4O$, 307.1553; found, 307.1552.

**1-(5-Phenyl-2*H*-tetrazol-2-yl)propan-2-one (31a)**[10]

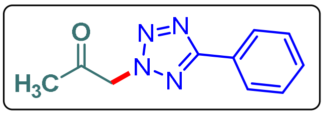

The title compound was obtained according to the general procedure (Method B) in pressure tube. White solid (75 mg, 74% yield); $R_f$ = 0.61 (2:8 EtOAc:hexane, silica gel); $^1$H NMR (600 MHz, $CDCl_3$): $\delta$ 8.15 (d, $J$ = 8.3 Hz, 2H), 7.51–7.46 (m, 3H), 5.48 (s, 2H), 2.24 (s, 3H) ppm; $^{13}$C NMR (101 MHz, $CDCl_3$): $\delta$ 197.9, 165.7, 130.6, 129.0, 127.0, 126.9, 61.0, 27.2 ppm; IR (KBr): 3062, 2951, 2939, 2855, 1759, 1472, 1350, 1238, 1177, 1010, 992, 851, 750, 682, 630 cm$^{-1}$; HRMS (ESI-TOF) *m/z:* $[M + H]^+$ calcd for $C_{10}H_{11}N_4O$, 203.0927; found, 203.0935.

**2-(5-Phenyl-2*H*-tetrazol-2-yl)octan-3-one (32a)**

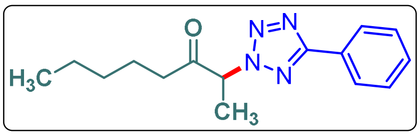

The title compound was obtained according to the general procedure, (Method B). Gummy (48 mg, 35% yield); $R_f$ = 0.63 (2:8 EtOAc:hexane, silica gel); $^1$H NMR (600 MHz, $CDCl_3$): $\delta$ 8.14 (dd, $J$ = 7.7, 1.9 Hz, 2H), 7.48–7.45 (m, 3H), 5.53 (q, $J$ = 7.3 Hz, 1H), 2.26 (t, $J$ = 7.3 Hz, 2H), 1.89 (d, $J$ = 7.3 Hz, 3H), 1.55–1.50 (m, 2H), 1.25–1.19 (m, 2H), 1.19–1.13 (m, 2H), 0.81 (t, $J$ = 7.2 Hz, 3H) ppm; $^{13}$C {$^1$H} NMR (151 MHz, $CDCl_3$): $\delta$ 203.8, 165.6, 130.7, 129.1, 127.3, 127.1, 67.3, 38.6, 31.2, 23.0, 22.5, 16.0, 14.0 ppm; IR (KBr): 2964, 2937, 2854, 1733, 1483, 1413, 1358, 1226, 1170, 1011, 985, 854, 763, 728, 691, 637 cm$^{-1}$; HRMS (ESI-TOF) *m/z:* $[M + H]^+$ calcd for $C_{15}H_{21}N_4O$, 273.1710; found, 273.1695.

**4-(5-Phenyl-2*H*-tetrazol-2-yl)octan-3-one (32'a)**

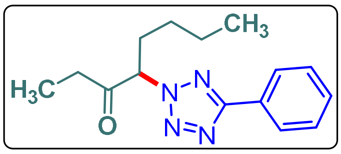


The title compound was obtained according to the general procedure, (Method B). Gummy (60 mg, 44% yield); $R_f$ = 0.60 (2:8 EtOAc:hexane, silica gel); $^1$H NMR (600 MHz, $CDCl_3$): $\delta$ 8.18 (dd, *J* = 7.7, 1.9 Hz, 2H), 7.52–7.48 (m, 3H), 5.44 (dd, *J* = 10.4, 4.7 Hz, 1H), 2.42–2.25 (m, 4H), 1.43–1.27 (m, 4H), 1.03 (t, *J* = 7.2 Hz, 3H), 0.88 (t, *J* = 7.2 Hz, 3H) ppm; $^{13}$C {$^1$H} NMR (151 MHz, $CDCl_3$): $\delta$ 204.2, 165.6, 130.7, 129.1, 127.3, 127.1, 72.1, 32.5, 29.9, 28.0, 22.1, 13.8, 7.4 ppm; IR (KBr): 2960, 2926, 2854, 1734, 1466, 1450, 1358, 1181, 1047, 1022, 788, 733, 693, 665 cm$^{-1}$; HRMS (ESI-TOF) *m/z:* [M + H]$^+$ calcd for $C_{15}H_{21}N_4O$, 273.1710; found, 273.1699.

**6-Methoxy-4-(5-phenyl-2*H*-tetrazol-2-yl)-3,4-dihydronaphthalen-1(2*H*)-one (33a)**

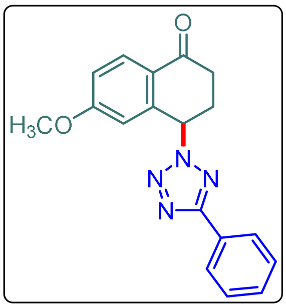


The title compound was obtained according to the general procedure, (Method B). Colorless solid (133 mg, 83% yield); $R_f$ = 0.34 (2:8 EtOAc:hexane, silica gel); $^1$H NMR (600 MHz, $CDCl_3$): $\delta$ 8.15–8.11 (m, 3H), 7.49–7.45 (m, 3H), 6.99 (dd, *J* = 8.8, 2.4 Hz, 1H), 6.45 (d, *J* = 2.3 Hz, 1H), 6.32 (dd, *J* = 7.3, 4.5 Hz, 1H), 3.77 (s, 3H), 3.13–3.07 (m, 1H), 2.96–2.88 (m, 1H), 2.80–2.69 (m, 2H) ppm; $^{13}$C {$^1$H} NMR (151 MHz, $CDCl_3$): $\delta$ 194.6, 165.6, 164.3, 140.7, 130.6, 130.4, 129.0, 127.2, 127.0, 125.7, 115.6, 112.3, 61.6, 55.7, 35.1, 29.1 ppm; IR (KBr): 3070, 2926, 2853, 1725, 1683, 1601, 1529, 1495, 1466, 1450, 1335, 1244, 1153, 1024, 905, 795, 734, 695, 552 cm$^{-1}$; HRMS (ESI-TOF) *m/z:* [M + H]$^+$ calcd for $C_{18}H_{17}N_4O_2$, 321.1346; found, 321.1336.

**4-(1-(5-Phenyl-2*H*-tetrazol-2-yl)pentyl)pyridine (34a)**

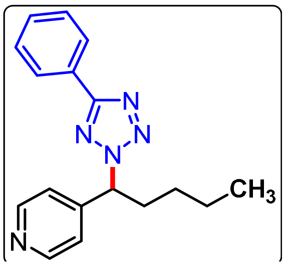


The title compound was obtained according to the general procedure, (Method B). Yellowish liquid (95 mg, 65% yield); $R_f$ = 0.48 (3:7 EtOAc:hexane, silica gel); $^1$H NMR (600 MHz, $CDCl_3$): $\delta$ 8.62 (d, *J* = 6.1 Hz, 2H), 8.16 (dd, *J* = 7.7, 1.8 Hz, 2H), 7.50–7.46 (m, 3H), 7.35 (dd, *J* = 4.6, 1.5 Hz, 2H), 5.89 (dd, *J* = 9.4, 6.1 Hz, 1H), 2.65–2.58 (m, 1H), 2.33–2.26 (m, 1H), 1.43–1.34 (m, 2H), 1.34–1.27 (m, 1H), 1.25–1.19 (m, 1H), 0.89 (t, *J* = 7.3 Hz, 3H) ppm; $^{13}$C {$^1$H} NMR (151 MHz, $CDCl_3$): $\delta$ 165.4, 150.6, 146.7, 130.6, 129.1, 127.3, 127.0, 122.0, 67.4, 34.9, 28.3, 22.2, 13.9 ppm; IR (KBr): 3031, 2955, 2928, 2864, 1598, 1451, 1415, 1338, 1276, 1197, 1024, 734,

694, 620, 567 cm$^{-1}$; HRMS (ESI-TOF) *m/z:* $[M + H]^+$ calcd for $C_{17}H_{20}N_5$, 294.1713; found, 294.1709.

**3-(5-Phenyl-2*H*-tetrazol-2-yl)butan-1-ol (35a)**[11]

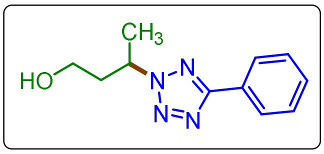


The title compound was obtained according to the general procedure, (Method C). Gummy (69 mg, 63% yield); $R_f = 0.25$ (3:7 EtOAc:hexane, silica gel); $^1H$ NMR (600 MHz, $CDCl_3$): $\delta$ 8.14 (d, $J = 7.1$ Hz, 2H), 7.51–7.44 (m, 3H), 5.33–5.25 (m, 1H), 3.69 (dt, $J = 10.8$, 5.3 Hz, 1H), 3.49 (ddd, $J = 11.2$, 8.6, 4.6 Hz, 1H), 2.34 (ddt, $J = 14.4$, 9.6, 4.9 Hz, 1H), 2.19 (ddt, $J = 13.9$, 8.7, 5.2 Hz, 1H), 1.72 (d, $J = 6.8$ Hz, 3H) ppm; $^{13}C\ \{^1H\}$ NMR (151 MHz, $CDCl_3$): $\delta$ 165.1, 130.4, 129.0, 127.6, 126.9, 58.8, 57.7, 38.8, 20.8 ppm; IR (KBr): 3354, 3066, 2931, 2863, 1641, 1459, 1352, 1244, 1190, 1061, 923, 787, 730, 695 cm$^{-1}$; HRMS (ESI-TOF): *m/z* calcd. for $C_{11}H_{15}N_4O$ $[M + H]^+$ 219.1240; found 219.1249.

**3-(5-(Naphthalen-2-yl)-2*H*-tetrazol-2-yl)butan-1-ol** (**35b**)

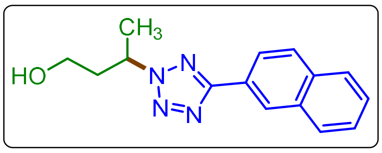


The title compound was obtained according to the general procedure, (Method C). Gummy (79 mg, 59% yield); $R_f = 0.27$ (3:7 EtOAc:hexane, silica gel); $^1H$ NMR (600 MHz, $CDCl_3$): $\delta$ 8.69 (s, 1H), 8.22 (dd, $J = 8.5$, 1.5 Hz, 1H), 7.96 (d, $J = 8.4$ Hz, 2H), 7.90–7.86 (m, 1H), 7.56–7.52 (m, 2H), 5.39–5.27 (m, 1H), 3.72 (dt, $J = 10.8$, 5.3 Hz, 1H), 3.52 (ddd, $J = 11.2$, 8.6, 4.5 Hz, 1H), 2.38 (ddd, $J = 19.3$, 9.6, 4.9 Hz, 1H), 2.22 (ddt, $J = 14.0$, 8.6, 5.2 Hz, 1H), 1.76 (d, $J = 6.8$ Hz, 3H) ppm; $^{13}C\ \{^1H\}$ NMR (151 MHz, $CDCl_3$): $\delta$ 165.2, 134.3, 133.3, 128.85, 128.80, 128.0, 127.2, 126.8, 126.7, 124.9, 124.1, 58.8, 57.8, 38.8, 20.8 ppm; IR (KBr): 3339, 3050, 2956, 2930, 2877, 1460, 1373, 1074, 953, 831, 760 cm$^{-1}$; HRMS (ESI-TOF): *m/z* calcd. for $C_{15}H_{17}N_4O$ $[M + H]^+$ 269.1397; found 269.1403.

**3-(5-([1,1'-Biphenyl]-4-yl)-2*H*-tetrazol-2-yl)butan-1-ol** (**35c**)

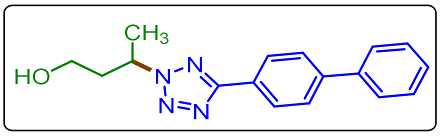


The title compound was obtained according to the general procedure, (Method C). Gummy (88 mg, 60% yield); $R_f = 0.23$ (3:7 EtOAc:hexane, silica gel); $^1H$ NMR (600 MHz, $CDCl_3$): $\delta$ 8.22 (d, $J = 8.4$ Hz, 2H), 7.72 (d, $J = 8.4$ Hz, 2H), 7.65 (d, $J = 7.2$ Hz, 2H), 7.47 (t, $J = 7.6$ Hz, 2H), 7.39 (t, $J = 7.4$ Hz, 1H), 5.34–5.26 (m, 1H), 3.71 (dt, $J = 10.9$, 5.3 Hz, 1H), 3.50 (ddd, $J = 11.2$, 8.6, 4.5 Hz, 1H), 2.36 (ddd, $J = 19.2$, 9.6, 4.9 Hz, 1H), 2.20 (ddt, $J = 14.1$, 8.6, 5.1 Hz, 1H), 1.74 (d, $J = 6.8$ Hz, 3H) ppm; $^{13}C\ \{^1H\}$ NMR (151 MHz, $CDCl_3$): $\delta$ 164.9, 143.1, 140.4, 129.0, 127.9, 127.7, 127.4, 127.2, 126.5, 58.8, 57.7, 38.8, 20.8

ppm; IR (KBr): 3346, 3030, 2979, 2923, 1625, 1547, 1469, 1379, 1229, 1151, 1049, 916, 847, 717, 643 cm$^{-1}$; HRMS (ESI-TOF): *m/z* calcd. for $C_{17}H_{19}N_4O$ [M + H]$^+$ 295.1553; found 295.1566.

**3-(5-(4-Methoxyphenyl)-2*H*-tetrazol-2-yl)butan-1-ol** (**35e**)

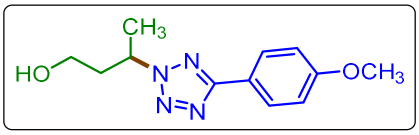


The title compound was obtained according to the general procedure, (Method C). Gummy (70 mg, 56% yield); $R_f$ = 0.24 (3:7 EtOAc:hexane, silica gel); $^1$H NMR (400 MHz, $CDCl_3$): $\delta$ 8.01 (d, $J$ = 8.9 Hz, 2H), 6.94 (d, $J$ = 8.9 Hz, 2H), 5.28–5.12 (m, 1H), 3.81 (s, 3H), 3.62 (dt, $J$ = 10.9, 5.4 Hz, 1H), 3.43 (ddd, $J$ = 11.2, 8.4, 4.7 Hz, 1H), 2.37 (s, 1H, OH-peak), 2.28 (ddd, $J$ = 14.2, 9.5, 4.8 Hz, 1H), 2.11 (ddt, $J$ = 14.0, 8.4, 5.2 Hz, 1H), 1.65 (d, $J$ = 6.8 Hz, 3H) ppm; $^{13}$C {$^1$H} NMR (101 MHz, $CDCl_3$): $\delta$ 164.9, 161.3, 128.5, 120.2, 114.4, 58.8, 57.6, 55.5, 38.8, 20.7 ppm; IR (KBr): 3354, 3094, 2960, 2834, 1603, 1497, 1253, 1180, 1031, 943, 839, 769 cm$^{-1}$; HRMS (ESI-TOF): *m/z* calcd. for $C_{12}H_{17}N_4O_2$ [M + H]$^+$ 249.1346; found 249.1353.

**3-(5-(4-(*tert*-Butyl)phenyl)-2*H*-tetrazol-2-yl)butan-1-ol** (**35f**)

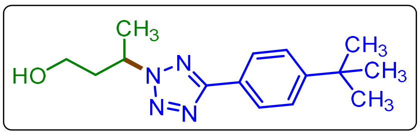


The title compound was obtained according to the general procedure, (Method C). Gummy (80 mg, 58% yield); $R_f$ = 0.38 (3:7 EtOAc:hexane, silica gel); $^1$H NMR (400 MHz, $CDCl_3$): $\delta$ 8.07 (d, $J$ = 8.6 Hz, 2H), 7.51 (d, $J$ = 8.6 Hz, 2H), 5.34–5.21 (m, 1H), 3.69 (dt, $J$ = 10.8, 5.3 Hz, 1H), 3.48 (ddd, $J$ = 11.2, 8.5, 4.7 Hz, 1H), 2.33 (ddd, $J$ = 19.2, 9.7, 4.9 Hz, 1H), 2.23–2.12 (m, 1H), 1.73 (d, $J$ = 6.8 Hz, 3H), 1.36 (s, 9H) ppm; $^{13}$C {$^1$H} NMR (101 MHz, $CDCl_3$): $\delta$ 165.1, 153.7, 126.8, 125.9, 124.9, 58.8, 57.7, 38.9, 35.0, 31.4, 20.7 ppm; IR (KBr): 3375, 2961, 2905, 2874, 1683, 1544, 1470, 1361, 1248, 1159, 1047, 934, 873 cm$^{-1}$; HRMS (ESI-TOF): *m/z* calcd. for $C_{15}H_{23}N_4O$ [M + H]$^+$ 275.1866; found 275.1878.

**3-(5-(3-Methoxyphenyl)-2*H*-tetrazol-2-yl)butan-1-ol** (**35g**)

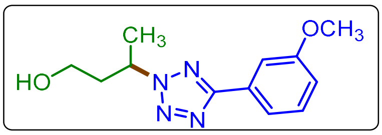


The title compound was obtained according to the general procedure, (Method C). Gummy (66 mg, 53% yield); $R_f$ = 0.26 (3:7 EtOAc:hexane, silica gel); $^1$H NMR (600 MHz, $CDCl_3$): $\delta$ 7.73 (d, $J$ = 7.6 Hz, 1H), 7.68 (s, 1H), 7.39 (t, $J$ = 8.0 Hz, 1H), 7.01 (d, $J$ = 8.8 Hz, 1H), 5.33–5.23 (m, 1H), 3.89 (s, 3H), 3.70–3.66 (m, 1H), 3.49–3.45 (m, 1H), 2.36–2.03 (m, 1H), 2.20–2.14 m, 1H), 1.72 (d, $J$ = 6.8 Hz, 3H) ppm; $^{13}$C {$^1$H} NMR (151 MHz, $CDCl_3$): $\delta$ 164.9, 160.1, 130.1, 128.8, 119.4, 116.8, 111.6, 58.7, 57.7, 55.6, 38.8, 20.8 ppm; IR (KBr):

3362, 3084, 2979, 2847, 1611, 1582, 1491, 1249, 1174, 1025, 941, 869, 764 $cm^{-1}$; HRMS (ESI-TOF): *m/z* calcd. for $C_{12}H_{17}N_4O_2$ $[M + H]^+$ 249.1346; found 249.1354.

**3-(5-(4-Fluorophenyl)-2*H*-tetrazol-2-yl)butan-1-ol** (**35y**)

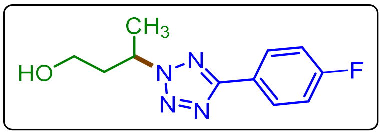


The title compound was obtained according to the general procedure, (Method C). Gummy (77 mg, 65% yield); $R_f$ = 0.25 (3:7 EtOAc:hexane, silica gel; $^1H$ NMR (600 MHz, $CDCl_3$): $\delta$ 8.17–8.08 (m, 2H), 7.20–7.13 (m, 2H), 5.33–5.22 (m, 1H), 3.70 (dt, $J$ = 10.9, 5.3 Hz, 1H), 3.49 (ddd, $J$ = 11.1, 8.5, 4.5 Hz, 1H), 2.33 (ddd, $J$ = 19.2, 9.6, 4.9 Hz, 1H), 2.18 (ddt, $J$ = 13.9, 8.6, 5.1 Hz, 1H), 1.72 (d, $J$ = 6.8 Hz, 3H) ppm; $^{13}C$ {$^1H$} NMR (151 MHz, $CDCl_3$): $\delta$ 164.3, 164.15 (d, $J$ = 249.15 Hz), 128.8 (d, $J$ = 8.45 Hz), 123.8 (d, $J$ = 3.3 Hz), 116.1 (d, $J$ = 21.9 Hz), 58.8, 57.7, 38.6, 20.8 ppm; $^{19}F$ {$^1H$} NMR (565 MHz, $CDCl_3$): $\delta$ -110.09 ppm; IR (KBr): 3327, 3096, 2957, 2910, 2867, 1461, 1369, 1193, 1050, 1022, 976, 843, 719, 648 $cm^{-1}$; HRMS (ESI-TOF): *m/z* calcd. for $C_{11}H_{14}FN_4O$ $[M + H]^+$ 237.1146; found 237.1159.

**3-(5-(4-Bromophenyl)-2*H*-tetrazol-2-yl)butan-1-ol** (**35i**)

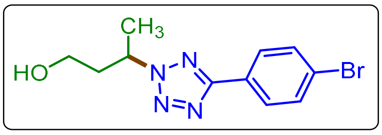


The title compound was obtained according to the general procedure, (Method C). Gummy (100 mg, 67% yield); $R_f$ = 0.29 (3:7 EtOAc:hexane, silica gel; $^1H$ NMR (600 MHz, $CDCl_3$): $\delta$ 8.00 (d, $J$ = 8.5 Hz, 2H), 7.61 (d, $J$ = 8.5 Hz, 2H), 5.31–5.23 (m, 1H), 3.68 (dt, $J$ = 10.9, 5.3 Hz, 1H), 3.47 (ddd, $J$ = 11.2, 8.5, 4.5 Hz, 1H), 2.32 (ddd, $J$ = 19.3, 9.6, 4.9 Hz, 1H), 2.17 (ddt, $J$ = 14.0, 8.6, 5.1 Hz, 1H), 1.70 (d, $J$ = 6.8 Hz, 3H) ppm; $^{13}C$ {$^1H$} NMR (151 MHz, $CDCl_3$): $\delta$ 164.2, 132.2, 128.4, 126.5, 124.7, 58.6, 57.8, 38.7, 20.8 ppm; IR (KBr): 3341, 3060, 2969, 2921, 2859, 1460, 1373, 1258, 1140, 1060, 849, 775, 610 $cm^{-1}$; HRMS (ESI-TOF): *m/z* calcd. for $C_{11}H_{14}{}^{79}BrN_4O$ $[M + H]^+$ 297.0346; found 297.0350.

**3-(5-(4-(Trifluoromethyl)phenyl)-2*H*-tetrazol-2-yl)butan-1-ol** (**35j**)

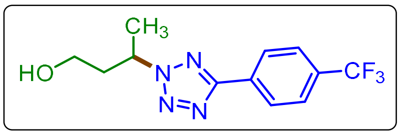


The title compound was obtained according to the general procedure, (Method C). Gummy (101 mg, 70% yield); $R_f$ = 0.27 (3:7 EtOAc:hexane, silica gel); $^1H$ NMR (600 MHz, $CDCl_3$): $\delta$ 8.28 (d, $J$ = 8.1 Hz, 2H), 7.75 (d, $J$ = 8.2 Hz, 2H), 5.36–5.28 (m, 1H), 3.71 (dt, $J$ = 10.9, 5.3 Hz, 1H), 3.50 (ddd, $J$ = 11.1, 8.7, 4.5 Hz, 1H), 2.35 (ddd, $J$ = 19.3, 9.6, 4.9 Hz, 1H), 2.20 (ddt, $J$ = 13.8, 8.5, 5.1 Hz, 1H), 1.74 (d, $J$ = 6.8 Hz, 3H) ppm; $^{13}C$ {$^1H$} NMR (151 MHz, $CDCl_3$): $\delta$ 163.9, 132.1 (d, $J$ = 32.62 Hz), 131.0, 127.6, 127.3, 126.3 (q, $J$ = 3.9 Hz), 126.0 (q, $J$ = 3.8 Hz), 124.0 (q, $J$ = 272.40 Hz), 58.8, 58.0, 38.7, 20.8 ppm; $^{19}F$ NMR (377

MHz, $CDCl_3$): $\delta$ -63.58 ppm; IR (KBr): 3345, 3086, 2960, 2918, 2853, 1466, 1327, 1161, 1068, 943, 856, 760 $cm^{-1}$; HRMS (ESI-TOF): *m/z* calcd. for $C_{12}H_{14}F_3N_4O$ $[M + H]^+$ 287.1114; found 287.1121.

**3-(5-(4-Nitrophenyl)-2*H*-tetrazol-2-yl)butan-1-ol** (**35k**)

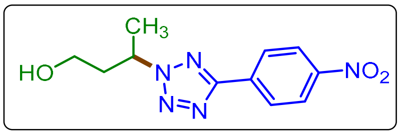


The title compound was obtained according to the general procedure, (Method C). Gummy (97 mg, 74% yield); $R_f$ = 0.20 (3:7 EtOAc:hexane, silica gel; $^1H$ NMR (400 MHz, $CDCl_3$): $\delta$ 8.35 (s, 4H), 5.40–5.28 (m, 1H), 3.73 (dt, *J* = 10.9, 5.4 Hz, 1H), 3.53 (ddd, *J* = 11.1, 8.3, 4.6 Hz, 1H), 2.42–2.32 (m, 1H), 2.25–2.17 (m, 1H), 1.75 (d, *J* = 6.8 Hz, 3H) ppm; $^{13}C$ $\{^1H\}$ NMR (101 MHz, $CDCl_3$): $\delta$ 163.2, 149.0, 133.6, 127.8, 124.4, 58.8, 58.3, 38.8, 20.8 ppm; IR (KBr): 3339, 3095, 3044, 2925, 2861, 1617, 1520, 1467, 1342, 1181, 1040, 910, 863, 706 $cm^{-1}$; HRMS (ESI-TOF): *m/z* calcd. for $C_{11}H_{14}N_5O_3$ $[M + H]^+$ 264.1091; found 264.1096.

**3-(5-(2-Bromophenyl)-2*H*-tetrazol-2-yl)butan-1-ol** (**35m**)

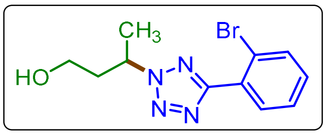


The title compound was obtained according to the general procedure, (Method C). Gummy (96 mg, 64% yield); $R_f$ = 0.26 (3:7 EtOAc:hexane, silica gel); $^1H$ NMR (600 MHz, $CDCl_3$): $\delta$ 7.80 (d, *J* = 7.7 Hz, 1H), 7.69 (d, *J* = 8.0 Hz, 1H), 7.39 (t, *J* = 7.5 Hz, 1H), 7.29 (t, *J* = 7.7 Hz, 1H), 5.31–5.25 (m, 1H), 3.66 (dt, *J* = 10.8, 5.2 Hz, 1H), 3.49–3.42 (m, 1H), 2.29 (ddt, *J* = 14.3, 9.5, 4.9 Hz, 1H), 2.15 (ddt, *J* = 14.0, 8.8, 5.1 Hz, 1H), 1.82 (s, 1H, OH-peak), 1.70 (d, *J* = 6.8 Hz, 3H) ppm; $^{13}C$ $\{^1H\}$ NMR (151 MHz, $CDCl_3$): $\delta$ 163.9, 134.1, 131.7, 131.4, 128.8, 127. 6, 122.2, 58.6, 57.9, 38.9, 20.7 ppm; IR (KBr): 3340, 3325, 3064, 2961, 2933, 1619, 1462, 1375, 1171, 1065, 989, 784 $cm^{-1}$; HRMS (ESI-TOF): *m/z* calcd. for $C_{11}H_{14}{}^{79}BrN_4O$ $[M + H]^+$ 297.0346; found 297.0354.

**(*E*)-3-(5-Styryl-2*H*-tetrazol-2-yl)butan-1-ol** (**35n**)

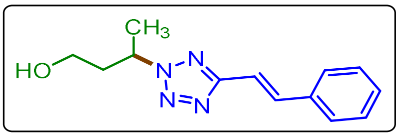


The title compound was obtained according to the general procedure, (Method C). Gummy (62 mg, 51% yield); $R_f$ = 0.30 (3:7 EtOAc:hexane, silica gel); $^1H$ NMR (600 MHz, $CDCl_3$): $\delta$ 7.70 (d, *J* = 16.5 Hz, 1H), 7.53 (d, *J* = 7.4 Hz, 2H), 7.36 (t, *J* = 7.5 Hz, 2H), 7.31 (t, *J* = 7.3 Hz, 1H), 7.12 (d, *J* = 16.5 Hz, 1H), 5.26–5.18 (m, 1H), 3.65 (dt, *J* = 10.6, 5.1 Hz, 1H), 3.43 (ddd, *J* = 11.4, 8.6, 4.4 Hz, 1H), 2.27 (ddt, *J* = 14.3, 9.5, 4.9 Hz, 1H), 2.13 (ddt, *J* = 14.1, 10.0, 5.1 Hz, 1H), 1.79 (s, 1H, OH-peak), 1.70 (d, *J* = 6.8 Hz, 3H) ppm; $^{13}C$ $\{^1H\}$ NMR (151 MHz, $CDCl_3$): $\delta$ 164.2, 136.4, 135.9, 129.2, 129.0, 127.3, 113.7, 58.8, 57.6, 38.8, 20.7 ppm;

IR (KBr): 3359, 3060, 2964, 2930, 2861, 2221, 1627, 1490, 1445, 1200, 1180, 955, 883, 753, 684 $cm^{-1}$; HRMS (ESI-TOF): *m/z* calcd. for $C_{13}H_{17}N_4O$ $[M + H]^+$ 245.1397; found 245.1403.

**3-Phenyl-3-(5-phenyl-2*H*-tetrazol-2-yl)propan-1-ol** (**36a**)

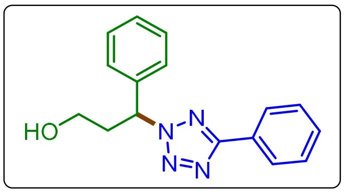


The title compound was obtained according to the general procedure, (Method C). Gummy (119 mg, 85% yield); $R_f = 0.33$ (3:7 EtOAc:hexane, silica gel); $^1H$ NMR (600 MHz, $CDCl_3$): $\delta$ 8.12 (dd, $J$ = 5.9, 1. 7 Hz, 2H), 7.47 (dd, $J$ = 9.8, 2.4 Hz, 2H), 7.46–7.39 (m, 3H), 7.34 (t, $J$ = 7.3 Hz, 2H), 7.31 (t, $J$ = 7.1 Hz, 1H), 6.24 (dd, $J$ = 9.3, 6.3 Hz, 1H), 3.65 (dt, $J$ = 11.1, 5.5 Hz, 1H), 3.61–3.49 (m, 1H), 2.85 (ddd, $J$ = 14.5, 9.9, 5.2 Hz, 1H), 2.60–2.52 (m, 1H) ppm; $^{13}C$ $\{^1H\}$ NMR (151 MHz, $CDCl_3$): $\delta$ 165.2, 137.6, 130.4, 129.1, 128.98, 127.6, 127.4, 127.0, 65.0, 58.8, 37.9 ppm; IR (KBr): 3340, 3025, 2938, 2921, 1941, 1600, 1455, 1167, 1066, 1045, 980, 744, 699 $cm^{-1}$; HRMS (ESI-TOF): *m/z* calcd. for $C_{16}H_{17}N_4O$ $[M + H]^+$ 281.1397; found 281.1410.

**3-(5-(4-Methoxyphenyl)-2*H*-tetrazol-2-yl)-3-phenylpropan-1-ol** (**36e**)

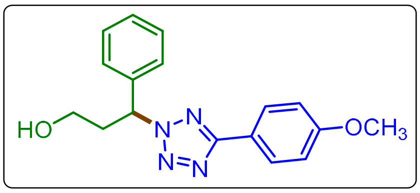


The title compound was obtained according to the general procedure, (Method C). Gummy (118 mg, 76% yield); $R_f$ = 0.31 (3:7 EtOAc:hexane, silica gel); $^1H$ NMR (400 MHz, $CDCl_3$): $\delta$ 8.08 (d, $J$ = 8.9 Hz, 2H), 7.49 (d, $J$ = 6.7 Hz, 2H), 7.40–7.32 (m, 3H), 6.98 (d, $J$ = 8.9 Hz, 2H), 6.24 (dd, $J$ = 9.2, 6.3 Hz, 1H), 3.86 (s, 3H), 3.68 (dt, $J$ = 11.1, 5.5 Hz, 1H), 3.62–3.53 (m, 1H), 2.87 (ddd, $J$ = 14.3, 10.4, 5.2 Hz, 1H), 2.57 (dt, $J$ = 14.1, 6.2 Hz, 1H) ppm; $^{13}C$ $\{^1H\}$ NMR (151 MHz, $CDCl_3$): $\delta$ 165.1, 161.3, 137.7, 129.1, 128.9, 128.5, 127.4, 120.2, 114.4, 64.8, 58.8, 55.5, 37.9 ppm; IR (KBr): 3354, 3021, 2930, 2917, 2855, 1936, 1601, 1495, 1245, 1161, 1057, 1041, 983, 832, 741, 690 $cm^{-1}$; HRMS (ESI-TOF): *m/z* calcd. for $C_{17}H_{19}N_4O_2$ $[M + H]^+$ 311.1503; found 311.1512.

**4-Phenyl-4-(5-phenyl-2*H*-tetrazol-2-yl)butan-1-ol** (**37a**)

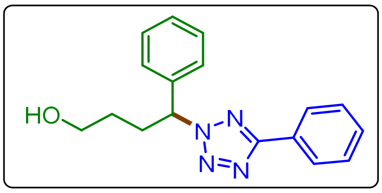


The title compound was obtained according to the general procedure, (Method C). Gummy (128 mg, 87% yield); $R_f$ = 0.41 (3:7 EtOAc:hexane, silica gel); $^1H$ NMR (600 MHz, $CDCl_3$): $\delta$ 8.14 (dd, $J$ = 7.9, 1.5 Hz, 2H), 7.43–7.49 (m, 5H), 7.36 (t, $J$ = 7.3 Hz, 2H), 7.32 (t, $J$ = 7.2 Hz, 1H), 5.97 (dd, $J$ = 9.1, 6.6 Hz, 1H), 3.74–3.65 (m, 2H), 2.76–2.67 (m, 1H), 2.53–2.44 (m, 1H), 1.61–1.56 (m, 1H), 1.54–1.49 (m, 1H) ppm;

$^{13}C\{^1H\}$ NMR (151 MHz, $CDCl_3$): $\delta$ 165.2, 137.8, 130.4, 129.1, 128.96, 128.93, 127.6, 127.3, 127.0, 68.5, 62.1, 32.0, 29.4 ppm; IR (KBr): 3348, 3114, 2940, 2924, 2864, 1944, 1600, 1466, 1165, 1055, 983, 745, 695 cm$^{-1}$; HRMS (ESI-TOF): *m/z* calcd. for $C_{17}H_{19}N_4O$ [M + H]$^+$ 295.1553; found 295.1561.

**3-(5-Phenyl-2*H*-tetrazol-2-yl)hexan-1-ol (38a)**[11]

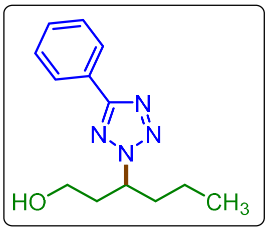


The title compound was obtained according to the general procedure, (Method C). Gummy (22 mg, 18% yield); $R_f$ = 0.28 (3:7 EtOAc:hexane, silica gel); $^1H$ NMR (600 MHz, $CDCl_3$): $\delta$ 8.17–8.09 (m, 2H), 7.48–7.42 (m, 3H), 5.13 (tt, *J* = 9.6, 4.4 Hz, 1H), 3.62 (dt, *J* = 10.5, 5.1 Hz, 1H), 3.35 (ddd, *J* = 11.1, 9.2, 4.5 Hz, 1H), 2.28 (ddt, *J* = 14.7, 9.4, 4.5 Hz, 1H), 2.20–2.15 (m, 1H), 2.13 (dt, *J* = 14.3, 4.7 Hz, 1H), 1.88 (ddd, *J* = 20.3, 10.5, 5.5 Hz, 1H), 1.27–1.23 (m, 1H), 1.16–1.10 (m, 1H), 0.88 (t, *J* = 7.4 Hz, 3H) ppm; $^{13}C\{^1H\}$ NMR (151 MHz, $CDCl_3$): $\delta$ 165.1, 130.4, 129.0, 127.7, 127.0, 61.9, 58.8, 37.5, 36.95, 19.2, 13.6 ppm; IR (KBr): 3406, 3333, 3067, 2960, 2927, 2865, 1531, 1462, 1363, 1190, 1059, 920, 722, 691 cm$^{-1}$; HRMS (ESI-TOF): *m/z* calcd. for $C_{13}H_{19}N_4O$ [M + H]$^+$ 247.1553; found 247.1559.

**4-(5-Phenyl-2*H*-tetrazol-2-yl)hexan-1-ol (38'a)**

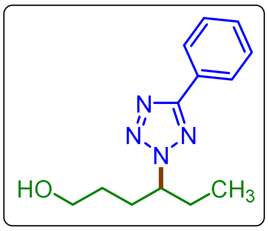


The title compound was obtained according to the general procedure, (Method C). Gummy (28 mg, 23% yield); $R_f$ = 0.26 (3:7 EtOAc:hexane, silica gel); $^1H$ NMR (600 MHz, $CDCl_3$): $\delta$ 8.15–8.11 (m, 2H), 7.48–7.43 (m, 3H), 4.78 (tt, *J* = 9.5, 4.7 Hz, 1H), 3.65–3.55 (m, 2H), 2.16 (ddd, *J* = 19.7, 10.0, 5.3 Hz, 1H), 2.12–2.07 (m, 1H), 2.07–2.00 (m, 1H), 1.97 (ddd, *J* = 10.4, 7.3, 3.6 Hz, 1H), 1.52–1.45 (m, 1H), 1.36–1.30 (m, 1H), 0.81 (t, *J* = 7.4 Hz, 3H) ppm; $^{13}C\{^1H\}$ NMR (151 MHz, $CDCl_3$): $\delta$ 165.1, 130.4, 129.0, 127.8, 127.0, 67.0, 62.1, 31.1, 29.0, 28.4, 10.6 ppm; IR (KBr): 3411, 3070, 2955, 2930, 2870, 1538, 1462, 1379, 1264, 1190, 1060, 930, 729 cm$^{-1}$; HRMS (ESI-TOF): *m/z* calcd. for $C_{13}H_{19}N_4O$ [M + H]$^+$ 247.1553; found 247.1564.

**5-(5-Phenyl-2*H*-tetrazol-2-yl)hexan-1-ol (38''a)**

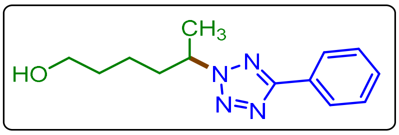


The title compound was obtained according to the general procedure, (Method C). Gummy (49 mg, 40% yield); $R_f$ = 0.25 (3:7 EtOAc:hexane, silica gel); $^1H$ NMR (600 MHz,

$CDCl_3$): $\delta$ 8.12 (dd, $J$ = 7.9, 1.4 Hz, 2H), 7.47–7.42 (m, 3H), 5.00–4.93 (m, 1H), 3.58 (t, $J$ = 6.4 Hz, 2H), 2.18–2.09 (m, 1H), 1.92 (ddt, $J$ = 14.0, 10.9, 5.6 Hz, 1H), 1.65 (d, $J$ = 6.8 Hz, 3H), 1.63 (s, 1H, OH-peak), 1.61–1.47 (m, 3H), 1.39–1.31 (m, 1H) ppm; $^{13}C\ \{^1H\}$ NMR (151 MHz, $CDCl_3$): $\delta$ 165.0, 130.3, 129.0, 127.8, 126.9, 62.6, 60.9, 36.2, 32.1, 22.3, 20.8 ppm; IR (KBr): 3409, 3070, 2950, 2933, 2924, 2864, 1460, 1357, 1179, 1055, 910, 724, cm$^{-1}$; HRMS (ESI-TOF): $m/z$ calcd. for $C_{13}H_{19}N_4O$ $[M + H]^+$ 247.1553; found 247.1561.

**3-(4-(4-(*tert*-Butyl)phenyl)-2*H*-1,2,3-triazol-2-yl)butan-1-ol (35s)**

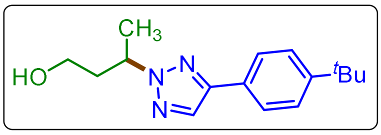


The title compound was obtained according to the general procedure, (Method C). Gummy (77 mg, 56% yield); $R_f$ = 0.38 (3:7 EtOAc:hexane, silica gel); $^1H$ NMR (600 MHz, $CDCl_3$): $\delta$ 7.78 (s, 1H), 7.67 (d, $J$ = 8.3 Hz, 2H), 7.42 (d, $J$ = 8.3 Hz, 2H), 5.01–4.90 (m, 1H), 3.58 (dt, $J$ = 10.6, 5.0 Hz, 1H), 3.36 (td, $J$ = 11.5, 10.3, 4.4 Hz, 1H), 2.17 (ddt, $J$ = 14.3, 9.4, 4.5 Hz, 1H), 2.08 (ddt, $J$ = 14.2, 9.5, 5.0 Hz, 1H), 1.62 (d, $J$ = 6.8 Hz, 3H), 1.31 (s, 9H) ppm; $^{13}C\ \{^1H\}$ NMR (151 MHz, $CDCl_3$): $\delta$ 151.7, 147.4, 130.5, 127.7, 125.9, 125.8, 59.1, 58.5, 39.3, 34.8, 31.4, 20.7 ppm; IR (KBr): 3401, 3168, 2973, 2901, 2865, 1561, 1483, 1369, 1311, 1273, 1128, 1041, 845, 730 cm$^{-1}$; HRMS (ESI-TOF): $m/z$ calcd. for $C_{16}H_{24}N_3O$ $[M + H]^+$ 274.1914; found 274.1919.

**3-(4-(4-Chlorophenyl)-2*H*-1,2,3-triazol-2-yl)butan-1-ol (35t)**

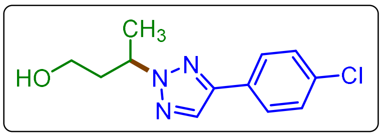


The title compound was obtained according to the general procedure, (Method C). Gummy (77 mg, 61% yield); $R_f$ = 0.31 (3:7 EtOAc:hexane, silica gel); $^1H$ NMR (600 MHz, $CDCl_3$): $\delta$ 7.81 (s, 1H), 7.71 (d, $J$ = 8.4 Hz, 2H), 7.39 (d, $J$ = 8.4 Hz, 2H), 5.02–4.96 (m, 1H), 3.66–3.60 (m, 1H), 3.44–3.38 (m, 1H), 2.22 (ddt, $J$ = 14.3, 9.5, 4.6 Hz, 1H), 2.11 (ddt, $J$ = 14.2, 9.6, 4.9 Hz, 2H) (OH peak merged), 1.65 (d, $J$ = 6.8 Hz, 3H) ppm; $^{13}C\ \{^1H\}$ NMR (151 MHz, $CDCl_3$): $\delta$ 146.4, 134.3, 130.6, 129.2, 129.1, 127.3, 59.2, 58.7, 39.3, 20.8 ppm; IR (KBr): 3392, 3130, 2980, 2936, 1610, 1535, 1477, 1385, 1246, 1088, 1053, 990, 834 cm$^{-1}$; HRMS (ESI-TOF): $m/z$ calcd. for $C_{12}H_{15}{}^{35}ClN_3O$ $[M + H]^+$ 252.0898; found 252.0907.

**2-Cyclohexyl-5-phenyl-2*H*-tetrazole (40a)[12]**

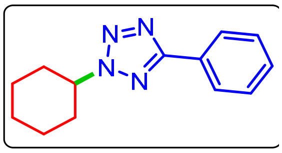


The title compound was obtained according to the general procedure, (Method D). White solid (87 mg, 76% yield); $R_f$ = 0.48 (0.5:9.5 EtOAc:hexane, silica gel); $^1H$ NMR (600 MHz, $CDCl_3$): $\delta$ 8.15 (dd, $J$ = 8.0, 1.4 Hz, 2H), 7.53–7.44 (m, 3H), 4.75 (tt, $J$ = 11.5, 3.9 Hz,

1H), 2.33–2.22 (m, 2H), 2.09–1.92 (m, 4H), 1.79–1.73 (m, 1H), 1.54–1.34 (m, 3H) ppm; $^{13}C\{^1H\}$ NMR (151 MHz, $CDCl_3$): $\delta$ 164.7, 130.2, 128.9, 127.8, 126.9, 63.3, 32.6, 25.1, 25.0 ppm; IR (KBr): 2933, 2858, 1526, 1451, 1343, 1272, 1190, 1002, 895, 823, 733, 694, 510 $cm^{-1}$; HRMS (ESI-TOF) *m/z:* $[M + H]^+$ calcd for $C_{13}H_{17}N_4$, 229.1448; found, 229.1433.

**5-(4-Bromophenyl)-2-cyclohexyl-2*H*-tetrazole (40i)**

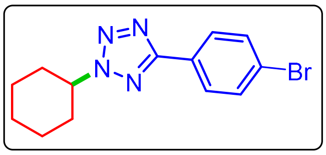


The title compound was obtained according to the general procedure, (Method D). White solid (129 mg, 84% yield); $R_f$ = 0.51 (0.5:9.5 EtOAc:hexane, silica gel); $^1H$ NMR (600 MHz, $CDCl_3$) $\delta$ 8.02 (d, $J$ = 8.6 Hz, 2H), 7.62 (d, $J$ = 8.6 Hz, 2H), 4.74 (tt, $J$ = 11.5, 3.9 Hz, 1H), 2.27 (dd, $J$ = 12.5, 3.3 Hz, 2H), 2.05–1.92 (m, 4H), 1.79–1.75 (m, 1H), 1.53–1.45 (m, 2H), 1.39–1.32 (m, 1H) ppm; $^{13}C\{^1H\}$ NMR (151 MHz, $CDCl_3$) $\delta$ 163.9, 132.2, 128.4, 126.8, 124.6, 63.5, 32.6, 25.1, 25.0 ppm; IR (KBr): 2932, 2858, 1731, 1603, 1453, 1268, 1004, 836, 755, 508 $cm^{-1}$; HRMS (ESI-TOF): *m/z* calcd. for $C_{13}H_{16}{}^{79}BrN_4$ $[M + H]^+$ 307.0553; found 307.0560.

**5-(2-Bromophenyl)-2-cyclohexyl-2*H*-tetrazole (40m)**

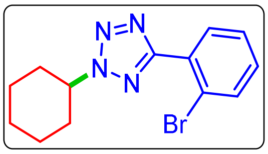


The title compound was obtained according to the general procedure, (Method D). White solid (125 mg, 81% yield); $R_f$ = 0.50 (0.5:9.5 EtOAc:hexane, silica gel); $^1H$ NMR (600 MHz, $CDCl_3$): $\delta$ 7.85 (dd, $J$ = 7.7, 1.6 Hz, 1H), 7.76–7.70 (m, 1H), 7.42 (td, $J$ = 7.6, 1.0 Hz, 1H), 7.32 (td, $J$ = 7.8, 1.7 Hz, 1H), 4.79 (tt, $J$ = 11.4, 3.9 Hz, 1H), 2.34–2.28 (m, 2H), 2.04 (qd, $J$ = 12.5, 3.6 Hz, 2H), 1.96 (dt, $J$ = 13.7, 3.4 Hz, 2H), 1.77 (dt, $J$ = 13.1, 3.5 Hz, 1H), 1.50 (qt, $J$ = 13.4, 3.4 Hz, 2H), 1.37 (qt, $J$ = 13.2, 3.2 Hz, 1H) ppm; $^{13}C\{^1H\}$ NMR (151 MHz, $CDCl_3$): $\delta$ 163.7, 134.2, 131.8, 131.2, 129.0, 127.5, 122.2, 63.5, 32.6, 25.1, 24.9 ppm; IR (KBr): 2931, 2858, 1725, 1599, 1565, 1518, 1446, 1349, 1265, 1182, 1142, 1030, 817, 748, 655, 455 $cm^{-1}$; HRMS (ESI-TOF): *m/z* calcd. for $C_{13}H_{16}{}^{79}BrN_4$ $[M + H]^+$ 307.0553; found 307.0562.

**2-Cyclohexyl-5-(thiophen-2-yl)-2*H*-tetrazole (40q)**

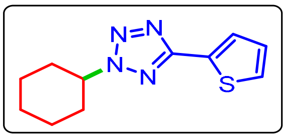


The title compound was obtained according to the general procedure, (Method D). White solid (85 mg, 72% yield); $R_f$ = 0.41 (0.5:9.5 EtOAc:hexane, silica gel; $^1H$ NMR (600 MHz, $CDCl_3$): $\delta$ 7.79 (dd, $J$ = 3.6, 1.0 Hz, 1H), 7.43 (dd, $J$ = 5.0, 1.0 Hz, 1H), 7.14 (dd, $J$ = 4.9, 3.7 Hz, 1H), 4.72 (tt, $J$ = 11.5, 3.9 Hz, 1H), 2.29–2.23 (m, 2H), 2.05–1.92 (m, 4H), 1.78–1.72 (m, 1H), 1.48 (qt, $J$ = 13.3, 3.3 Hz, 2H), 1.36 (tt, $J$ = 12.5, 3.5 Hz, 1H) ppm; $^{13}C$

{$^1$H} NMR (151 MHz, $CDCl_3$): $\delta$ 160.8, 129.6, 128.0, 127.7, 127.6, 63.5, 326, 25.1, 24.9 ppm; IR (KBr): 2932, 2858, 1729, 1571, 1475, 1452, 1390, 1224, 1187, 1040, 1000, 968, 849, 754, 706, 500 cm$^{-1}$; HRMS (ESI-TOF) *m/z:* $[M + H]^+$ calcd for $C_{11}H_{15}N_4S$, 235.1012; found, 235.1000.

**(8S,13S,14S)-3-((*tert*-Butyldimethylsilyl)oxy)-13-methyl-9-(5-phenyl-2*H*-tetrazol-2-yl)-7,8,9,11,12,13,15,16-octahydro-6*H*-cyclopenta[*a*]phenanthren-17(14*H*)-one (41a)**

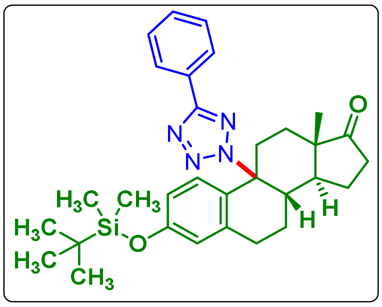

The title compound was obtained according to the general procedure, (Method B). Gummy (82 mg, 62% yield); $R_f$ = 0.51 (1:9 EtOAc:hexane, silica gel); $^1$H NMR (600 MHz, $CDCl_3$): $\delta$ 8.10 (dd, $J$ = 7.7, 1.5 Hz, 2H), 7.54 (d, $J$ = 8.7 Hz, 1H), 7.47–7.43 (m, 3H), 6.78 (dd, $J$ = 8.6, 2.4 Hz, 1H), 6.66 (d, $J$ = 2.3 Hz, 1H), 3.15 (dd, $J$ = 8.8, 2.8 Hz, 1H), 2.88–2.70 (m, 4H), 2.54 (dd, $J$ = 19.3, 8.1 Hz, 1H), 2.16–2.09 (m, 1H), 2.05–1.99 (m, 1H), 1.84–1.73 (m, 4H), 1.64–1.58 (m, 1H), 1.38–1.32 (m, 1H), 1.20 (s, 3H), 0.99 (s, 9H), 0.24 (s, 6H) ppm; $^{13}$C {$^1$H} NMR (151 MHz, $CDCl_3$): $\delta$ 218.9, 164.3, 156.0, 139.3, 130.2, 130.1, 128.9, 127.9, 126.9, 123.7, 120.6, 118.5, 70.98 (disappear in DEPT-45 ), 47.6, 43.1, 39.2, 35.8, 32.7, 28.2, 25.7, 25.0, 22.1, 19.3, 18.3, 13.8, -4.16, -4.18 ppm; IR (KBr): 3054, 3019, 2953, 2910, 2861, 1729, 1594, 1501, 1483, 1399, 1284, 1131, 1036, 928, 832, 725, 689, 596 cm$^{-1}$; HRMS (ESI-TOF) *m/z:* $[M + H]^+$ calcd for $C_{31}H_{41}N_4O_2Si$, 529.2993; found, 529.3017.

**3-(5-Phenyl-2H-tetrazol-2-yl)butyl (2S,5R)-3,3-dimethyl-7-oxo-4-thia-1-azabicyclo[3.2.0]heptane-2-carboxylate 4,4-dioxide (42a)**

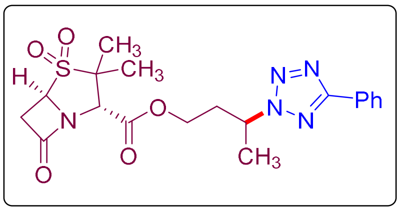

The title compound was obtained according to the general procedure, (Method B). The title compound was isolated as mixture of two stereoisomers determined by $^1$H and $^{13}$C NMR. Gummy (220 mg, 51% yield); $R_f$ = 0.23 (3:7 EtOAc:hexane, silica gel); $^1$H NMR (400 MHz, $CDCl_3$): $\delta$ 8.13–8.06 (m, 2H), 7.48–7.39 (m, 3H), 5.17–5.05 (m, 1H), 4.61–4.46 (m, 1H), 4.28/4.27 (2s, 1H), 4.27–3.83 (m, 2H), 3.32–3.18 (m, 2H), 2.52–2.43 (m, 1H), 2.34–2.21 (m, 1H), 1.70 (dd, $J$ = 6.8, 3.9 Hz, 3H), 1.57/1.51 (2s, 3H), 1.33 (s, 3H) ppm; $^{13}$C {$^1$H} NMR (101 MHz, $CDCl_3$): $\delta$ 170.96, 170.85, 166.85, 166.81, 165.2, 165.1, 130.5, 129.1, 127.3, 126.9, 63.2, 62.8, 62.6, 61.14, 61.08, 57.8, 38.28, 38.2, 34.8, 20.9, 20.7, 20.26, 20.20, 18.6, 18.5 ppm; IR

(KBr): 3010, 2943, 2856, 1791, 1758, 1533, 1464, 1458, 1342, 1277, 1262, 1089, 954, 751 $cm^{-1}$; HRMS (ESI-TOF) *m/z:* $[M + H]^+$ calcd for $C_{19}H_{24}N_5O_5S$, 434.1493; found, 434.1506.

**3-(5-(4-((*tert*-Butylperoxy)carbonyl)phenyl)-2*H*-tetrazol-2-yl)butyl acetate (1za')**

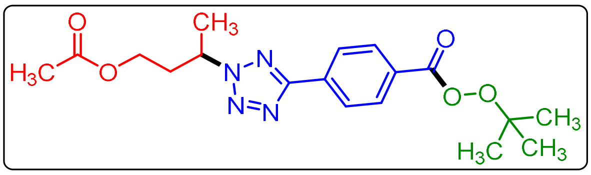


The title compound was obtained according to the general procedure, (Method A). Gummy (115 mg, 61% yield); $R_f$ = 0.31 (2:8 EtOAc:hexane, silica gel); $^1H$ NMR (600 MHz, $CDCl_3$): $\delta$ 8.25 (d, $J$ = 8.4 Hz, 2H), 8.07 (d, $J$ = 8.4 Hz, 2H), 5.24–5.15 (m, 1H), 4.17–4.11 (m, 1H), 4.00–3.95 (m, 1H), 2.54–2.46 (m, 1H), 2.31–2.25 (m, 1H), 1.99 (s, 3H), 1.74 (d, $J$ = 6.8 Hz, 3H), 1.44 (s, 9H) ppm; $^{13}C$ {$^1H$} NMR (151 MHz, $CDCl_3$): $\delta$ 170.9, 164.1, 164.0, 132.2, 129.9, 129.3, 127.1, 84.4, 60.7, 58.4, 35.2, 26.4, 20.9, 20.8 ppm; IR (KBr): 2983, 2932, 2854, 1744, 1617, 1569, 1538, 1463, 1424, 1367, 1234, 1190, 1178, 1056, 1017, 1005, 863, 740, 692, 604 $cm^{-1}$; HRMS (ESI-TOF) *m/z:* $[M + H]^+$ calcd for $C_{18}H_{25}N_4O_5$, 377.1819; found, 377.1822.

**2-Methyl-5-phenyl-2*H*-tetrazole (44a)**[5]

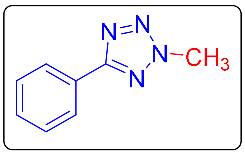


The title compound was obtained according to the general procedure, (Method B). White solid (46 mg, 58% yield); $R_f$ = 0.27 (0.5:9.5 EtOAc:hexane, silica gel); $^1H$ NMR (600 MHz, $CDCl_3$): $\delta$ 8.14 (d, $J$ = 7.8 Hz, 2H), 7.49 (t, $J$ = 7.1 Hz, 3H), 4.41 (s, 3H); $^{13}C${$^1H$} NMR (151 MHz, $CDCl_3$): $\delta$ 165.4, 130.5, 129.1, 127.5, 126.9, 39.6; IR (KBr): 3071, 2956, 2926, 2853, 1718, 1449, 1190, 1048, 923, 792, 694 $cm^{-1}$; HRMS (ESI-TOF) *m/z:* $[M + H]^+$ calcd for $C_8H_9N_4$, 161.1822; found, 161.1824.

**1,1-Diphenyl-2-(5-phenyl-2*H*-tetrazol-2-yl)ethan-1-ol (45a)**[5]

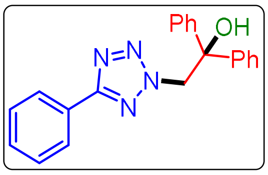


The title compound was obtained according to the general procedure, (Method B). White solid (136 mg, 79% yield); $R_f$ = 0.26 (1:9 EtOAc:hexane, silica gel); $^1H$ NMR (600 MHz, $CDCl_3$): $\delta$ 8.17 (dd, $J$ = 6.7, 3.0 Hz, 2H), 7.49 (dd, $J$ = 5.0, 1.7 Hz, 3H), 7.39–7.34 (m, 6H), 7.20 (dd, $J$ = 6.8, 2.9 Hz, 4H), 4.85 (d, $J$ = 7.5 Hz, 2H), 3.92 (t, $J$ = 7.6 Hz, 1H) (3.90 (t), $D_2O$ exchangeable) ppm; $^{13}C$ {$^1H$} NMR (151 MHz, $CDCl_3$): $\delta$ 164.5, 139.2, 130.7, 129.0, 128.8, 128.6, 128.3, 127.1, 127.0, 78.8, 69.2 ppm; IR (KBr): 3433, 3397, 3059,

3033, 1942, 1600, 1464, 1447, 1409, 1367, 1307, 1193, 1084, 1013, 748, 735, 693 $cm^{-1}$; HRMS (ESI-TOF) *m/z:* $[M + H]^+$ calcd for $C_{21}H_{19}N_4O$, 343.1553; found, 343.1561.

## 9. Computational details

All the structures have been optimized at density functional theory using (U)M06-2X[13] and (U)wB97XD[14] functions with 6-311G(d,p)[15] basis set. Geometries have been optimized to their energy minima that were confirmed by frequency calculations. Unrestricted formalism has been utilized for all open shell (unpaired electron) species during optimization. All these calculations were performed using Gaussian 09 suite of program.[16]

Bond dissociation energies have been estimated for the formation of carbon centered radicals. Reaction involving homolytic cleavage of C–H bonds towards the formation the radical isomers have been considered, and the enthalpy change associated with the reaction has been used for the estimation of the BDEs. We have estimated the first bond dissociation energies of various C–H bonds in several species such that the resulting products are one of the carbons centered radical isomers. Also, we have estimated the H-abstraction energy barriers using the transition state calculations, which have been characterized by single imaginary frequency. In such reactions, $^t$BuO radical has been used for the H-abstraction from the species to form various radical isomers.

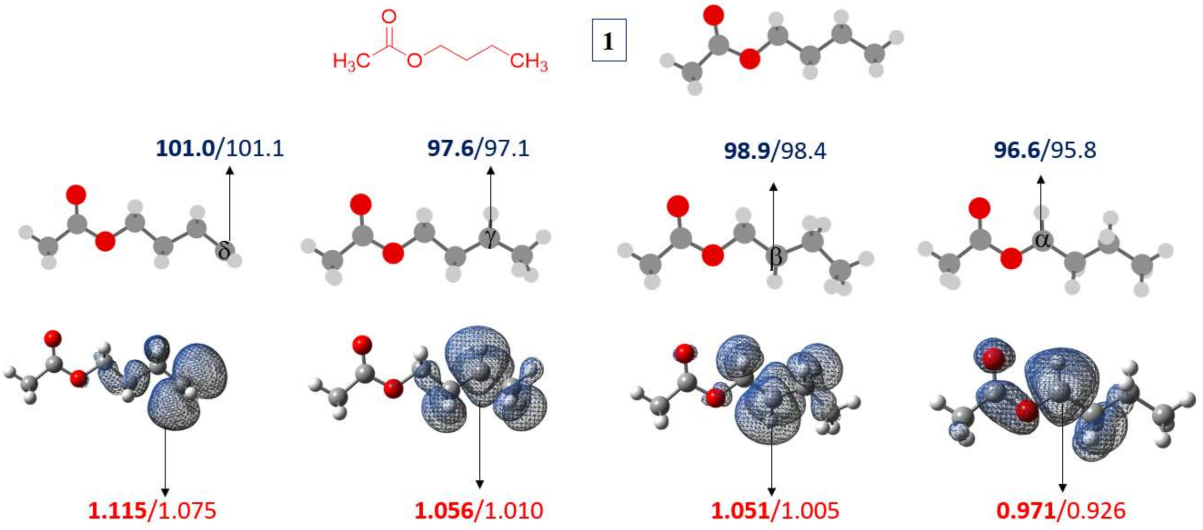


**Figure S5.** Possible isomeric radicals expected of *n*-butyl acetate (**1**) and the corresponding BDEs (in kcal/mol) are mentioned in blue; The spin density plots corresponding to those radical isomers are rendered at isovalue 0.05 and the values are indicated in red. {**Bold** - (U)M06-2X/6-311G(d,p) and Normal font - (U)wB97XD/6-311G(d,p) levels of theory}

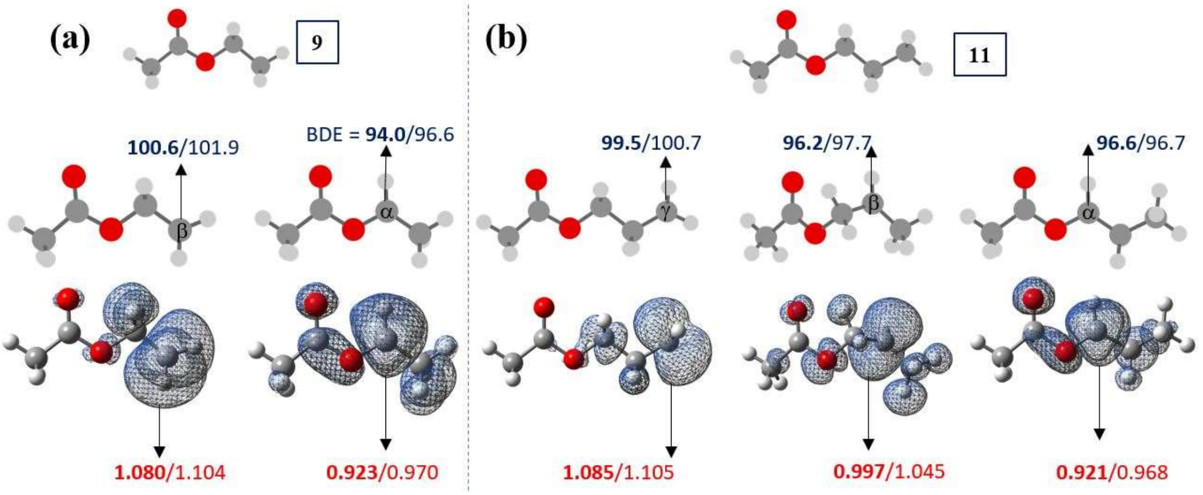


**Figure S6.** Possible isomeric radicals expected of (a) ethyl acetate (**7**), and (b) propyl acetate (**9**). The corresponding BDEs (in kcal/mol) are mentioned in blue; The spin density plots corresponding to those radical isomers are rendered at isovalue 0.05 and the values are indicated in red. {**Bold** - (U)M06-2X/6-311G(d,p) and Normal font - (U)wB97XD/6-311G(d,p) levels of theory}

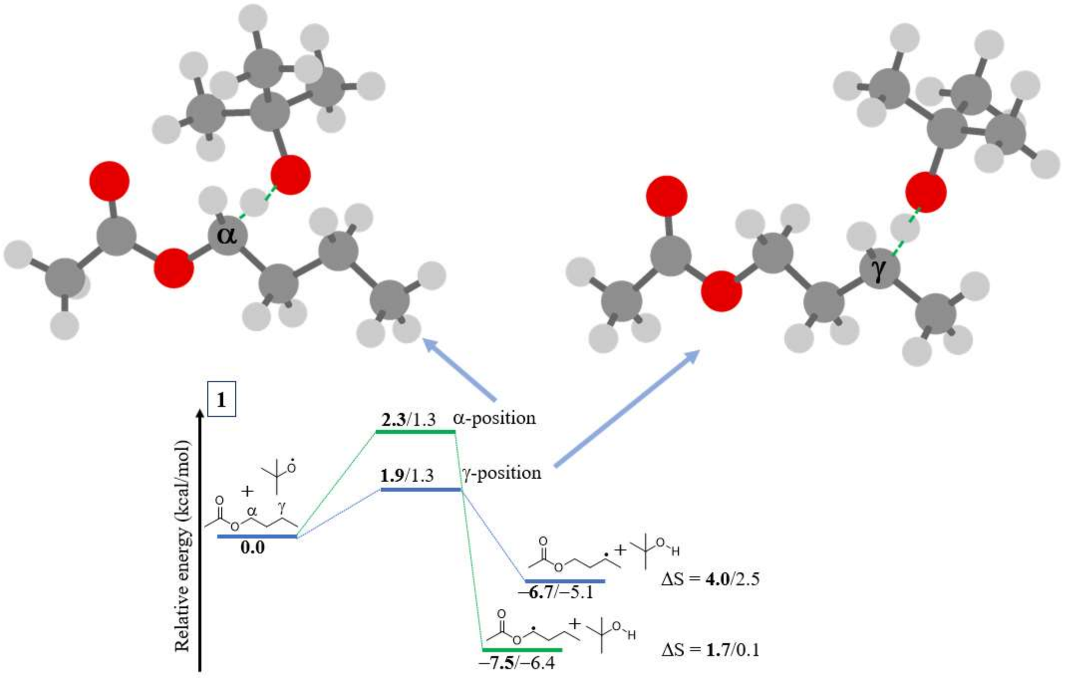


**Figure S7.** Energy profile depicting the kinetic favorability of γ-radical over α-radical formation in **1**; the energies relative to the **1** + $^{t}$BuO radical are indicated (in kcal/mol), the thermodynamic entropy changes accompanying the reactions are indicated (in cal/K-mol); **Bold** - (U)M06-2X/6-311G(d,p) and Normal font - (U)wB97XD/6-311G(d,p) levels of theory)

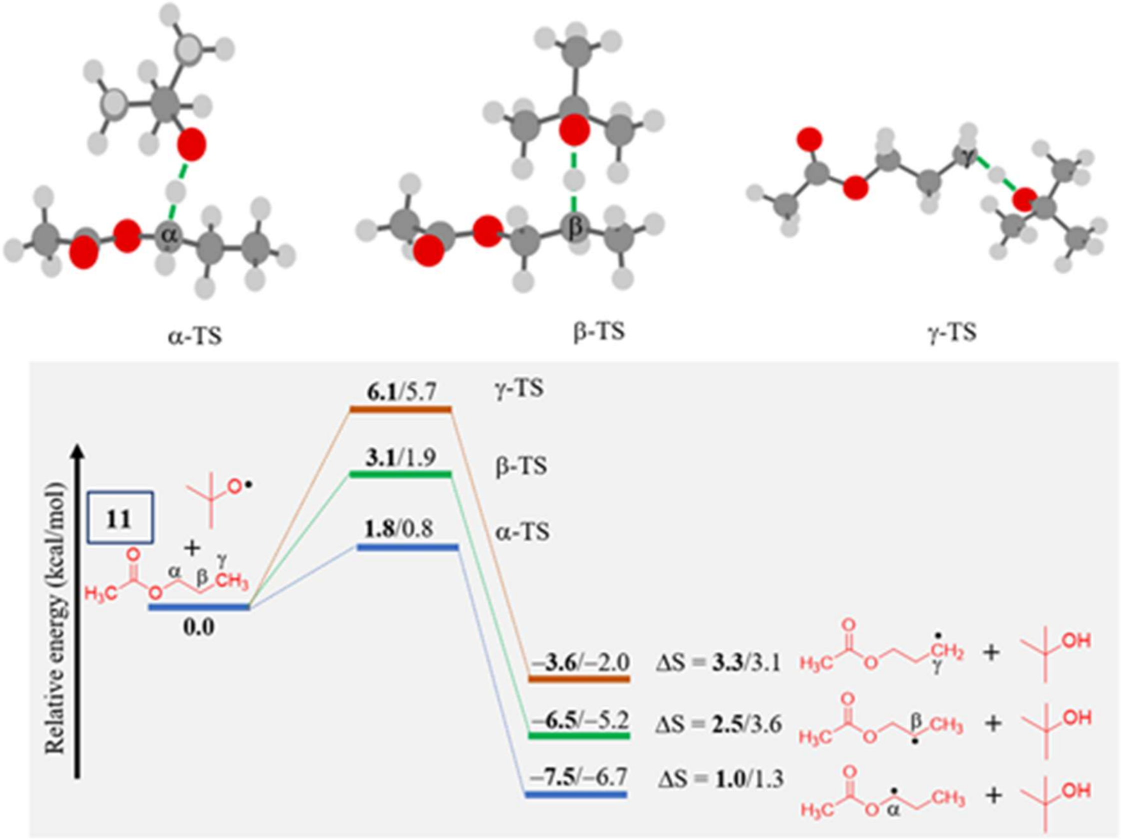


**Figure S8**. Energy profile depicting the kinetic and thermodynamic favorability of radical formations in **11**; the energies relative to the **11** + $^{t}$BuO radical are indicated (in kcal/mol), the thermodynamic entropy changes accompanying the reactions are indicated (in cal/K-mol); **Bold** - (U)M06-2X/6-311G(d,p) and Normal font - (U)wB97XD/6-311G(d,p) levels of theory.

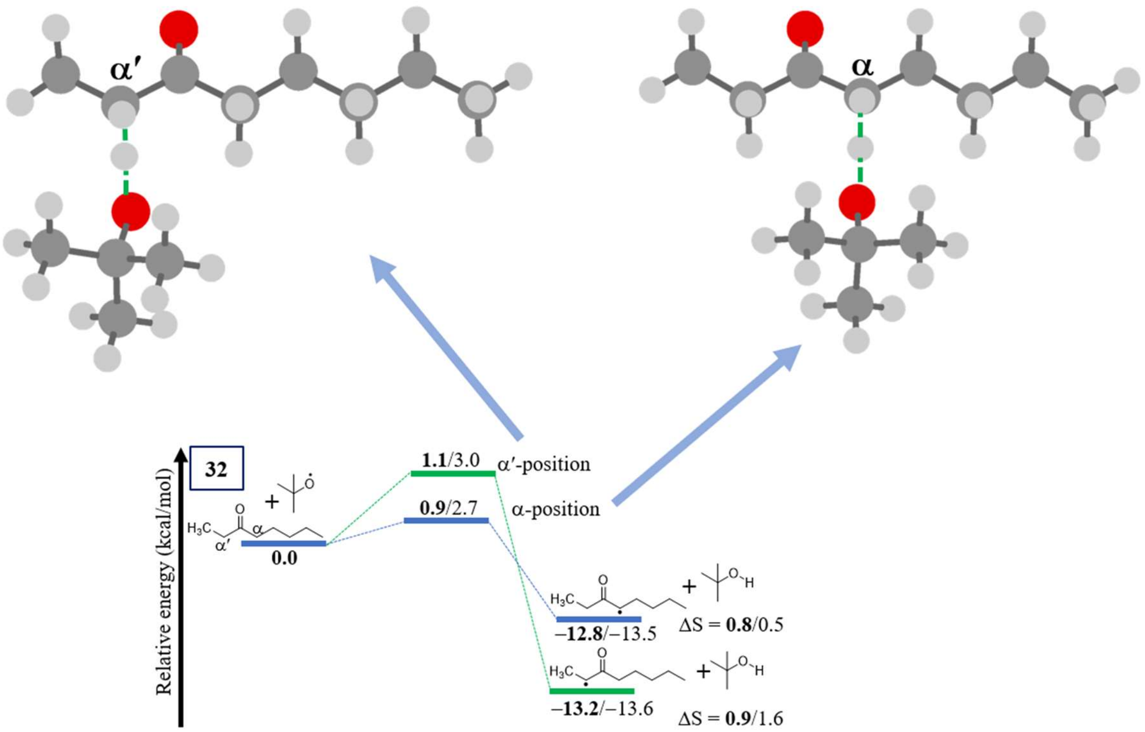


**Figure S9.** Energy profile depicting the formation of the radicals **32a** and **32'a**; the energies relative to the **32** + $^t$BuO radical are indicated (in kcal/mol), the thermodynamic entropy changes accompanying the reactions are indicated (in cal/K-mol); **Bold** - (U)M06-2X/6-311G(d,p) and Normal font - (U)wB97XD/6-311G(d,p) levels of theory.

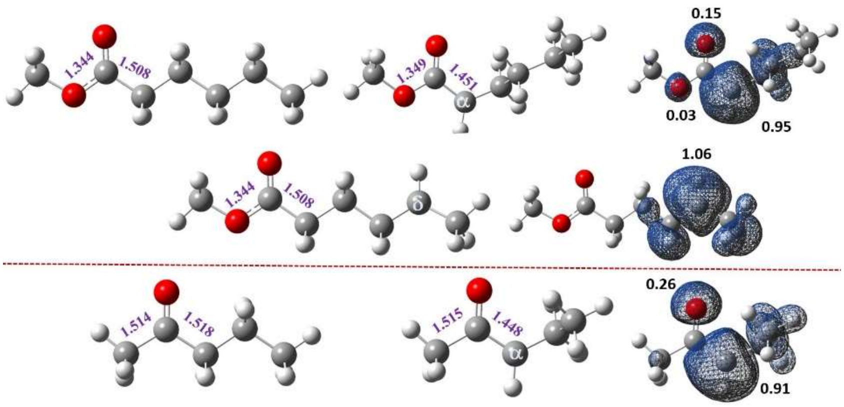


**Figure S10**. Optimized geometries of methyl caproate (**24**) and its α- and δ-radicals, 2-pentanone and its α-radical at (U)M06-2X/6-311G(d,p) level of theory; The spin density plots and important bond distances are depicted.

## 10. References

1. M. Uyanik, D. Suzuki, T. Yasui and K. Ishihara, *Angew. Chem. Int. Ed.* 2011, **50**, 5331.

2. S. Yamada, D. Morizono and K. Yamamoto, *Tetrahedron Lett.* 1992, **33**, 4329.

3. M. Uyanik, H. Okamoto, T. Yasui and K. Ishihara, *Science* 2010, **328**, 1376.

4. W. Wei, C. Zhang, Y. Xu and X. Wan, *Chem. Commun.* 2011, **47**, 10827.

5. S. Rajamanickam, C. Sah, B. A. Mir, S. Ghosh, G. Sethi, V. Yadav, S. Venkataramani and B. K. Patel, *J. Org. Chem.,* 2020, **85**, 2118.

6. S. Kumar, S. Dubey, N. Saxena and S. K. Awasthi, *Tetrahedron Lett*. 2014, **55**, 6034.

7. C. Wang, L. Zhang, A. Ren, P. Lu and Y.Wang, *Org. Lett.* 2013, **15**, 2982.

8. K. Mergemeier and M. Lehr, *Anal. Biochem.* 2018, **549**, 29.

9. Y. Ding, X. Zhang, D. Zhang, Y. Chen, Z. Wu, P. Wang, W. Xue, B. Song and S. Yang, *Tetrahedron Lett.,* 2015, **56**, 831.

10. H. Umemoto, T.; Onaka, Y.; Miki, A.; Nakamura and T. Maegawa, *Synlett* 2015, **26**, 205.

11. U. Uria, J. L. Vicario, D. Badía and L. Carrillo, *Chem. Commun.* 2007, 2509.

12. A. D. Lisakova, D. S. Ryabukhin, R. E. Trifonov, V. A. Ostrovskii and A. V. Vasilyev, *Tetrahedron Lett.* 2015, **56**, 7020.

13. Y. Zhao and D. G. Truhlar, *Theor. Chem. Acc.* 2008, **120**, 215.

14. J.-D. Chai and M. Head-Gordon, *PCCP* 2008, **10**, 6615.

15. R. Krishnan, J. S, Binkley, R. Seeger, and J. A. Pople, *J. Chem. Phys*. 1980, **72**, 650.

16. M. J. Frisch, G. W. Trucks, H. B. Schlegel, G. E. Scuseria, M. A. Robb, J. R. Cheeseman, G. Scalmani, V. Barone, G. A. Petersson, H. Nakatsuji, X. Li, M. Caricato, A. Marenich, J. Bloino, B. G. Janesko, R. Gomperts, B. Mennucci, H. P. Hratchian, J. V. Ortiz, A. F. Izmaylov, J. L. Sonnenberg, D. Williams-Young, F. Ding, F. Lipparini, F. Egidi, J. Goings, B. Peng, A. Petrone, T. Henderson, D. Ranasinghe, V. G. Zakrzewski, J. Gao, N. Rega, G. Zheng, W. Liang, M. Hada, M. Ehara, K. Toyota, Fukuda, J. Hasegawa, M. Ishida, T. Nakajima, Y. Honda, O. Kitao, H. Nakai, T. Vreven, K. Throssell, J. A. Montgomery, J. J. E. Peralta, F. Ogliaro, M. Bearpark, J. J. Heyd, E. Brothers, K. N. Kudin, V. N., Staroverov, T. Keith, R. Kobayashi, J. Normand, K. Raghavachari, A. Rendell, J. C. Burant, S. S. Iyengar, J. Tomasi, M. Cossi, J. M. Millam, M. Klene, C. Adamo, R. Cammi, J. W. Ochterski, R. L. Martin, K. Morokuma, O. Farkas, J. B. Foresman, and D. J. Fox, Gaussian 09, Revision A.02 *Gaussian, Inc., Wallingford CT,* (2016).

# 11. NMR spectra

## 3-(5-Phenyl-2*H*-tetrazol-2-yl)butyl acetate (1a): $^{1}$H NMR (600 MHz, $CDCl_3$)

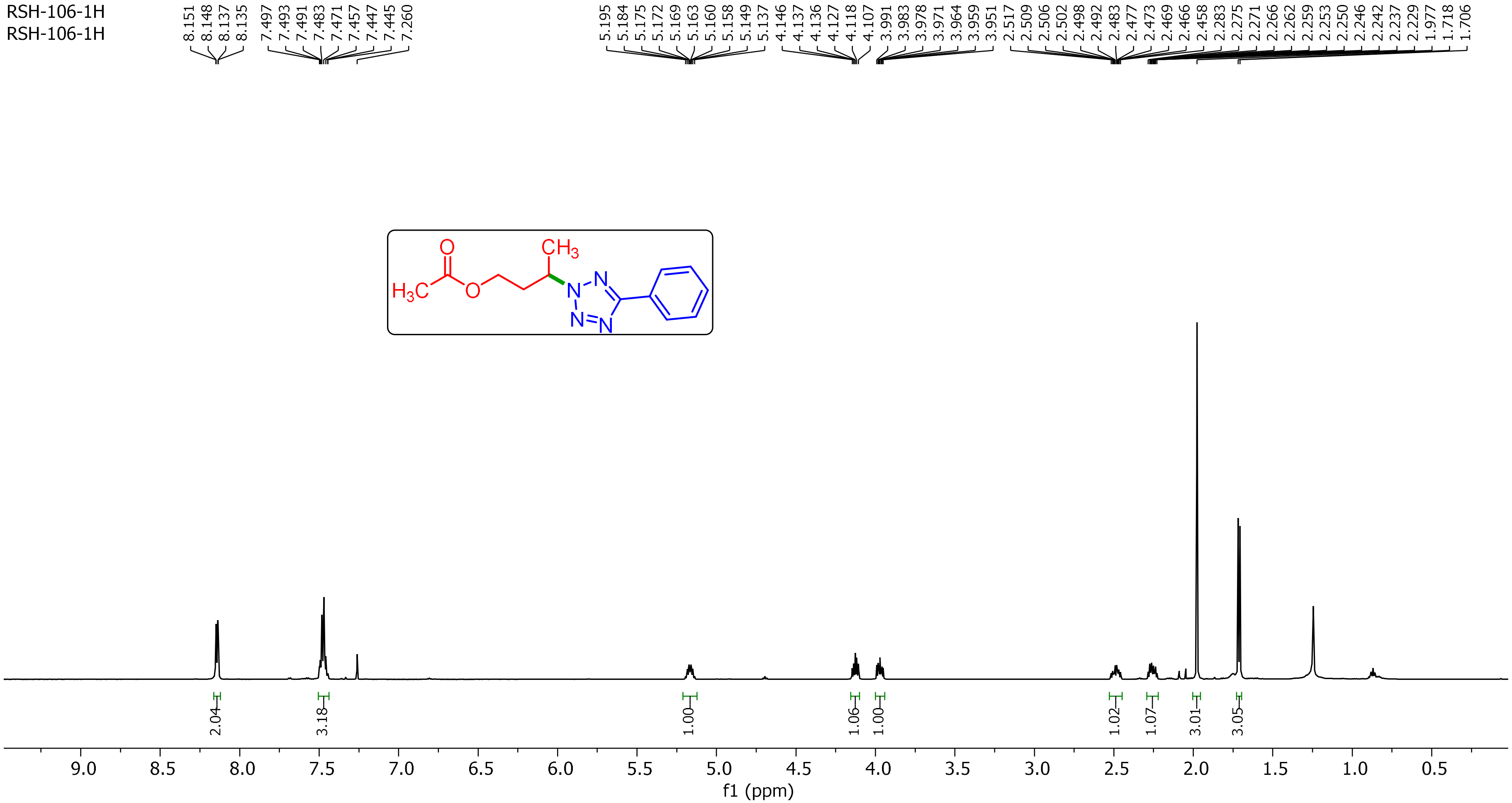

**3-(5-Phenyl-2*H*-tetrazol-2-yl)butyl acetate (1a): $^{13}$C NMR (151 MHz, CDCl$_3$)**

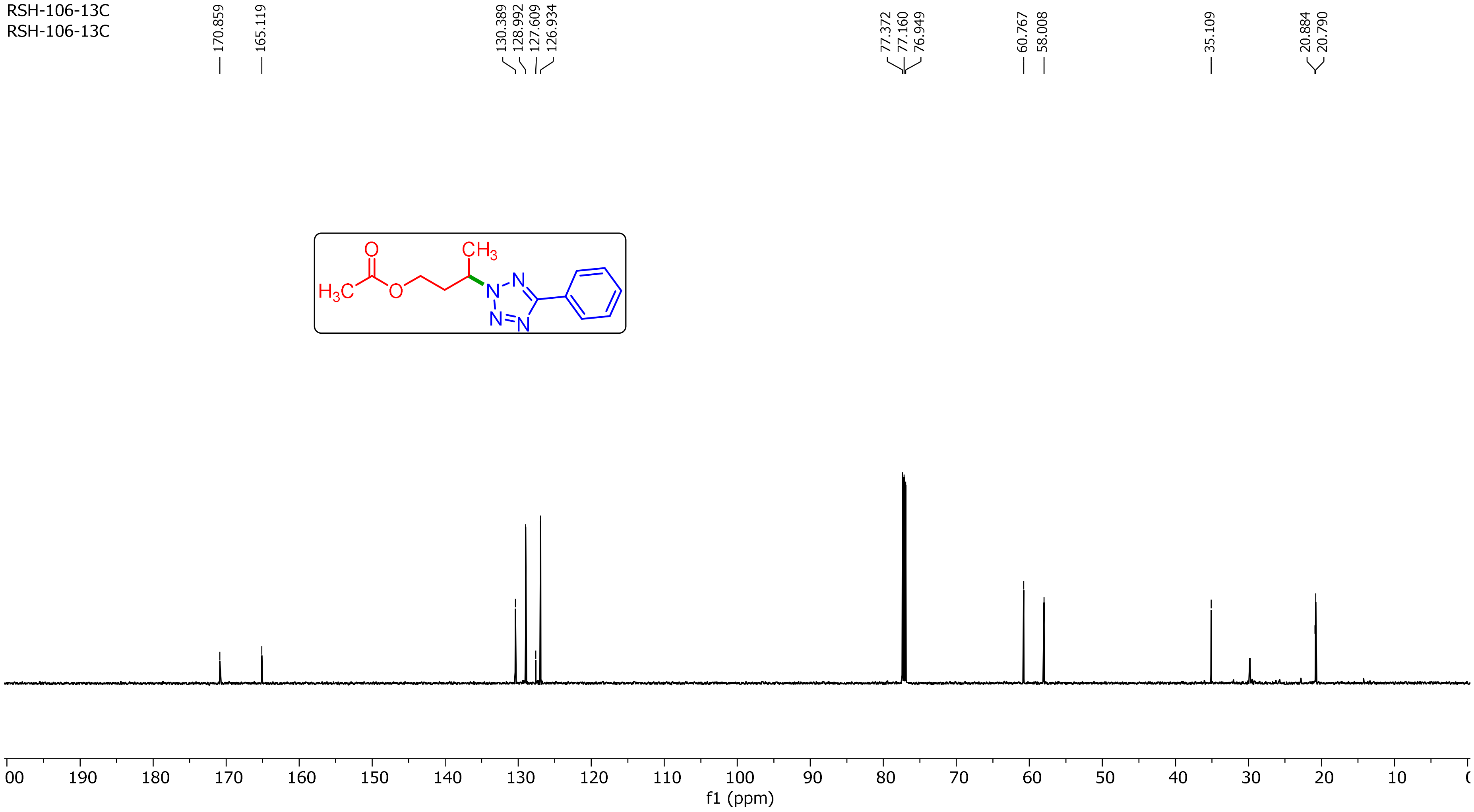

**3-(5-(Naphthalen-2-yl)-2*H*-tetrazol-2-yl)butyl acetate (1b): $^{1}$H NMR (600 MHz, CDCl$_3$)**

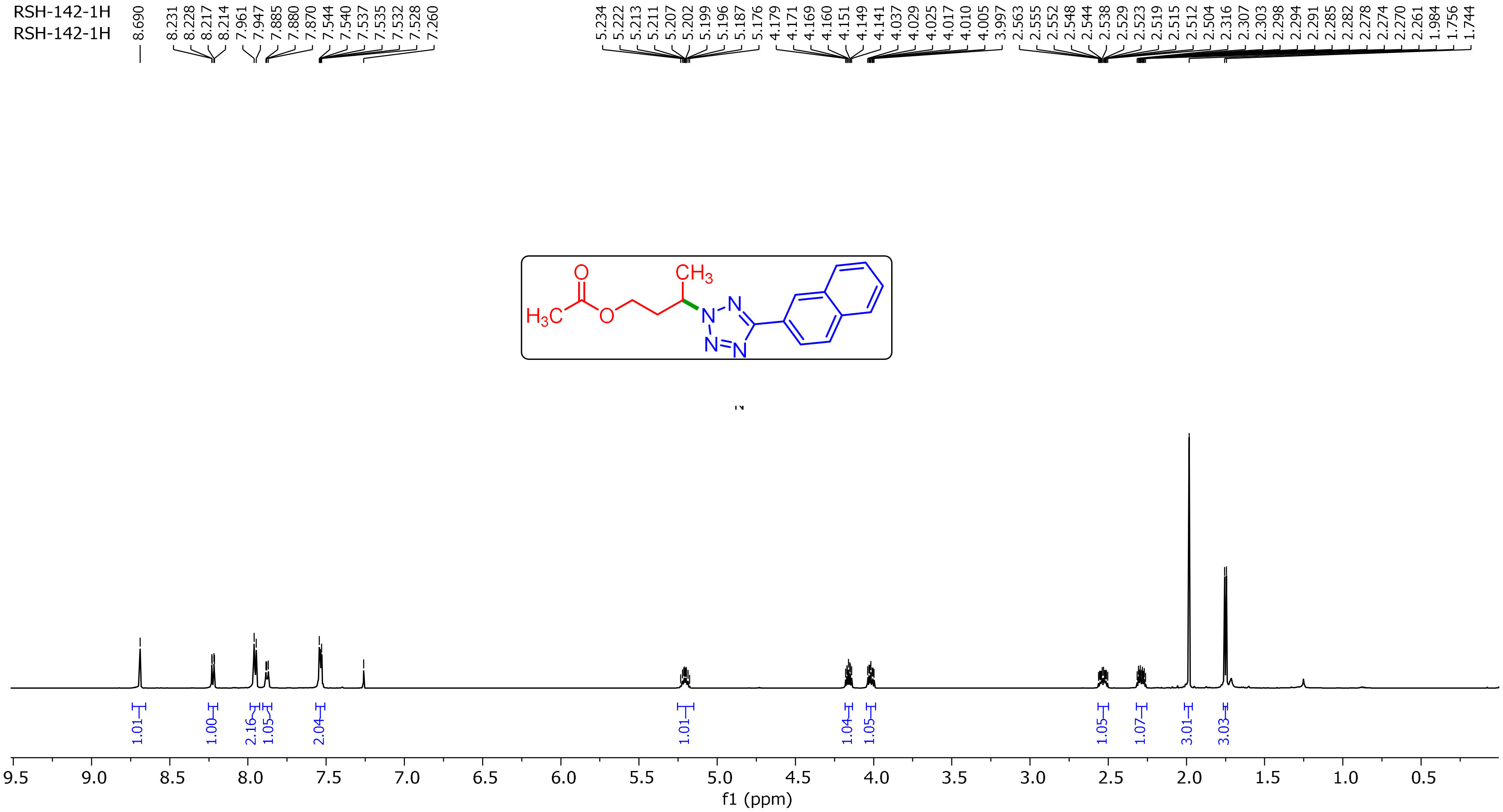

**3-(5-(Naphthalen-2-yl)-2*H*-tetrazol-2-yl)butyl acetate (1b): $^{13}$C NMR (151 MHz, $CDCl_3$)**

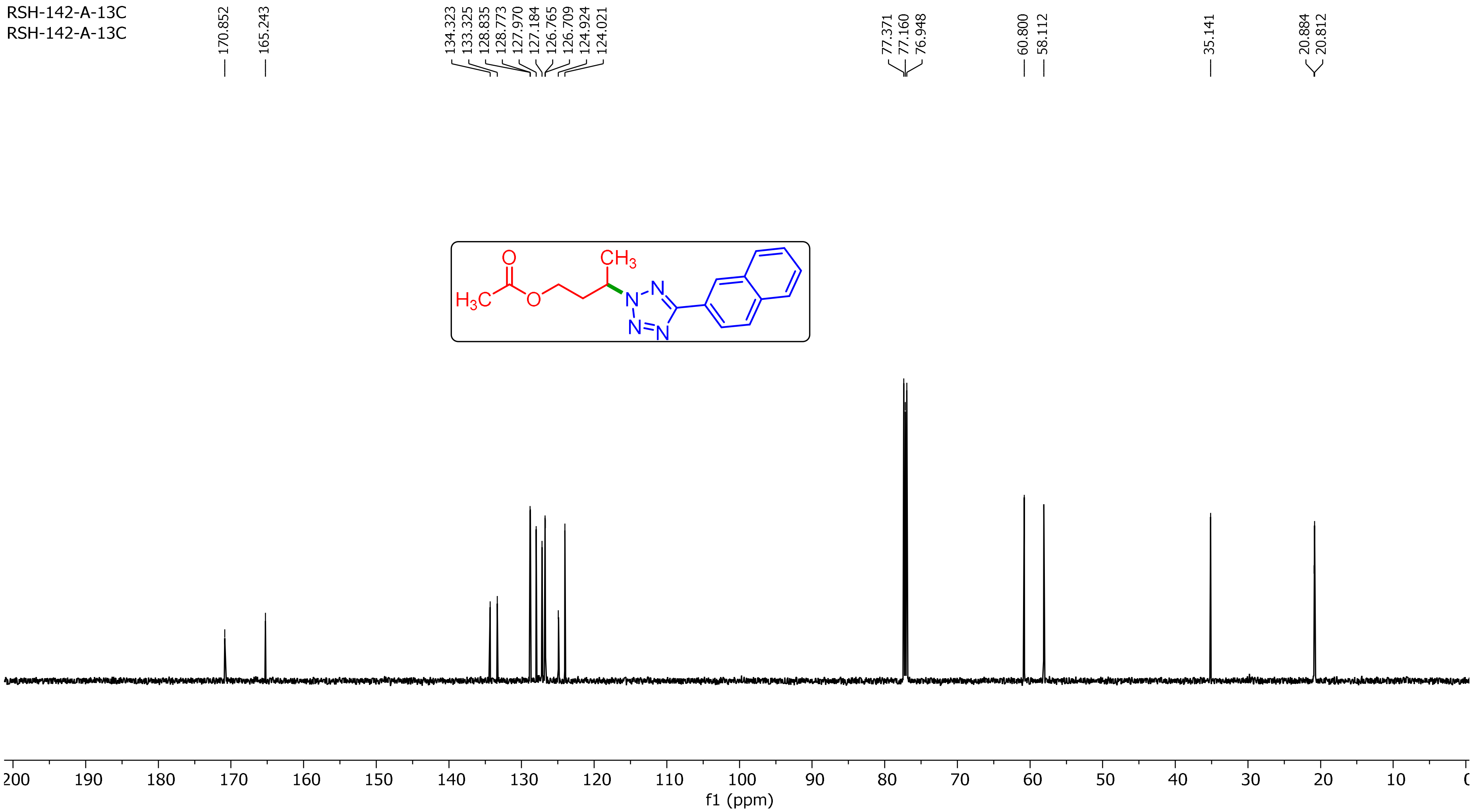

**3-(5-([1,1'-Biphenyl]-4-yl)-2*H*-tetrazol-2-yl)butyl acetate (1c): $^{1}$H NMR (600 MHz, $CDCl_3$)**

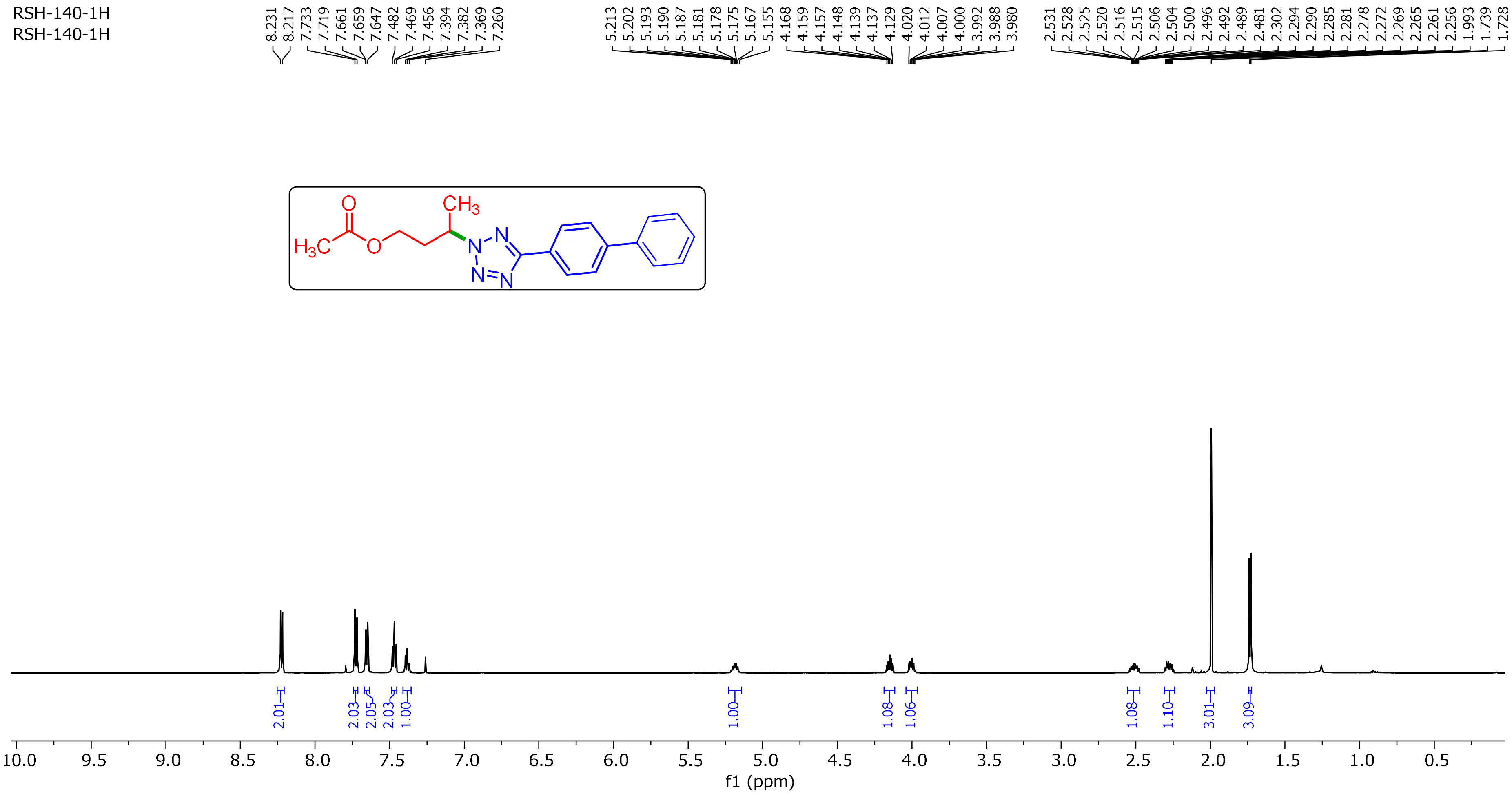

**3-(5-([1,1'-Biphenyl]-4-yl)-2*H*-tetrazol-2-yl)butyl acetate (1c): $^{13}$C NMR (151 MHz, CDCl$_3$)**

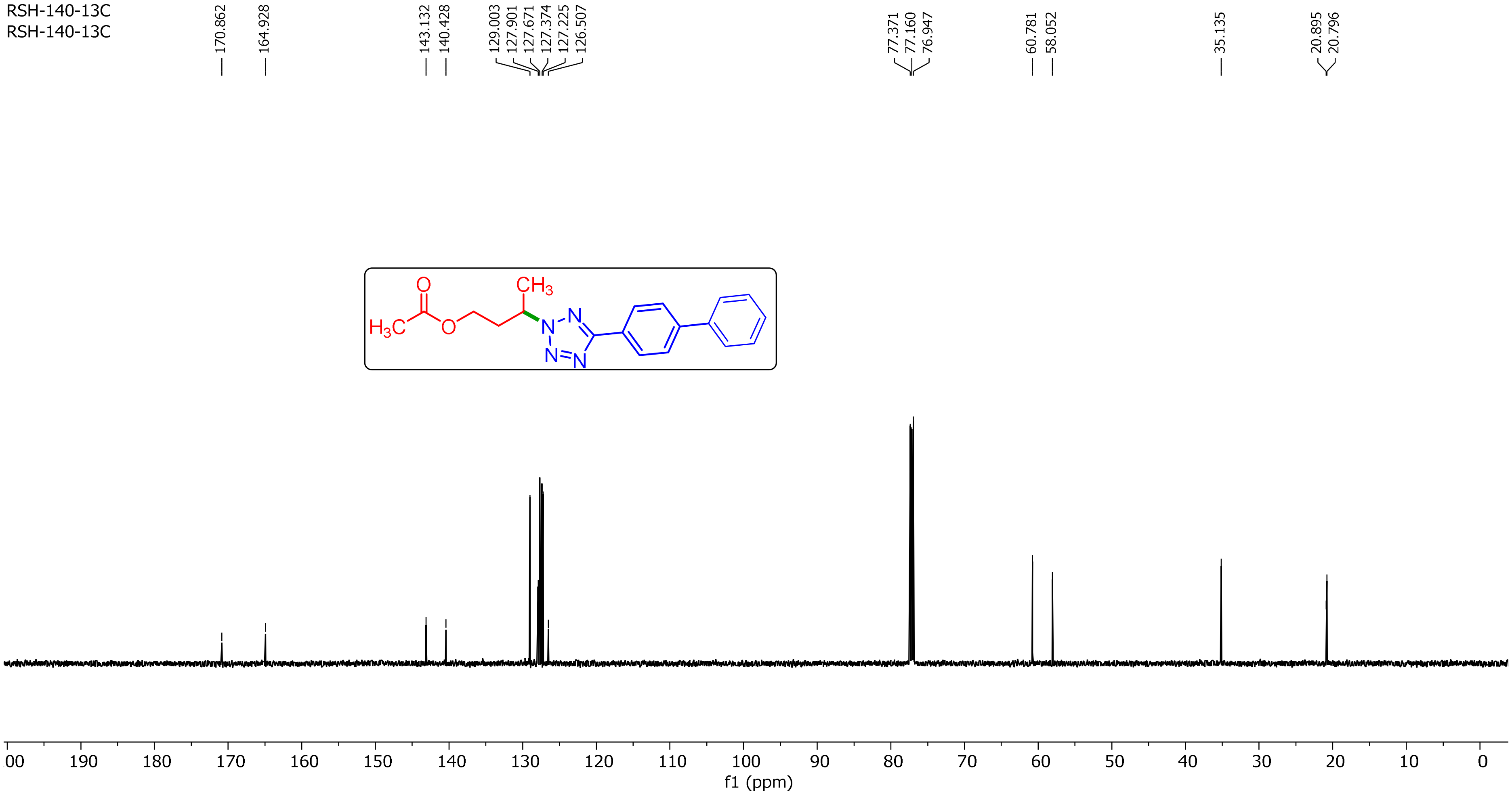

**3-(5-(*p*-Tolyl)-2*H*-tetrazol-2-yl)butyl acetate (1d): $^{1}$H NMR (600 MHz, CDCl$_3$)**

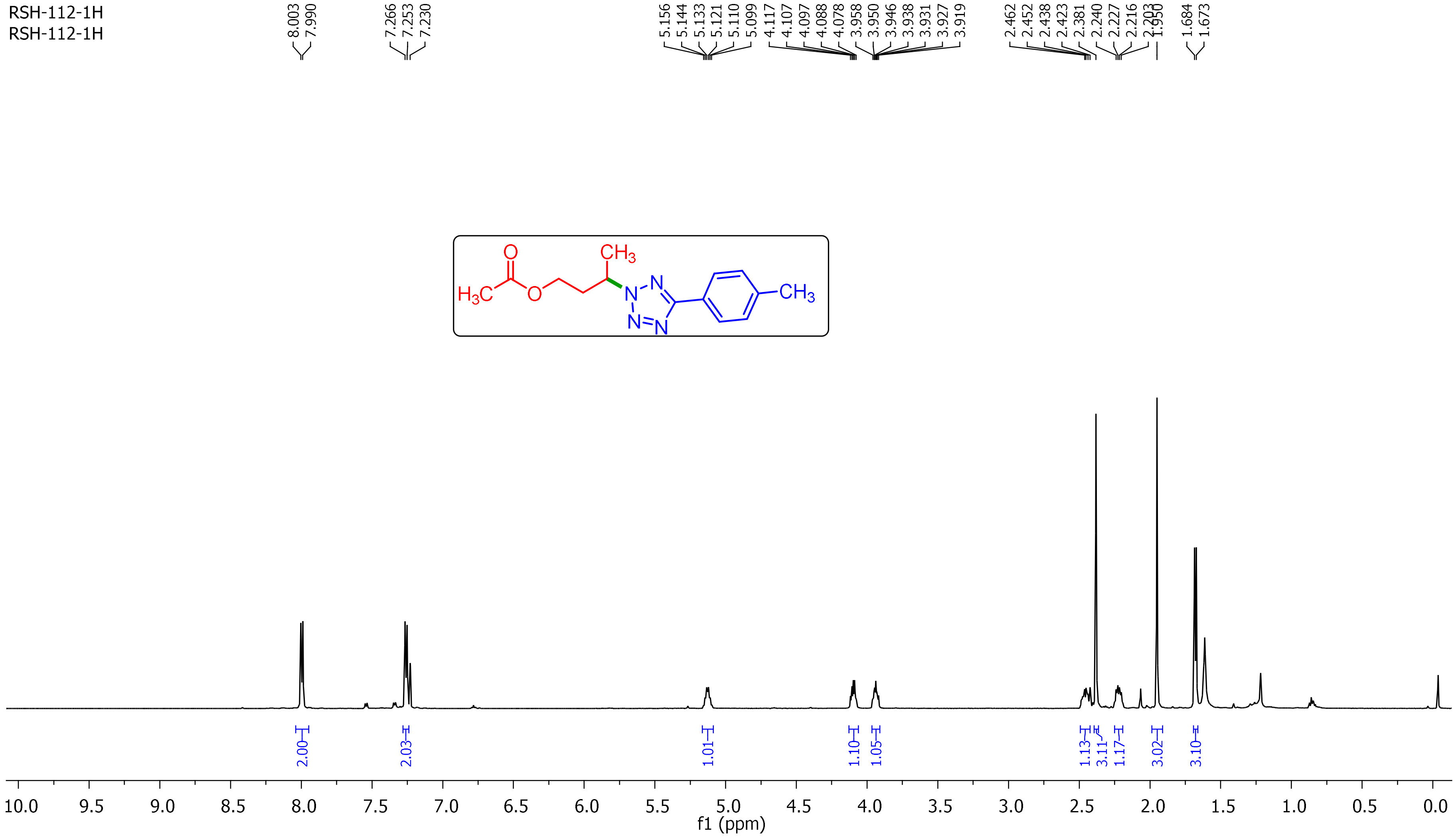

**3-(5-(*p*-Tolyl)-2*H*-tetrazol-2-yl)butyl acetate (1d): $^{13}$C NMR (151 MHz, $CDCl_3$)**

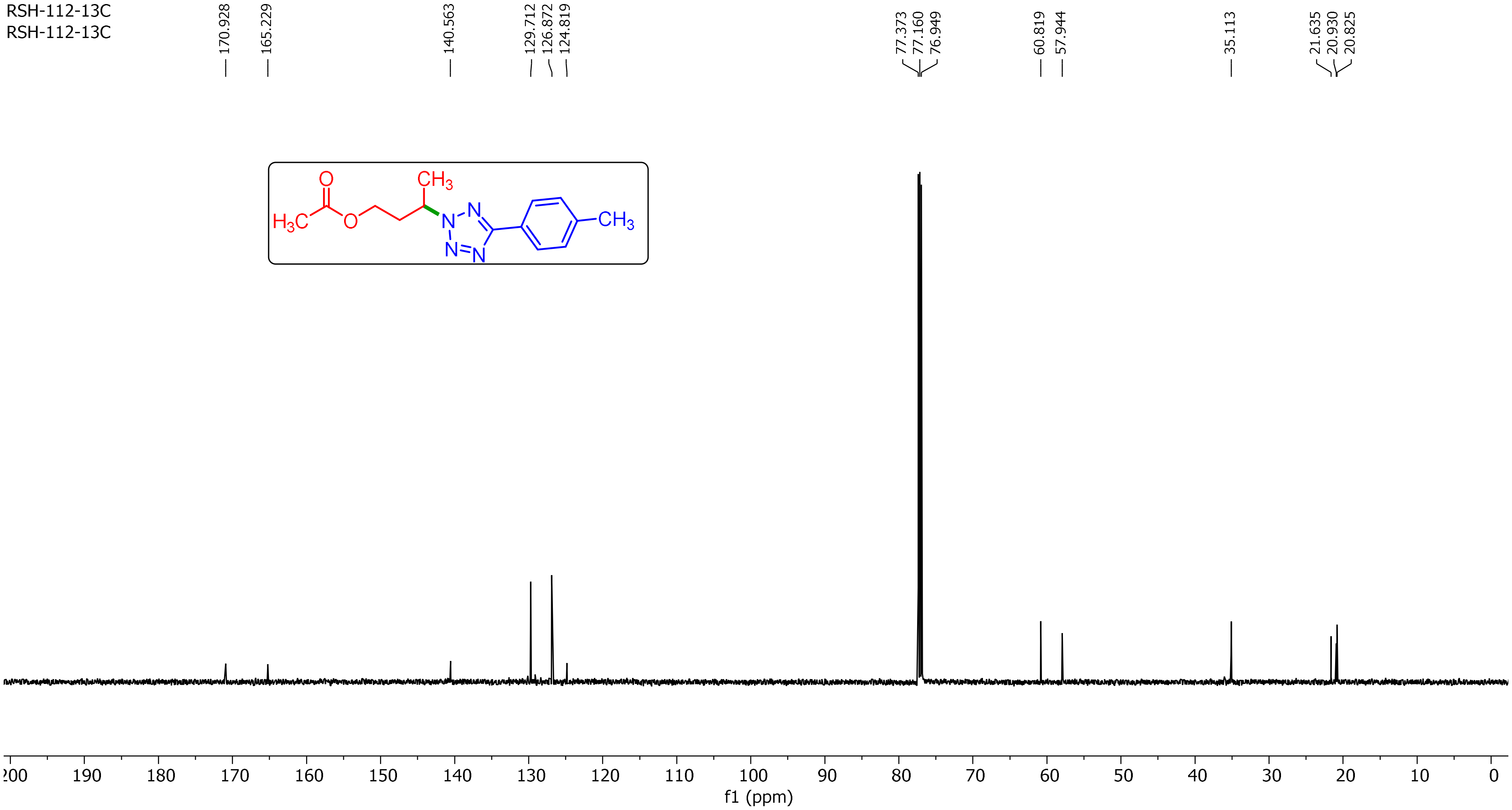

**3-(5-(4-Methoxyphenyl)-2*H*-tetrazol-2-yl)butyl acetate (1e): $^{1}$H NMR (600 MHz, $CDCl_3$)**

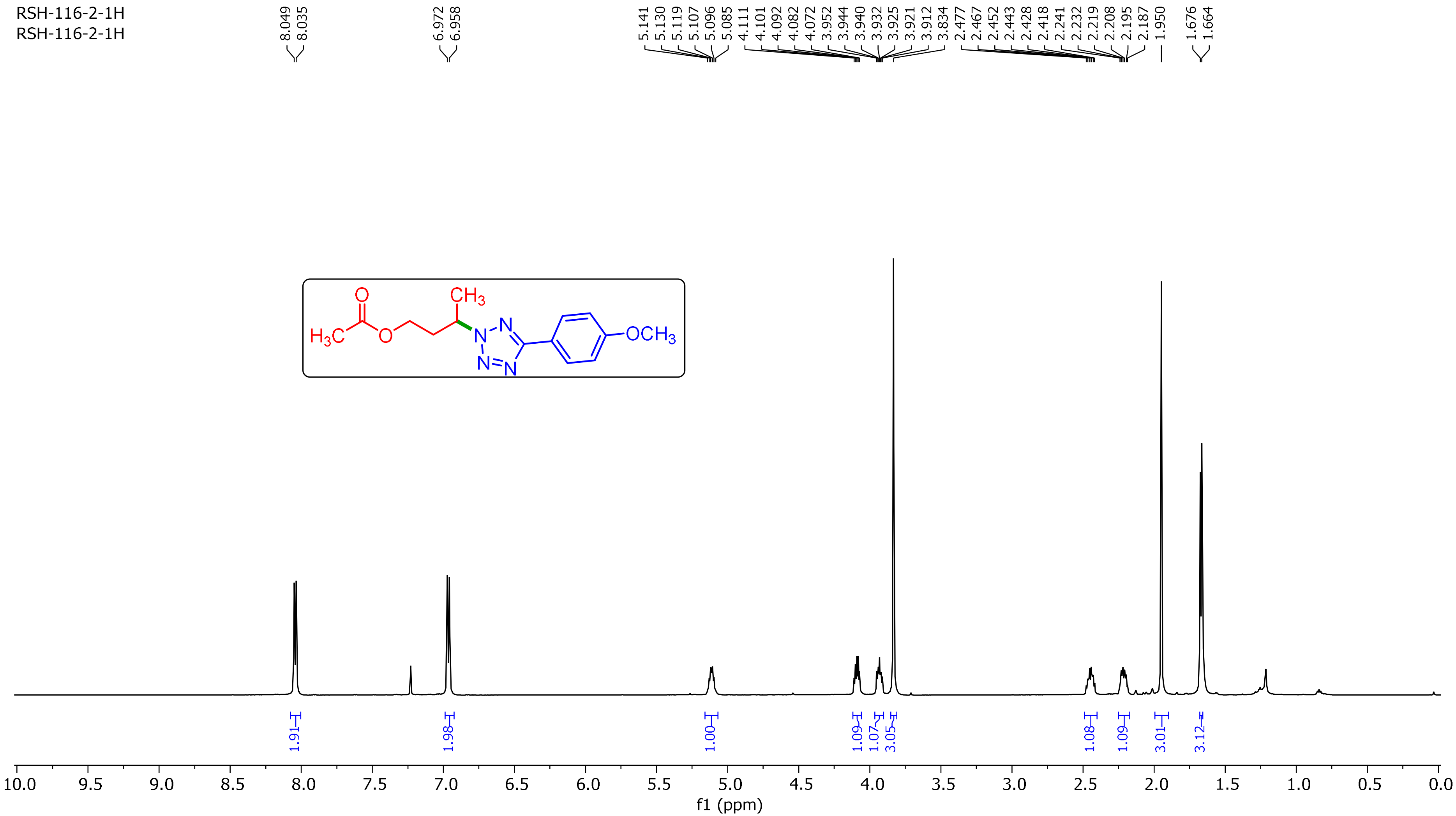

**3-(5-(4-Methoxyphenyl)-2*H*-tetrazol-2-yl)butyl acetate (1e): $^{13}$C NMR (151 MHz, CDCl$_3$)**

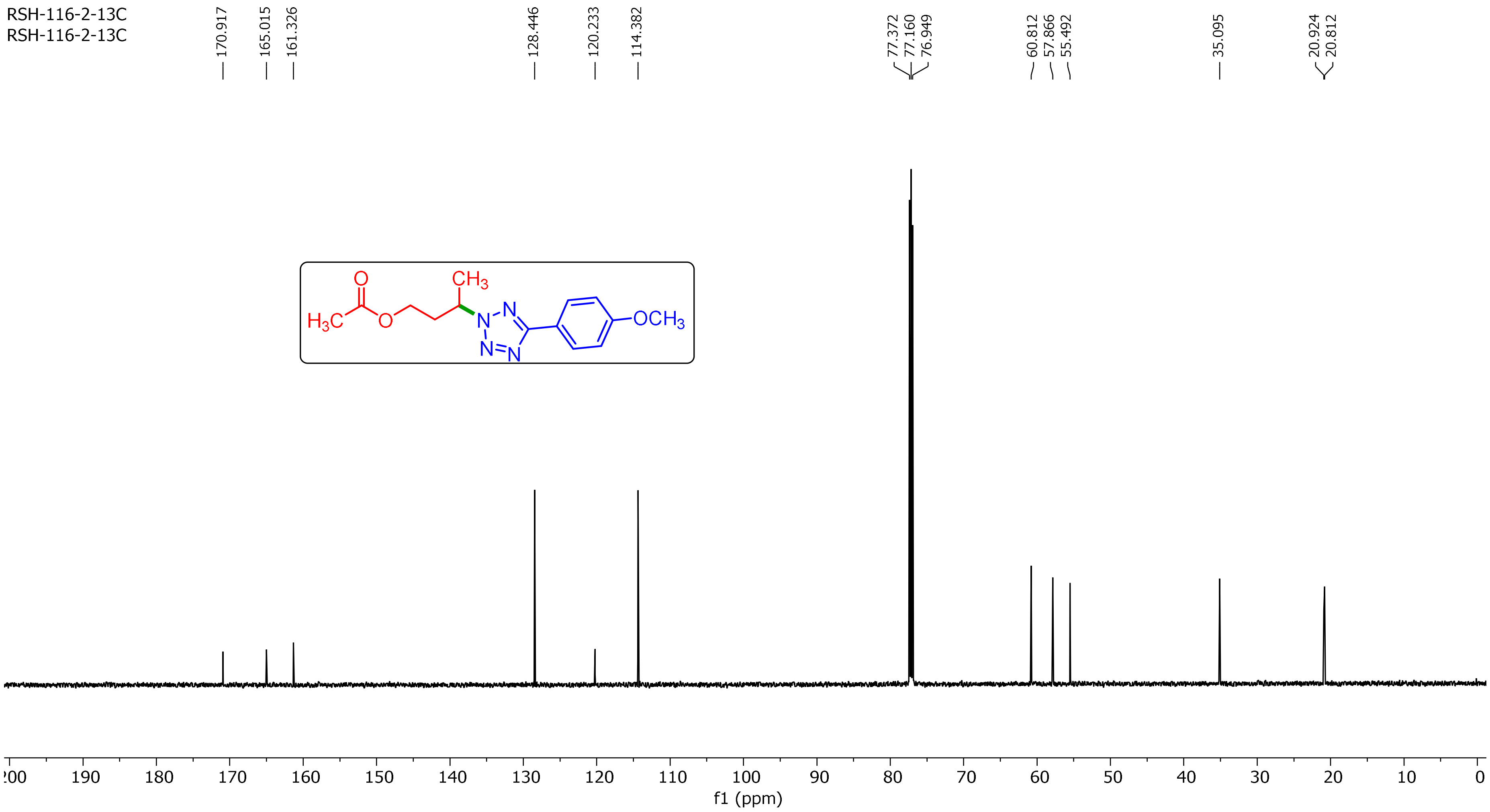

**3-(5-(4-(*tert*-Butyl)phenyl)-2*H*-tetrazol-2-yl)butyl acetate (1f): $^{1}$H NMR (600 MHz, CDCl$_3$)**

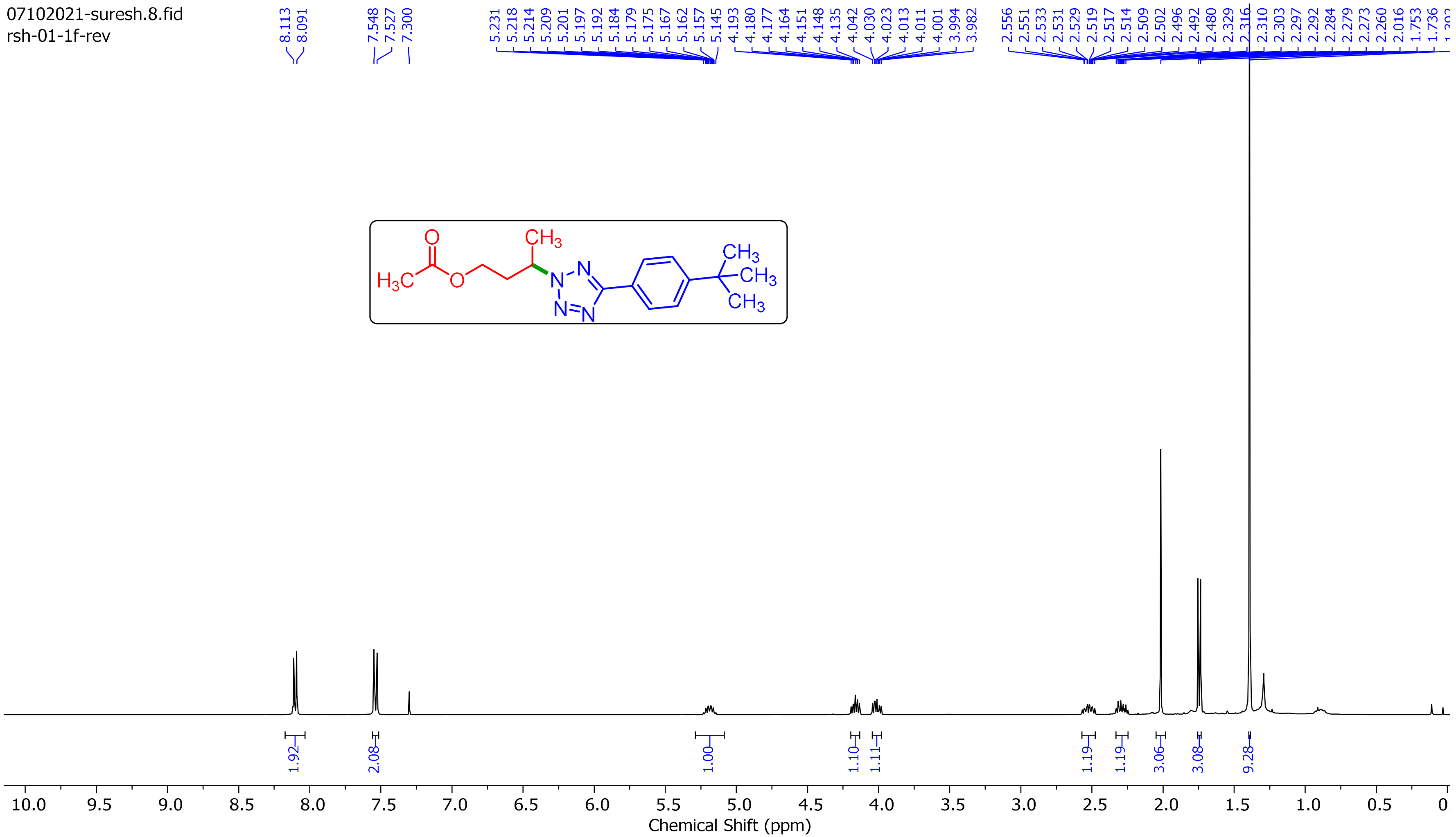

**3-(5-(4-(*tert*-Butyl)phenyl)-2*H*-tetrazol-2-yl)butyl acetate (1f): $^{13}$C NMR (151 MHz, CDCl$_3$)**

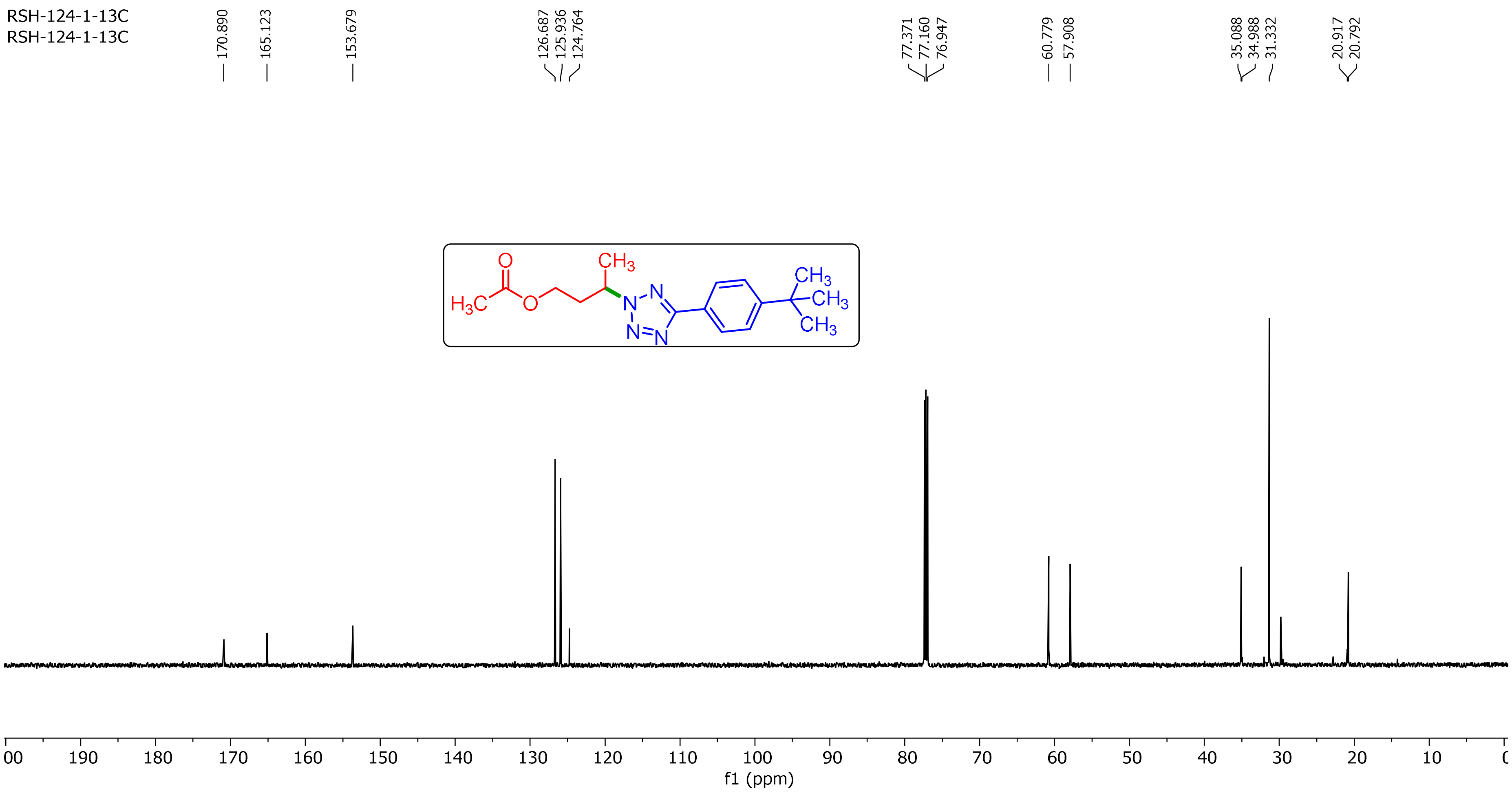

**3-(5-(3-Methoxyphenyl)-2*H*-tetrazol-2-yl)butyl acetate (1g): $^{1}$H NMR (600 MHz, $CDCl_3$)**

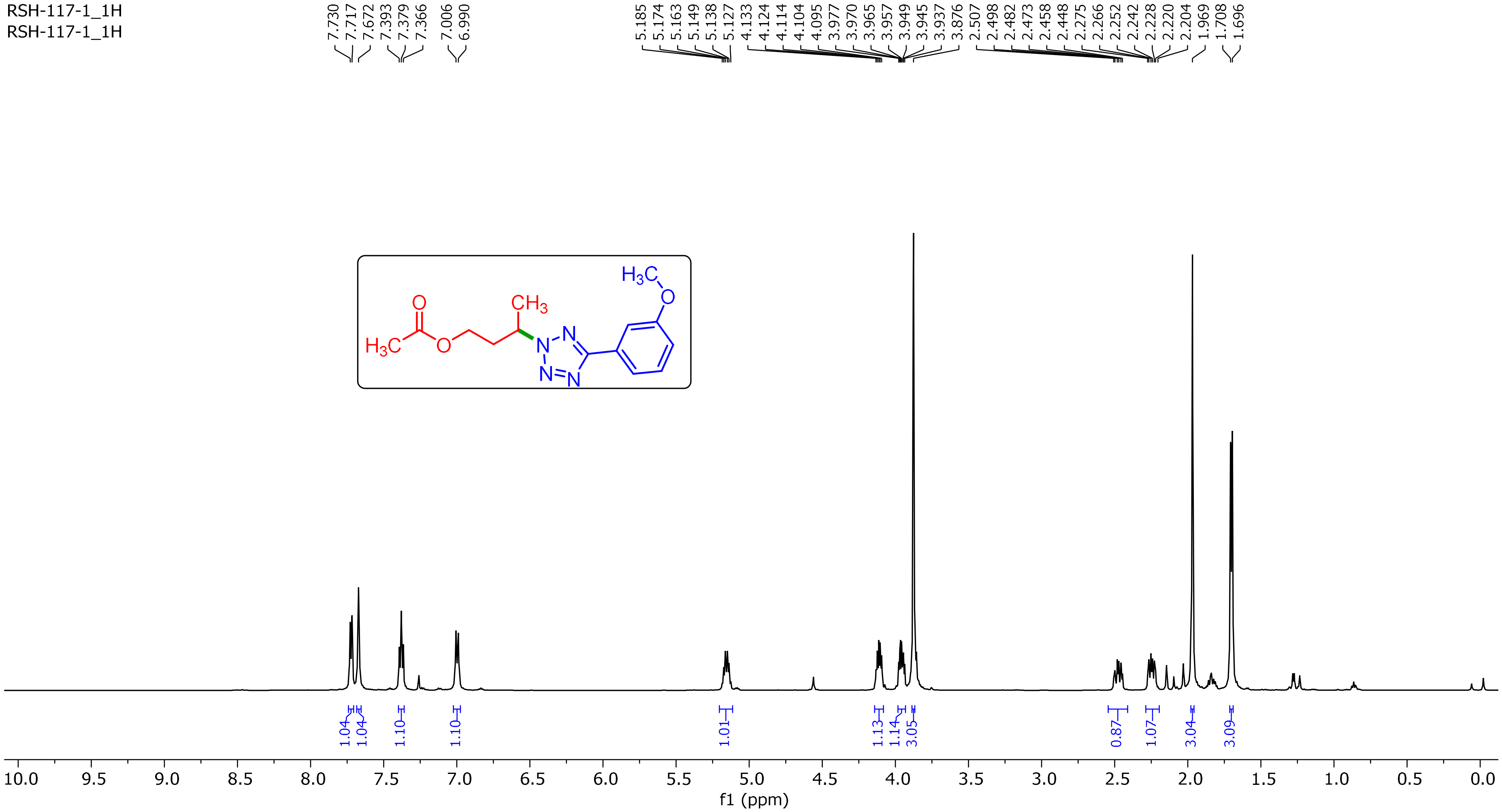

**3-(5-(3-Methoxyphenyl)-2*H*-tetrazol-2-yl)butyl acetate (1g): $^{13}C$ NMR (151 MHz, $CDCl_3$)**

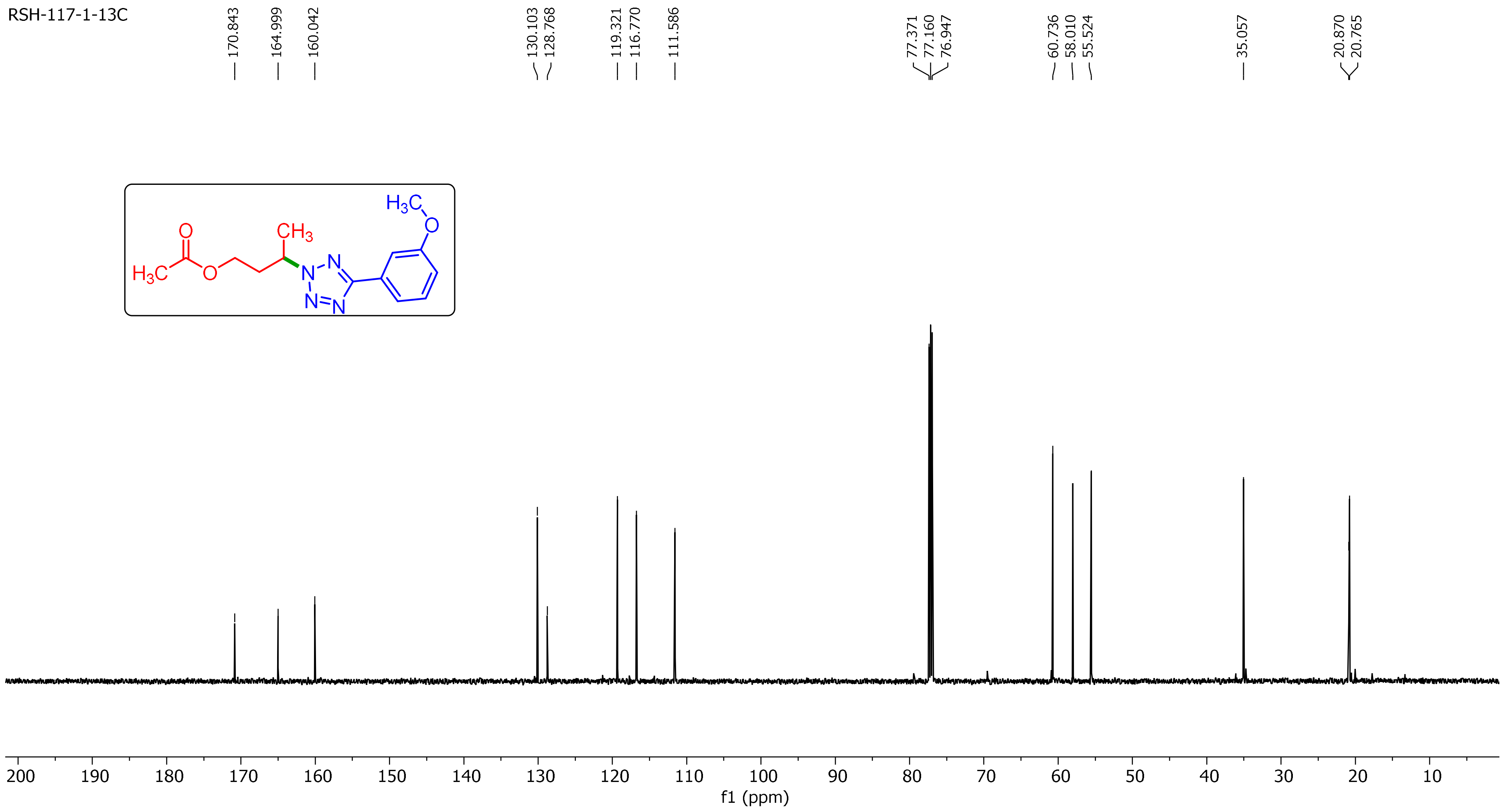

**3-(5-(4-Chlorophenyl)-2*H*-tetrazol-2-yl)butyl acetate (1h): $^{1}$H NMR (600 MHz, CDCl₃)**

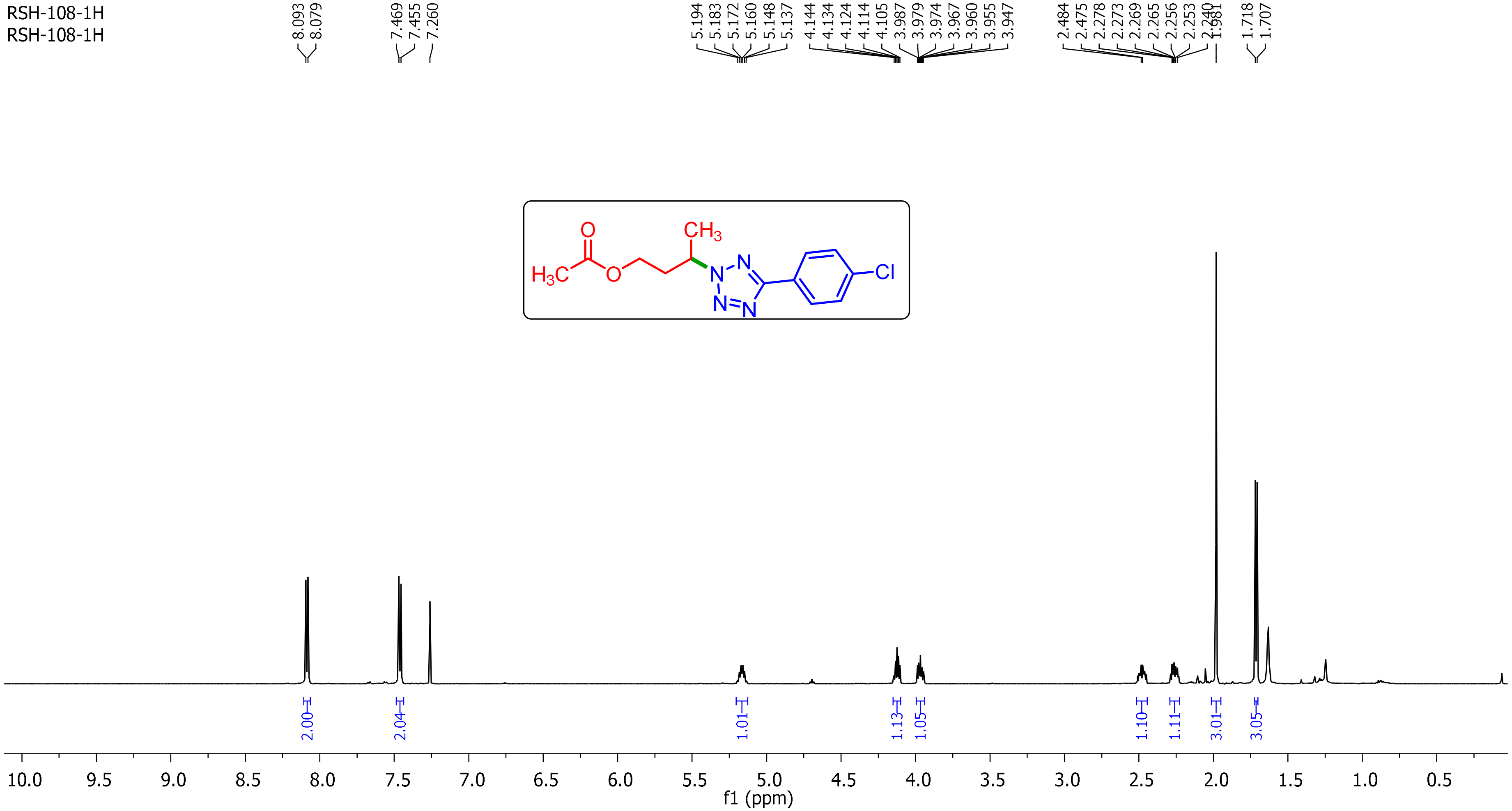

**3-(5-(4-Chlorophenyl)-2*H*-tetrazol-2-yl)butyl acetate (1h): $^{13}$C NMR (151 MHz, $CDCl_3$)**

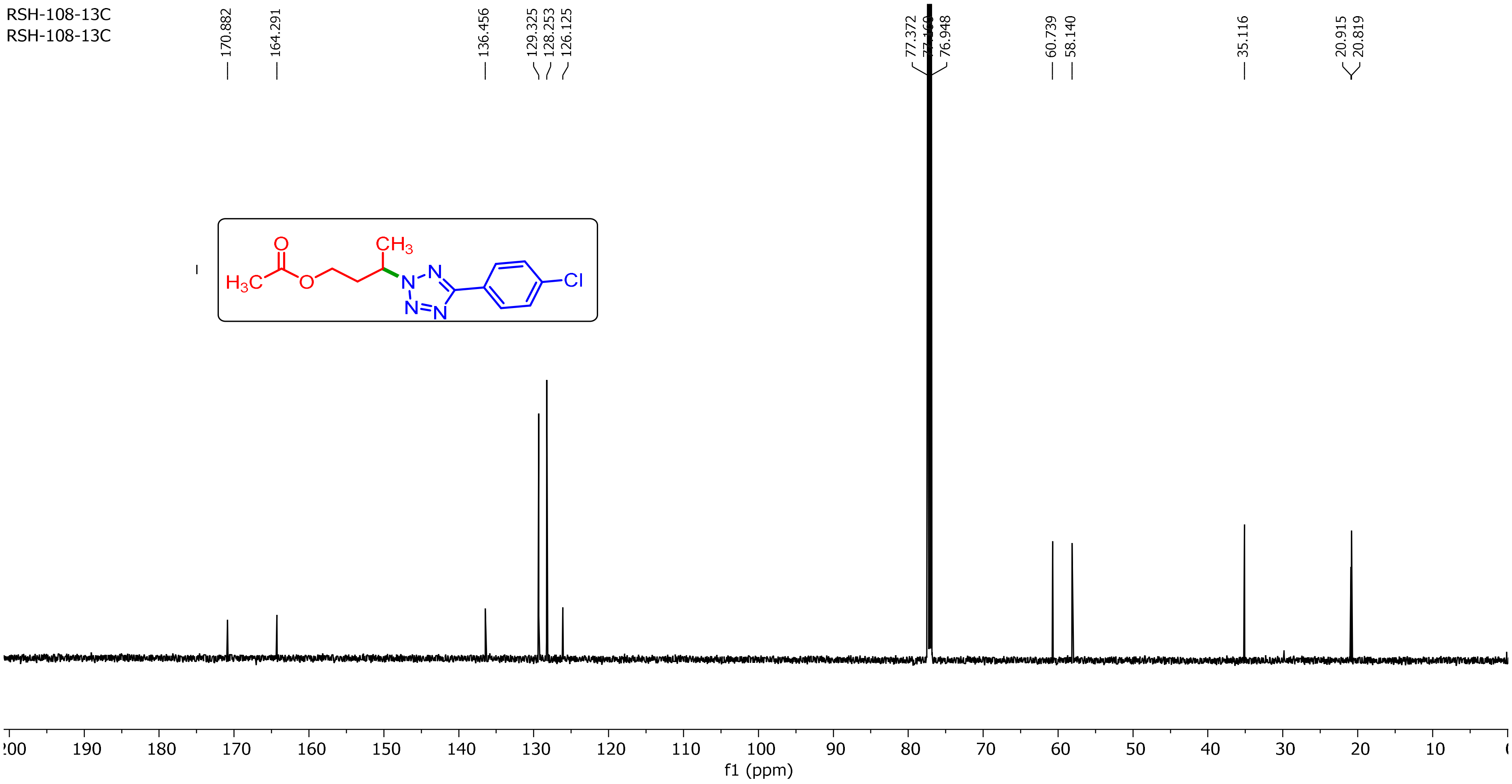

**3-(5-(4-Bromophenyl)-2*H*-tetrazol-2-yl)butyl acetate (1i): $^{1}$H NMR (400 MHz, $CDCl_3$)**

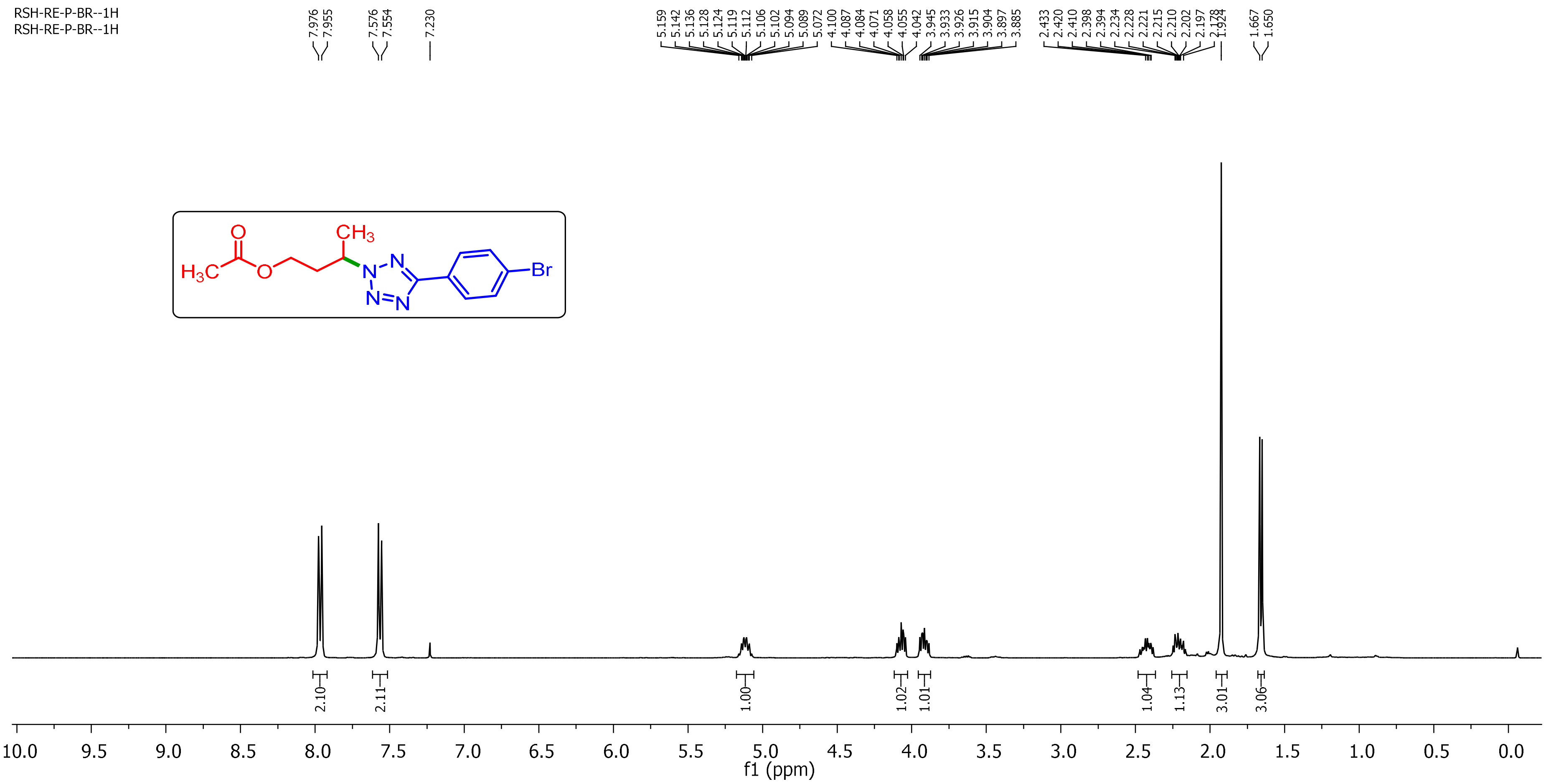

**3-(5-(4-Bromophenyl)-2*H*-tetrazol-2-yl)butyl acetate (1i): $^{13}$C NMR (151 MHz, $CDCl_3$)**

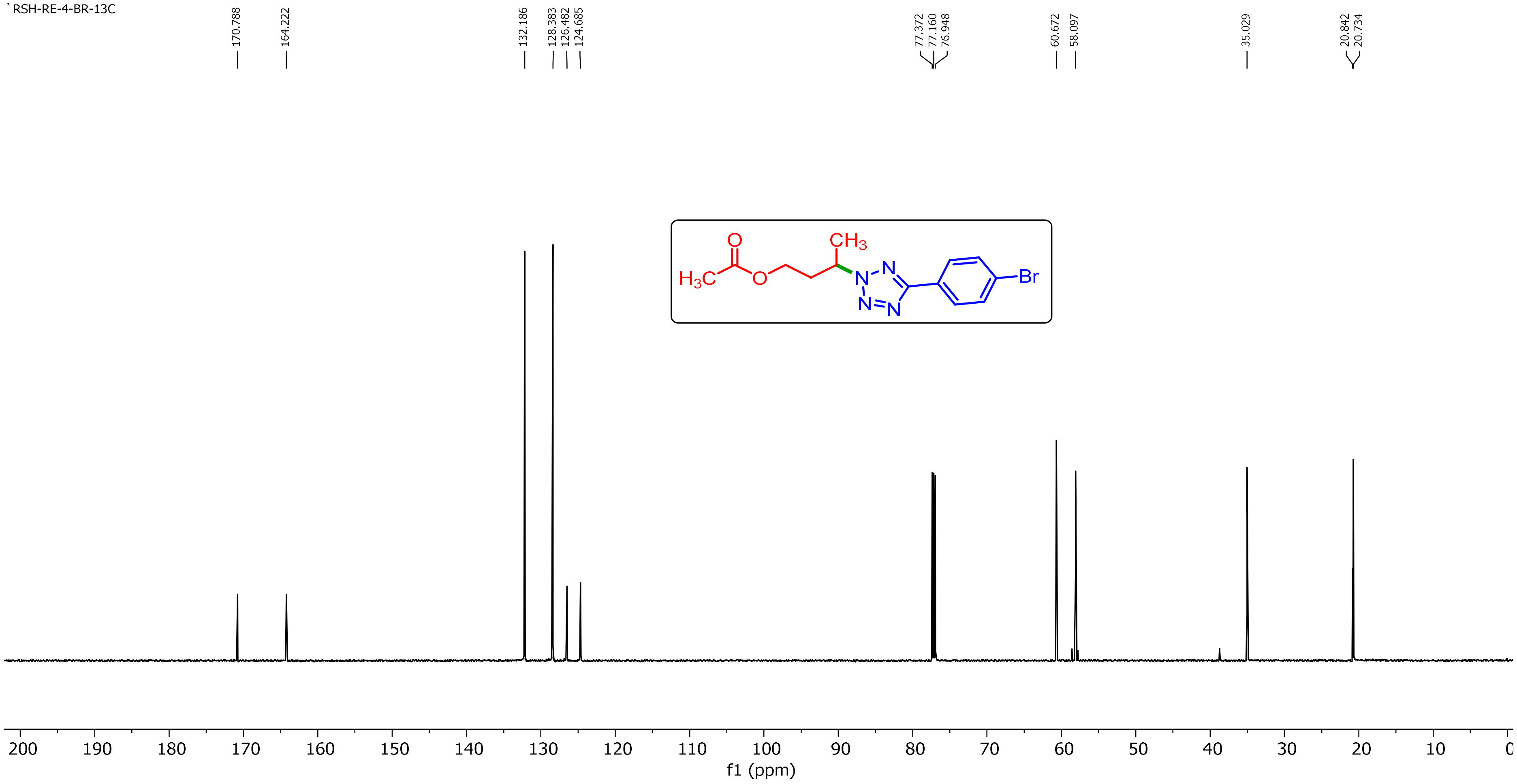

## 3-(5-(4-(Trifluoromethyl)phenyl)-2*H*-tetrazol-2-yl)butyl acetate (1j): $^{1}$H NMR (600 MHz, $CDCl_3$)

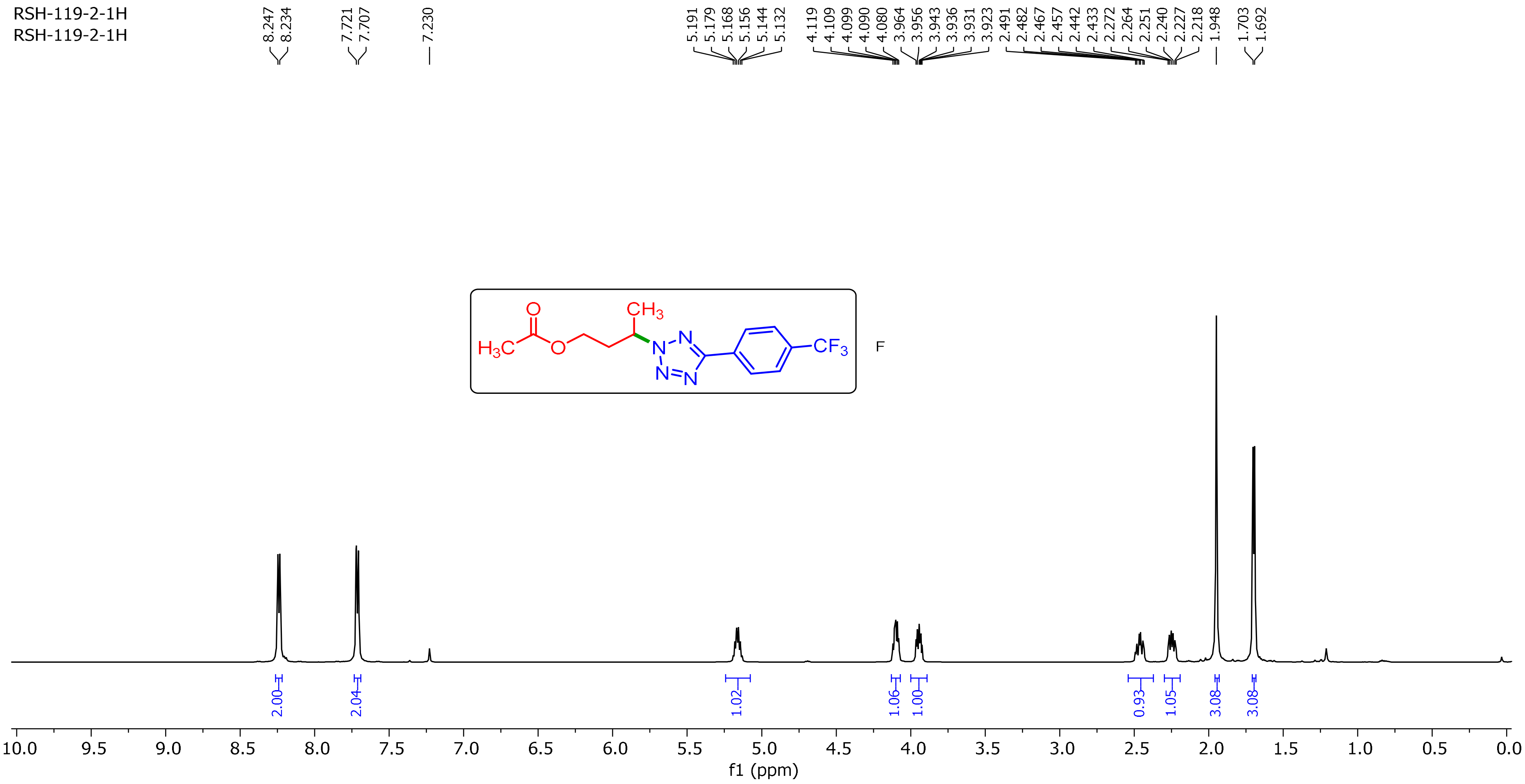

**3-(5-(4-(Trifluoromethyl)phenyl)-2*H*-tetrazol-2-yl)butyl acetate (1j): $^{13}$C NMR (151 MHz, $CDCl_3$)**

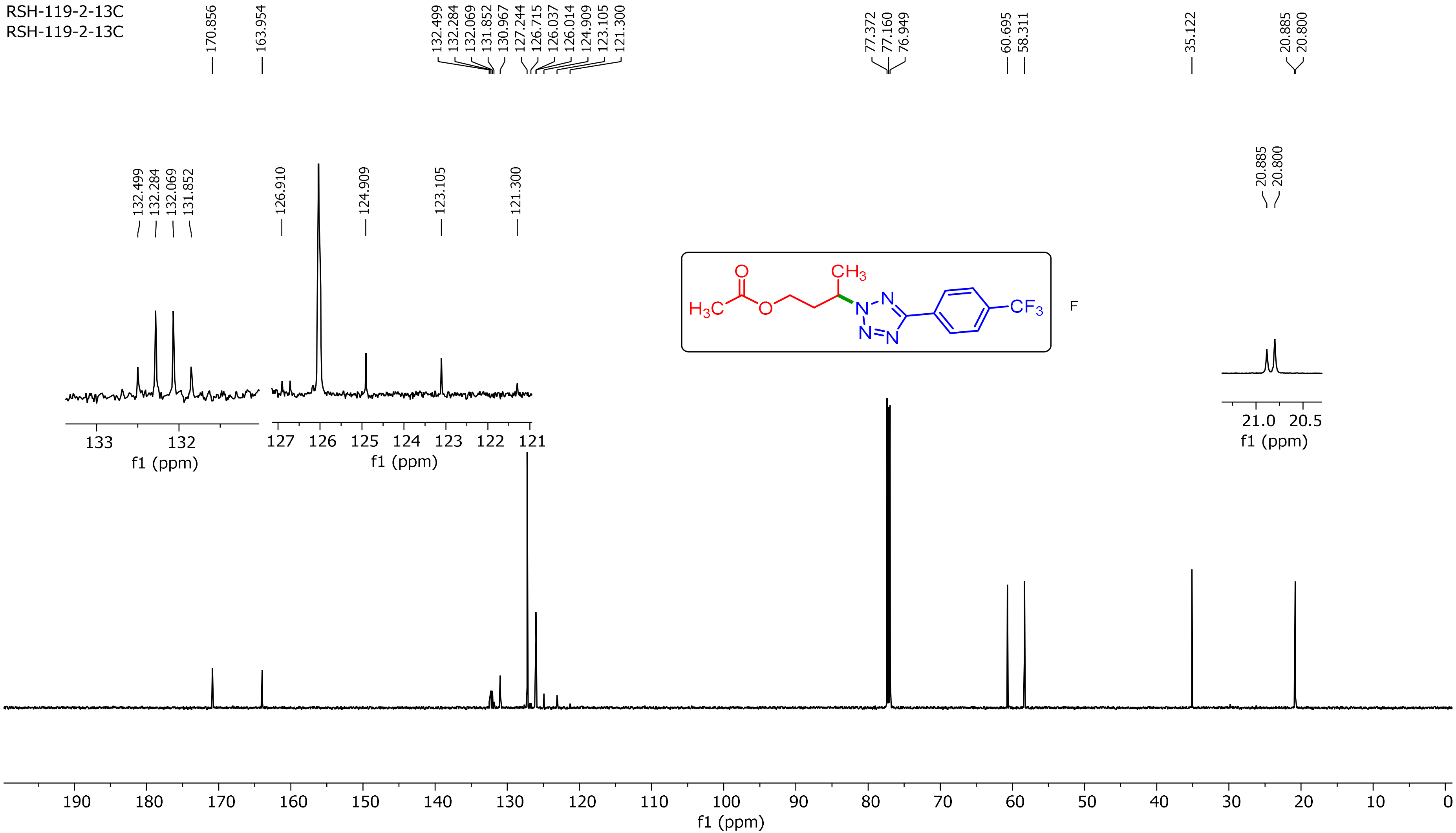

**3-(5-(4-(Trifluoromethyl)phenyl)-2*H*-tetrazol-2-yl)butyl acetate (1j): $^{19}$F NMR (565 MHz, $CDCl_3$)**

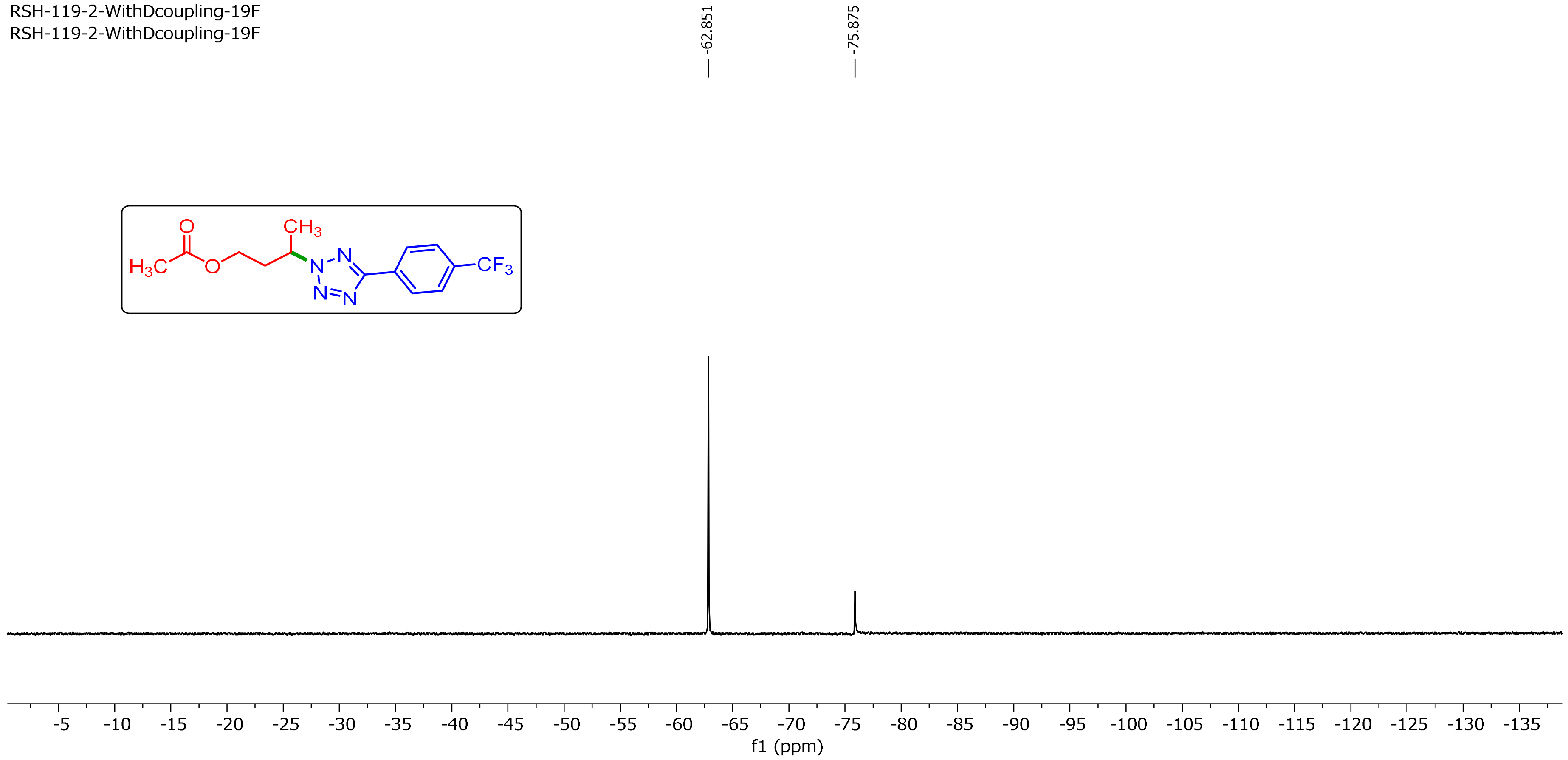

**3-(5-(4-(Trifluoromethyl)phenyl)-2*H*-tetrazol-2-yl)butyl acetate (1j): $^{19}$F NMR (565 MHz, $CDCl_3$)**

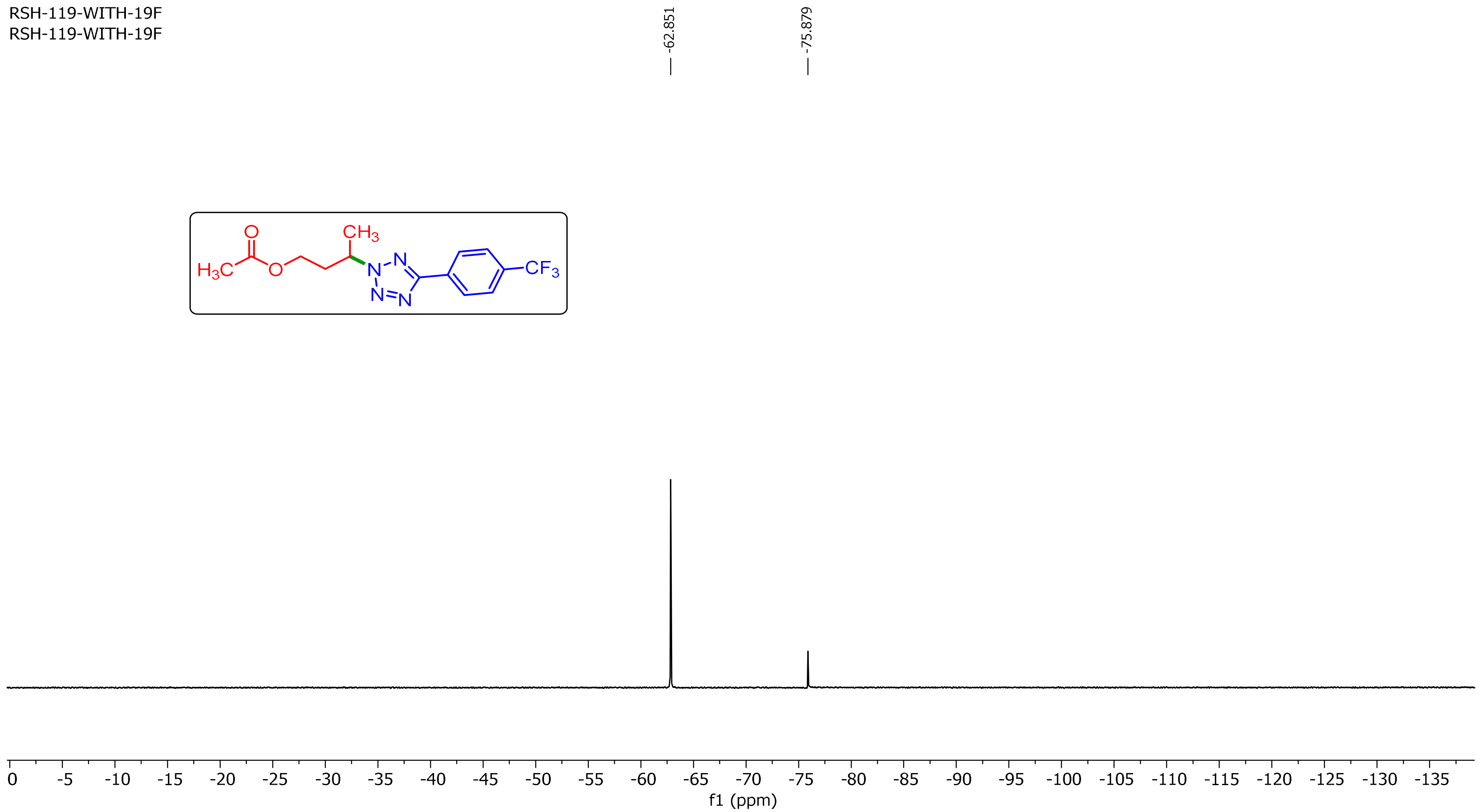

**3-(5-(4-Nitrophenyl)-2*H*-tetrazol-2-yl)butyl acetate (1k): $^{1}$H NMR (600 MHz, $CDCl_3$)**

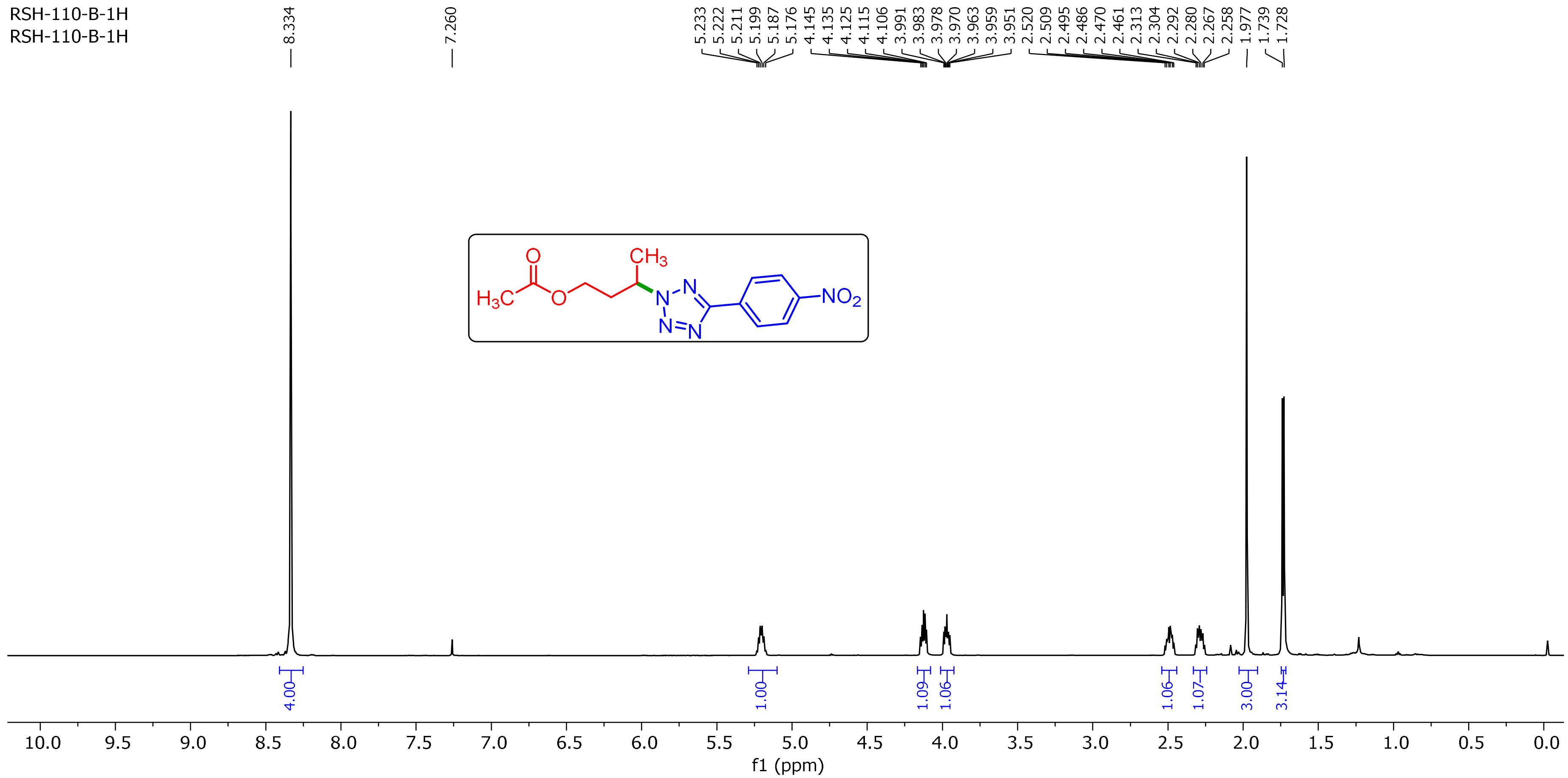

**3-(5-(4-Nitrophenyl)-2*H*-tetrazol-2-yl)butyl acetate (1k): $^{13}$C NMR (151 MHz, CDCl$_3$)**

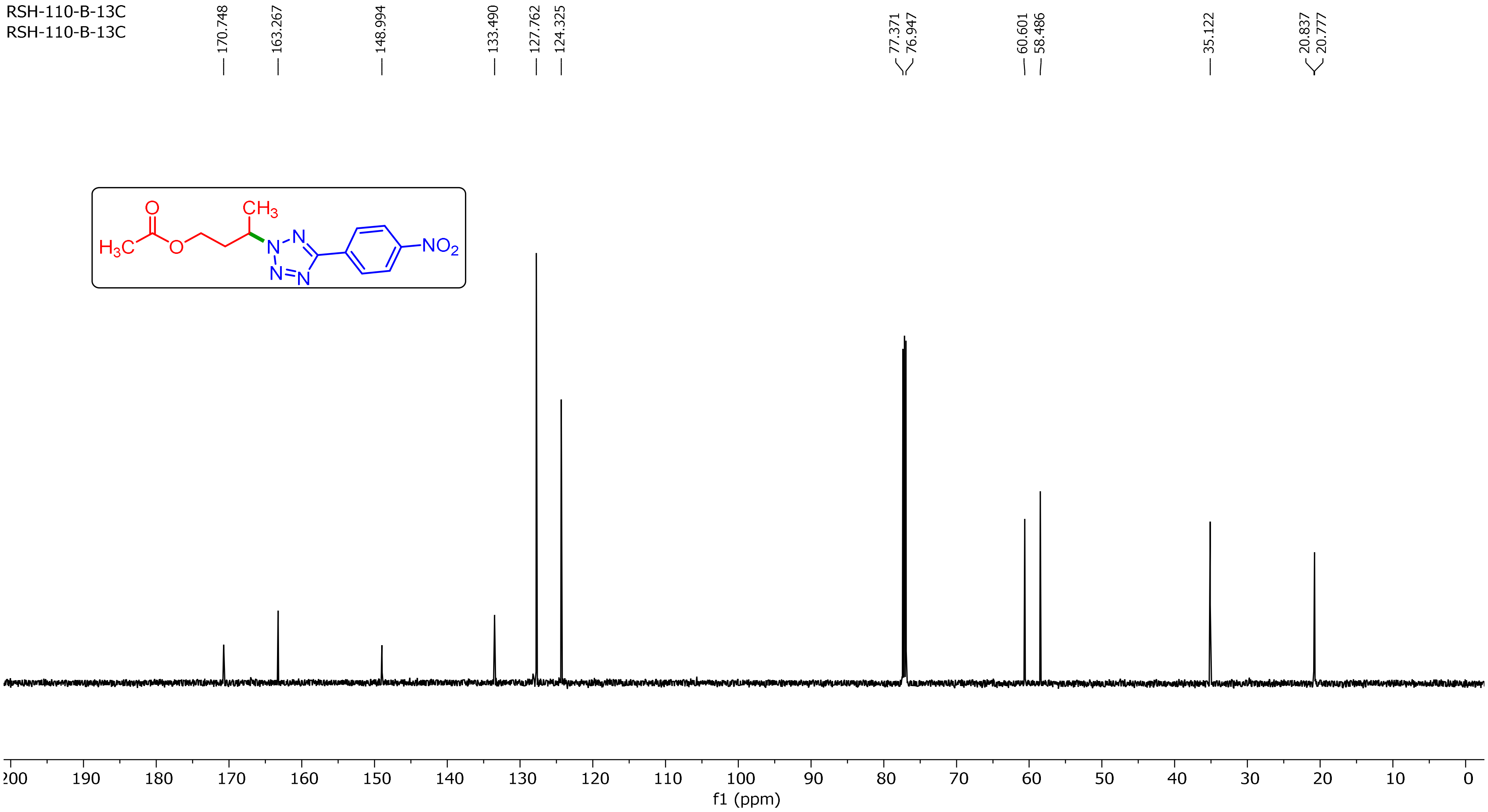

**3-(5-(2-Chlorophenyl)-2*H*-tetrazol-2-yl)butyl acetate (1l): $^1$H NMR (600 MHz, $CDCl_3$)**

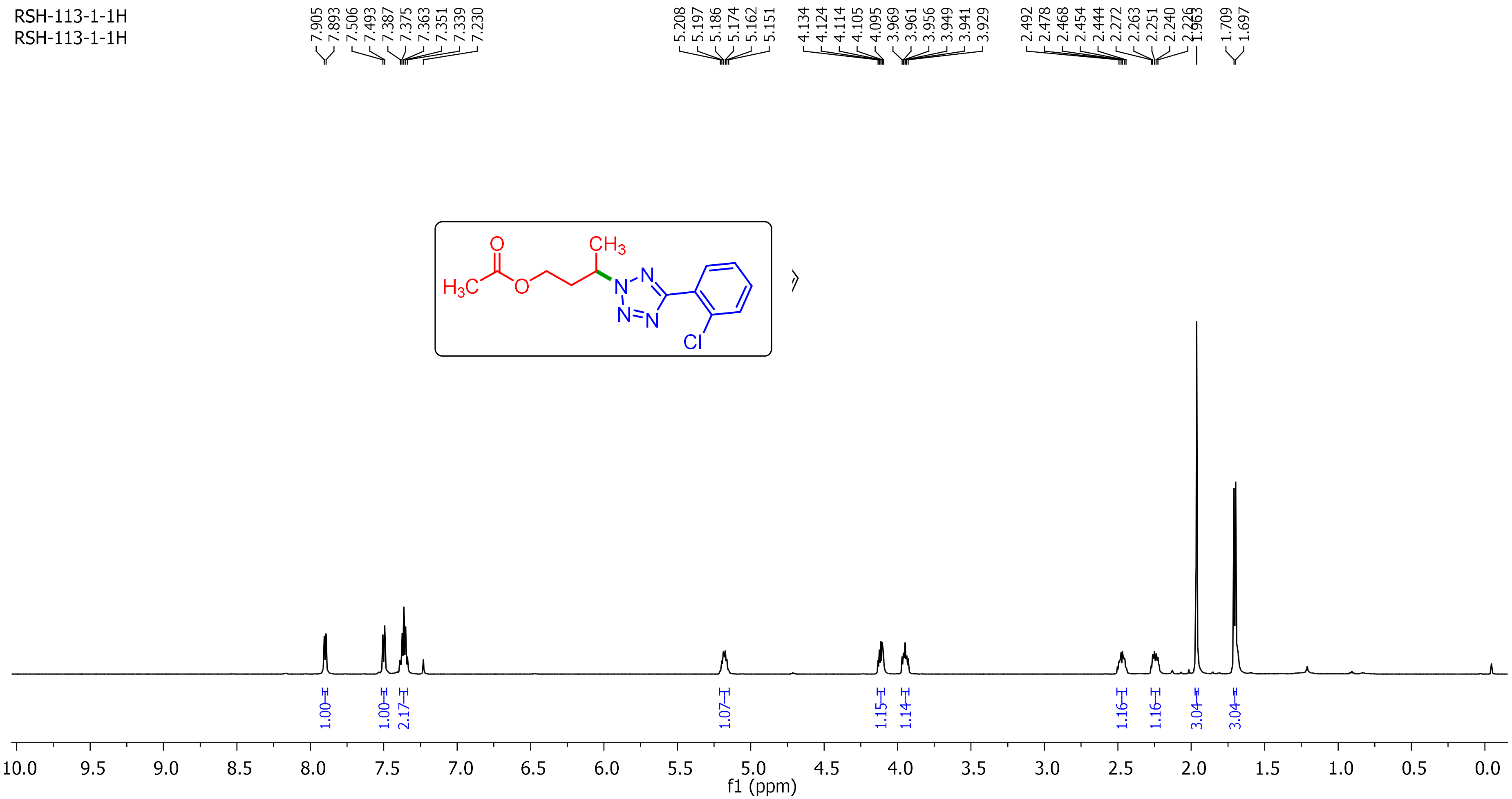

**3-(5-(2-Chlorophenyl)-2*H*-tetrazol-2-yl)butyl acetate (1l): $^{13}$C NMR (151 MHz, $CDCl_3$)**

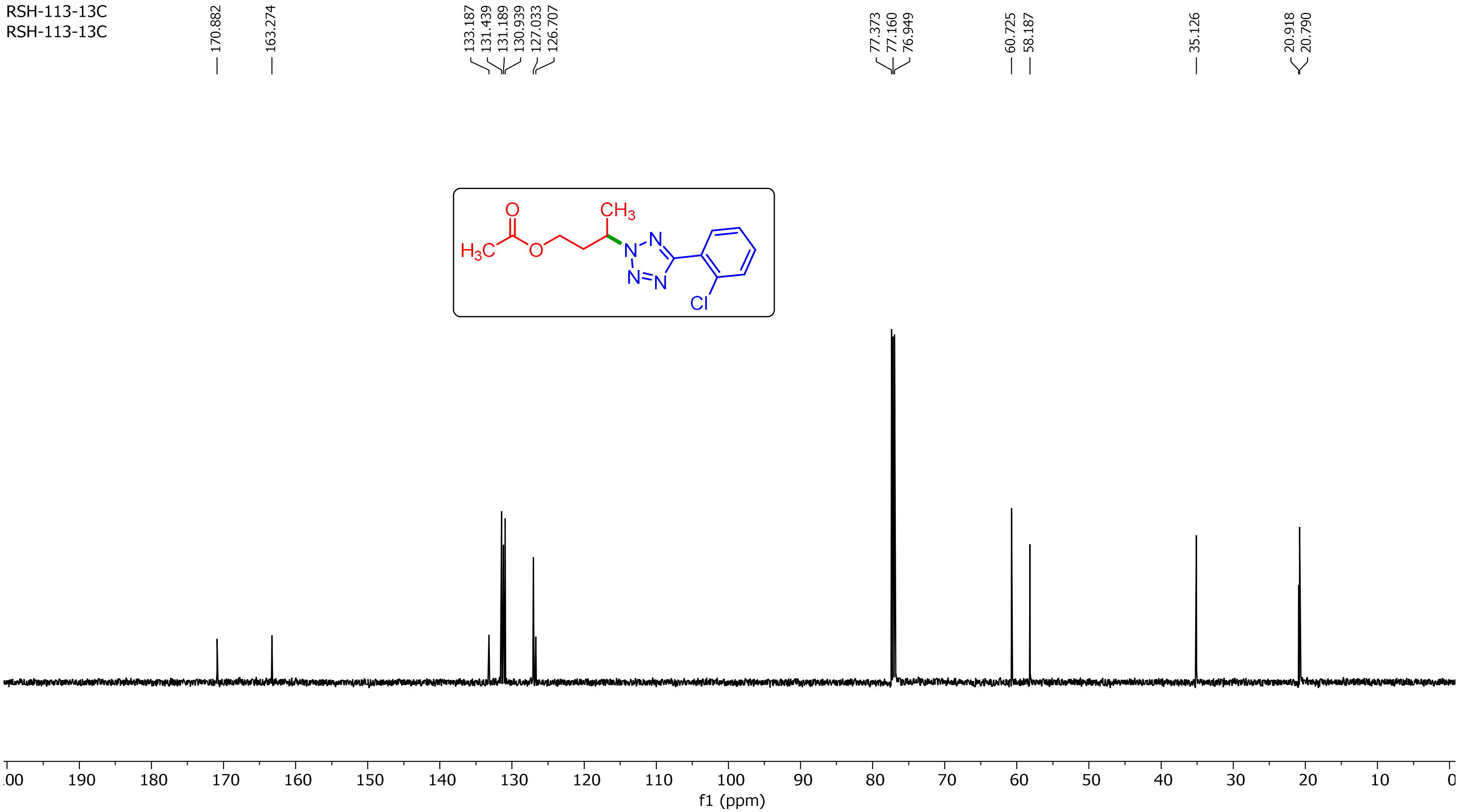

**3-(5-(2-Bromophenyl)-2*H*-tetrazol-2-yl)butyl acetate (1m): $^1$H NMR (600 MHz, $CDCl_3$)**

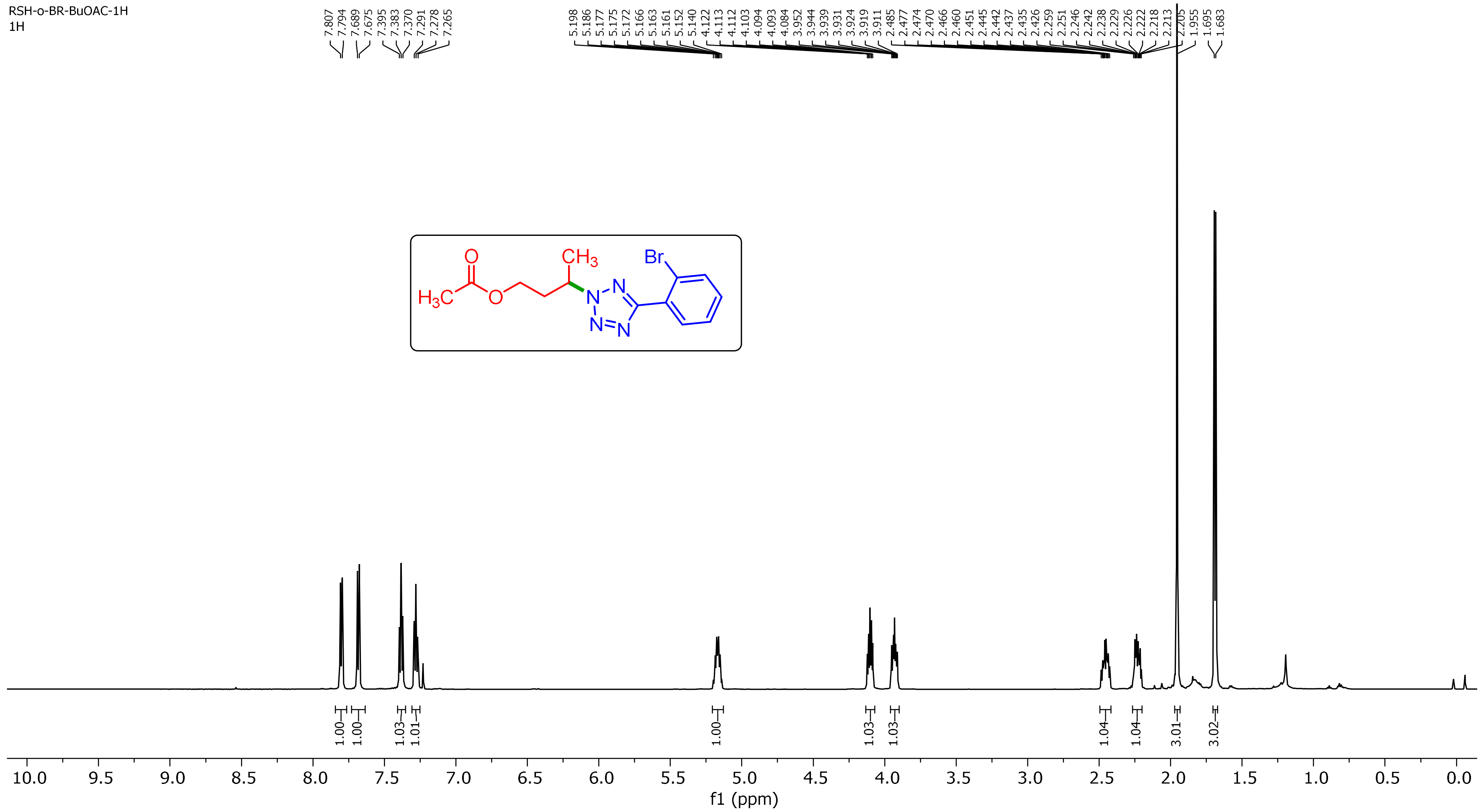

**3-(5-(2-Bromophenyl)-2*H*-tetrazol-2-yl)butyl acetate (1m): $^{13}$C NMR (151 MHz, CDCl$_3$)**

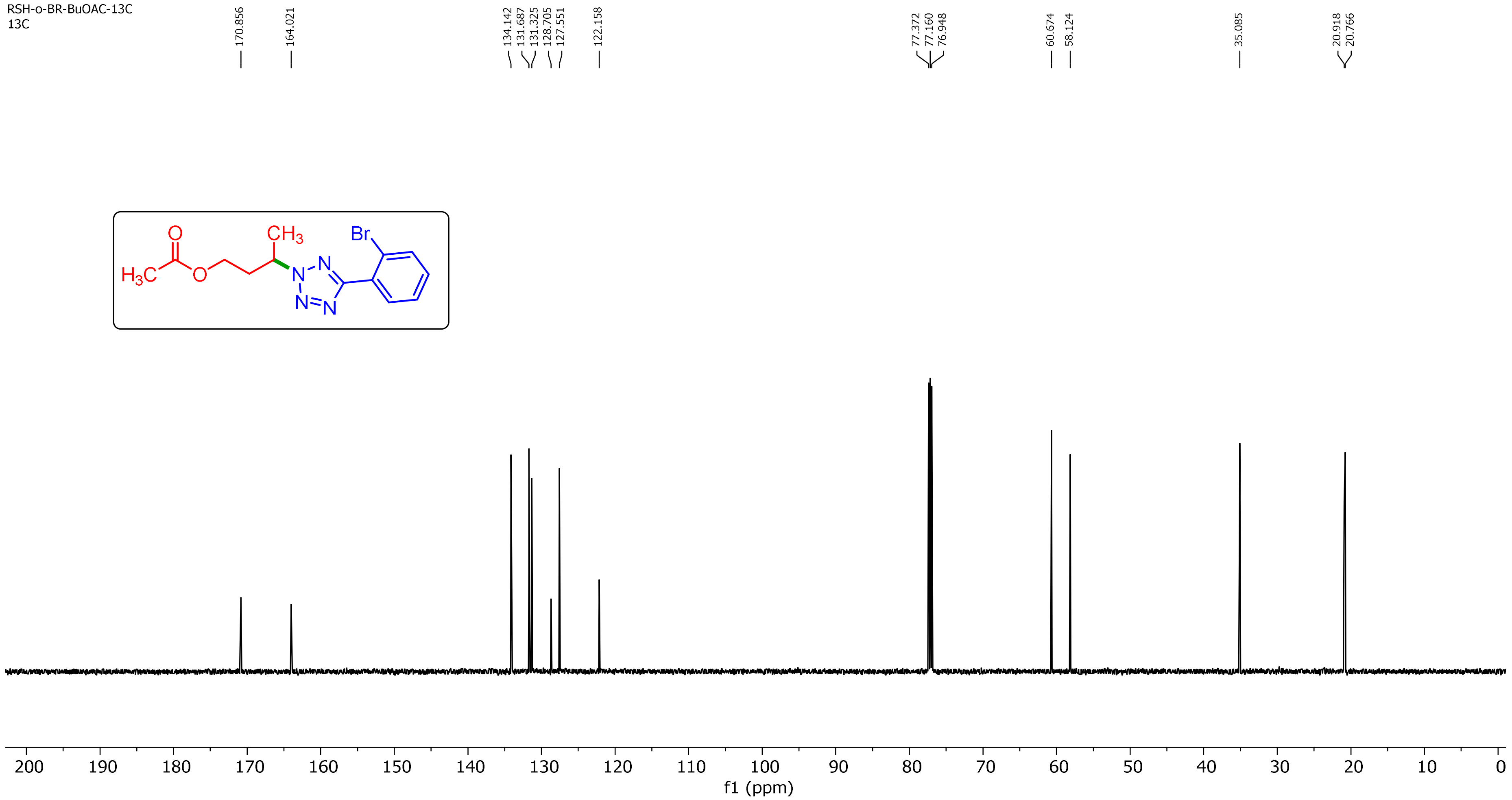

**(*E*)-3-(5-Styryl-2*H*-tetrazol-2-yl)butyl acetate (1n): $^{1}$H NMR (600 MHz, $CDCl_3$)**

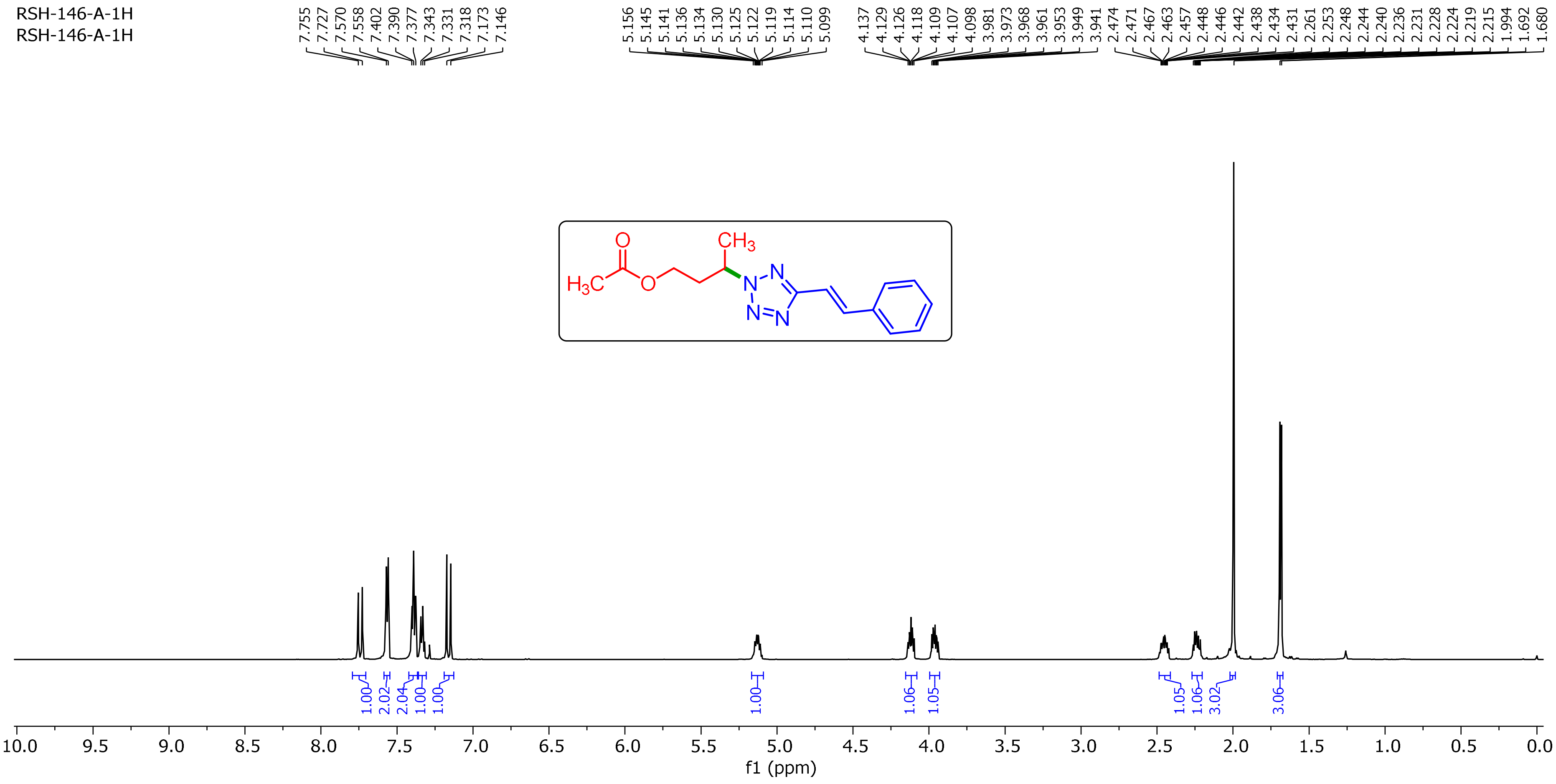

**(*E*)-3-(5-Styryl-2*H*-tetrazol-2-yl)butyl acetate (1n): $^{13}$C NMR (151 MHz, $CDCl_3$)**

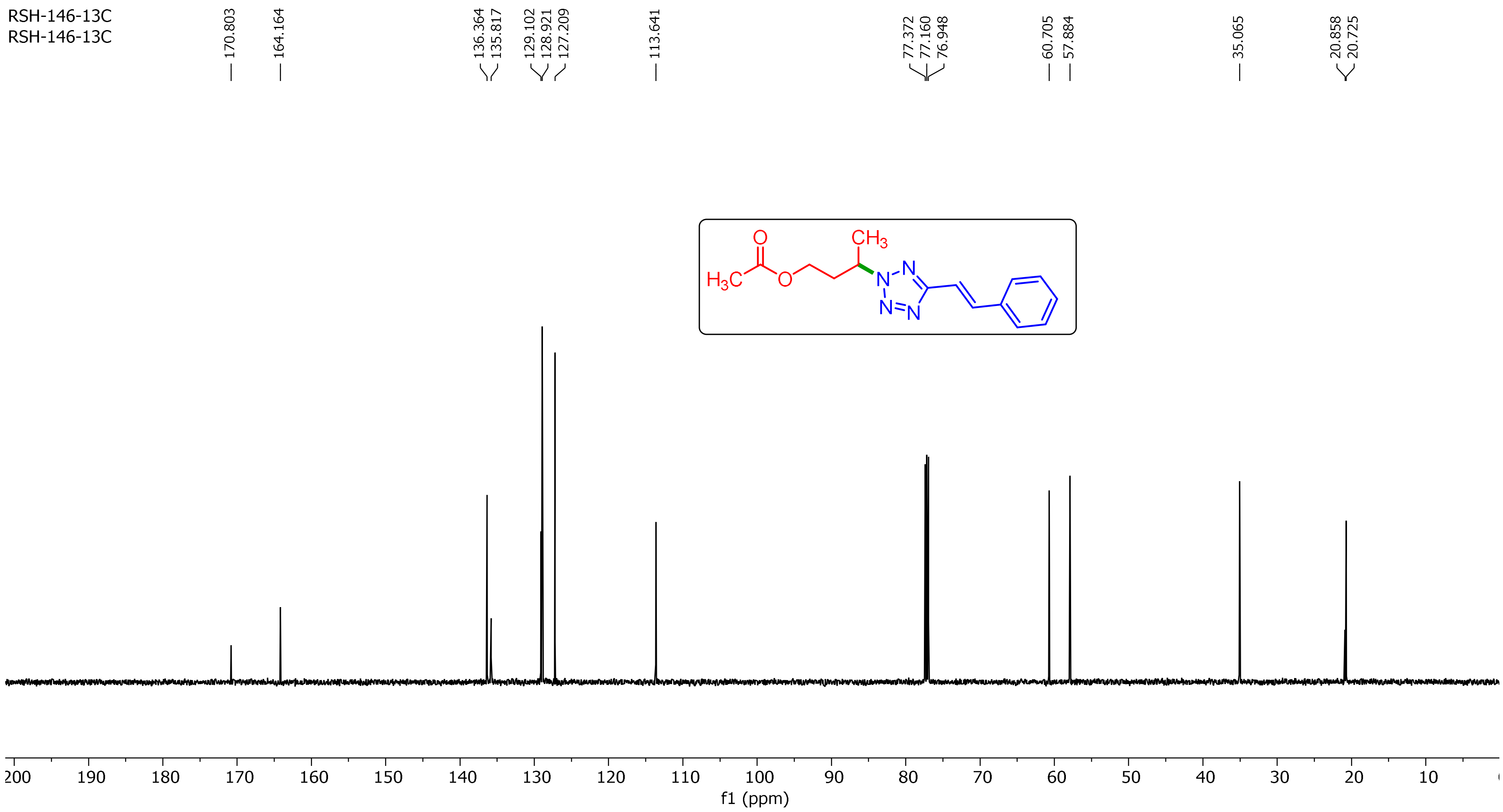

**3-(5-(2-Bromo-4-methylphenyl)-2*H*-tetrazol-2-yl)butyl acetate (1o): $^{1}$H NMR (600 MHz, CDCl$_3$)**

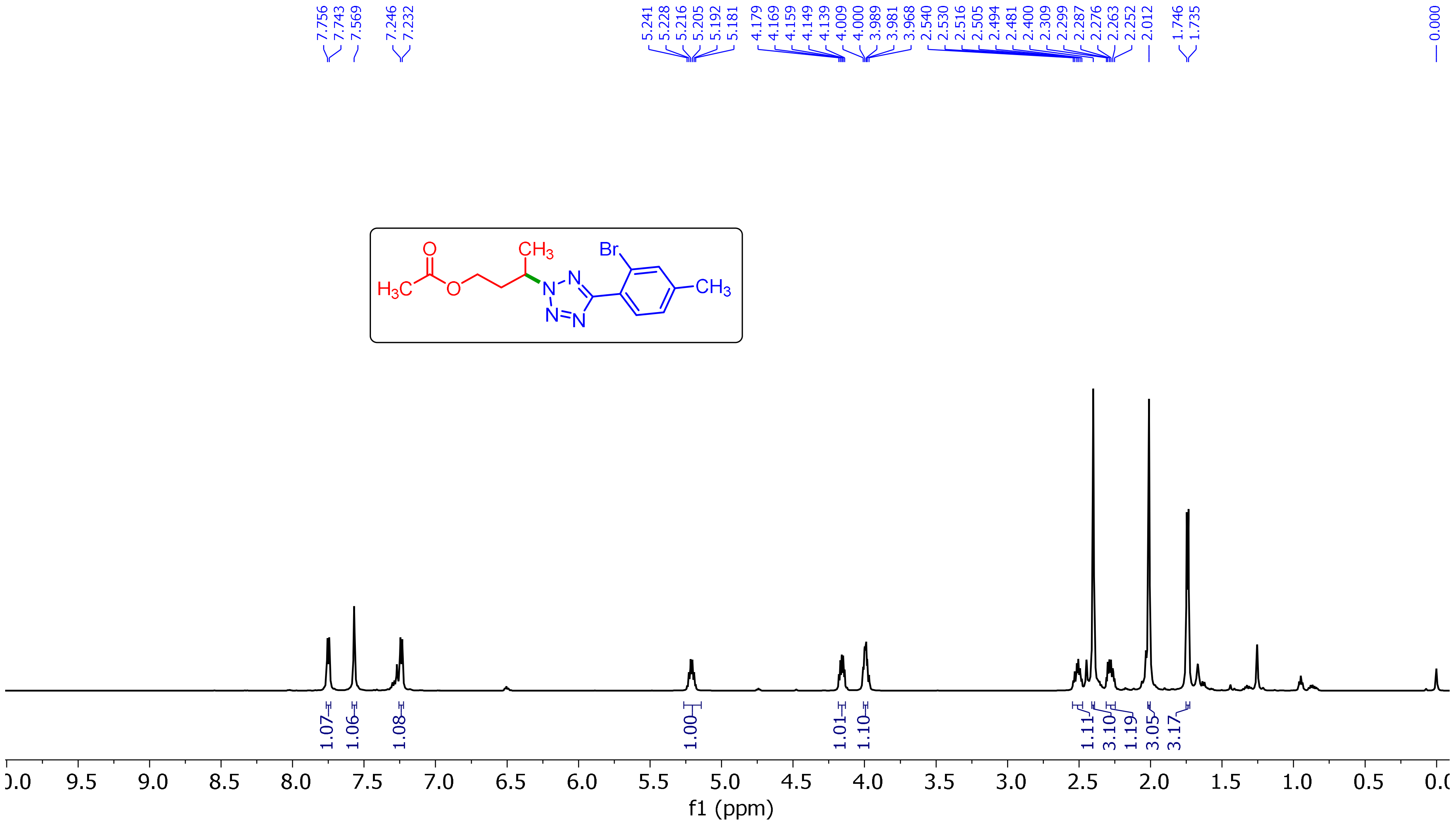

**3-(5-(2-Bromo-4-methylphenyl)-2*H*-tetrazol-2-yl)butyl acetate (1o): $^{13}$C NMR (151 MHz, $CDCl_3$)**

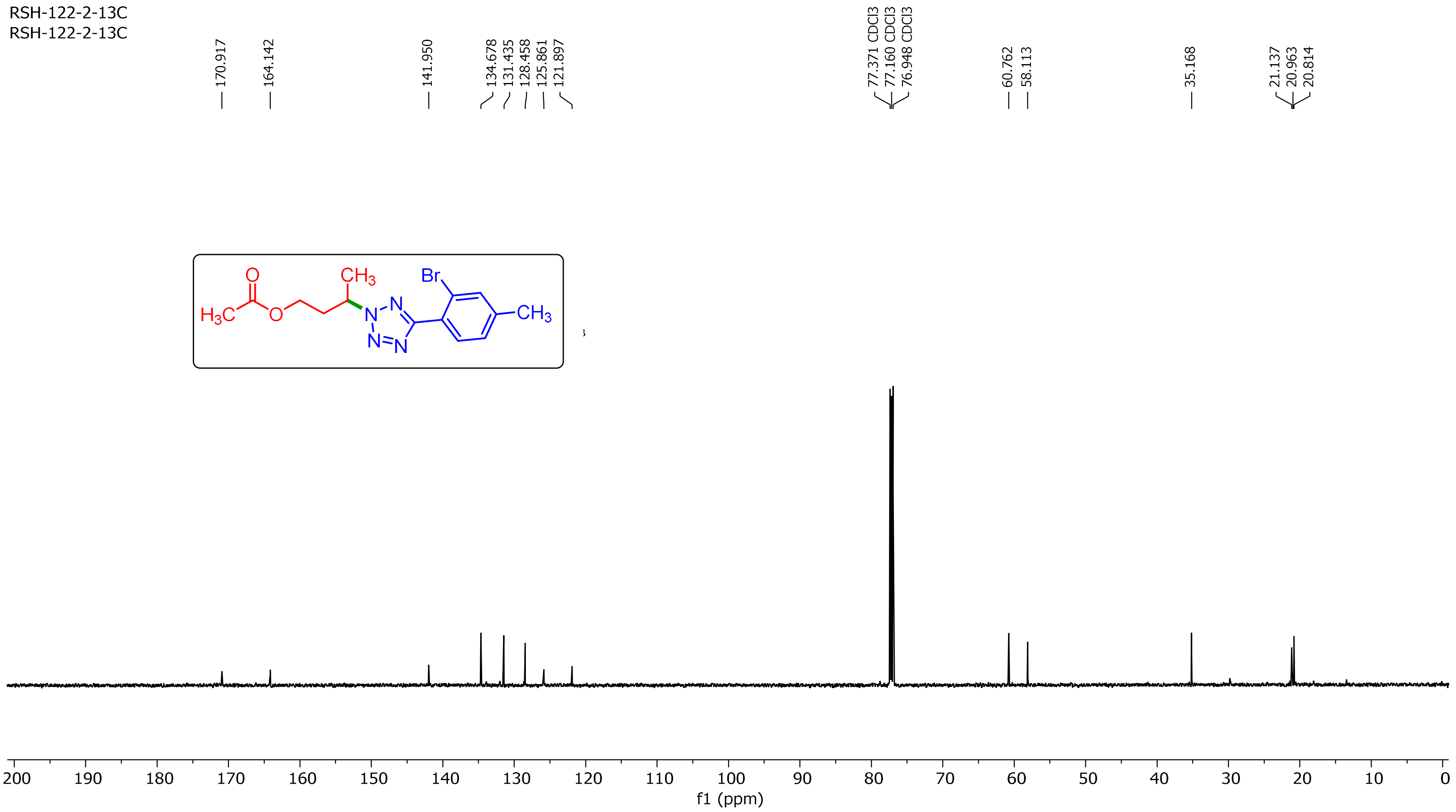

**3-(5-(3,4,5-Trimethoxyphenyl)-2*H*-tetrazol-2-yl)butyl acetate (1p): $^{1}$H NMR (600 MHz, CDCl3)**

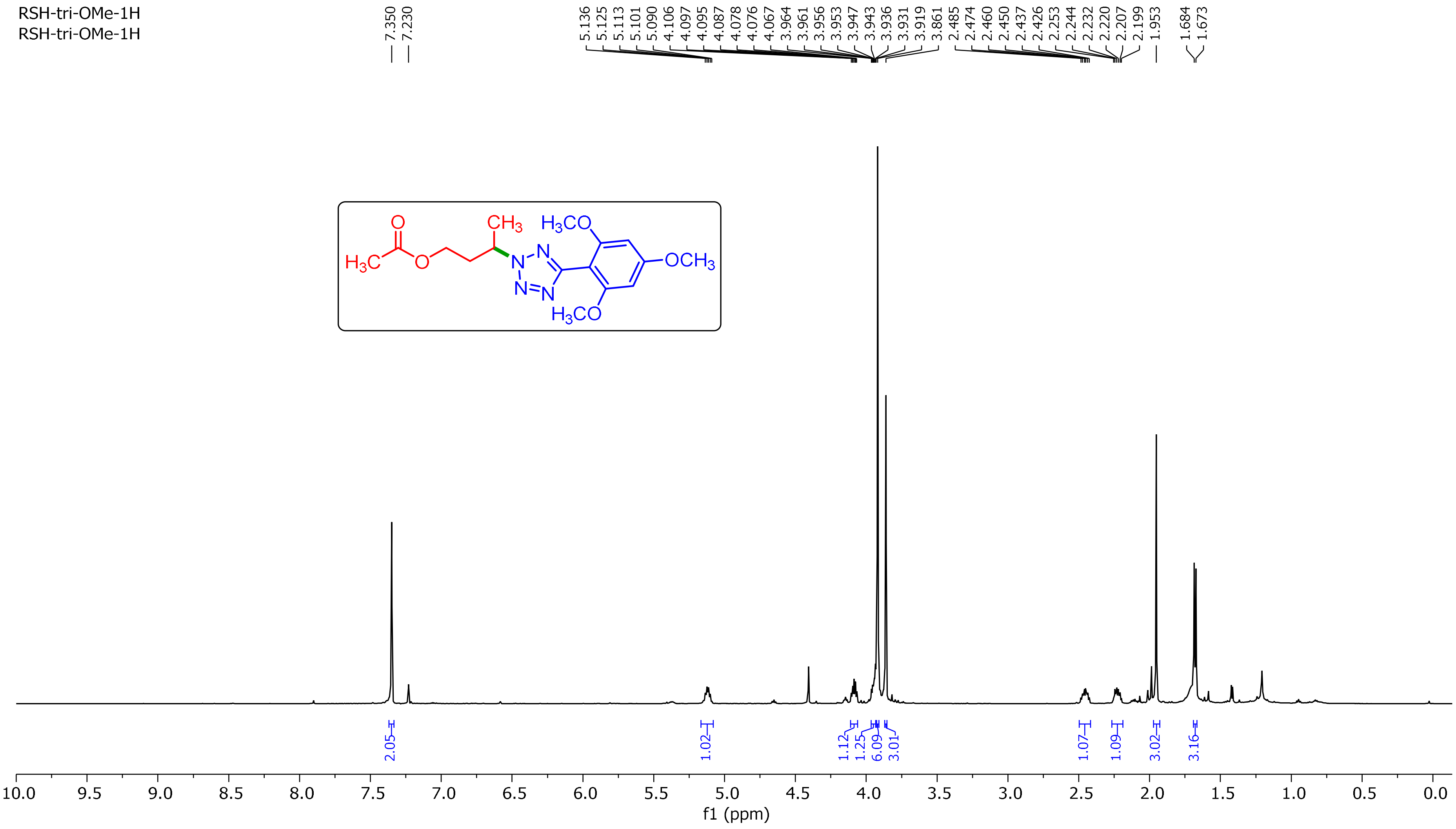

**3-(5-(3,4,5-Trimethoxyphenyl)-2*H*-tetrazol-2-yl)butyl acetate (1p): $^{13}$C NMR (151 MHz, $CDCl_3$)**

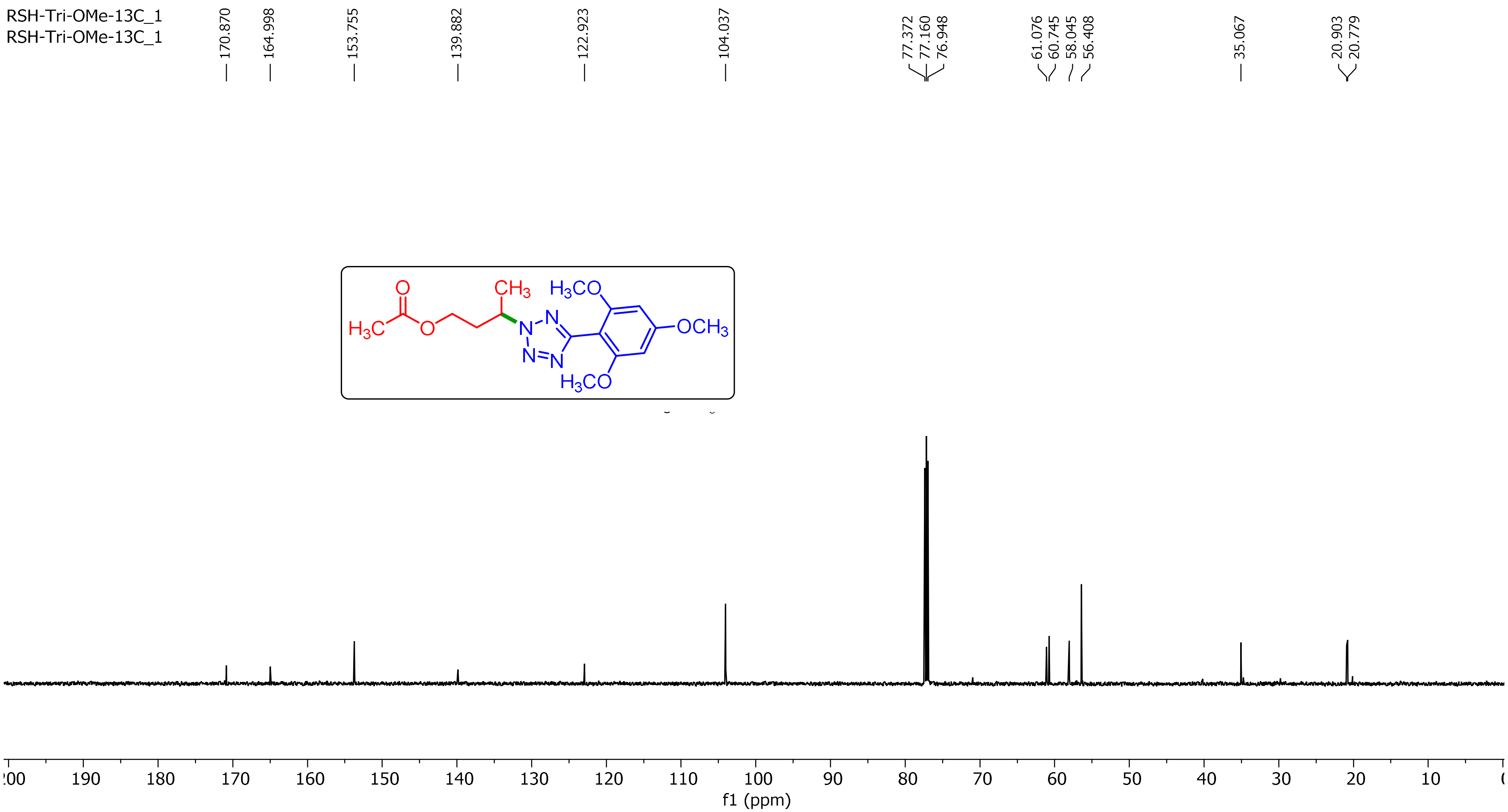

**3-(5-(Thiophen-2-yl)-2*H*-tetrazol-2-yl)butyl acetate (1q): $^{1}$H NMR (600 MHz, $CDCl_3$)**

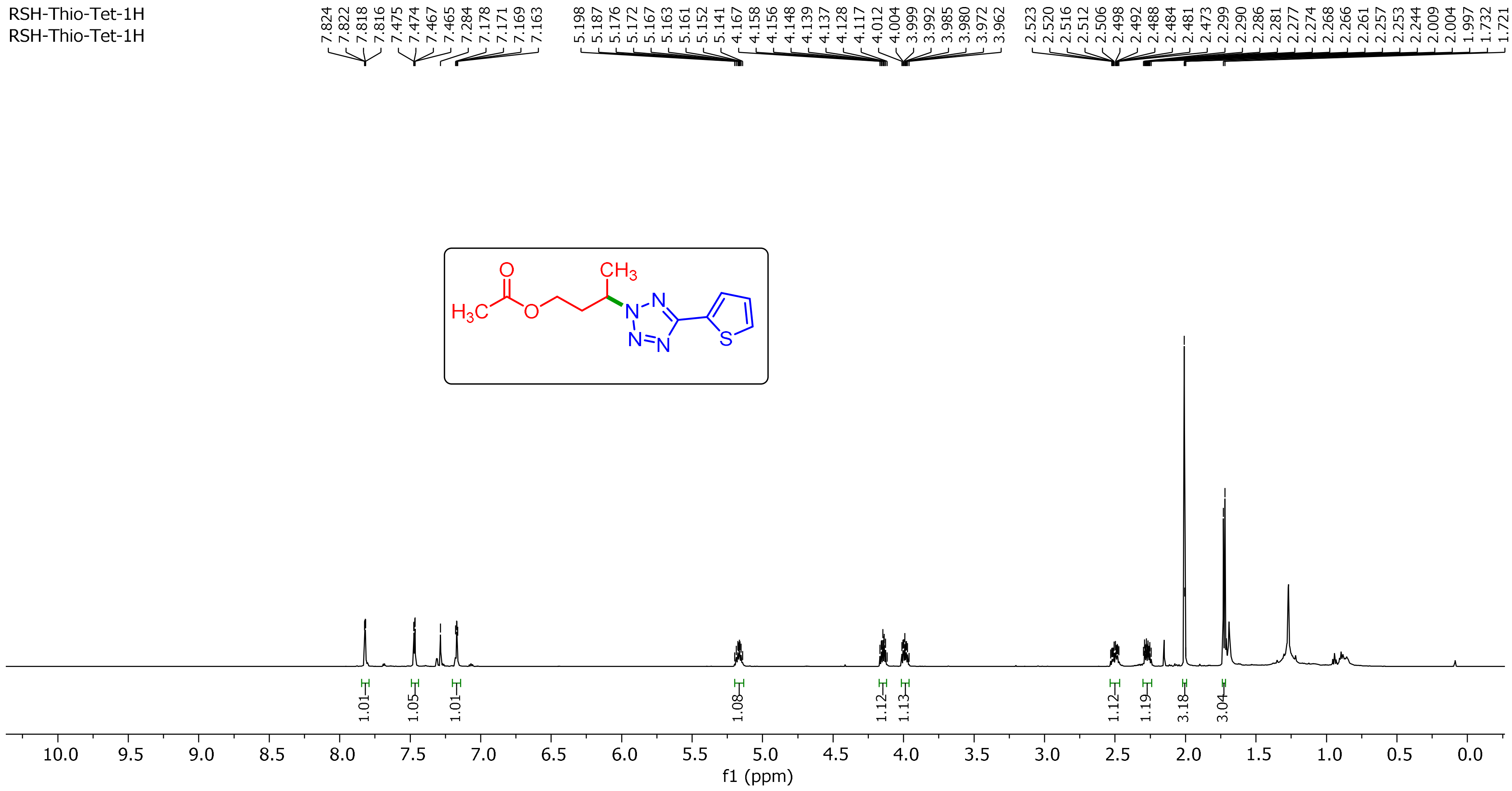

**3-(5-(Thiophen-2-yl)-2*H*-tetrazol-2-yl)butyl acetate (1q): $^{13}$C NMR (151 MHz, $CDCl_3$)**

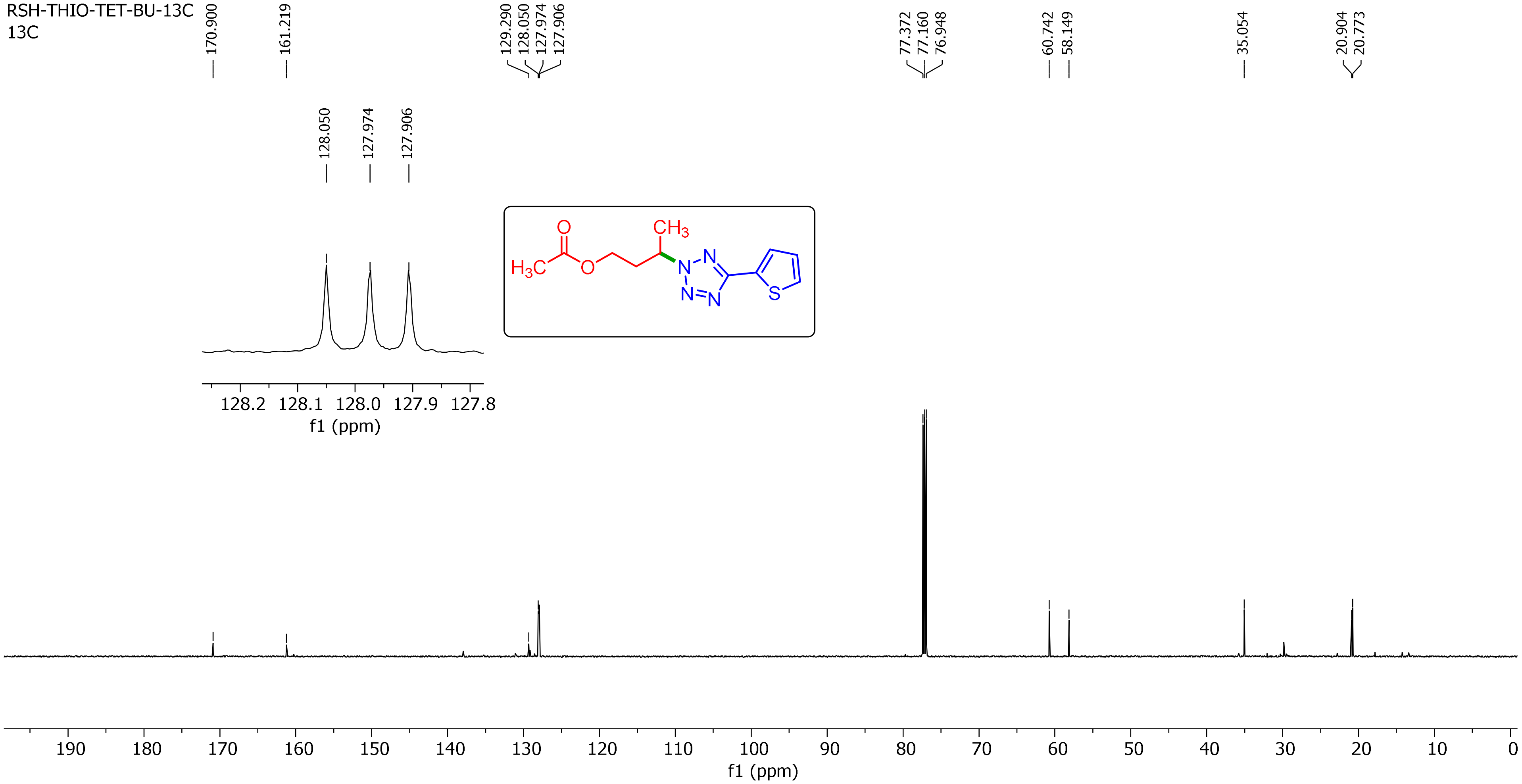

**3-(4-Phenyl-2*H*-1,2,3-triazol-2-yl)butyl acetate (1r): $^{1}$H NMR (600 MHz, $CDCl_3$)**

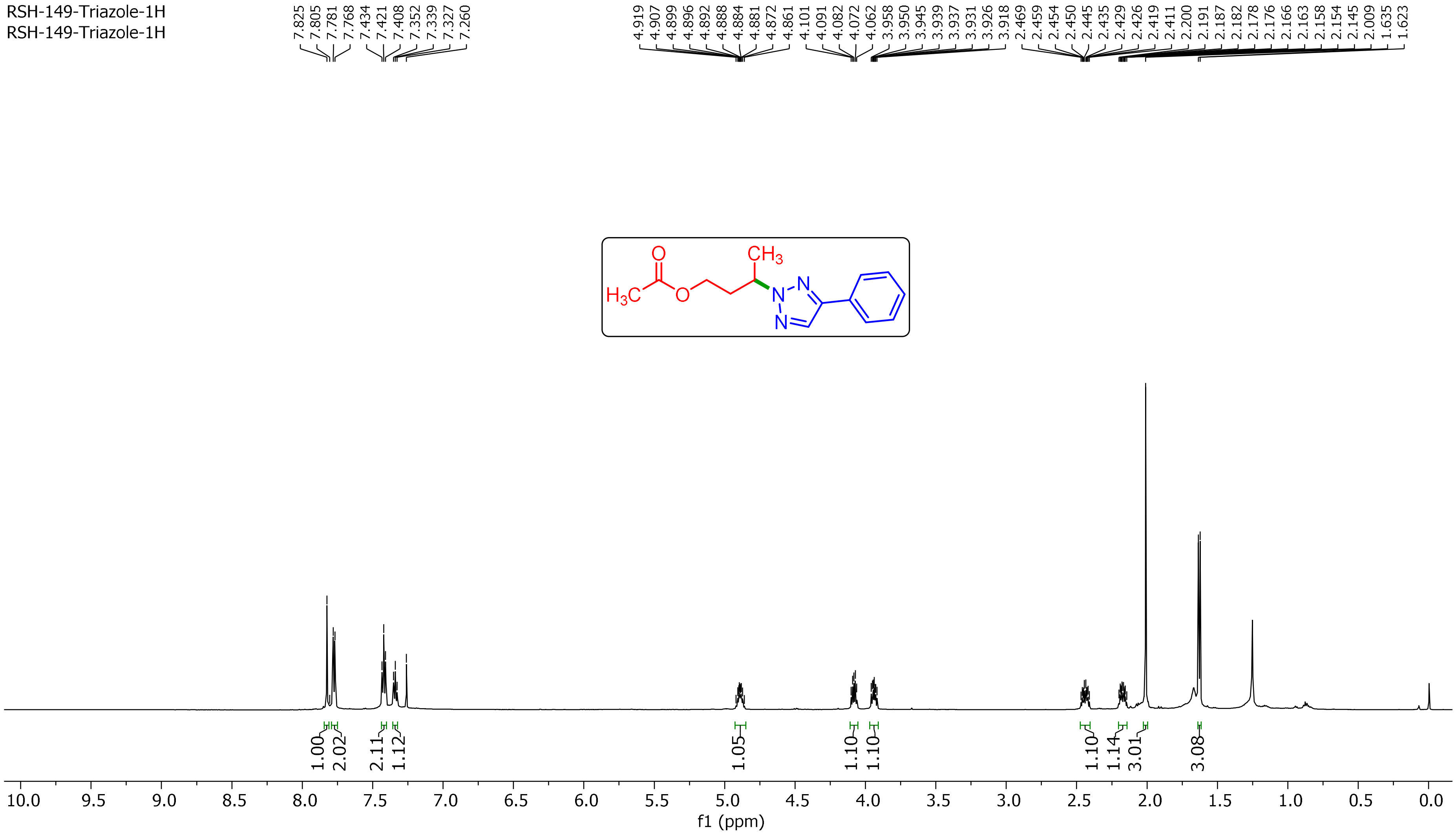

**3-(4-Phenyl-2*H*-1,2,3-triazol-2-yl)butyl acetate (1r): $^{13}$C NMR (151 MHz, CDCl$_3$)**

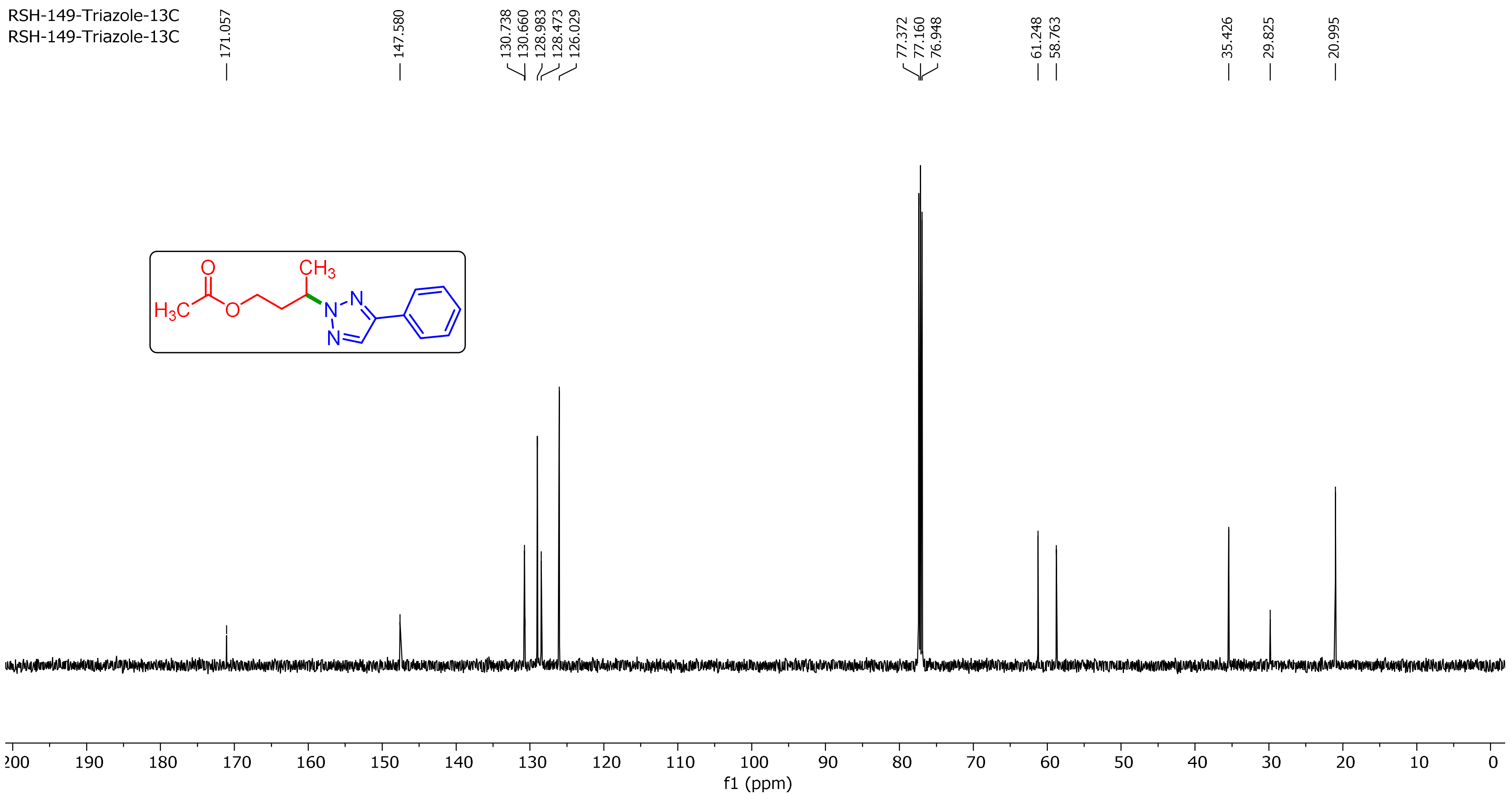

**3-(4-(4-(*tert*-Butyl)phenyl)-2*H*-1,2,3-triazol-2-yl)butyl acetate (1s): $^1$H NMR (600 MHz, CDCl$_3$)**

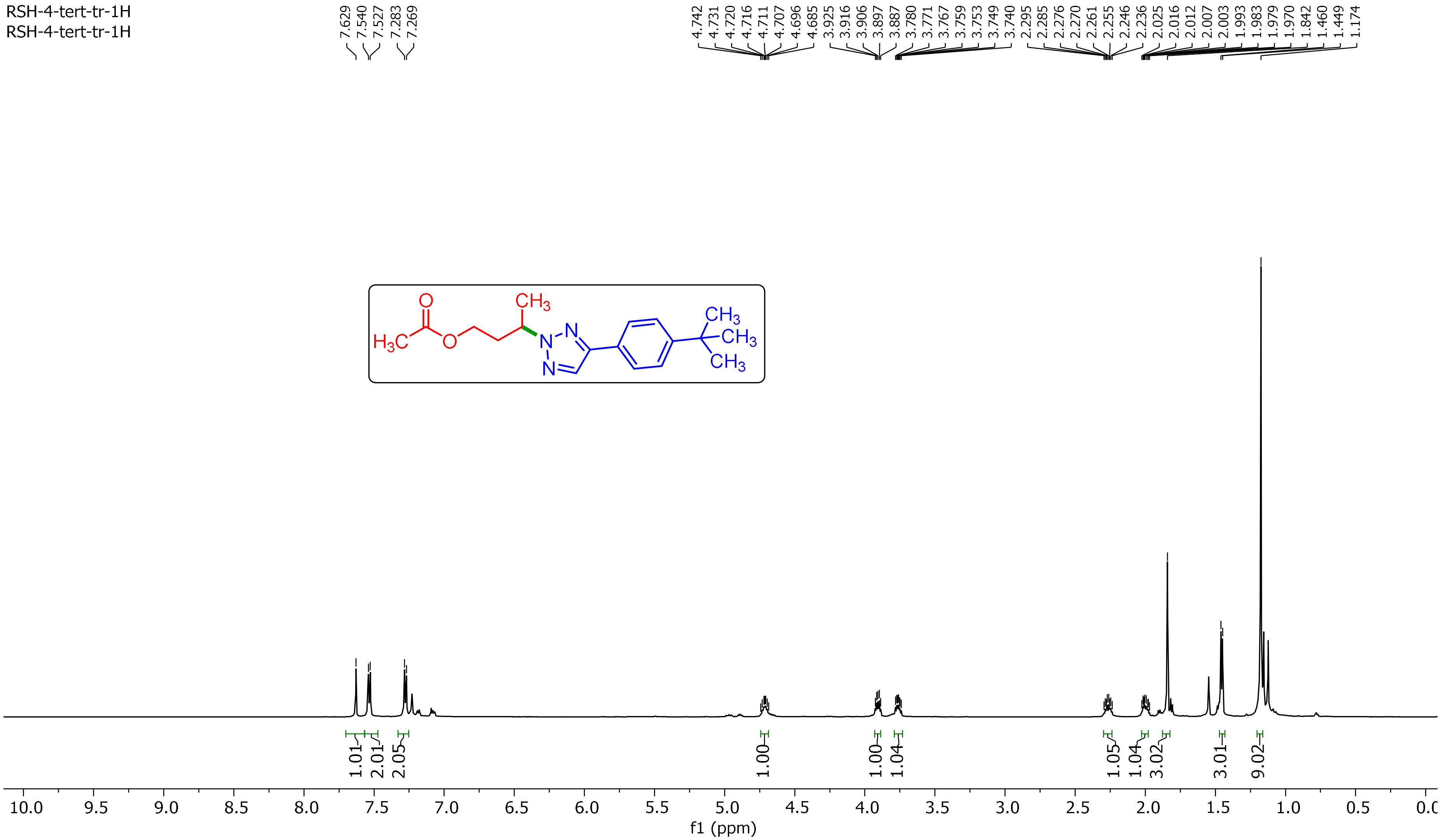

**3-(4-(4-(*tert*-Butyl)phenyl)-2*H*-1,2,3-triazol-2-yl)butyl acetate (1s): $^{13}$C NMR (151 MHz, $CDCl_3$)**

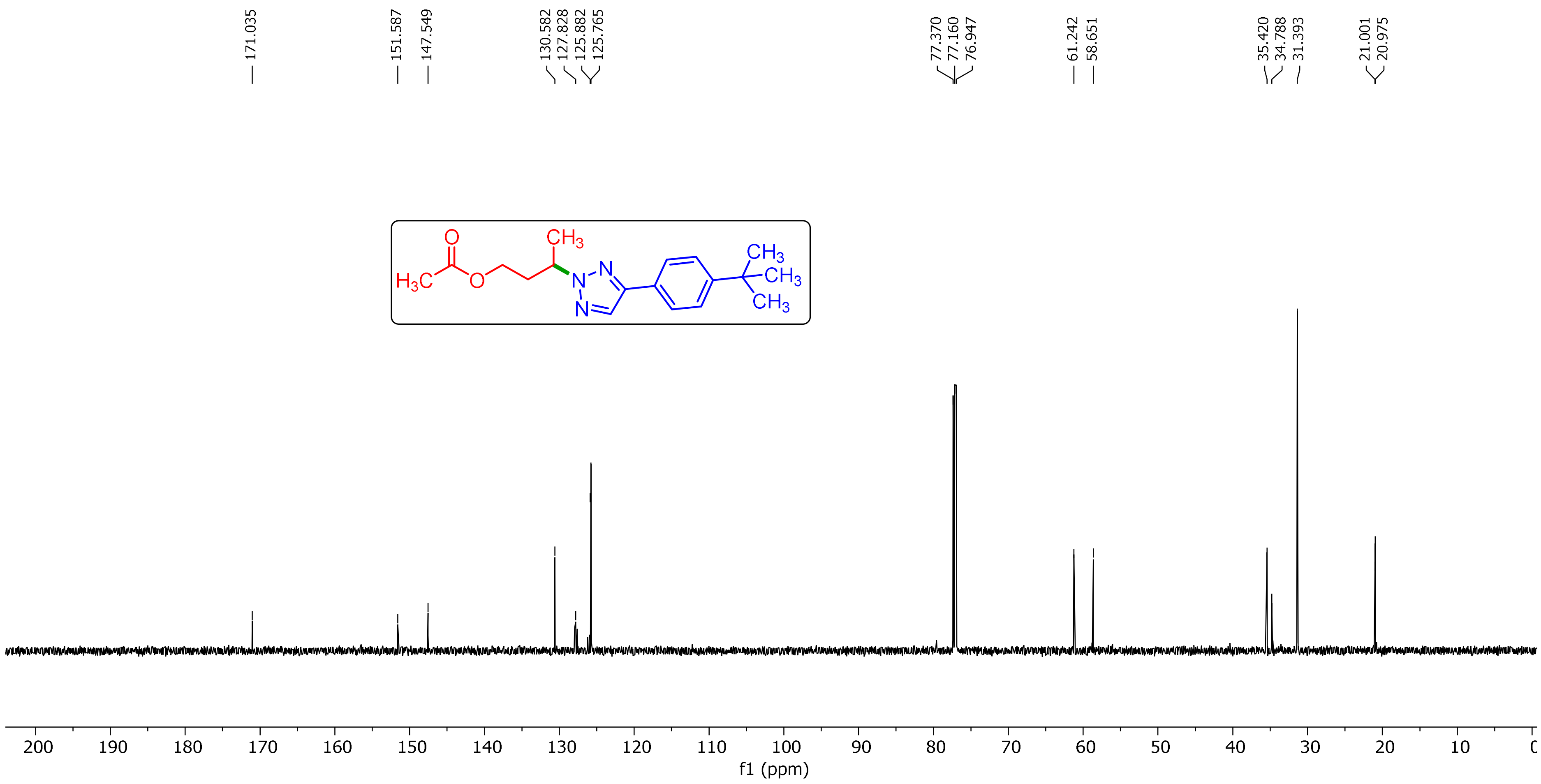

**3-(4-(4-Chlorophenyl)-2*H*-1,2,3-triazol-2-yl)butyl acetate (1t): $^{1}$H NMR (600 MHz, $CDCl_3$)**

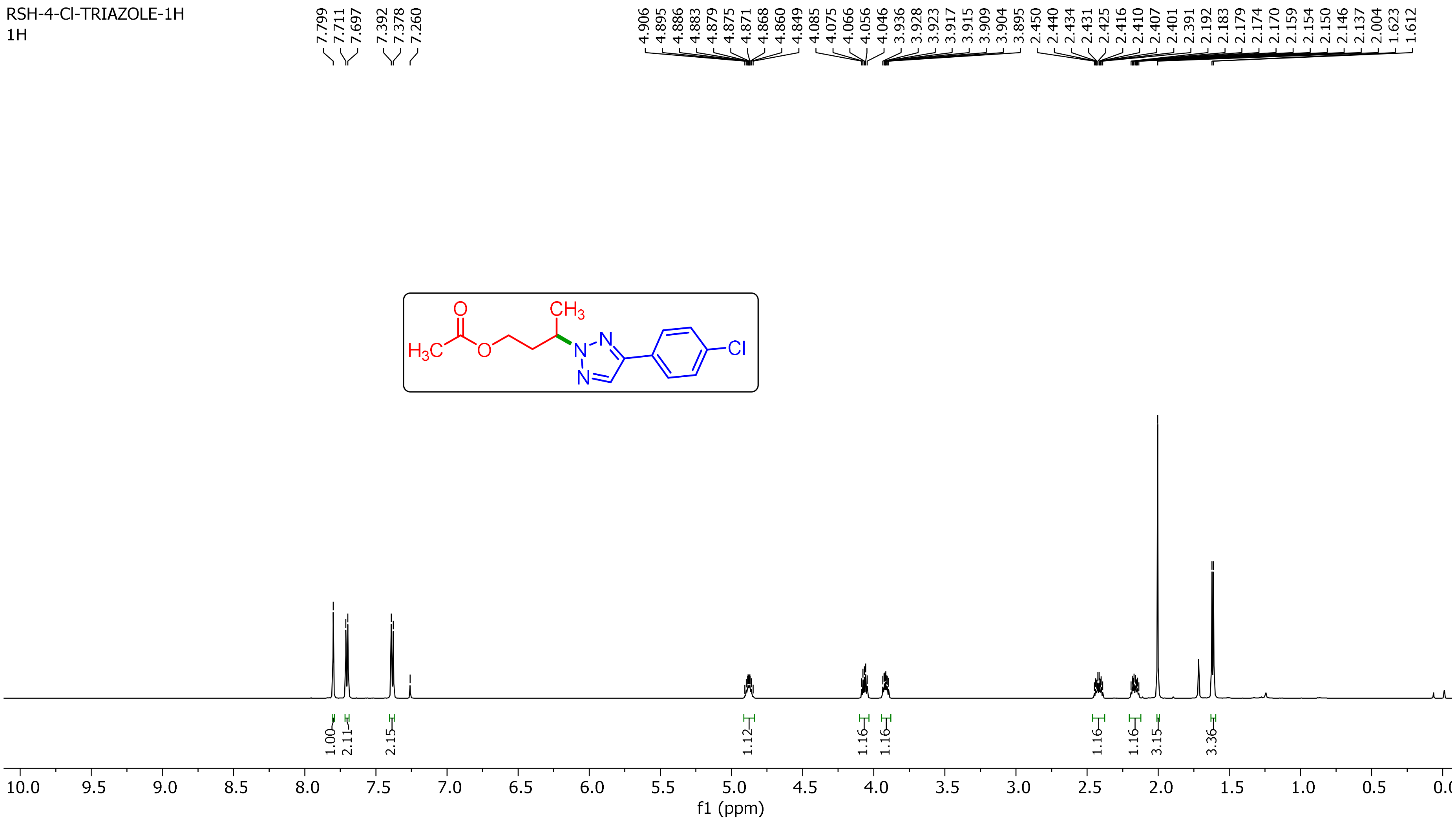

**3-(4-(4-Chlorophenyl)-2*H*-1,2,3-triazol-2-yl)butyl acetate (1t): $^{13}$C NMR (151 MHz, CDCl$_3$)**

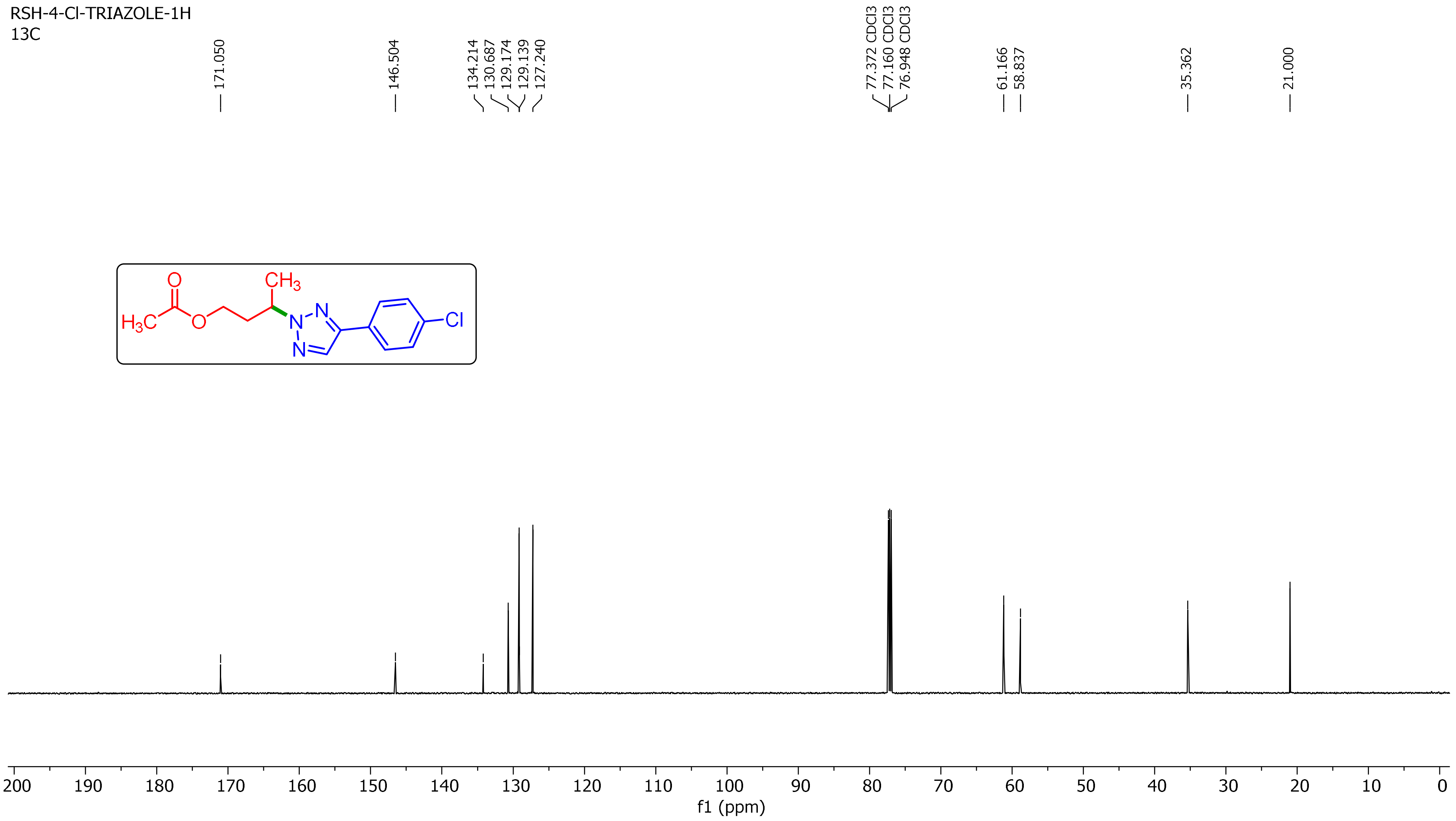

**3-(1,1-Dioxido-3-oxobenzo[*d*]isothiazol-2(3*H*)-yl)butyl acetate (1u): $^{1}$H NMR (500 MHz, $CDCl_3$)**

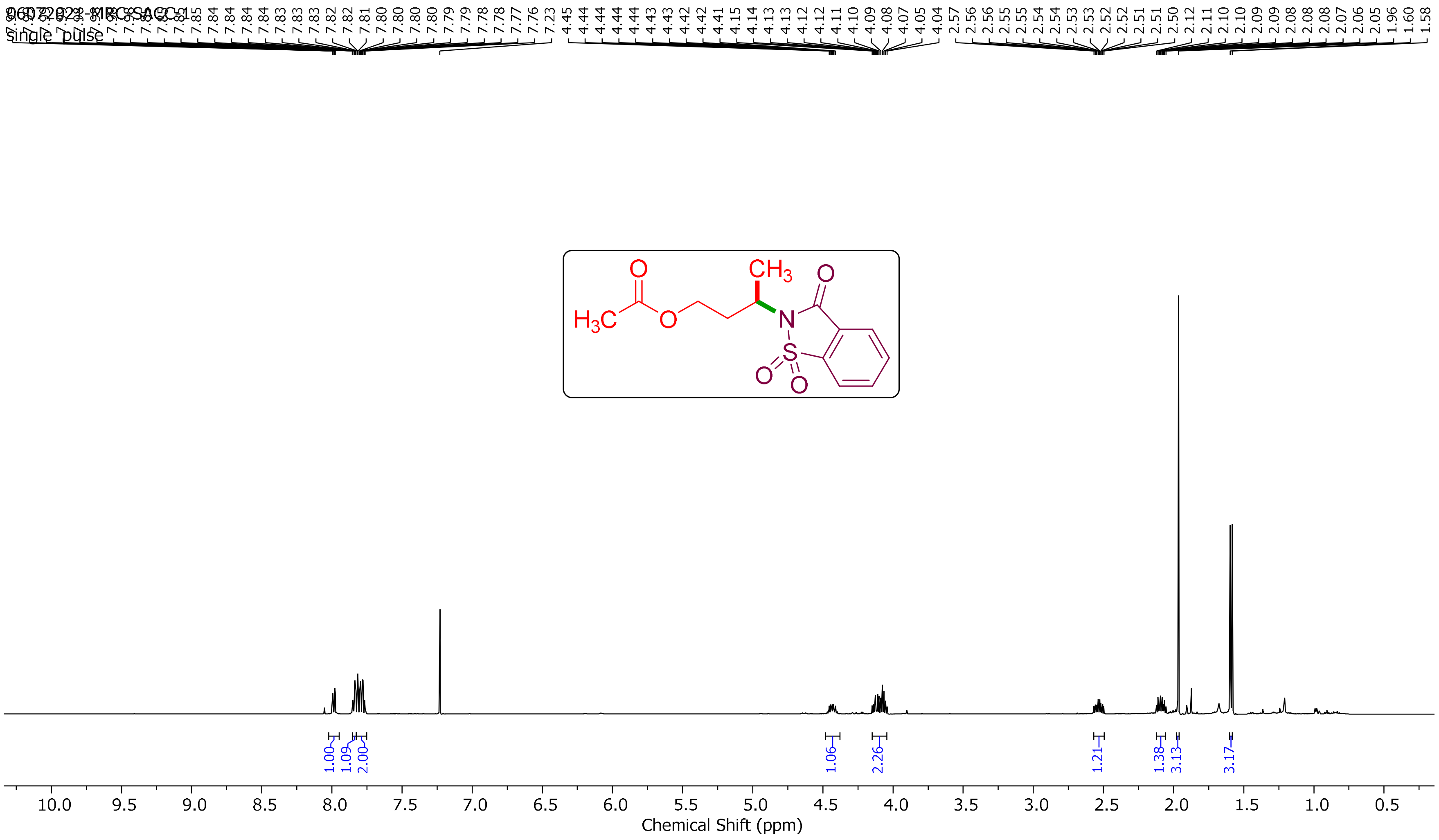

**3-(1,1-Dioxido-3-oxobenzo[*d*]isothiazol-2(3*H*)-yl)butyl acetate (1u): $^{1}$H NMR (500 MHz, $CDCl_3$)**

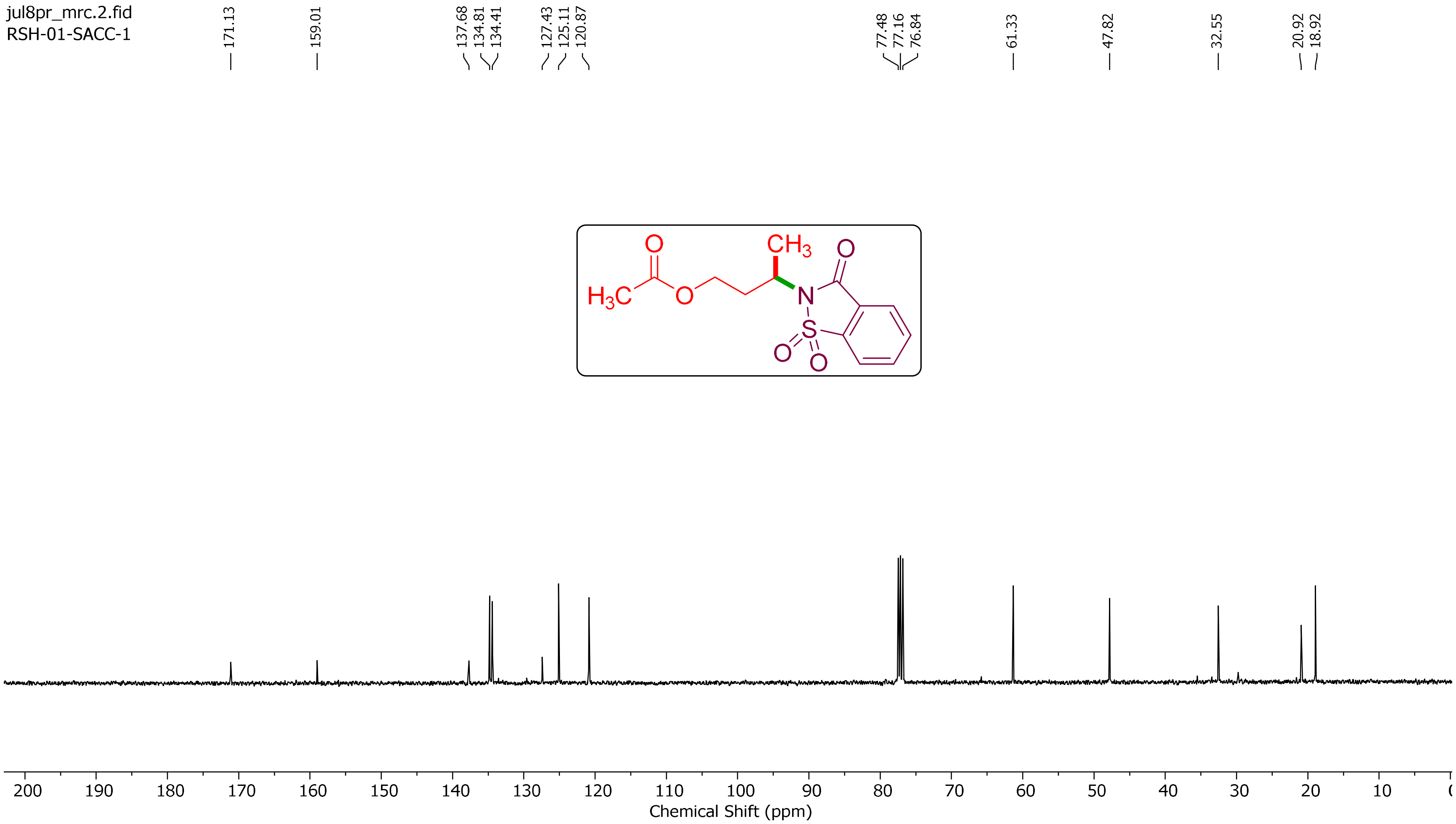

**Indolo[*2,1-b*]quinazoline-6,12-dione (X): $^{1}$H NMR (600 MHz, $CDCl_3$)**

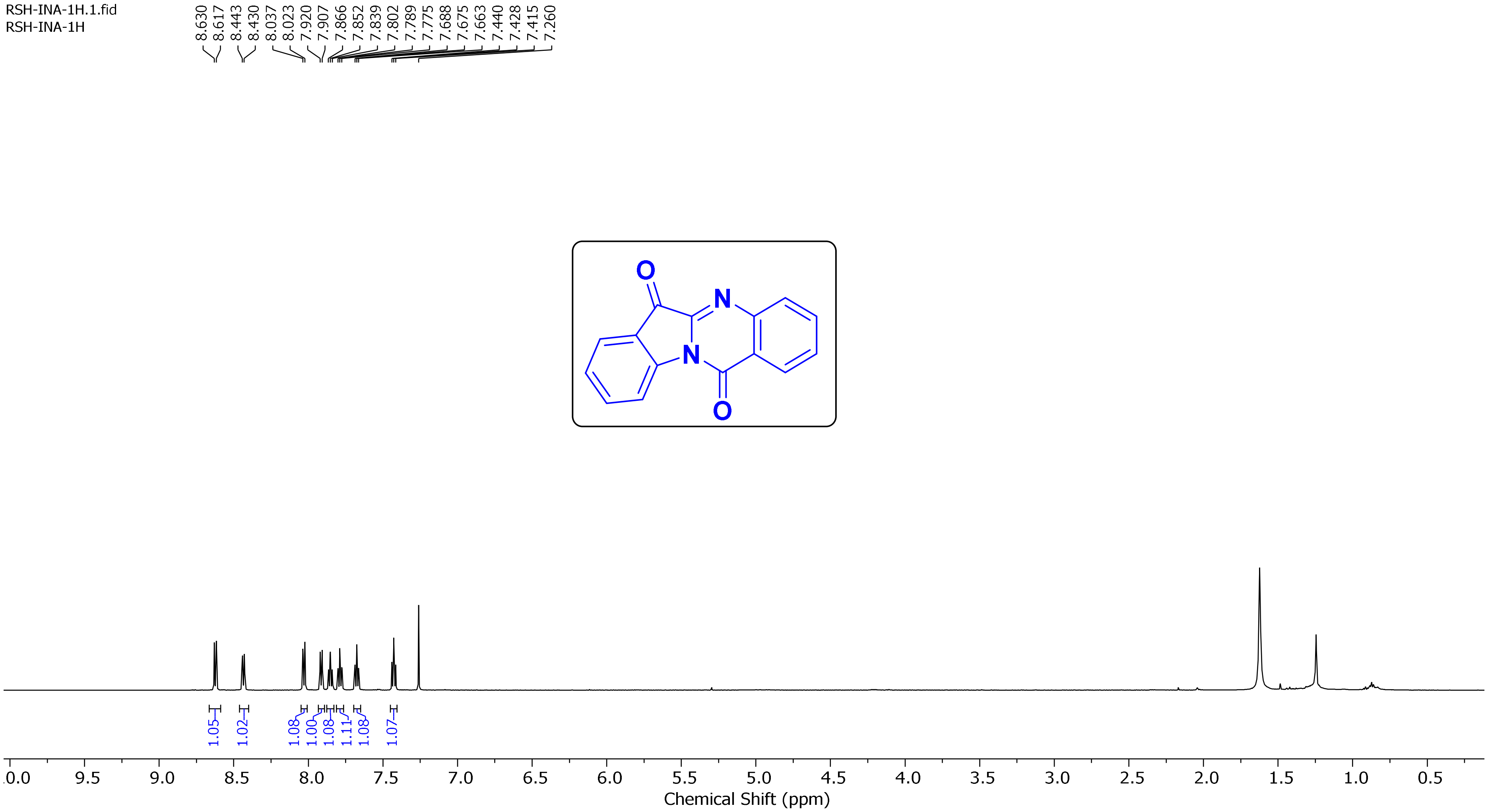

**Indolo[*2,1-b*]quinazoline-6,12-dione (X): $^{13}$C NMR (600 MHz, $CDCl_3$)**

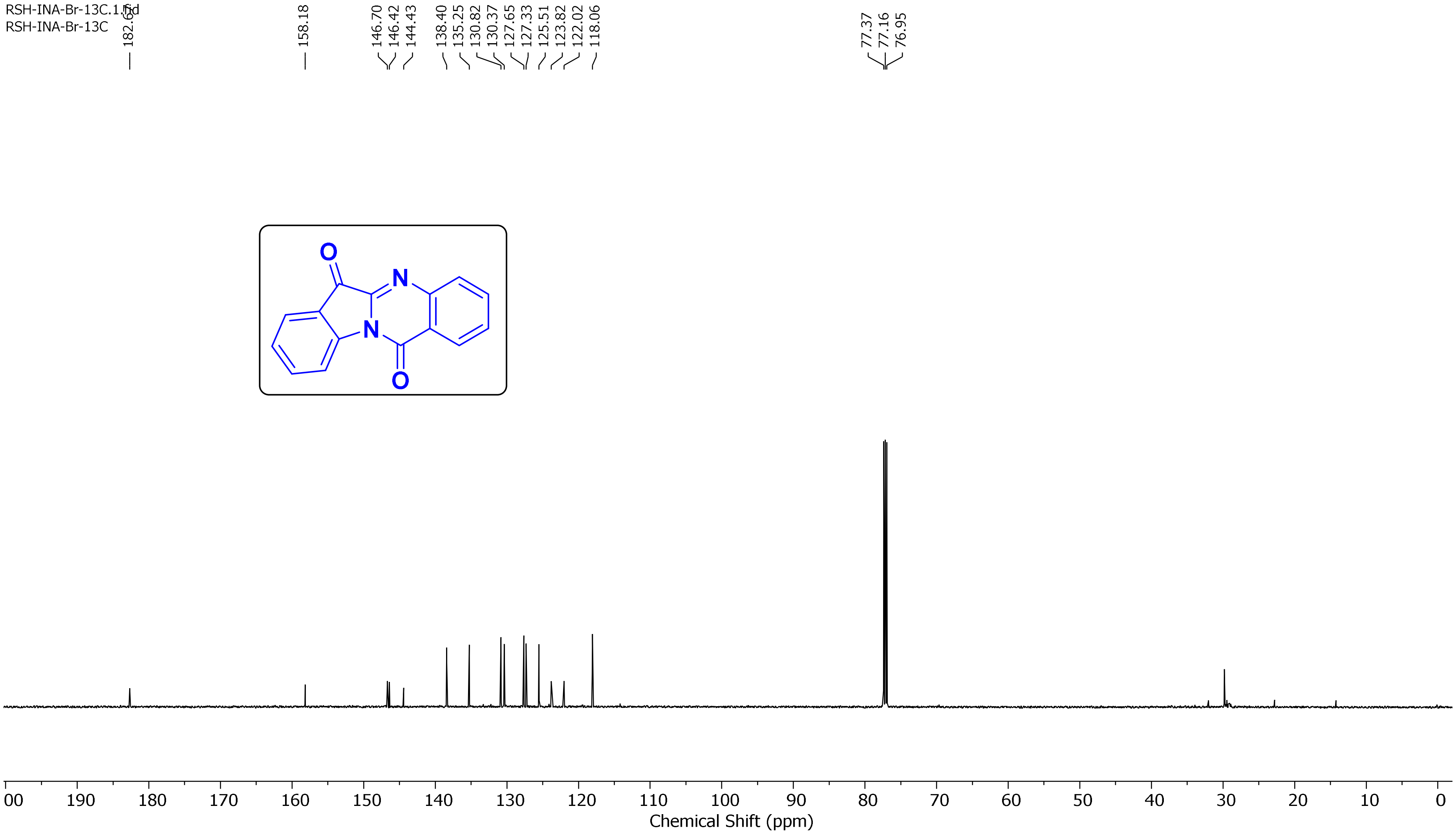

**3-(5-Phenyl-2*H*-tetrazol-2-yl)butyl benzoate (2a): $^1$H NMR (400 MHz, $CDCl_3$)**

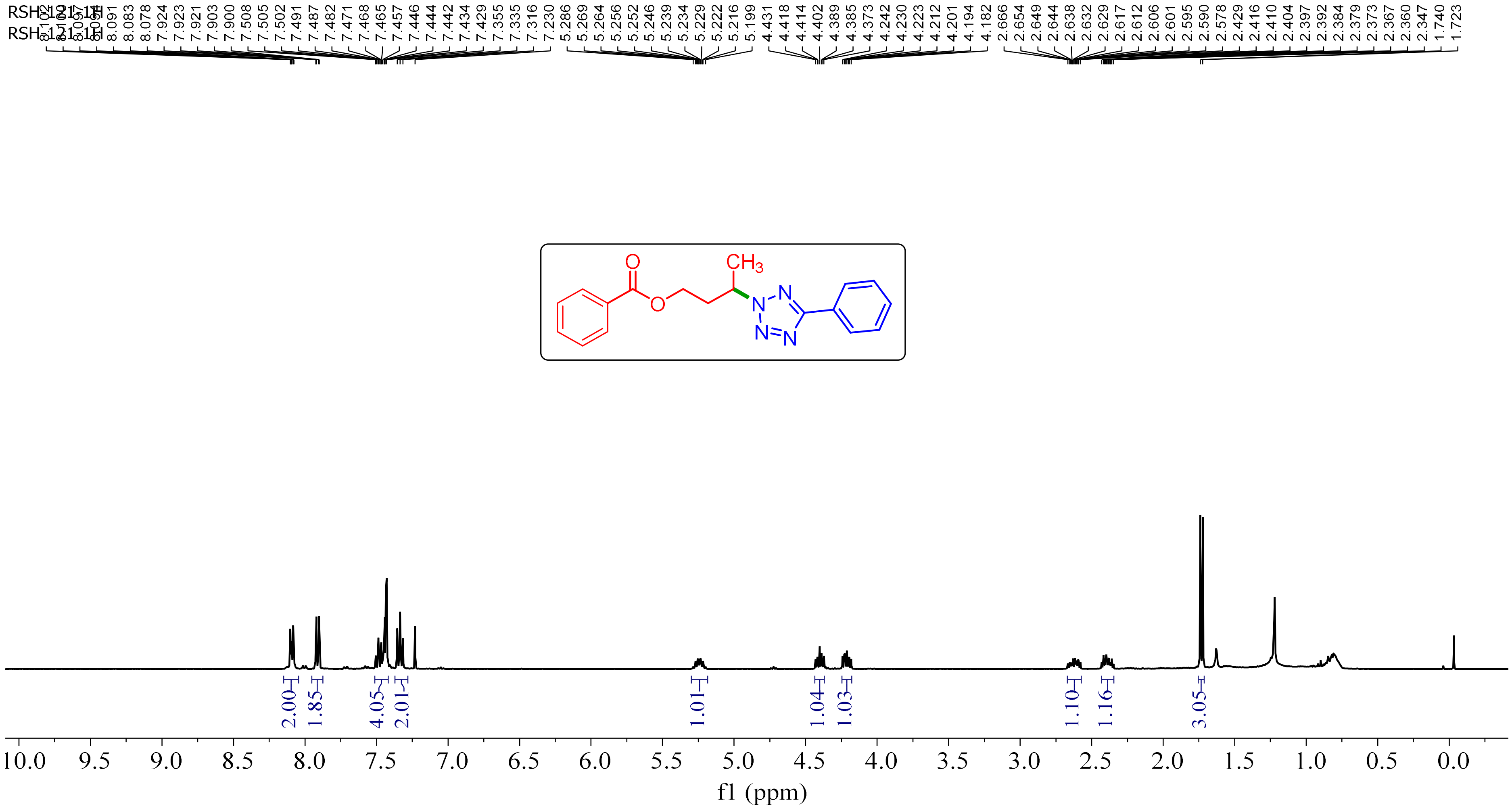

**3-(5-Phenyl-2*H*-tetrazol-2-yl)butyl benzoate (2a): $^{13}$C NMR (151 MHz, CDCl$_3$)**

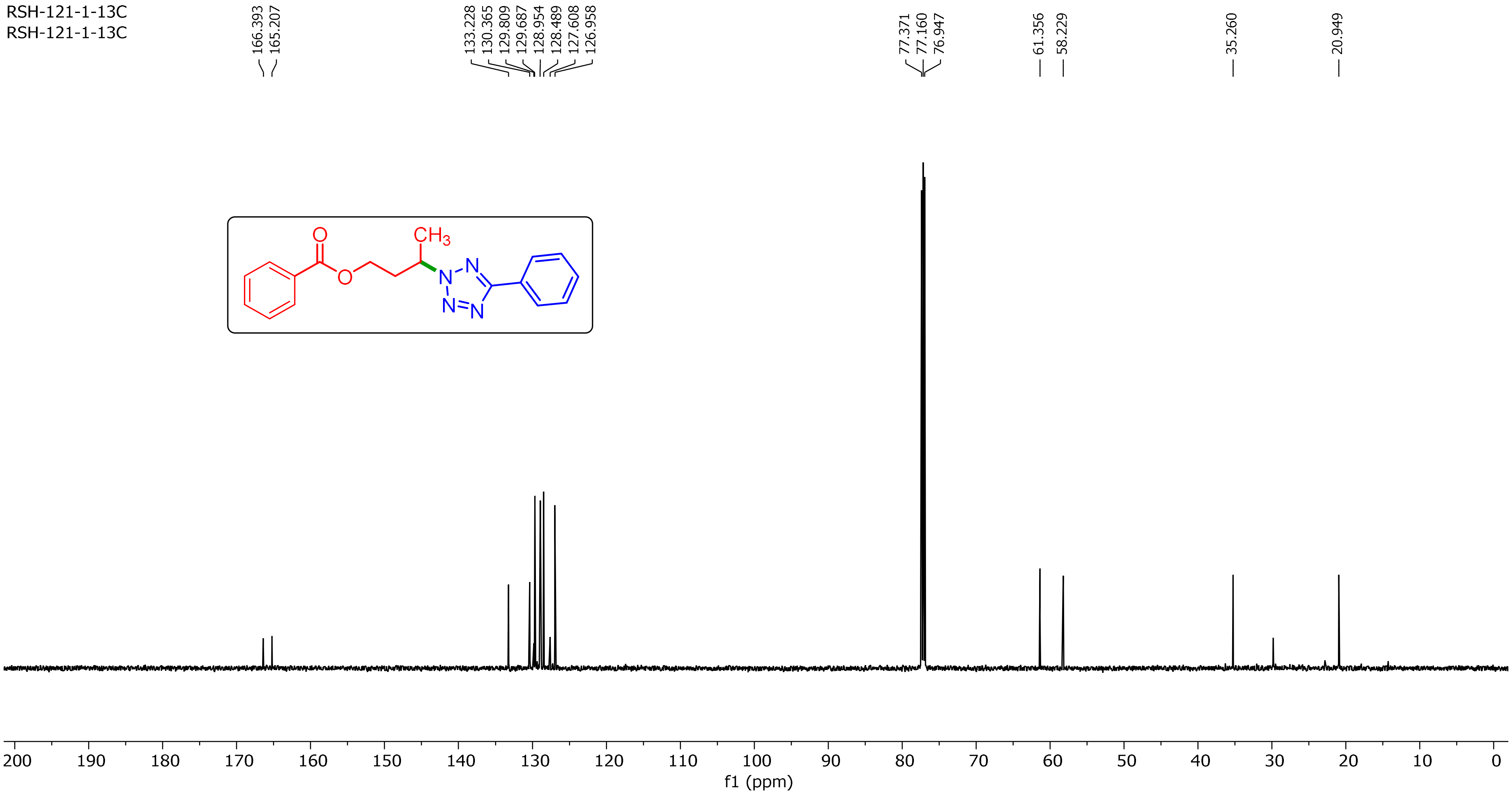

**3-Phenyl-3-(5-phenyl-2*H*-tetrazol-2-yl)propyl acetate (3a): $^{1}$H NMR (600 MHz, $CDCl_3$)**

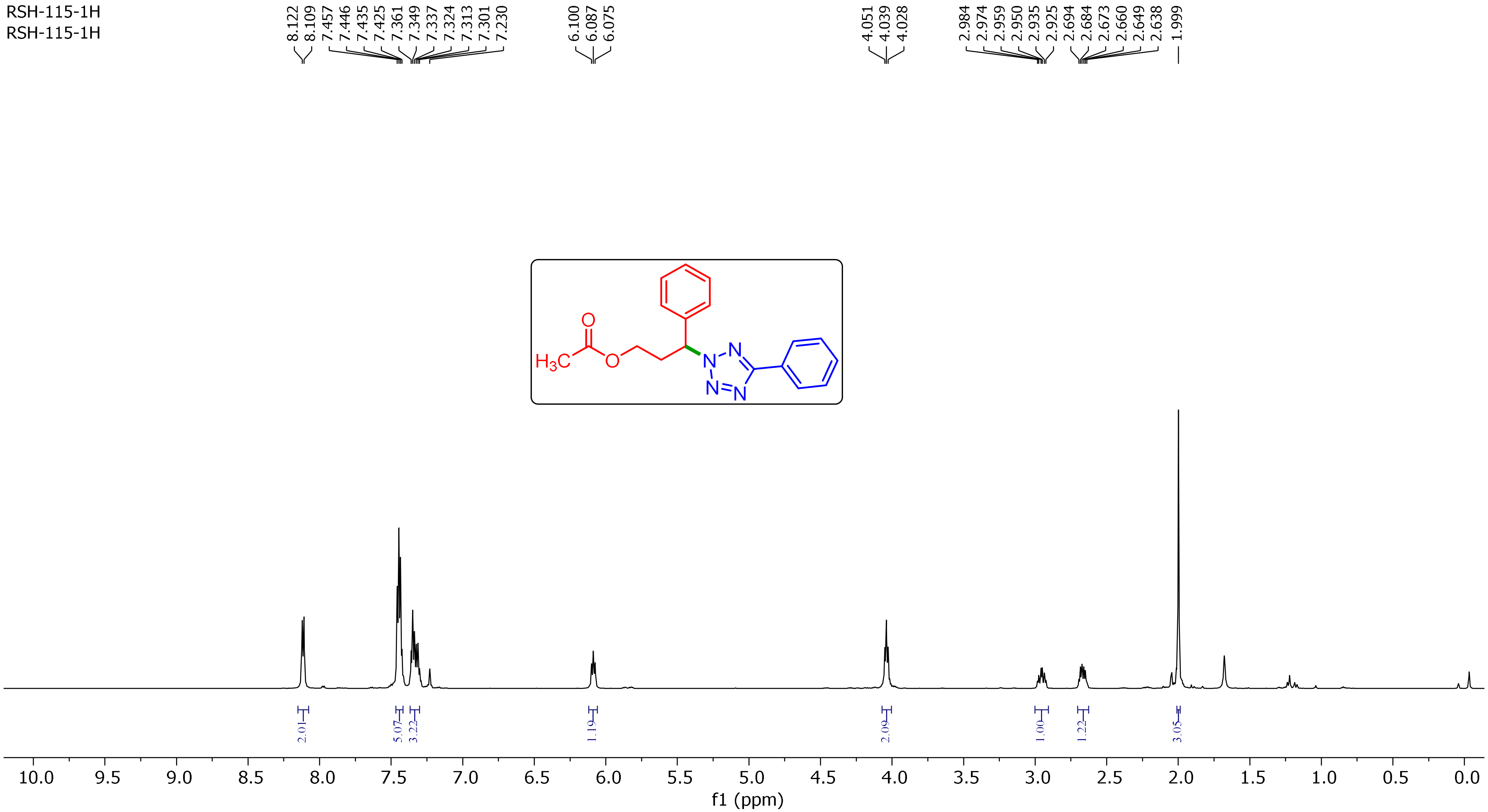

**3-Phenyl-3-(5-phenyl-2*H*-tetrazol-2-yl)propyl acetate (3a): $^{13}C$ NMR (151 MHz, $CDCl_3$)**

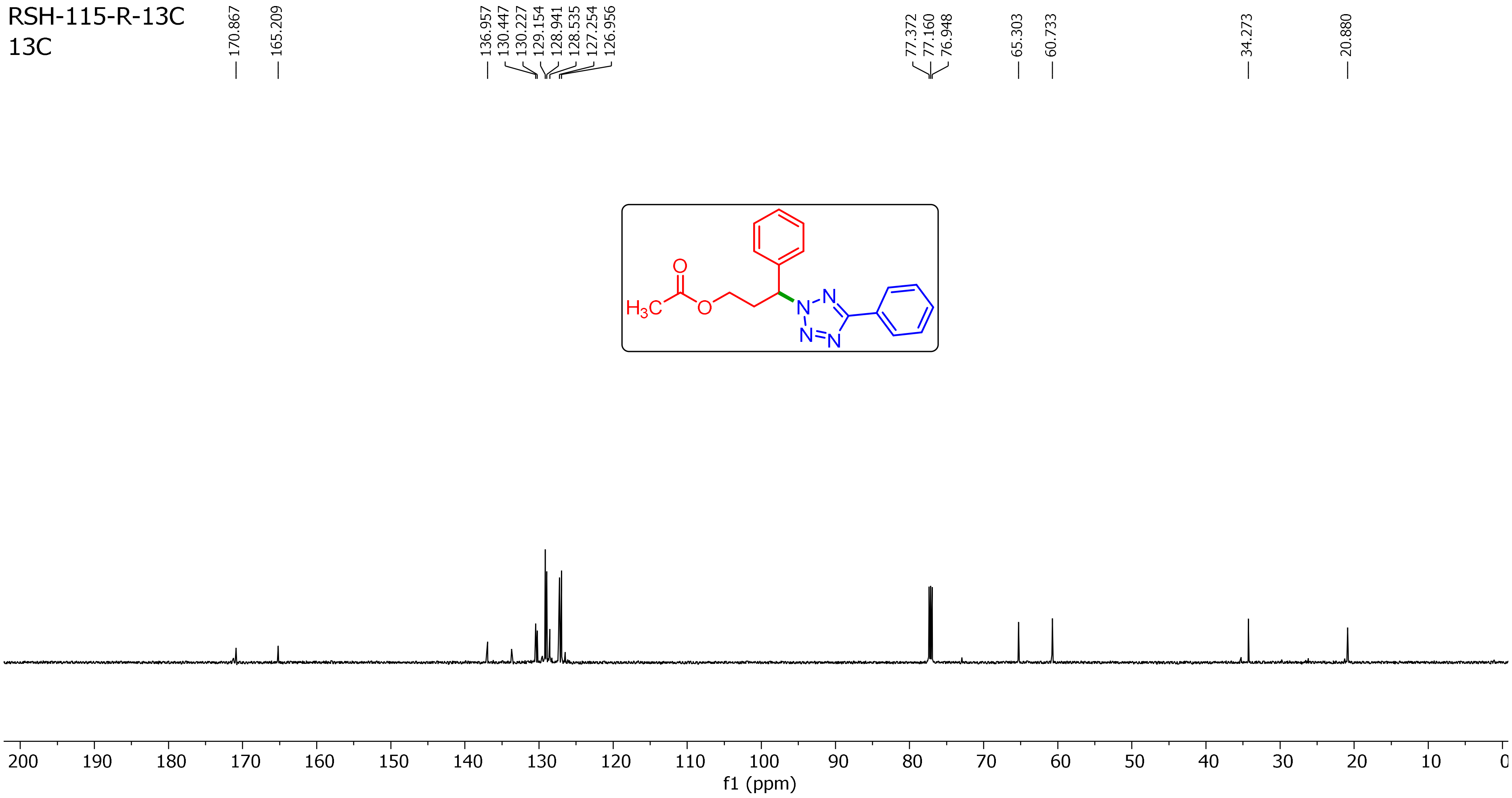

**4-Phenyl-4-(5-phenyl-2*H*-tetrazol-2-yl)butyl acetate (4a): $^{1}$H NMR (600 MHz, $CDCl_3$)**

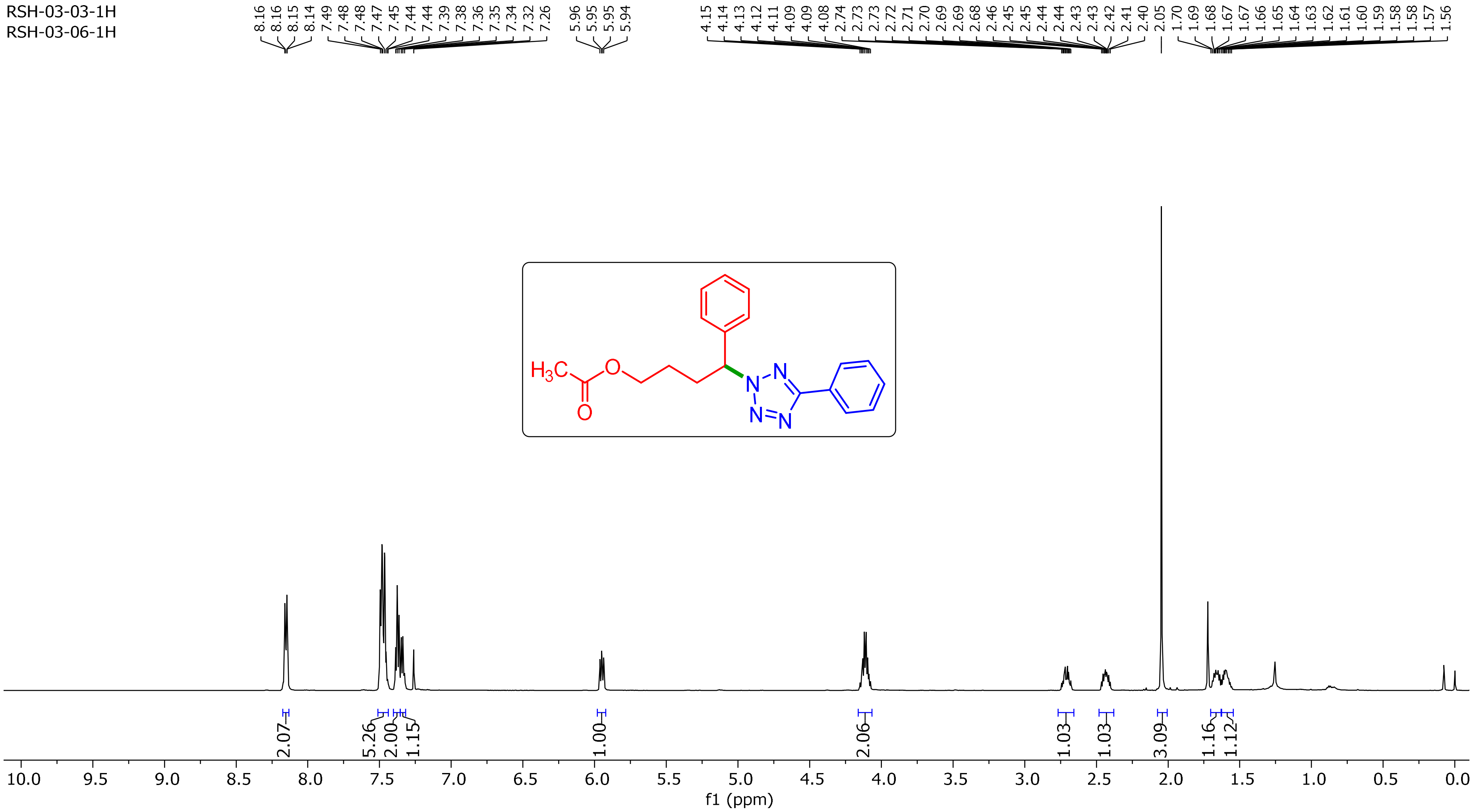

**4-Phenyl-4-(5-phenyl-2*H*-tetrazol-2-yl)butyl acetate (4a): $^{13}$C NMR (151 MHz, CDCl$_3$)**

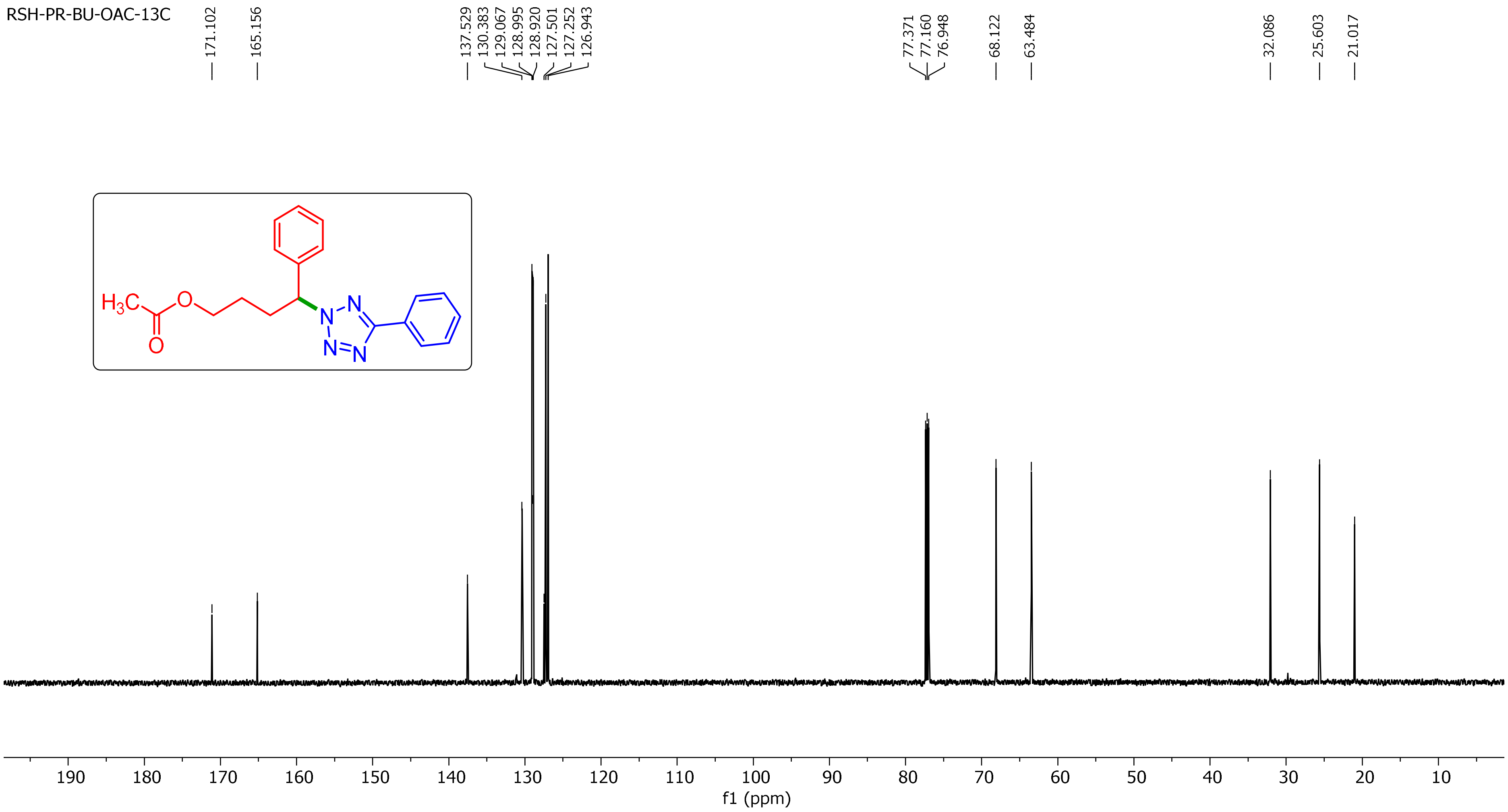

**3-Methyl-3-(5-phenyl-2*H*-tetrazol-2-yl)butyl acetate (5a): $^{1}$H NMR (600 MHz, CDCl3)**

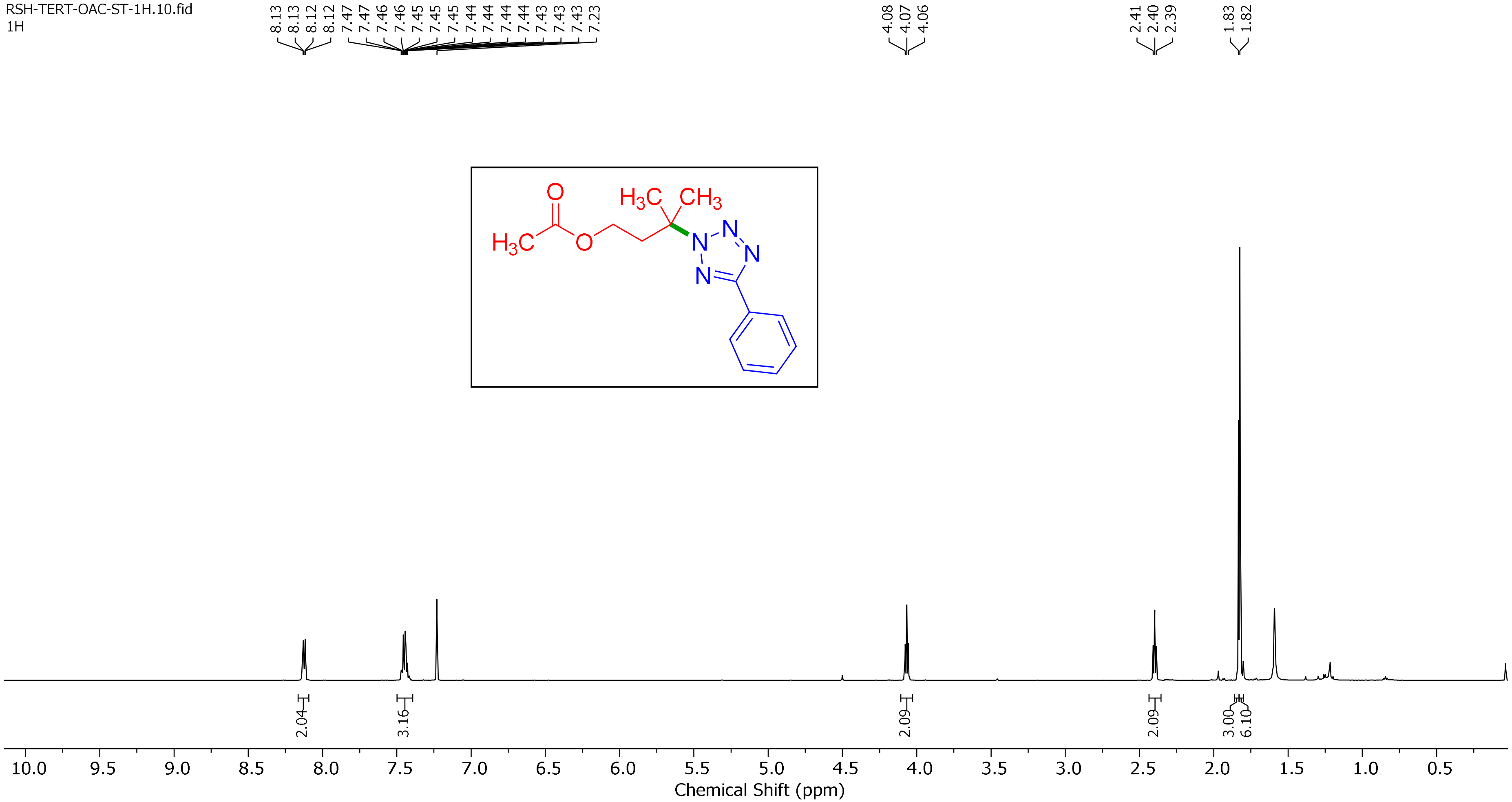

**3-Methyl-3-(5-phenyl-2*H*-tetrazol-2-yl)butyl acetate (5a): $^{13}$C NMR (151 MHz, CDCl$_3$)**

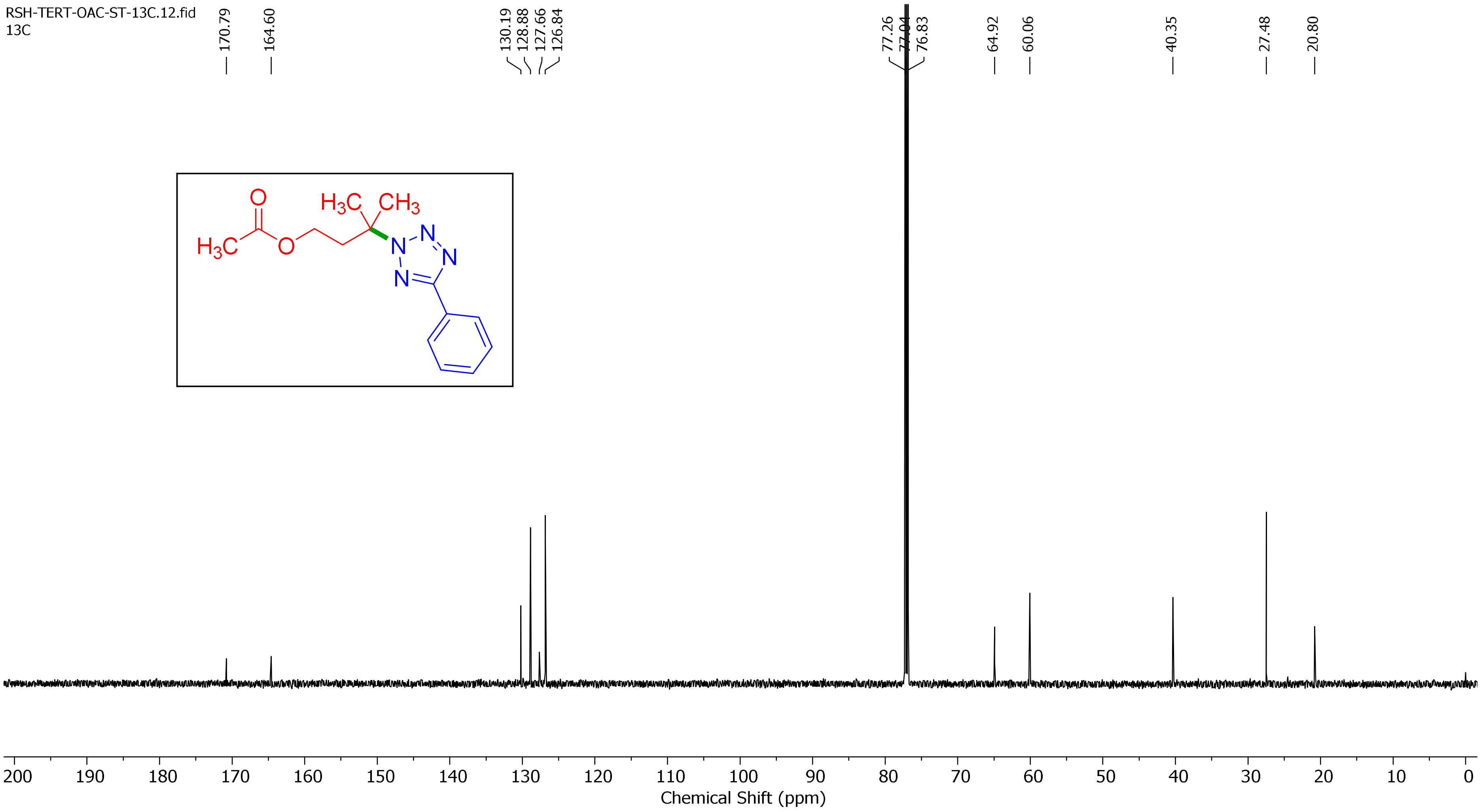

**4-(5-Phenyl-2*H*-tetrazol-2-yl)pentyl acetate (6a) (major) + 3-(5-Phenyl-2*H*-tetrazol-2-yl)pentyl acetate (6'a) (minor): $^{1}$H NMR (600 MHz, $CDCl_3$)**

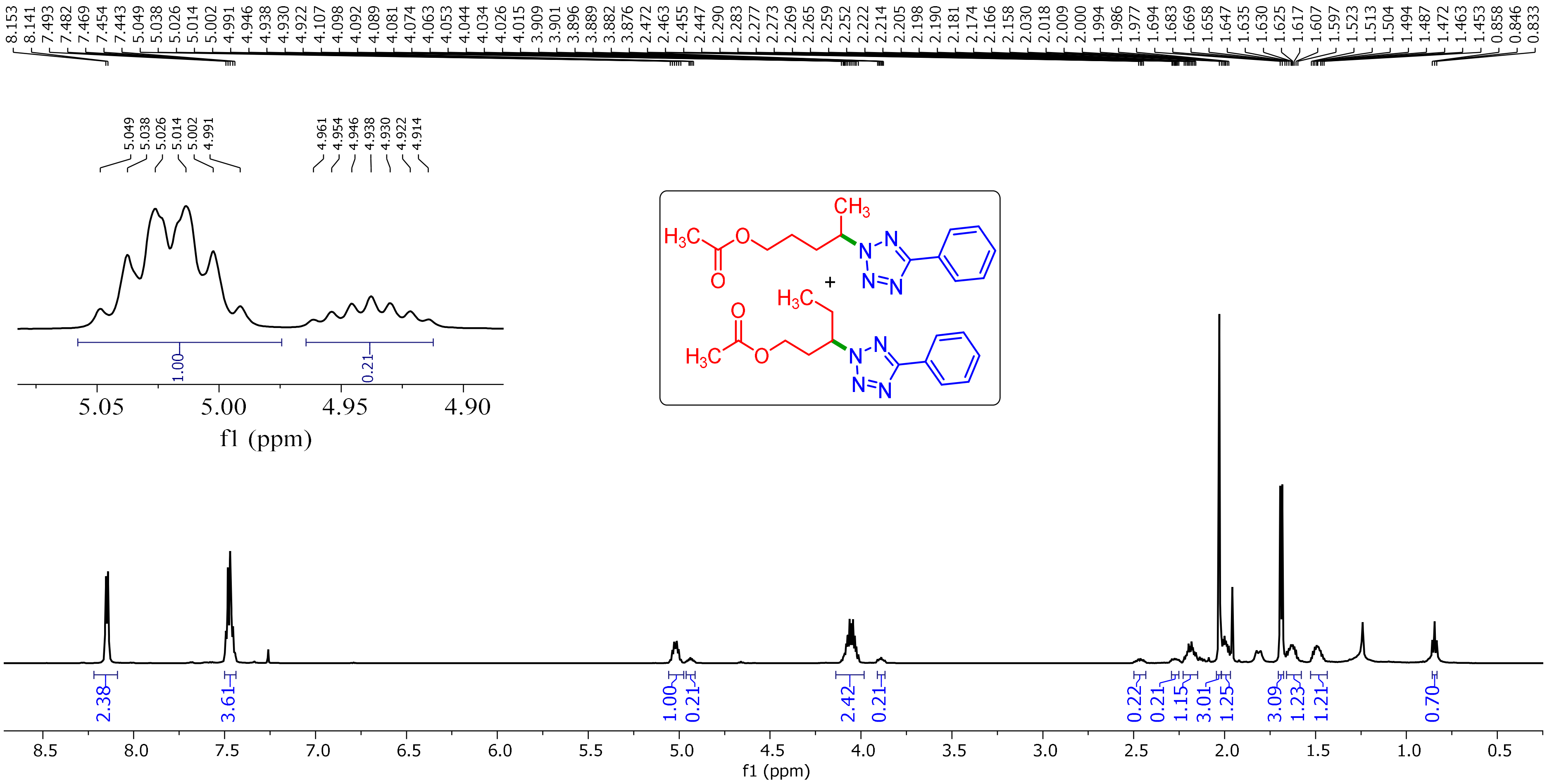

**4-(5-Phenyl-2*H*-tetrazol-2-yl)pentyl acetate (6a) (major) + 3-(5-Phenyl-2*H*-tetrazol-2-yl)pentyl acetate (6'a) (minor): $^{13}C$ NMR (151 MHz, $CDCl_3$)**

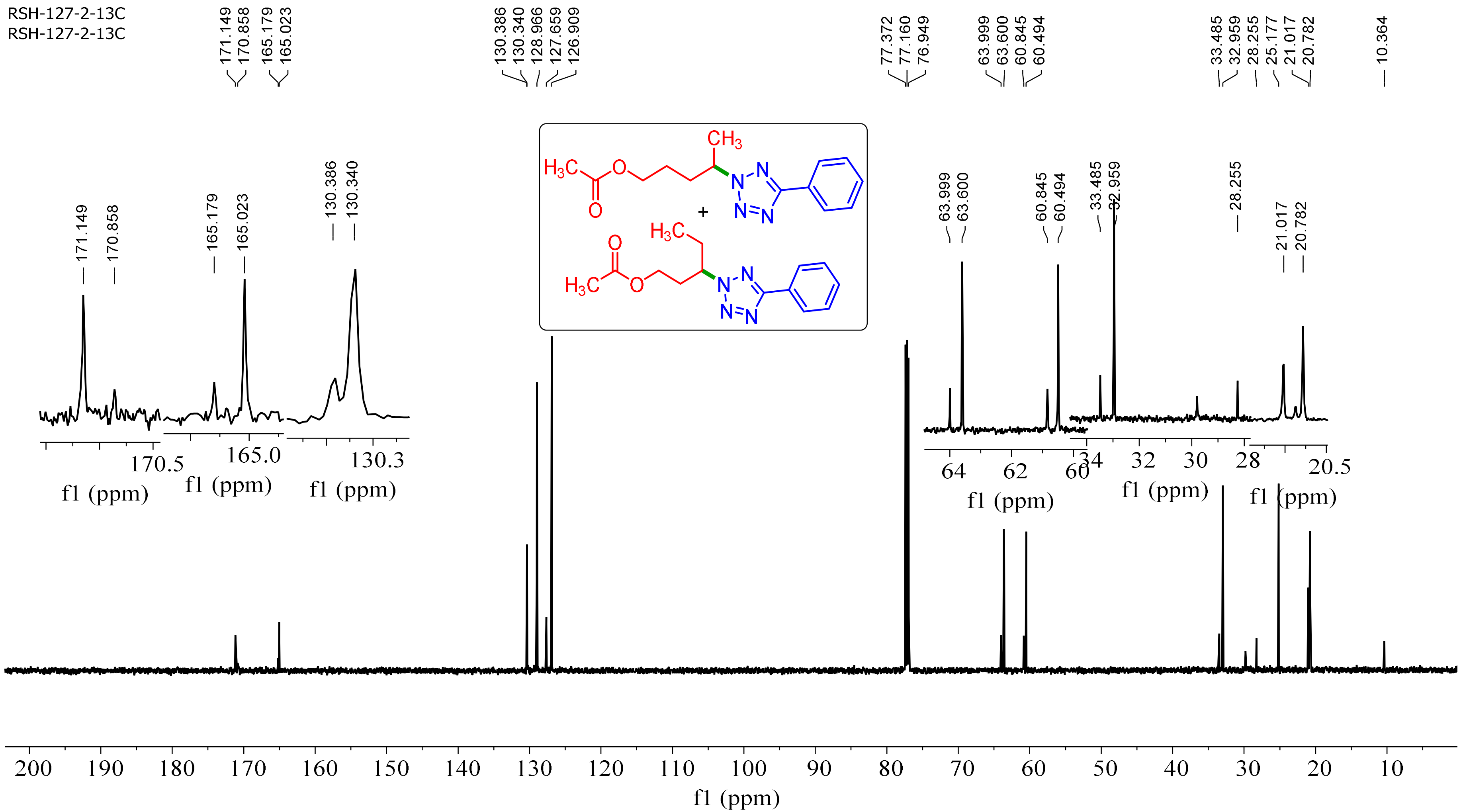

**5-(5-Phenyl-2*H*-tetrazol-2-yl)hexyl acetate (7a) (major) + 4-(5-Phenyl-2*H*-tetrazol-2-yl)hexyl acetate (7'a) (minor): $^{1}$H NMR (600 MHz, $CDCl_3$)**

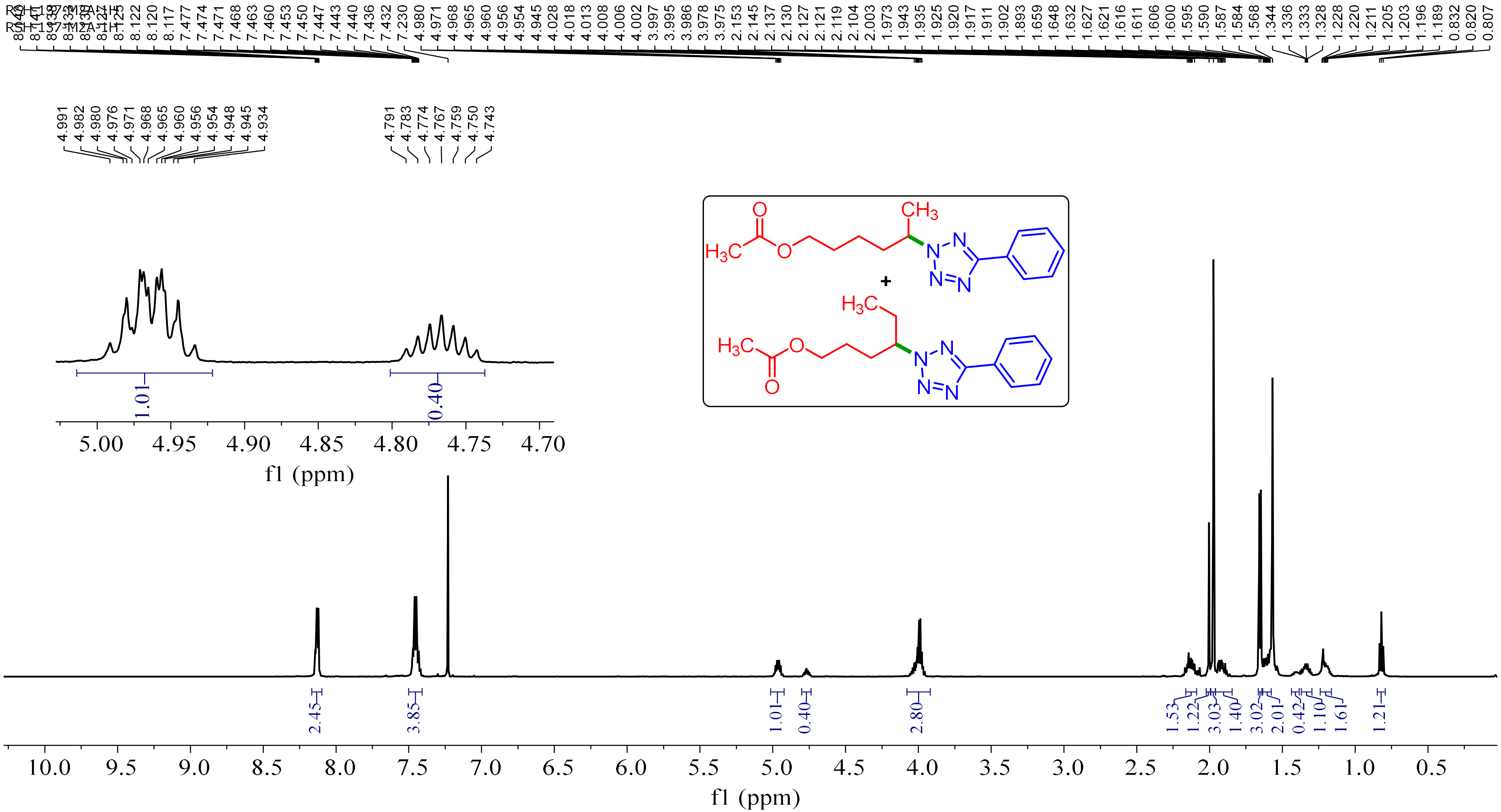

**5-(5-Phenyl-2*H*-tetrazol-2-yl)hexyl acetate (7a) (major) + 4-(5-Phenyl-2*H*-tetrazol-2-yl)hexyl acetate (7'a) (minor): $^{13}$C NMR (151 MHz, CDCl$_3$)**

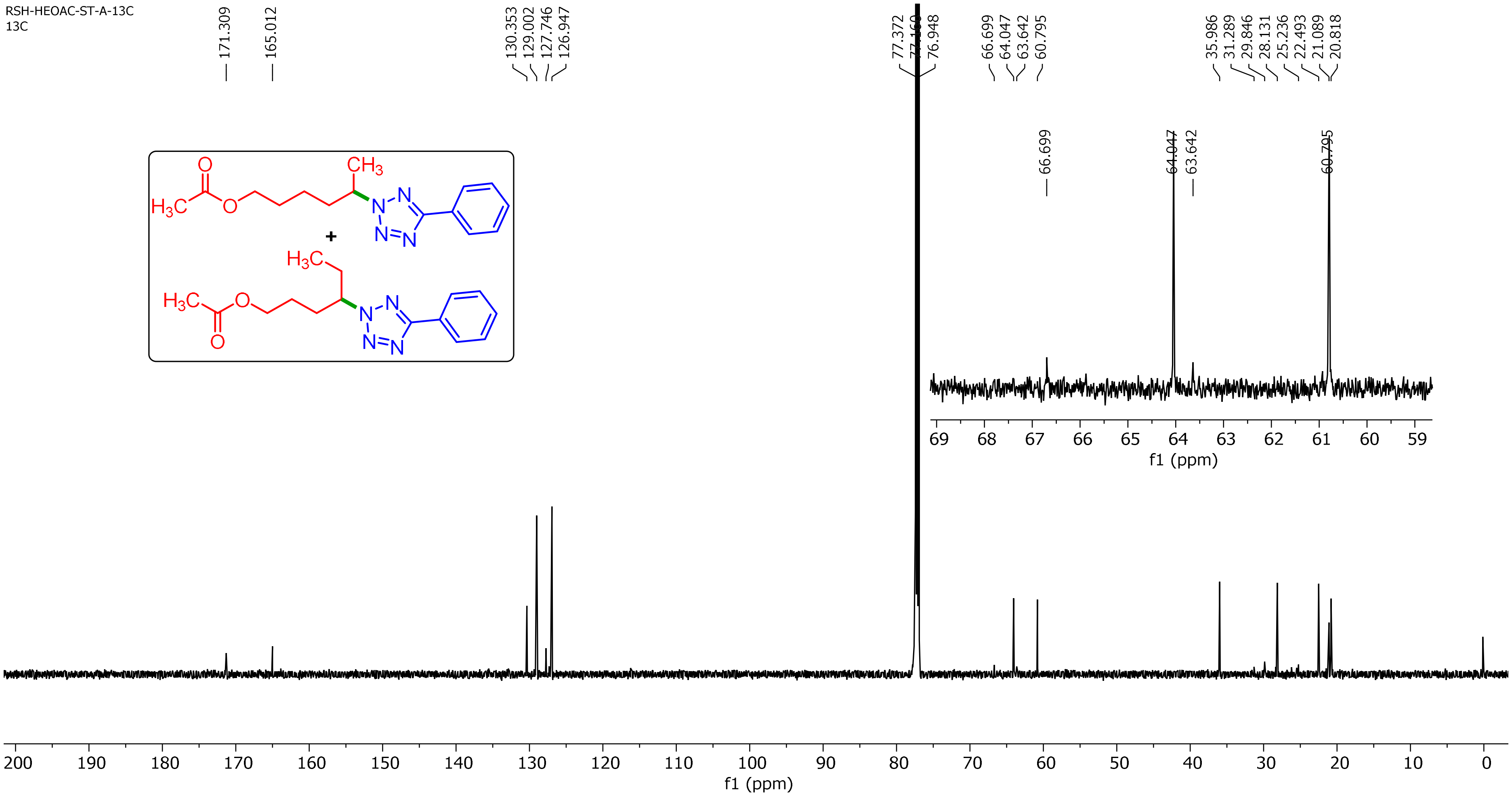

**1-(5-Phenyl-2*H*-tetrazol-2-yl)ethyl acetate (9a): $^{1}$H NMR (600 MHz, $CDCl_3$)**

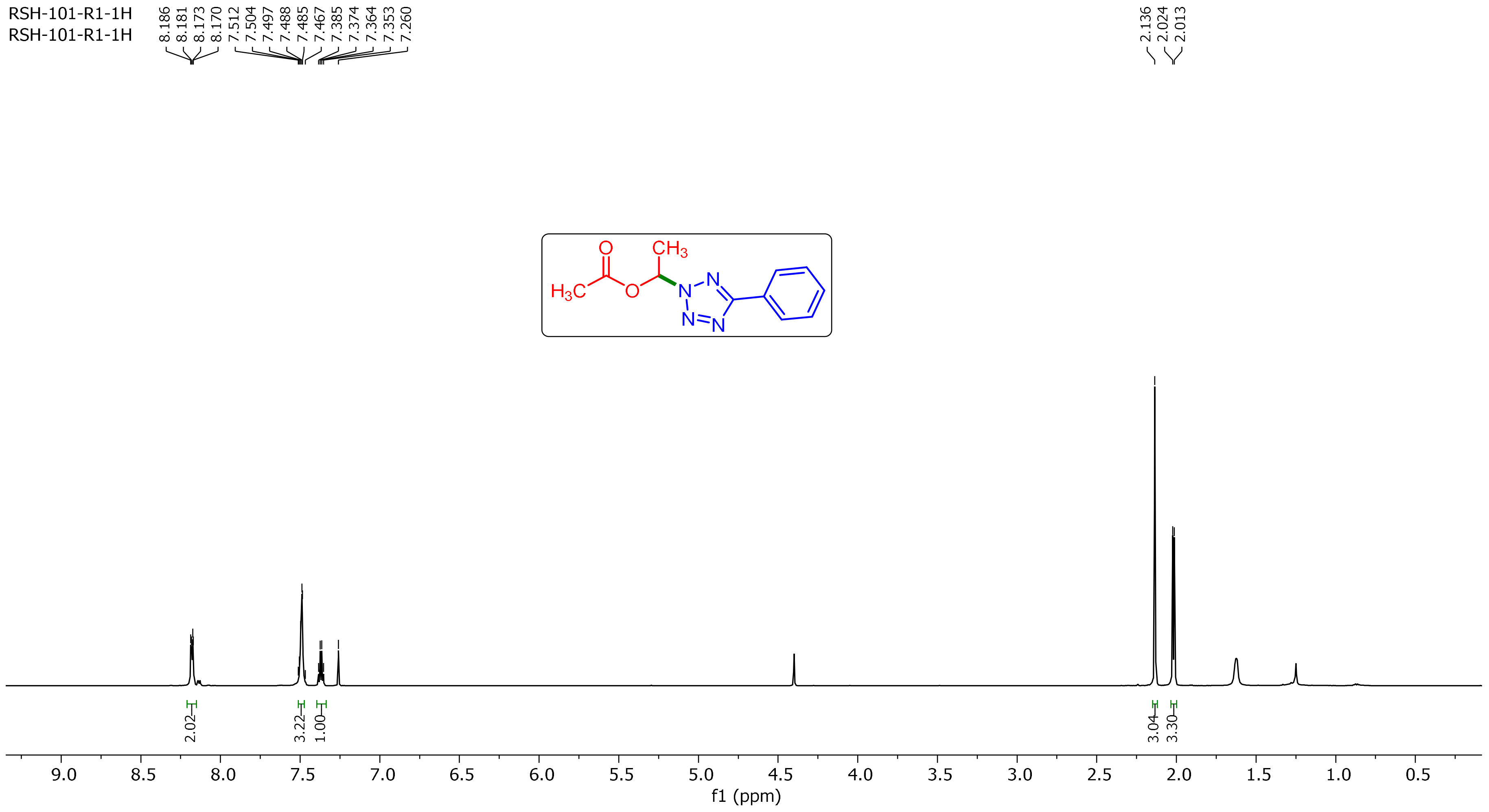

**1-(5-Phenyl-2*H*-tetrazol-2-yl)ethyl acetate (9a): $^{13}$C NMR (151 MHz, $CDCl_3$)**

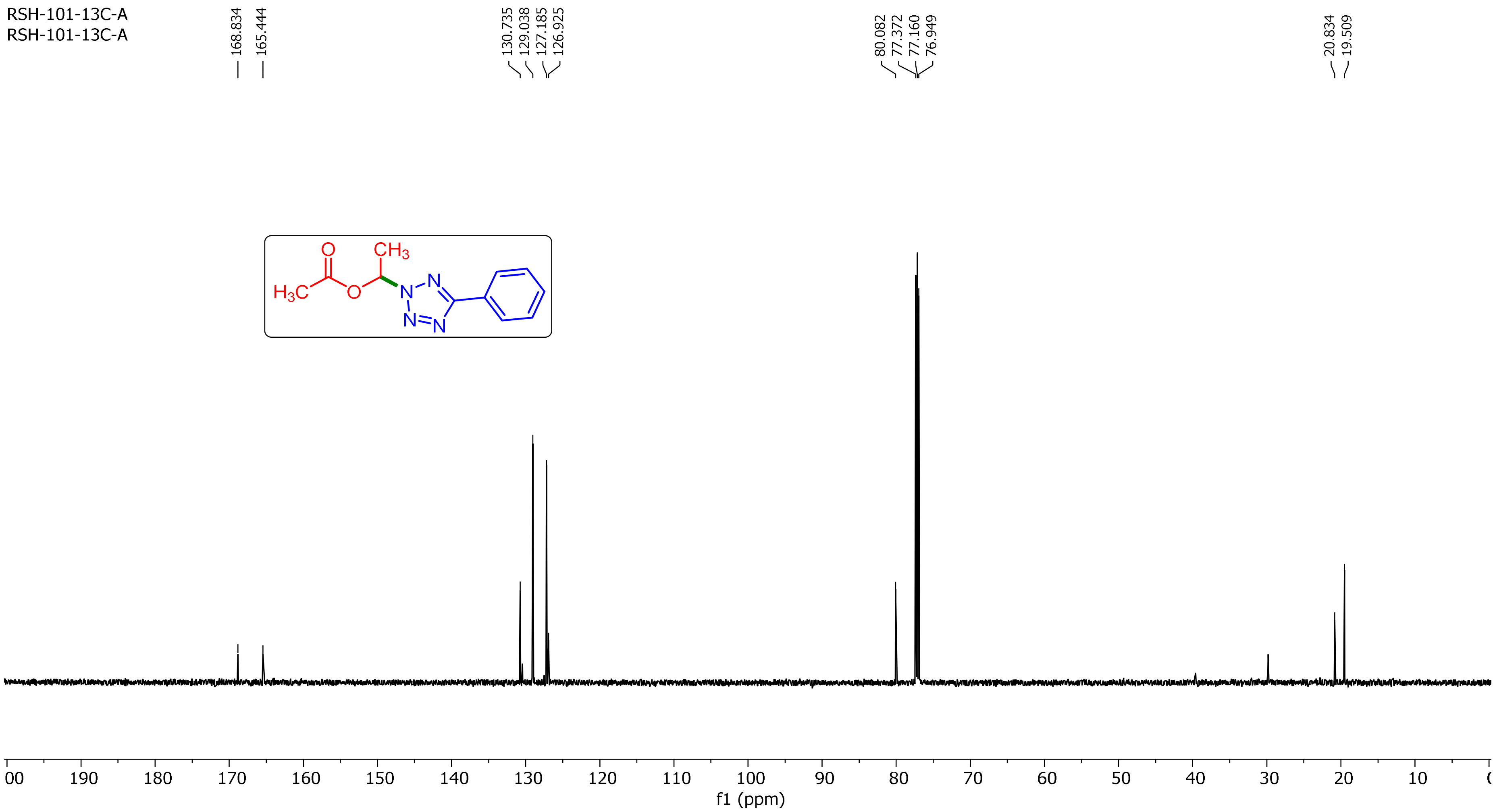

**1-(5-(4-Chlorophenyl)-2*H*-tetrazol-2-yl)ethyl acetate (9h): $^1$H NMR (600 MHz, CDCl$_3$)**

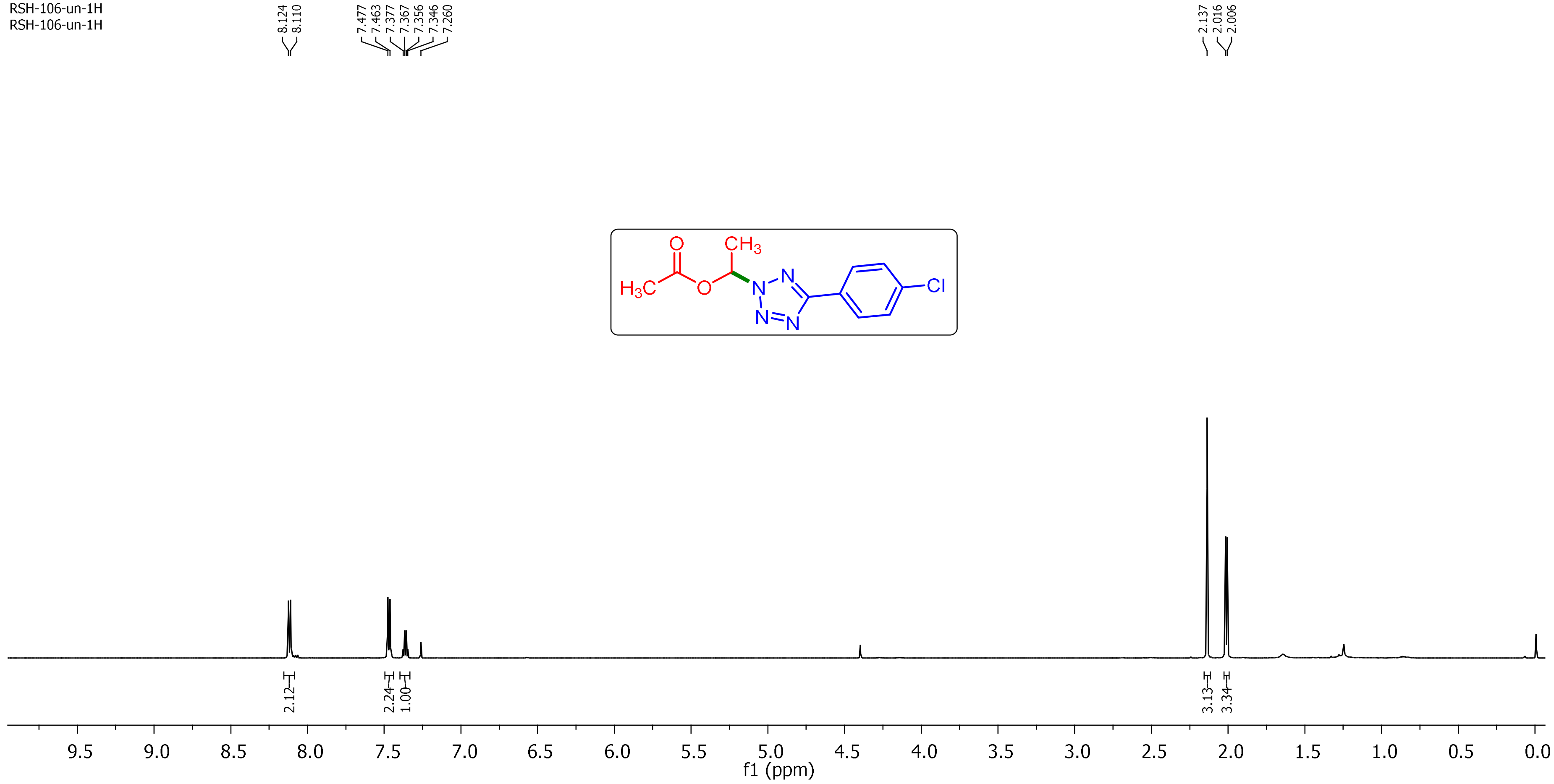

**1-(5-(4-Chlorophenyl)-2*H*-tetrazol-2-yl)ethyl acetate (9h): $^{13}$C NMR (151 MHz, CDCl$_3$)**

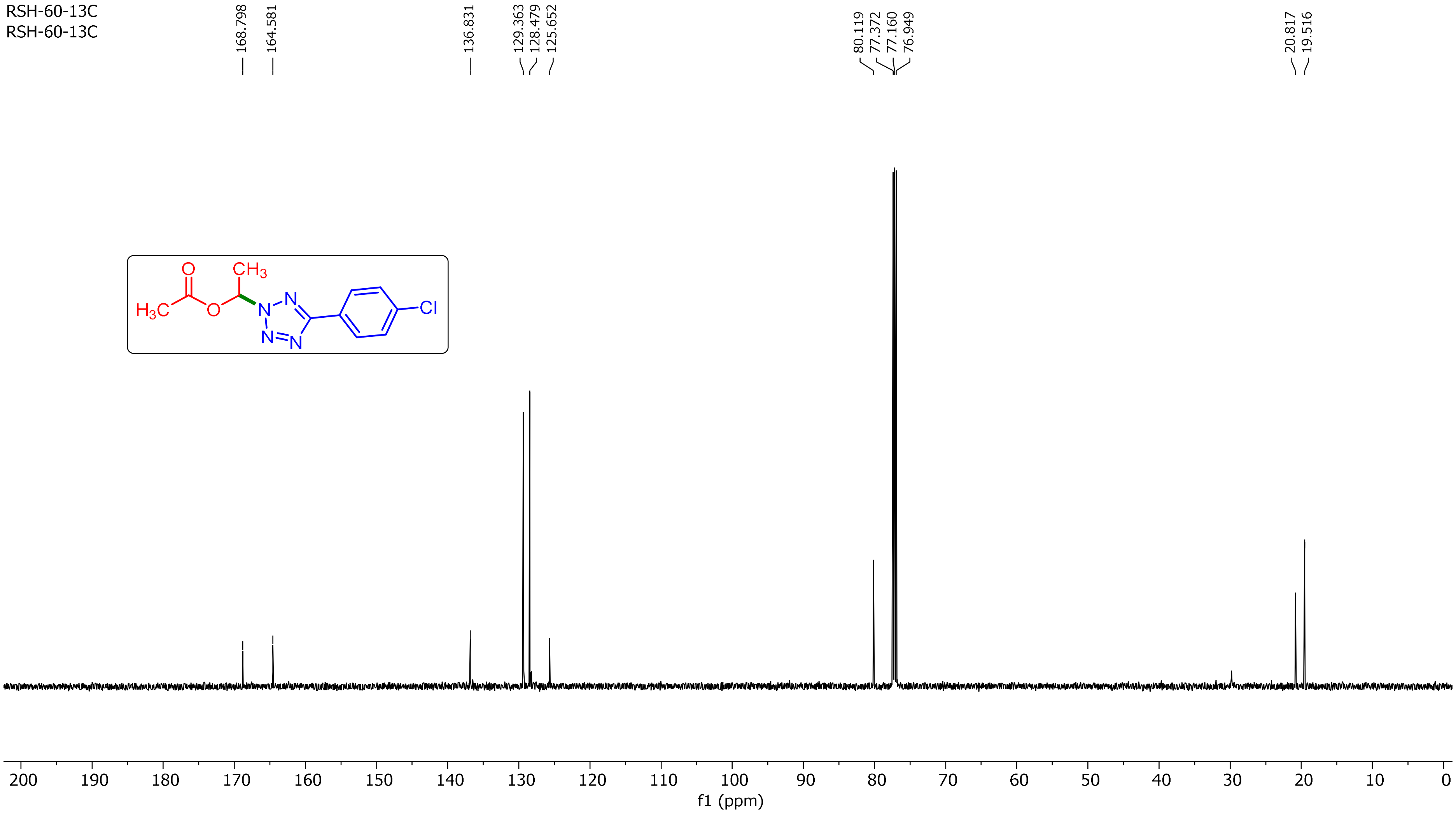

**1-(5-(4-Bromophenyl)-2*H*-tetrazol-2-yl)ethyl acetate (9i): $^{1}$H NMR (600 MHz, $CDCl_3$)**

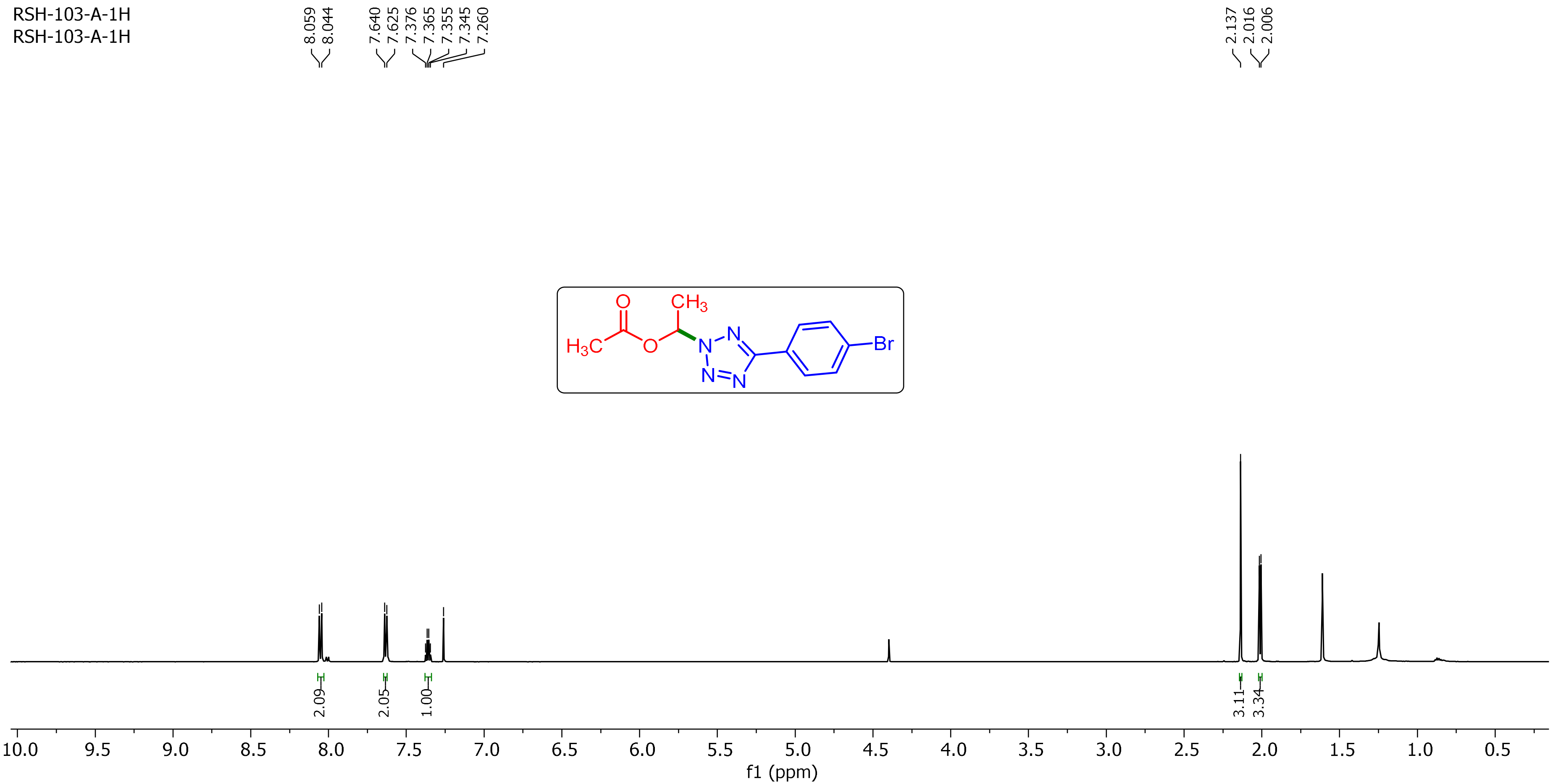

**1-(5-(4-Bromophenyl)-2*H*-tetrazol-2-yl)ethyl acetate (9i): $^{13}$C NMR (151 MHz, CDCl$_3$)**

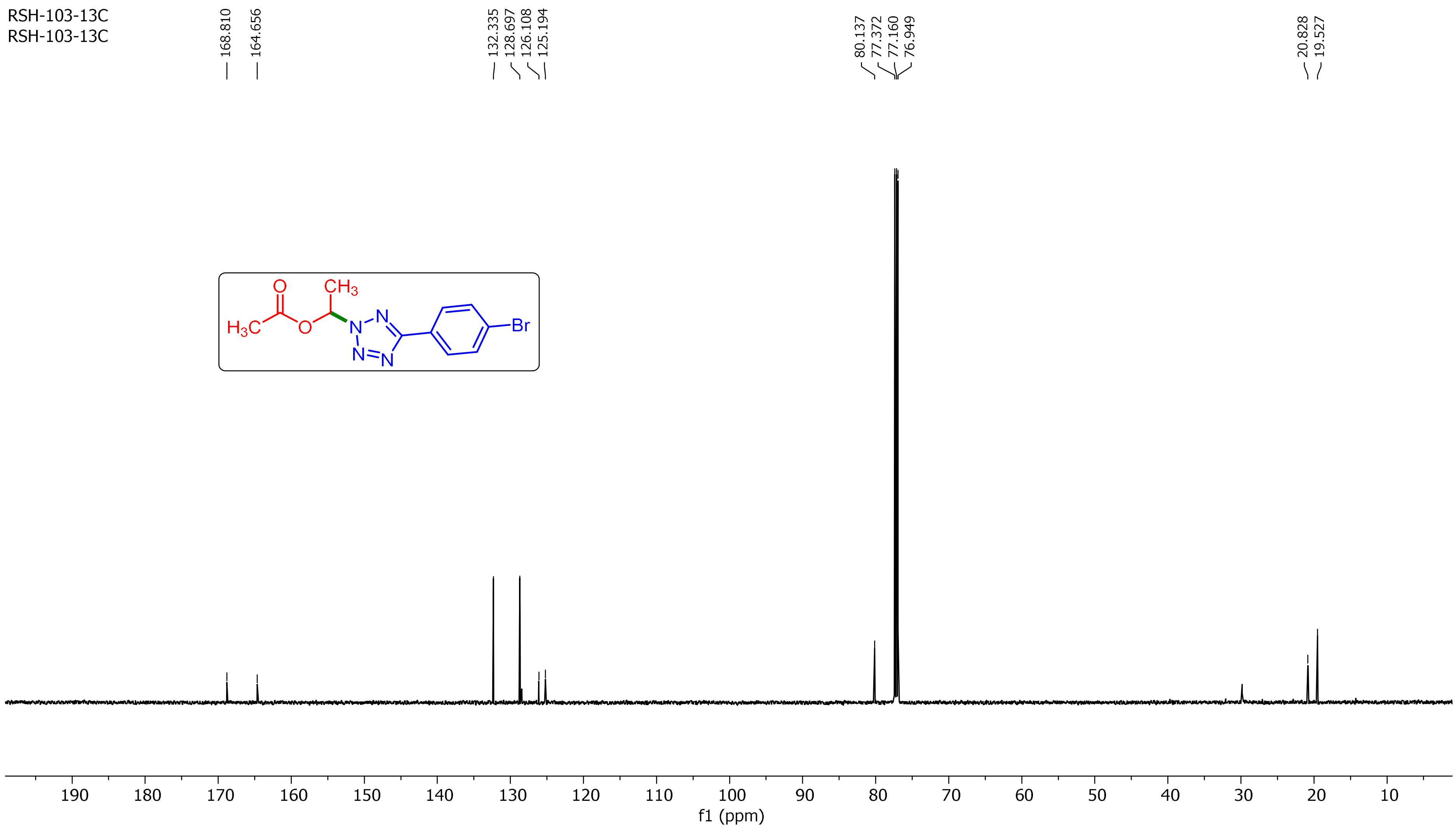

**1-(5-(*p*-Tolyl)-2*H*-tetrazol-2-yl)ethyl acetate (9d) + 2-Methyl-5-(*p*-tolyl)-2*H*-tetrazole (dm): $^1$H NMR (600 MHz, $CDCl_3$)**

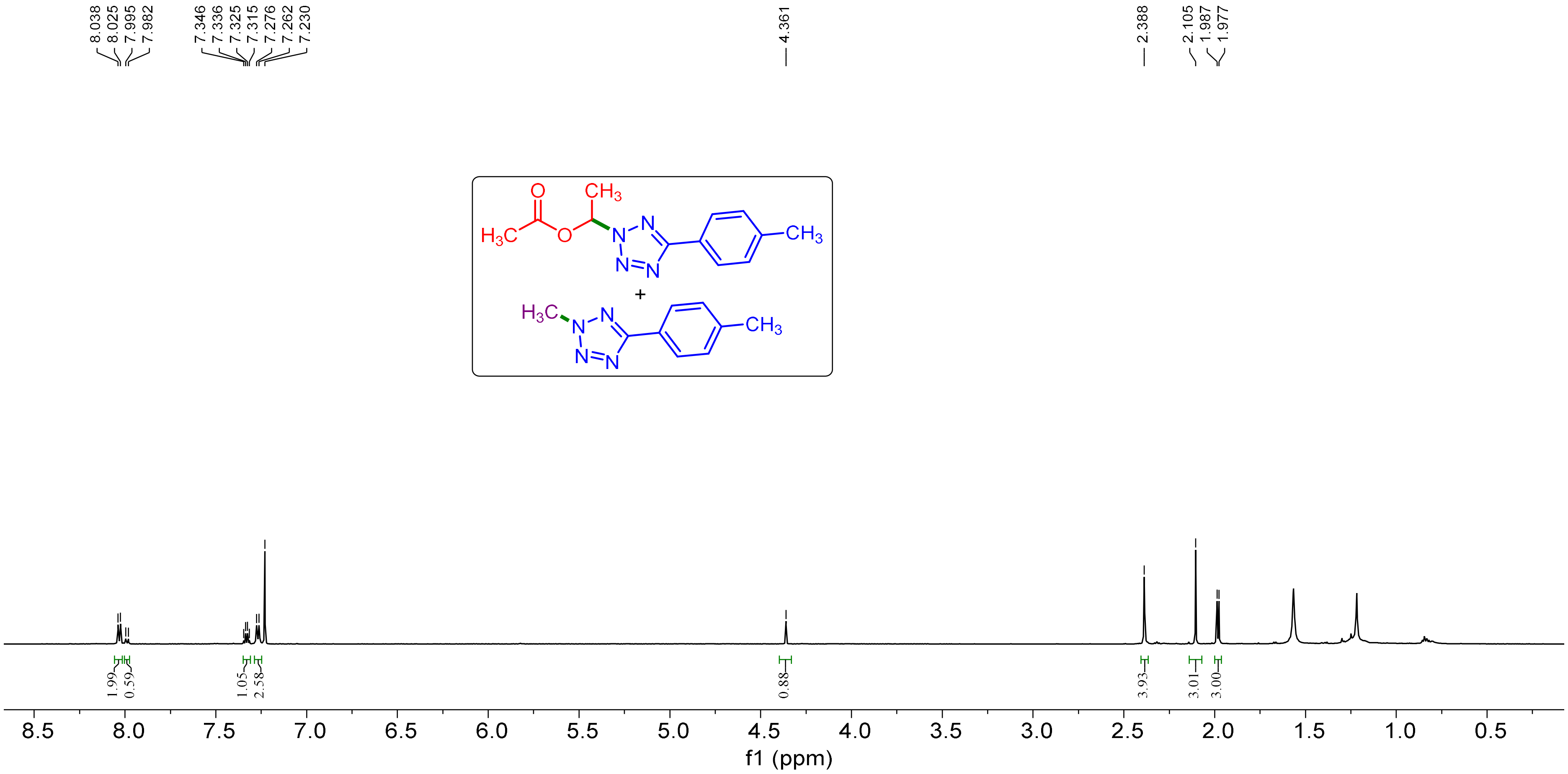

**1-(5-(*p*-Tolyl)-2*H*-tetrazol-2-yl)ethyl acetate (9d) + 2-Methyl-5-(*p*-tolyl)-2*H*-tetrazole (dm): $^{13}$C NMR (151 MHz, $CDCl_3$)**

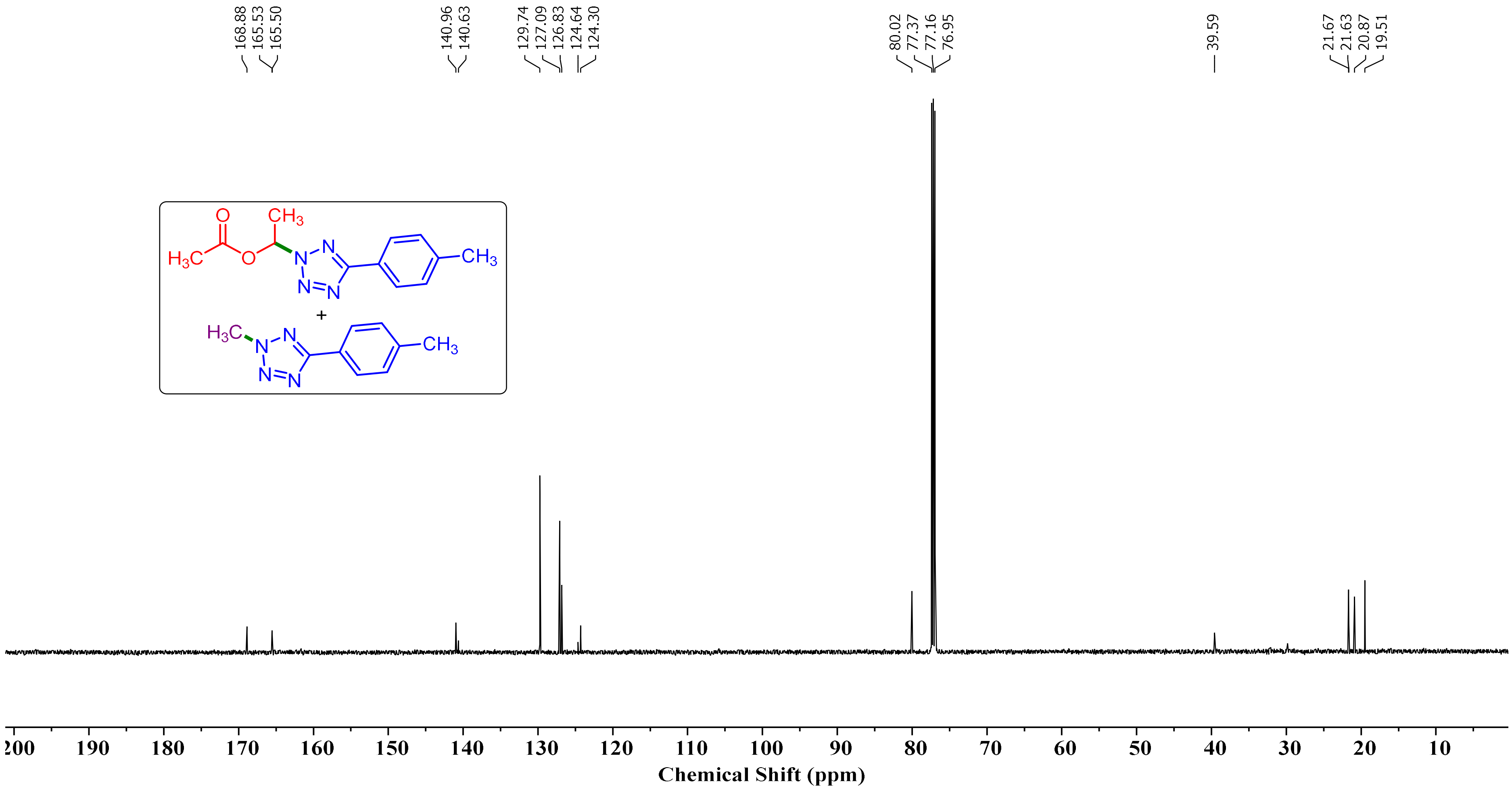

**1-(5-(3-Methoxyphenyl)-2*H*-tetrazol-2-yl)ethyl acetate (9g): $^{1}$H NMR (600 MHz, $CDCl_3$)**

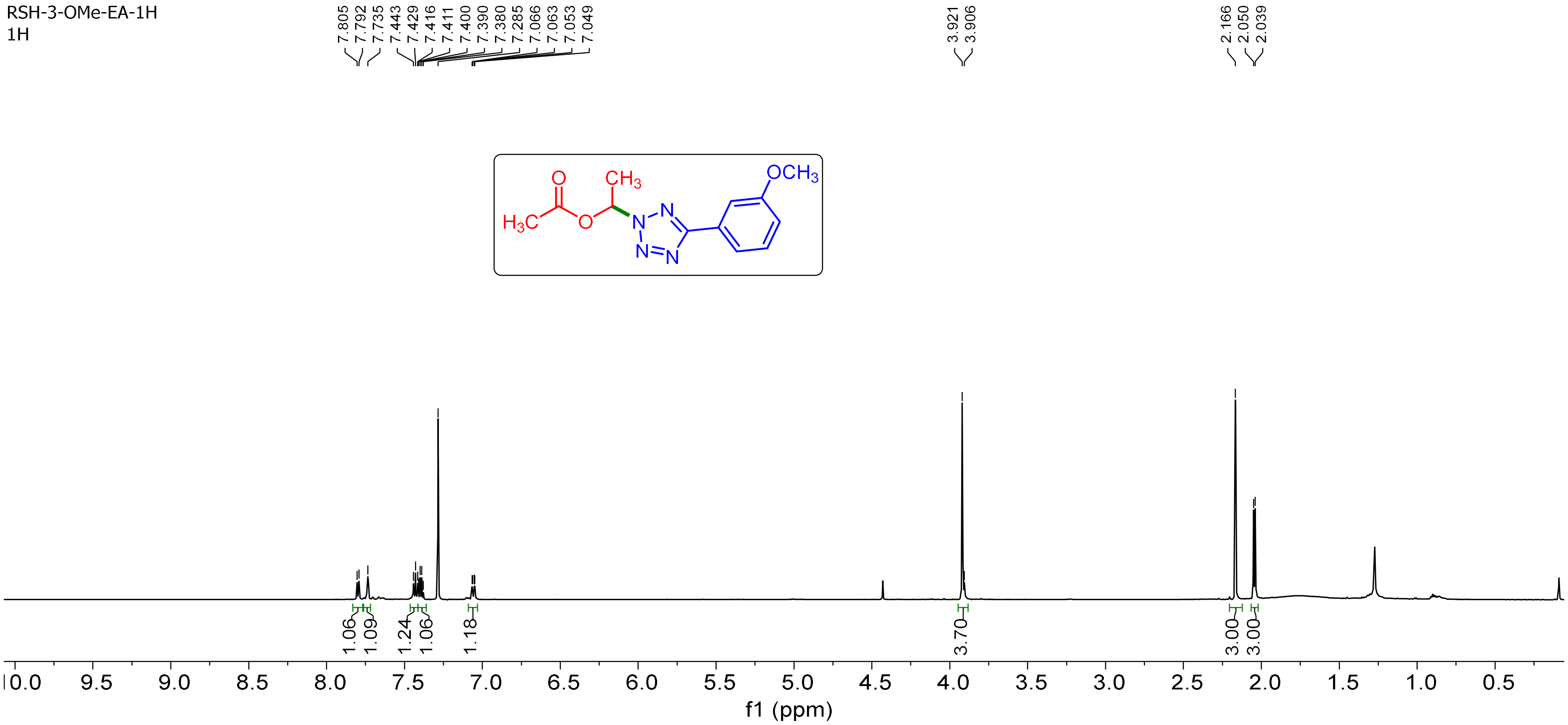

**1-(5-(3-Methoxyphenyl)-2*H*-tetrazol-2-yl)ethyl acetate (9g): $^{13}$C NMR (151 MHz, $CDCl_3$)**

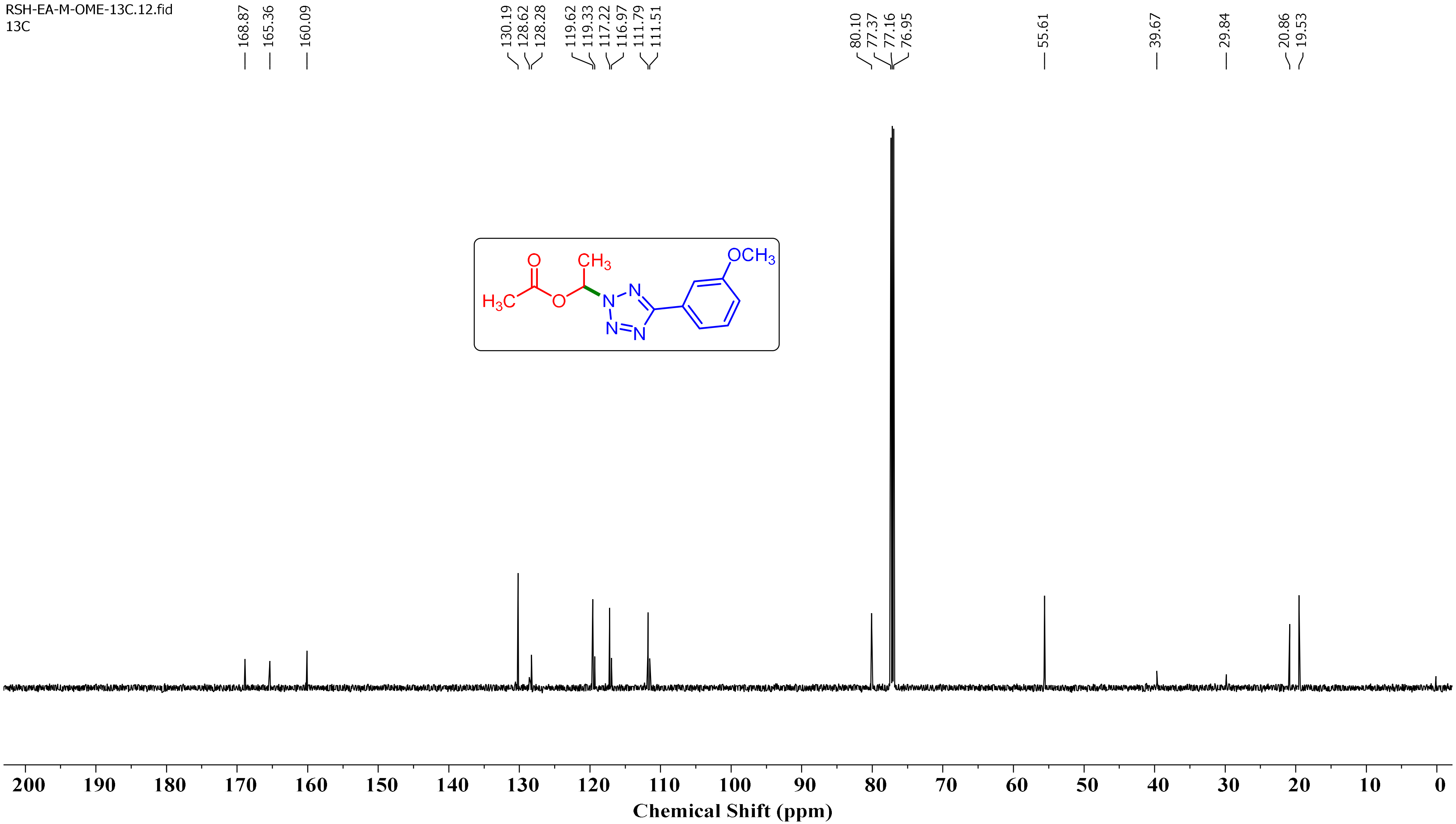

**1-(5-Phenyl-2*H*-tetrazol-2-yl)ethyl benzoate (10a): $^{1}$H NMR (600 MHz, $CDCl_3$)**

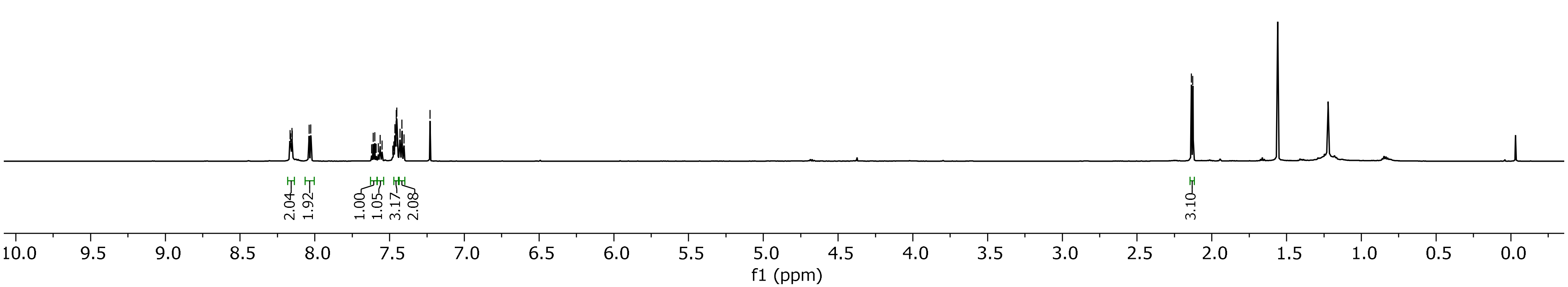


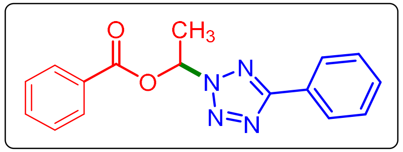

**1-(5-Phenyl-2*H*-tetrazol-2-yl)ethyl benzoate (10a): $^{13}$C NMR (151 MHz, CDCl$_3$)**

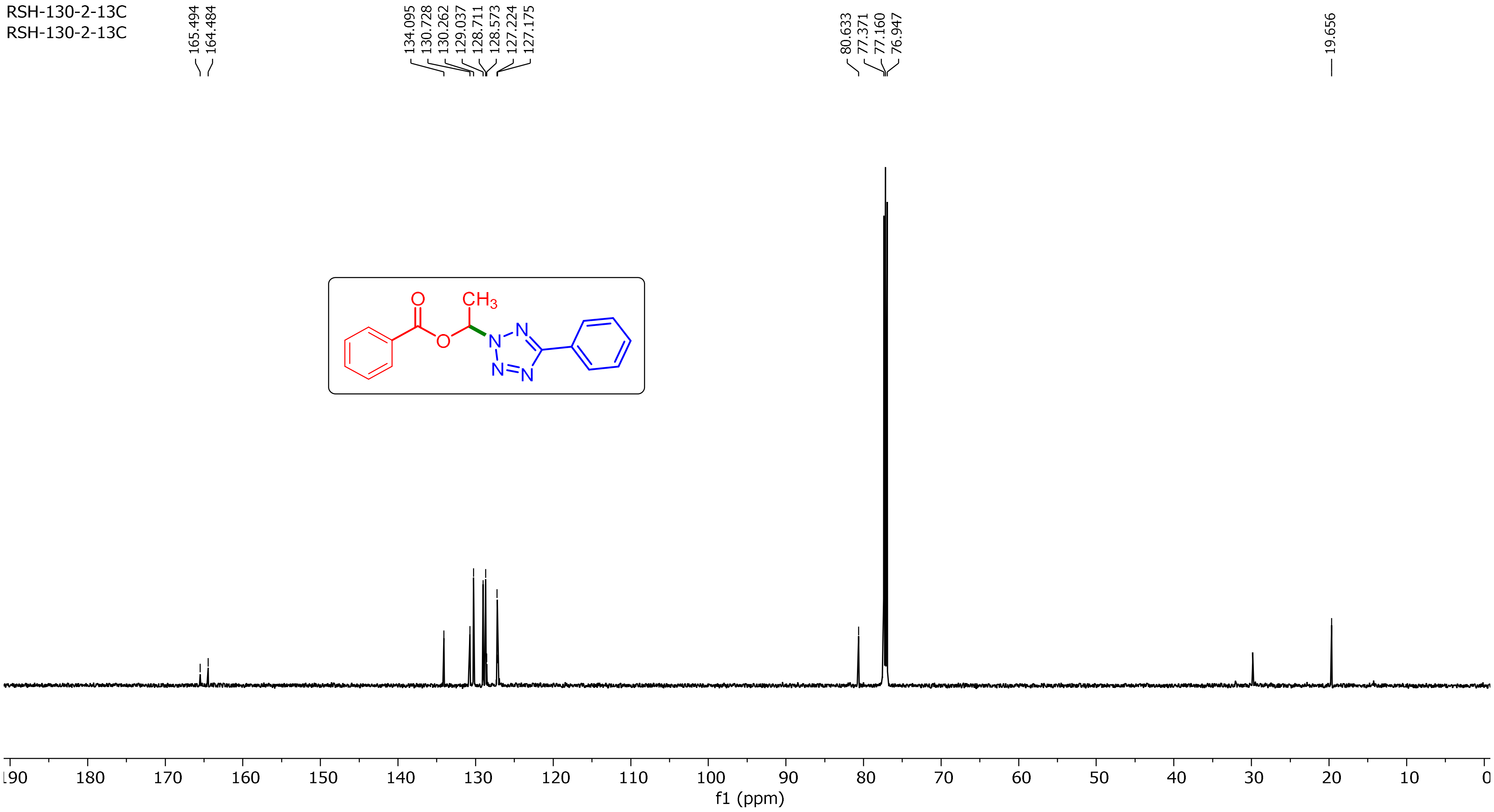

**1-(5-Phenyl-2*H*-tetrazol-2-yl)propyl acetate (11a): $^1$H NMR (600 MHz, $CDCl_3$)**

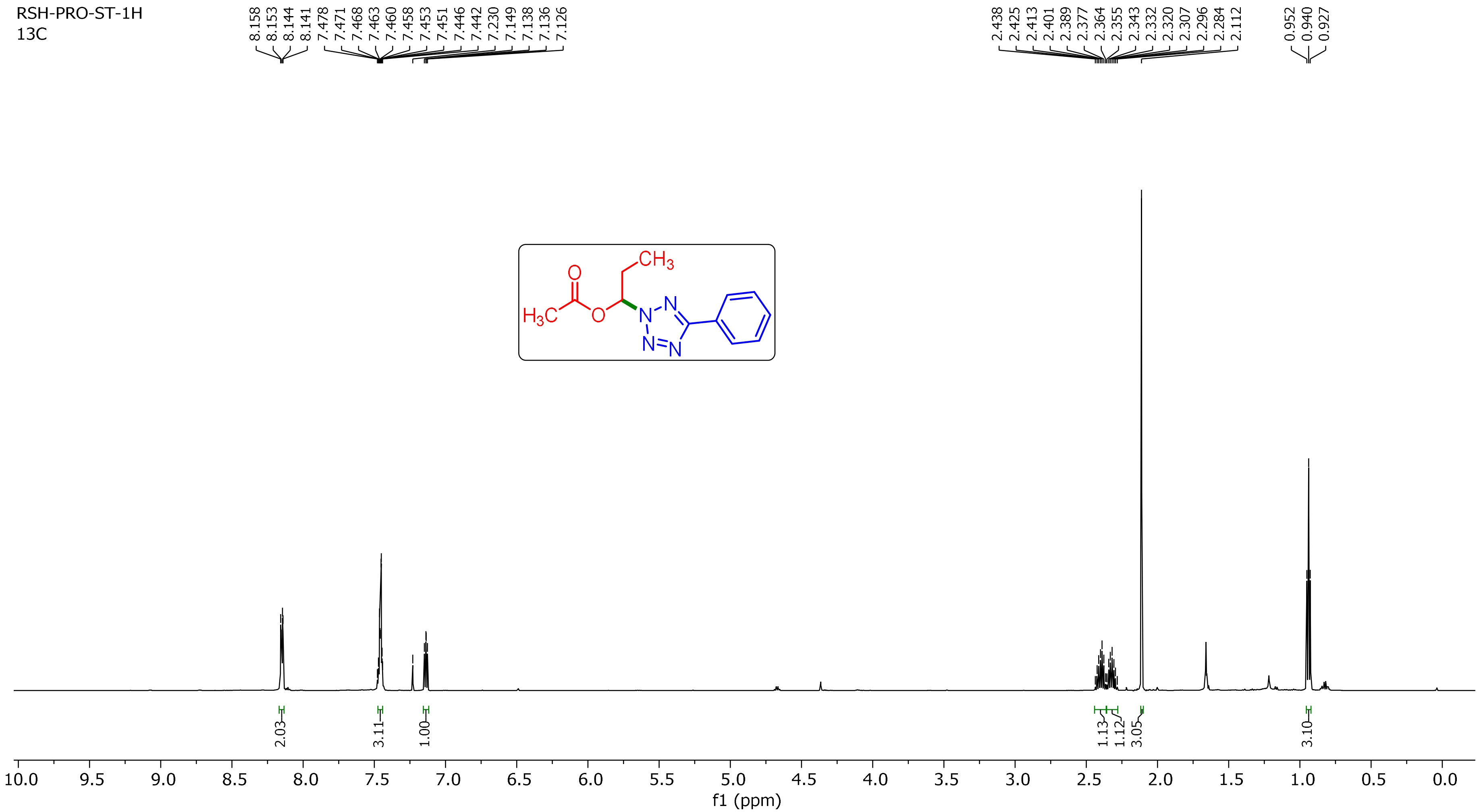

**1-(5-Phenyl-2*H*-tetrazol-2-yl)propyl acetate (11a): $^{13}$C NMR (151 MHz, CDCl$_3$)**

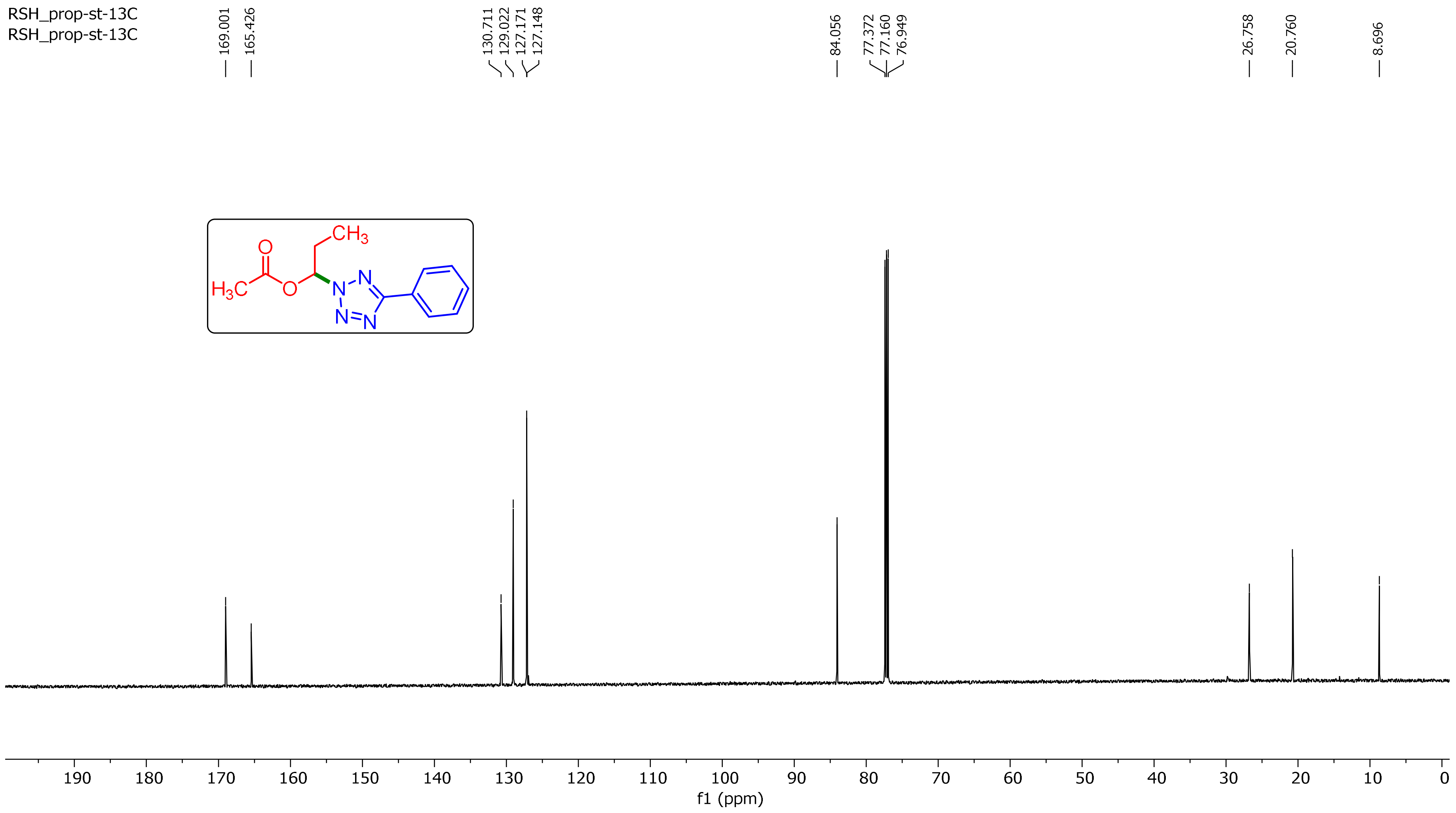

**3-(5-Phenyl-2*H*-tetrazol-2-yl)propyl acetate (11'a): $^{1}$H NMR (600 MHz, $CDCl_3$)**

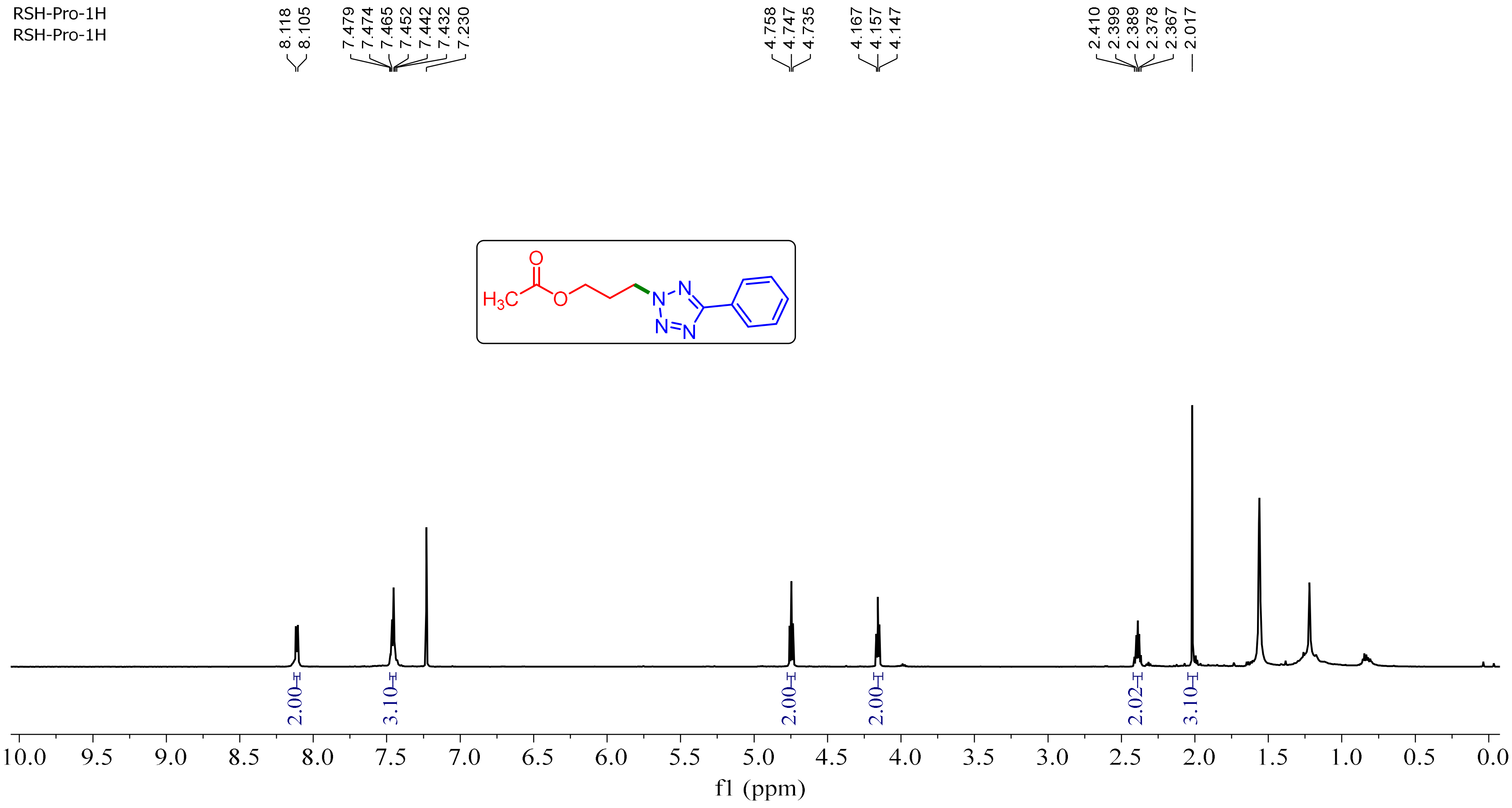

**3-(5-Phenyl-2*H*-tetrazol-2-yl)propyl acetate (11'a): $^{13}$C NMR (151 MHz, $CDCl_3$)**

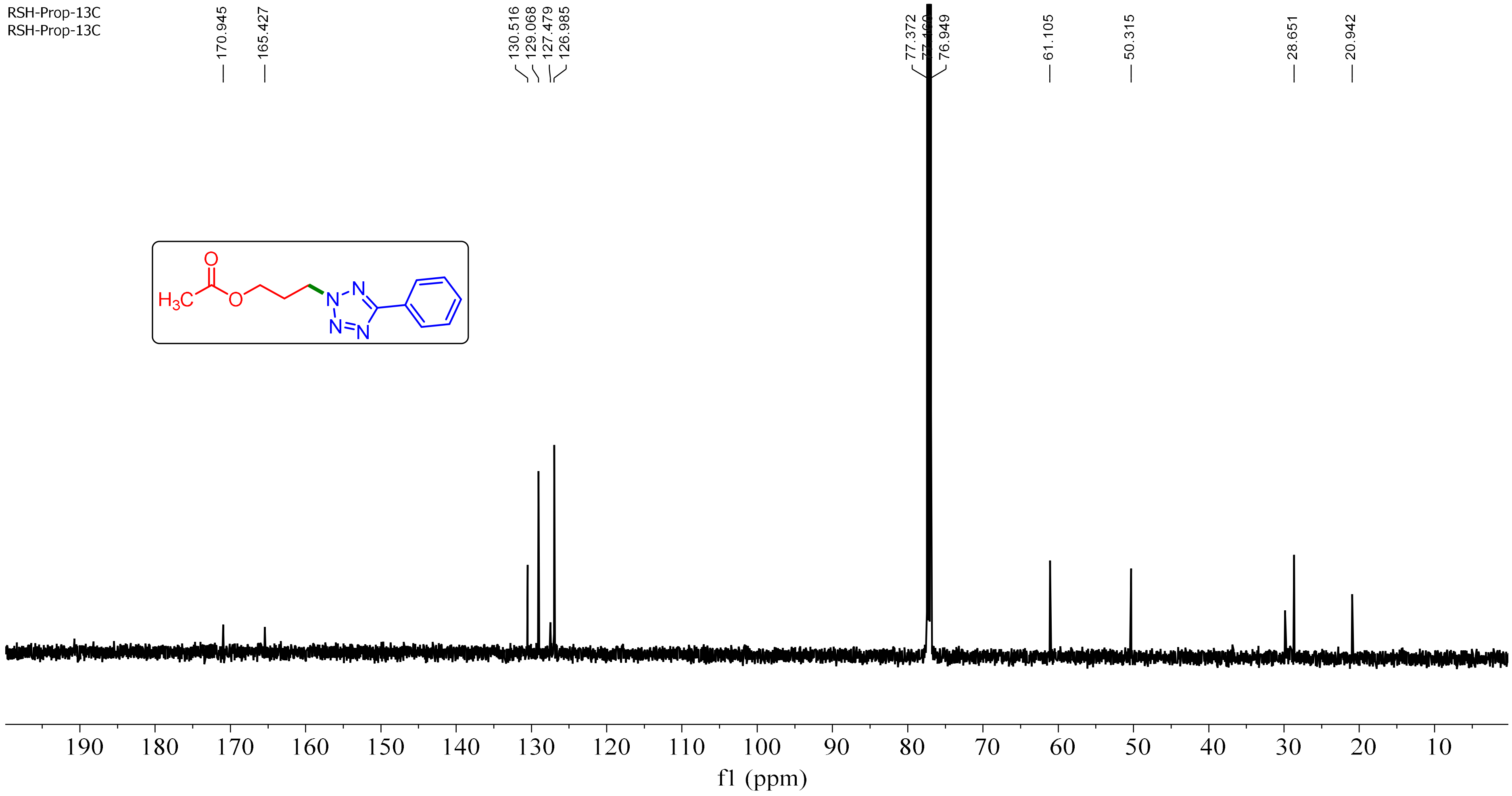

**N-Butyl-4-nitro-N-(3-(5-phenyl-2H-tetrazol-2-yl)butyl)benzenesulfonamide (14a): $^{1}$H NMR (600 MHz, CDCl₃)**

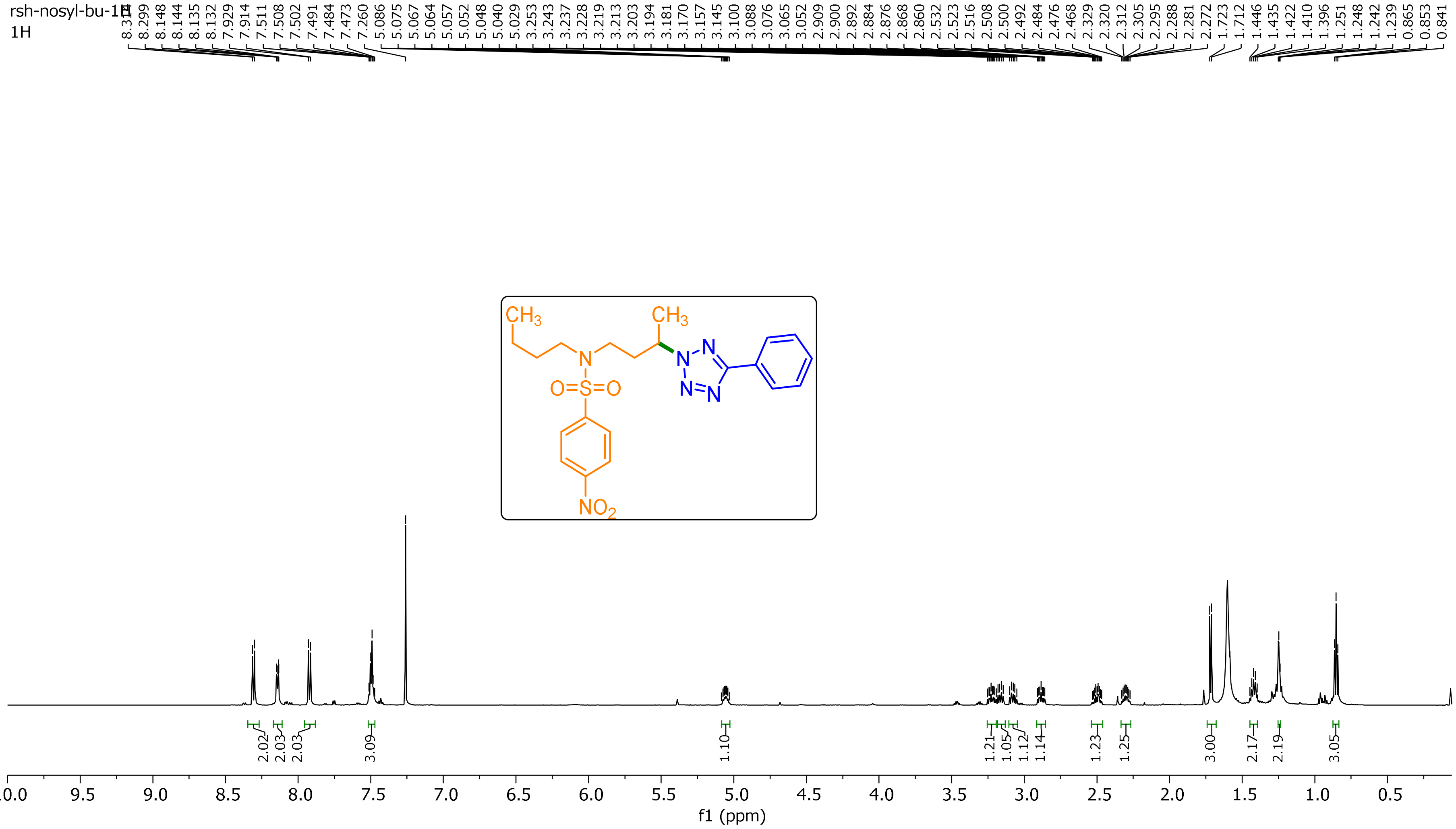

***N*-Butyl-4-nitro-*N*-(3-(5-phenyl-2*H*-tetrazol-2-yl)butyl)benzenesulfonamide (14a): $^{13}$C NMR (151 MHz, CDCl$_3$)**

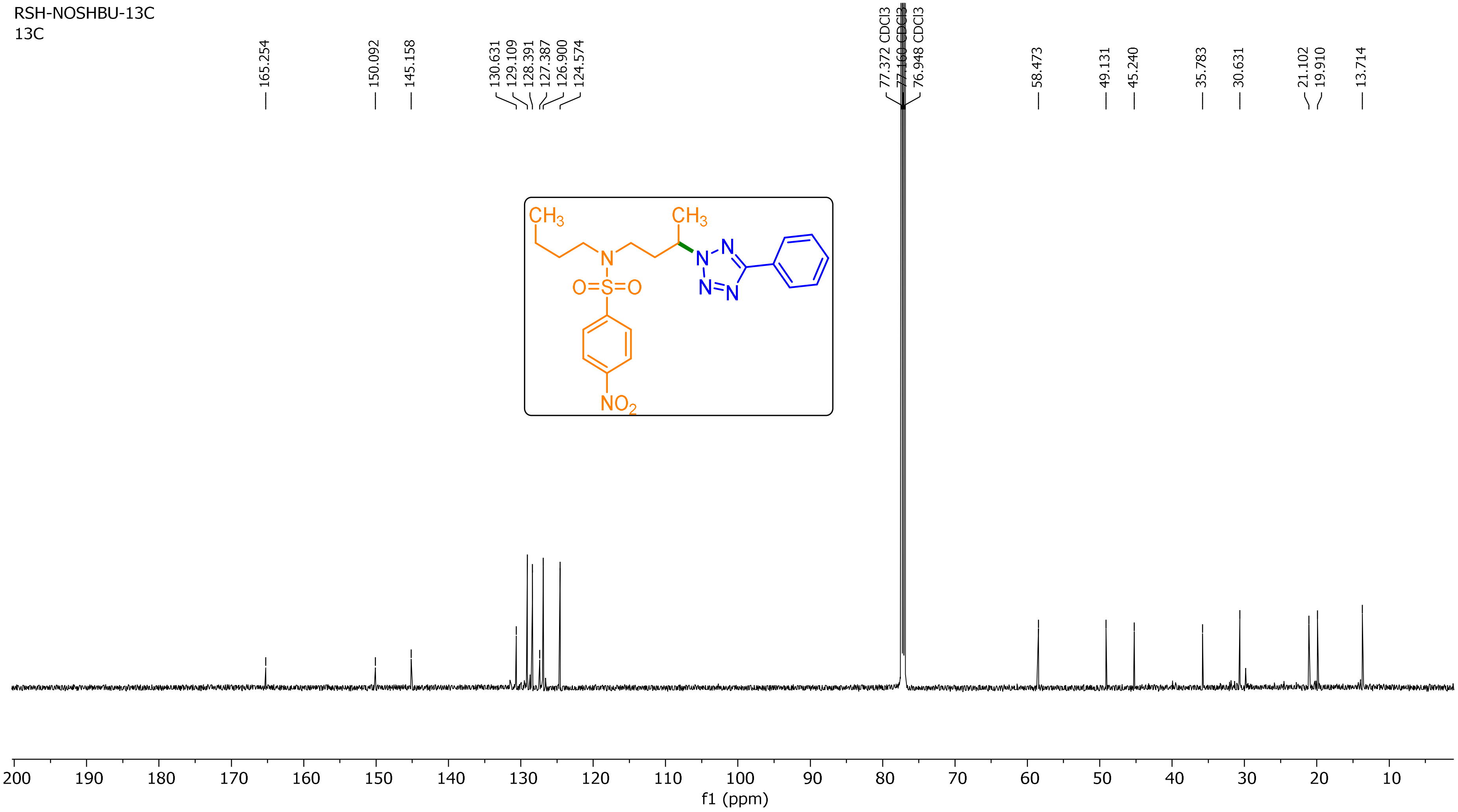

**2-(3-(5-Phenyl-2*H*-tetrazol-2-yl)butyl)isoindoline-1,3-dione (15a): $^{1}$H NMR (400 MHz, $CDCl_3$)**

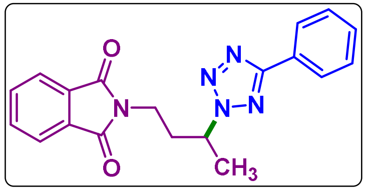

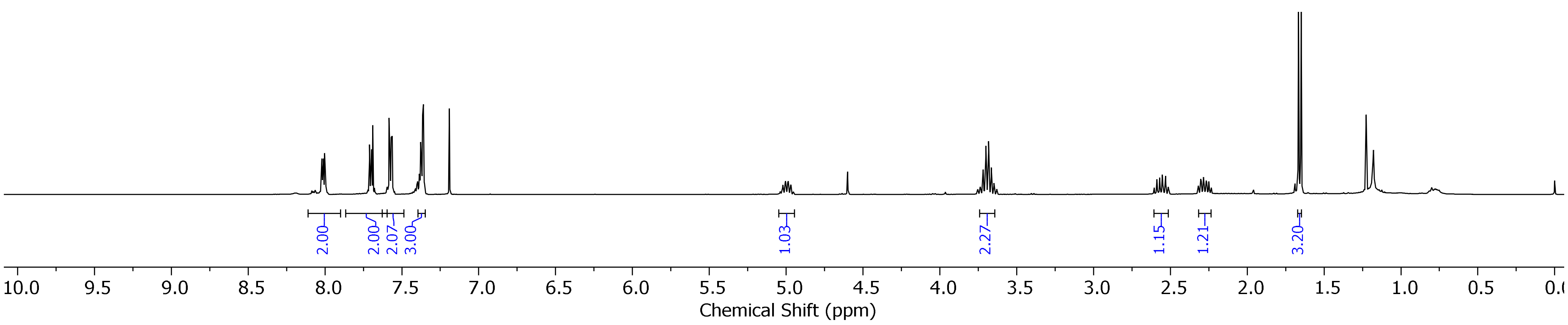

**2-(3-(5-Phenyl-2*H*-tetrazol-2-yl)butyl)isoindoline-1,3-dione (15a): $^{13}C$ NMR (151 MHz, $CDCl_3$)**

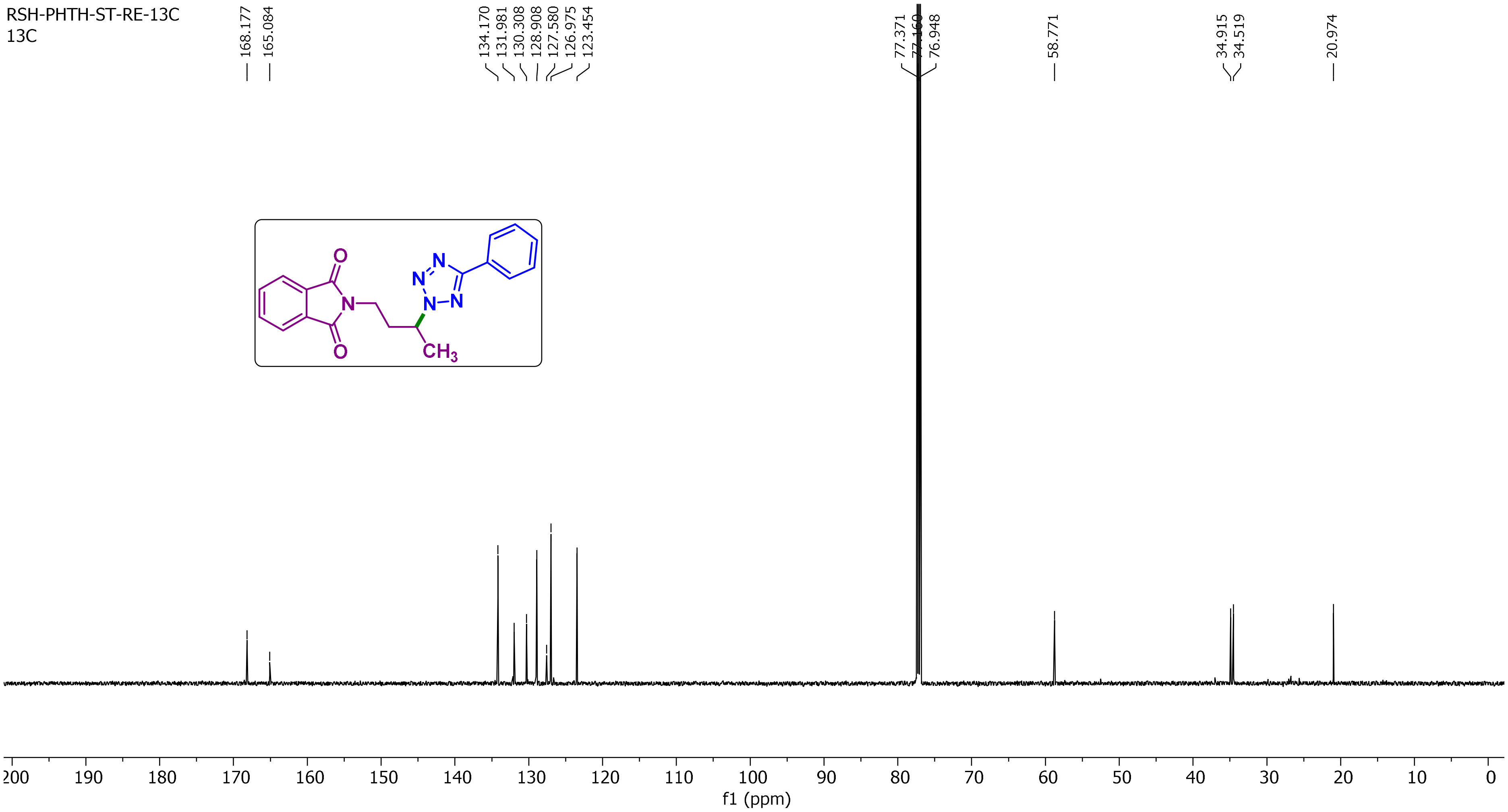

**2-(3-(5-(4-Methoxyphenyl)-2*H*-tetrazol-2-yl)butyl)isoindoline-1,3-dione (15e): $^{1}$H NMR (600 MHz, $CDCl_3$)**

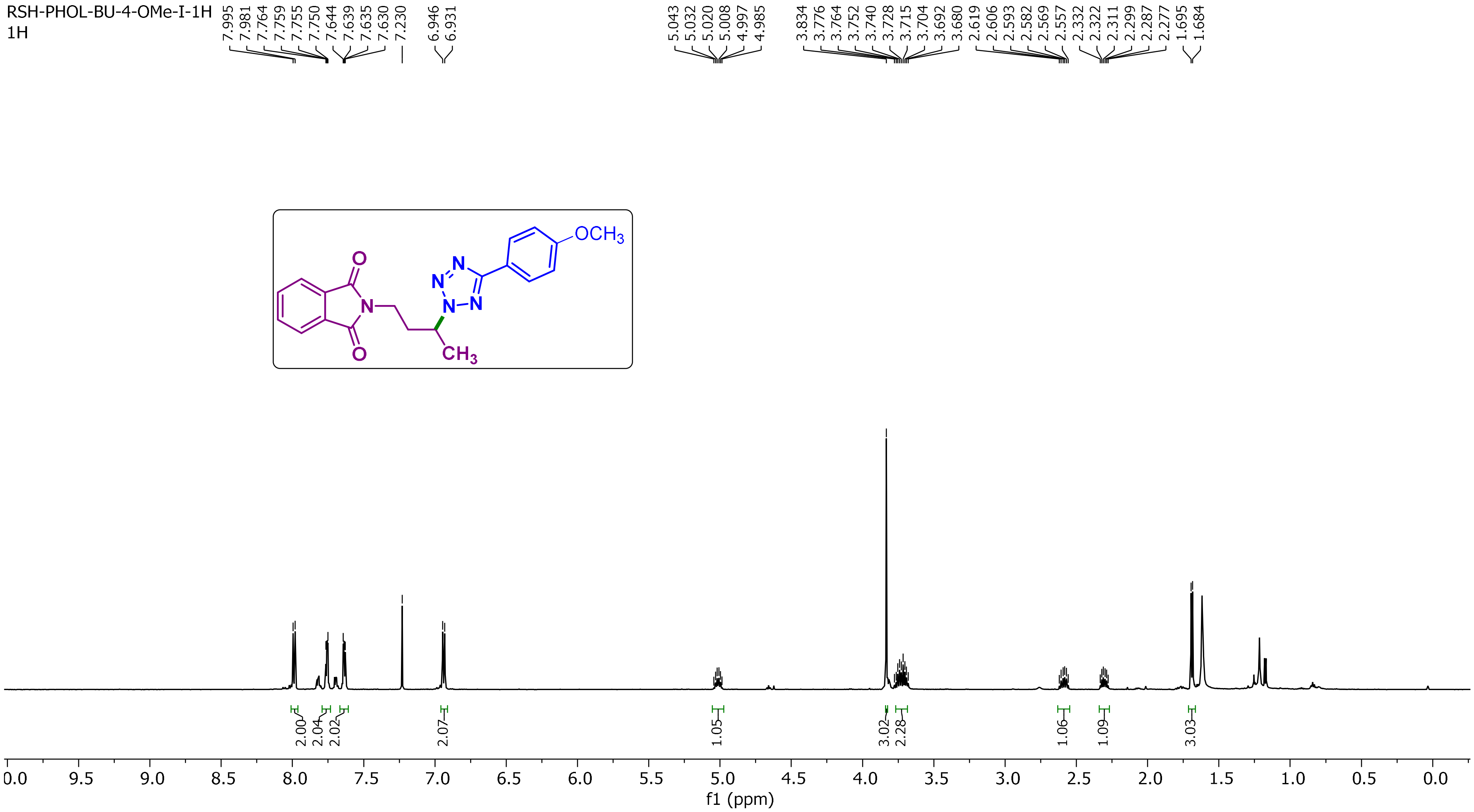

**2-(3-(5-(4-Methoxyphenyl)-2*H*-tetrazol-2-yl)butyl)isoindoline-1,3-dione (15e): $^{13}$C NMR (151 MHz, CDCl$_3$)**

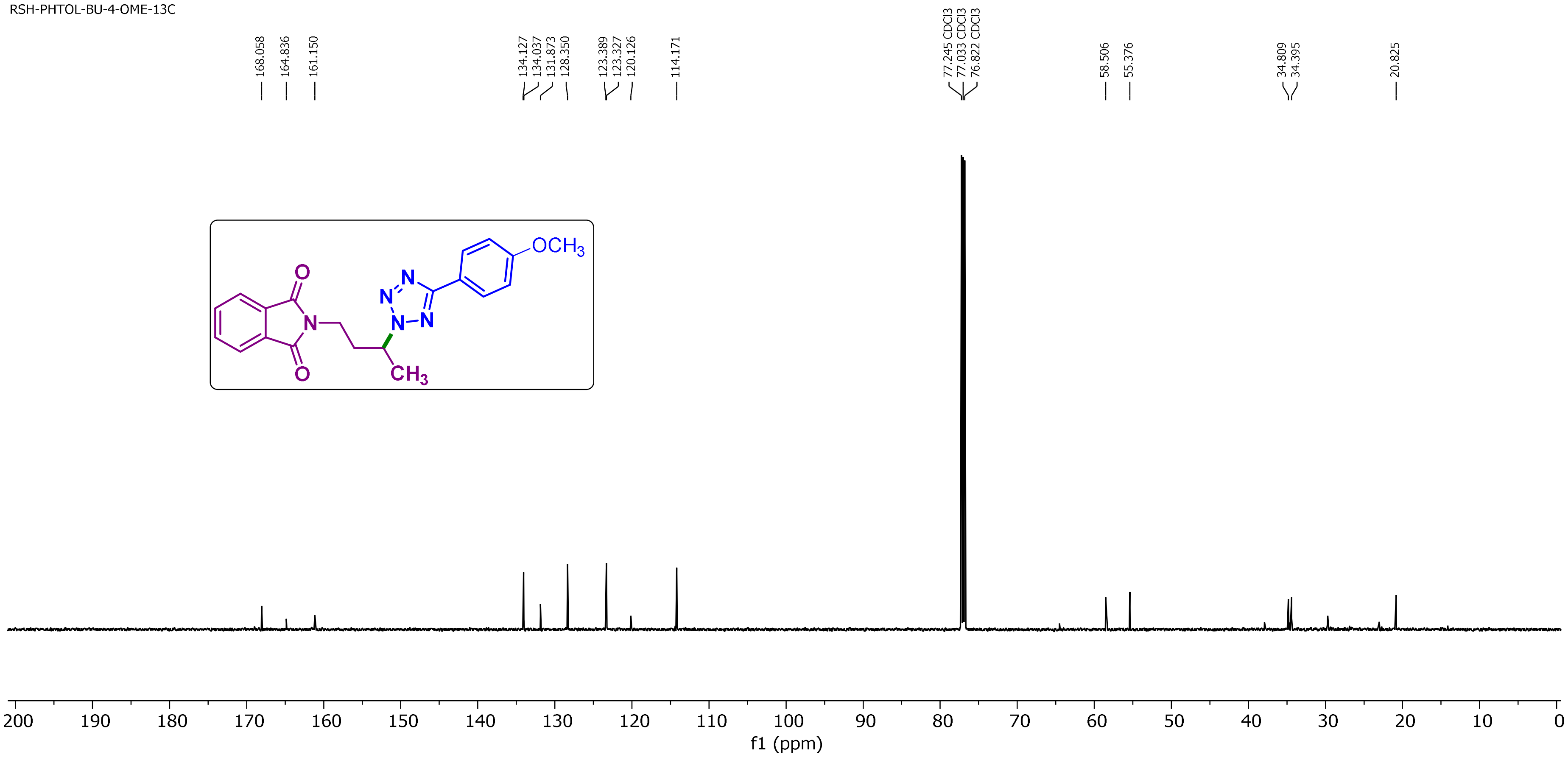

**2-(3-(5-(4-Nitrophenyl)-2*H*-tetrazol-2-yl)butyl)isoindoline-1,3-dione (15k): $^{1}$H NMR (600 MHz, $CDCl_3$)**

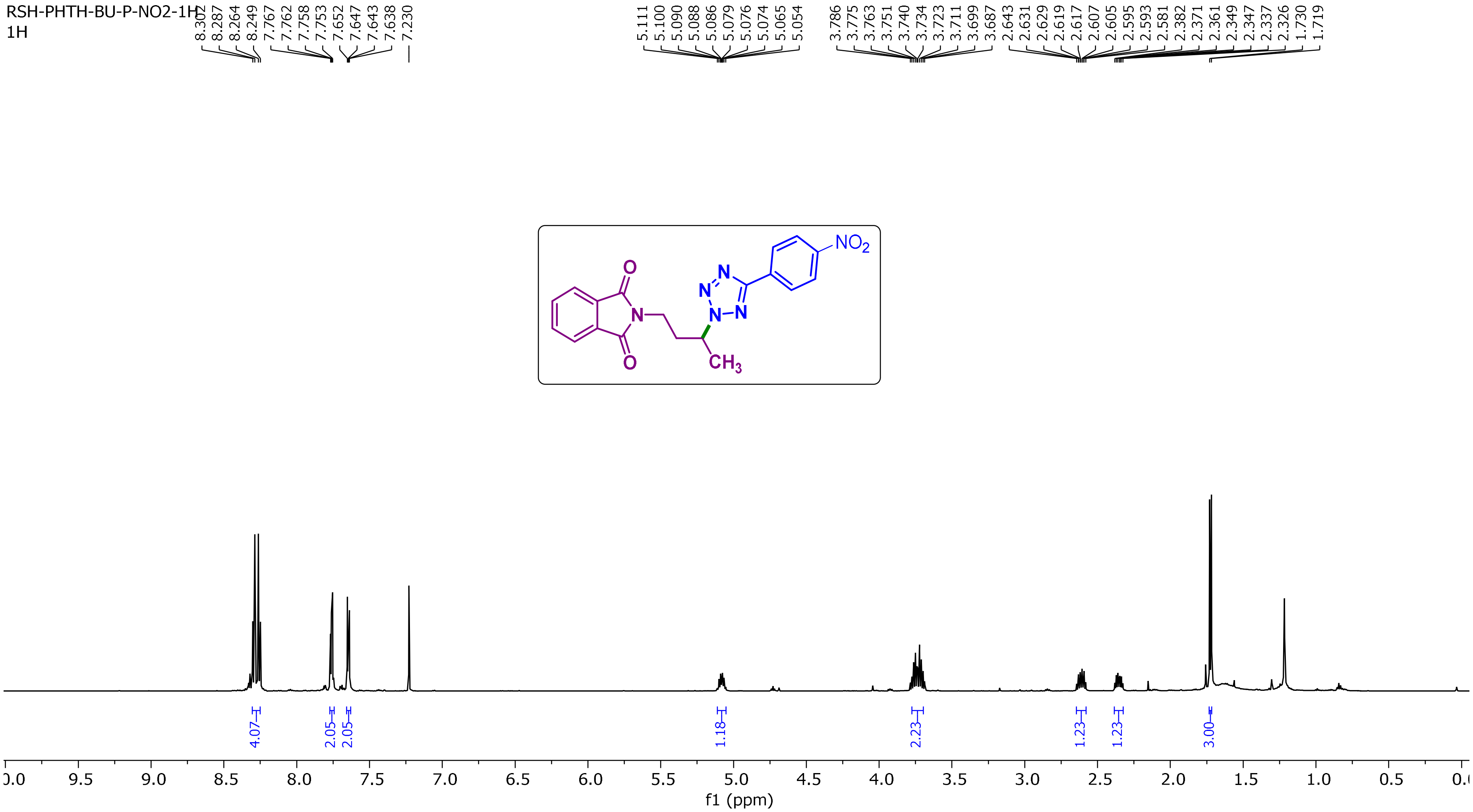

**2-(3-(5-(4-Nitrophenyl)-2*H*-tetrazol-2-yl)butyl)isoindoline-1,3-dione (15k): $^{13}$C NMR (151 MHz, $CDCl_3$)**

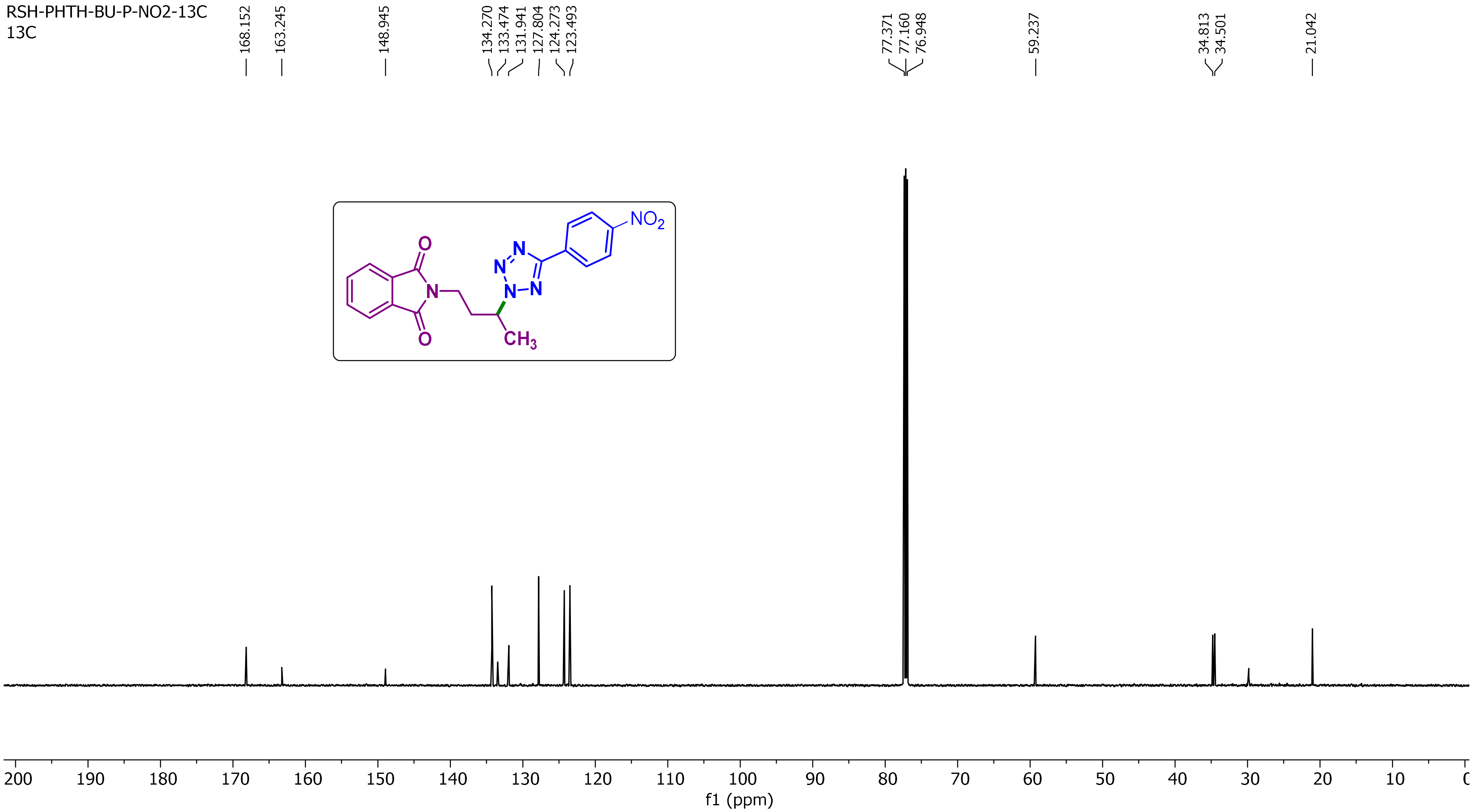

**5-Nitro-2-(3-(5-phenyl-2*H*-tetrazol-2-yl)butyl)benzo[*d*]isothiazol-3(2*H*)-one 1,1-dioxide (16a): $^{1}$H NMR (600 MHz, CDCl₃)**

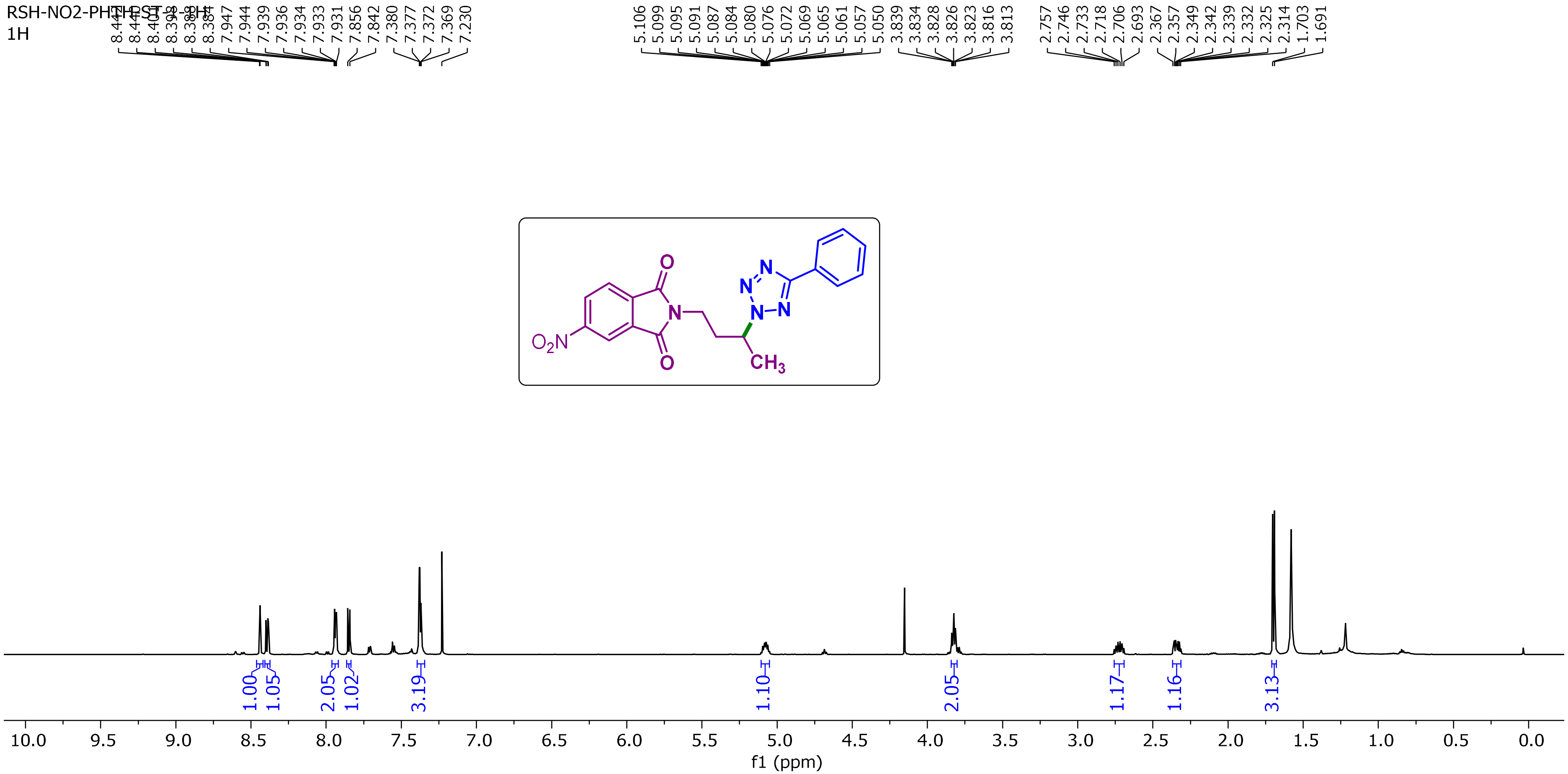

**5-Nitro-2-(3-(5-phenyl-2*H*-tetrazol-2-yl)butyl)benzo[*d*]isothiazol-3(2*H*)-one 1,1-dioxide (16a): $^{13}$C NMR (151 MHz, CDCl$_3$)**

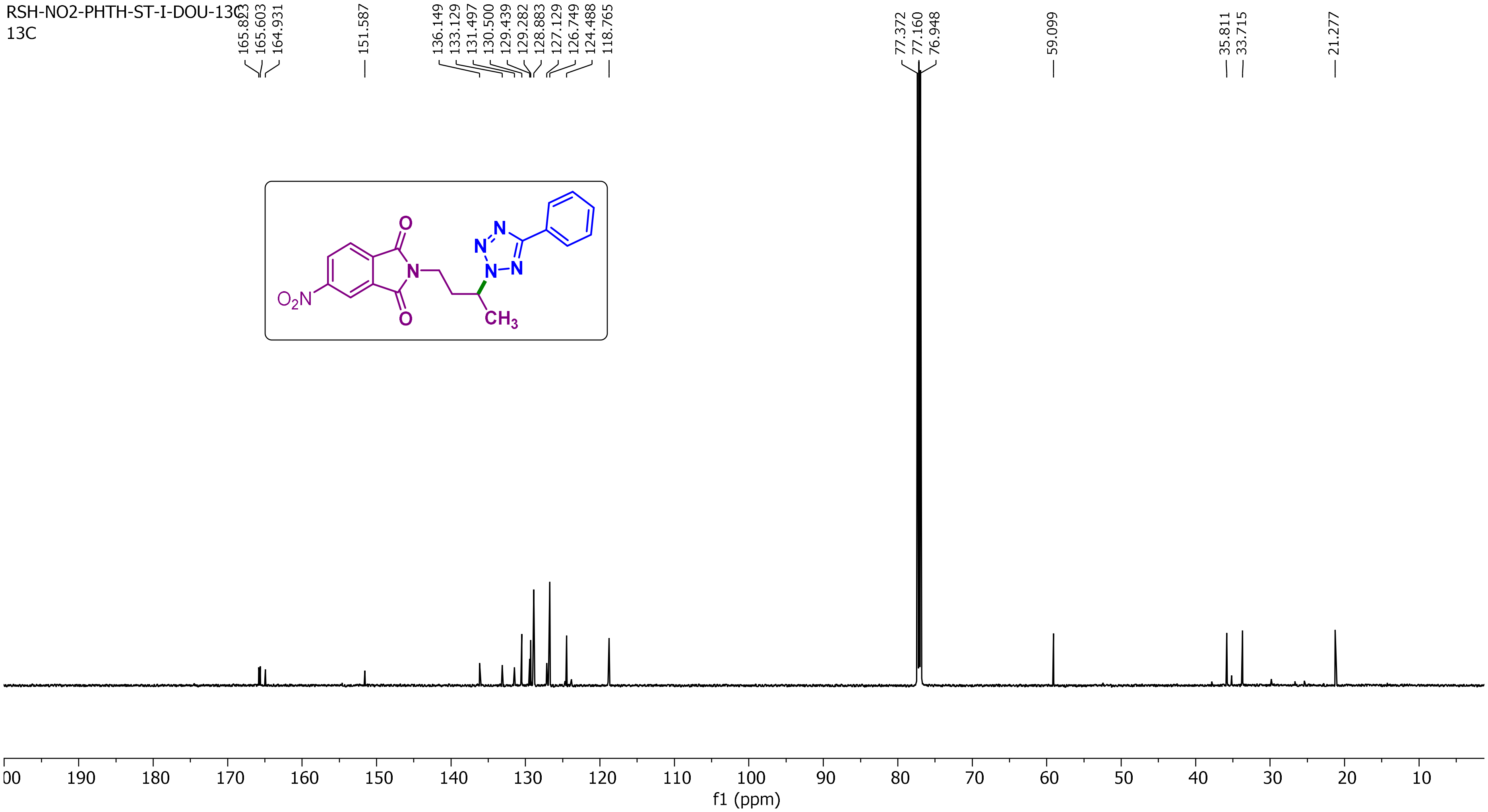

**2-(4-(5-Phenyl-2*H*-tetrazol-2-yl)pentyl)isoindoline-1,3-dione (17a) + 2-(3-(5-Phenyl-2*H*-tetrazol-2-yl)pentyl)isoindoline-1,3-dione (17'a): $^{1}$H NMR (600 MHz, $CDCl_3$)**

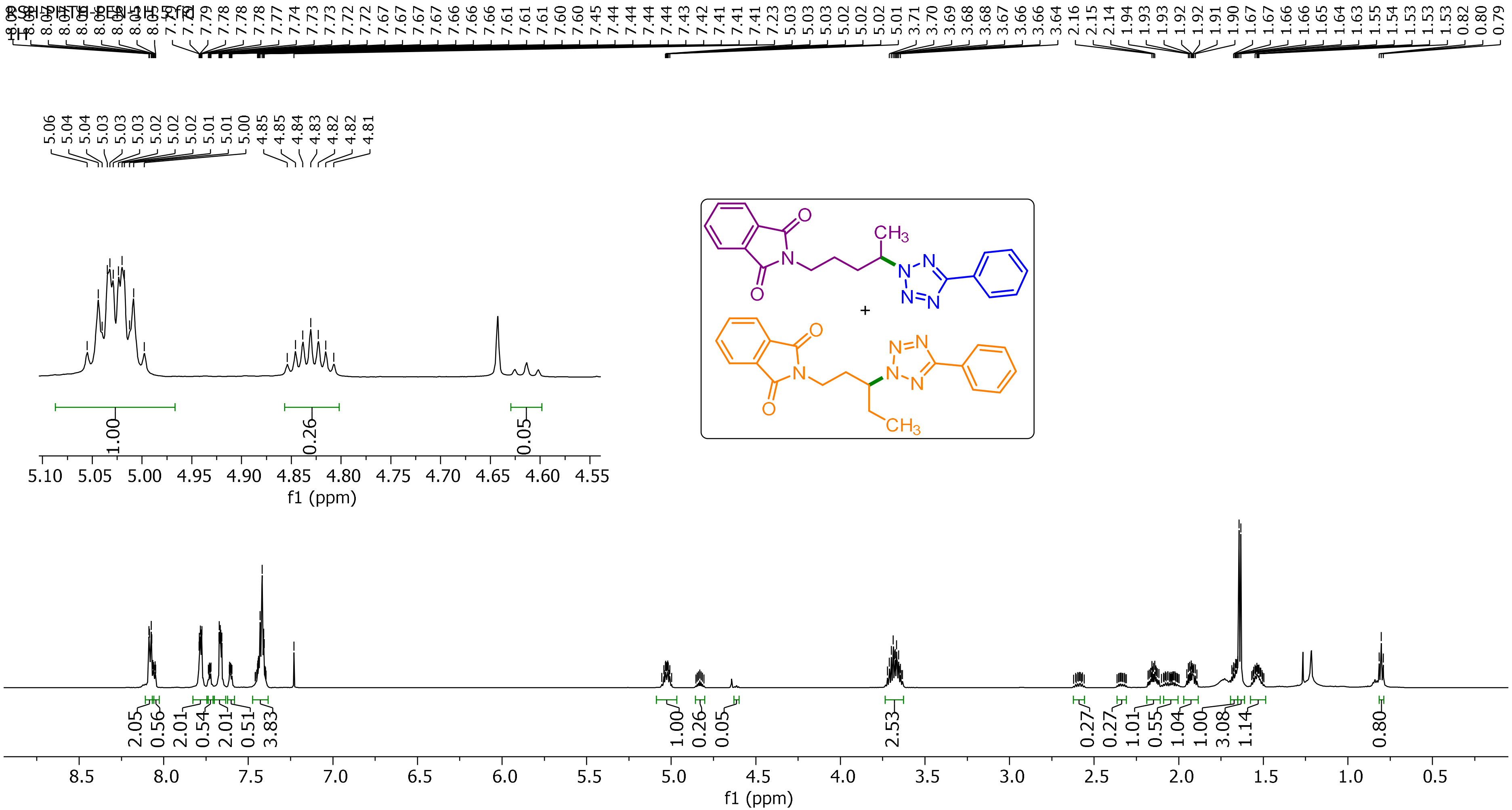

**2-(4-(5-Phenyl-2*H*-tetrazol-2-yl)pentyl)isoindoline-1,3-dione (17a) + 2-(3-(5-Phenyl-2*H*-tetrazol-2-yl)pentyl)isoindoline-1,3-dione (17'a):**

**$^{13}C$ NMR (151 MHz, $CDCl_3$)**

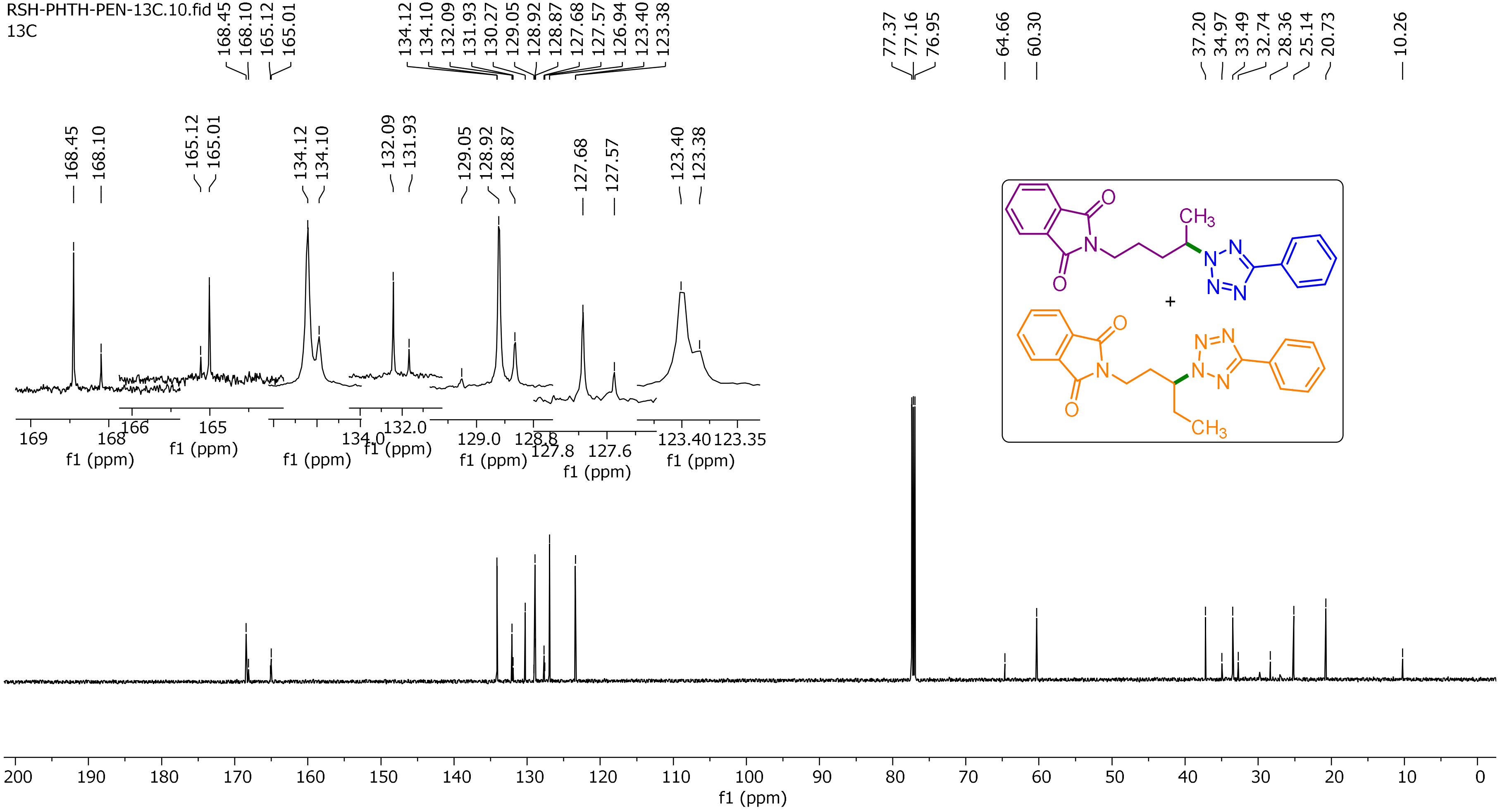

**2-(5-(5-Phenyl-2*H*-tetrazol-2-yl)hexyl)isoindoline-1,3-dione (18a):** $^{1}$**H NMR (600 MHz, CDCl$_3$)**

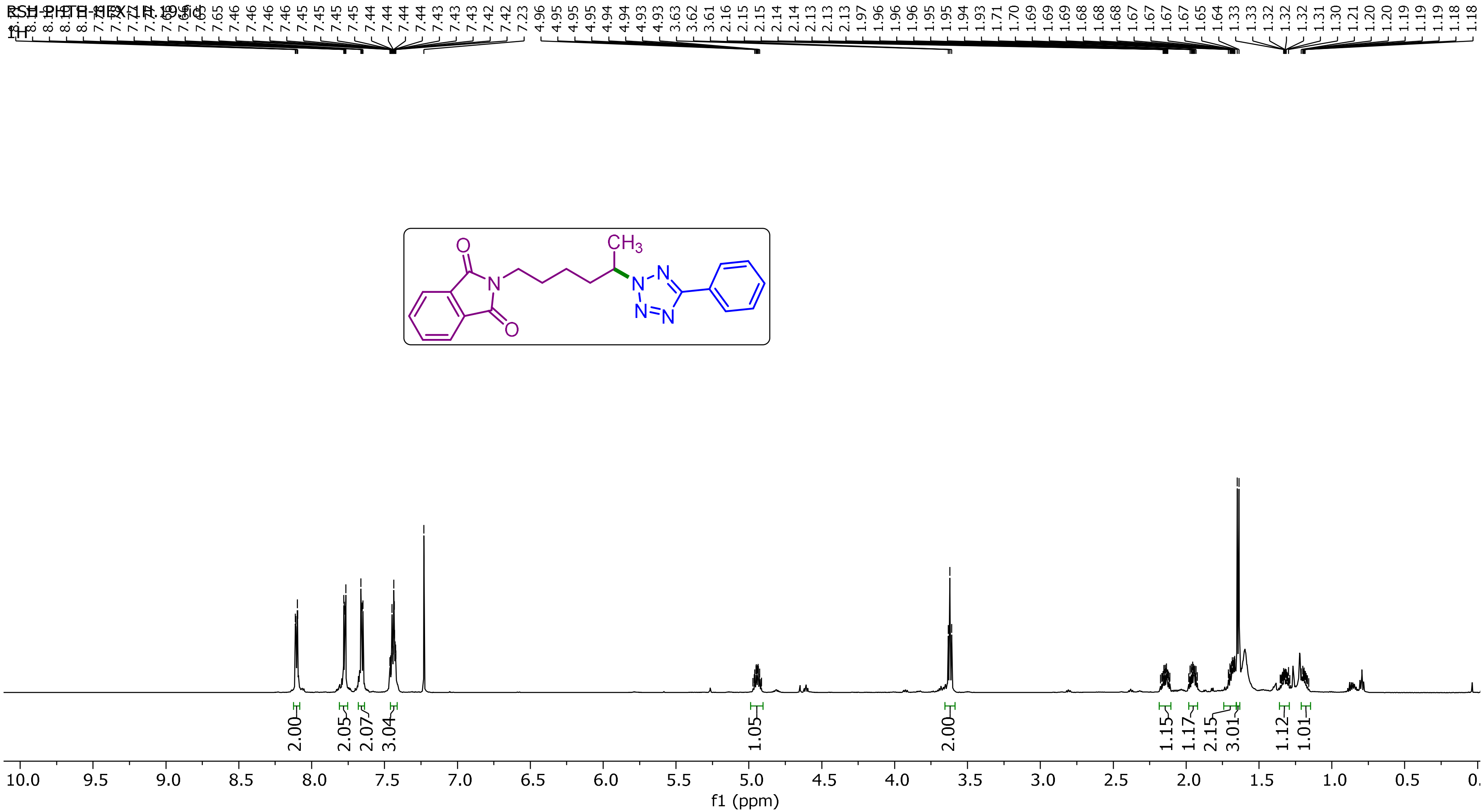

**2-(5-(5-Phenyl-2*H*-tetrazol-2-yl)hexyl)isoindoline-1,3-dione (18a): $^{13}$C NMR (151 MHz, $CDCl_3$)**

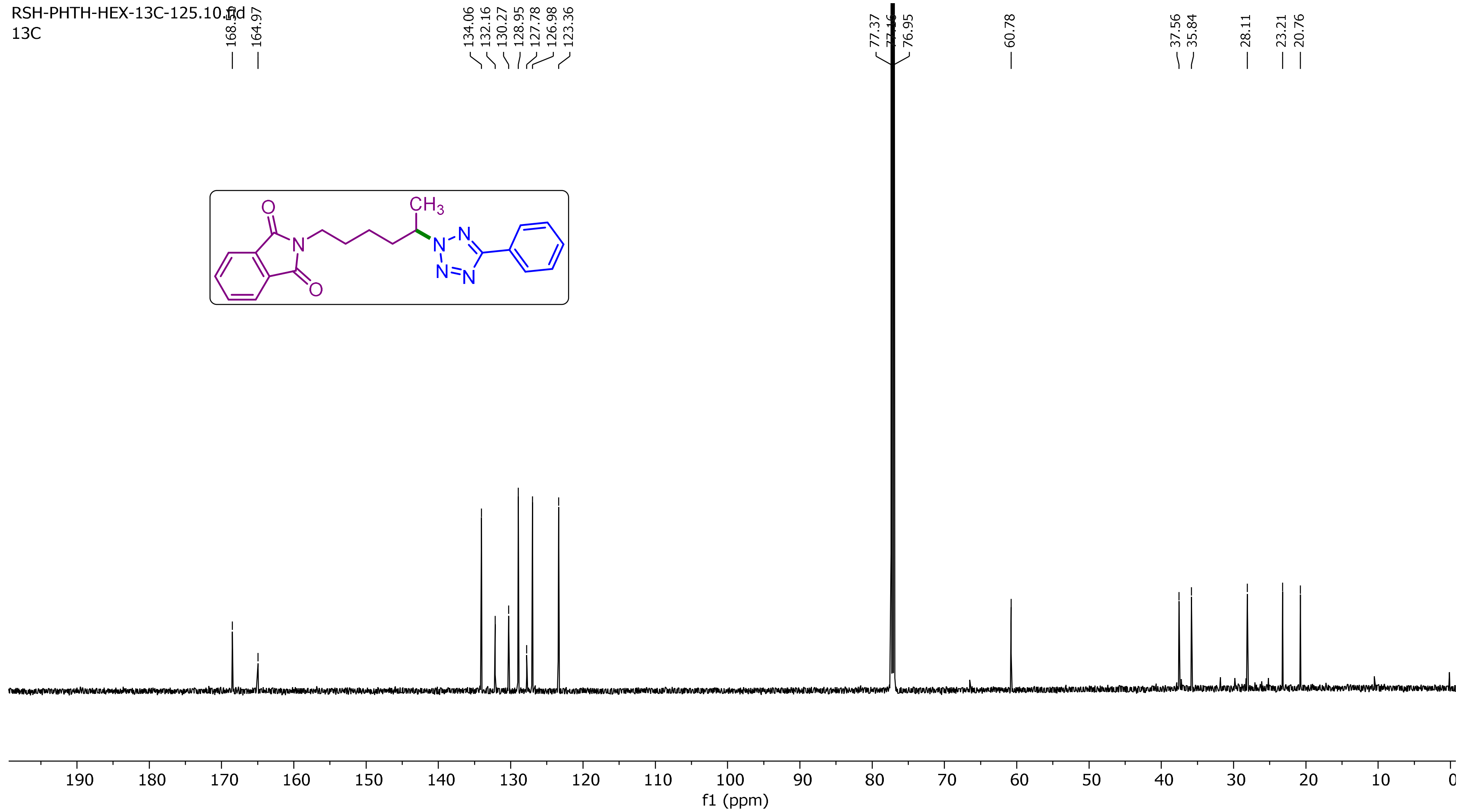

**2-(4-((4-Nitrophenyl)sulfonyl)butan-2-yl)-5-phenyl-2*H*-tetrazole (20a): $^{1}$H NMR (600 MHz, CDCl₃)**

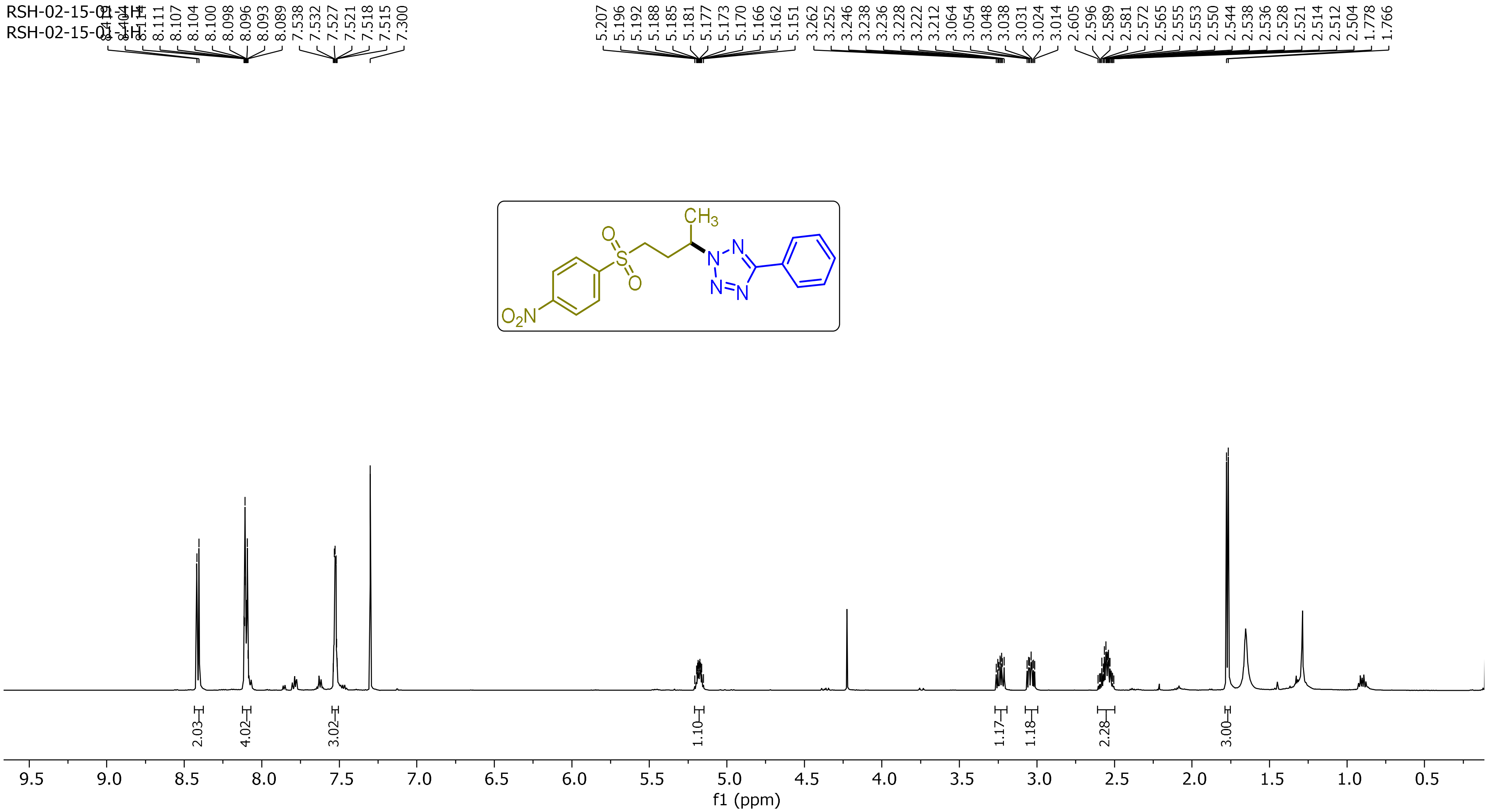

**2-(4-((4-Nitrophenyl)sulfonyl)butan-2-yl)-5-phenyl-2*H*-tetrazole (20a): $^{13}$C NMR (151 MHz, CDCl$_3$)**

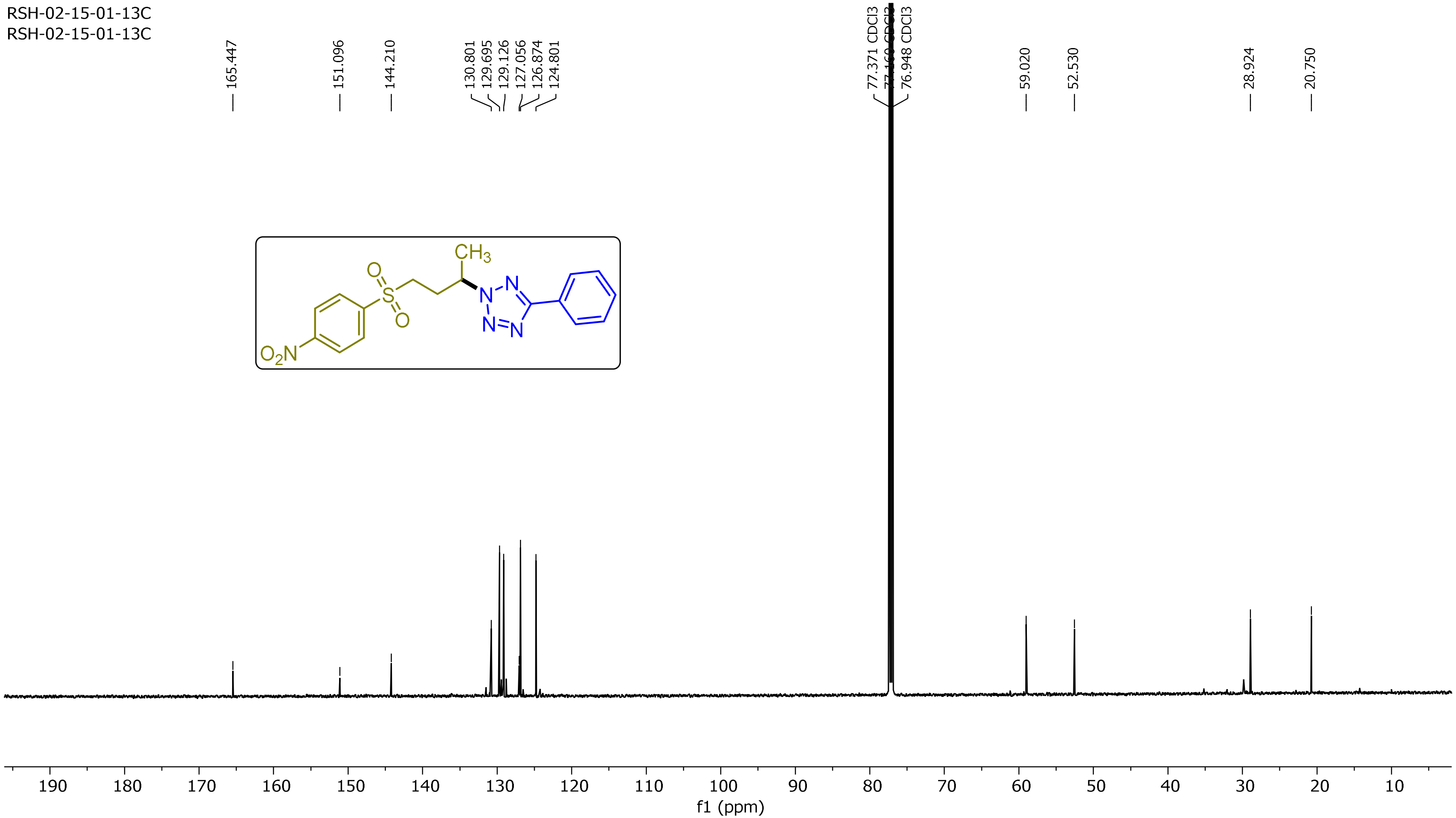

**2-(4-(Butylsulfonyl)butan-2-yl)-5-phenyl-2*H*-tetrazole (21a): $^1$H NMR (600 MHz, $CDCl_3$)**

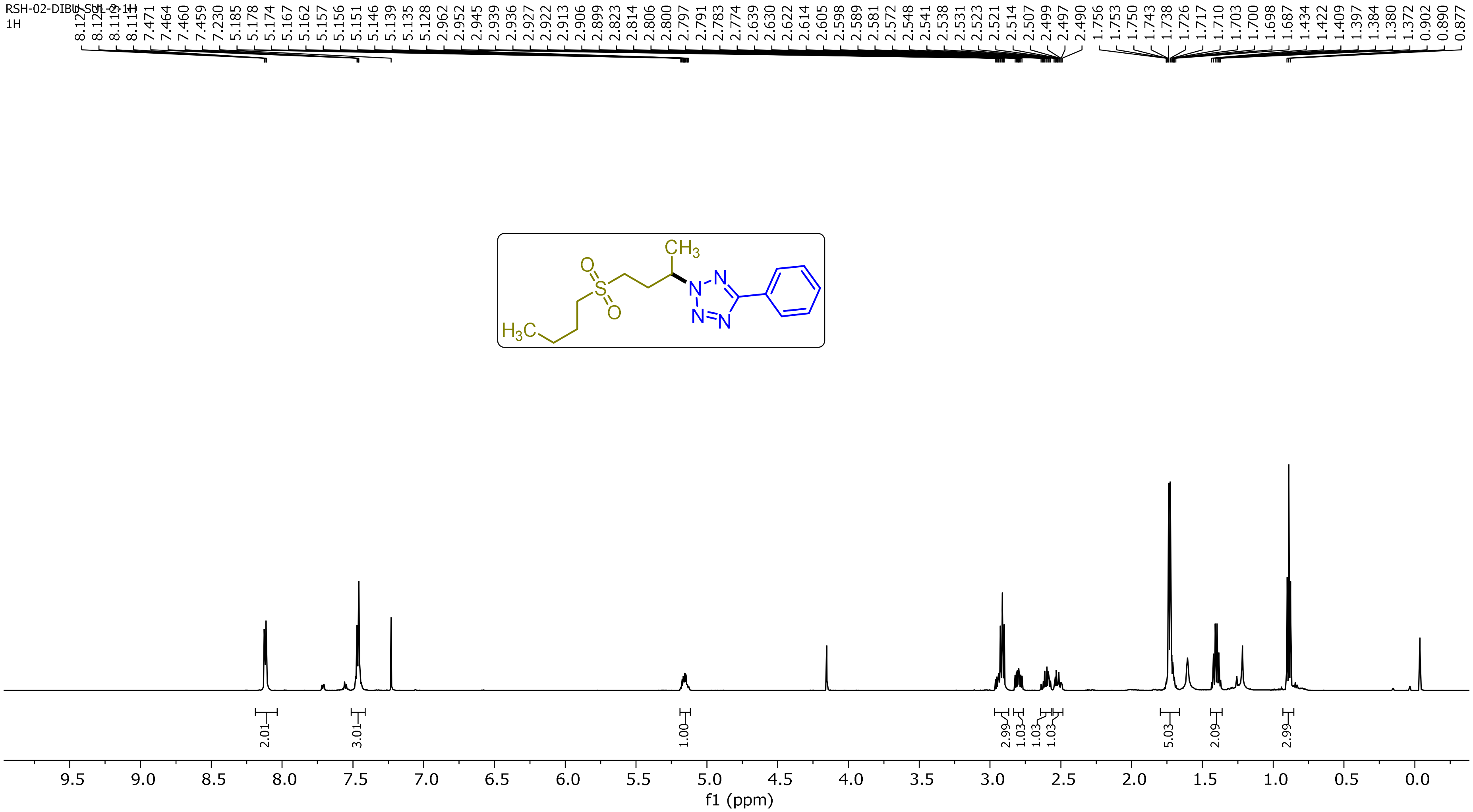

## 2-(4-(Butylsulfonyl)butan-2-yl)-5-phenyl-2*H*-tetrazole (21a): $^{13}C$ NMR (151 MHz, $CDCl_3$)

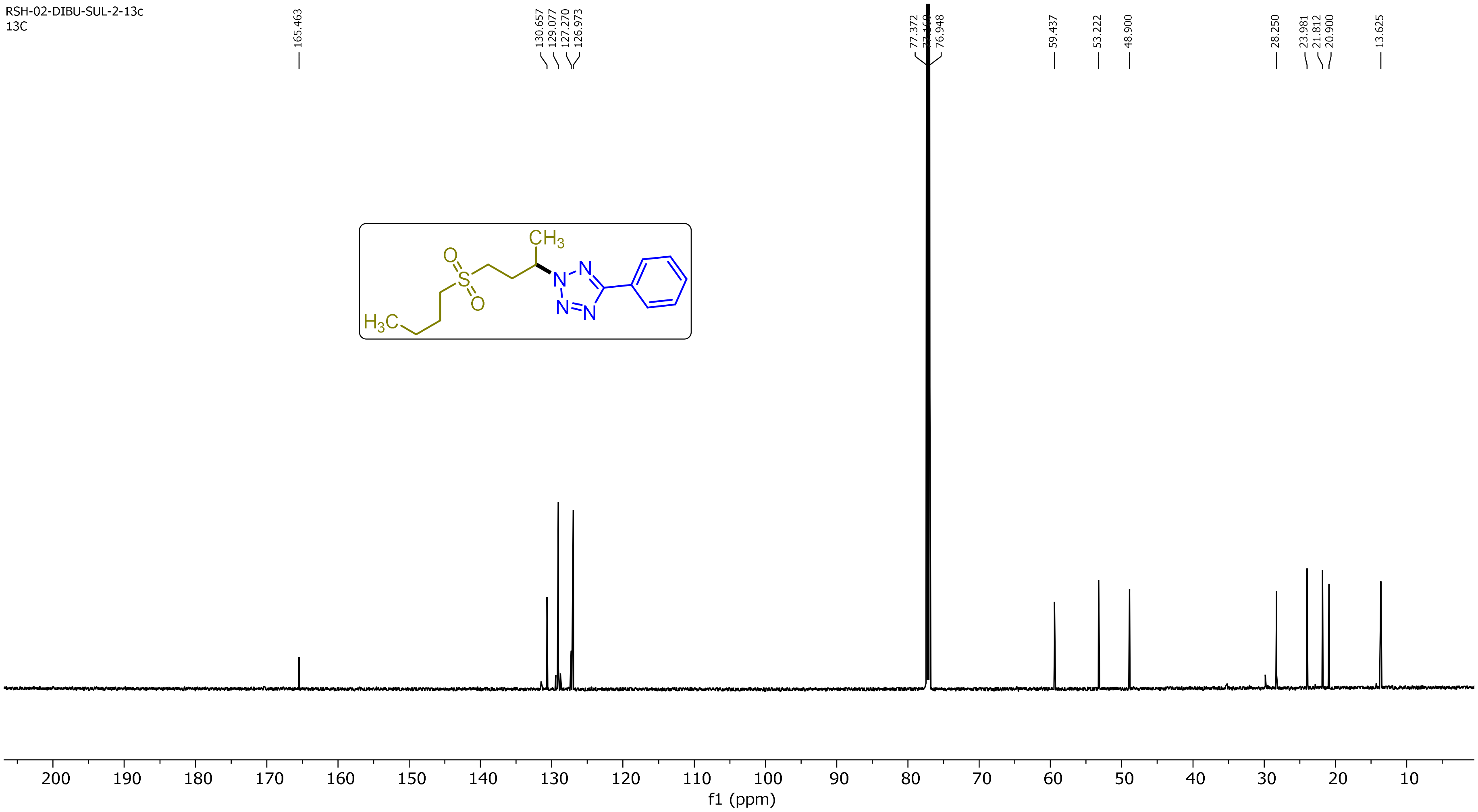

**2-(3-(5-Phenyl-2*H*-tetrazol-2-yl)butyl)benzo[*d*]isothiazol-3(2*H*)-one 1,1-dioxide (22a): $^{1}$H NMR (600 MHz, CDCl₃)**

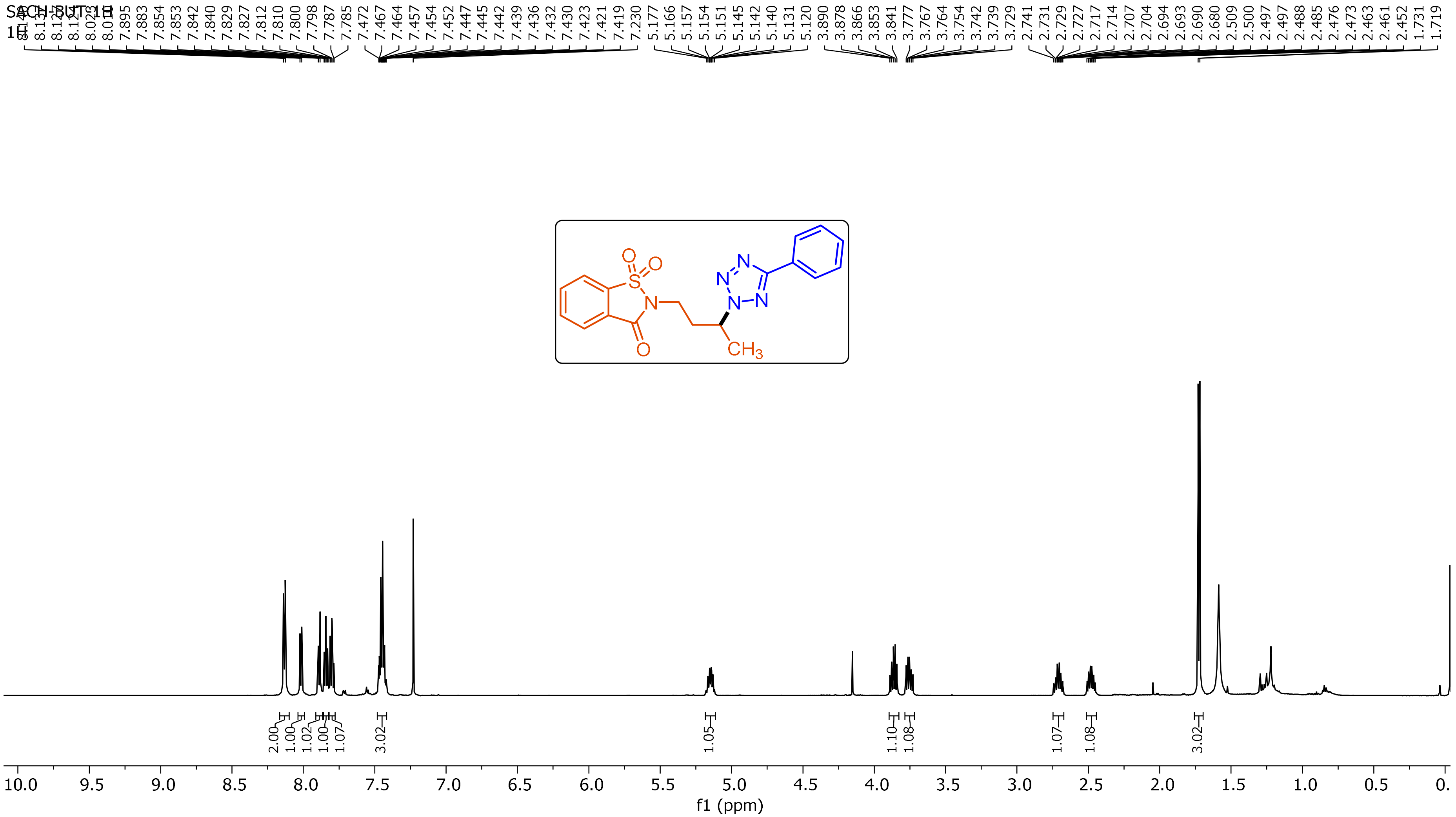

**2-(3-(5-Phenyl-2*H*-tetrazol-2-yl)butyl)benzo[*d*]isothiazol-3(2*H*)-one 1,1-dioxide (22a): $^{13}$C NMR (151 MHz, CDCl$_3$)**

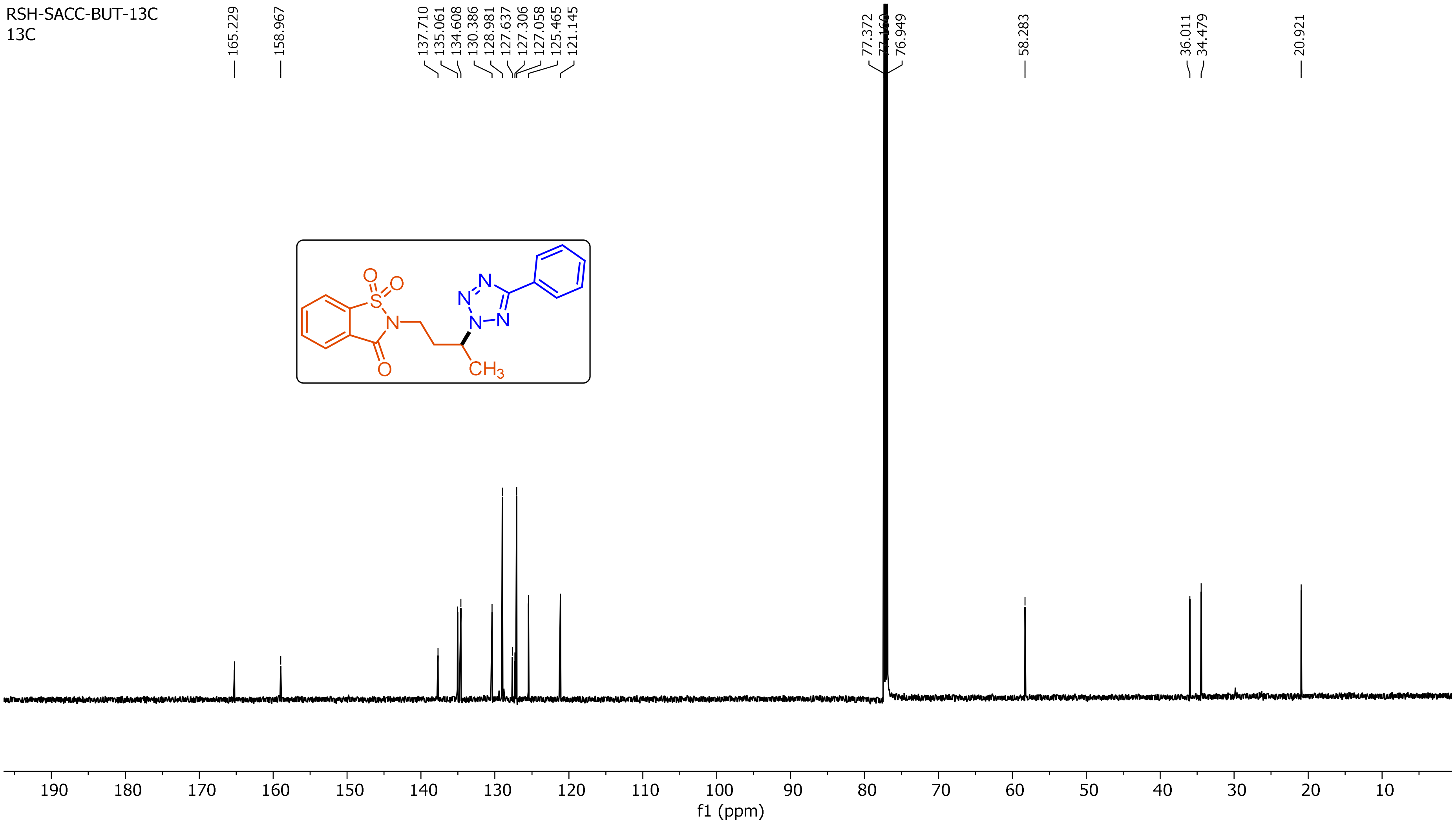

**Methyl 5-(5-phenyl-2*H*-tetrazol-2-yl)hexanoate (24a): $^{1}$H NMR (600 MHz, $CDCl_3$)**

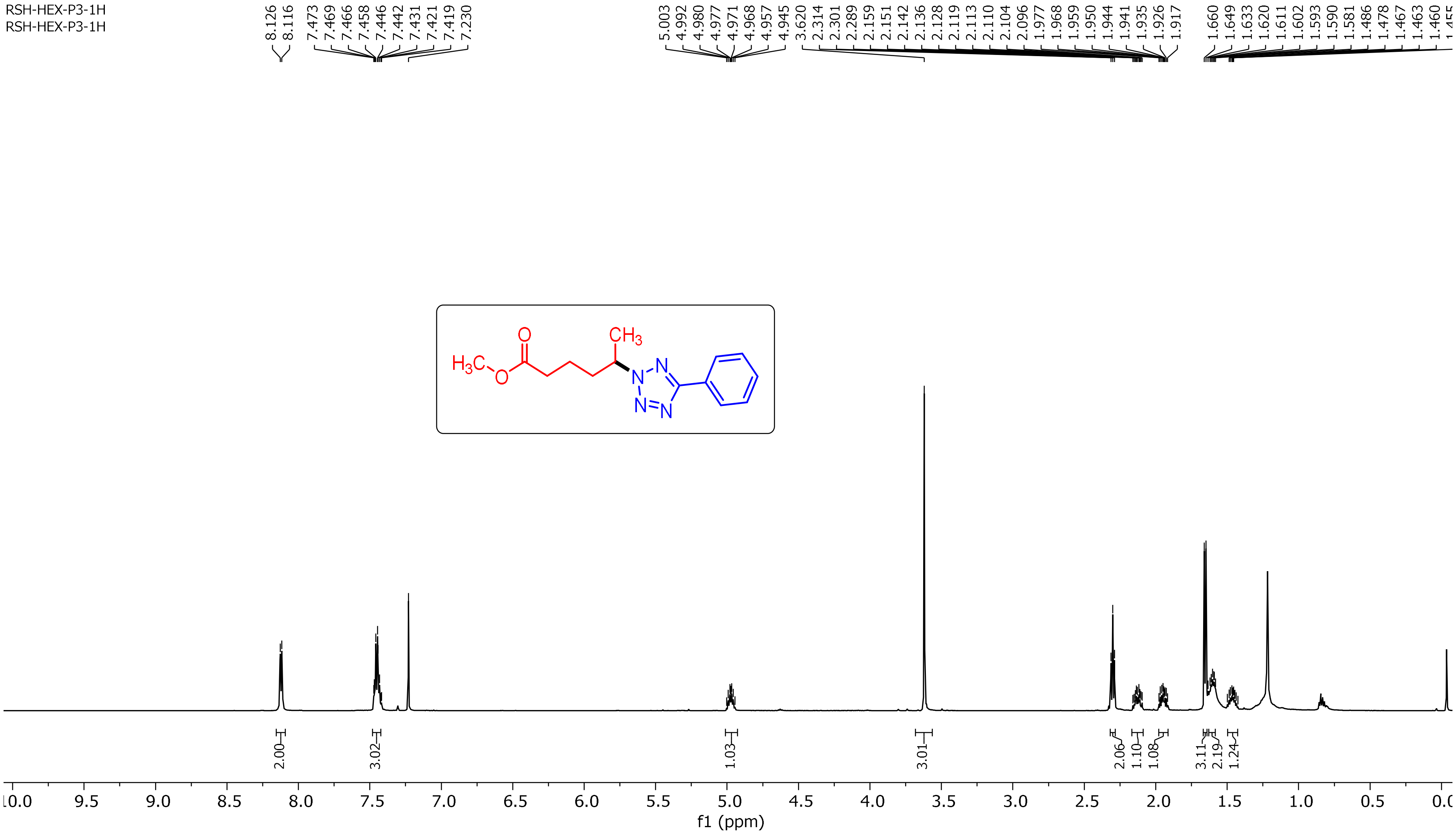

**Methyl 5-(5-phenyl-2*H*-tetrazol-2-yl)hexanoate (24a): $^{13}$C NMR (151 MHz, CDCl$_3$)**

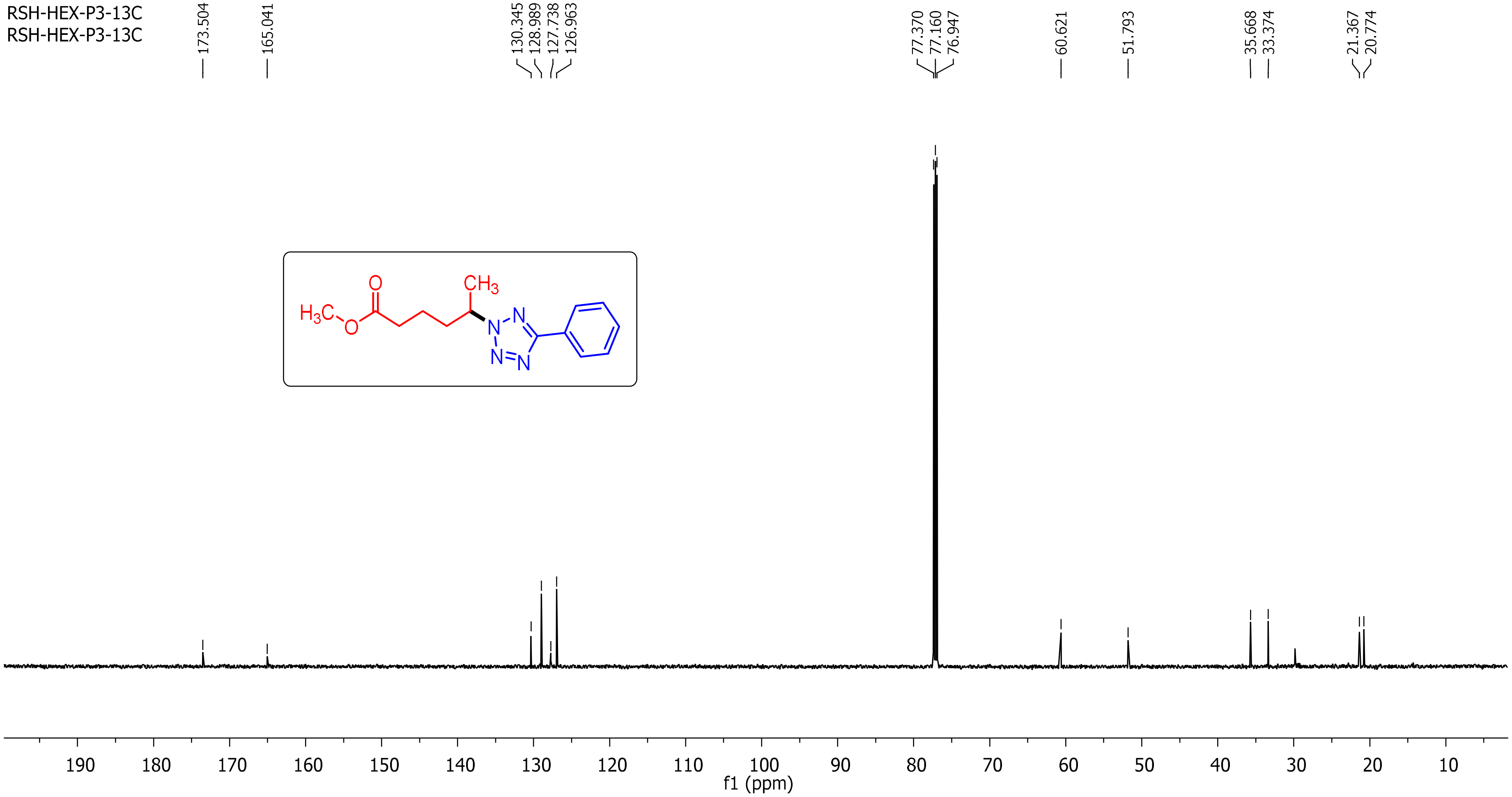

**4-(5-Phenyl-2*H*-tetrazol-2-yl)pentanenitrile (25a): $^{1}H$ NMR (600 MHz, $CDCl_3$)**

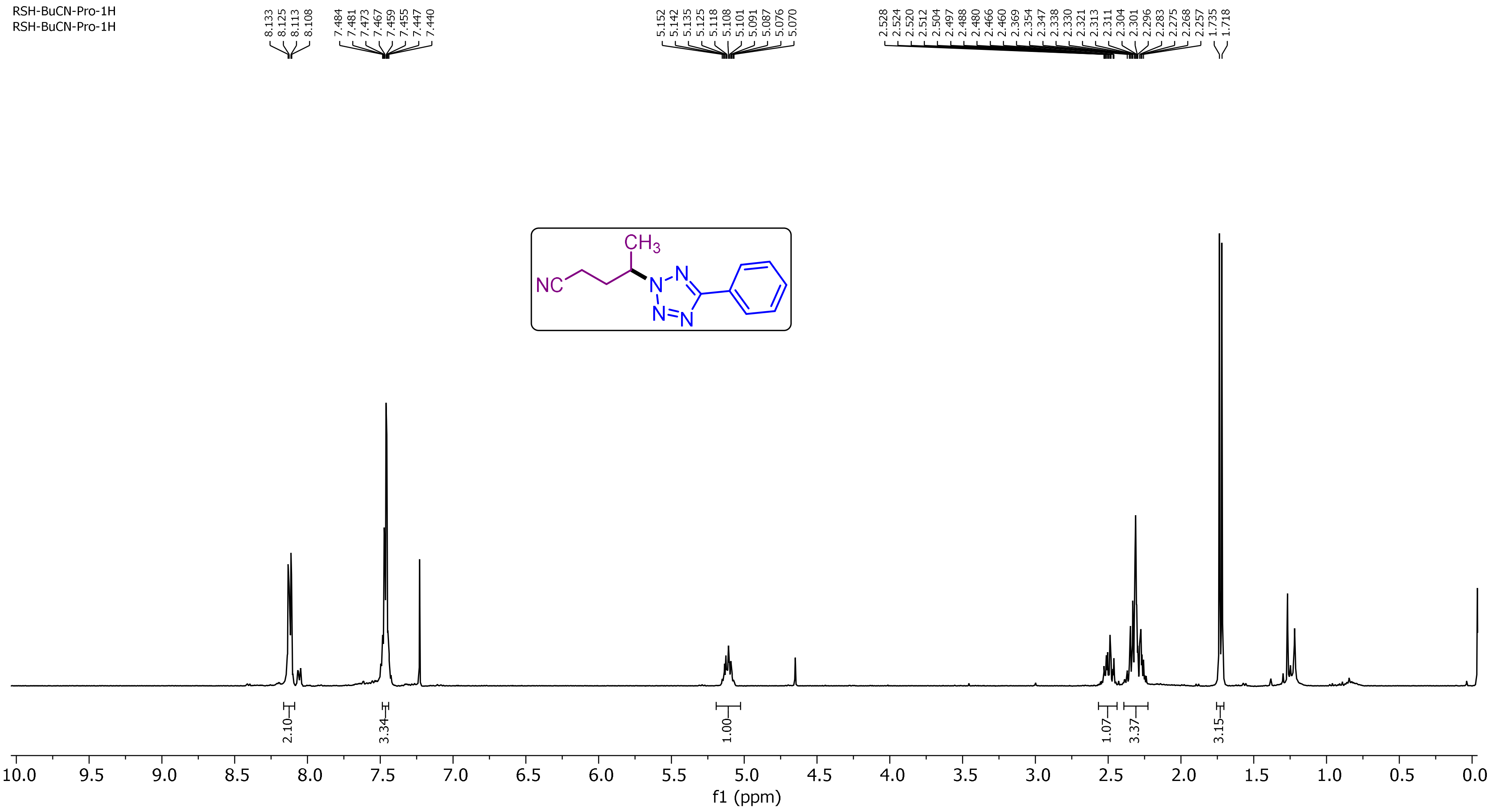

## 4-(5-Phenyl-2*H*-tetrazol-2-yl)pentanenitrile (25a): $^{13}$C NMR (151 MHz, $CDCl_3$)

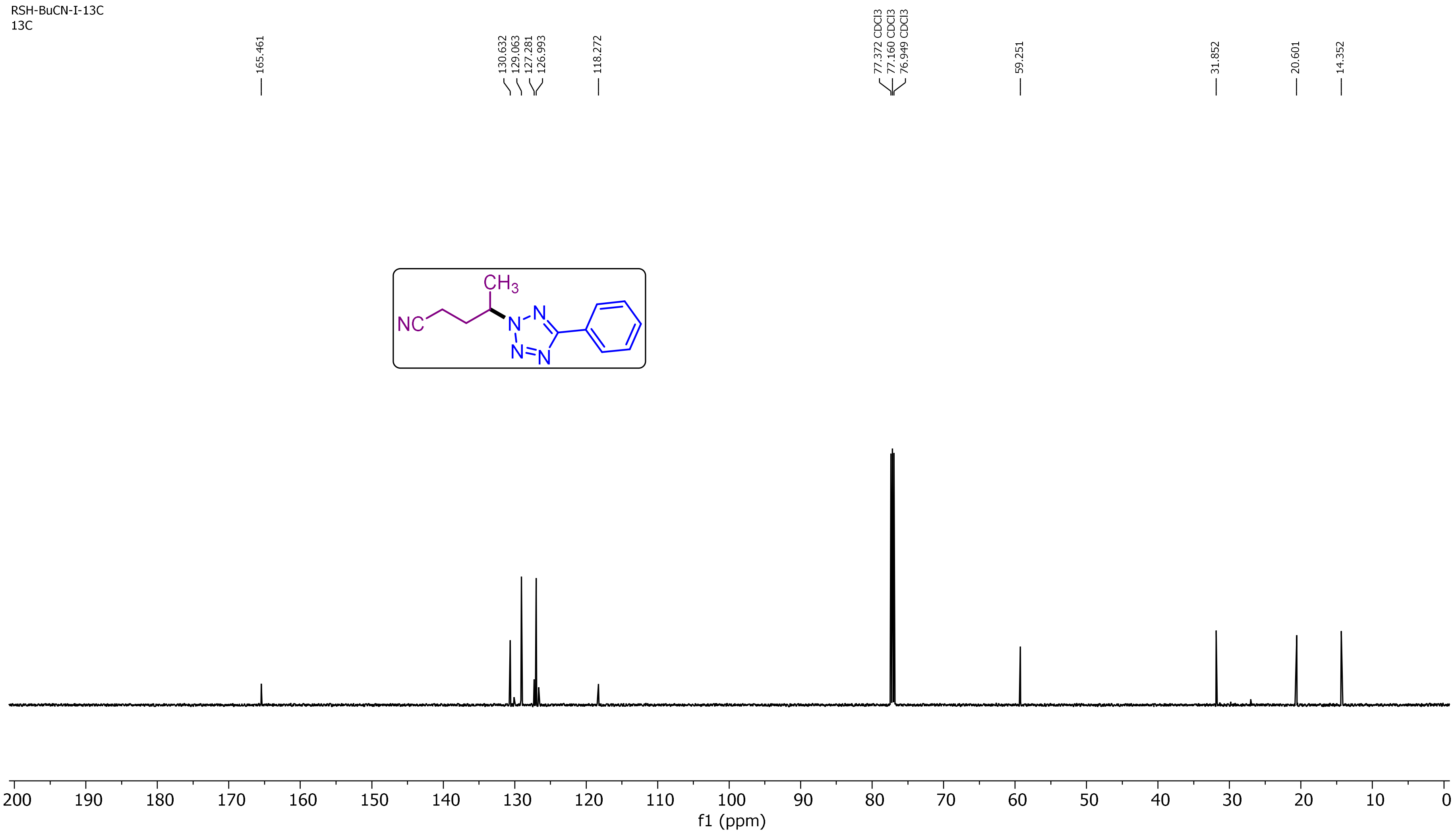

**Dibutyl (3-(5-phenyl-2*H*-tetrazol-2-yl)butyl) phosphate (27a): $^1$H NMR (600 MHz, $CDCl_3$)**

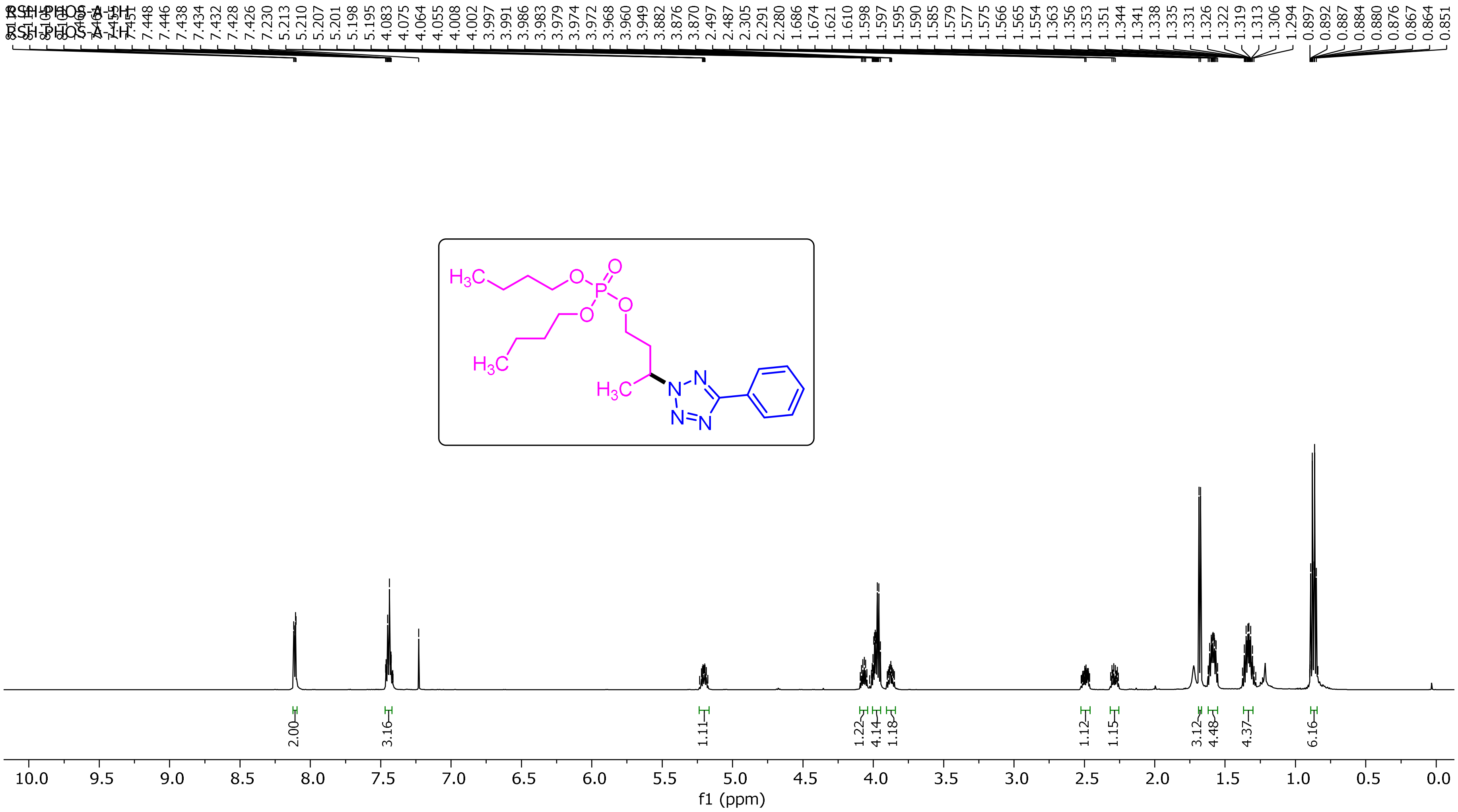

**Dibutyl (3-(5-phenyl-2*H*-tetrazol-2-yl)butyl) phosphate (27a): $^{13}C$ NMR (151 MHz, $CDCl_3$)**

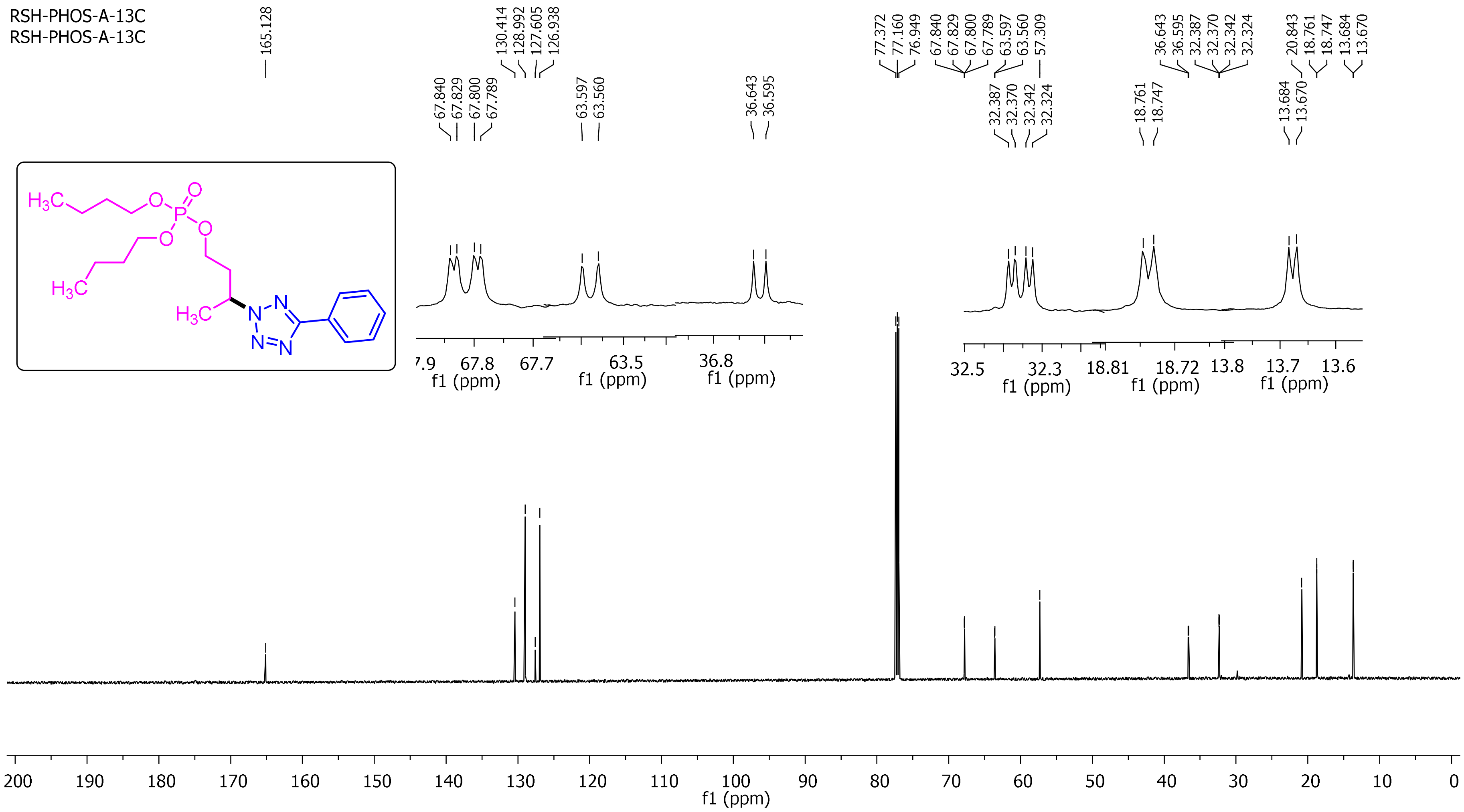

**Dibutyl (3-(5-phenyl-2*H*-tetrazol-2-yl)butyl) phosphate (27a): $^{31}$P {$^{1}$H} NMR (162 MHz, CDCl$_3$)**

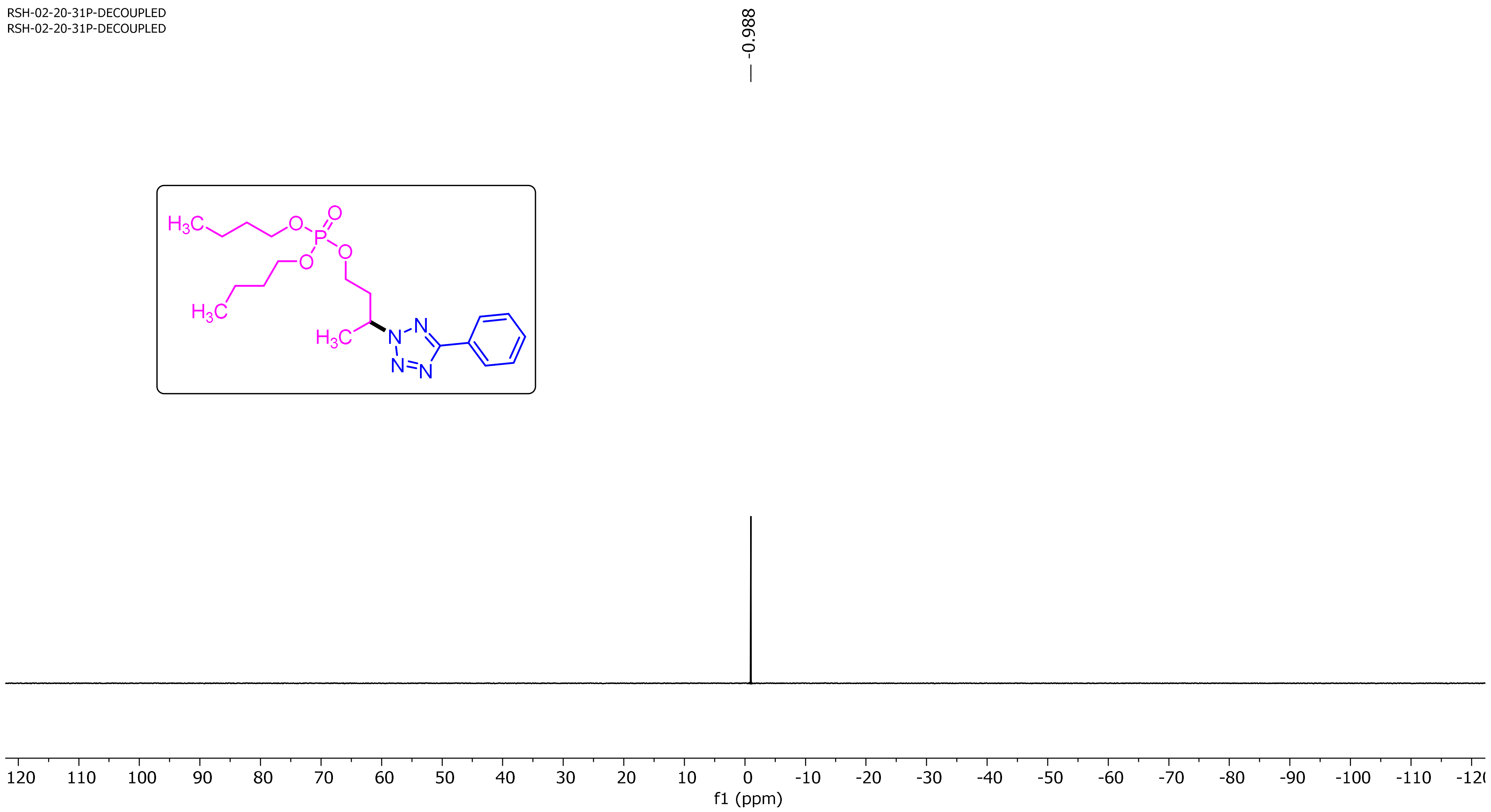

**Dibutyl (3-(5-phenyl-2*H*-tetrazol-2-yl)butyl) phosphate (27a): $^{31}$P NMR (162 MHz, CDCl$_3$)**

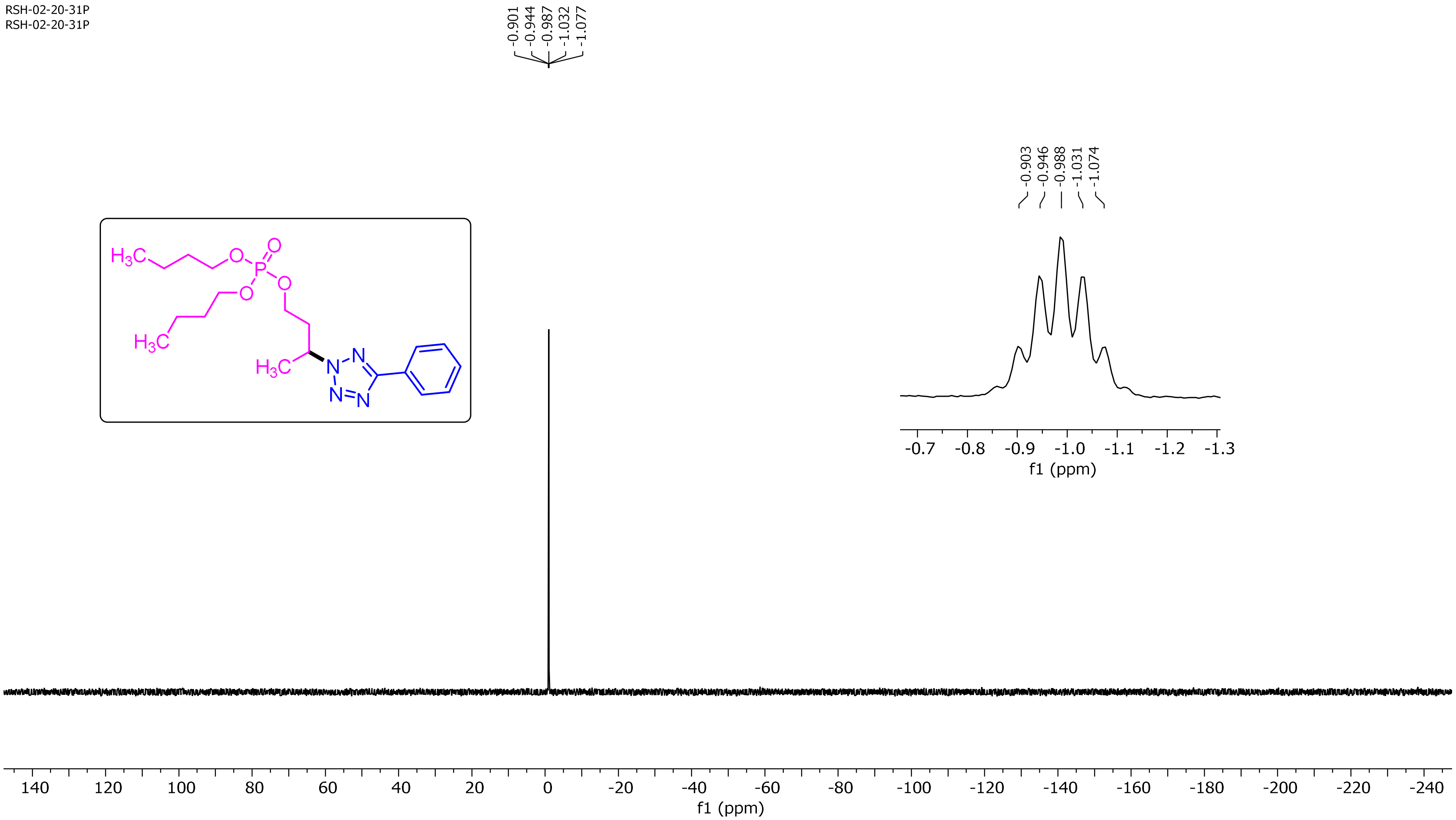

**Dibutyl (3-(5-(4-methoxyphenyl)-2*H*-tetrazol-2-yl)butyl) phosphate (27e): $^{1}$H NMR (600 MHz, CDCl$_3$)**

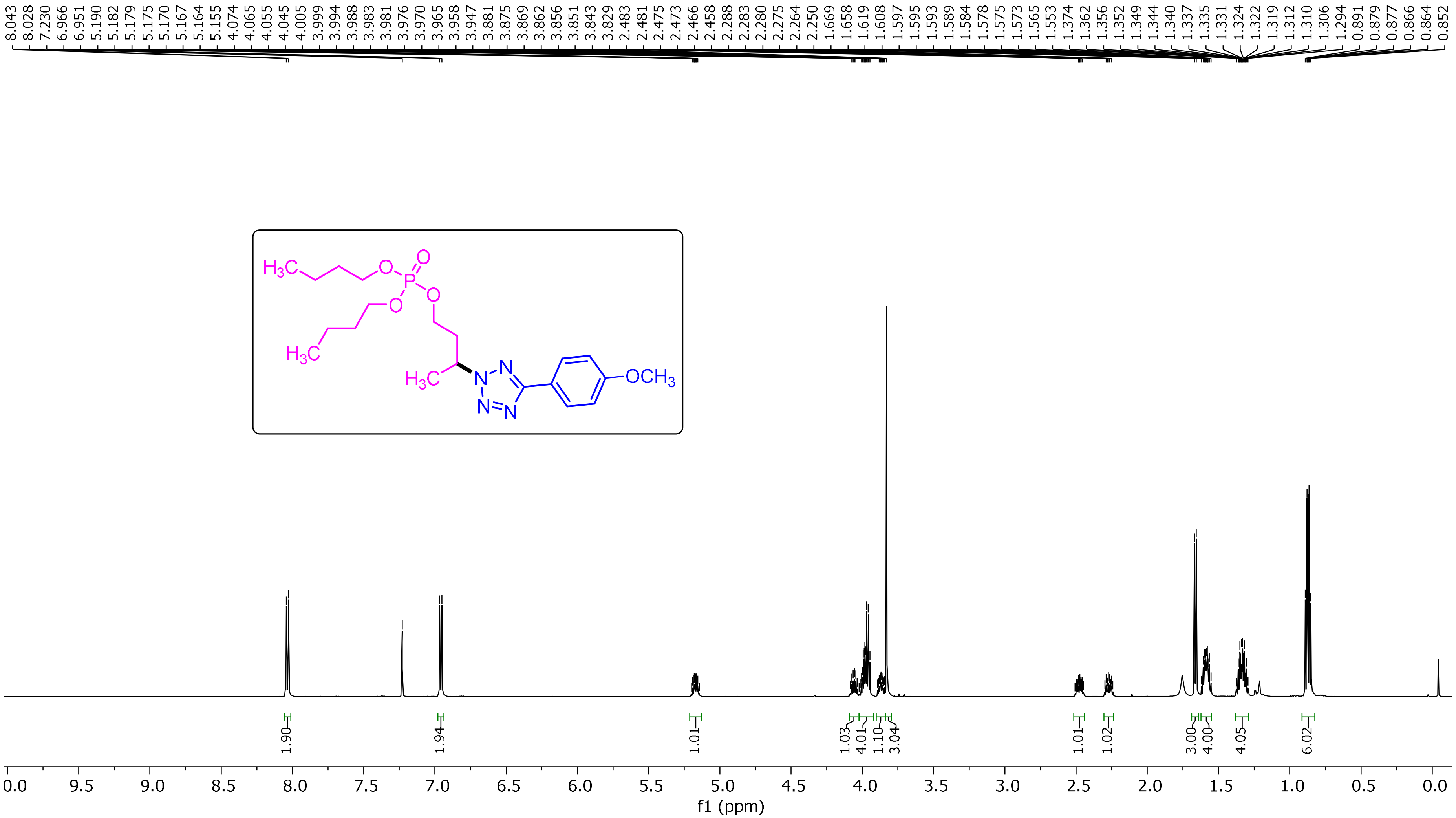

**Dibutyl (3-(5-(4-methoxyphenyl)-2*H*-tetrazol-2-yl)butyl) phosphate (27e): $^{13}$C NMR (151 MHz, CDCl$_3$)**

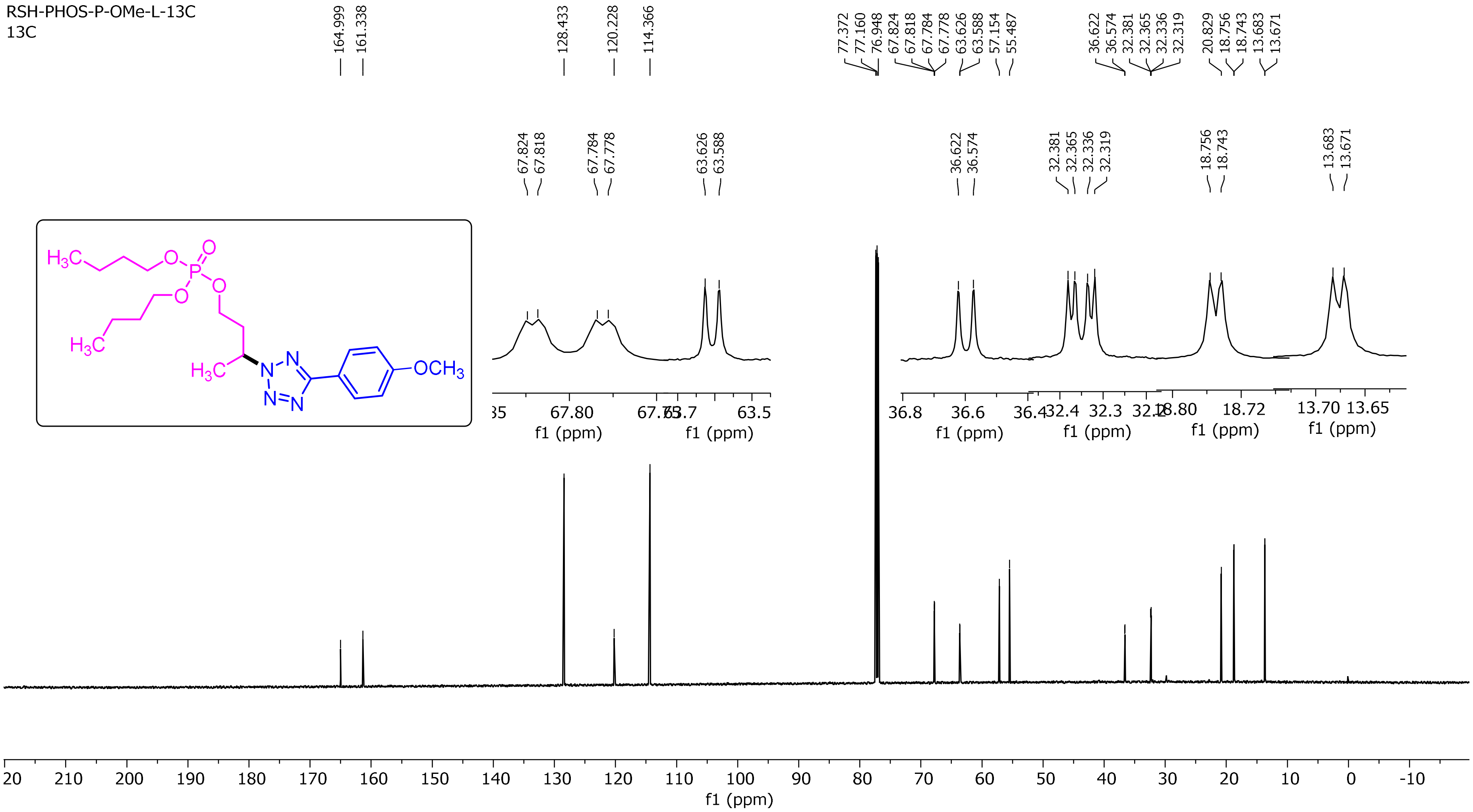

**Dibutyl (3-(5-(4-methoxyphenyl)-2*H*-tetrazol-2-yl)butyl) phosphate (27e): $^{31}$P {$^{1}$H} NMR (162 MHz, CDCl$_3$)**

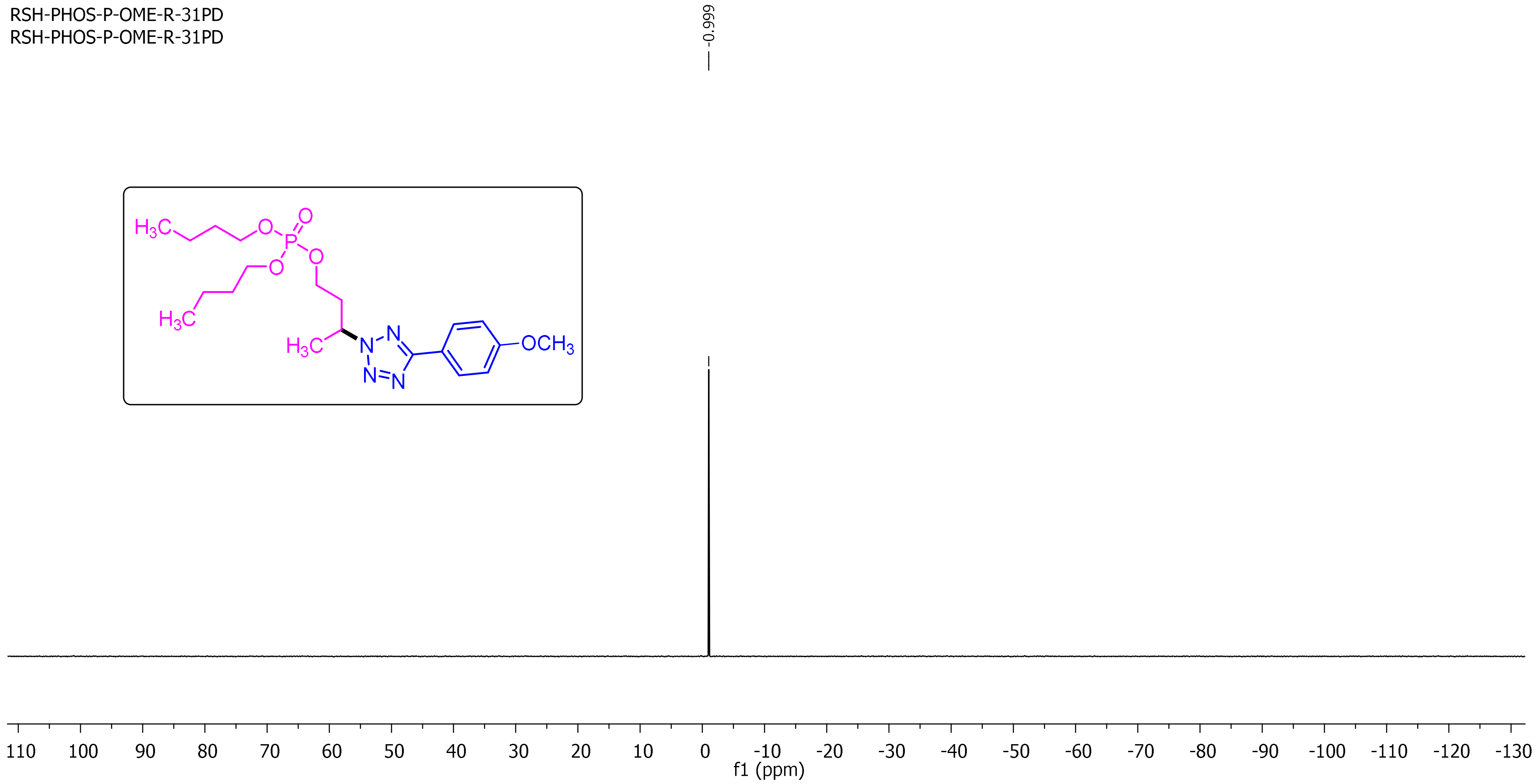

**Dibutyl (3-(5-(4-methoxyphenyl)-2*H*-tetrazol-2-yl)butyl) phosphate (27e): $^{31}$P NMR (162 MHz, $CDCl_3$)**

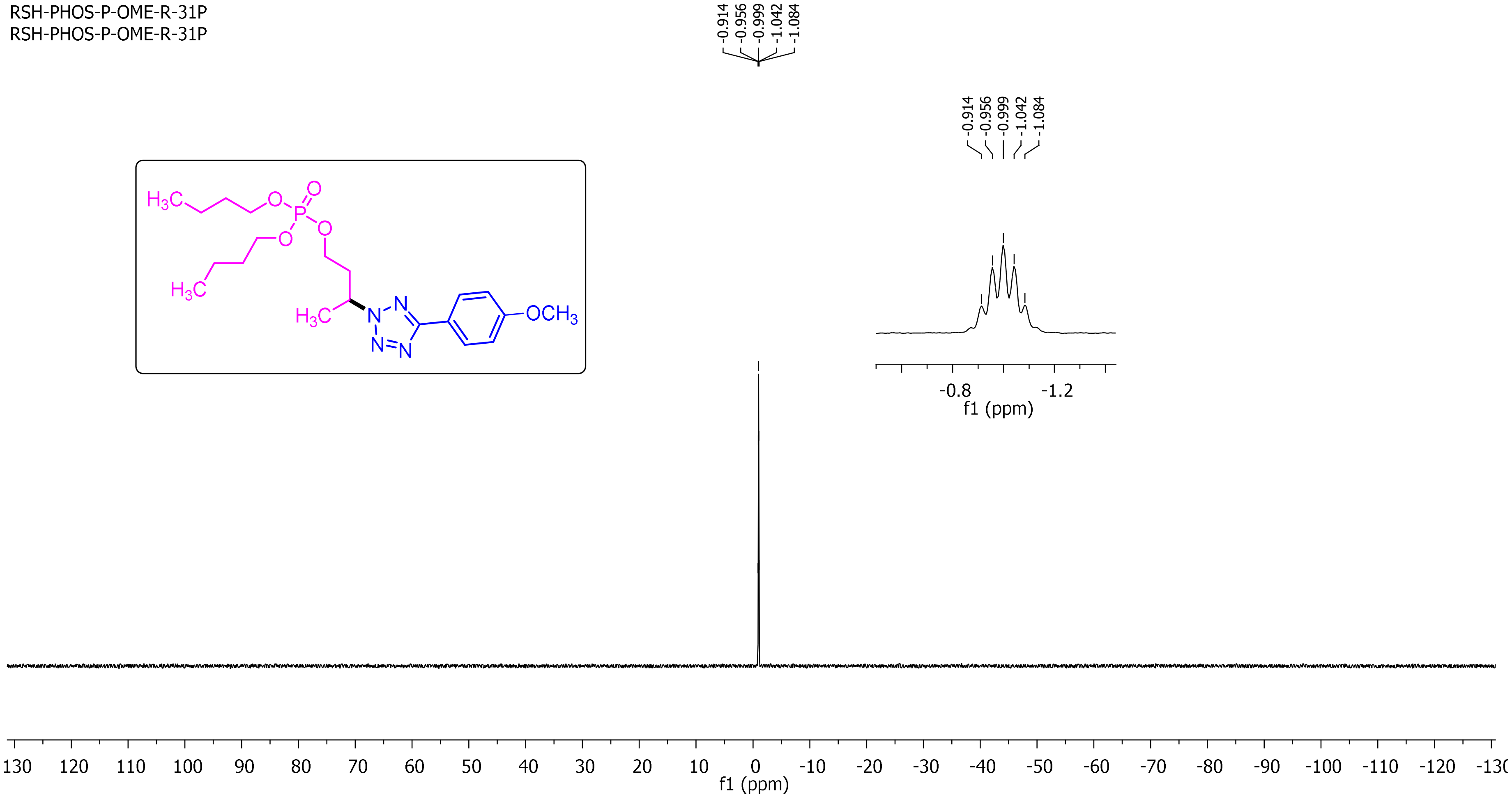

**Dibutyl (3-(5-(4-(trifluoromethyl)phenyl)-2*H*-tetrazol-2-yl)butyl) phosphate (27j): $^{1}$H NMR (600 MHz, $CDCl_3$)**

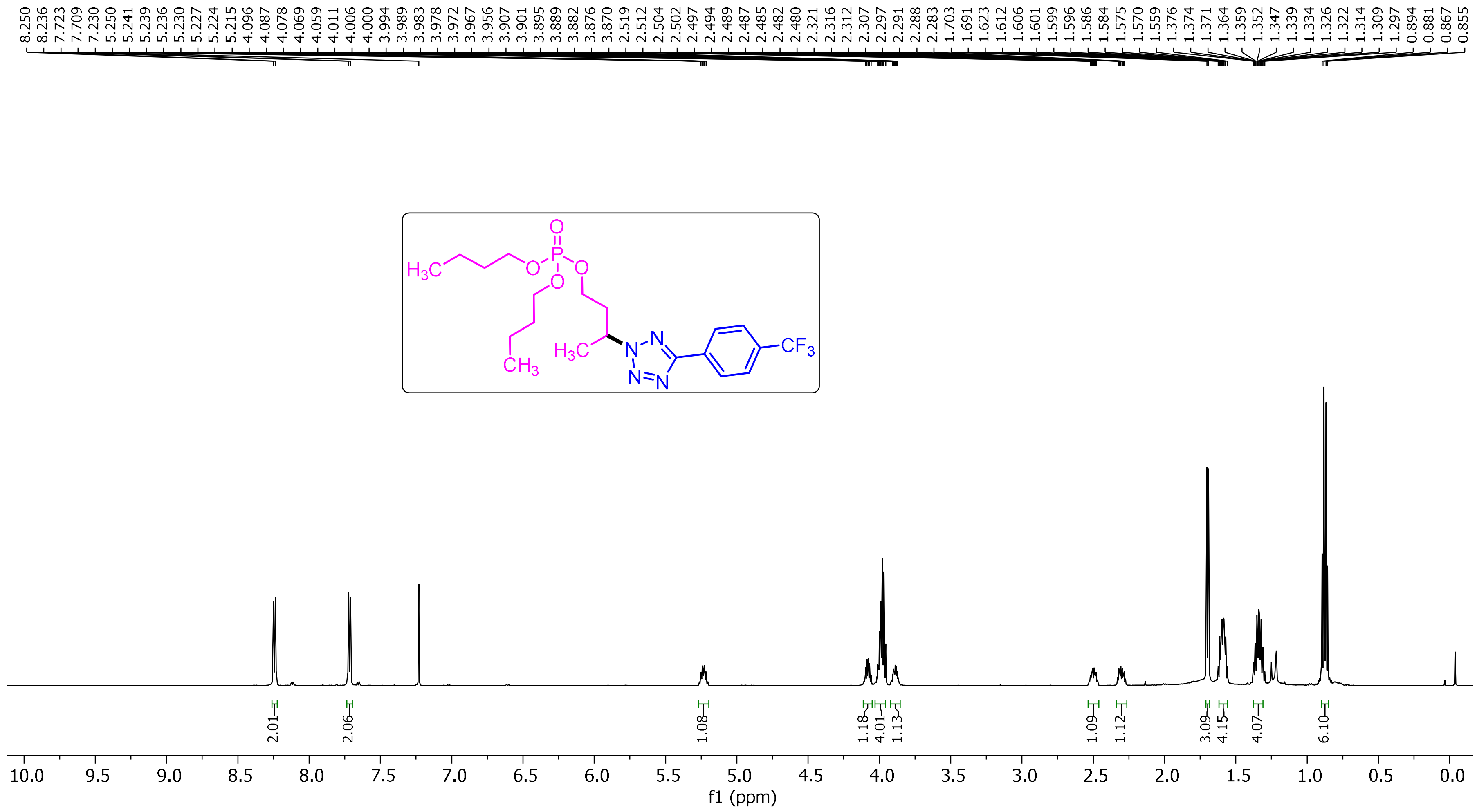

**Dibutyl (3-(5-(4-(trifluoromethyl)phenyl)-2*H*-tetrazol-2-yl)butyl) phosphate (27j): $^{13}$C NMR (151 MHz, $CDCl_3$)**

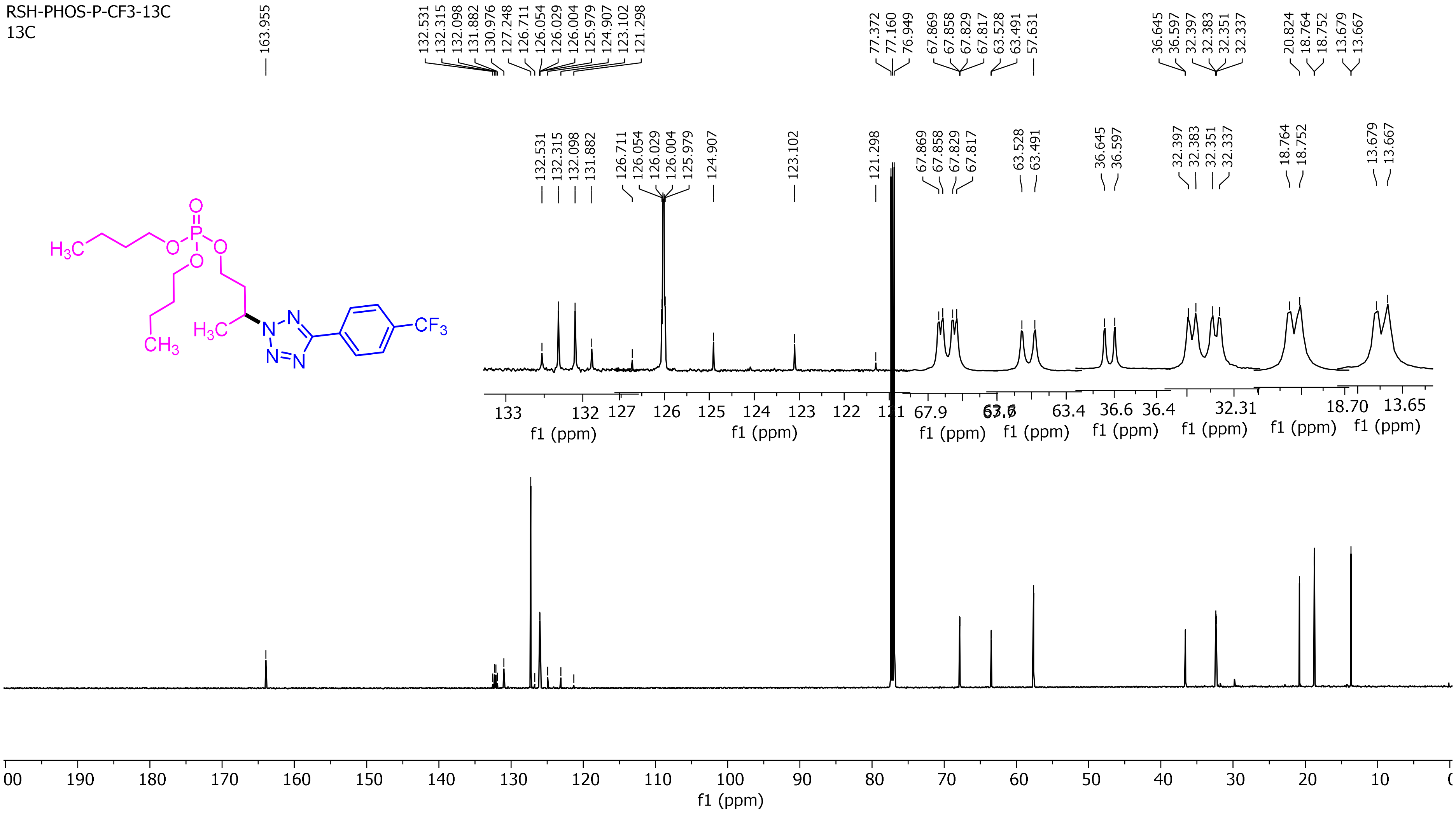

**Dibutyl (3-(5-(4-(trifluoromethyl)phenyl)-2*H*-tetrazol-2-yl)butyl) phosphate (27j): $^{31}$P {$^{1}$H} NMR (162 MHz, $CDCl_3$)**

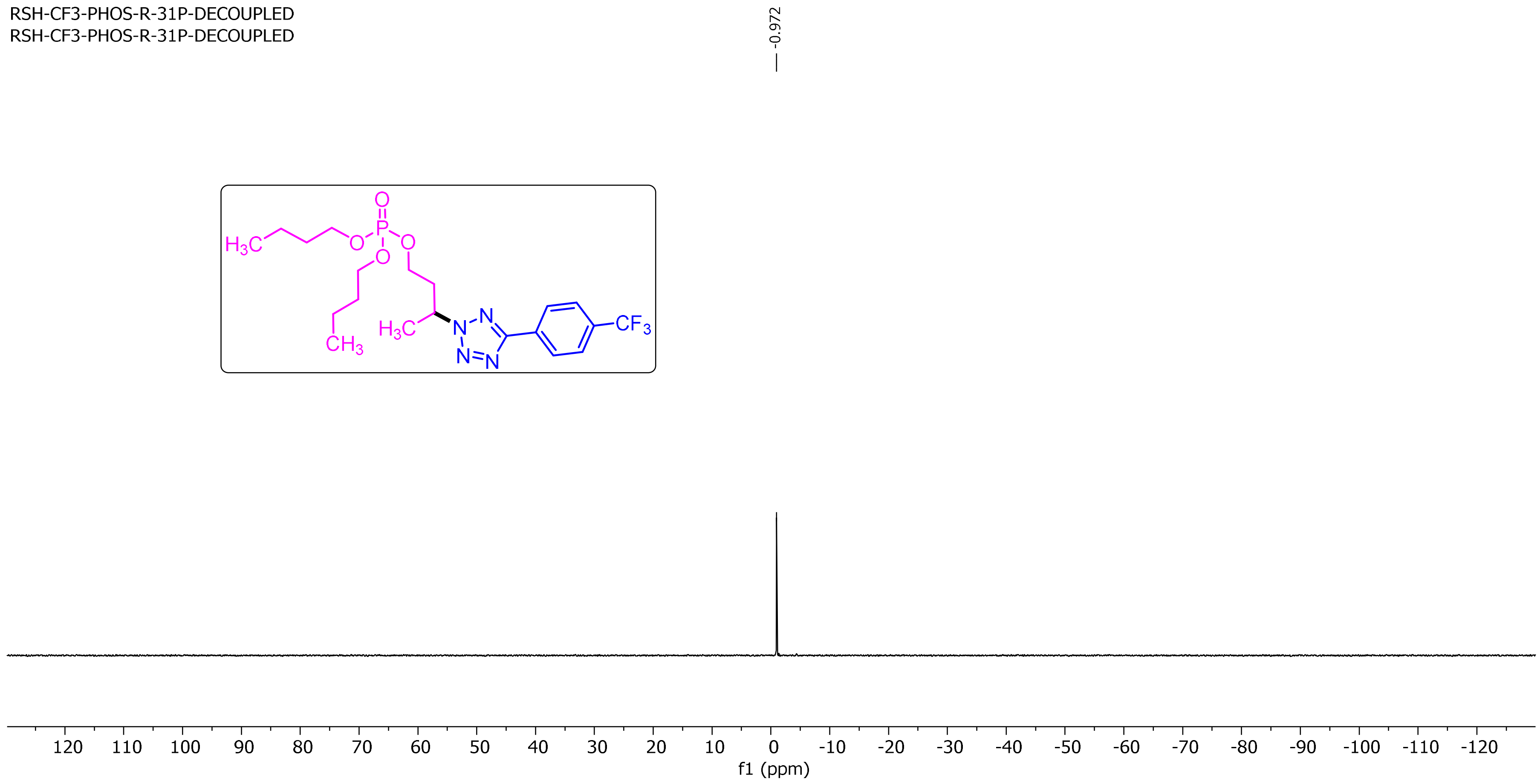

**Dibutyl (3-(5-(4-(trifluoromethyl)phenyl)-2*H*-tetrazol-2-yl)butyl) phosphate (27j): $^{19}F$ {$^{1}H$} NMR (377 MHz, $CDCl_3$)**

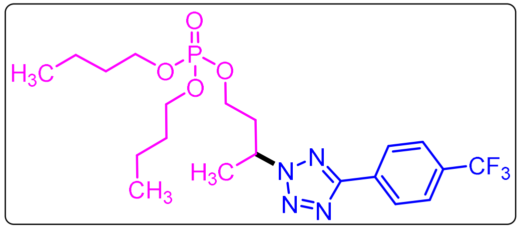


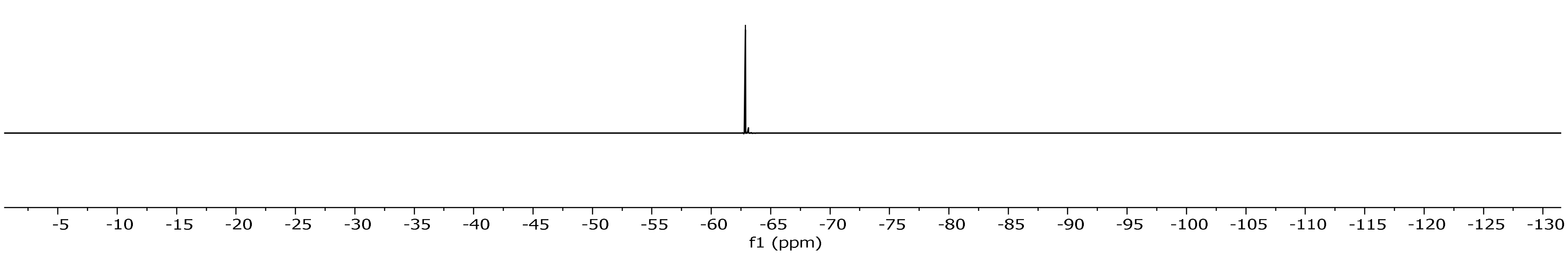

**N-Butylbenzamide (28'a): $^{1}$H NMR (400 MHz, $CDCl_3$)**

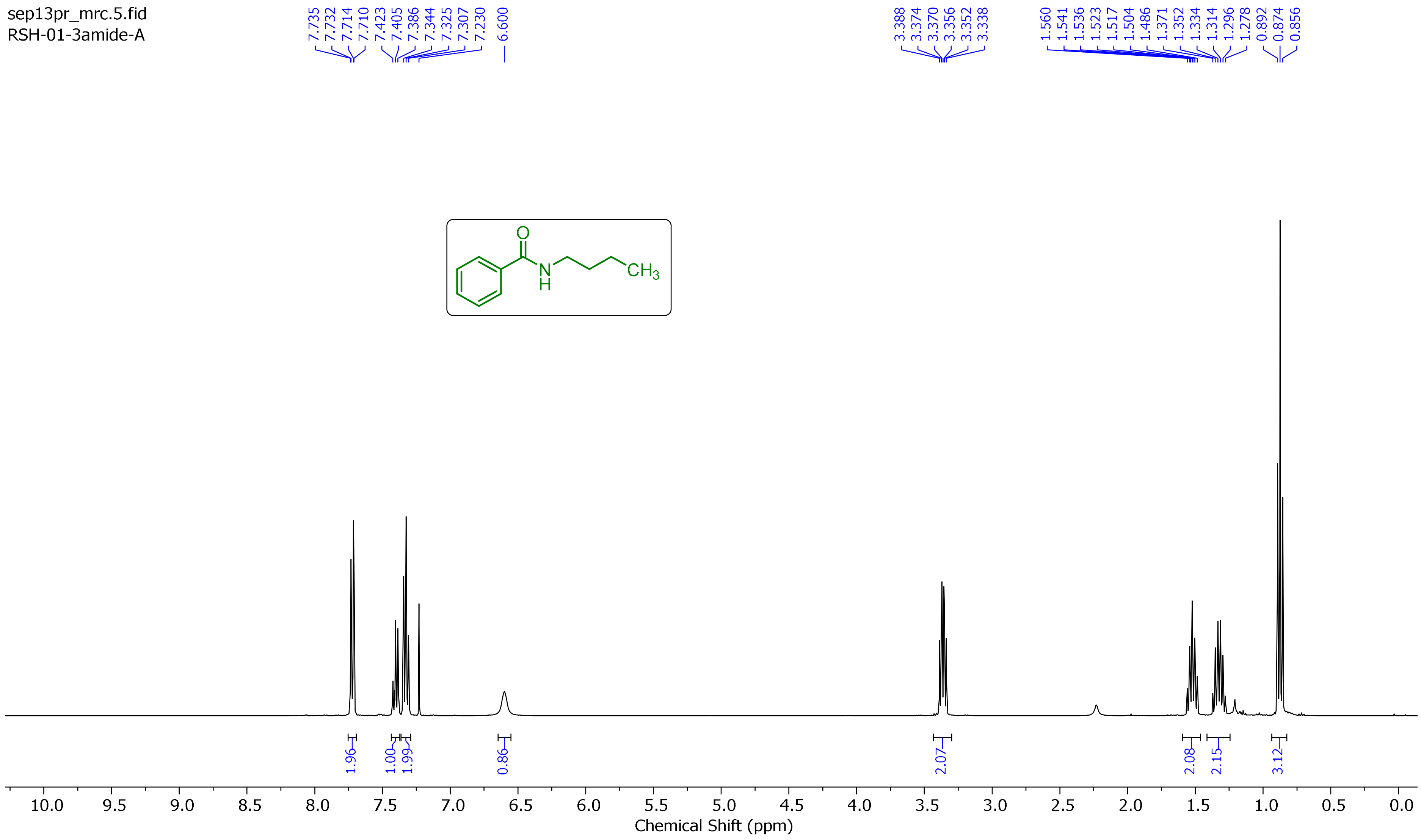

**$N$-Butylbenzamide (28'a): $^{13}$C NMR** (101 **MHz, $CDCl_3$**)

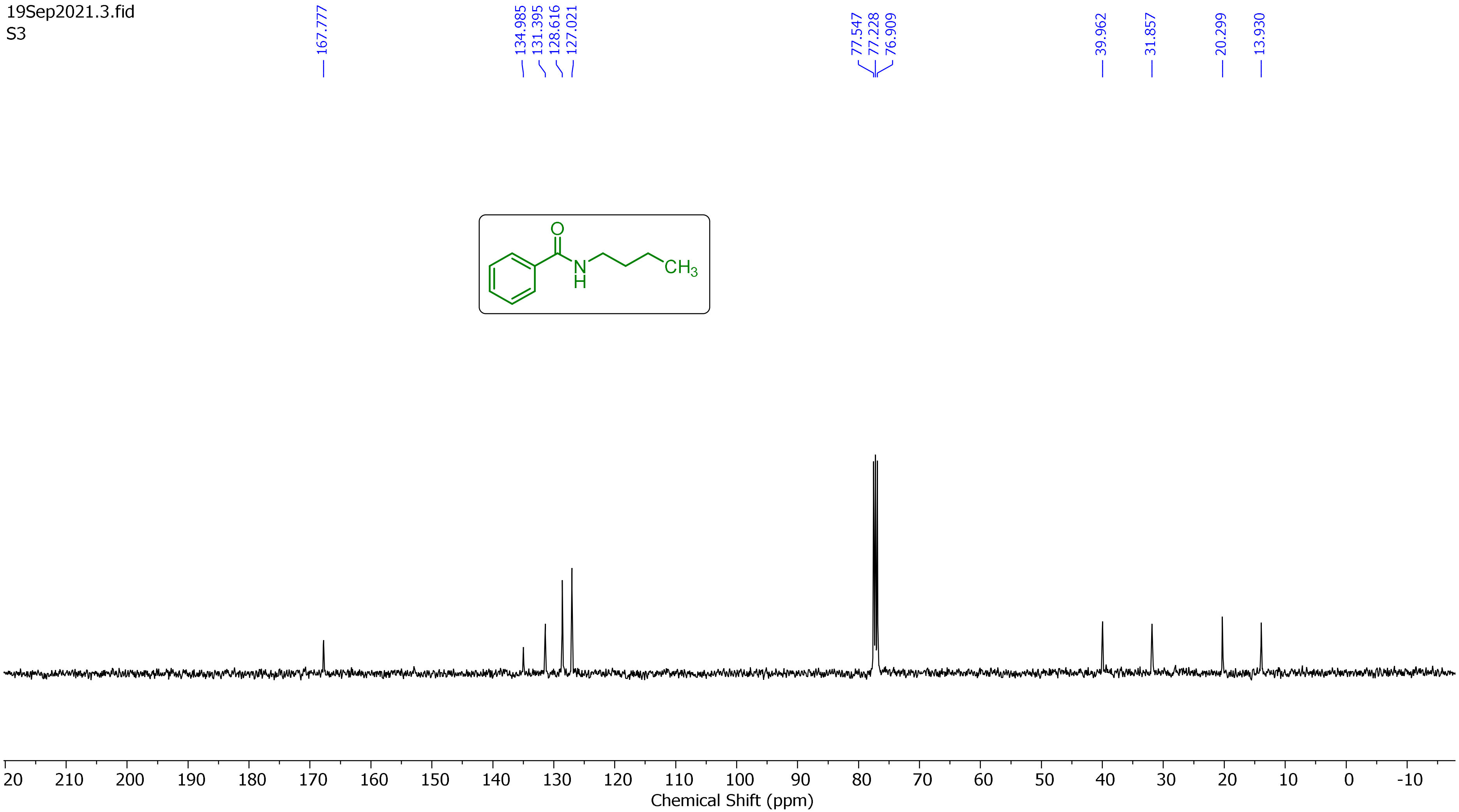

**N-Butylbenzamide N-Methoxy-N-((5-phenyl-2H-tetrazol-2-yl)methyl)pentanamide (29'a): $^1$H NMR (400 MHz, CDCl$_3$)**

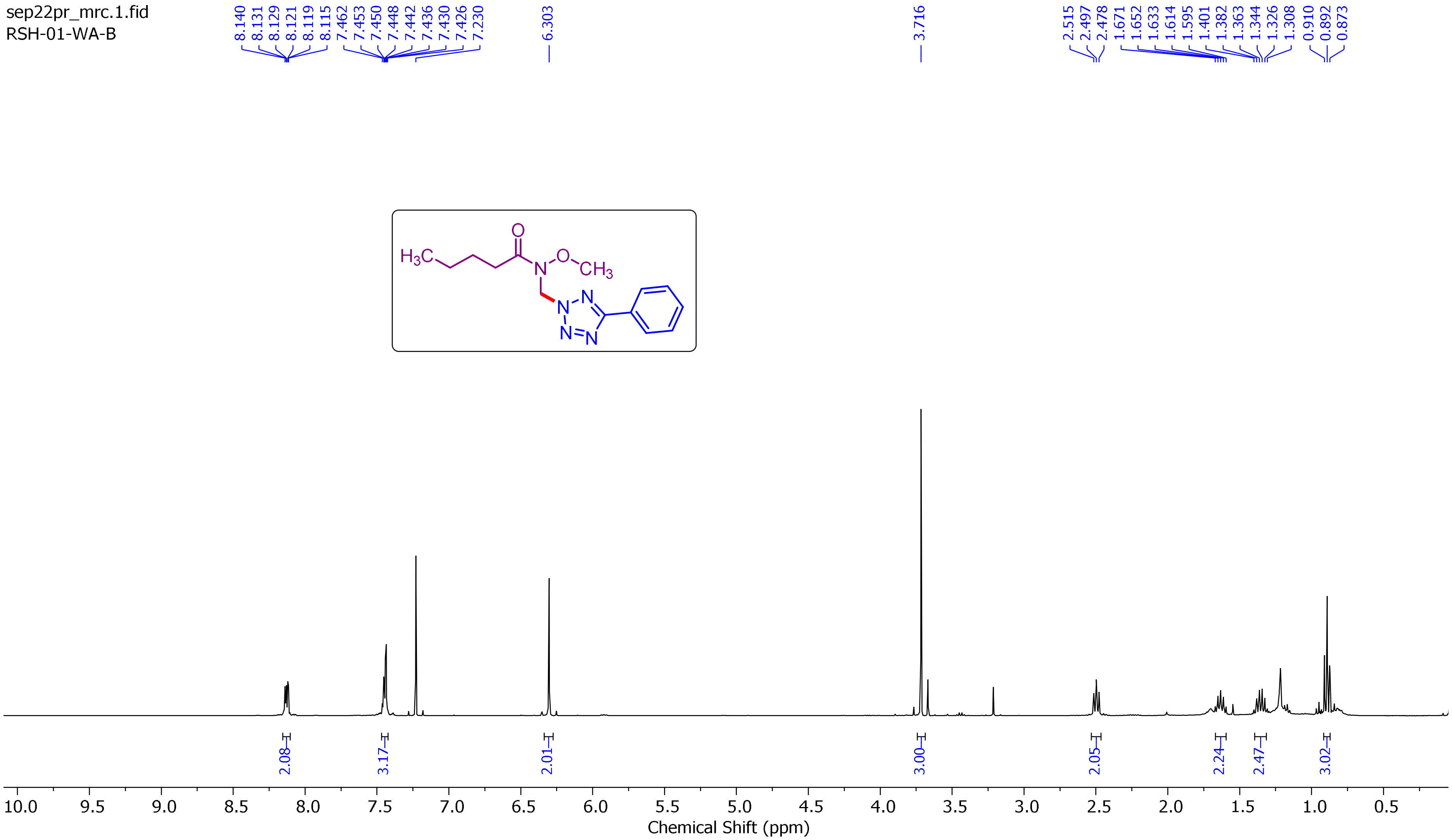

***N*-Butylbenzamide *N*-Methoxy-*N*-((5-phenyl-2*H*-tetrazol-2-yl)methyl)pentanamide (29'a): $^{1}$H NMR (101 MHz, $CDCl_3$)**

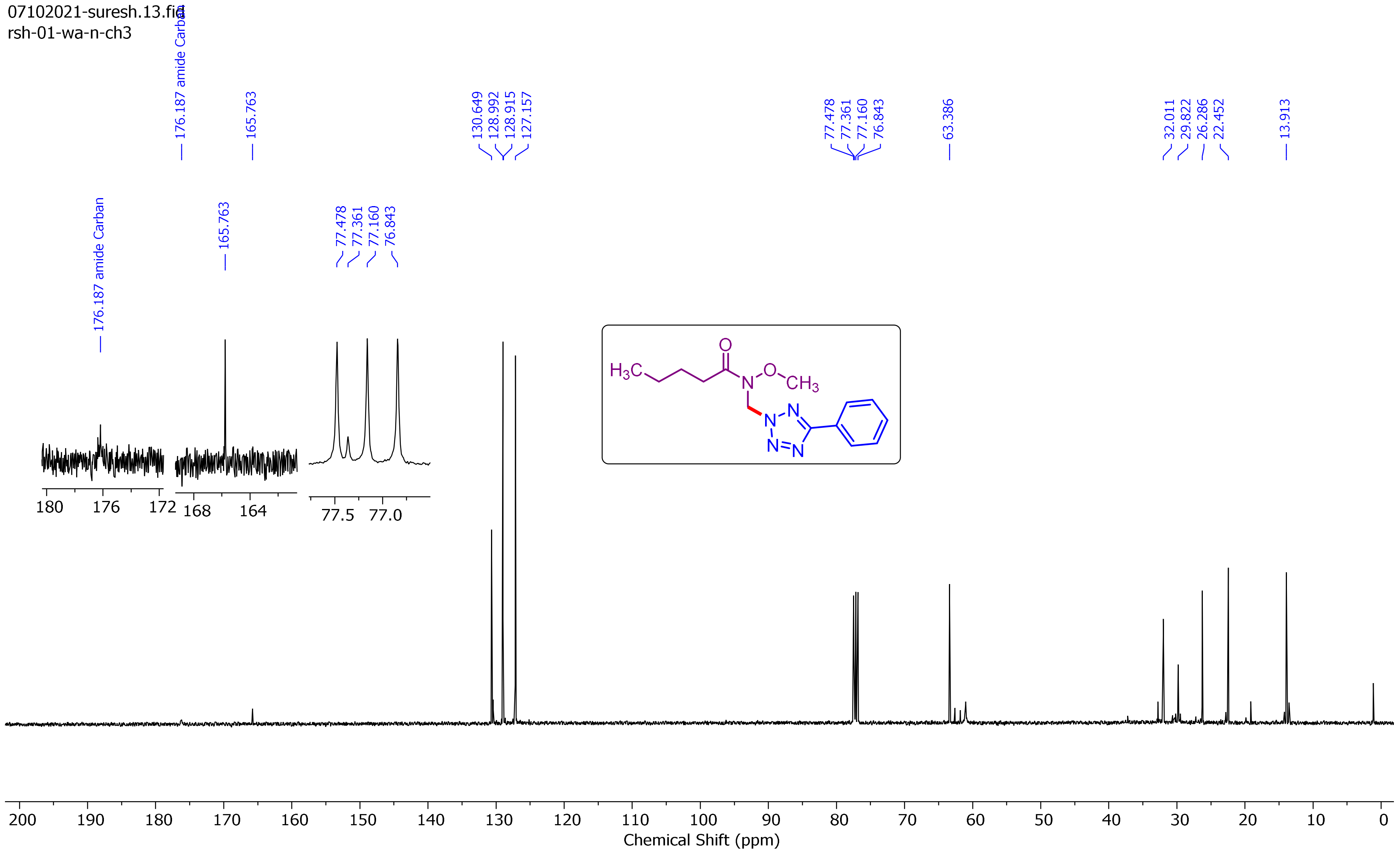

***N*-Methyl-*N*-((5-phenyl-2*H*-tetrazol-2-yl)methoxy)pentanamide (29''a) + Uncharacterised inseparable impurity: $^{1}$H NMR (400 MHz, CDCl$_3$)**

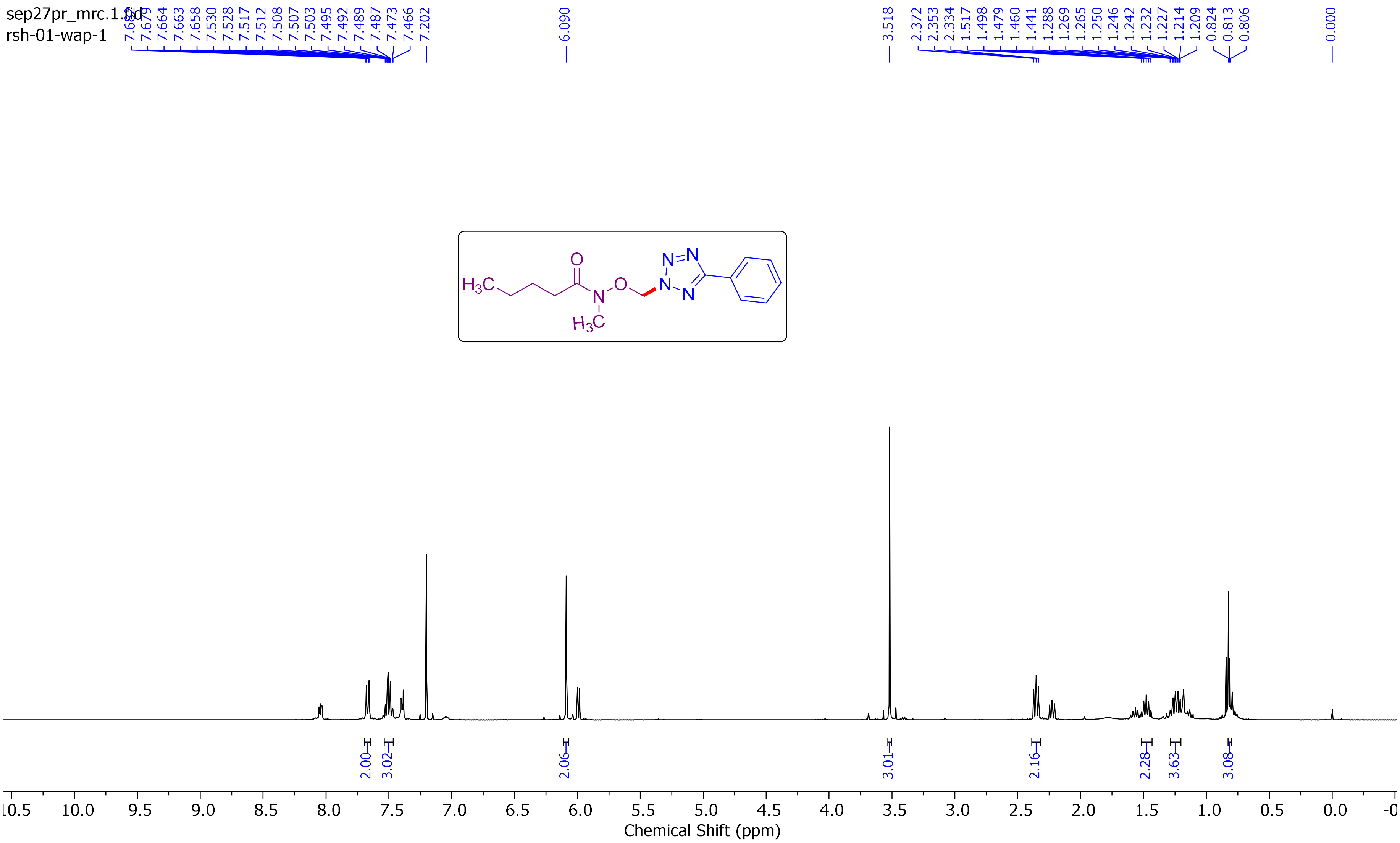

**$N$-Methyl-$N$-((5-phenyl-2$H$-tetrazol-2-yl)methoxy)pentanamide (29''a) + Uncharacterised inseparable impurity: $^{13}$C NMR** (101 **MHz,** **$CDCl_3$**)

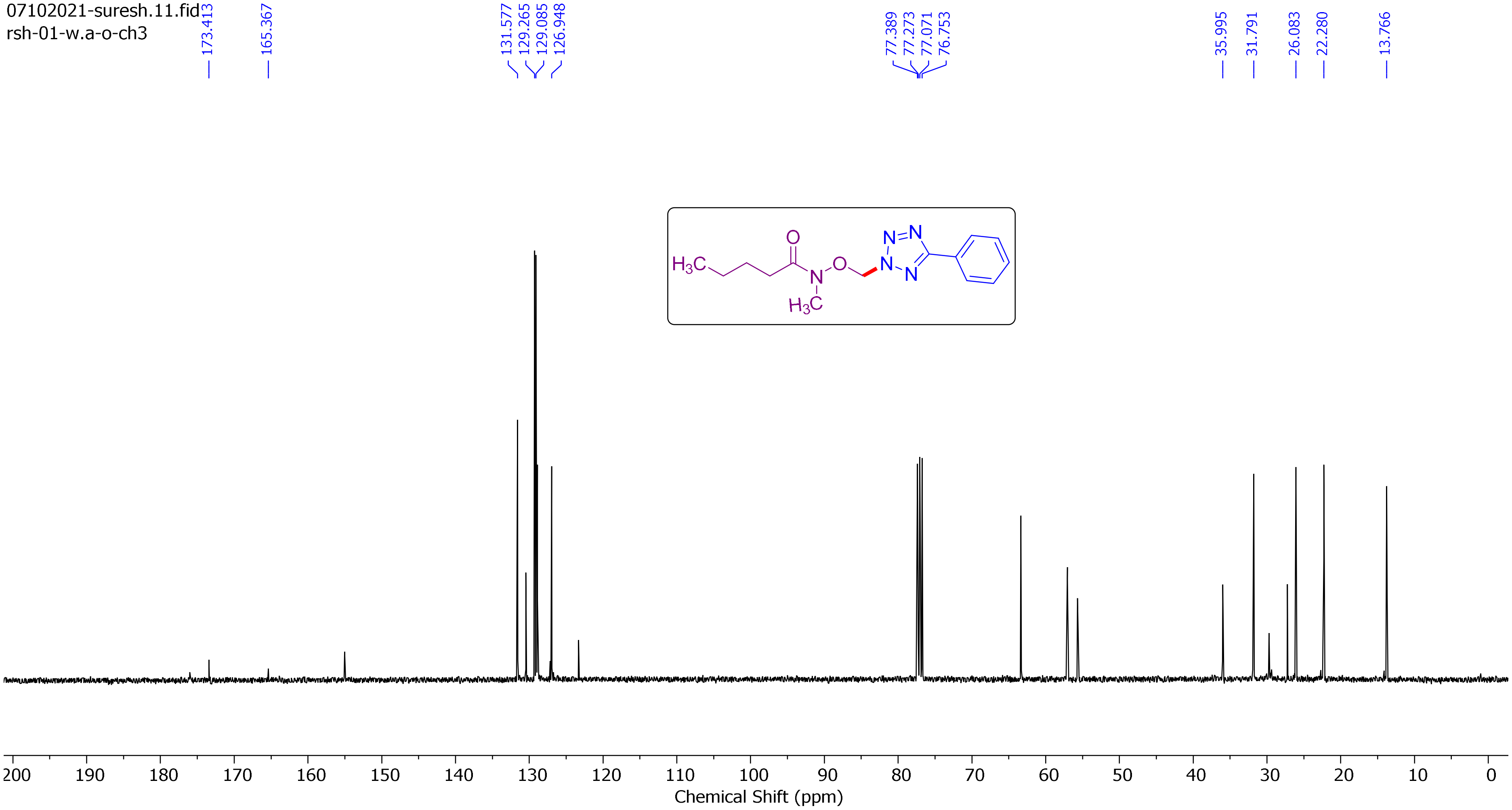

**1-Phenyl-2-(5-phenyl-2*H*-tetrazol-2-yl)pentan-1-one (30a): $^{1}$H NMR (400 MHz, CDCl₃)**

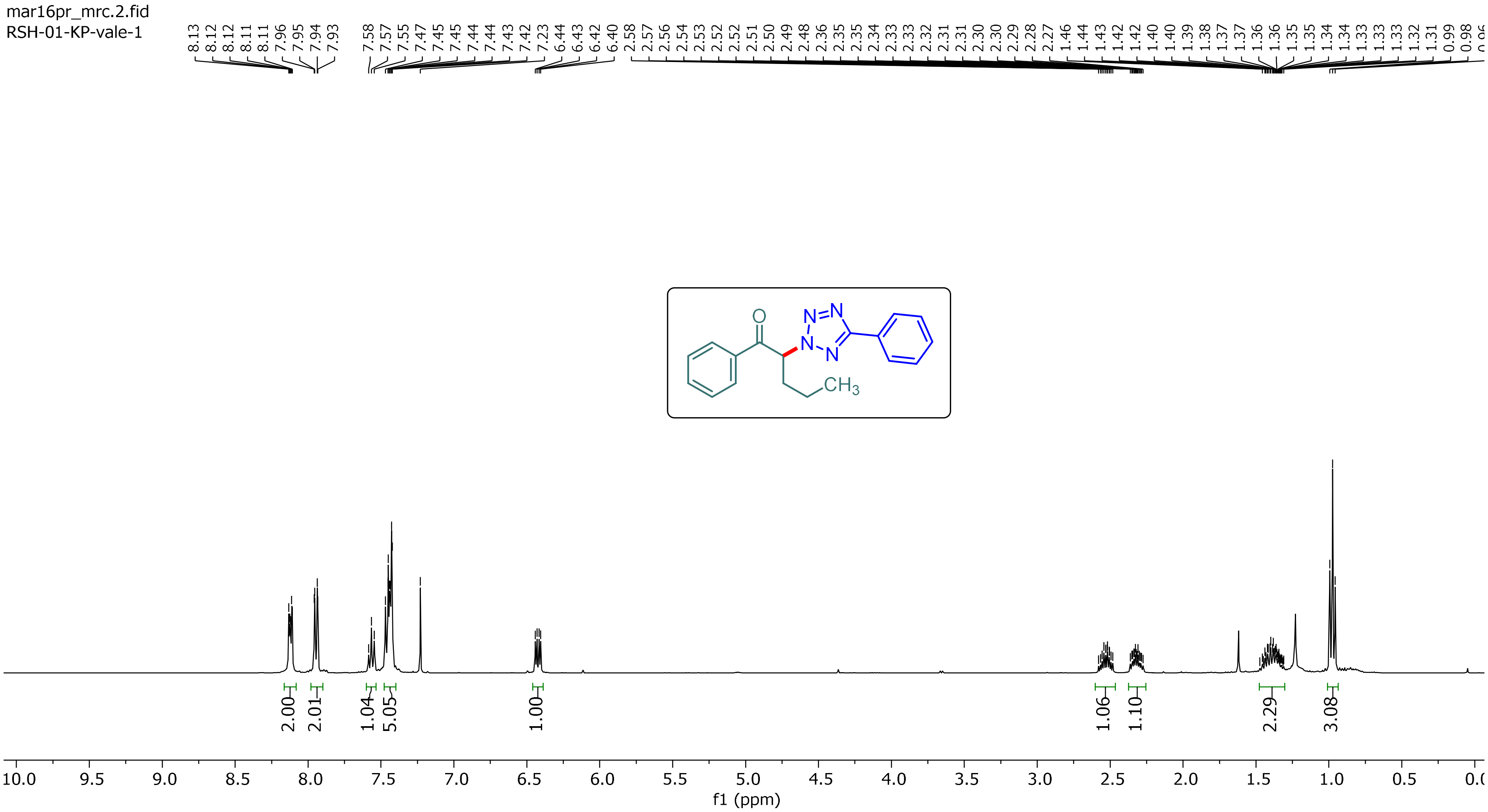

**1-Phenyl-2-(5-phenyl-2*H*-tetrazol-2-yl)pentan-1-one (30a): $^{13}C$ NMR (101 MHz, $CDCl_3$)**

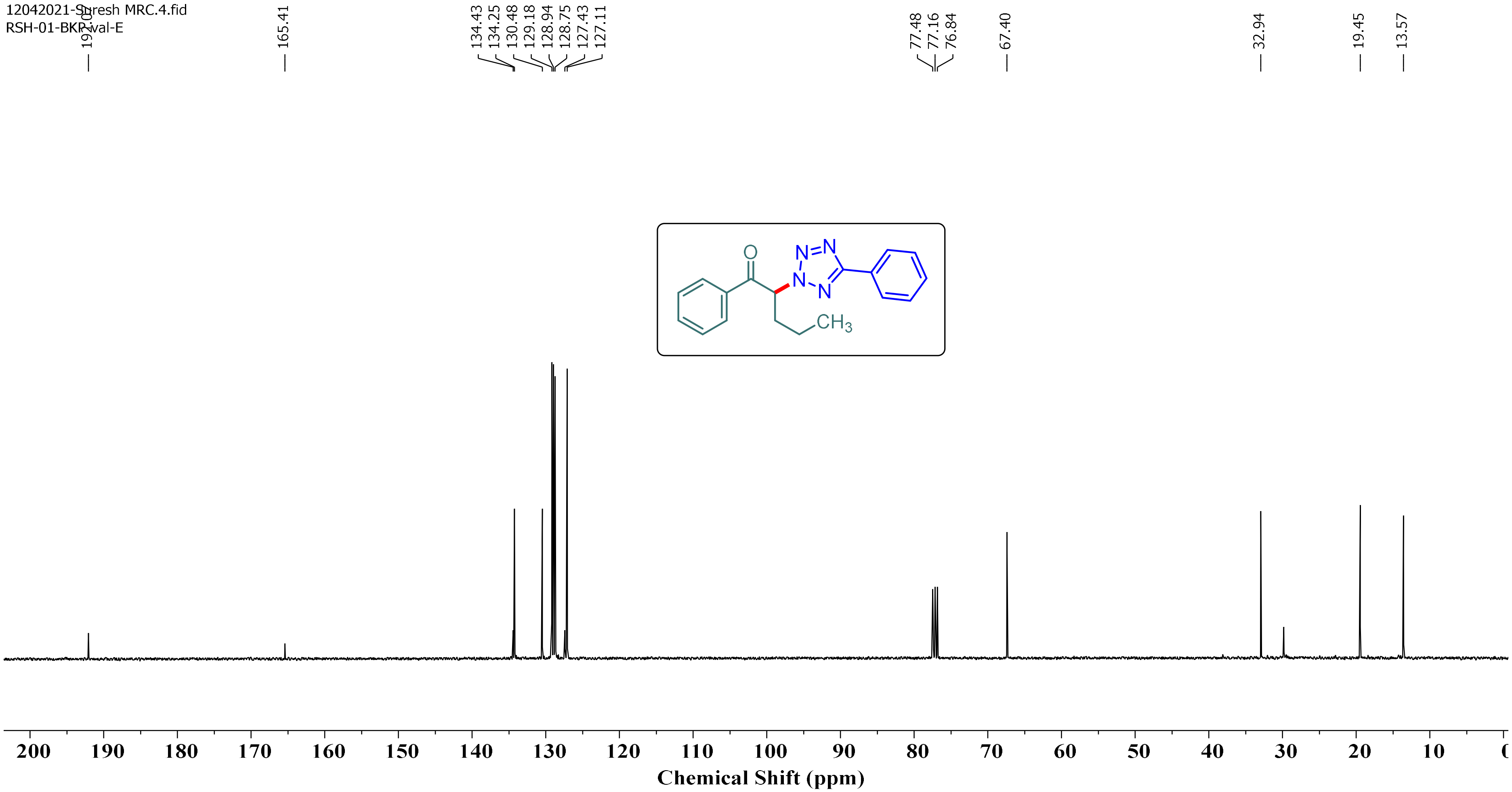

**1-(5-Phenyl-2*H*-tetrazol-2-yl)propan-2-one (31a): $^{1}$H NMR (600 MHz, $CDCl_3$)**

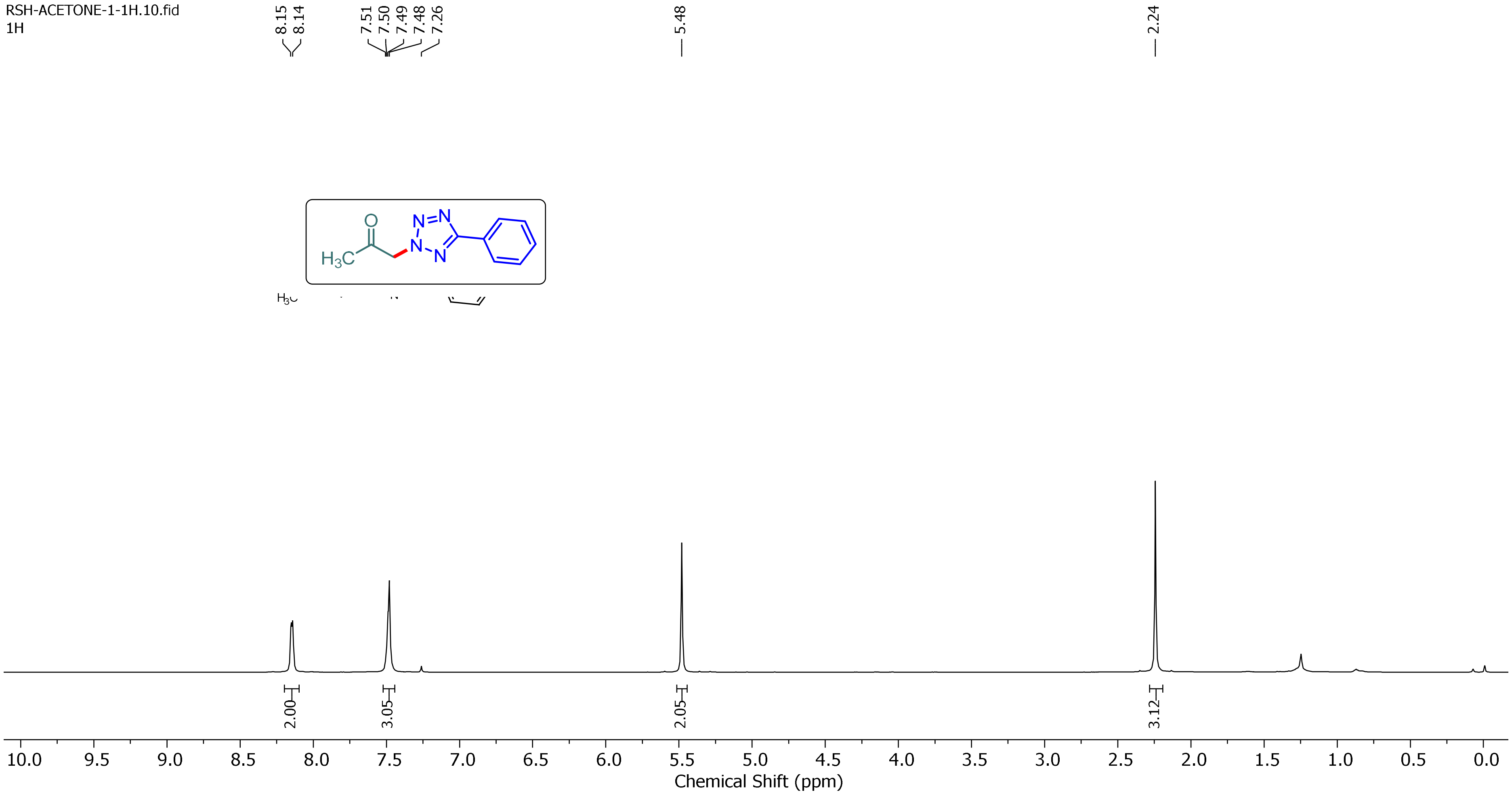

**1-(5-Phenyl-2*H*-tetrazol-2-yl)propan-2-one (31a): $^{13}$C NMR (151 MHz, $CDCl_3$)**

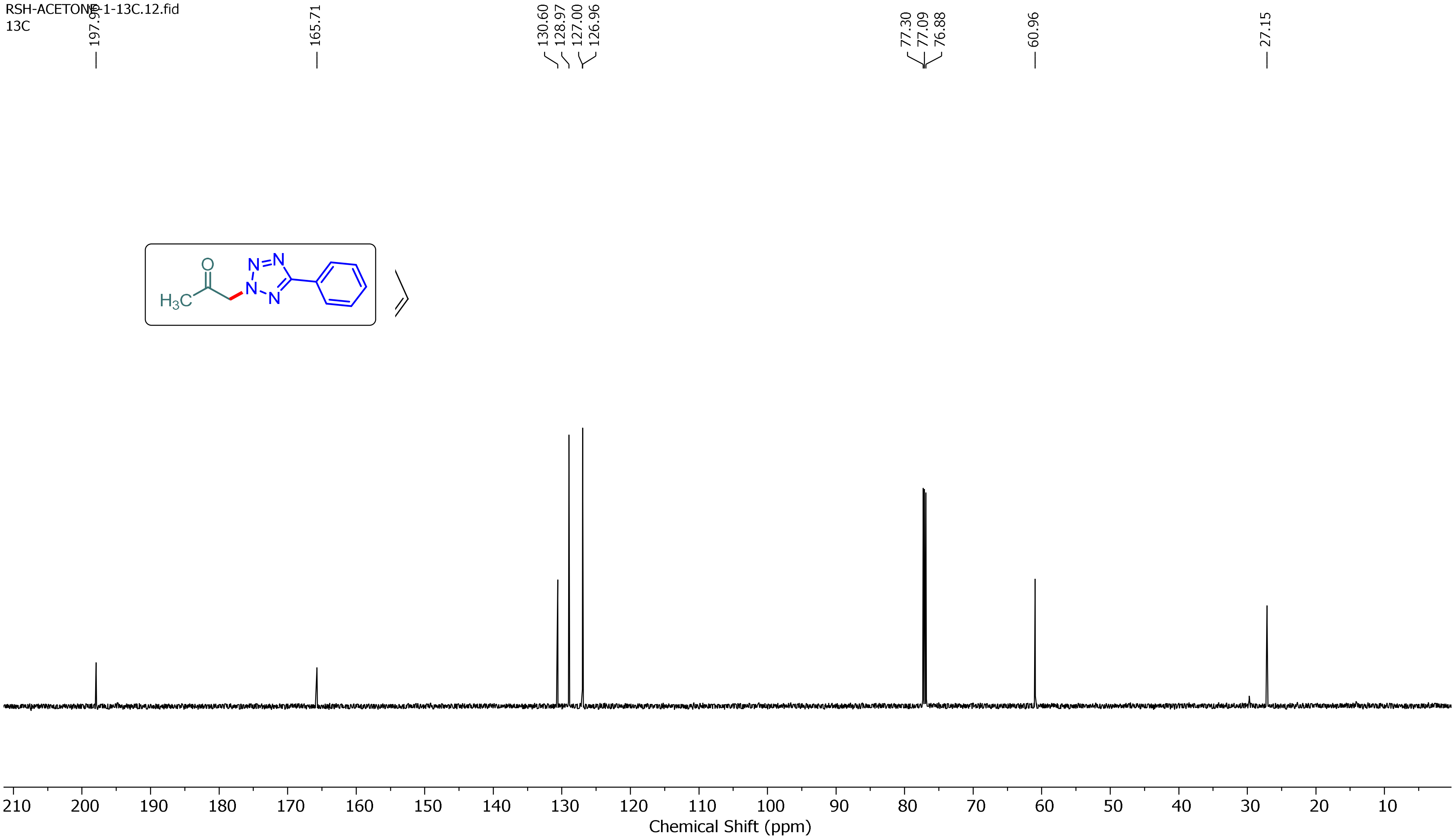

**2-(5-Phenyl-2*H*-tetrazol-2-yl)octan-3-one (32a): $^{1}$H NMR (600 MHz, $CDCl_3$)**

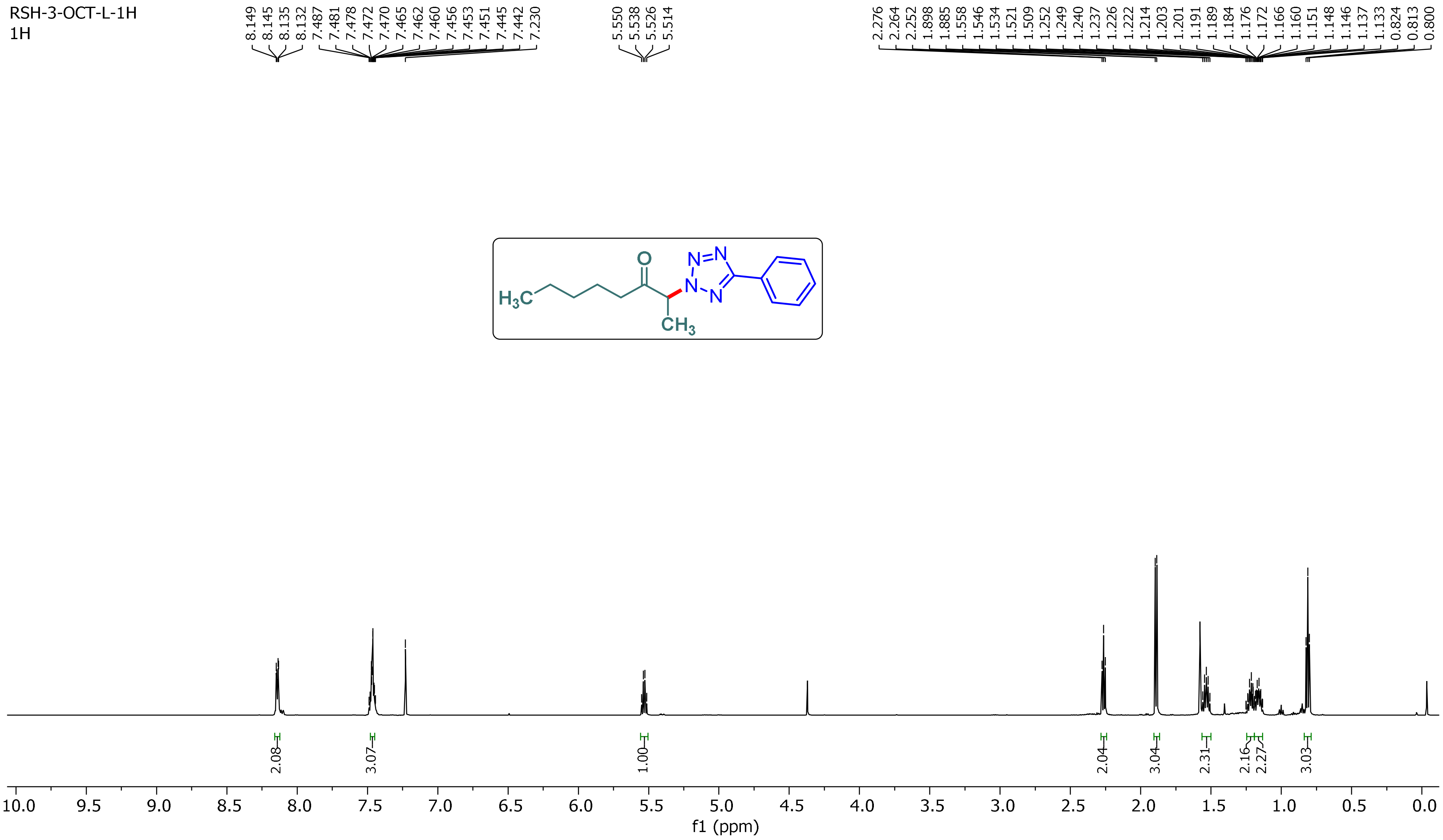

**2-(5-Phenyl-2*H*-tetrazol-2-yl)octan-3-one (32a):** $^{13}$**C NMR (151 MHz, CDCl$_3$)**

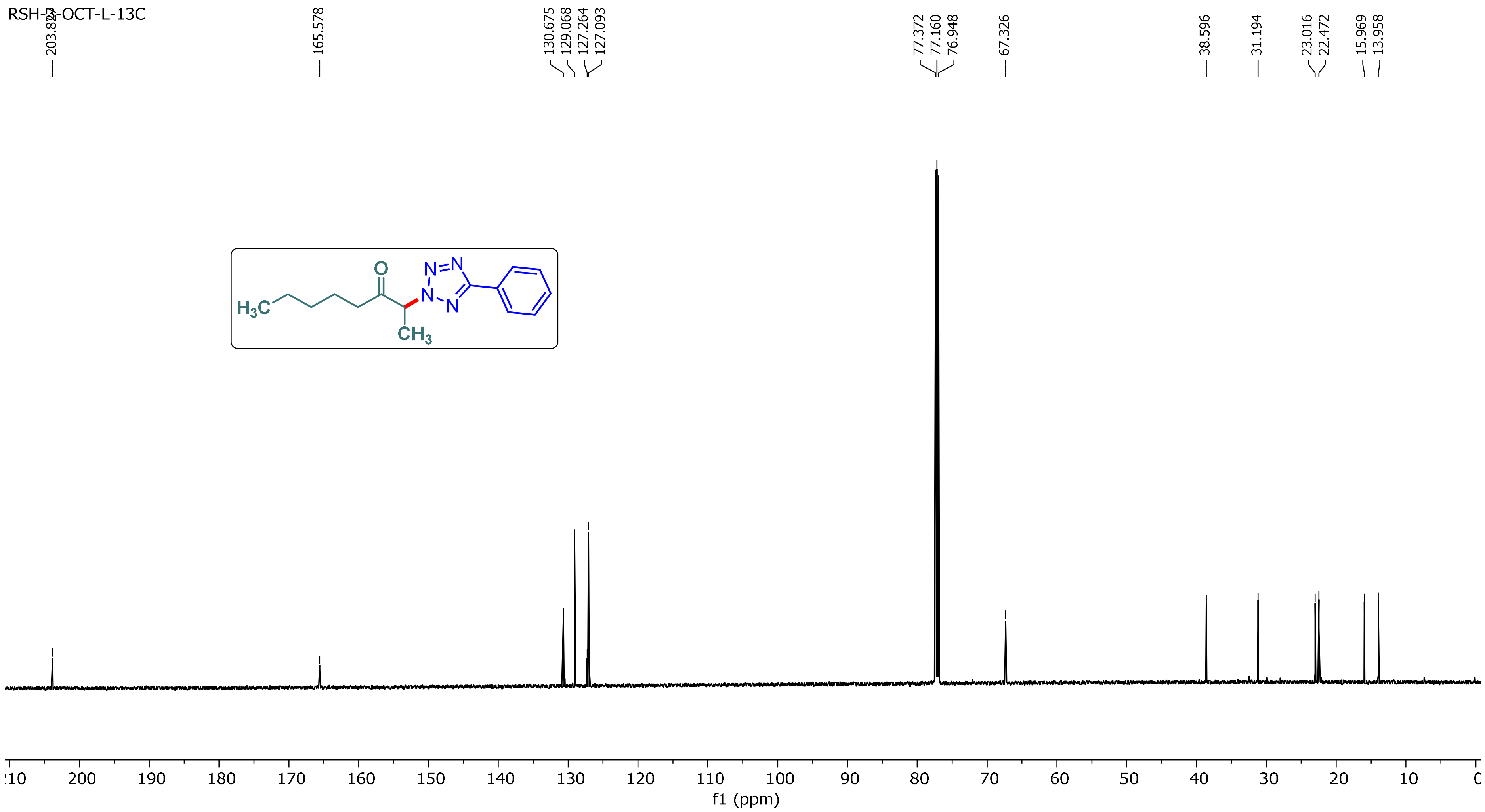

**4-(5-Phenyl-2*H*-tetrazol-2-yl)octan-3-one (32'a): $^{1}$H NMR (600 MHz, $CDCl_3$)**

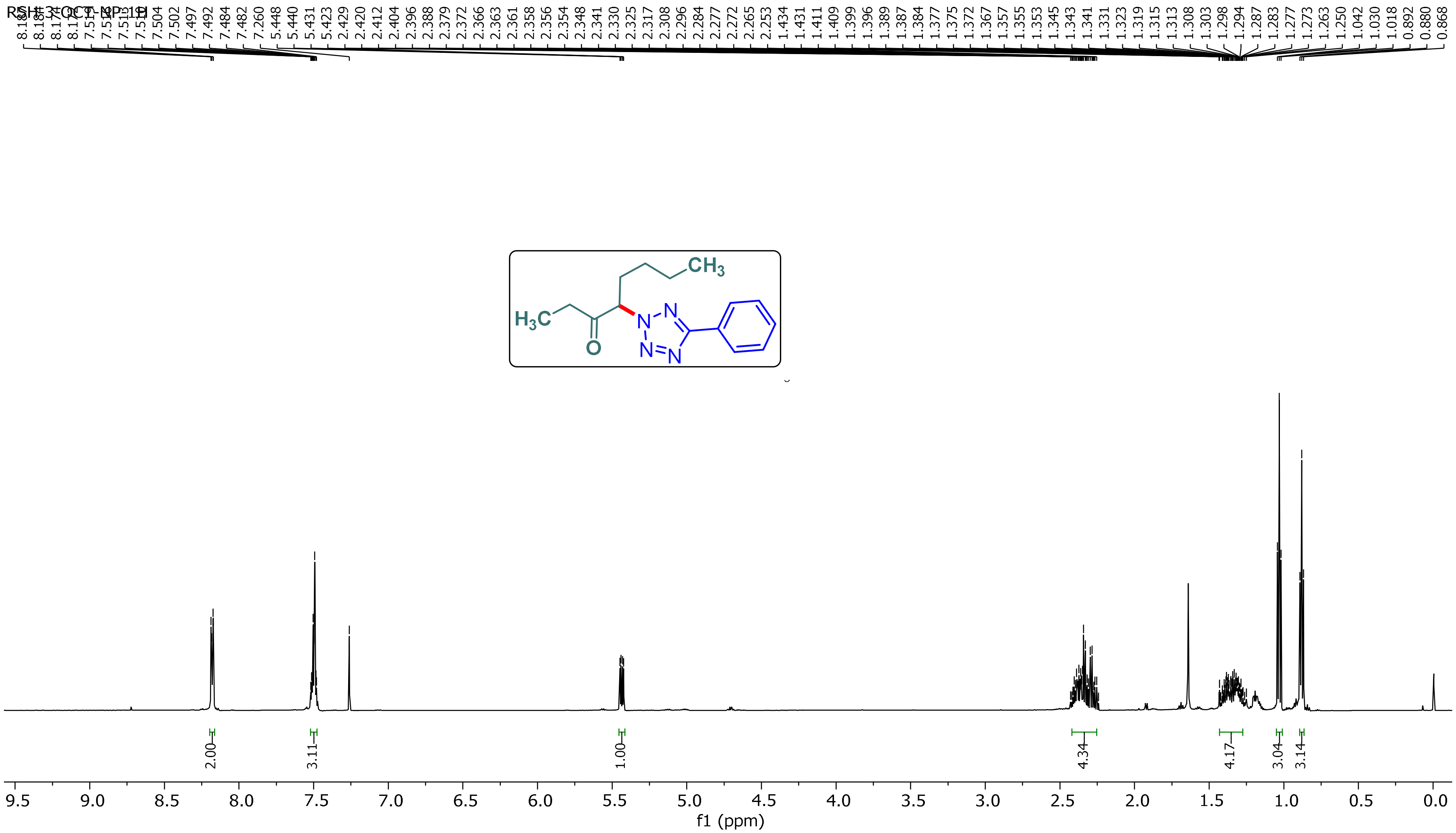

**4-(5-Phenyl-2*H*-tetrazol-2-yl)octan-3-one (32'a): $^{13}$C NMR (151 MHz, $CDCl_3$)**

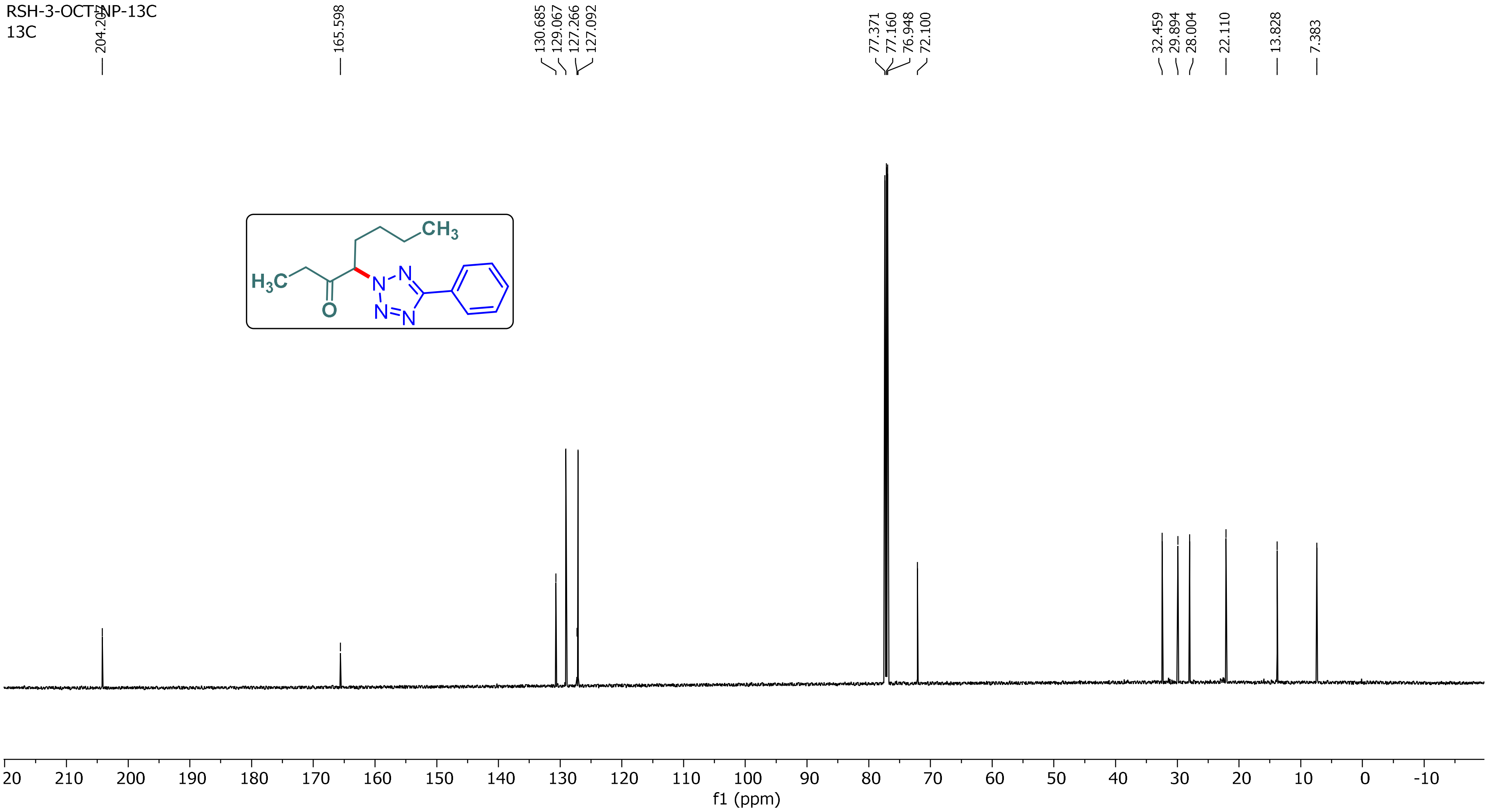

**6-Methoxy-4-(5-phenyl-2*H*-tetrazol-2-yl)-3,4-dihydronaphthalen-1(2*H*)-one (33a): $^{1}$H NMR (600 MHz, $CDCl_3$)**

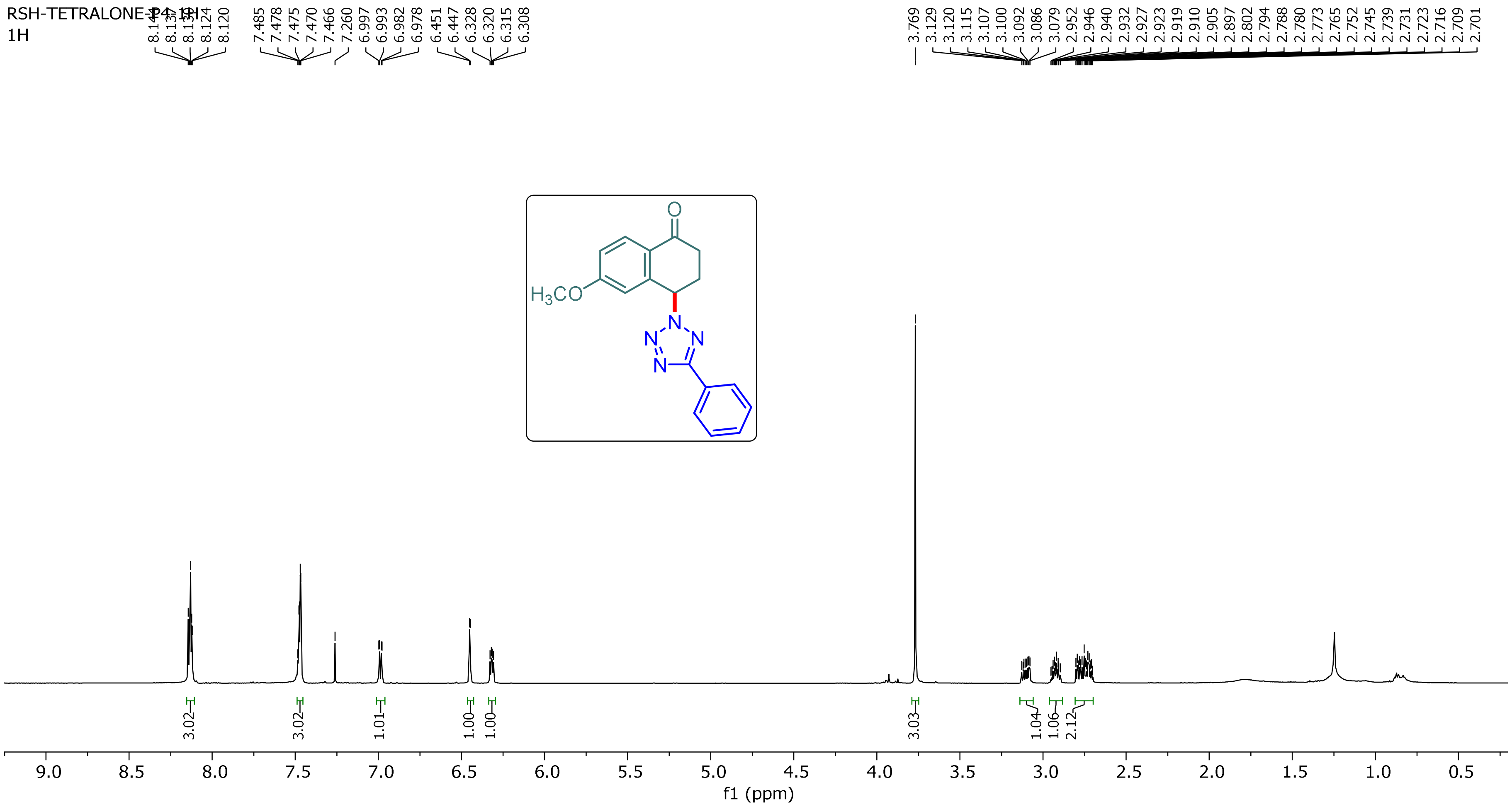

**6-Methoxy-4-(5-phenyl-2*H*-tetrazol-2-yl)-3,4-dihydronaphthalen-1(2*H*)-one (33a): $^{13}$C NMR (151 MHz, CDCl$_3$)**

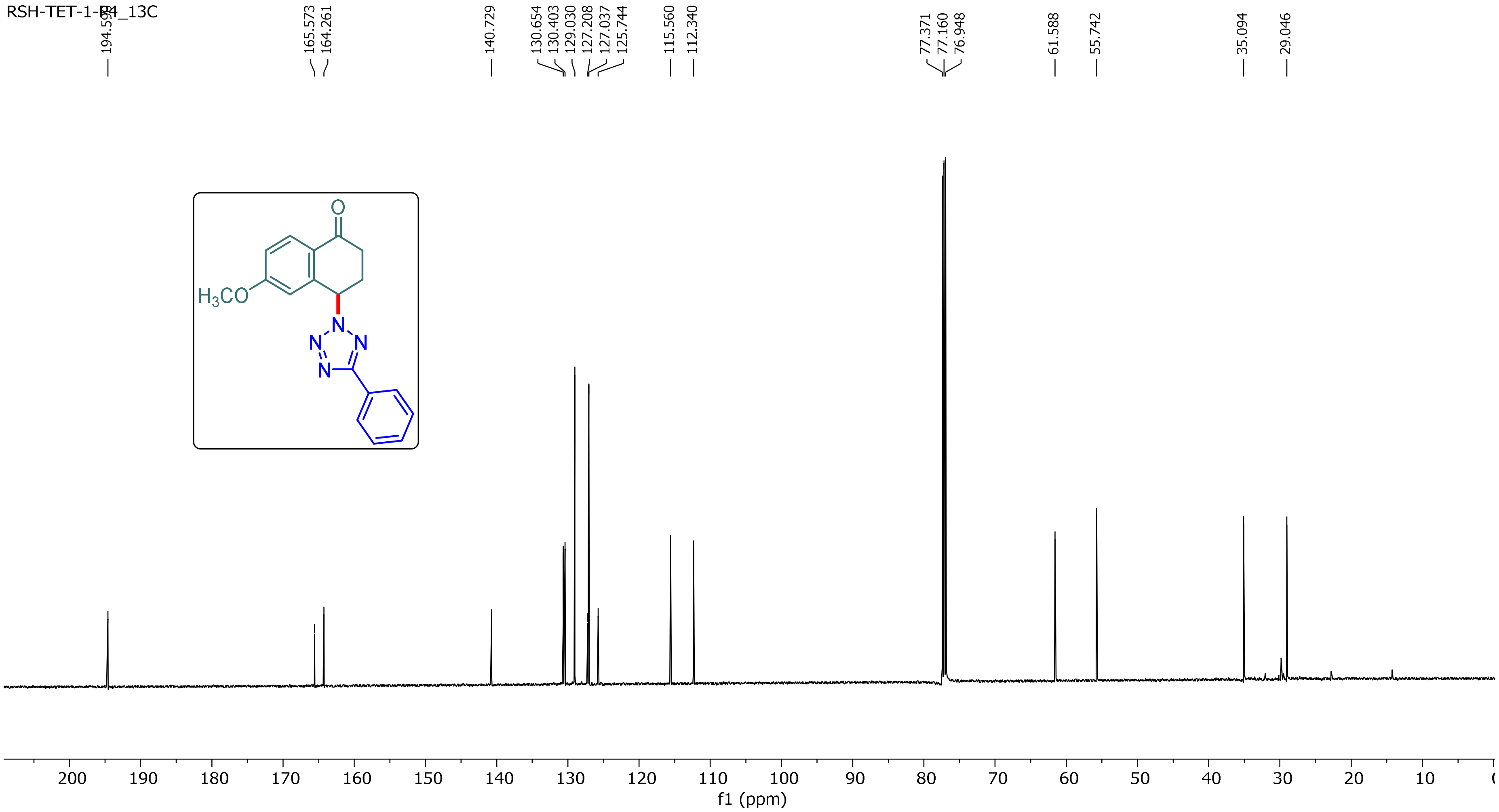

**4-(1-(5-Phenyl-2*H*-tetrazol-2-yl)pentyl)pyridine (34a)**: $^{1}$**H NMR (600 MHz, CDCl$_3$)**

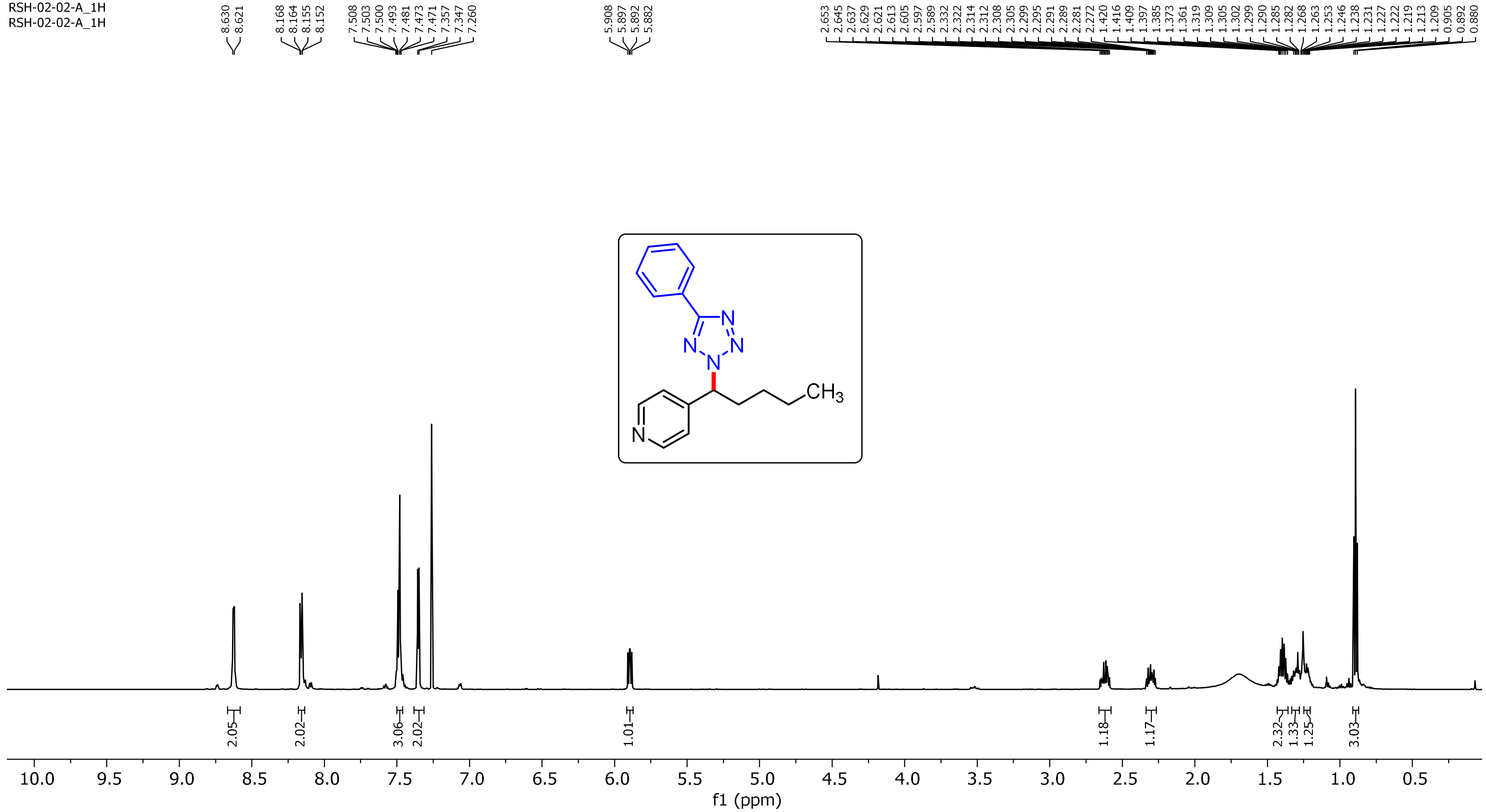

## 4-(1-(5-Phenyl-2*H*-tetrazol-2-yl)pentyl)pyridine (34a): $^{13}$C NMR (151 MHz, $CDCl_3$)

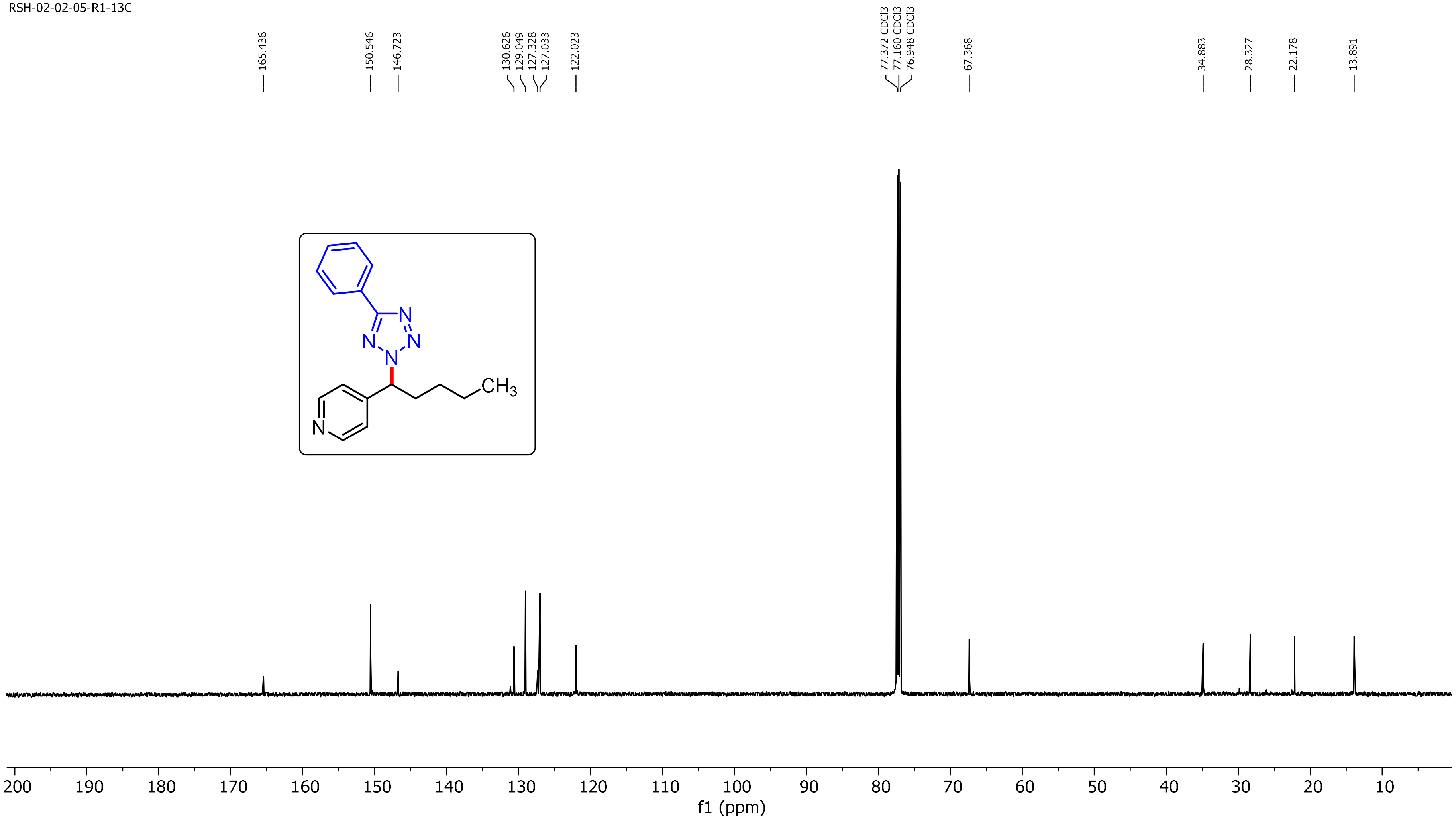

## 3-(5-Phenyl-2*H*-tetrazol-2-yl)butan-1-ol (35a): $^{1}$H NMR (600 MHz, $CDCl_3$)

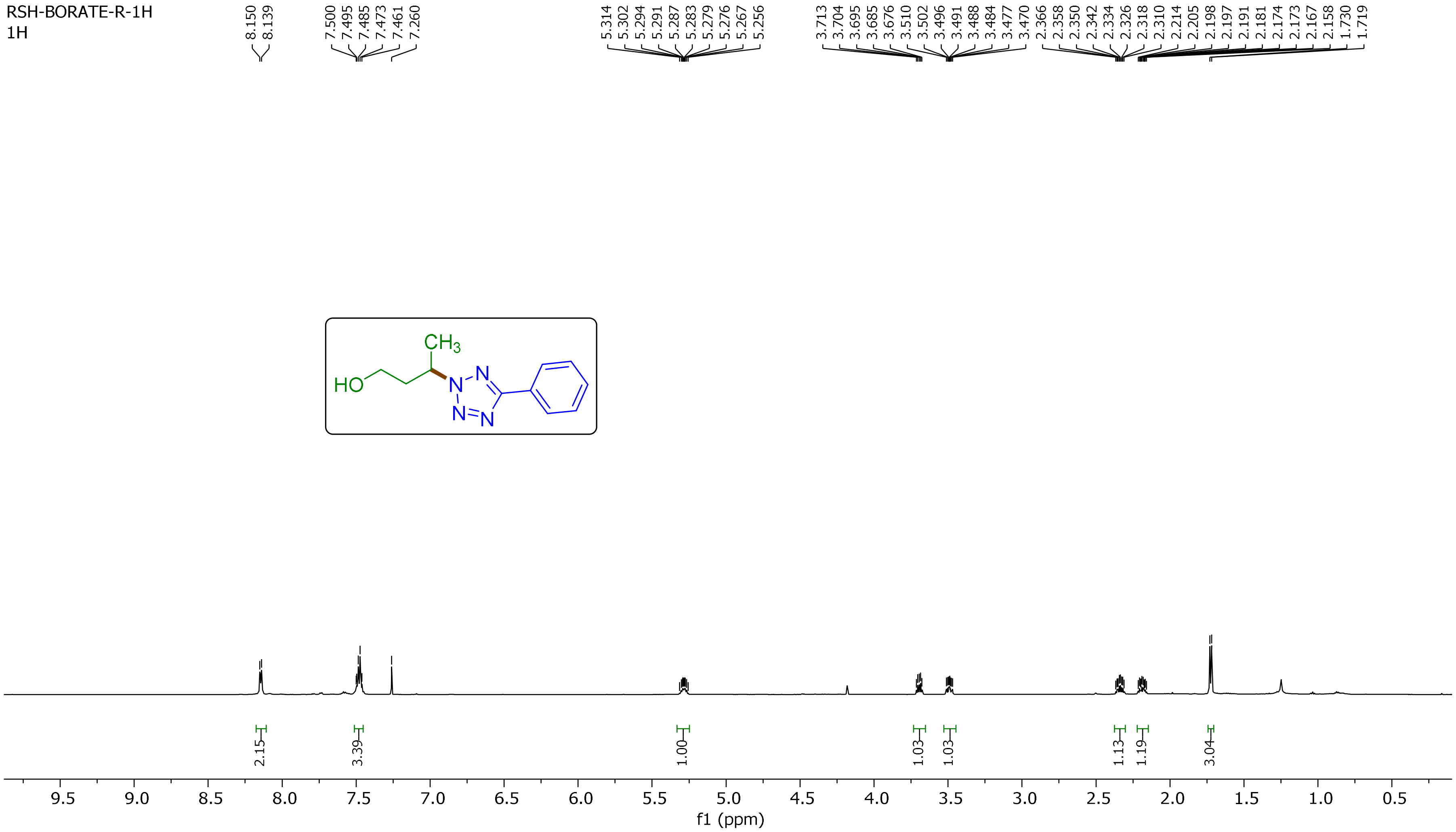

**3-(5-Phenyl-2*H*-tetrazol-2-yl)butan-1-ol (35a): $^{13}$C NMR (151 MHz, CDCl$_3$)**

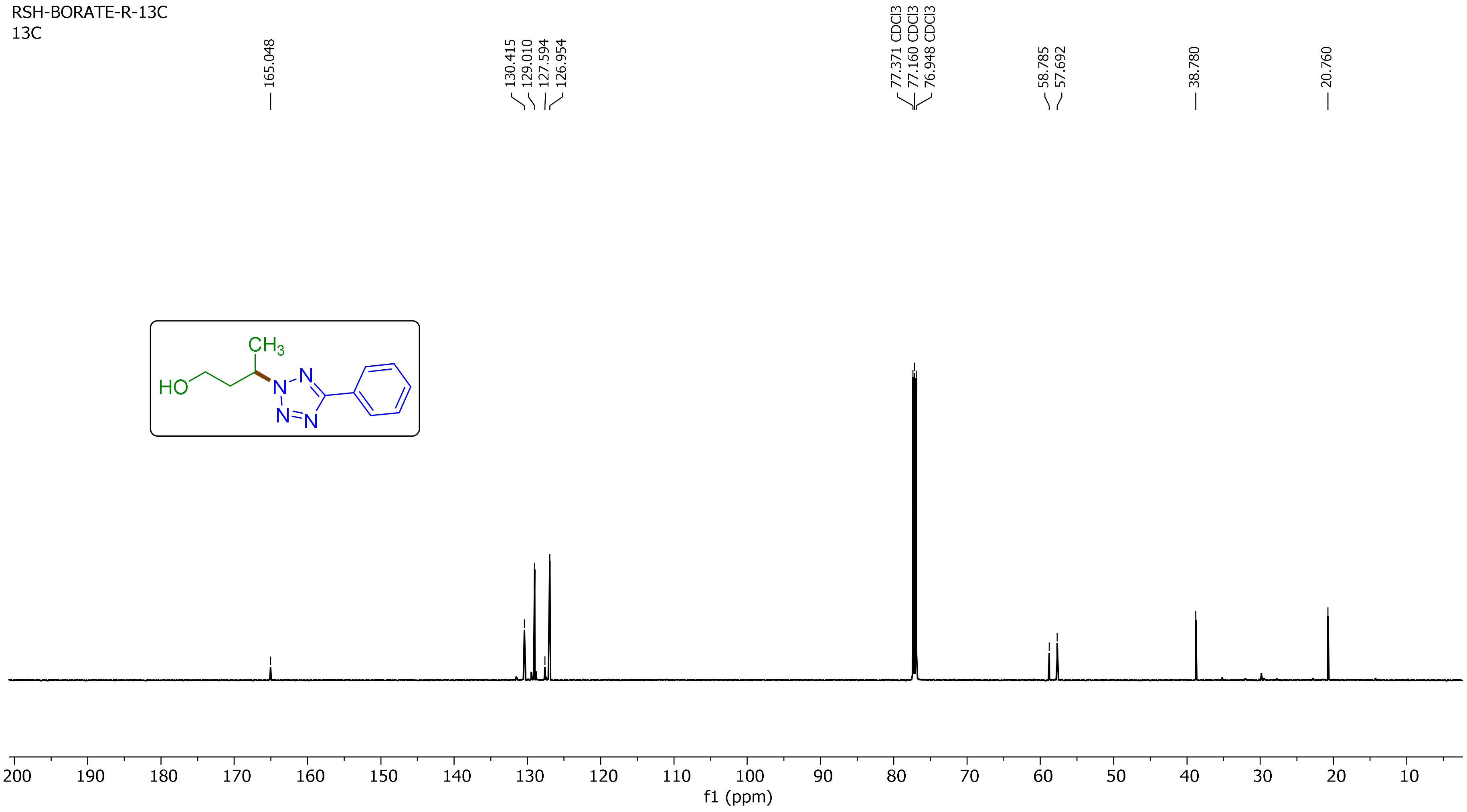

**3-(5-(Naphthalen-2-yl)-2*H*-tetrazol-2-yl)butan-1-ol (35b): $^{1}$H NMR (600 MHz, $CDCl_3$)**

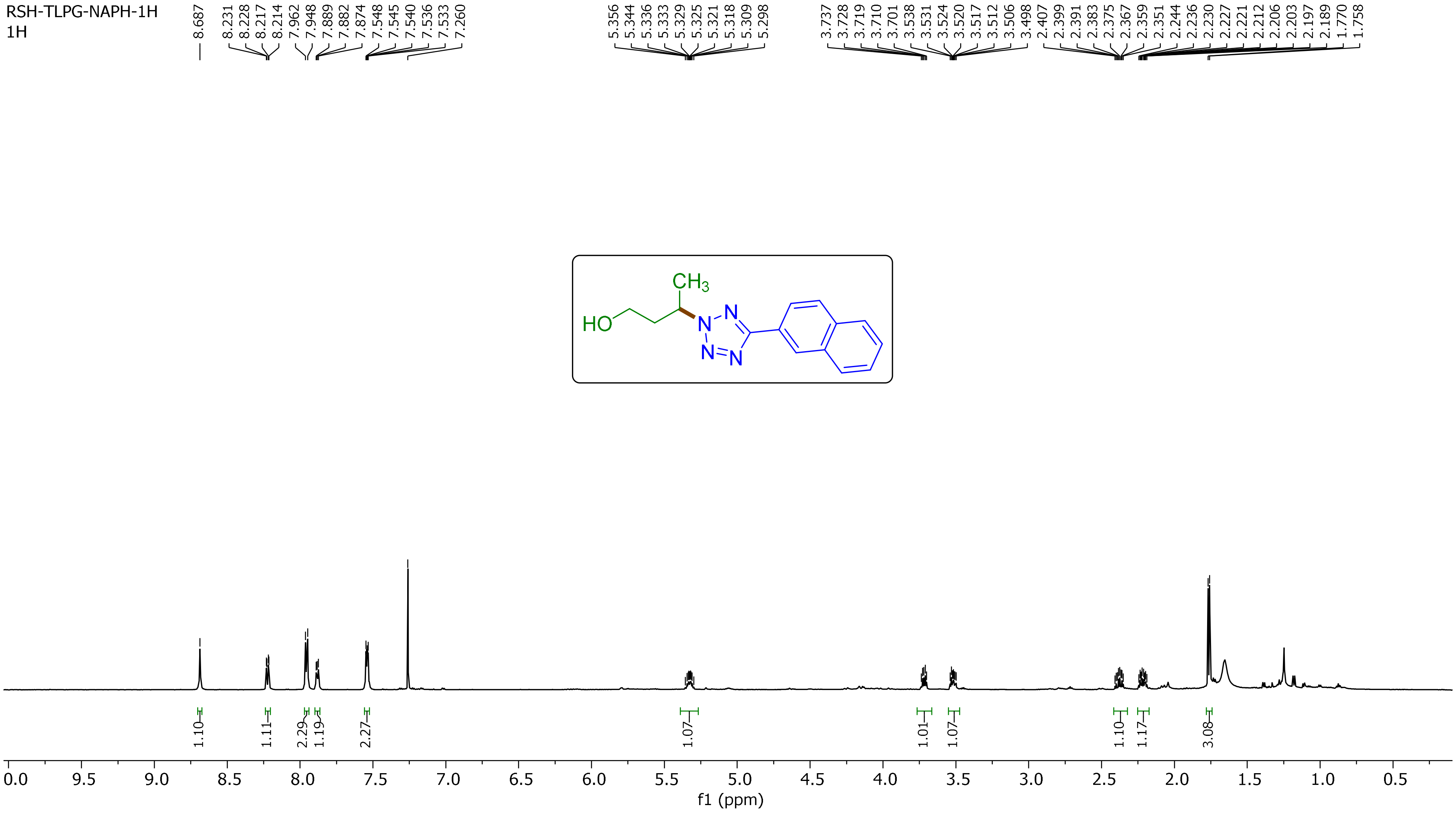

**3-(5-(Naphthalen-2-yl)-2*H*-tetrazol-2-yl)butan-1-ol (35b): $^{13}$C NMR (151 MHz, CDCl$_3$)**

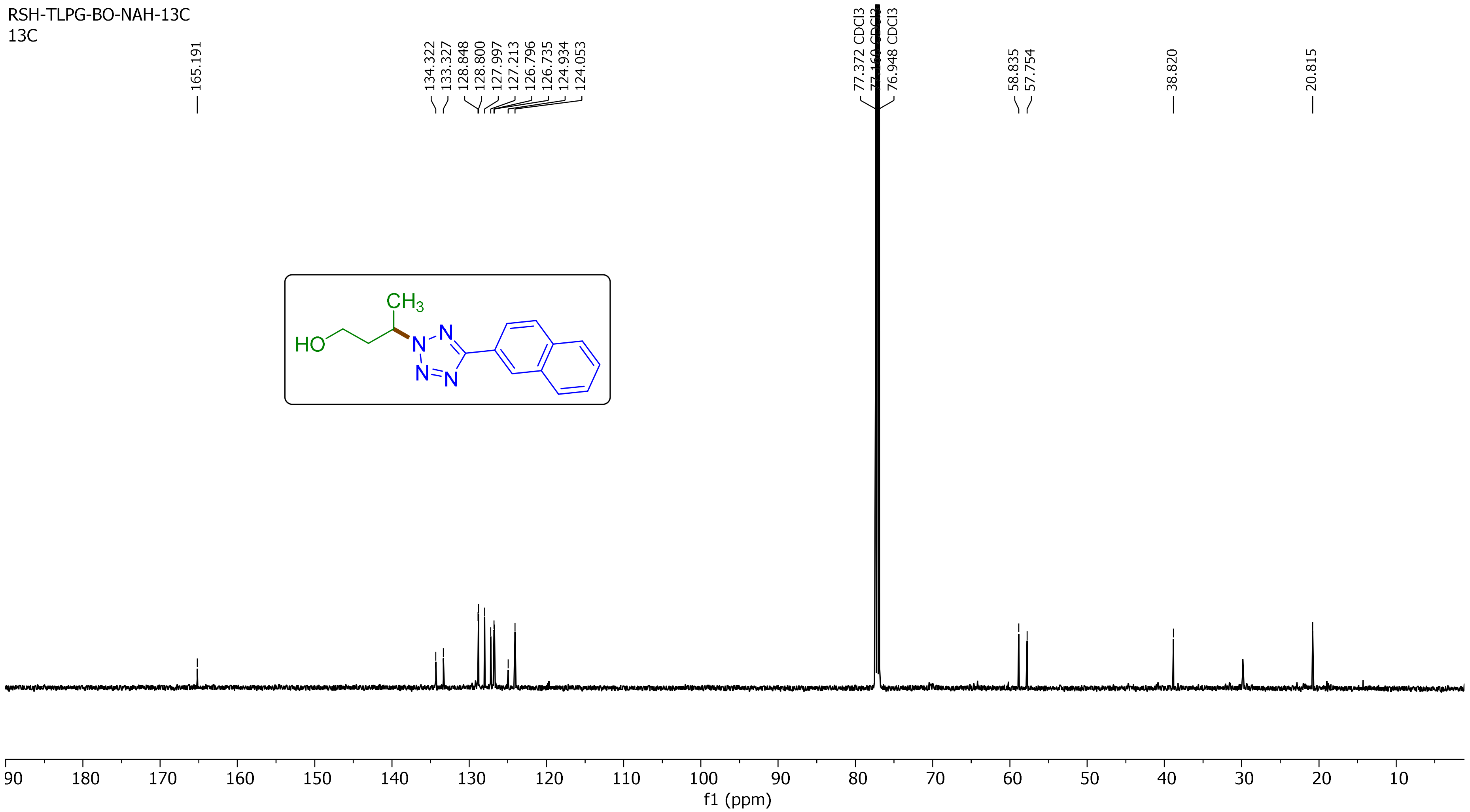

**3-(5-([1,1'-Biphenyl]-4-yl)-2*H*-tetrazol-2-yl)butan-1-ol (35c): $^{1}$H NMR (600 MHz, CDCl$_3$)**

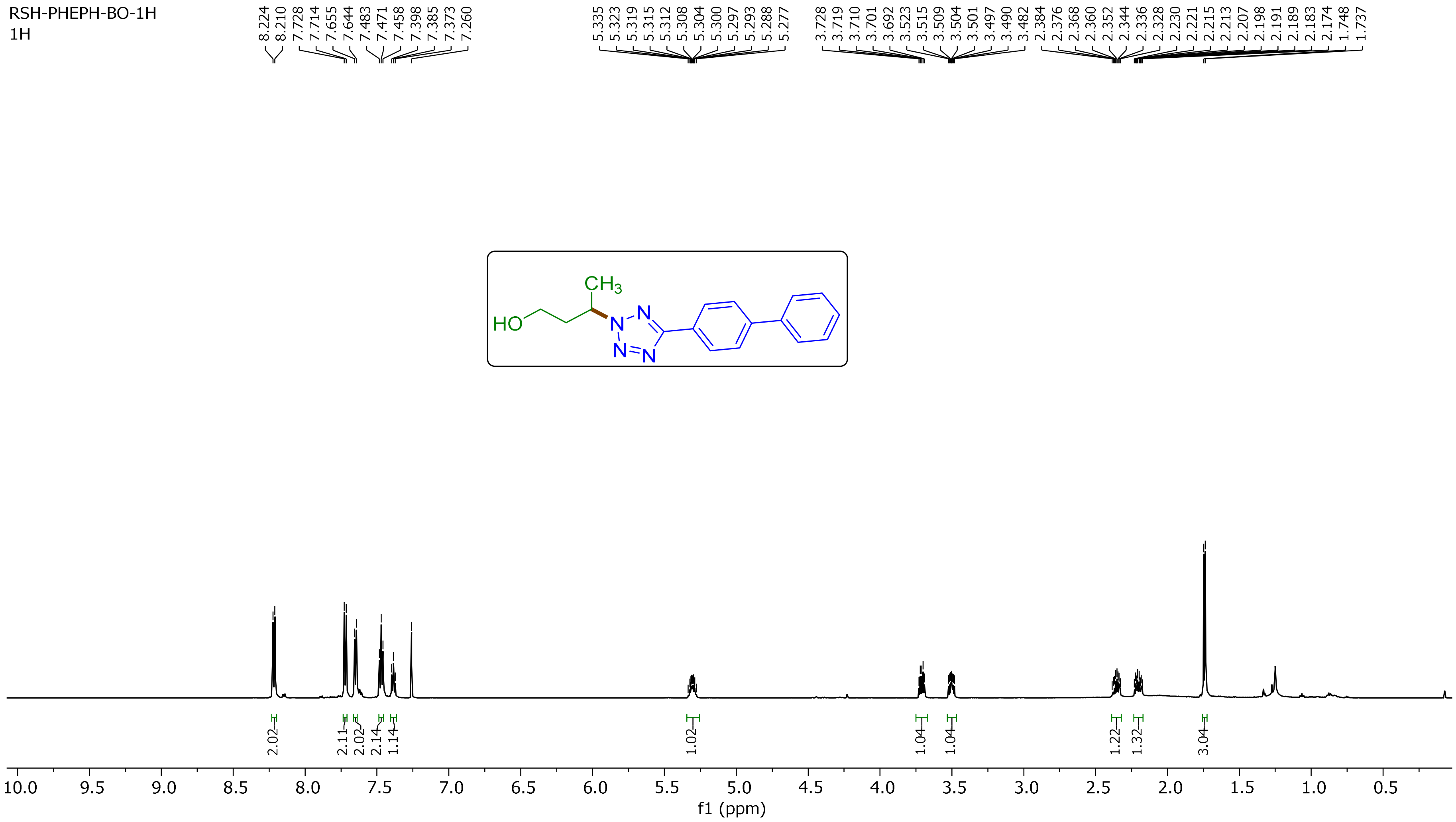

**3-(5-([1,1'-Biphenyl]-4-yl)-2*H*-tetrazol-2-yl)butan-1-ol (35c): $^{13}$C NMR (151 MHz, $CDCl_3$)**

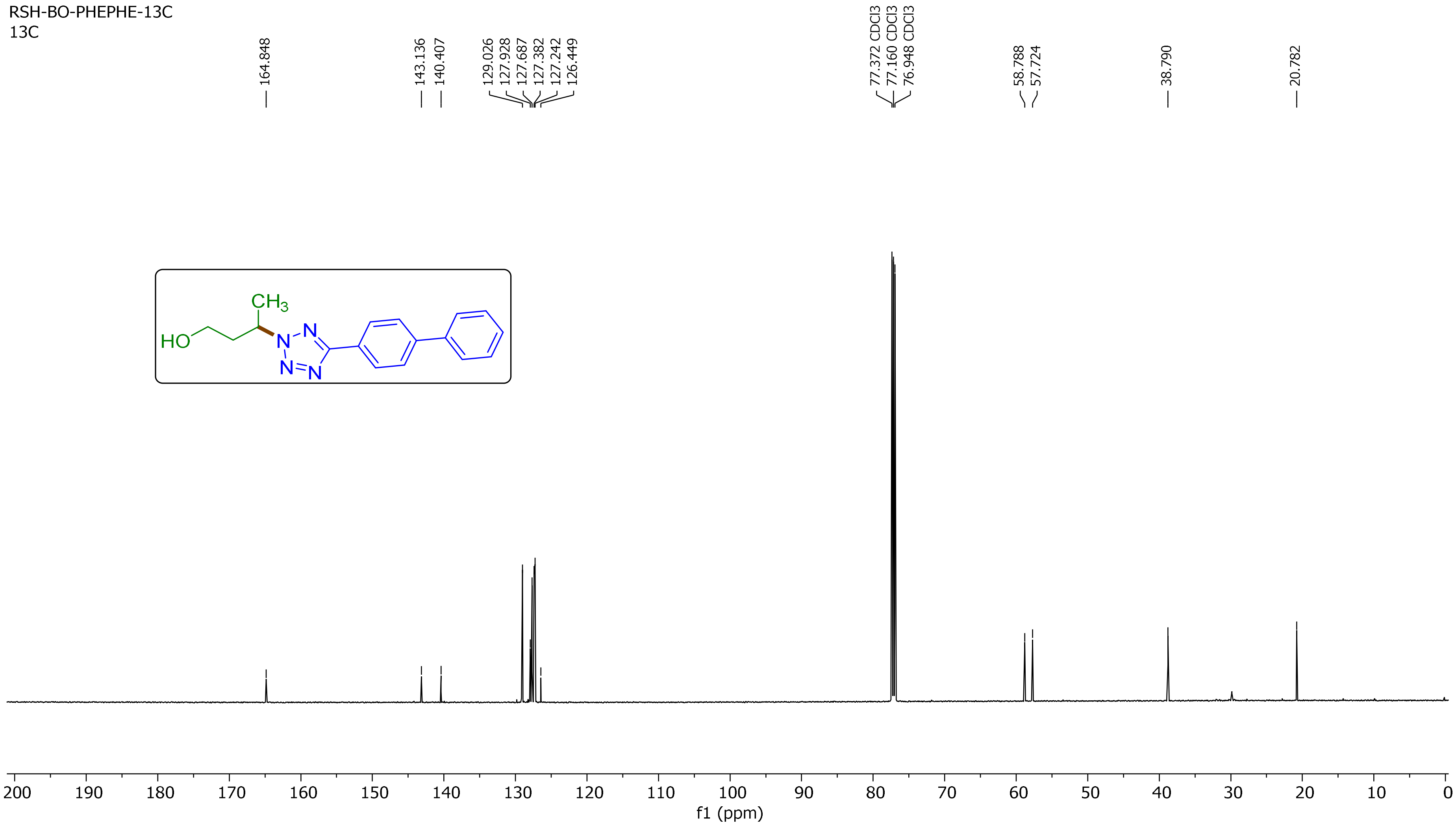

**3-(5-(4-Methoxyphenyl)-2*H*-tetrazol-2-yl)butan-1-ol (35e): $^{1}$H NMR (400 MHz, $CDCl_3$)**

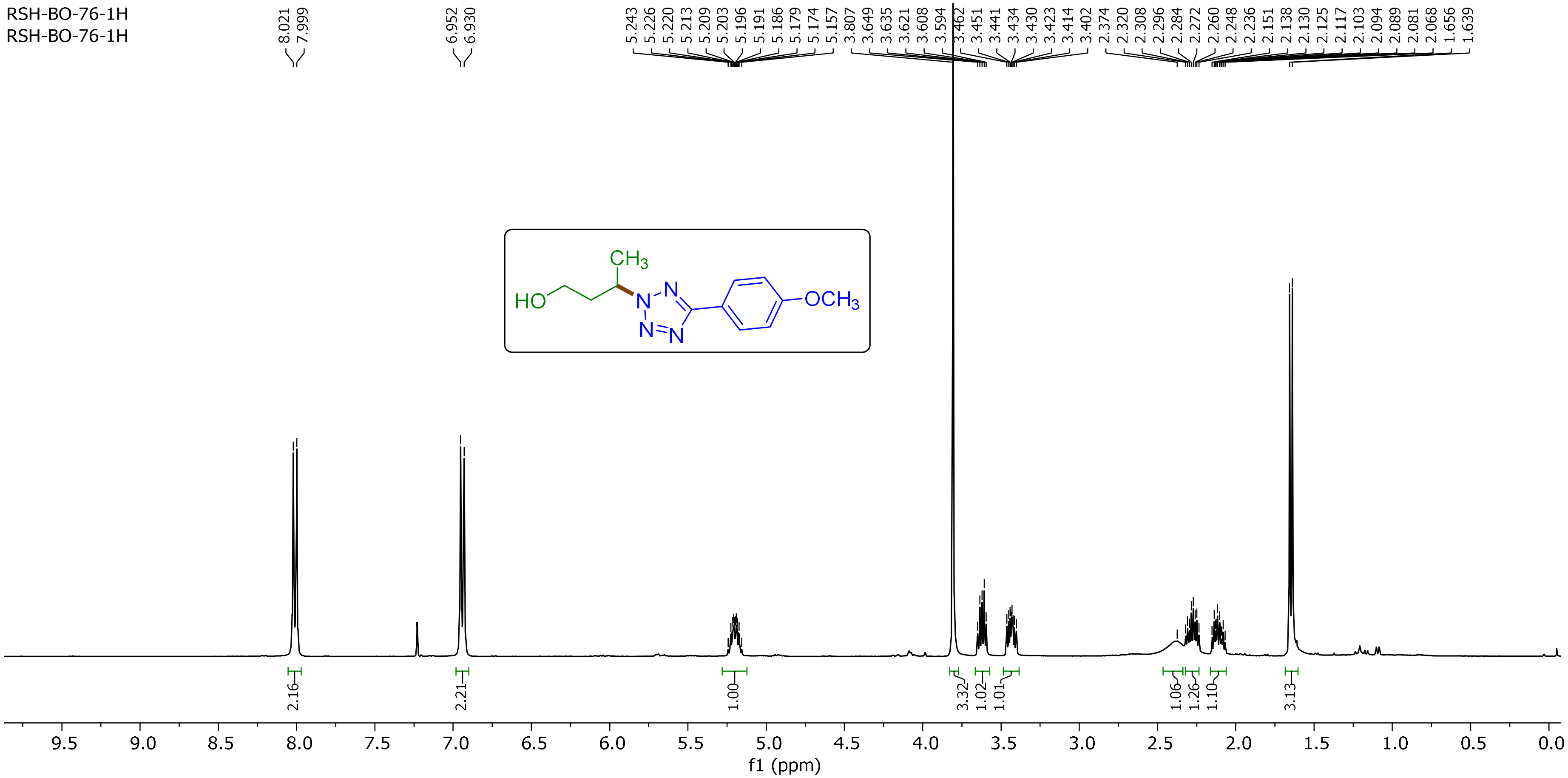

**3-(5-(4-Methoxyphenyl)-2*H*-tetrazol-2-yl)butan-1-ol (35e): $^{13}$C NMR (101 MHz, CDCl$_3$)**

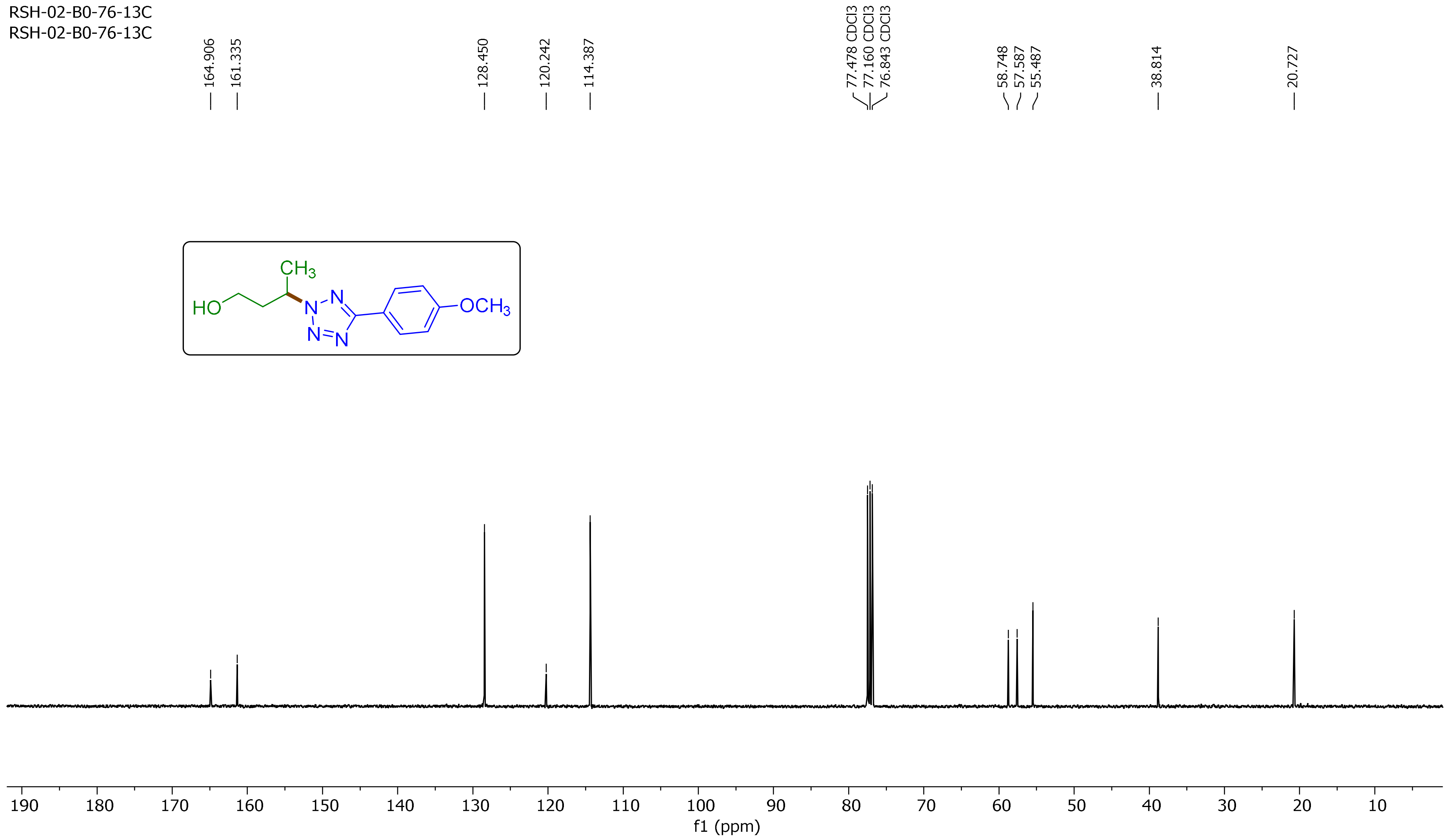

**3-(5-(4-(*tert*-Butyl)phenyl)-2*H*-tetrazol-2-yl)butan-1-ol (35f): $^{1}$H NMR (400 MHz, $CDCl_3$)**

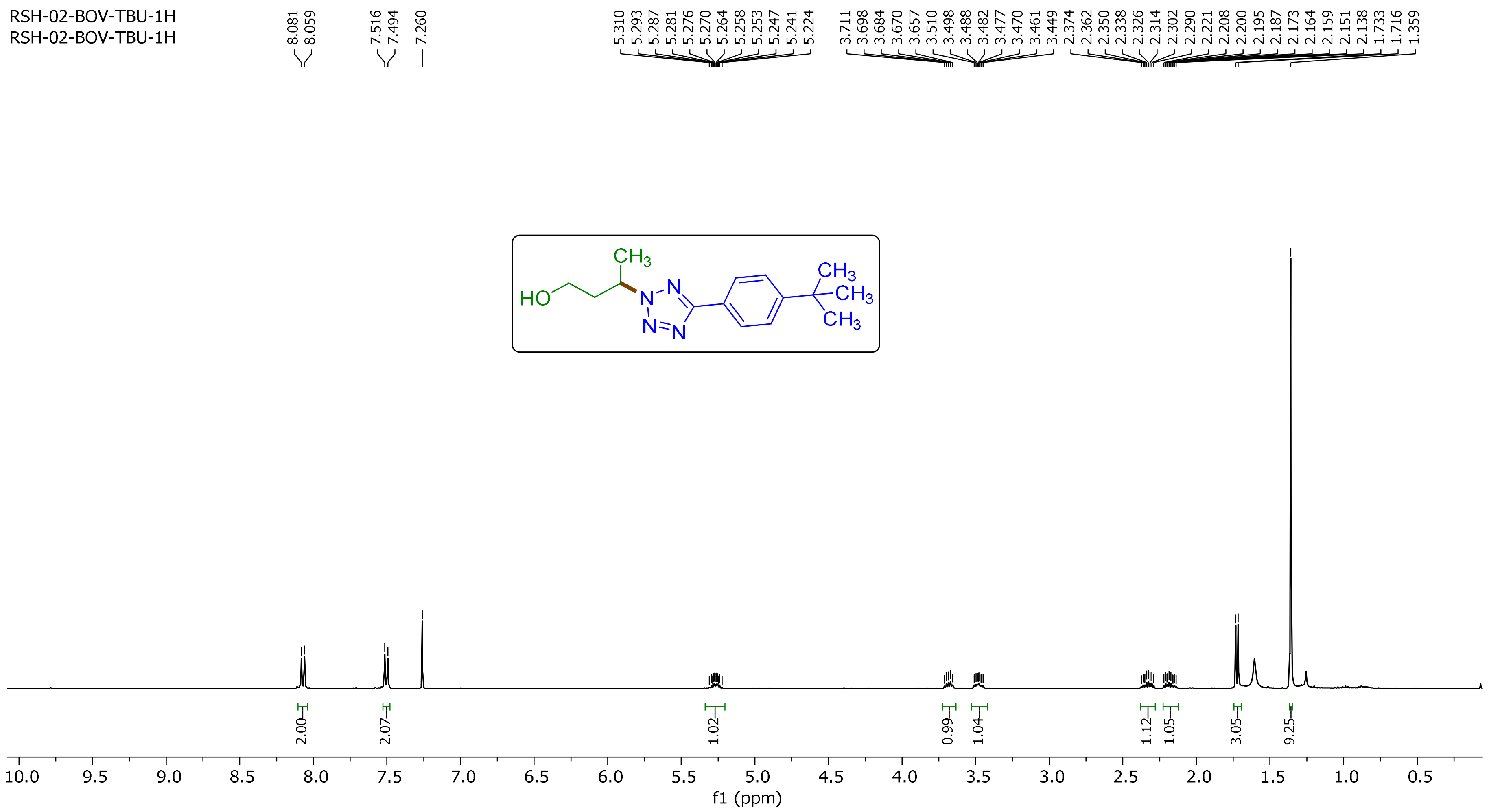

**3-(5-(4-(*tert*-Butyl)phenyl)-2*H*-tetrazol-2-yl)butan-1-ol (35f): $^{13}$C NMR (101 MHz, $CDCl_3$)**

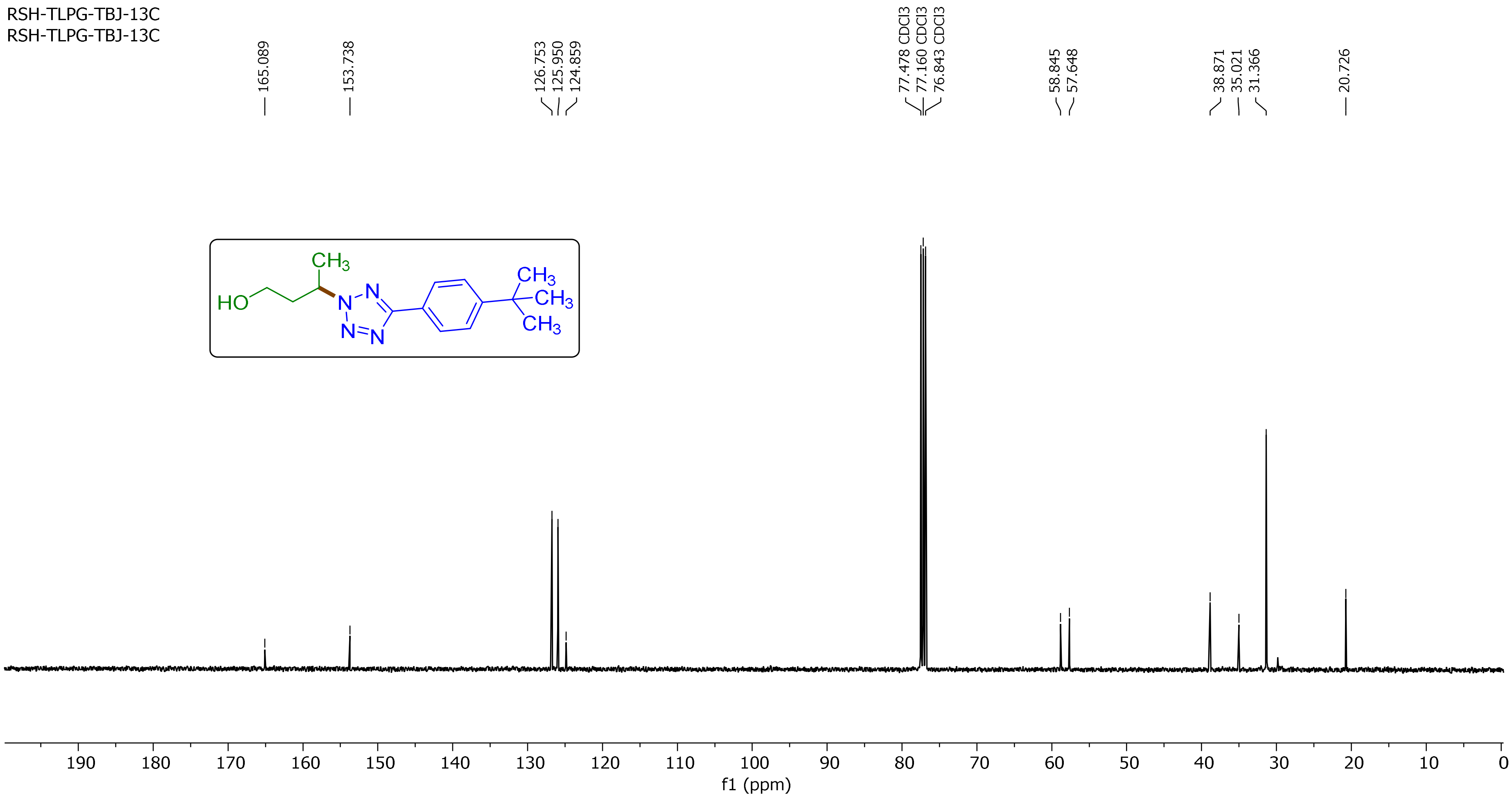

**3-(5-(3-Methoxyphenyl)-2*H*-tetrazol-2-yl)butan-1-ol (35g): $^{1}$H NMR (600 MHz, $CDCl_3$)**

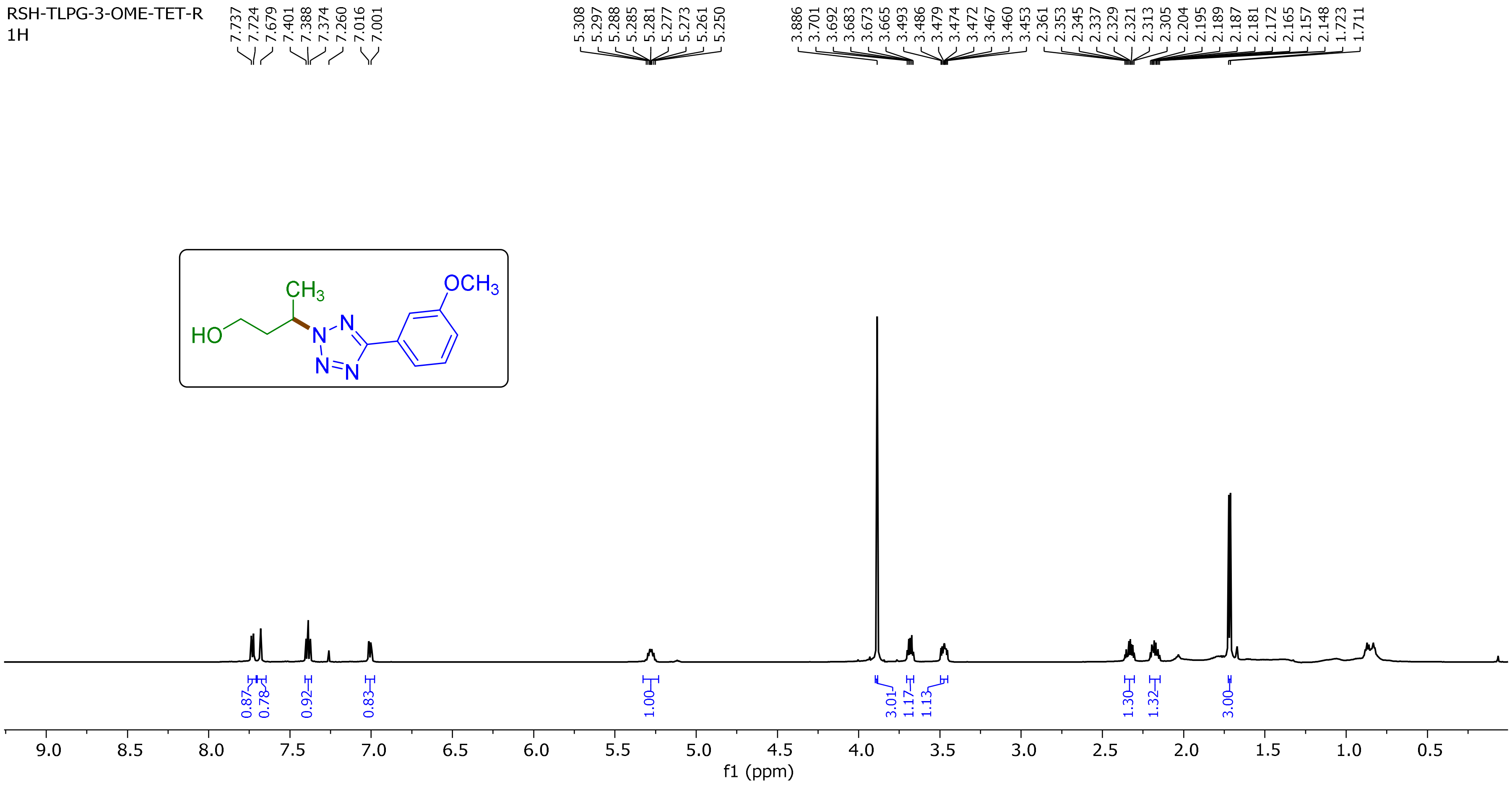

**3-(5-(3-Methoxyphenyl)-2*H*-tetrazol-2-yl)butan-1-ol (35g): $^{13}$C NMR (151 MHz, $CDCl_3$)**

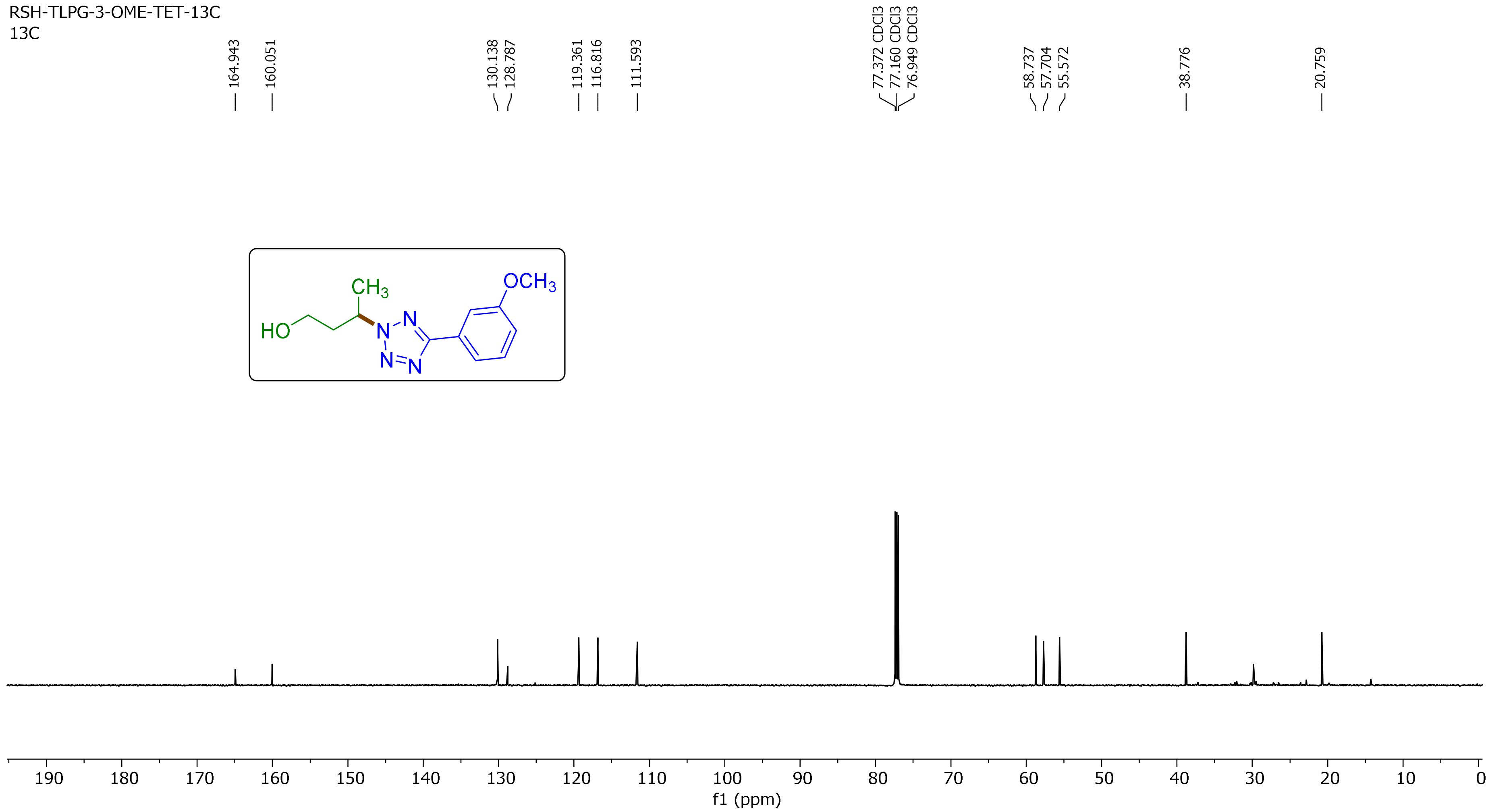

**3-(5-(4-Fluorophenyl)-2*H*-tetrazol-2-yl)butan-1-ol (35y): $^1$H NMR (600 MHz, $CDCl_3$)**

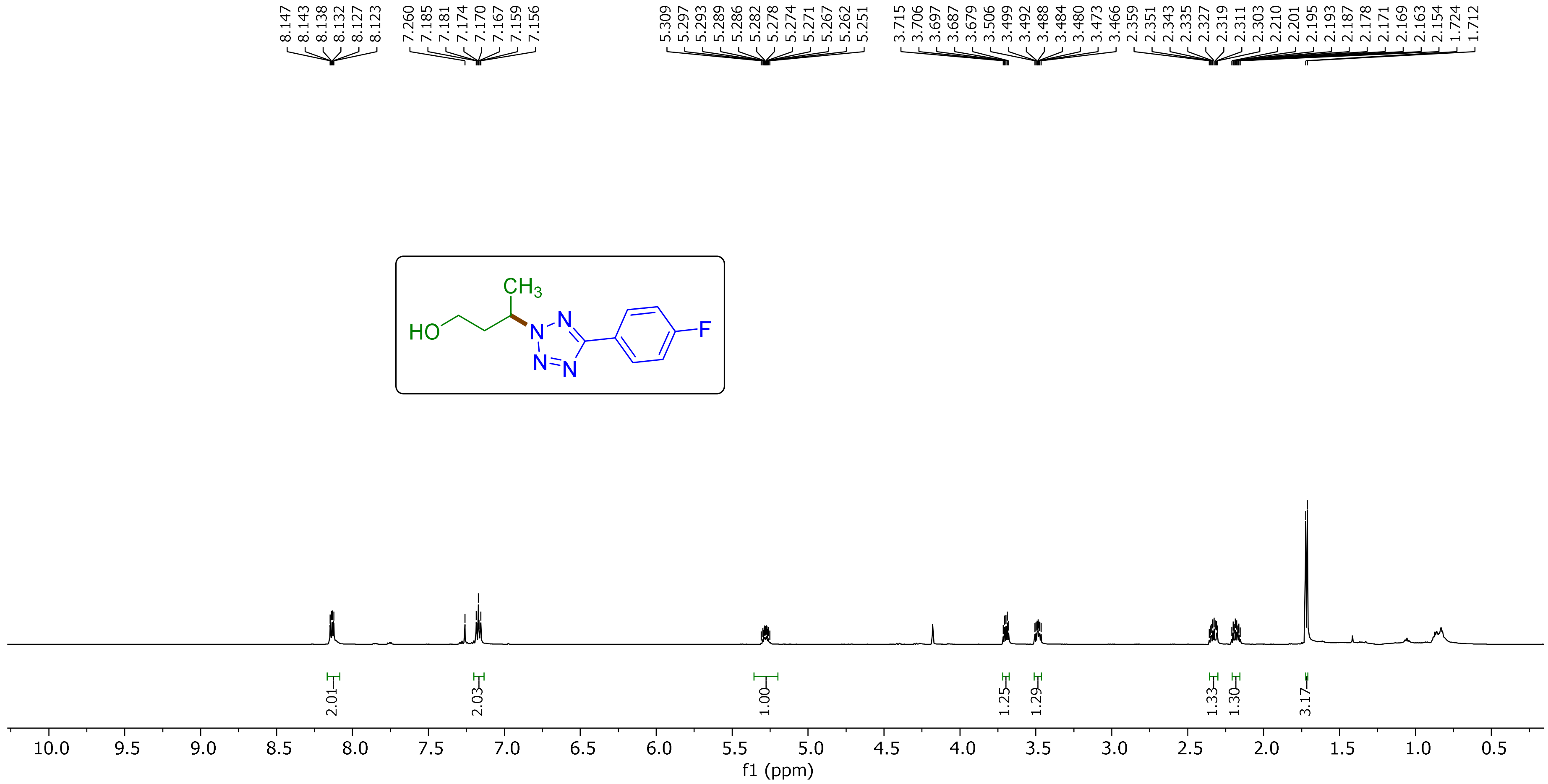

**3-(5-(4-Fluorophenyl)-2*H*-tetrazol-2-yl)butan-1-ol (35y): $^{13}$C NMR (151 MHz, CDCl$_3$)**

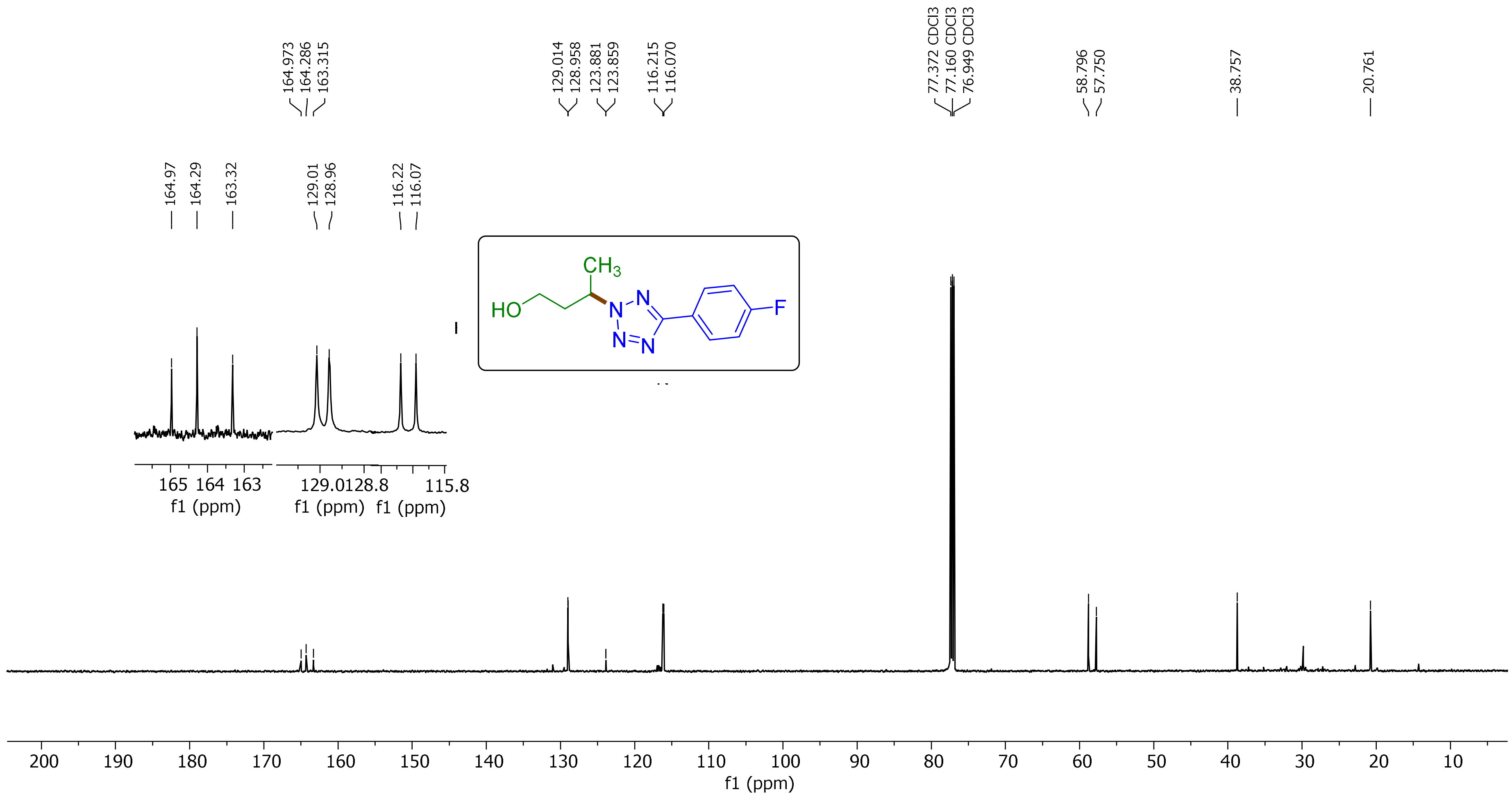

**3-(5-(4-Fluorophenyl)-2*H*-tetrazol-2-yl)butan-1-ol (35y): $^{19}$F {$^{1}$H} NMR (565 MHz, CDCl$_3$)**

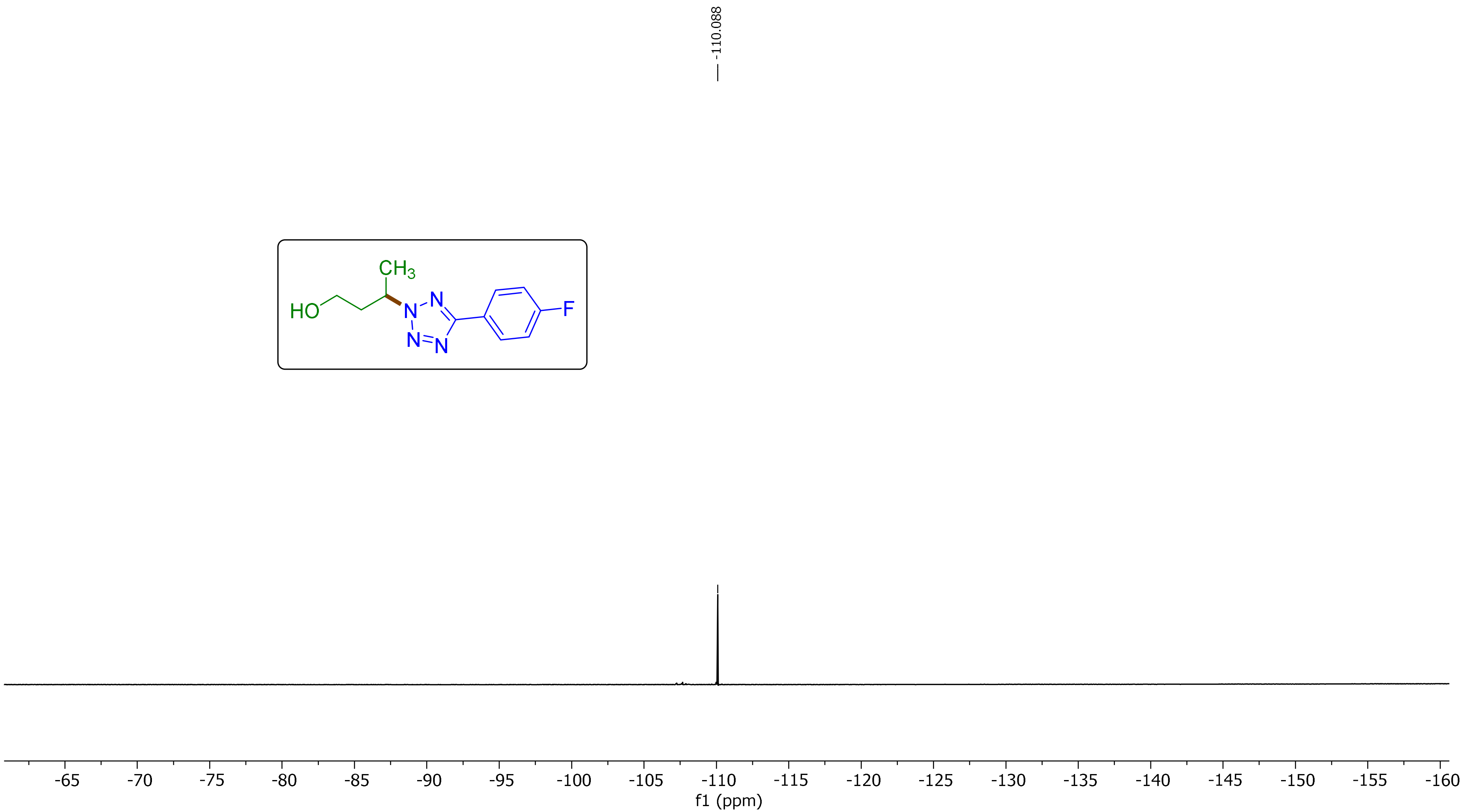

**3-(5-(4-Bromophenyl)-2*H*-tetrazol-2-yl)butan-1-ol (35i): $^{1}$H NMR (600 MHz, $CDCl_3$)**

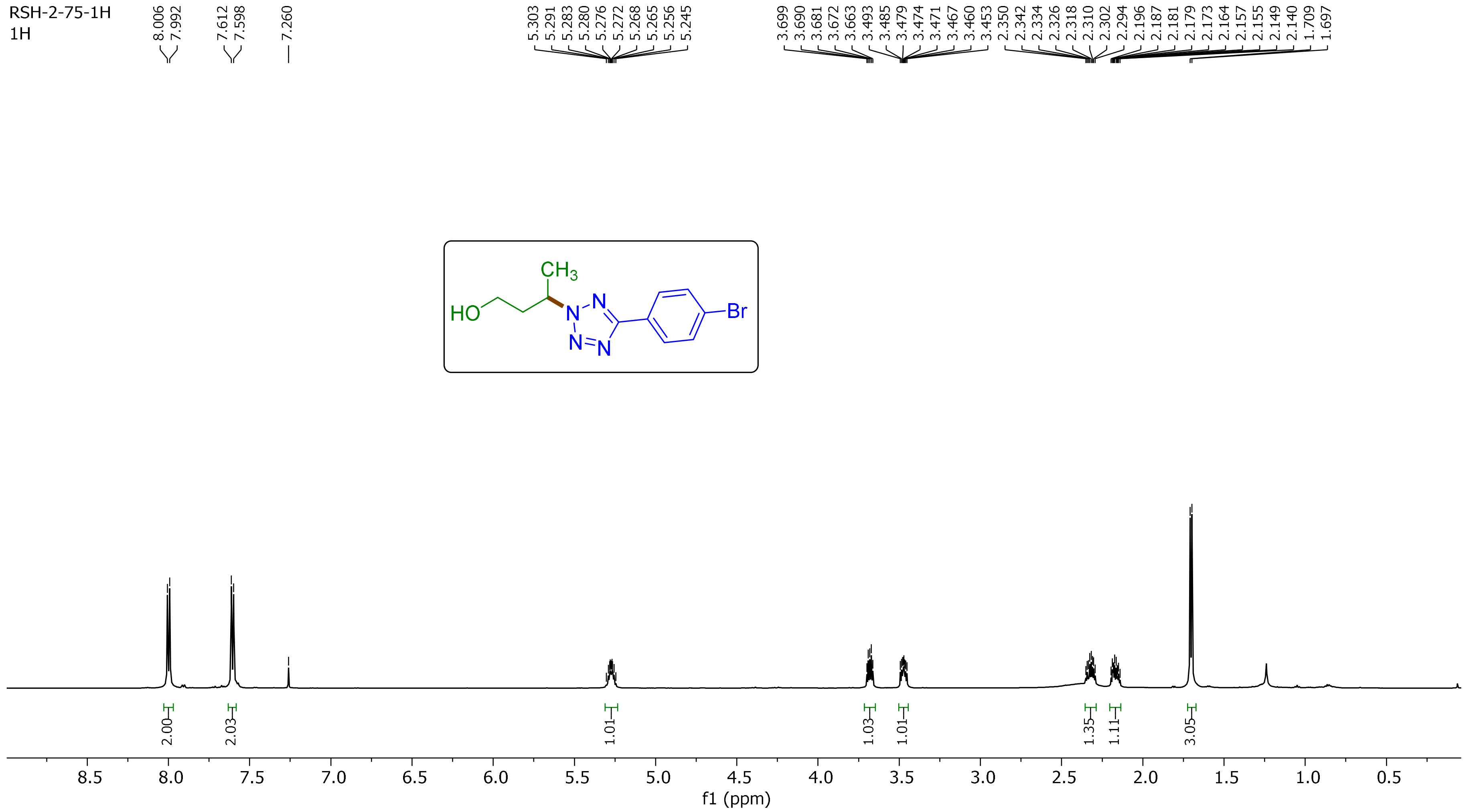

**3-(5-(4-Bromophenyl)-2*H*-tetrazol-2-yl)butan-1-ol (35i): $^{13}$C NMR (151 MHz, $CDCl_3$)**

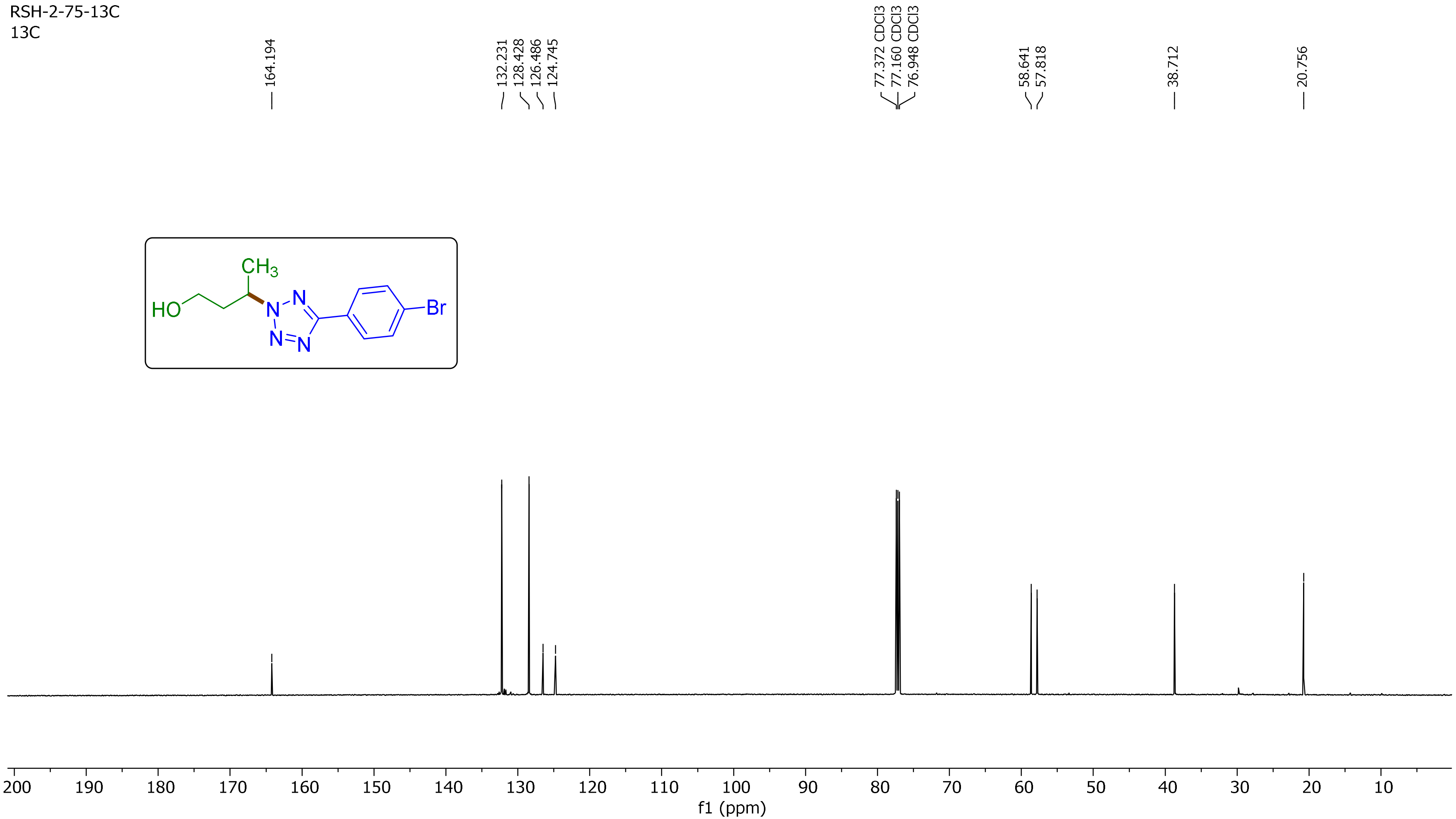

**3-(5-(4-(Trifluoromethyl)phenyl)-2*H*-tetrazol-2-yl)butan-1-ol (35j): $^{1}$H NMR (600 MHz, $CDCl_3$)**

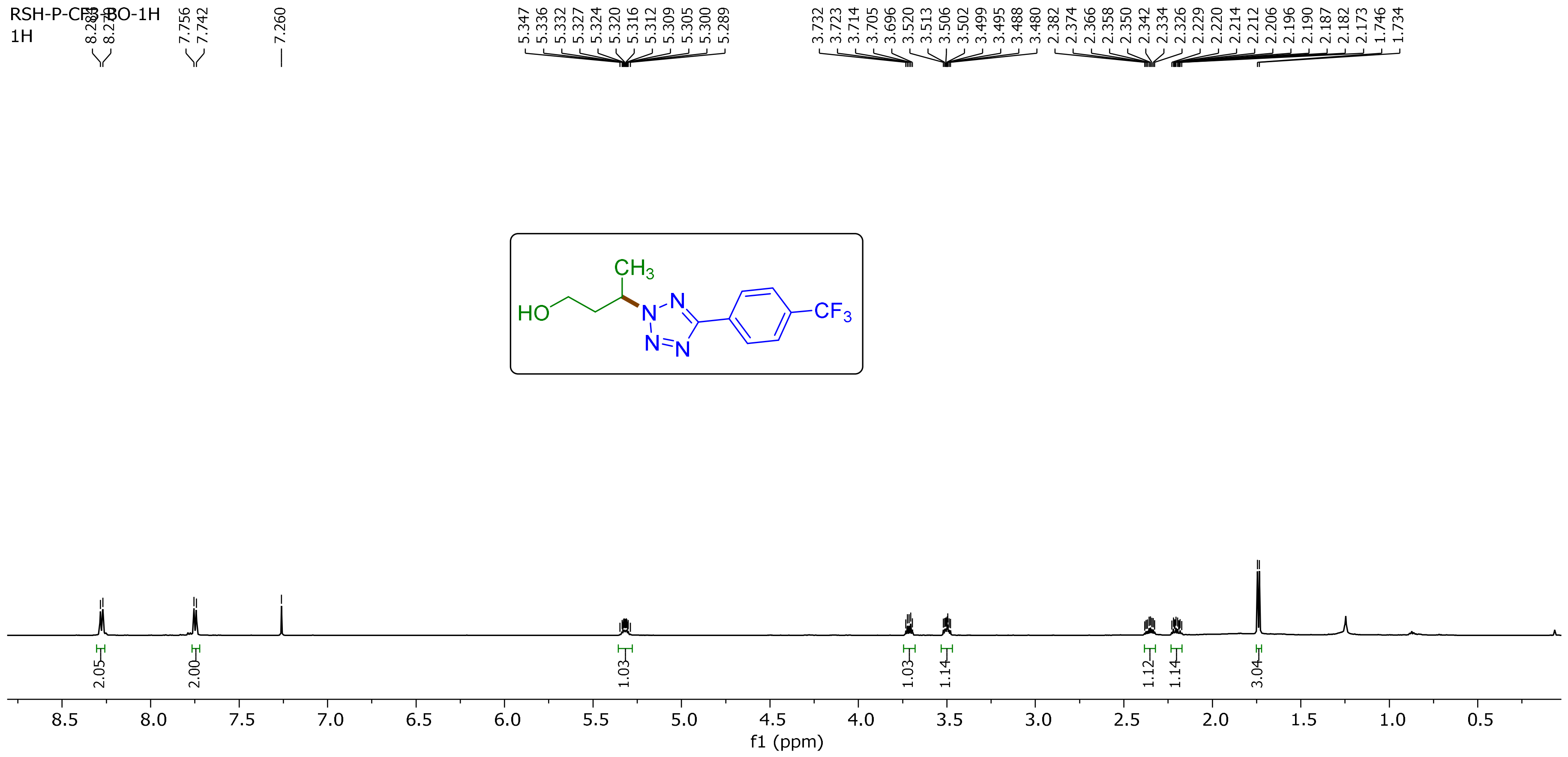

**3-(5-(4-(Trifluoromethyl)phenyl)-2*H*-tetrazol-2-yl)butan-1-ol (35j): $^{13}$C NMR (151 MHz, CDCl₃)**

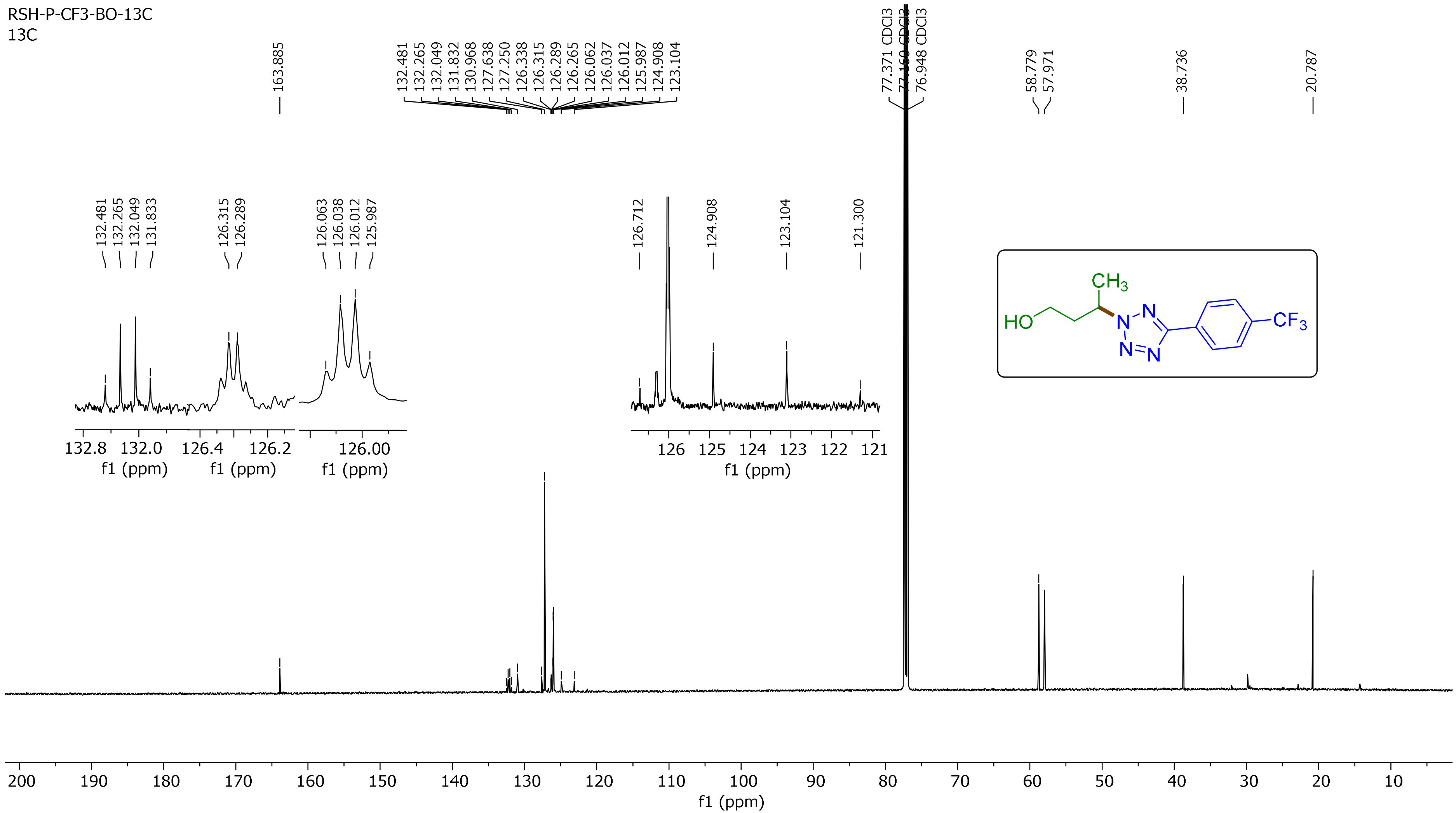

**3-(5-(4-(Trifluoromethyl)phenyl)-2*H*-tetrazol-2-yl)butan-1-ol (35j): $^{19}$F NMR (377 MHz, CDCl$_3$)**

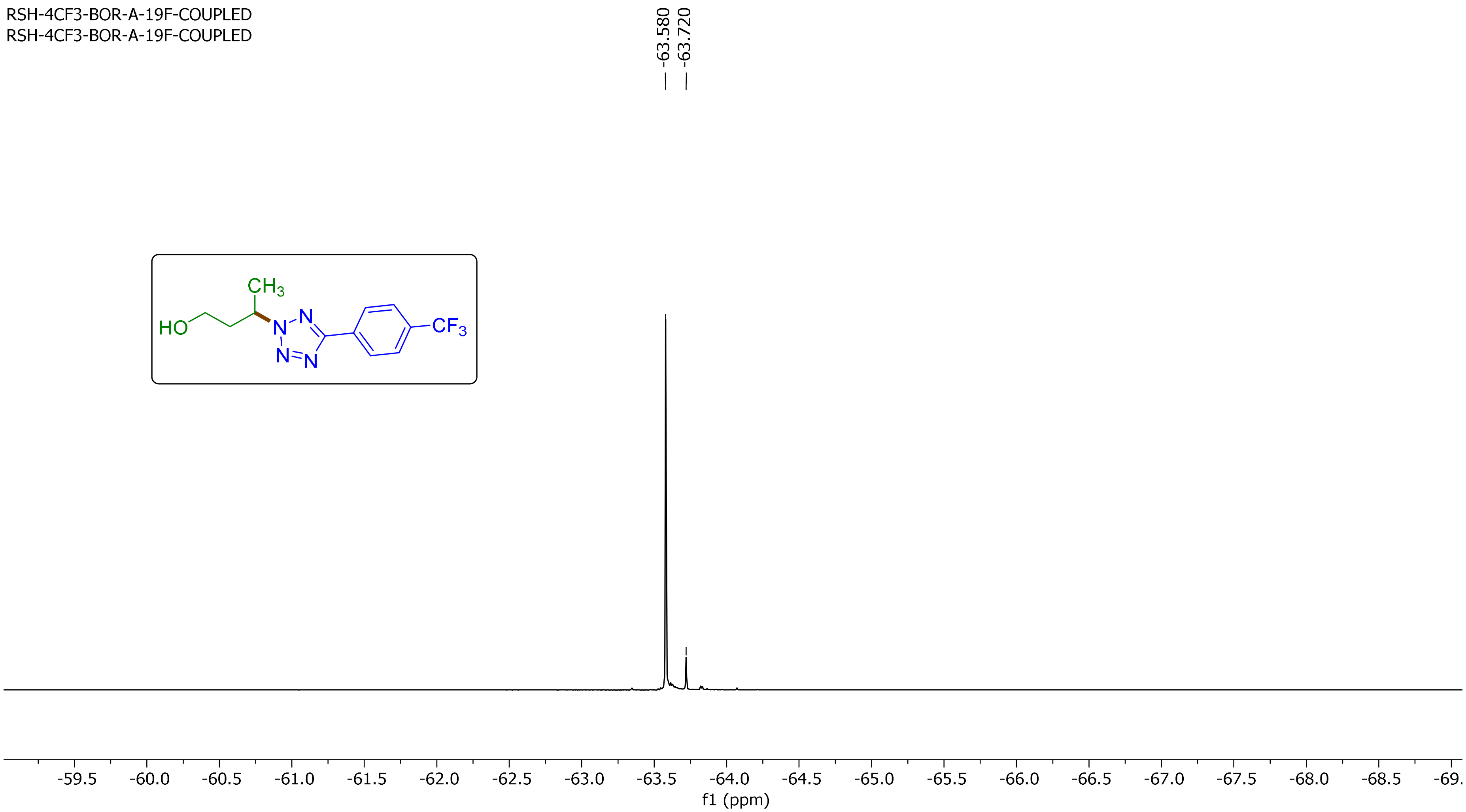

**3-(5-(4-Nitrophenyl)-2*H*-tetrazol-2-yl)butan-1-ol (35k): $^1$H NMR (400 MHz, $CDCl_3$)**

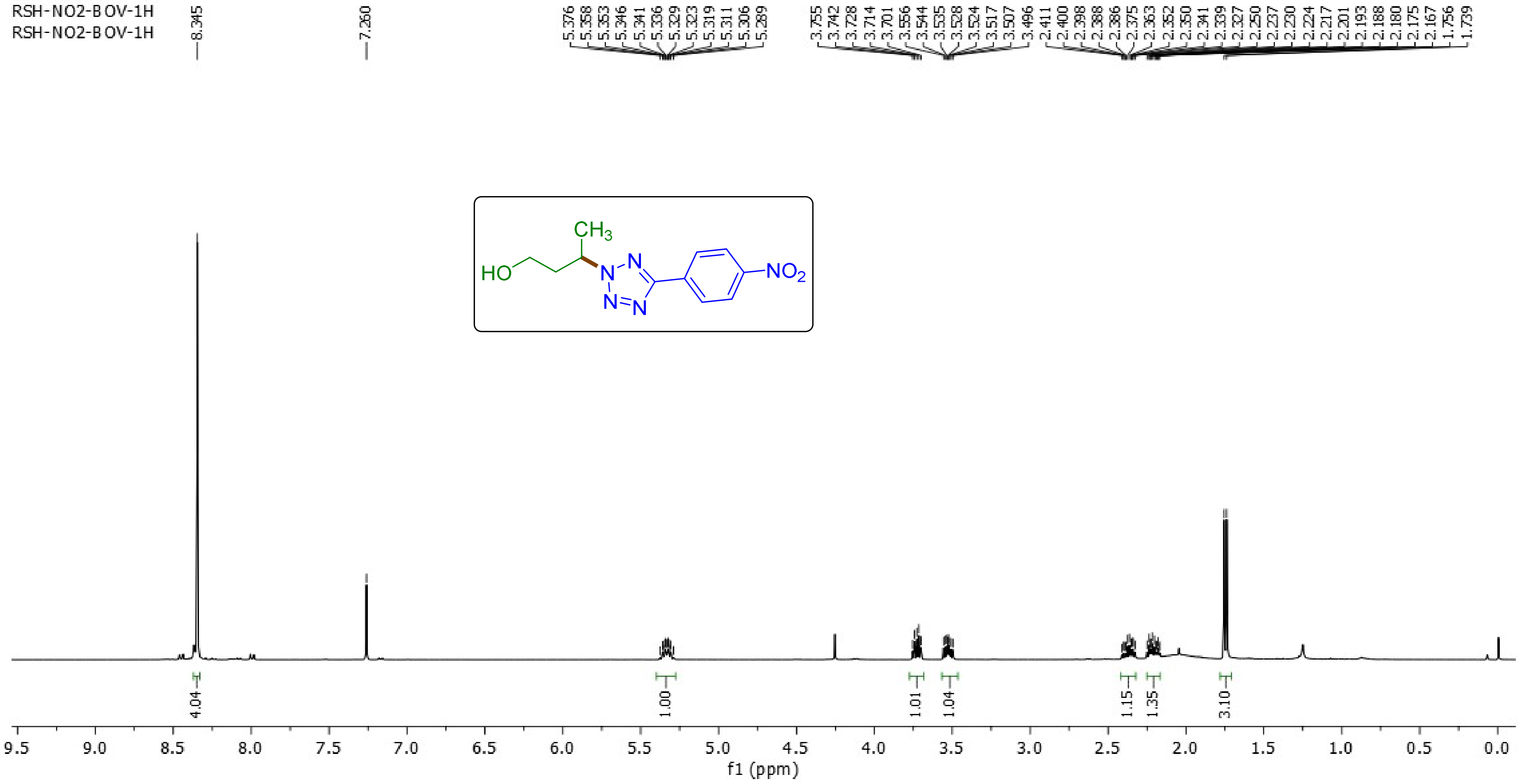

**3-(5-(4-Nitrophenyl)-2*H*-tetrazol-2-yl)butan-1-ol (35k): $^{13}$C NMR (101 MHz, CDCl$_3$)**

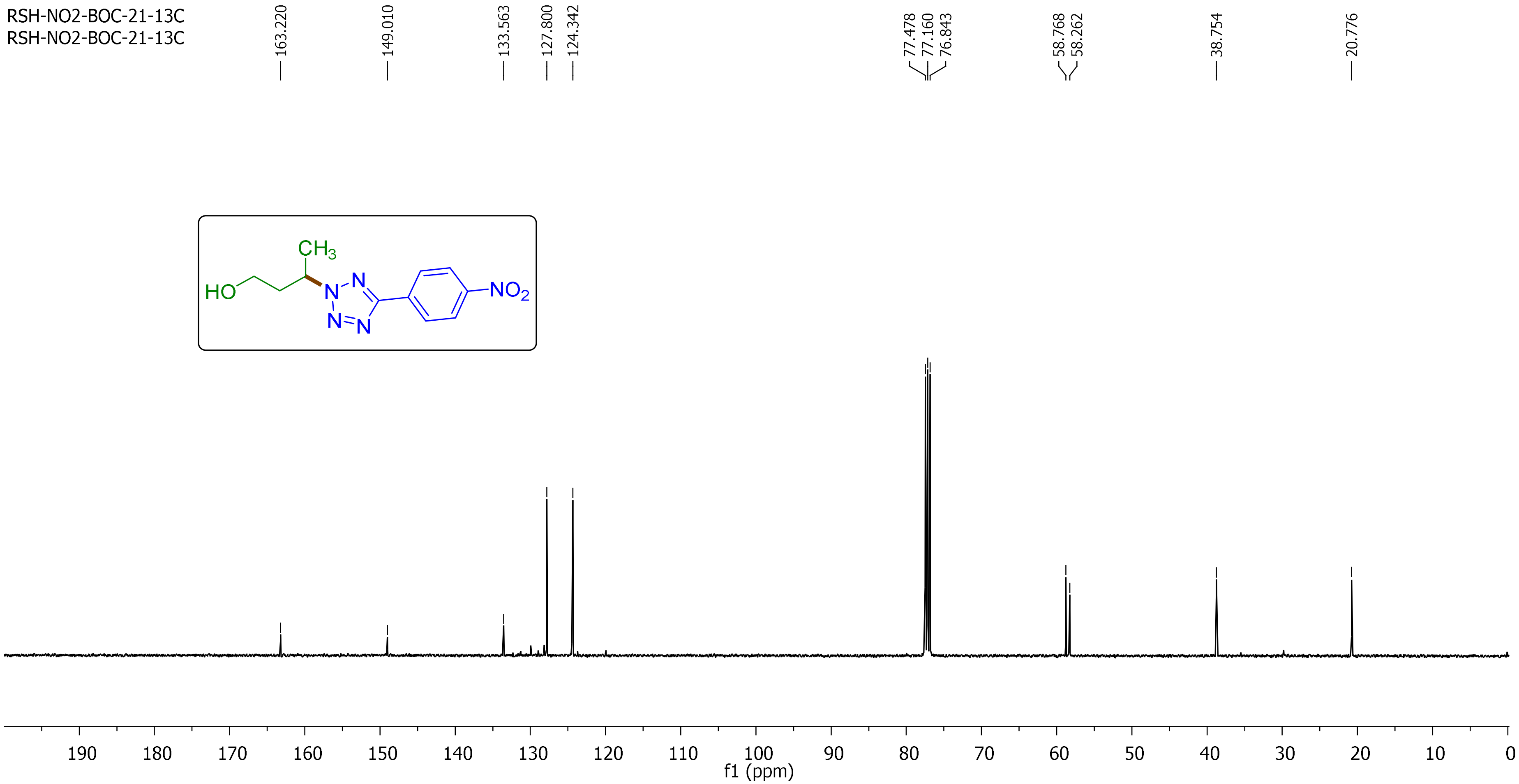

**3-(5-(2-Bromophenyl)-2*H*-tetrazol-2-yl)butan-1-ol (35m): $^{1}$H NMR (600 MHz, $CDCl_3$)**

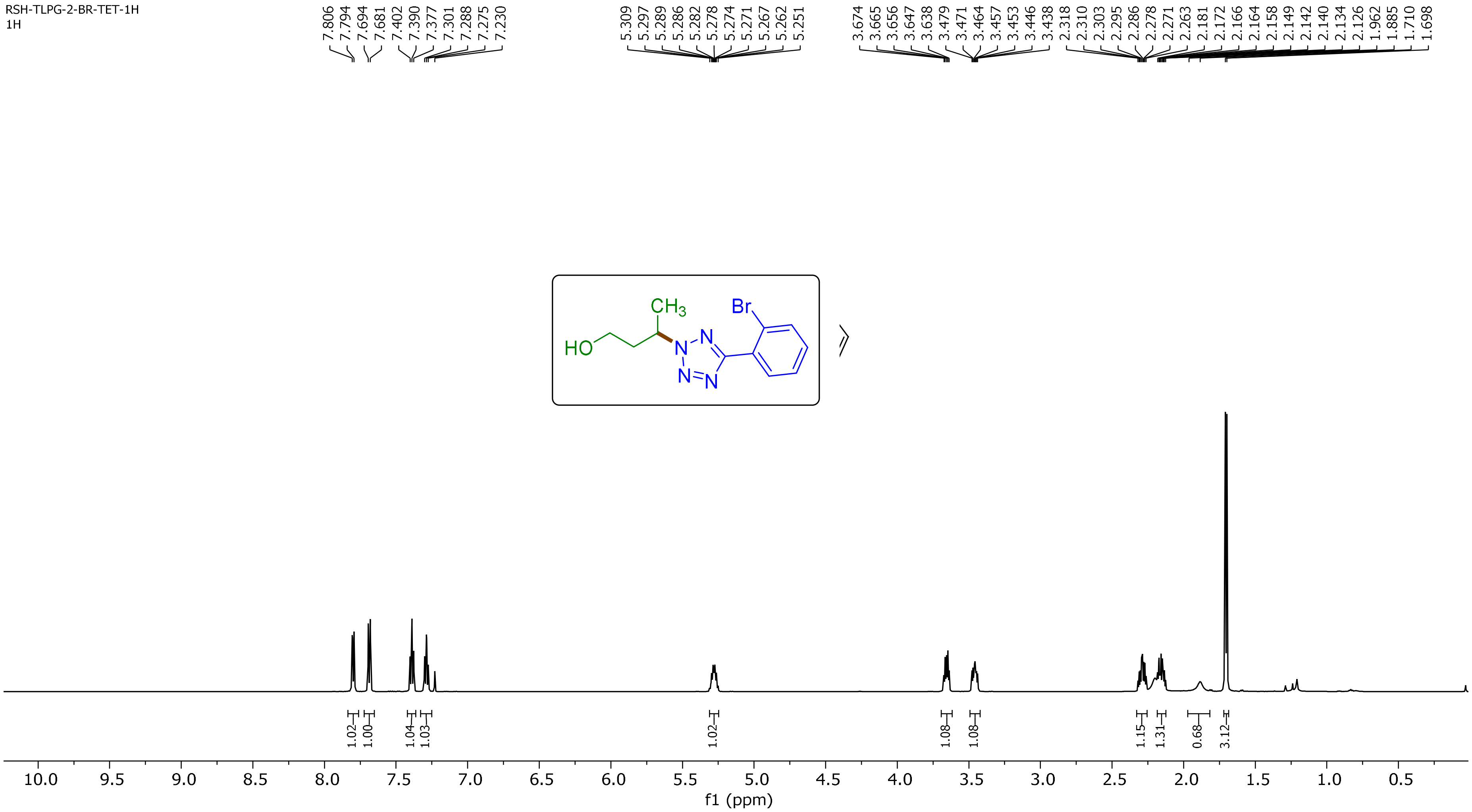

**3-(5-(2-Bromophenyl)-2*H*-tetrazol-2-yl)butan-1-ol (35m)**: $^{13}$**C NMR (151 MHz, CDCl$_3$)**

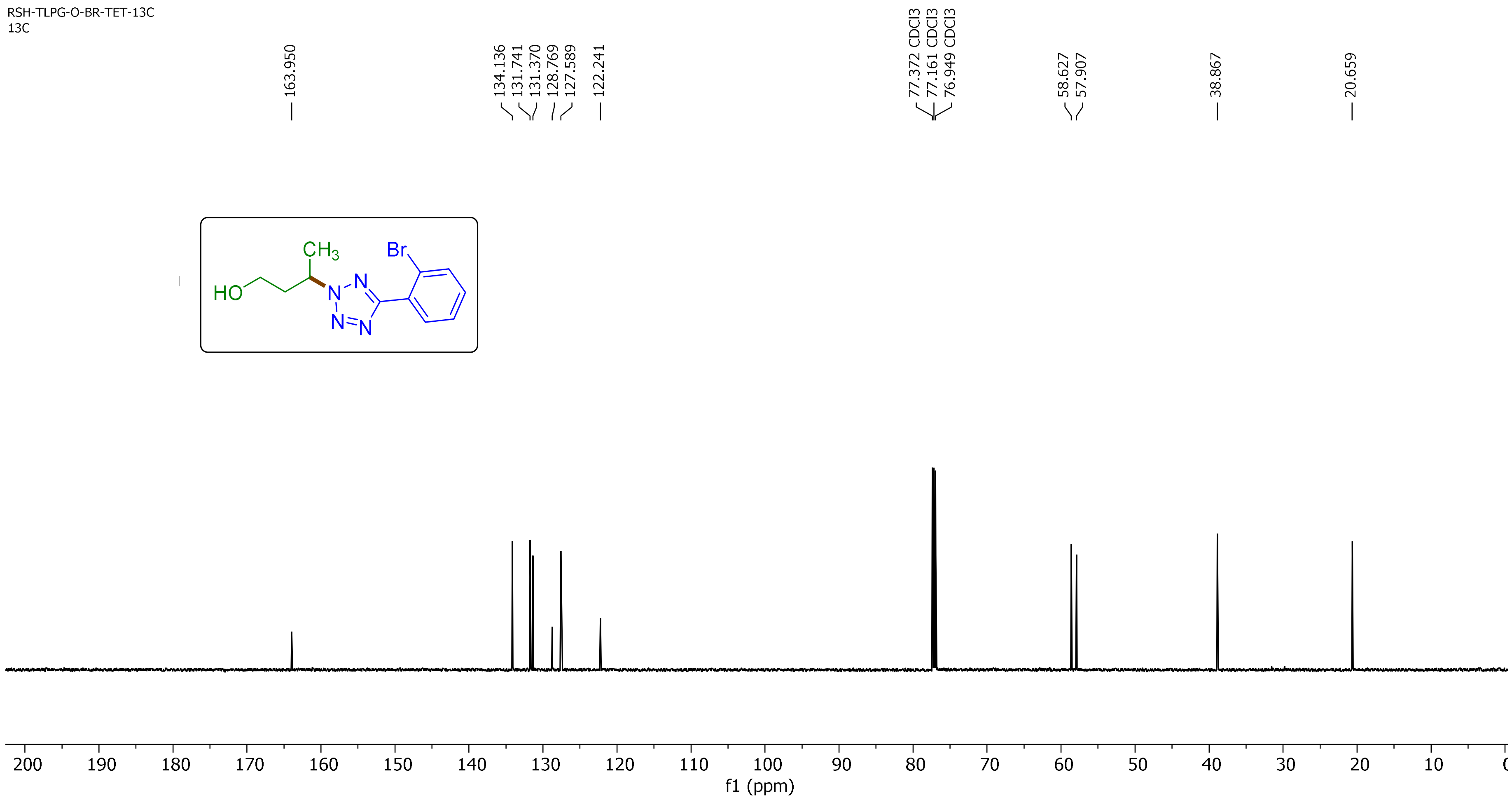

**(*E*)-3-(5-Styryl-2*H*-tetrazol-2-yl)butan-1-ol (35n): $^{1}$H NMR (600 MHz, $CDCl_3$)**

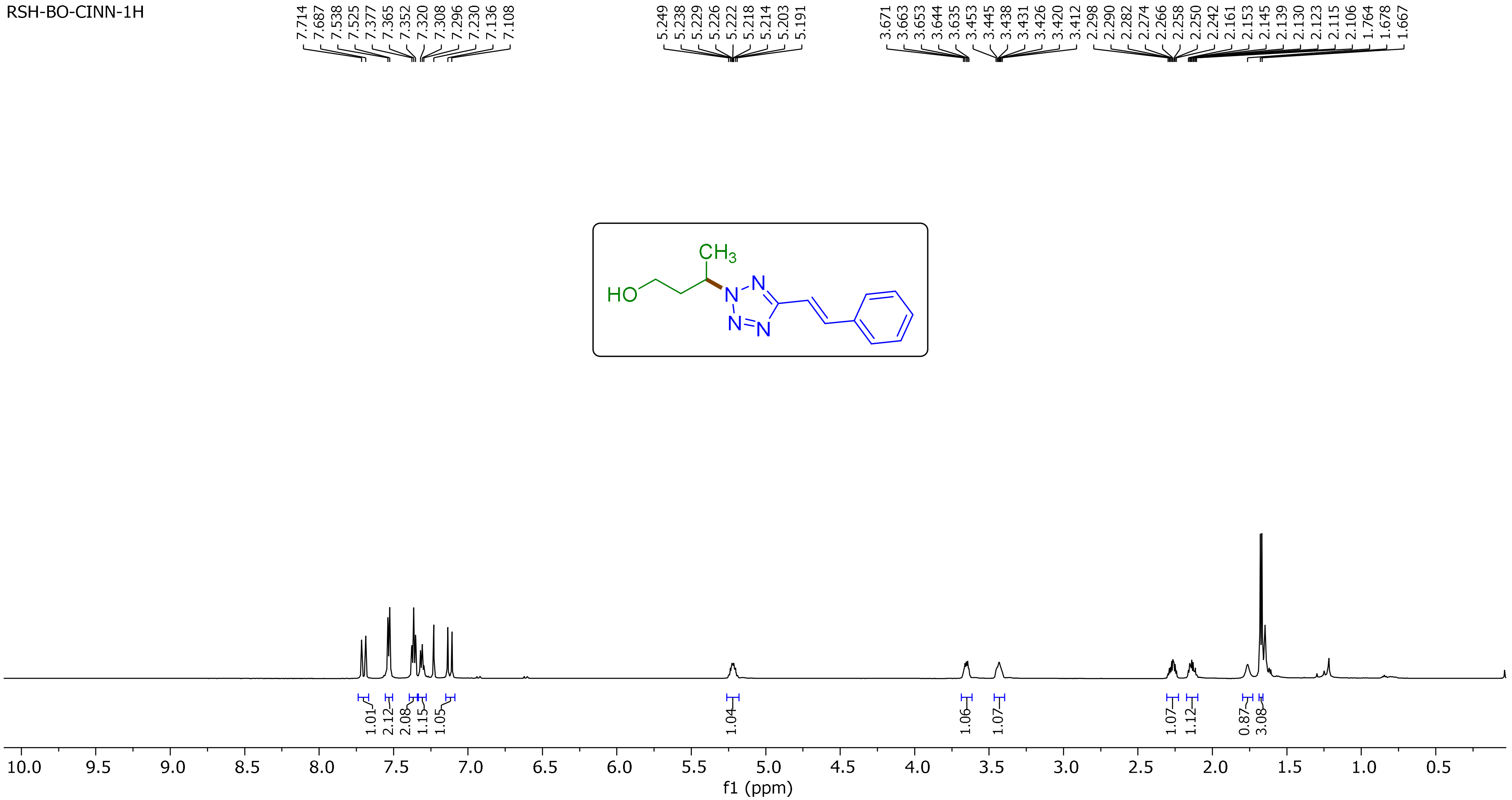

## (*E*)-3-(5-Styryl-2*H*-tetrazol-2-yl)butan-1-ol (35n): $^{13}$C NMR (151 MHz, $CDCl_3$)

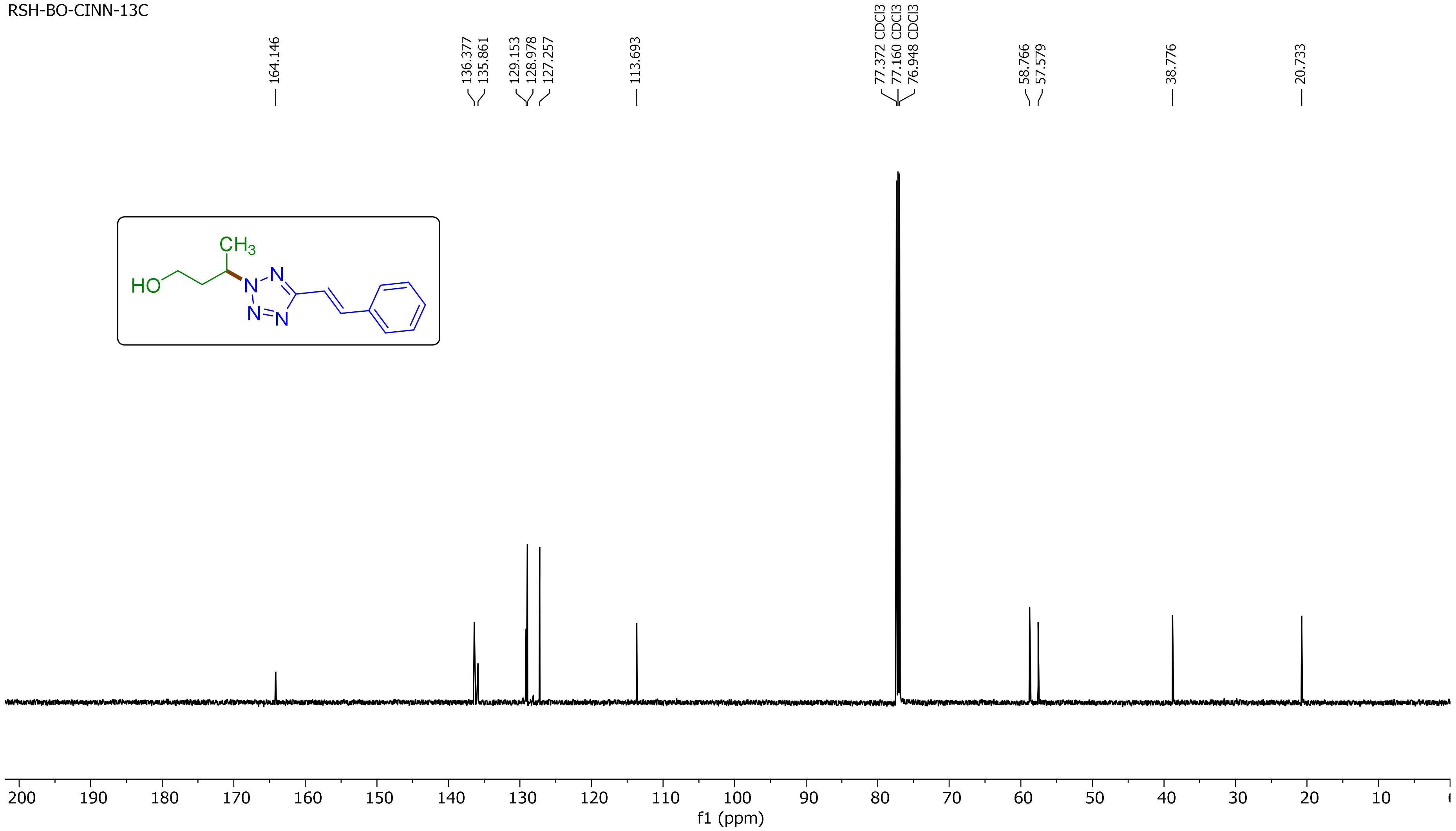

**3-Phenyl-3-(5-phenyl-2*H*-tetrazol-2-yl)propan-1-ol (36a): $^1$H NMR (600 MHz, CDCl$_3$)**

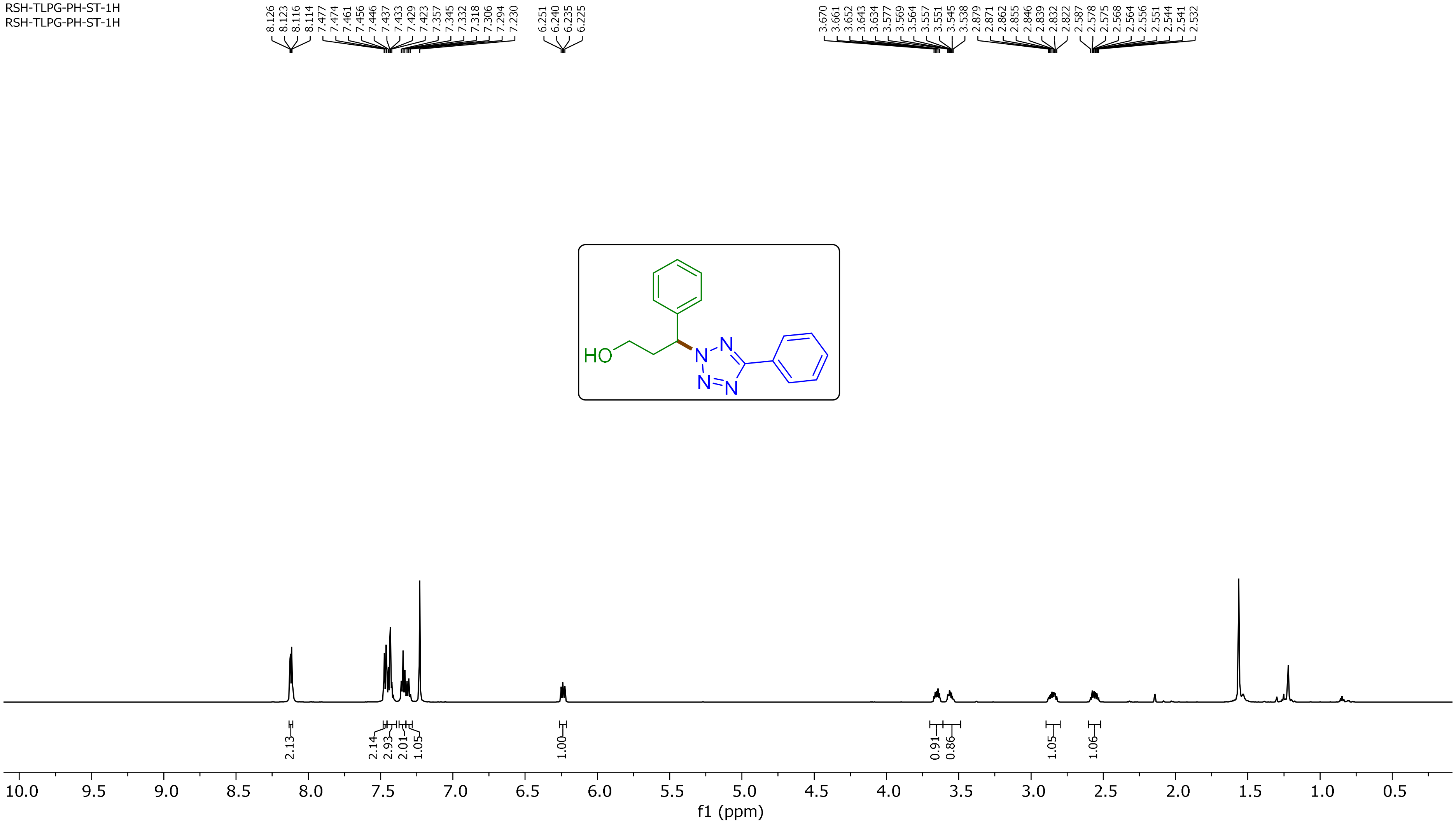

**3-Phenyl-3-(5-phenyl-2*H*-tetrazol-2-yl)propan-1-ol (36a): $^{13}$C NMR (151 MHz, $CDCl_3$)**

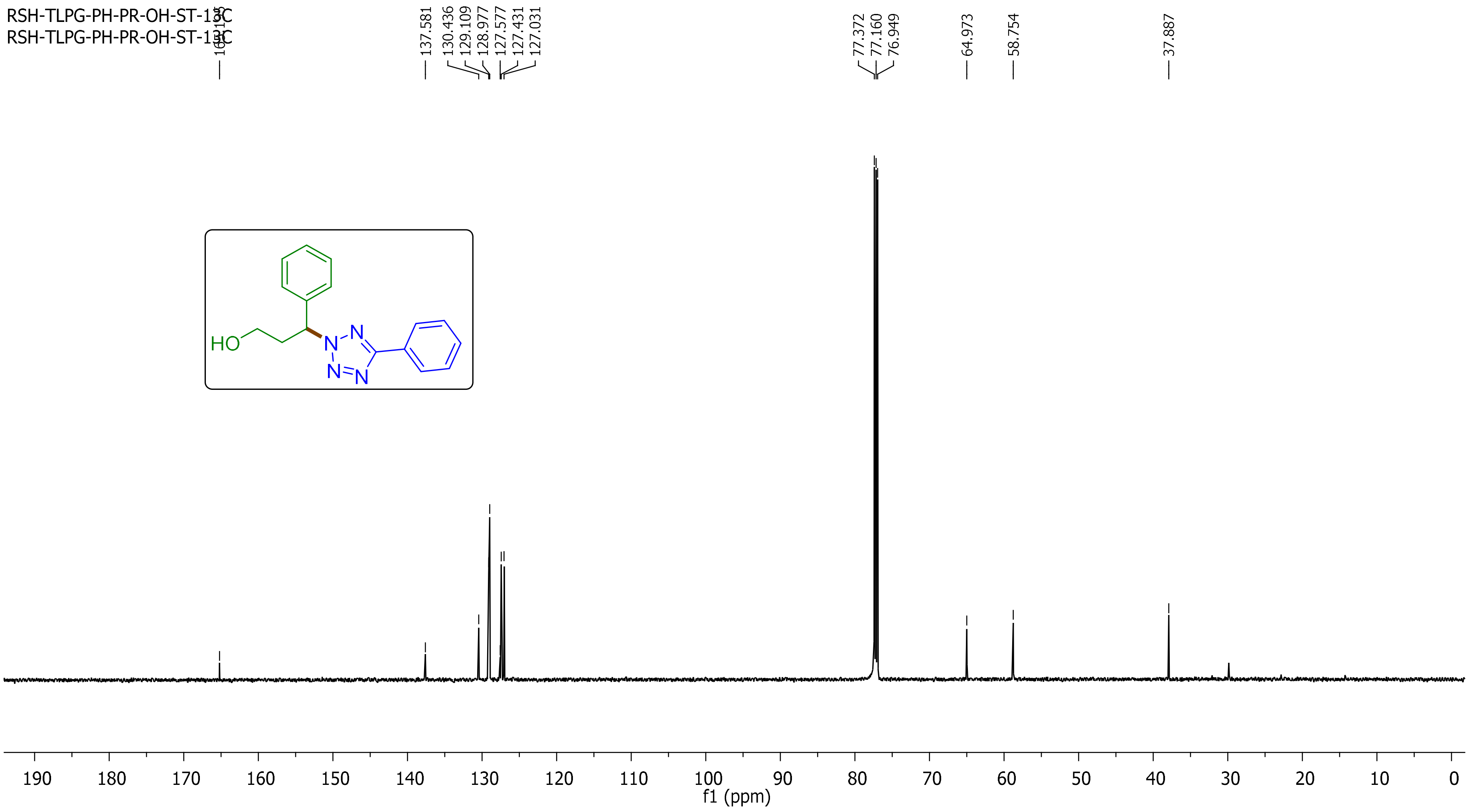

**3-(5-(4-Methoxyphenyl)-2*H*-tetrazol-2-yl)-3-phenylpropan-1-ol (37e): $^{1}$H NMR (400 MHz, CDCl$_3$)**

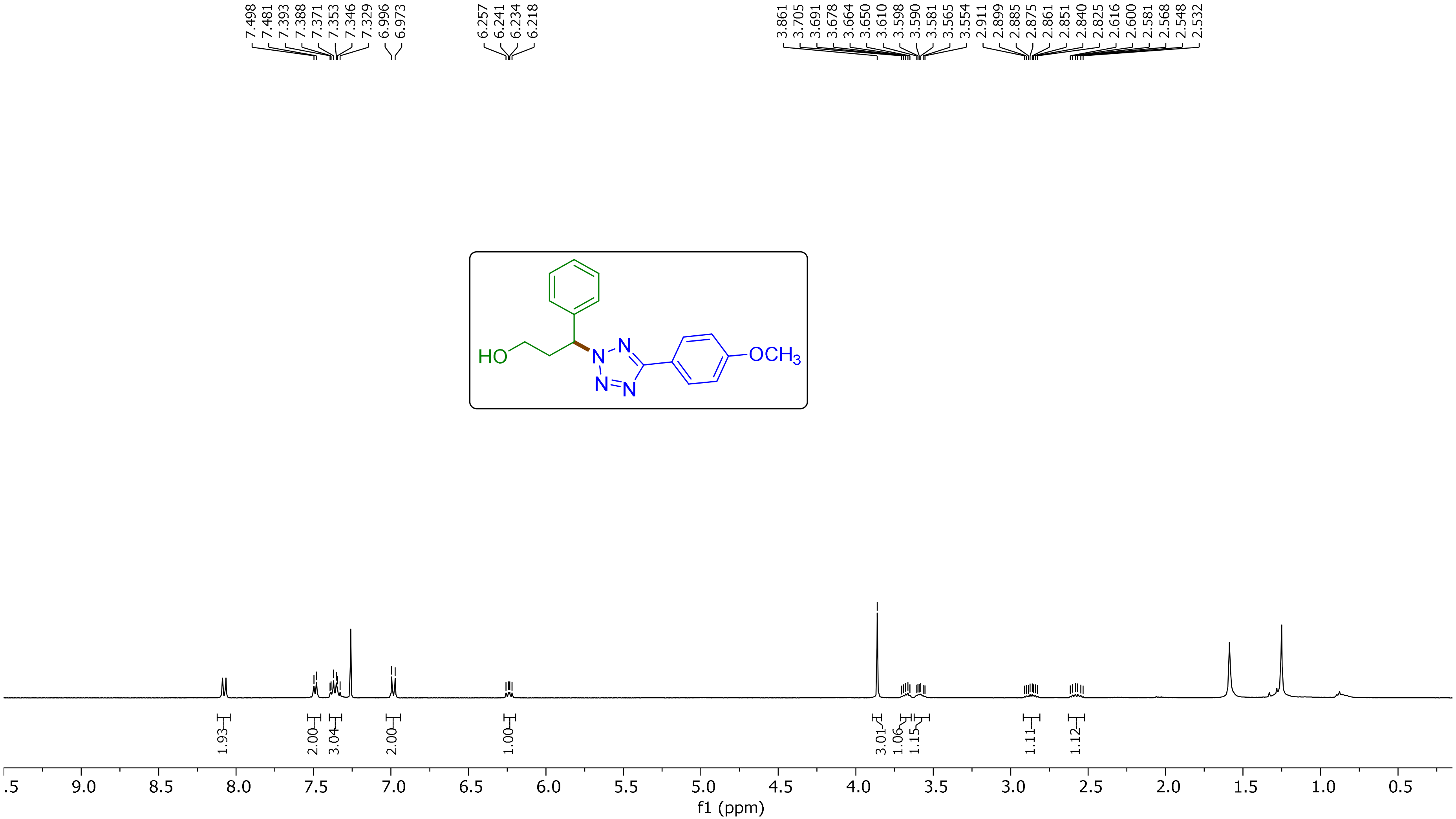

**3-(5-(4-Methoxyphenyl)-2*H*-tetrazol-2-yl)-3-phenylpropan-1-ol (37e): $^{13}$C NMR (151 MHz, CDCl$_3$)**

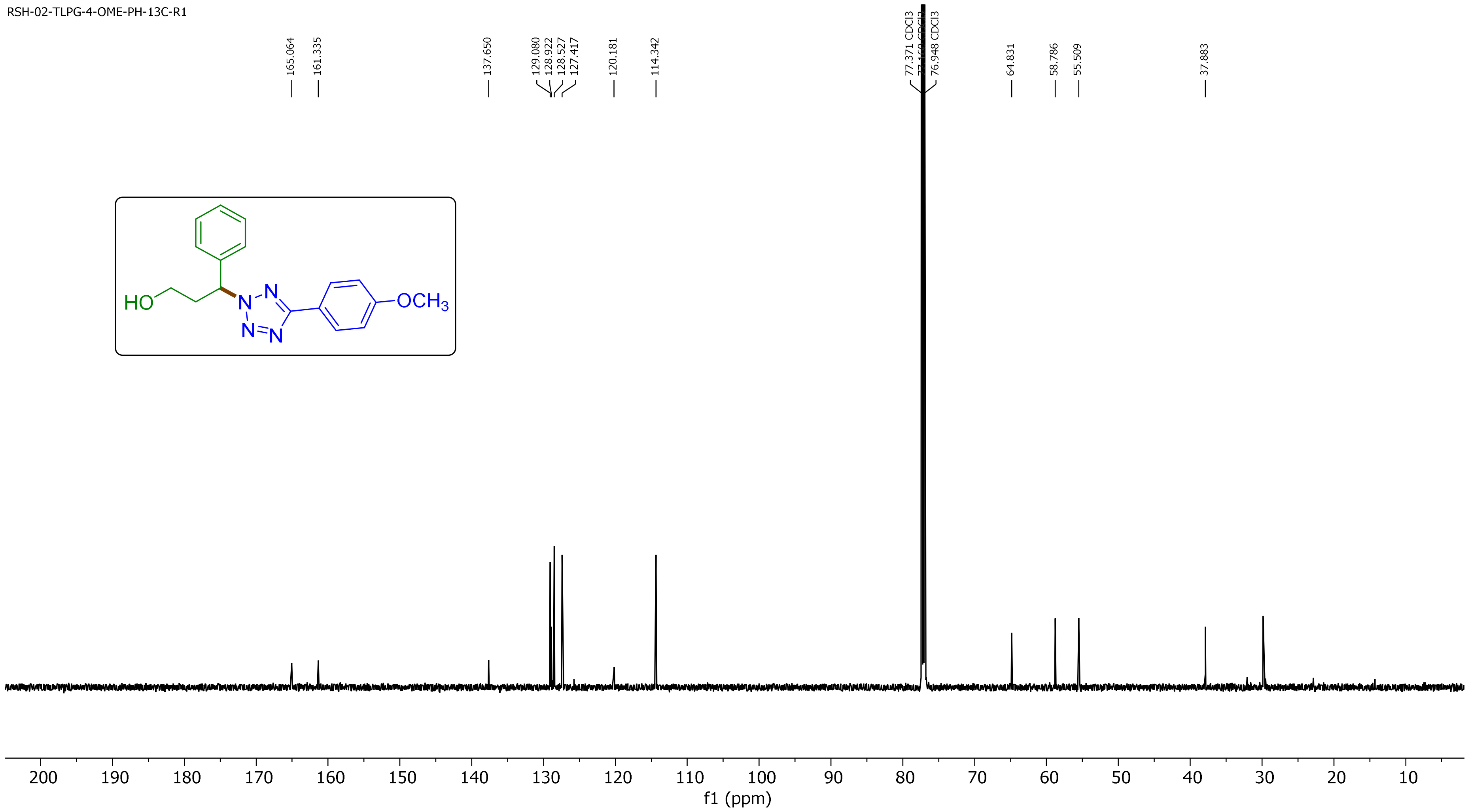

**4-Phenyl-4-(5-phenyl-2*H*-tetrazol-2-yl)butan-1-ol (38a): $^{1}$H NMR (600 MHz, CDCl$_3$)**

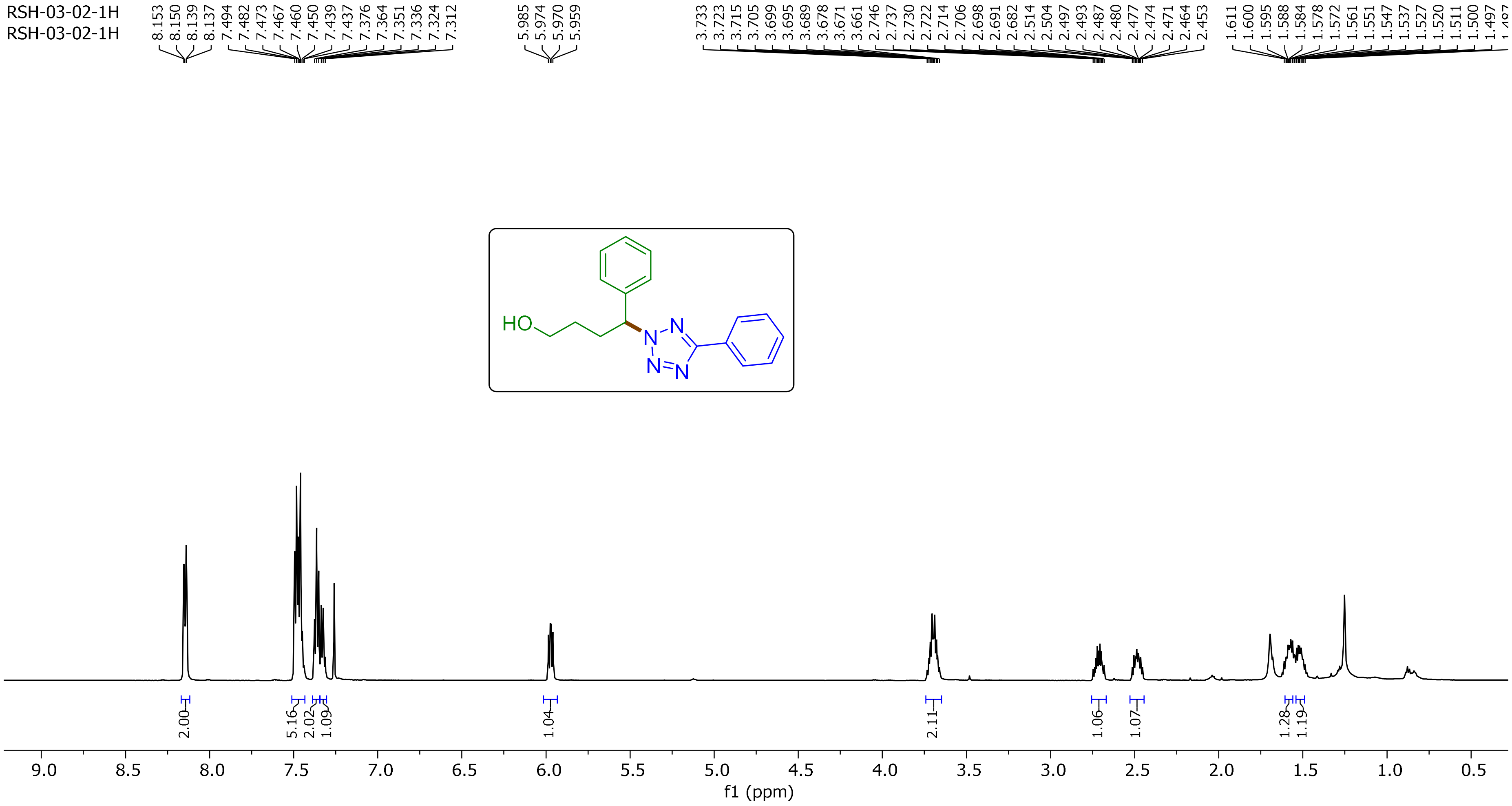

**4-Phenyl-4-(5-phenyl-2*H*-tetrazol-2-yl)butan-1-ol (38a): $^{13}$C NMR (151 MHz, CDCl$_3$)**

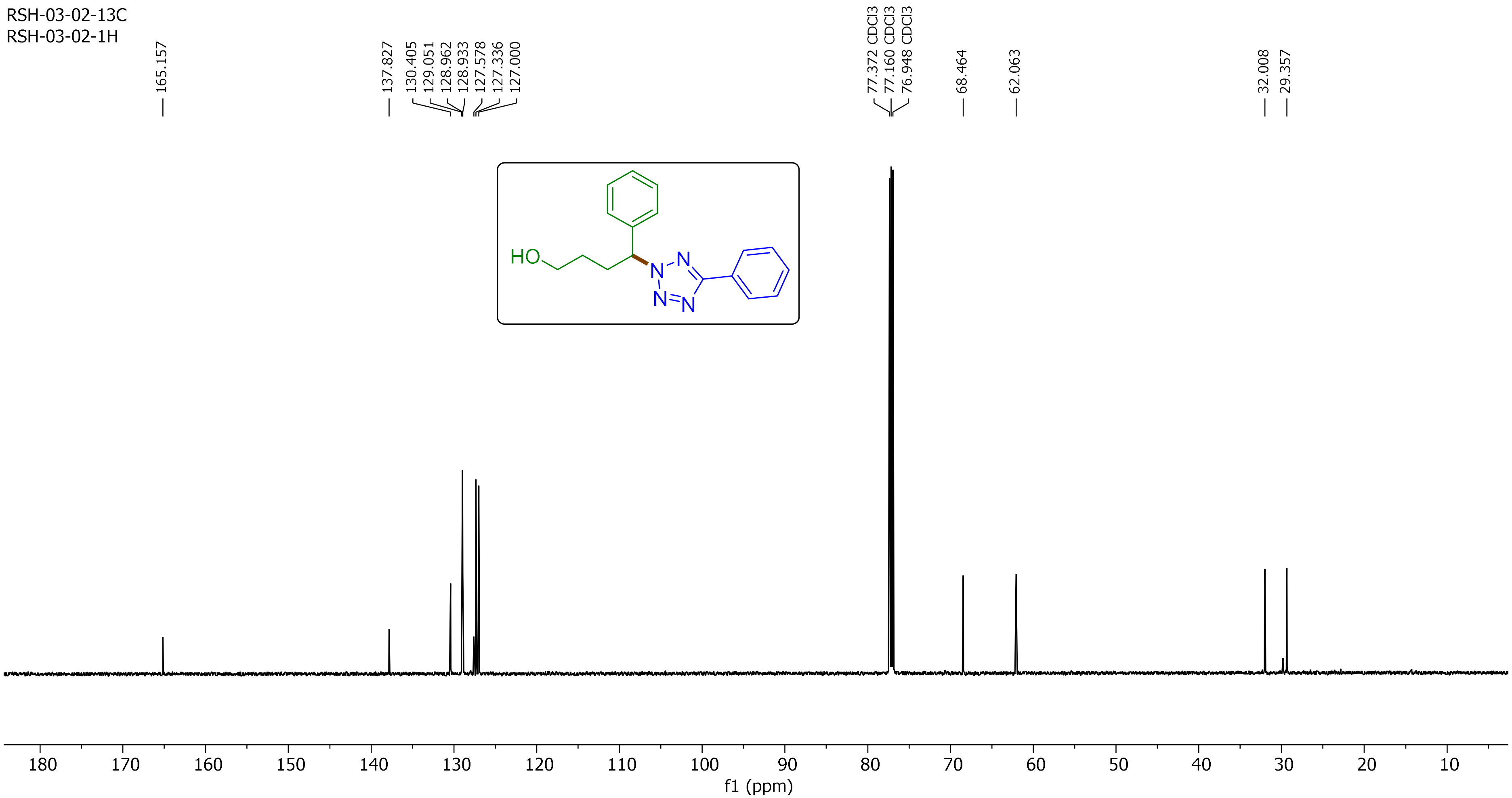

**3-(5-Phenyl-2*H*-tetrazol-2-yl)hexan-1-ol (38a): $^1$H NMR (600 MHz, $CDCl_3$)**

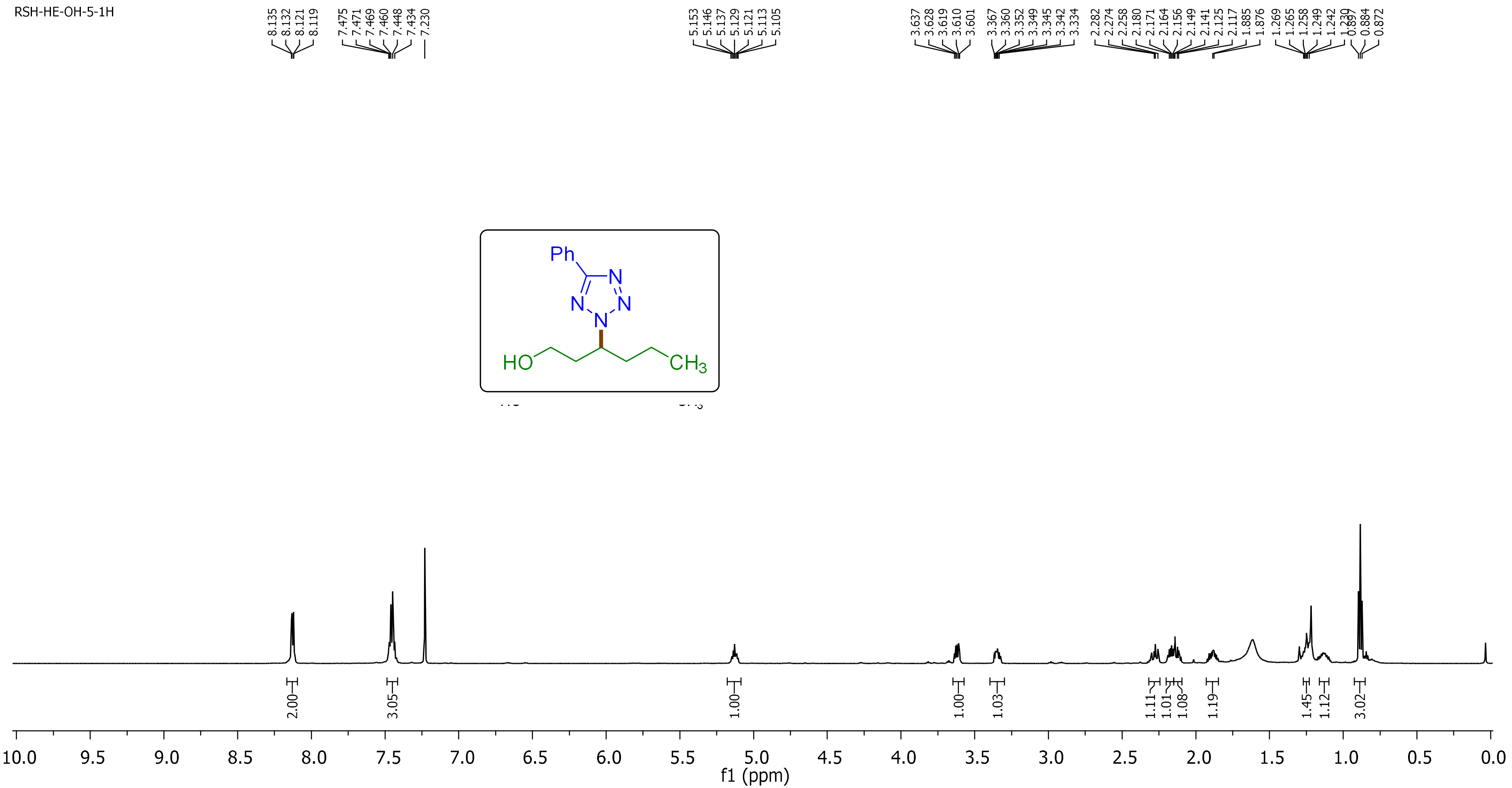

**3-(5-Phenyl-2*H*-tetrazol-2-yl)hexan-1-ol (38a): $^{13}$C NMR (151 MHz, $CDCl_3$)**

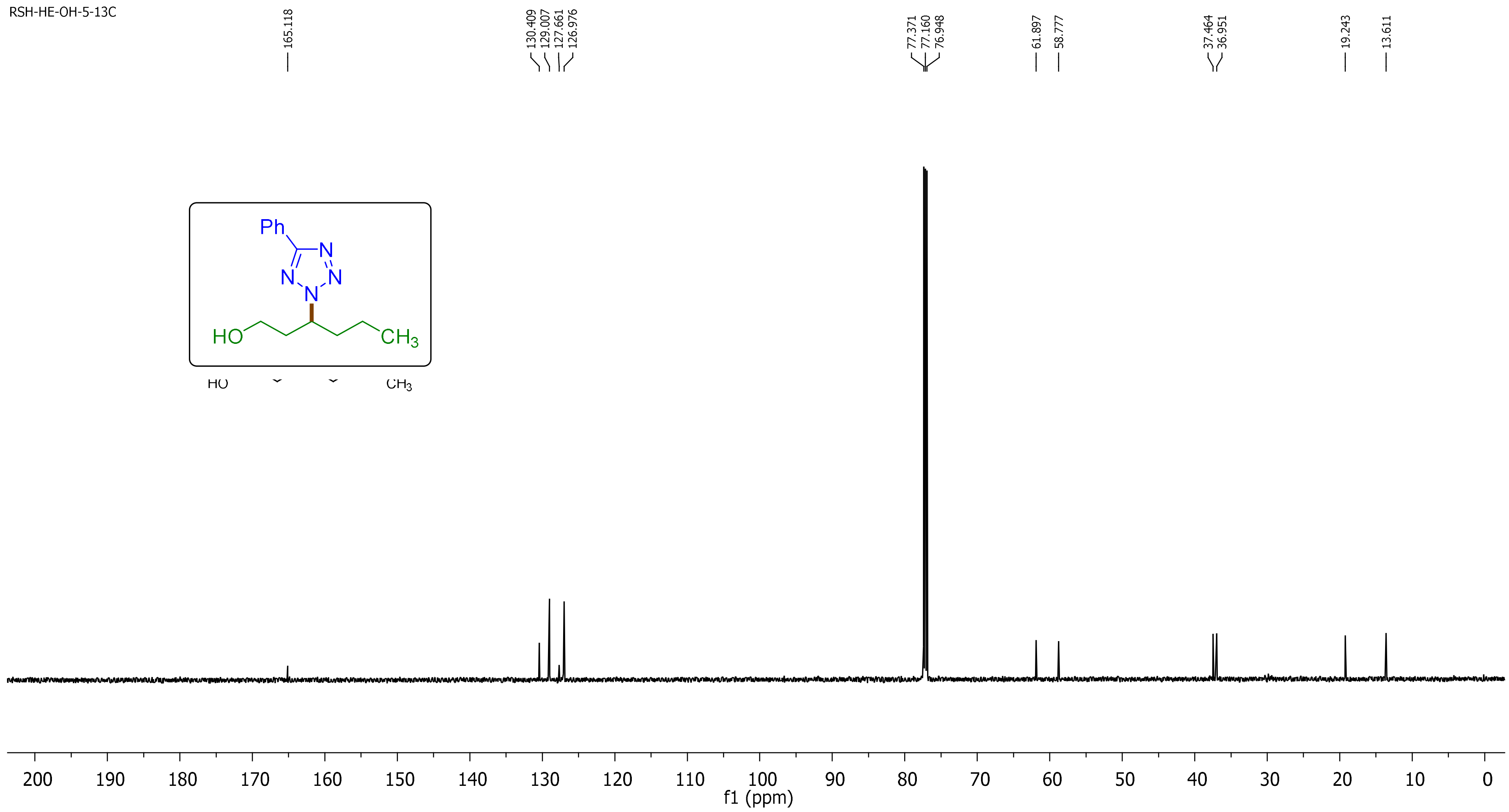

**3-(5-Phenyl-2*H*-tetrazol-2-yl)hexan-1-ol (38a): $^{13}C$ {$^{1}H$} DEPT-135 NMR (151 MHz, $CDCl_3$)**

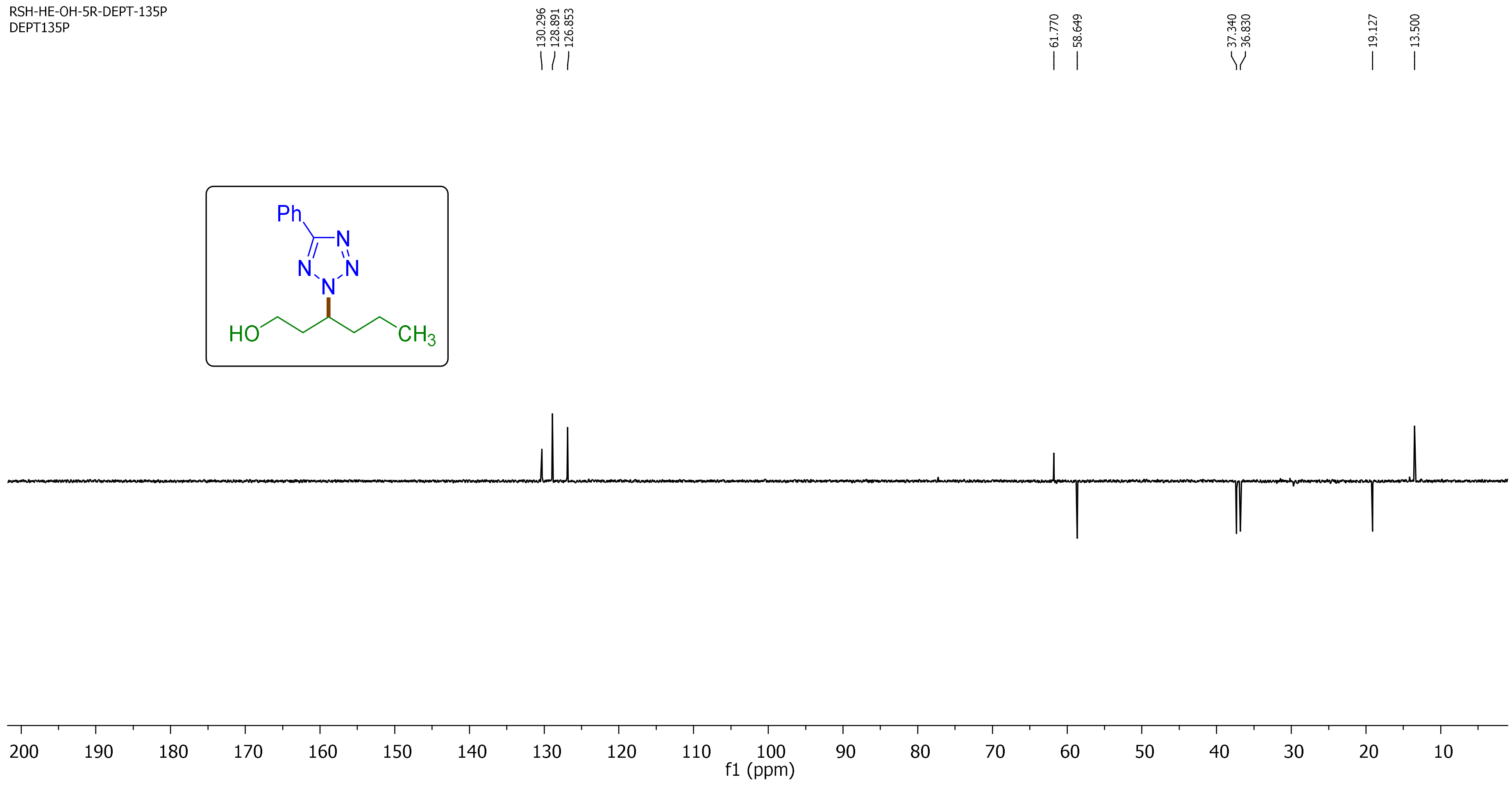

**3-(5-Phenyl-2*H*-tetrazol-2-yl)hexan-1-ol (38a): HSQC NMR**

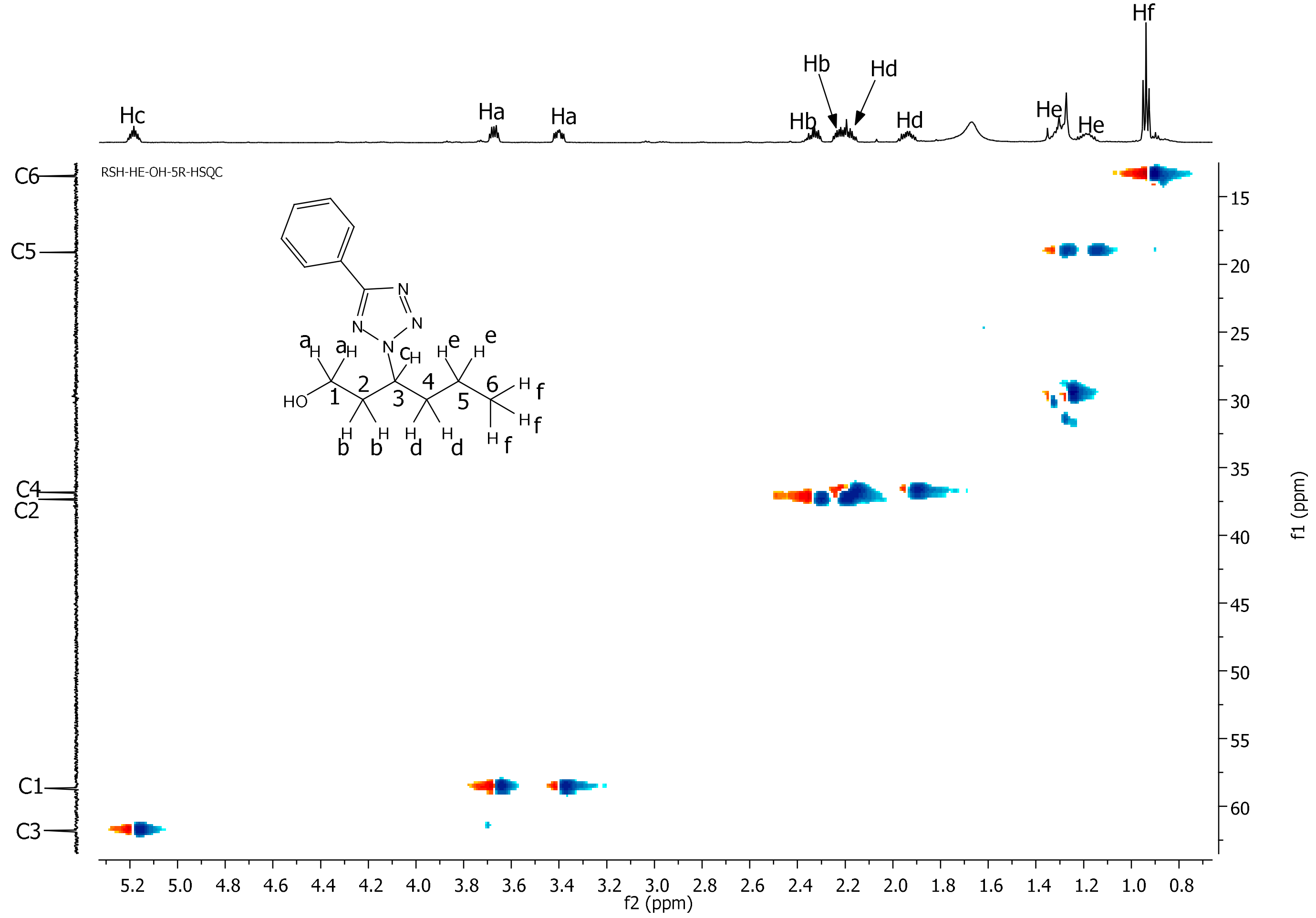

**4-(5-Phenyl-2*H*-tetrazol-2-yl)hexan-1-ol (38'a): $^{1}$H NMR (600 MHz, $CDCl_3$)**

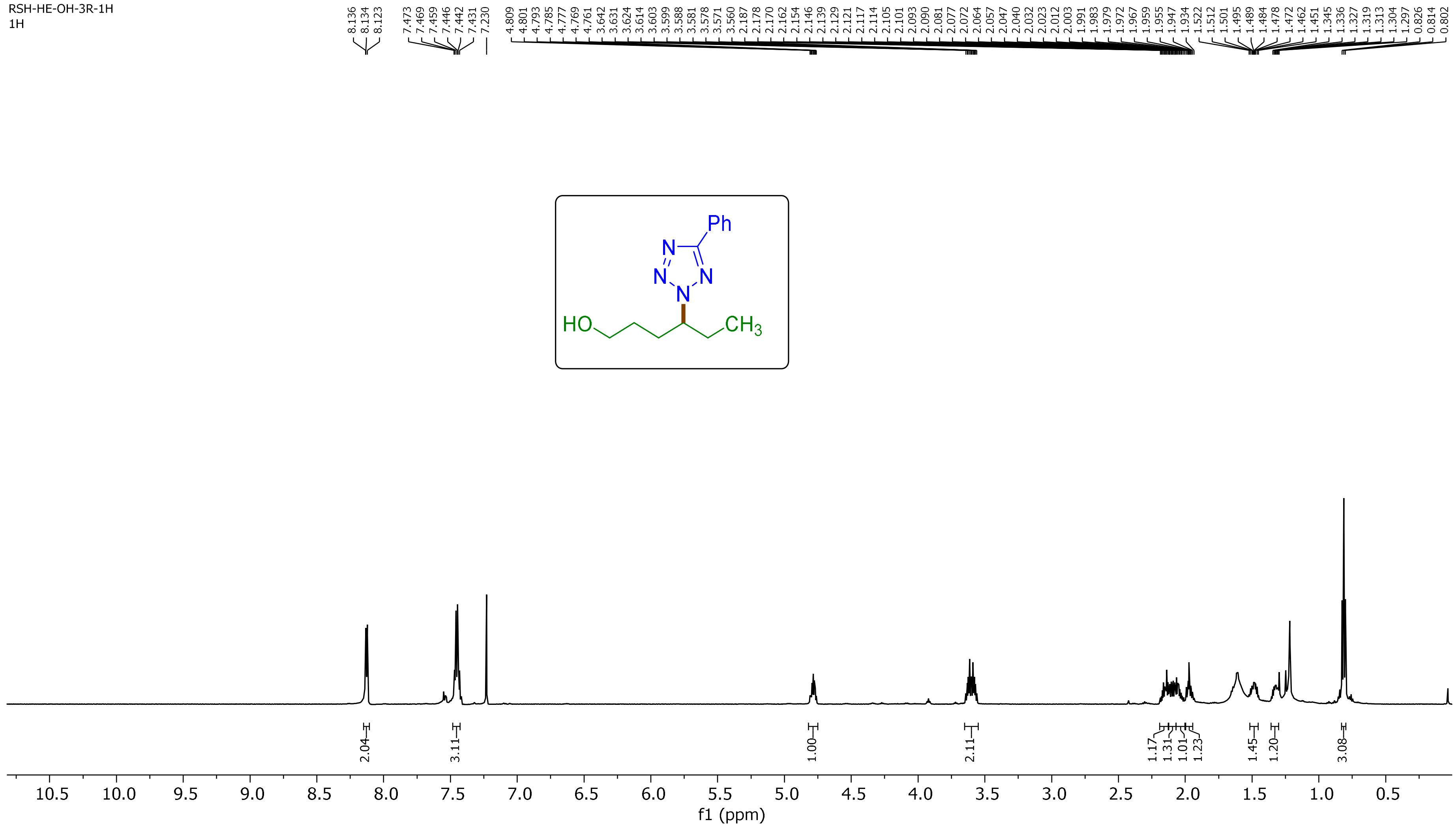

**4-(5-Phenyl-2*H*-tetrazol-2-yl)hexan-1-ol (38'a): $^{13}$C NMR (151 MHz, CDCl$_3$)**

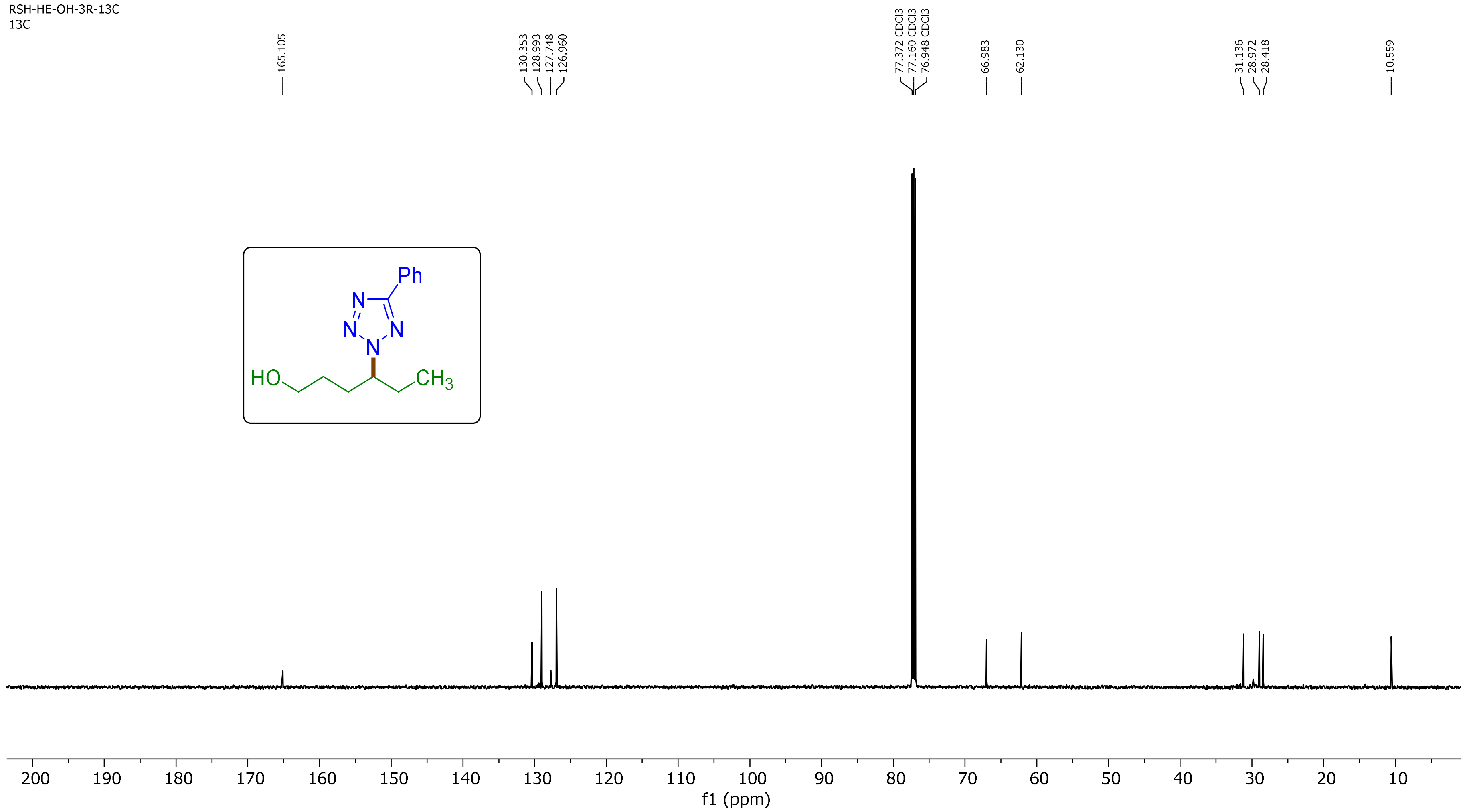

**4-(5-Phenyl-2*H*-tetrazol-2-yl)hexan-1-ol (38'a): $^{13}C\ \{^1H\}$ DEPT-135 NMR (151 MHz, $CDCl_3$)**

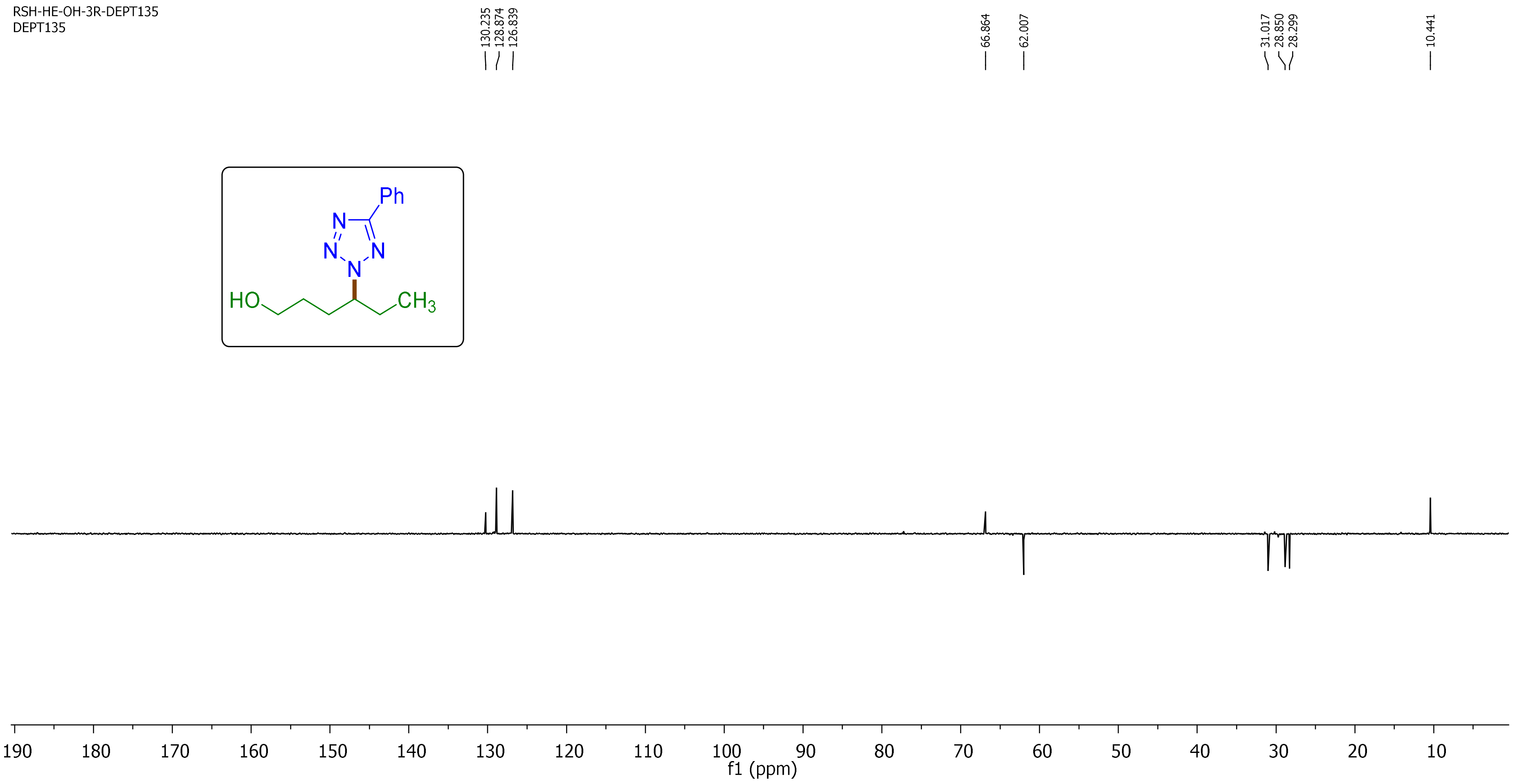

**4-(5-Phenyl-2*H*-tetrazol-2-yl)hexan-1-ol (38'a): HSQC NMR**

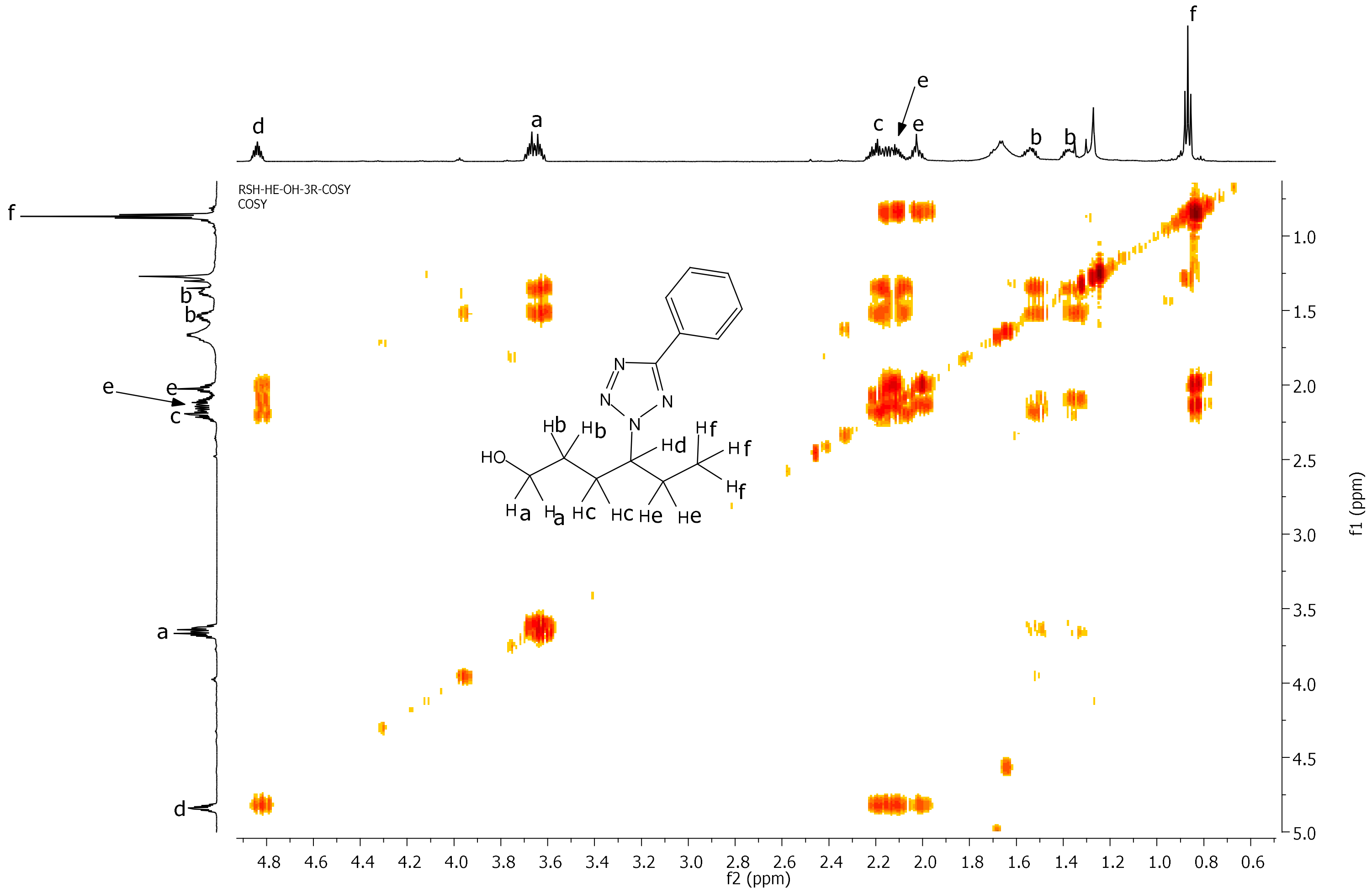

**5-(5-Phenyl-2*H*-tetrazol-2-yl)hexan-1-ol (38''a): $^{1}$H NMR (600 MHz, CDCl₃)**

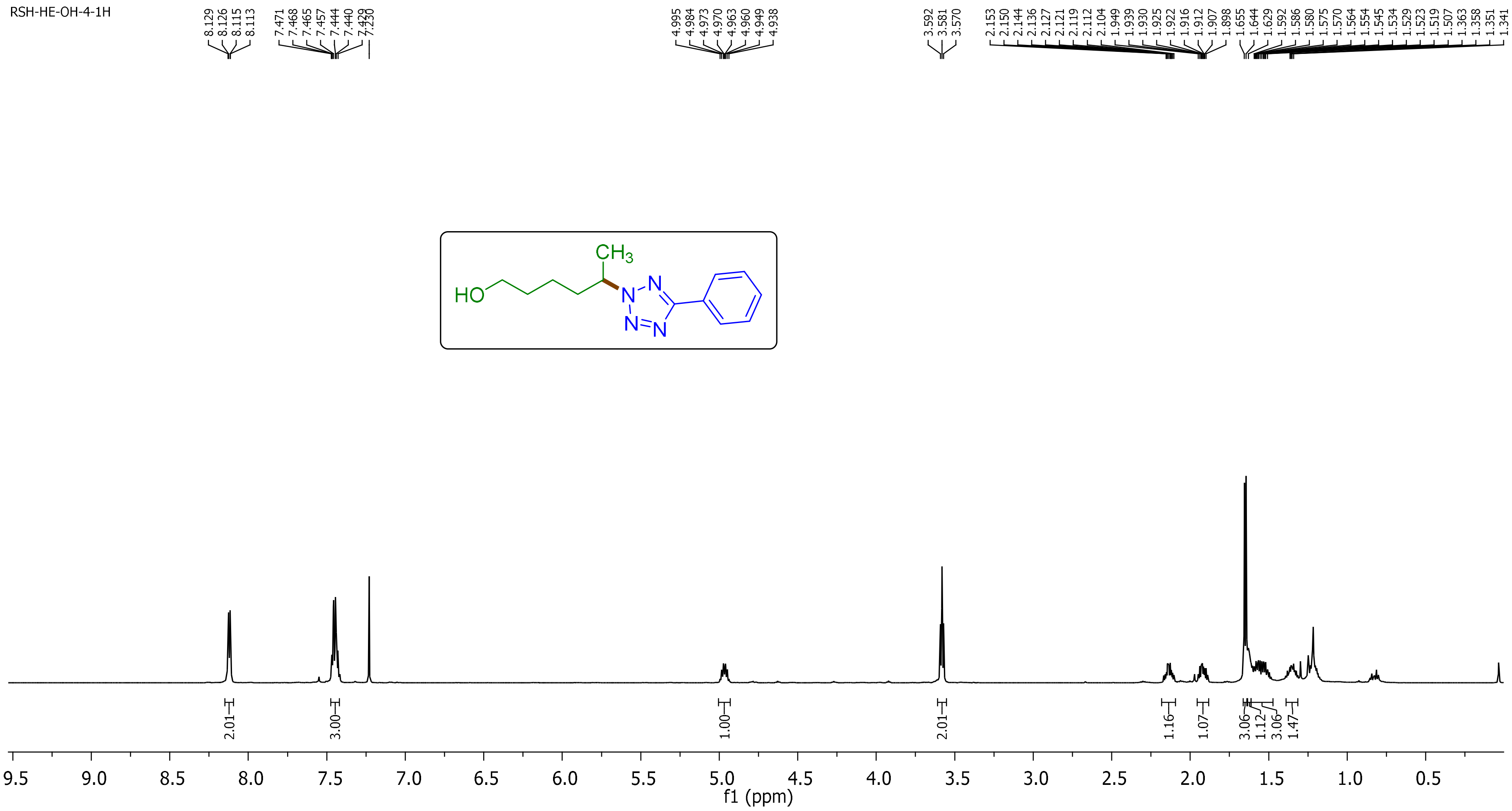

**5-(5-Phenyl-2*H*-tetrazol-2-yl)hexan-1-ol (38''a): $^{13}$C NMR (151 MHz, CDCl$_3$)**

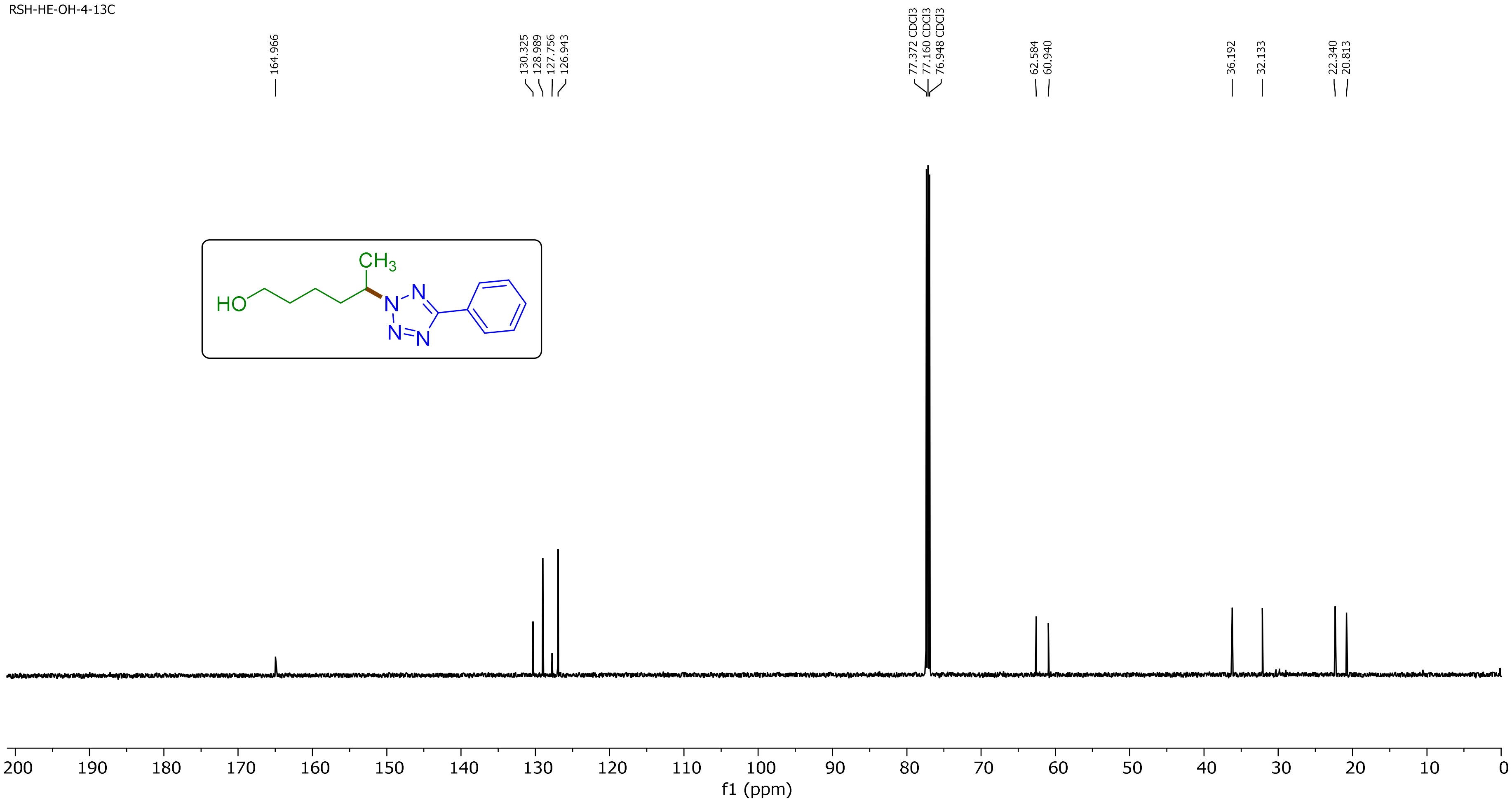

**3-(4-(4-(*tert*-Butyl)phenyl)-2*H*-1,2,3-triazol-2-yl)butan-1-ol (35s): $^{1}$H NMR (600 MHz, CDCl$_3$)**

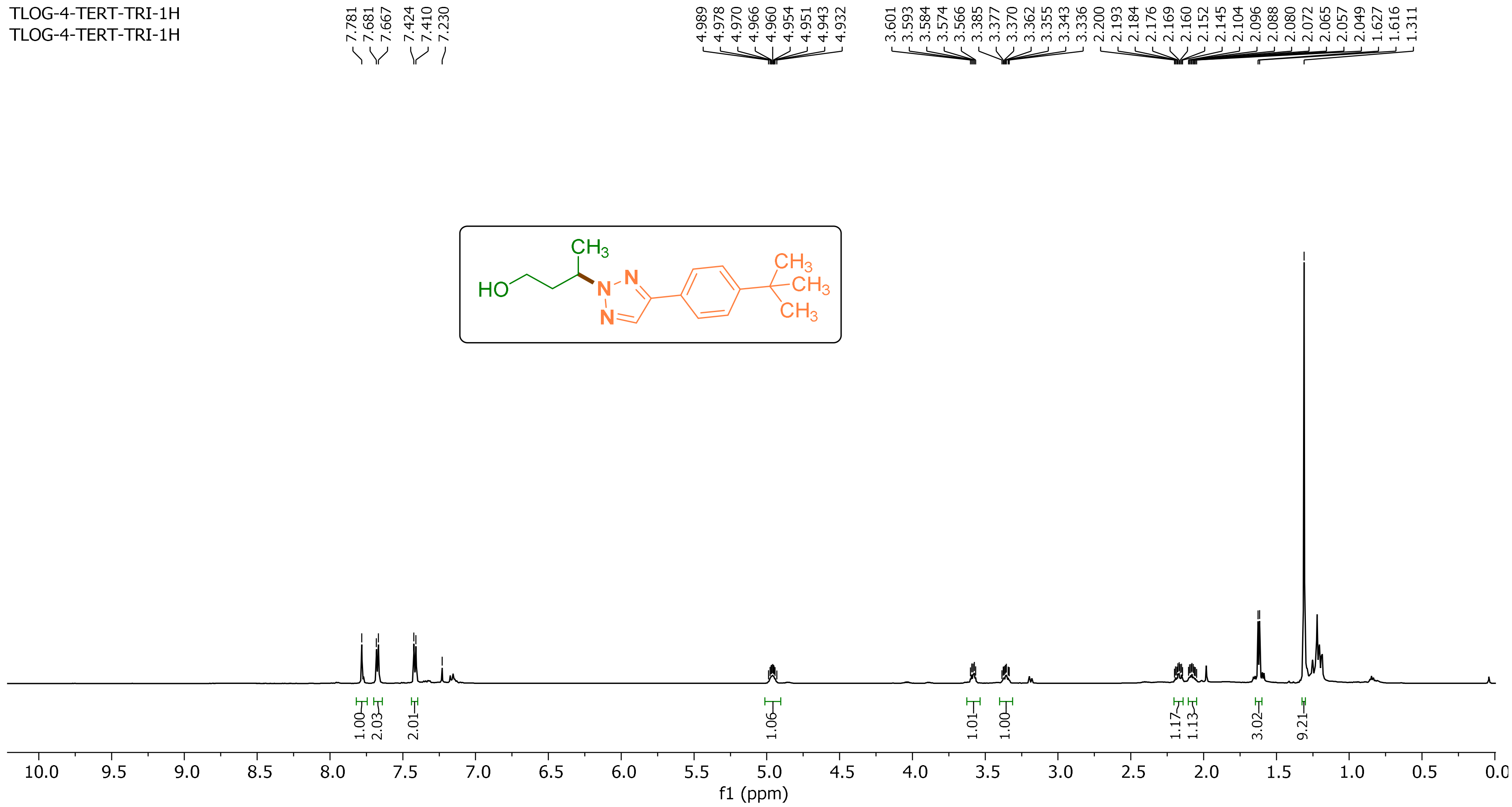

**3-(4-(4-(*tert*-Butyl)phenyl)-2*H*-1,2,3-triazol-2-yl)butan-1-ol (35s): $^{13}$C NMR (151 MHz, CDCl$_3$)**

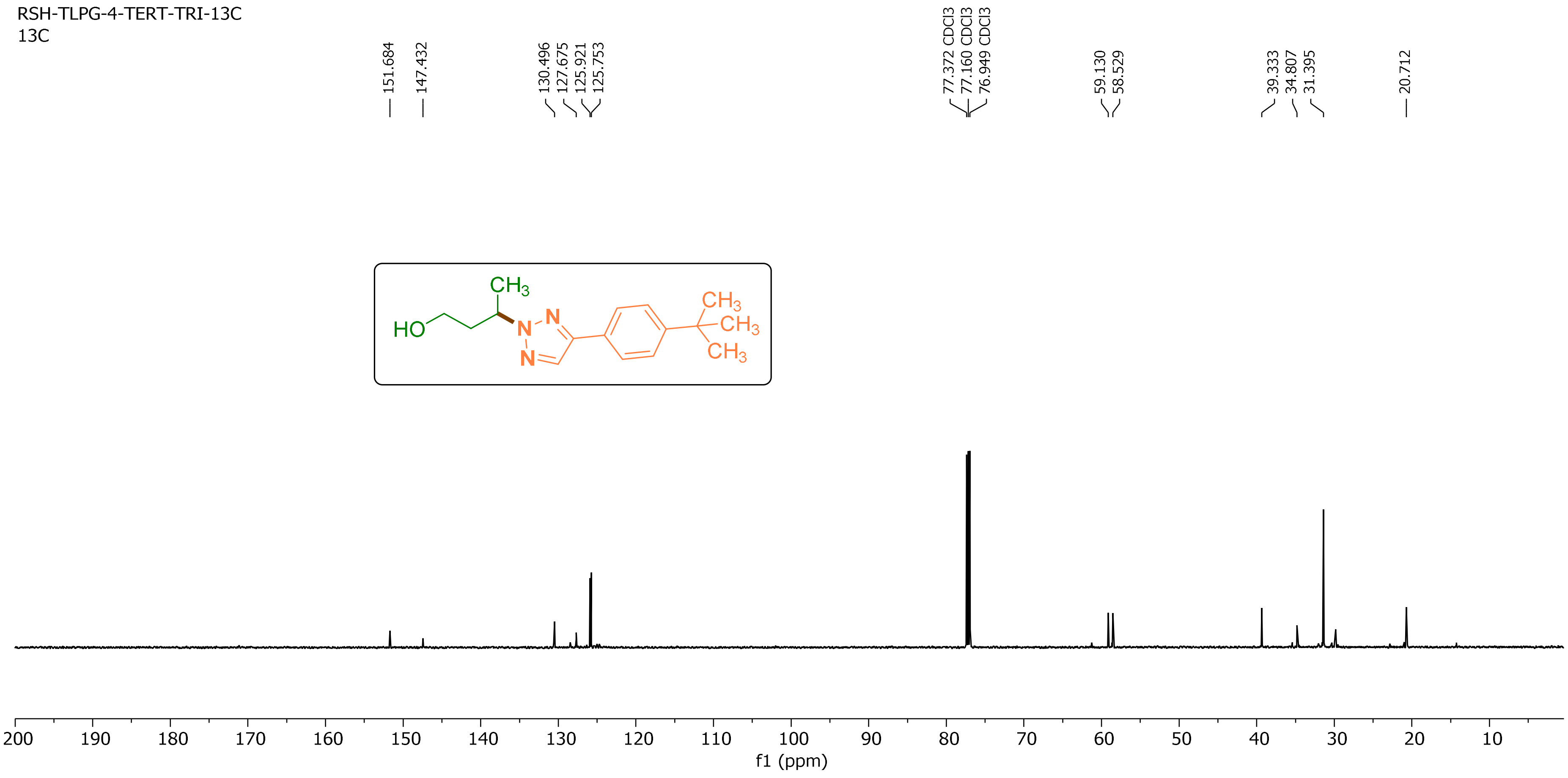

**3-(4-(4-Chlorophenyl)-2*H*-1,2,3-triazol-2-yl)butan-1-ol (35t): $^{1}$H NMR (600 MHz, CDCl₃)**

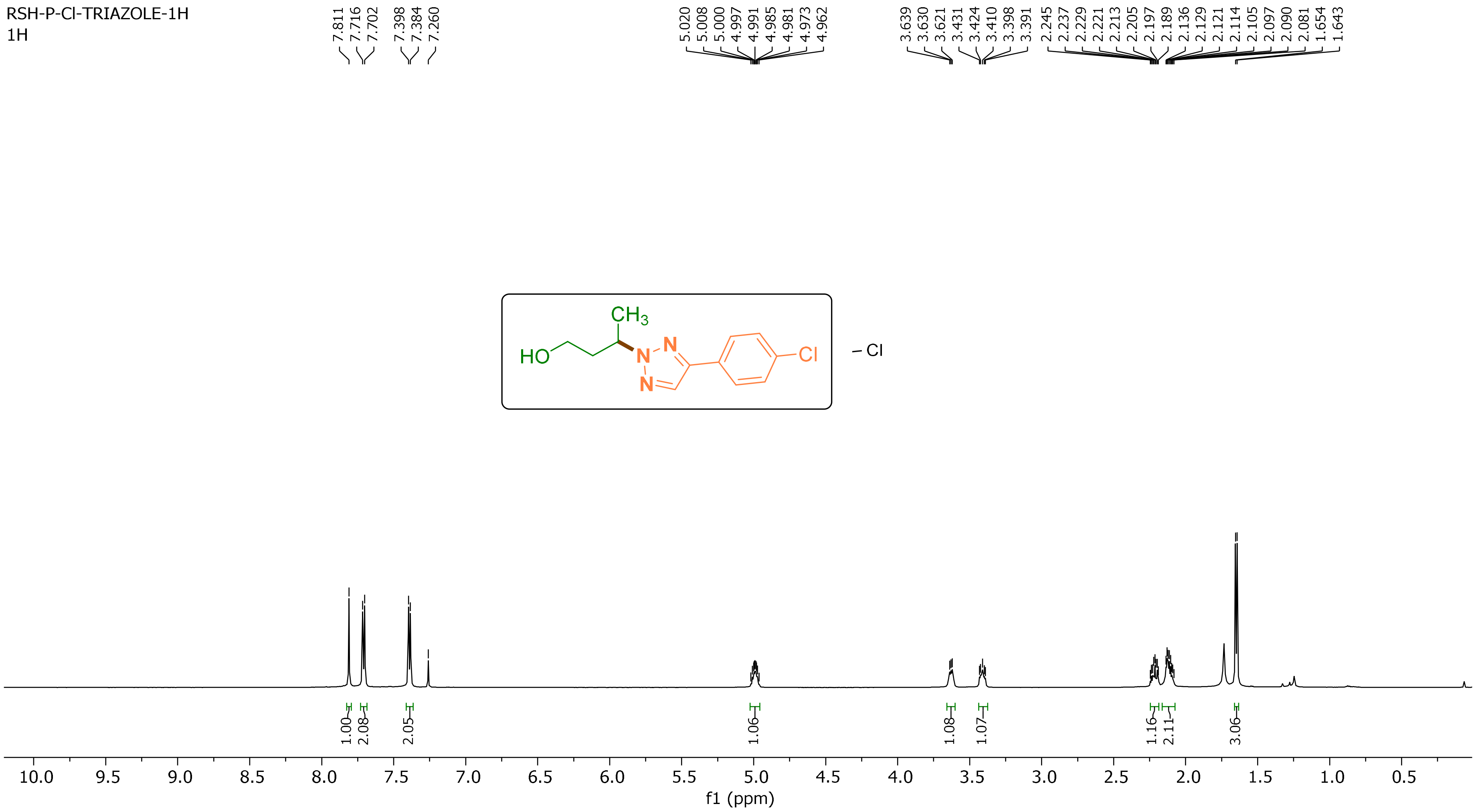

**3-(4-(4-Chlorophenyl)-2*H*-1,2,3-triazol-2-yl)butan-1-ol (35t): $^{13}$C NMR (151 MHz, $CDCl_3$)**

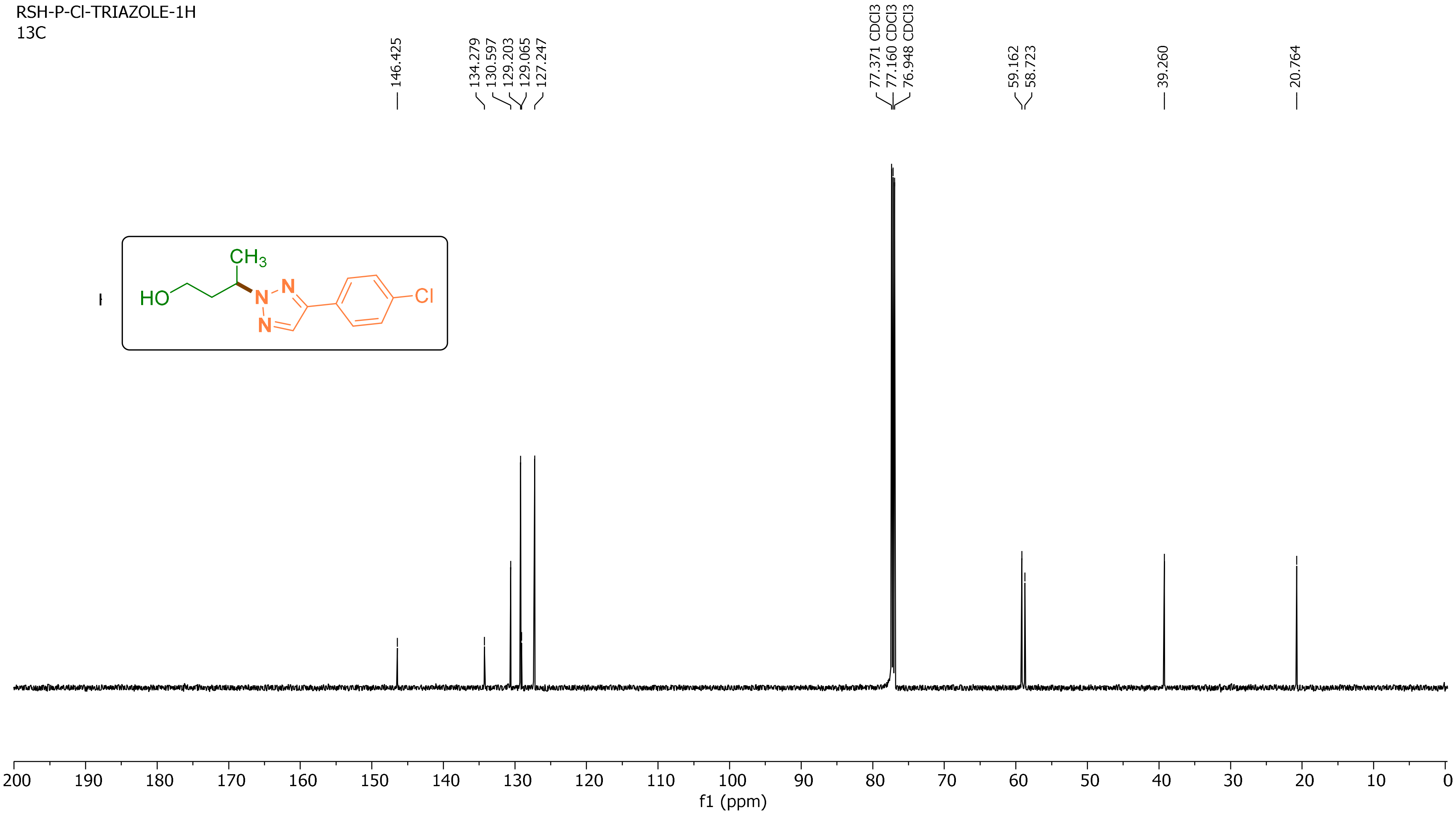

**2-(Octan-4-yl)-5-phenyl-2*H*-tetrazole (39a-C4) + 2-(Octan-3-yl)-5-phenyl-2*H*-tetrazole (39a-C3) + 2-(Octan-2-yl)-5-phenyl-2*H*-tetrazole (39a-C2) + 2-Octyl-5-phenyl-2H-tetrazole (39a-C1): $^1$H NMR (400 MHz, $CDCl_3$)**

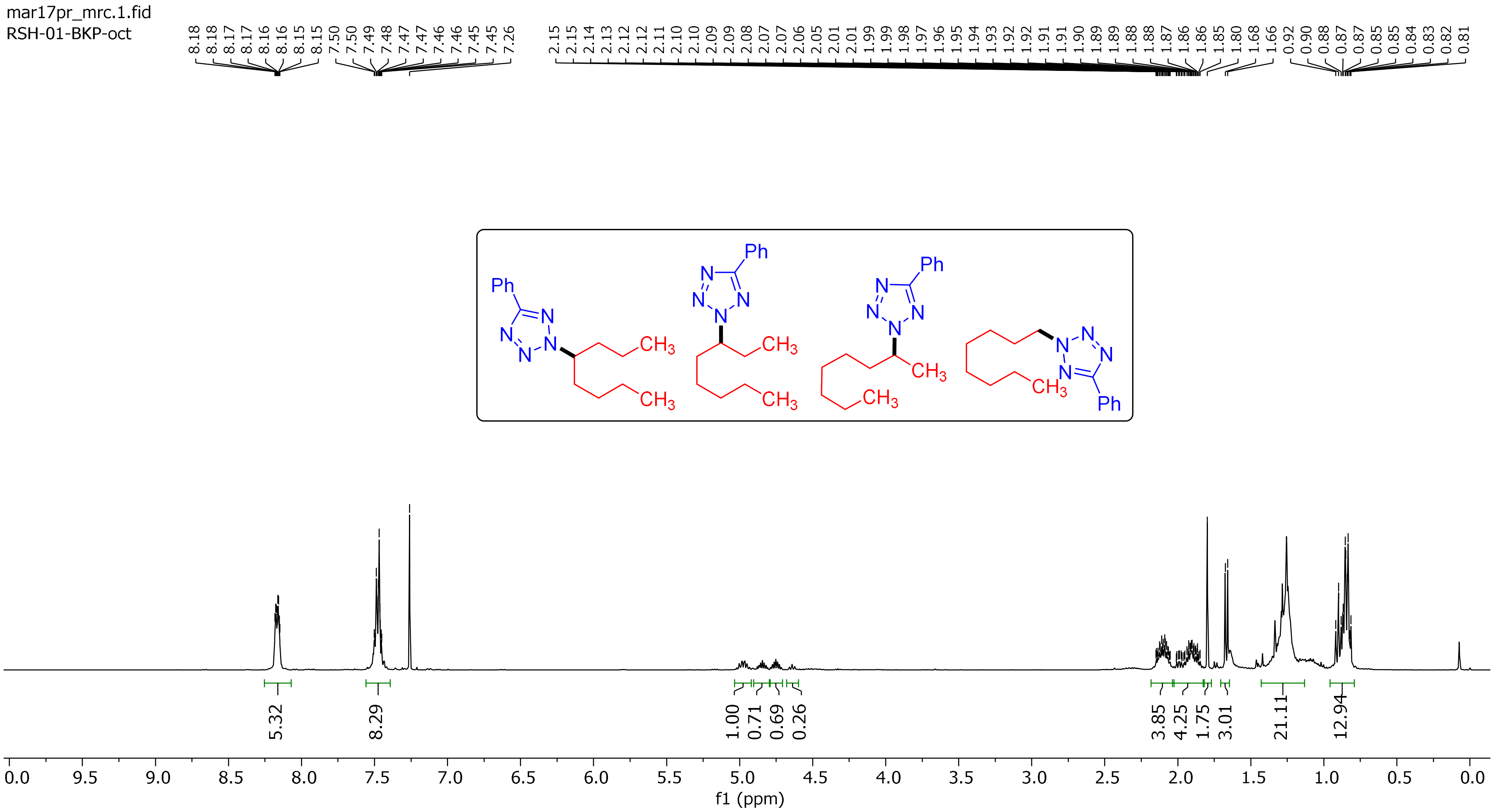

**2-Cyclohexyl-5-phenyl-2*H*-tetrazole (40a): $^1$H NMR (600 MHz, $CDCl_3$)**

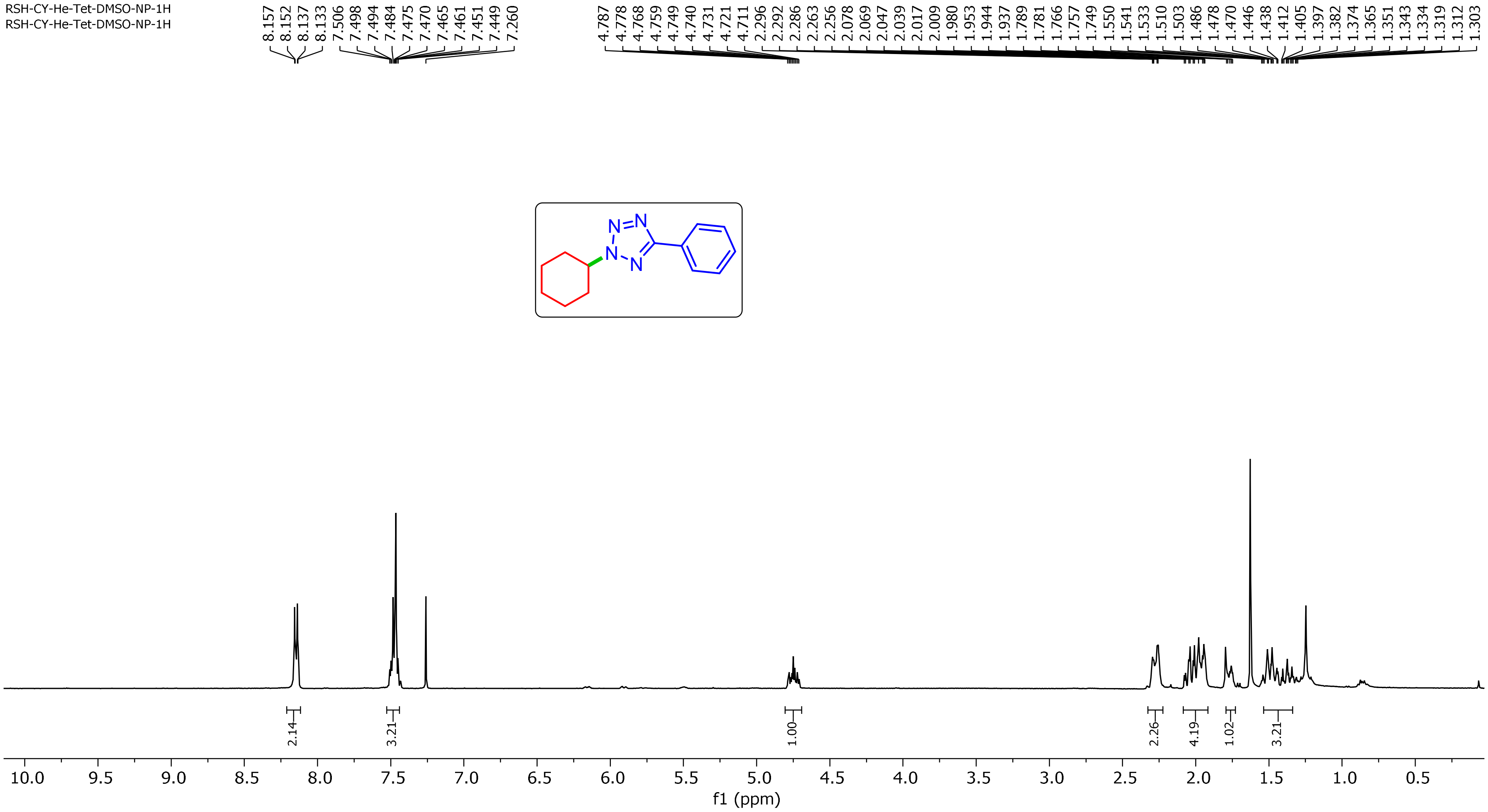

**2-Cyclohexyl-5-phenyl-2*H*-tetrazole (40a): $^{13}$C NMR (151 MHz, $CDCl_3$)**

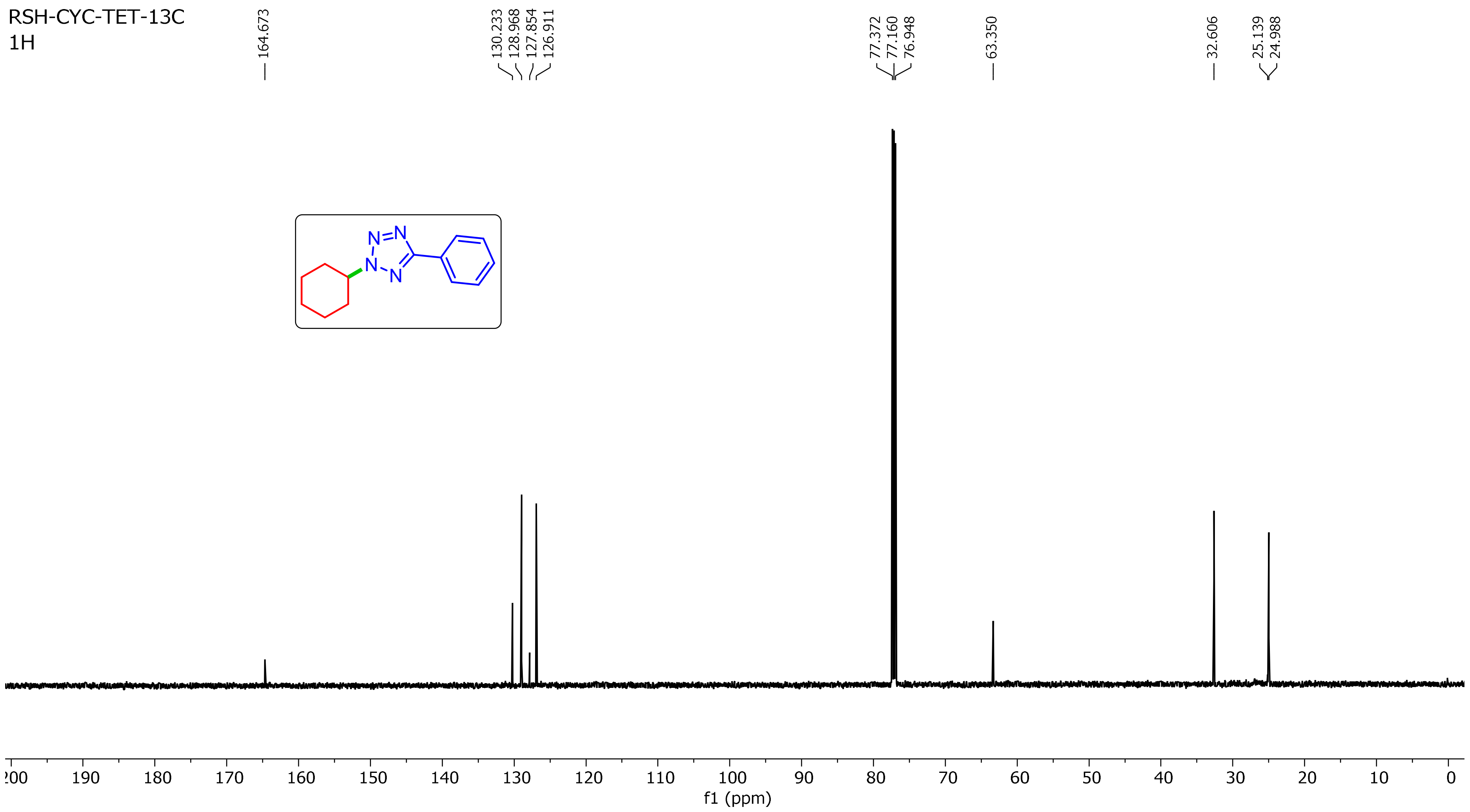

## 5-(4-Bromophenyl)-2-cyclohexyl-2*H*-tetrazole (40i): $^{1}$H NMR (600 MHz, $CDCl_3$)

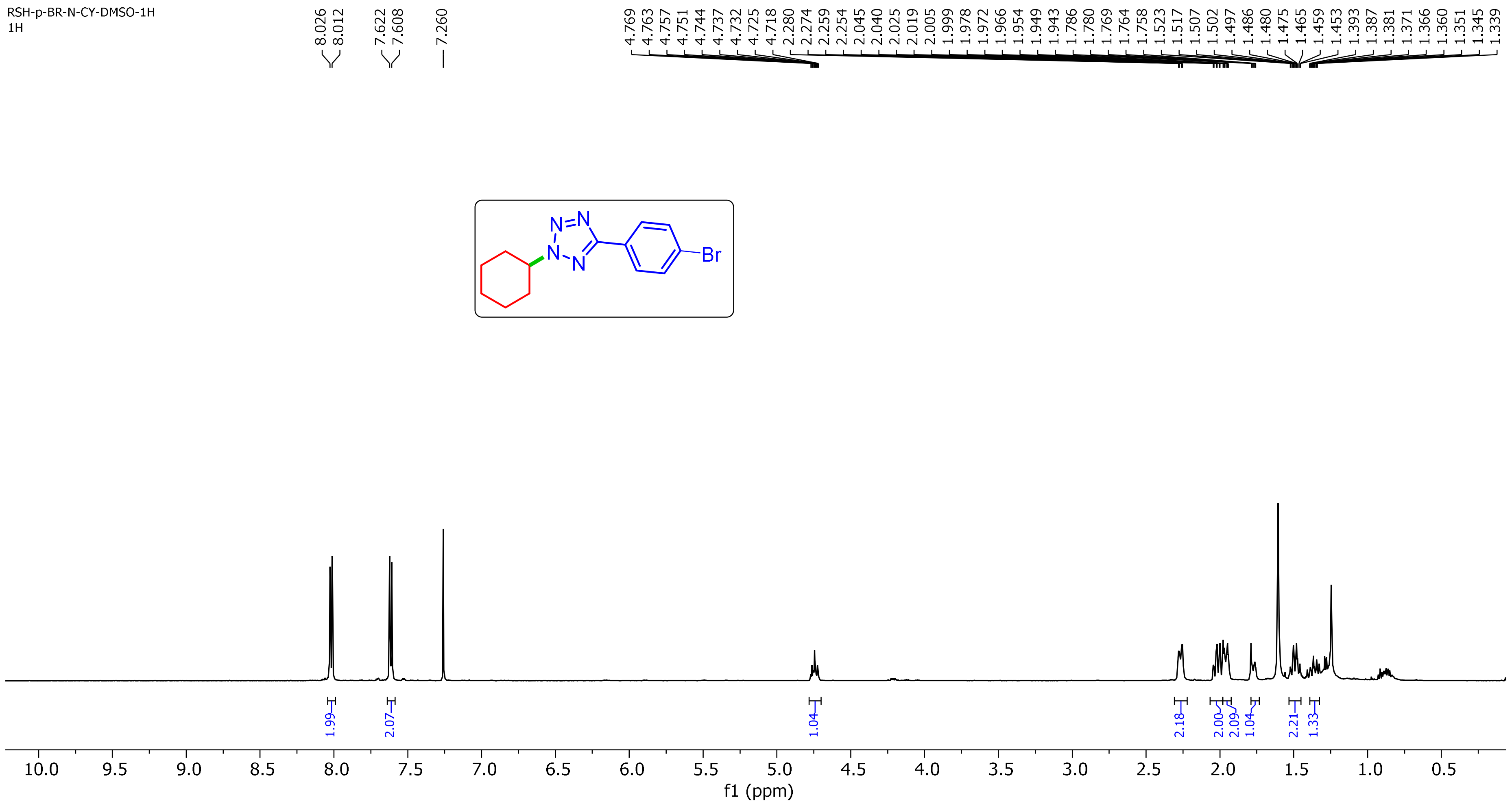

**5-(4-Bromophenyl)-2-cyclohexyl-2*H*-tetrazole (40i): $^{13}$C NMR (151 MHz, $CDCl_3$)**

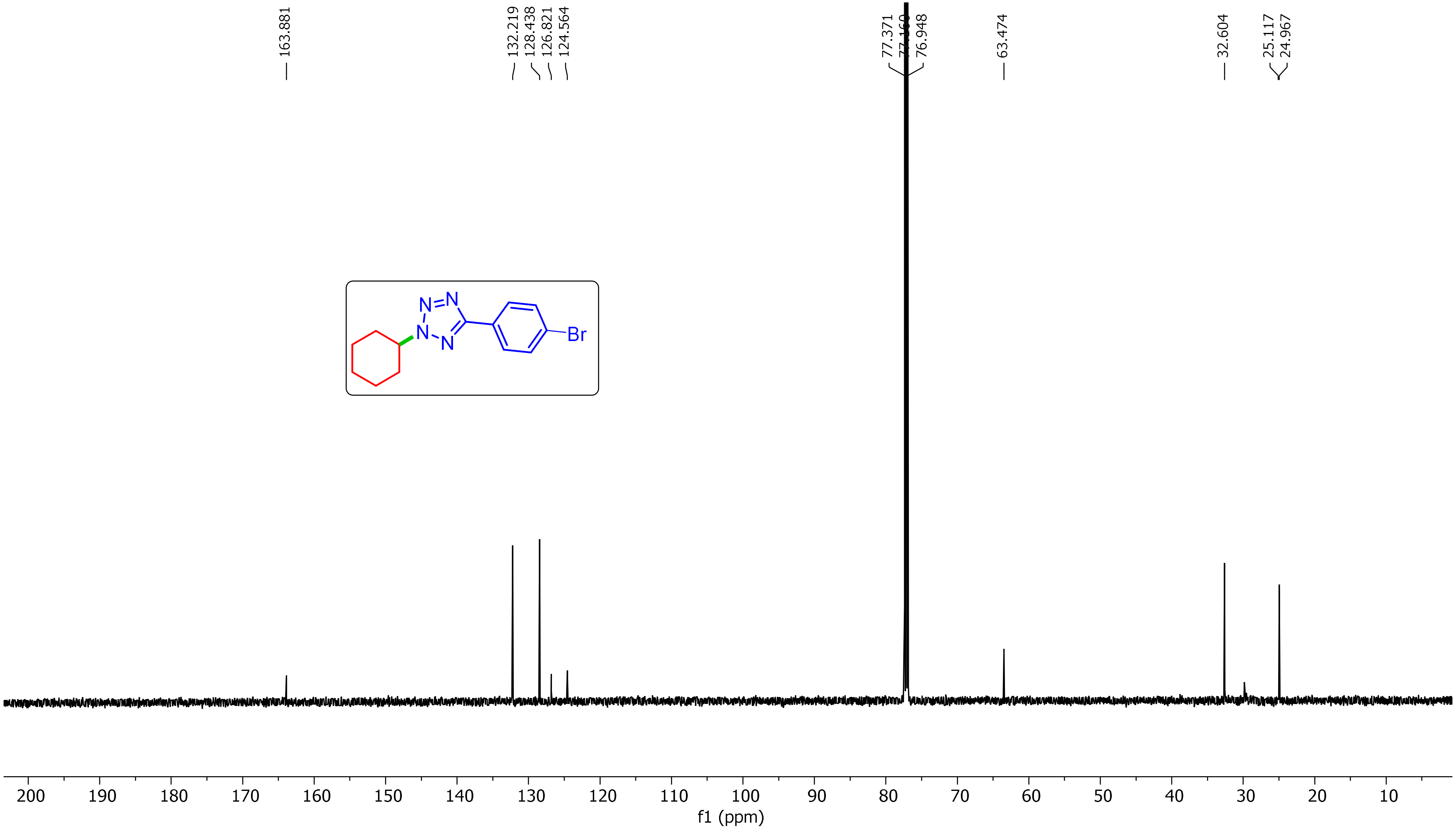

**5-(2-Bromophenyl)-2-cyclohexyl-2*H*-tetrazole (40m): $^{1}$H NMR (600 MHz, $CDCl_3$)**

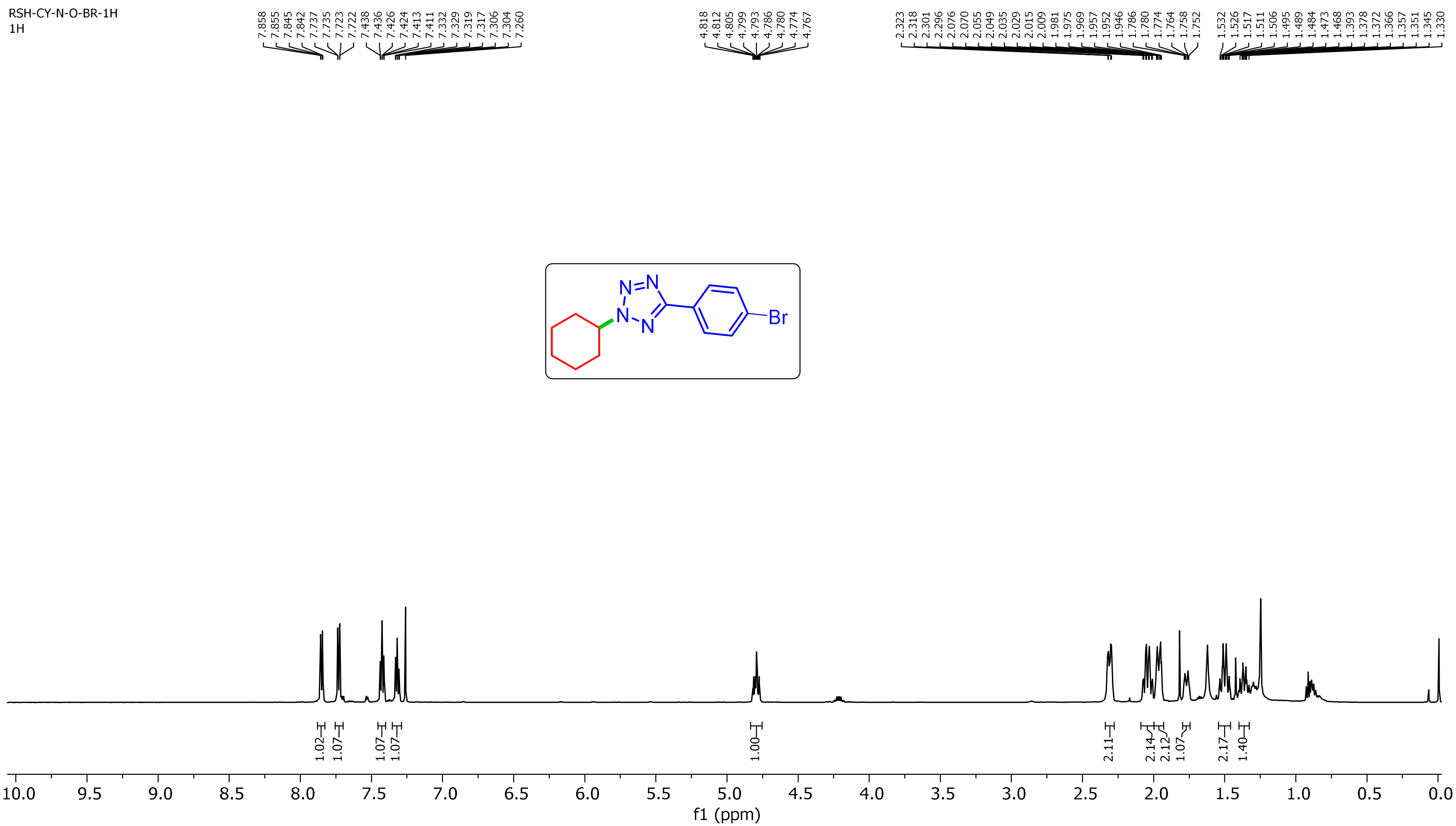

**5-(2-Bromophenyl)-2-cyclohexyl-2*H*-tetrazole (40m): $^{13}$C NMR (151 MHz, $CDCl_3$)**

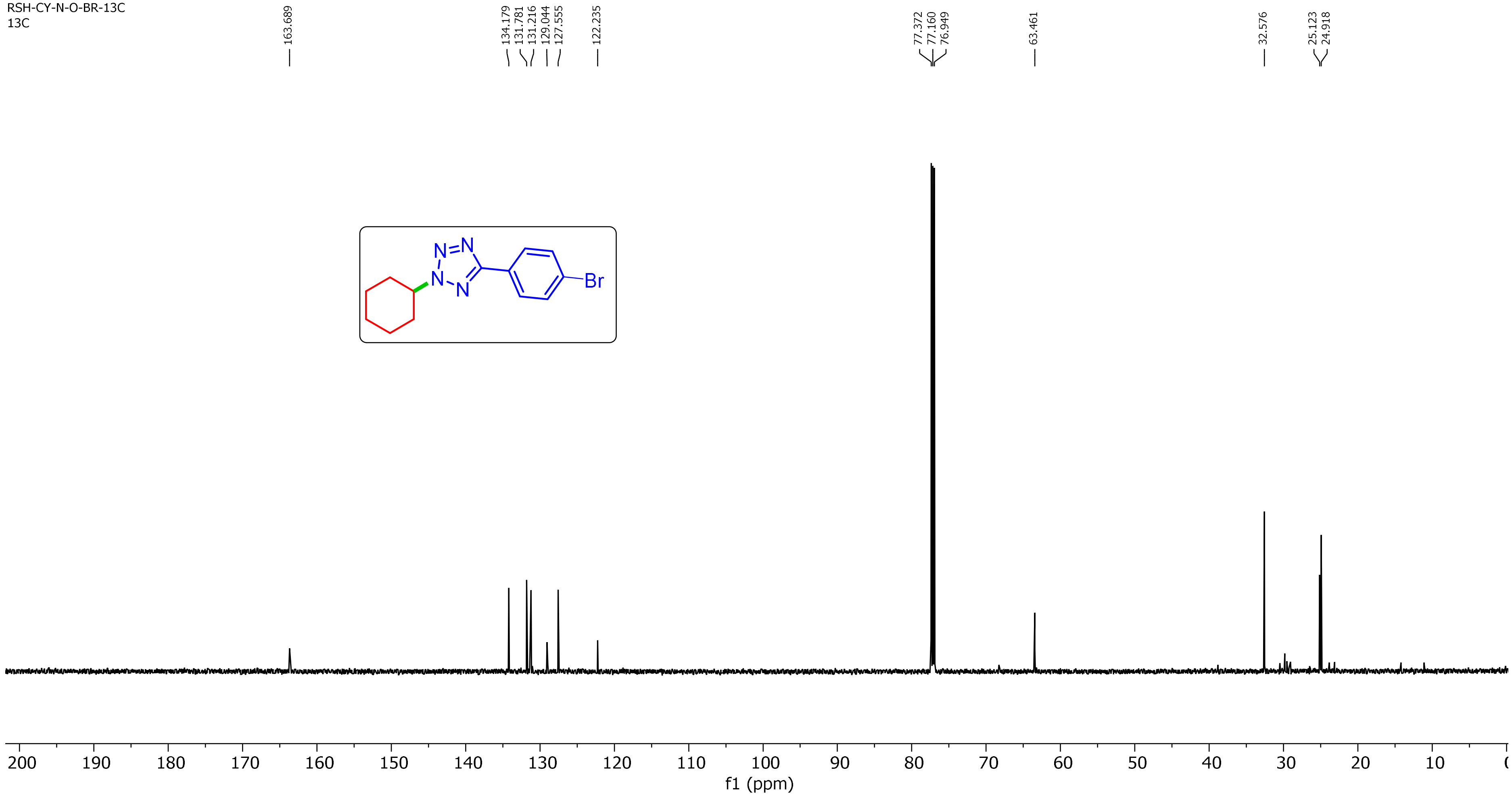

**2-Cyclohexyl-5-(thiophen-2-yl)-2*H*-tetrazole (40q): $^{1}$H NMR (600 MHz, CDCl$_3$)**

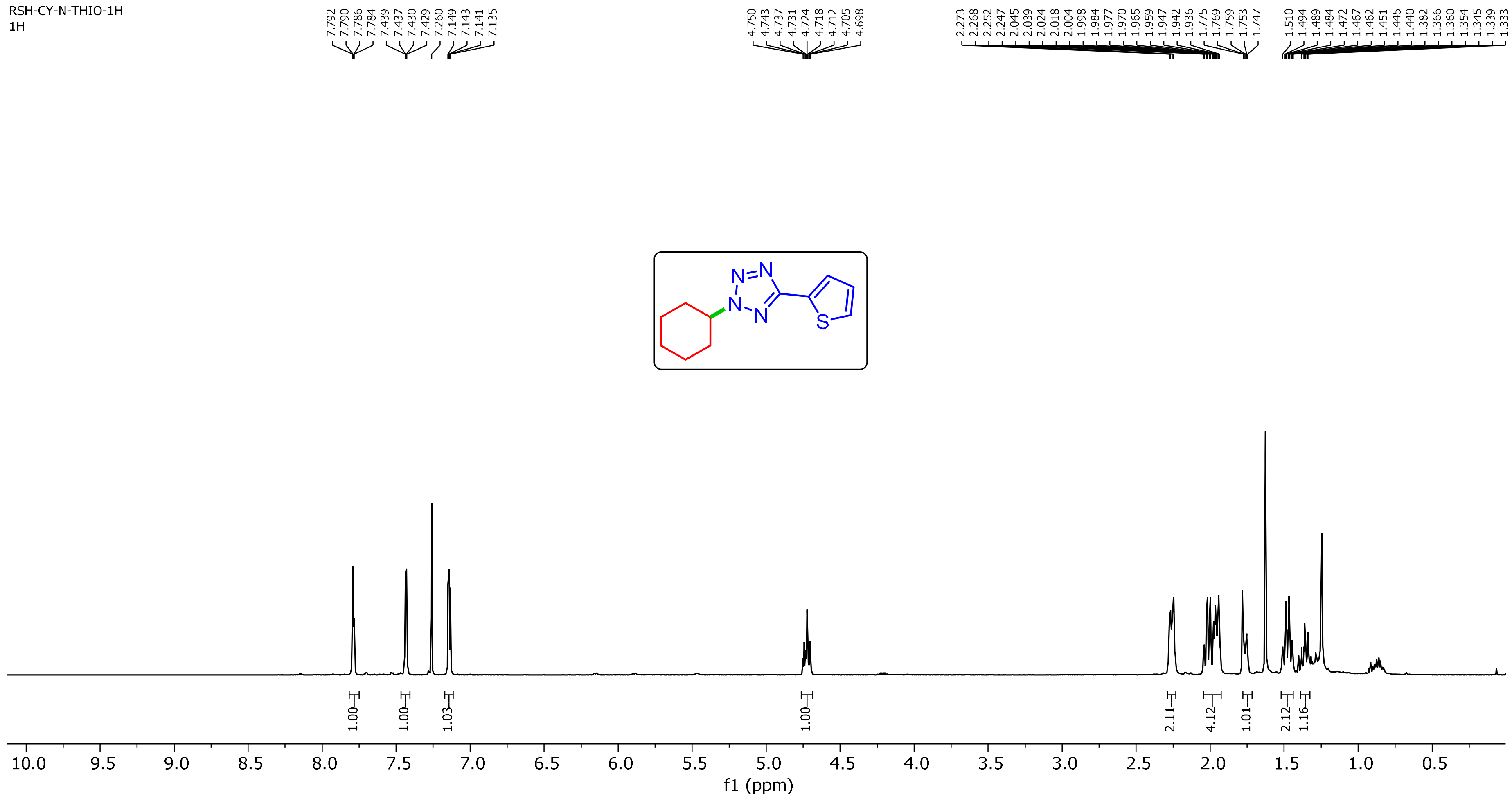

**2-Cyclohexyl-5-(thiophen-2-yl)-2*H*-tetrazole (40q): $^{13}$C NMR (151 MHz, $CDCl_3$)**

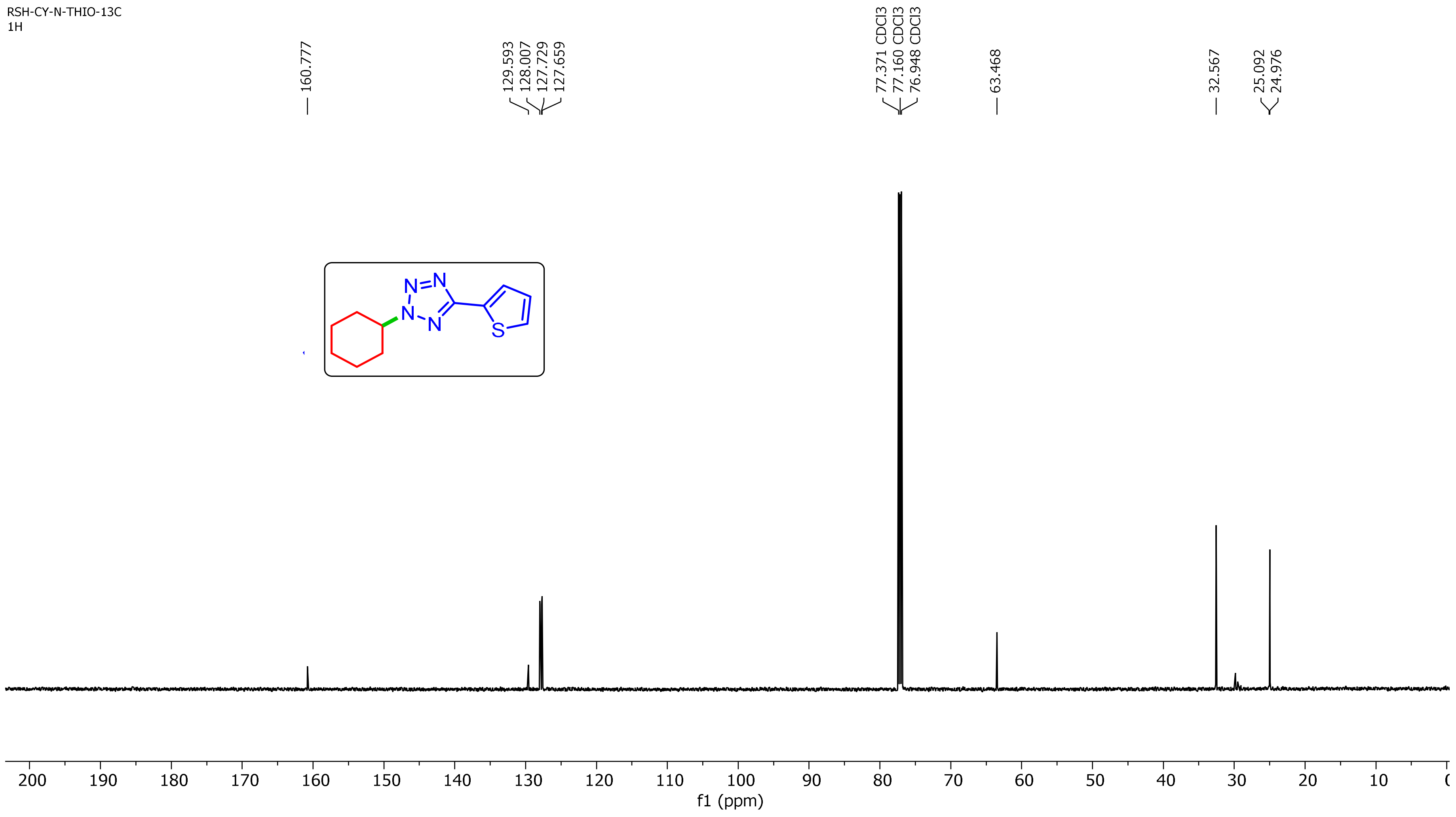

**(8S,13S,14S)-3-((*tert*-Butyldimethylsilyl)oxy)-13-methyl-9-(5-phenyl-2*H*-tetrazol-2-yl)-7,8,9,11,12,13,15,16-octahydro-6*H*-cyclopenta[*a*]phenanthren-17(14*H*)-one (41a): $^{1}$H NMR (600 MHz, CDCl$_3$)**

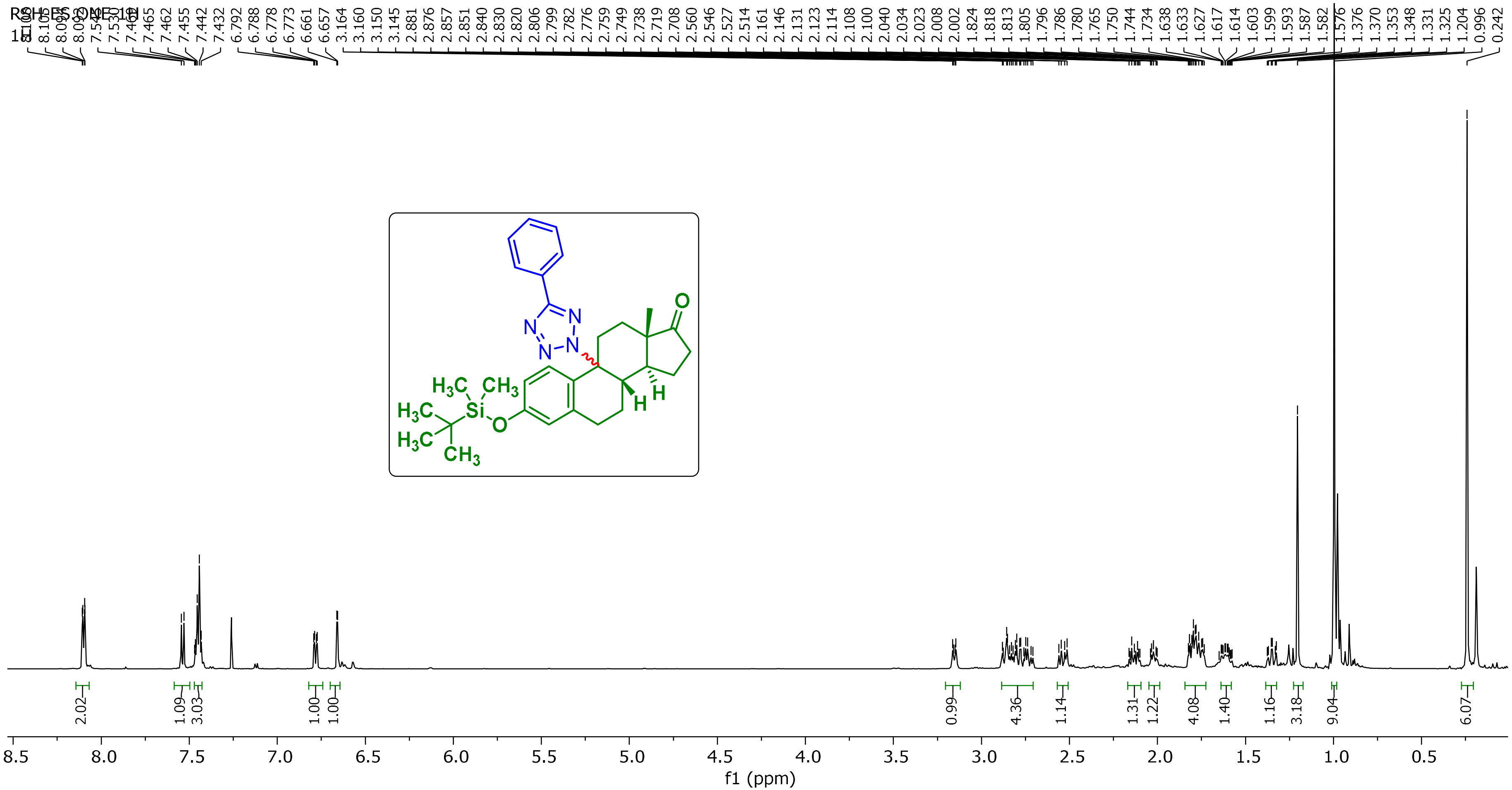

**(8S,13S,14S)-3-((*tert*-Butyldimethylsilyl)oxy)-13-methyl-9-(5-phenyl-2*H*-tetrazol-2-yl)-7,8,9,11,12,13,15,16-octahydro-6*H*-cyclopenta[*a*]phenanthren-17(14*H*)-one (41a): $^{13}$C NMR (151 MHz, CDCl$_3$)**

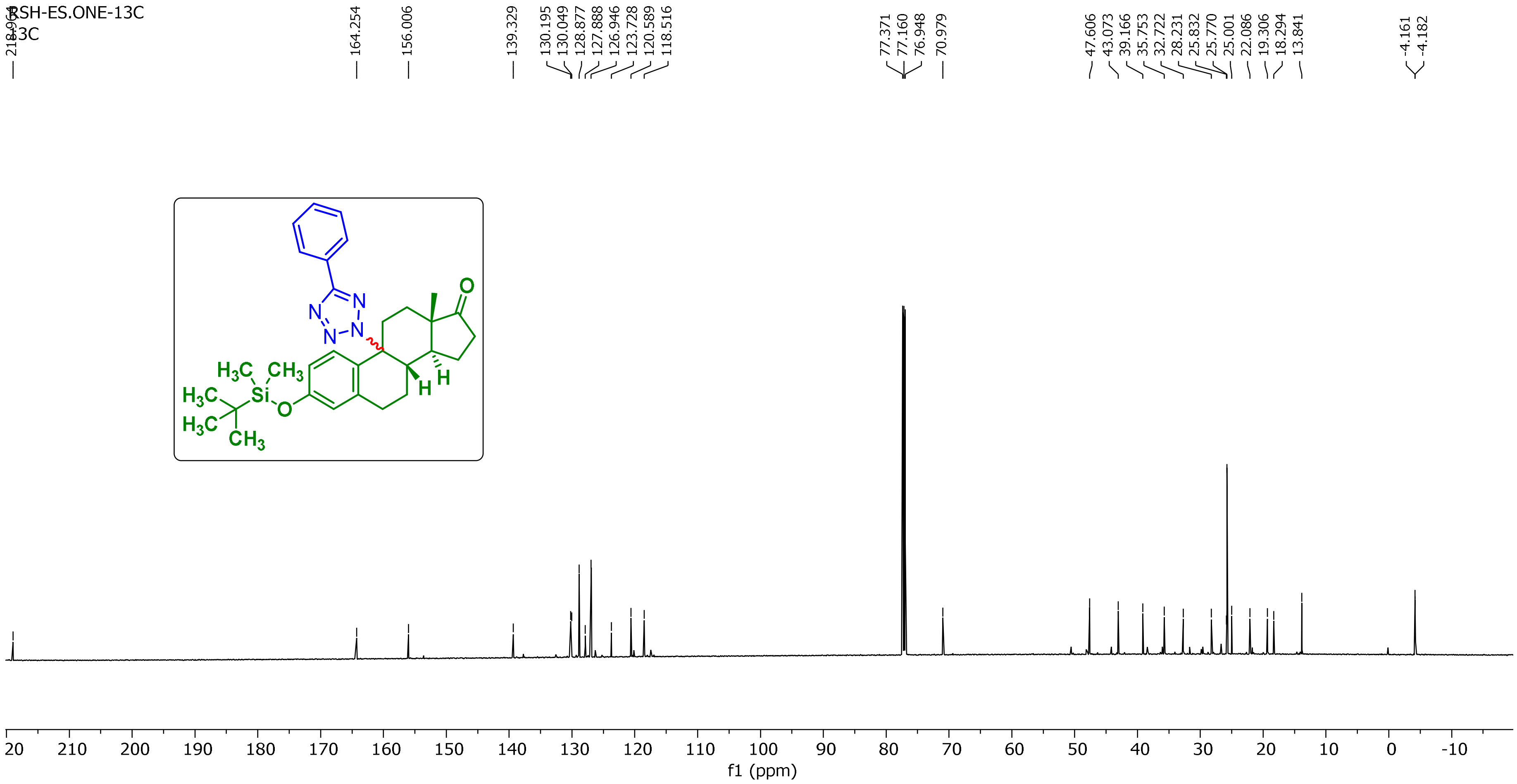

**(S)-3-(5-Phenyl-2*H*-tetrazol-2-yl)butyl (2S,5R)-3,3-dimethyl-7-oxo-4-thia-1-azabicyclo[3.2.0]heptane-2-carboxylate 4,4-dioxide (42a): $^{1}$H NMR (400 MHz, $CDCl_3$)**

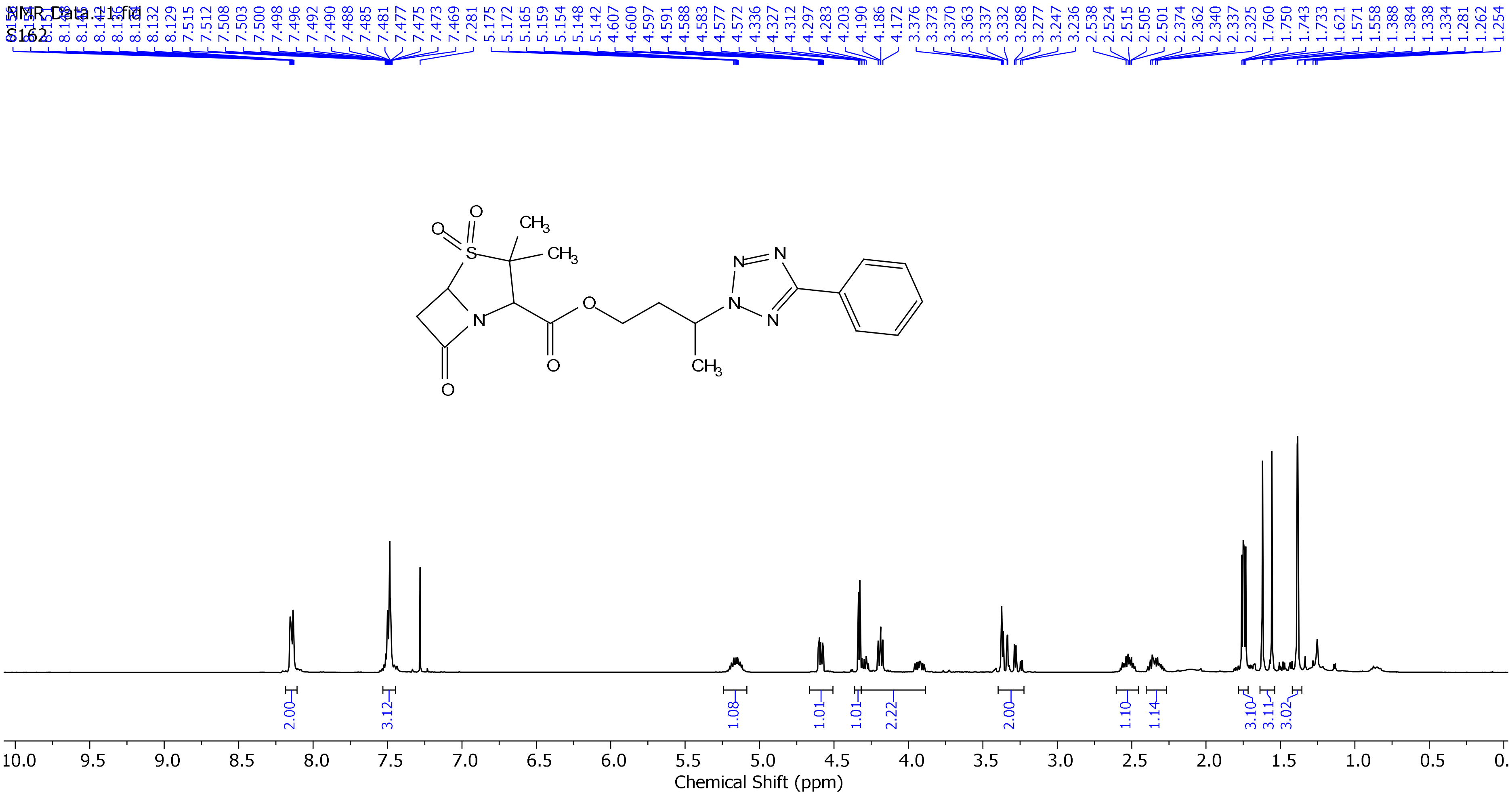

**(S)-3-(5-Phenyl-2*H*-tetrazol-2-yl)butyl (2S,5R)-3,3-dimethyl-7-oxo-4-thia-1-azabicyclo[3.2.0]heptane-2-carboxylate 4,4-dioxide (42a): $^{13}$C NMR (101 MHz, $CDCl_3$)**

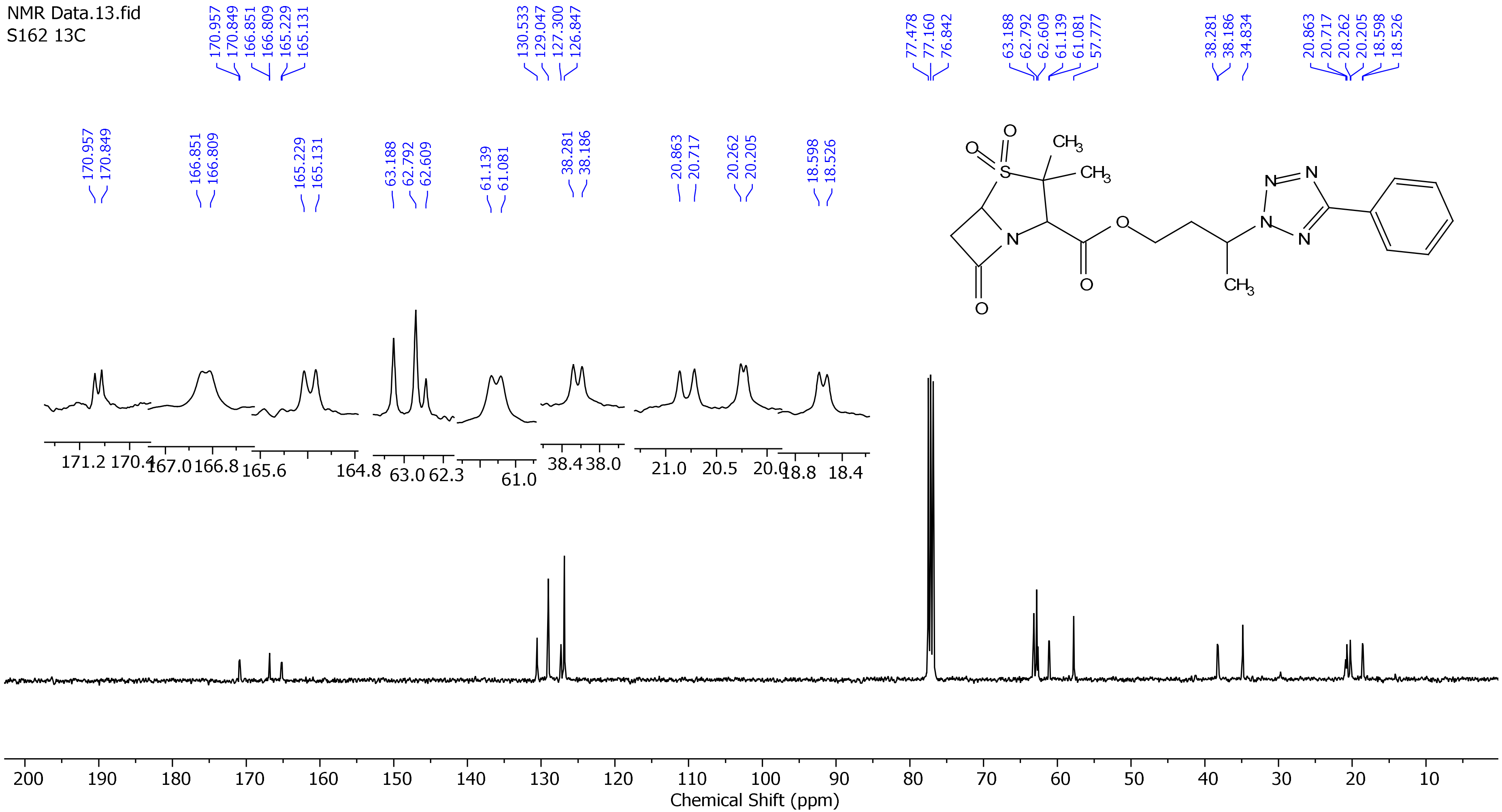

**3-(5-(4-((*tert*-Butylperoxy)carbonyl)phenyl)-2*H*-tetrazol-2-yl)butyl acetate (1za'): $^{1}$H NMR (600 MHz, $CDCl_3$)**

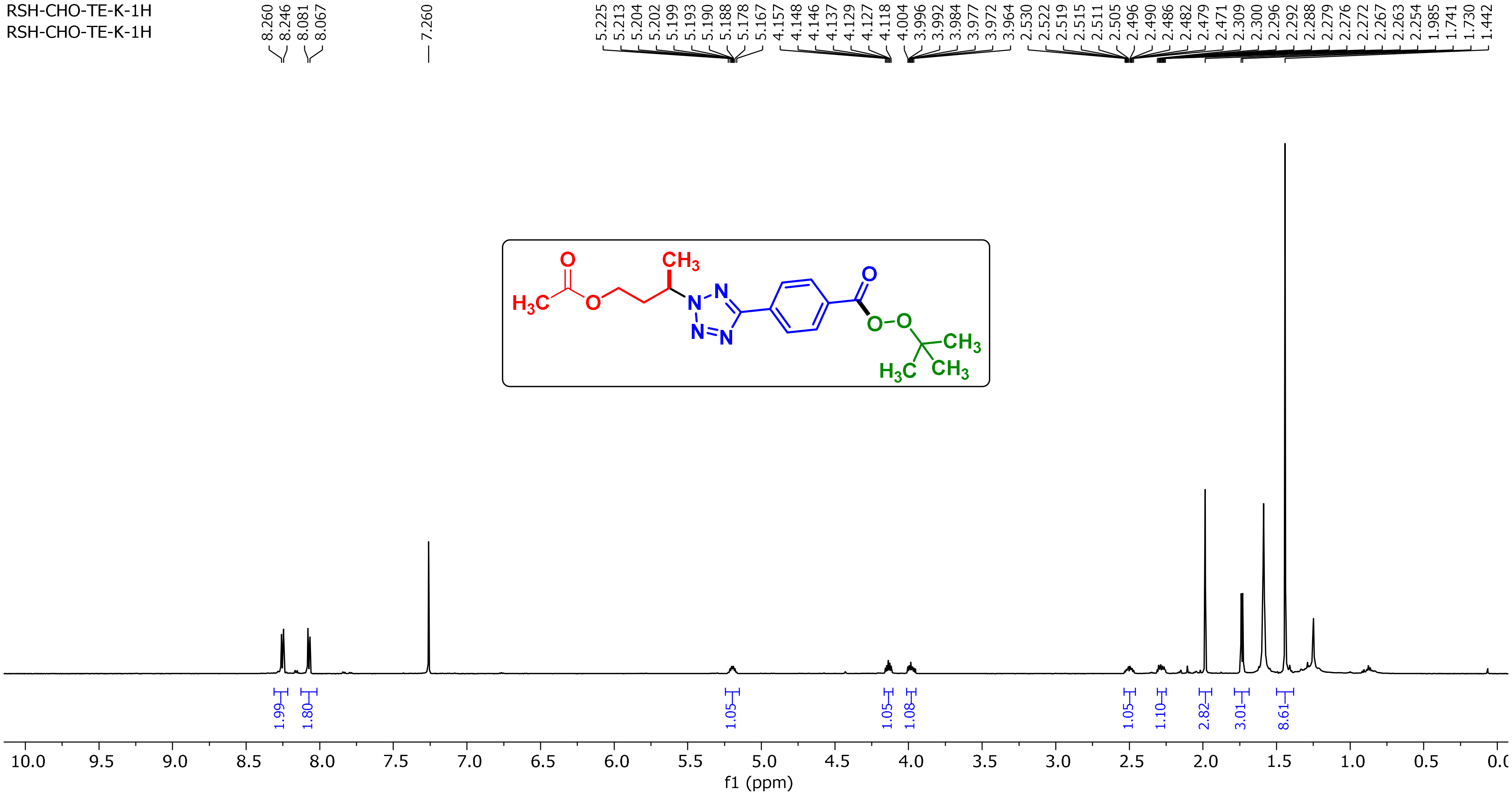

**3-(5-(4-((*tert*-Butylperoxy)carbonyl)phenyl)-2*H*-tetrazol-2-yl)butyl acetate (1za'): $^{13}$C NMR (151 MHz, $CDCl_3$)**

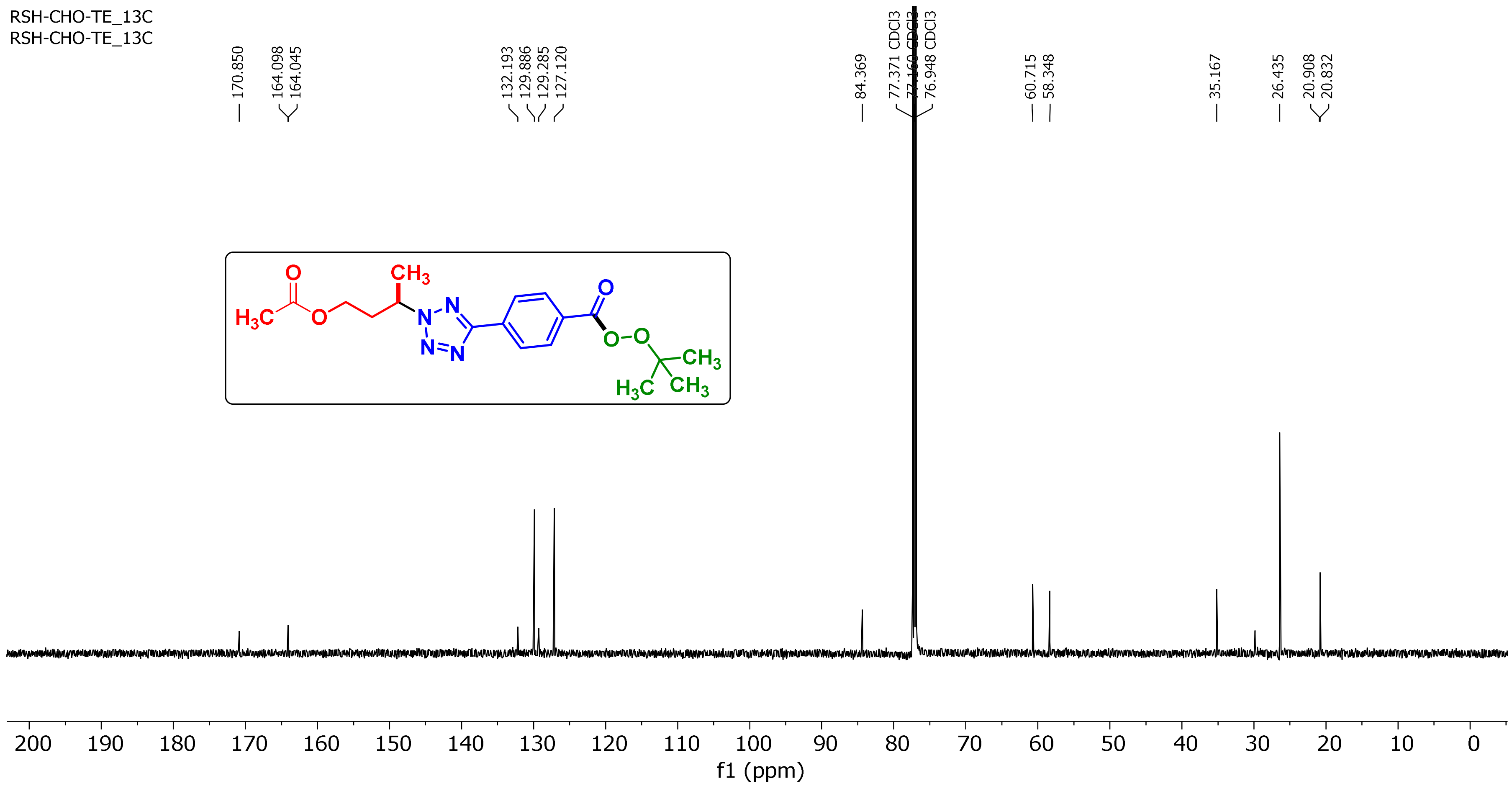

**2-Methyl-5-phenyl-2*H*-tetrazole (44a): $^{1}$H NMR (600 MHz, $CDCl_3$)**

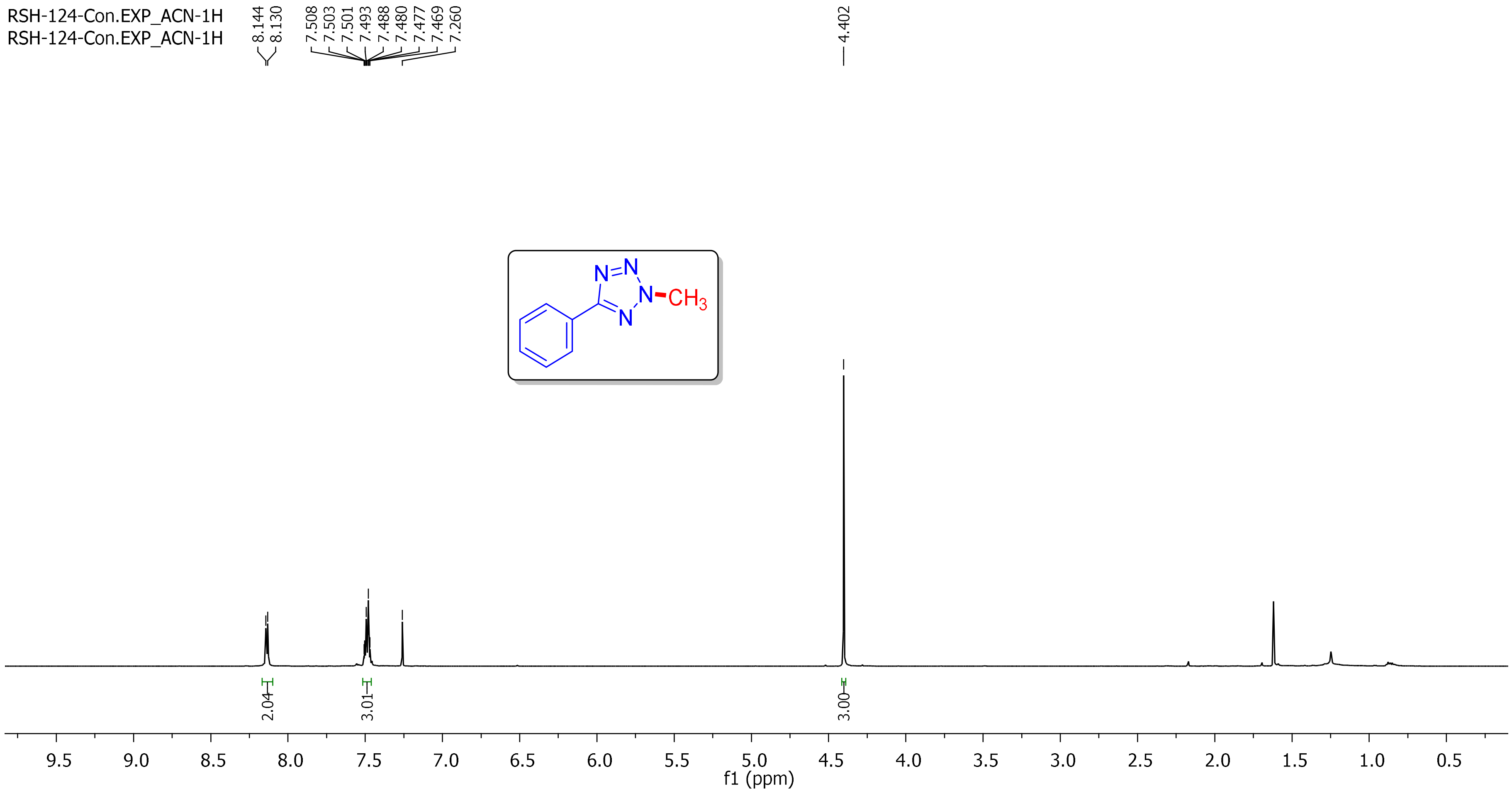

**2-Methyl-5-phenyl-2*H*-tetrazole (44a): $^{13}$C NMR (151 MHz, CDCl$_3$)**

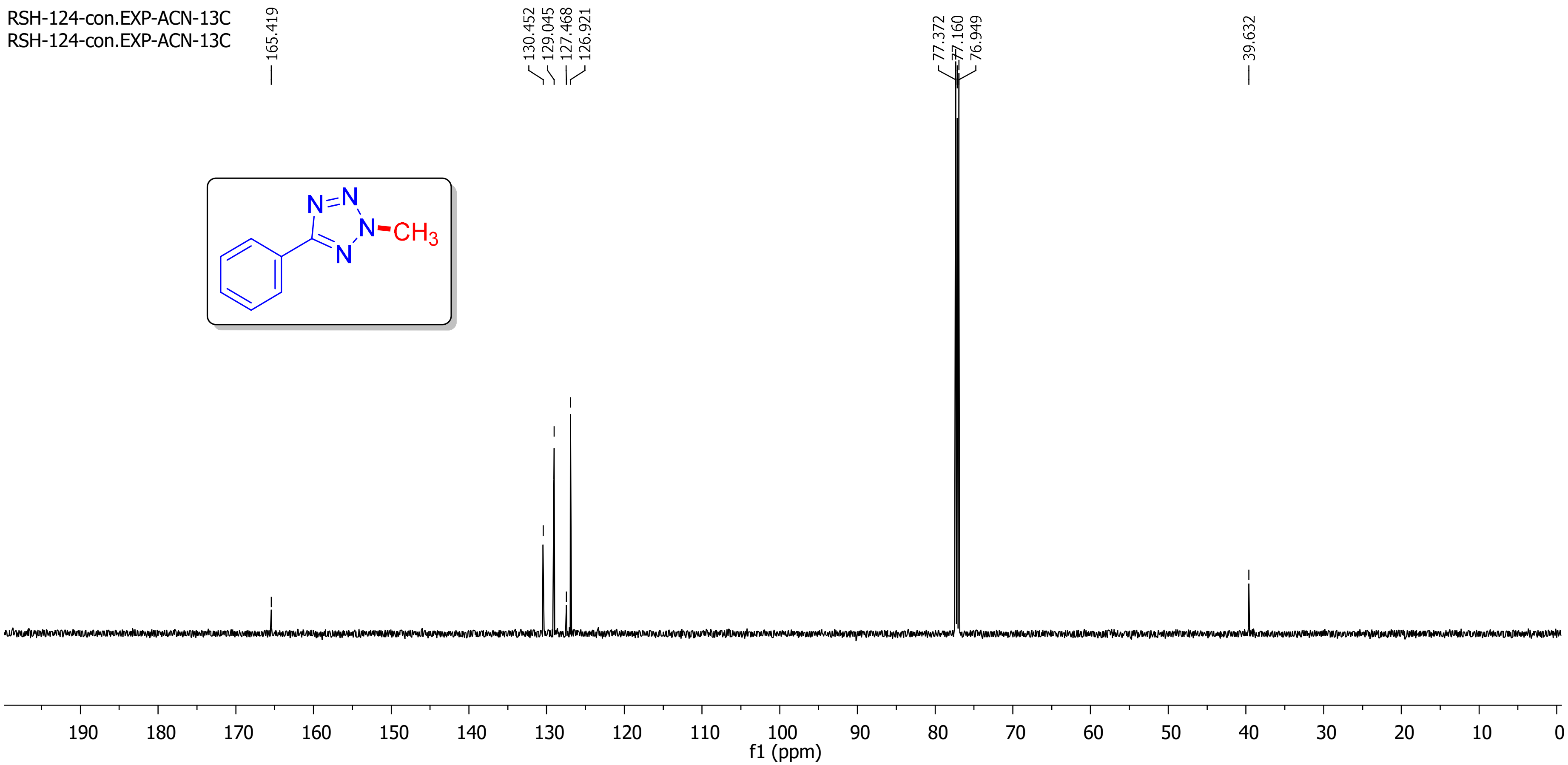

**1,1-Diphenyl-2-(5-phenyl-2*H*-tetrazol-2-yl)ethan-1-ol (45a): $^{1}$H NMR (600 MHz, $CDCl_3$)**

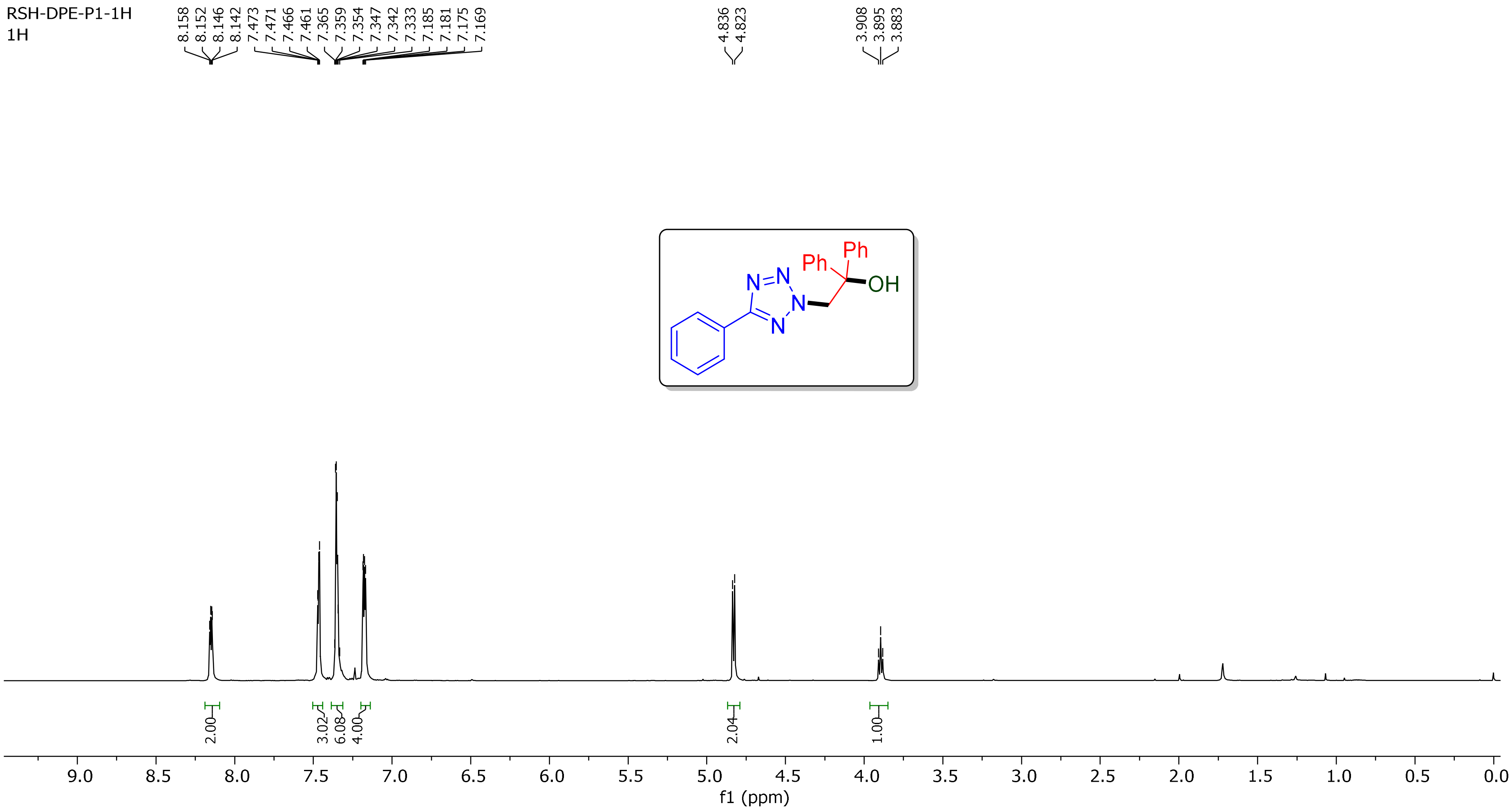

**1,1-Diphenyl-2-(5-phenyl-2*H*-tetrazol-2-yl)eth1tan-1-ol (45a):** [1]**H NMR (600 MHz, $CDCl_3 + D_2O$)**

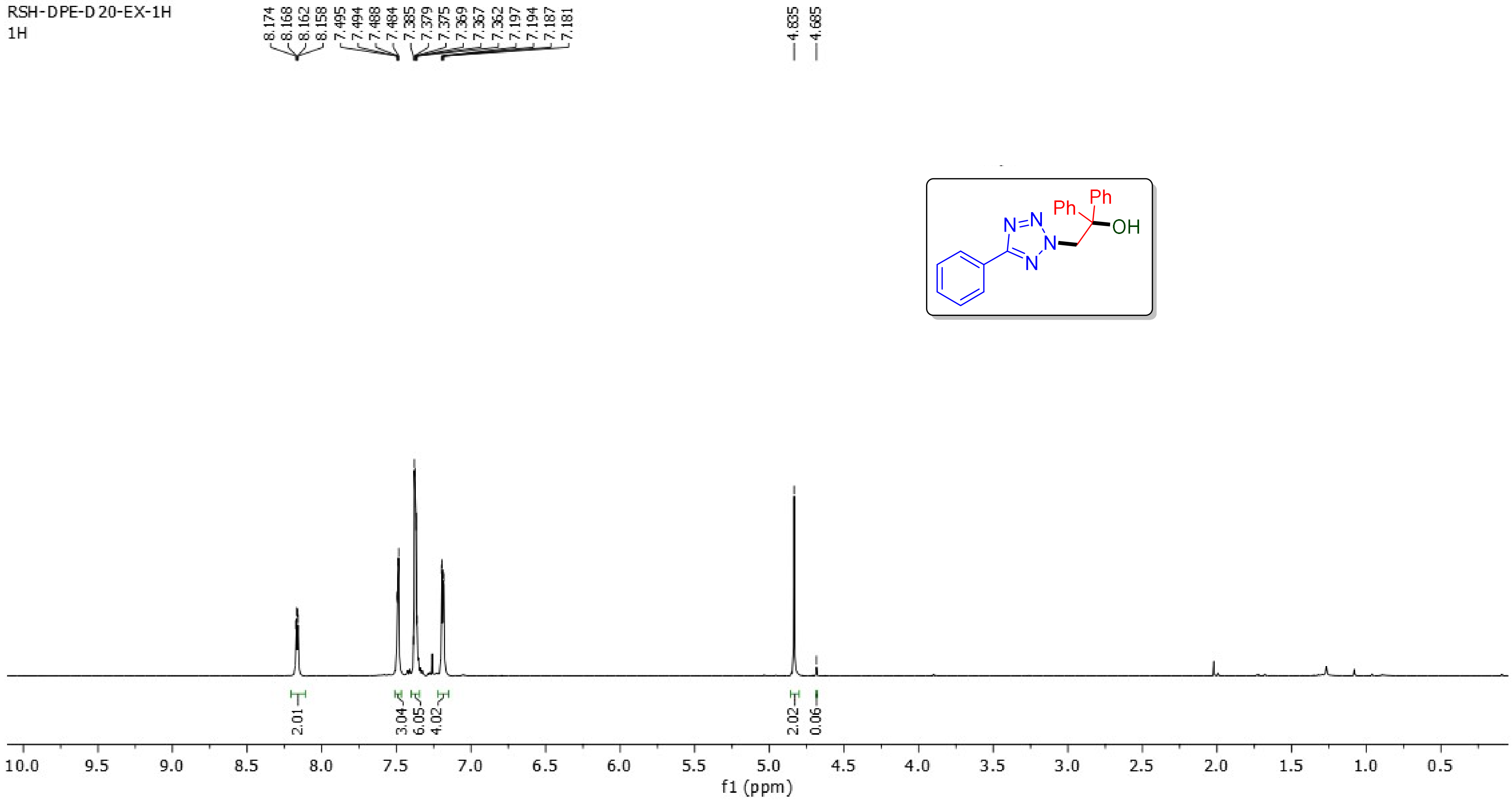

**1,1-Diphenyl-2-(5-phenyl-2*H*-tetrazol-2-yl)ethan-1-ol (45a): $^{13}$C NMR (151 MHz, $CDCl_3$)**

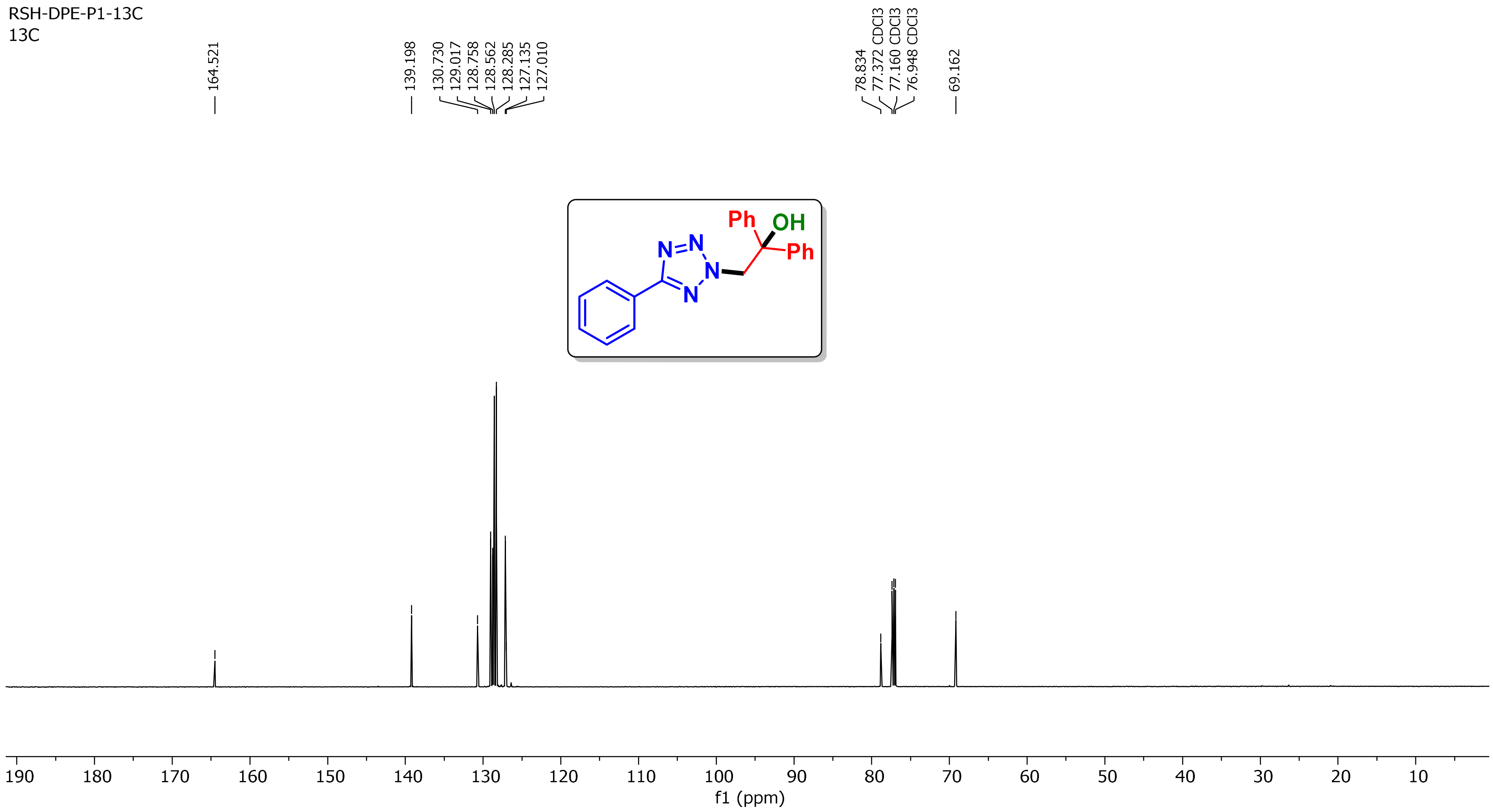

## 12. Electronic and thermodynamic parameters

**Table S4**: Electronic and thermodynamic parameters at (U)M06-2X/6-311G(d,p) (bold) and (U)wB97XD/6-311G(d,p) (normal) levels of theory.

| Species | Thermal corrected Electronic energy (E) | ZPVE (Hartree) | Lowest frequency (cm$^{-1}$) | Spin Contamination | | Point group | Electronic state | Free Energy (G) (Hartree) | Enthalpy (H) (Hartree) | Entropy (S) (Cal/Mol-Kelvin) |
|---|---|---|---|---|---|---|---|---|---|---|
| | | | | Before annhilation | After annhilation | | | | | |
| 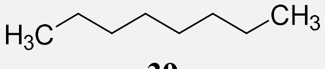 39 | **–315.358547** | **0.247189** | **44.5** | **0.0000** | **0.0000** | $C_{2h}$ | $^1A_g$ | –315.406280 | –315.357602 | **102.451** |
| | –315.441345 | 0.247394 | 50.6 | 0.0000 | 0.0000 | | | –315.488221 | –315.440400 | 100.647 |
| 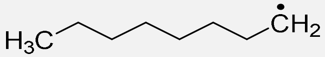 | **–314.702346** | **0.232095** | **47.0** | **0.7536** | **0.7500** | Cs | $^2A'$ | –314.752293 | –314.701402 | **107.109** |
| | –314.780555 | 0.232023 | 42.5 | 0.7545 | 0.7500 | | | –314.831074 | –314.779611 | 108.314 |
| 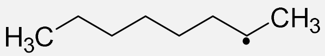 | **–314.707365** | **0.232012** | **38.5** | **0.7549** | **0.7500** | $C_1$ | $^2A$ | –314.757814 | –314.706421 | **108.165** |
| | –314.786858 | 0.231733 | 38.8 | 0.7546 | 0.7500 | | | –314.837203 | –314.785914 | 107.947 |
| 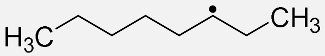 | **–314.706989** | **0.232263** | **35.5** | **0.7548** | **0.7500** | $C_1$ | $^2A$ | –314.757471 | –315.706045 | **108.234** |
| | –314.786099 | 0.232272 | 44.2 | 0.7546 | 0.7500 | | | –314.835486 | –314.785155 | 105.930 |
| 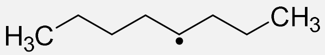 | **–314.707111** | **0.232269** | **31.7** | **0.7548** | **0.7500** | $C_1$ | $^2A$ | –314.757621 | –314.706167 | **108.295** |
| | –314.786029 | 0.232592 | 33.0 | 0.7546 | 0.7500 | | | –314.835123 | –314.785085 | 105.315 |
| 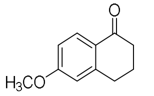 33 | **–576.533167** | **0.209387** | **67.5** | **0.0000** | **0.0000** | $C_1$ | $^1A$ | –576.580895 | –576.532223 | **102.438** |
| | –576.583653 | 0.209346 | 717.7 | 0.0000 | 0.0000 | | | –576.631250 | –576.582709 | 102.164 |
| 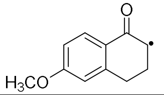 | **–575.892507** | **0.195749** | **70.4** | **0.7666** | **0.7500** | $C_1$ | $^2A$ | –575.940664 | –575.891562 | **103.343** |
| | –575.939775 | 0.195429 | 70.4 | 0.7657 | 0.7500 | | | –575.987955 | –575.938830 | 103.392 |
| 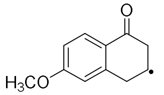 | **–575.880801** | **0.194097** | **56.8** | **0.7551** | **0.7500** | $C_1$ | $^2A$ | –575.930432 | –575.879857 | **106.444** |
| | –575.927912 | 0.193717 | 59.6 | 0.7549 | 0.7500 | | | –575.977604 | –575.926968 | 106.573 |
| | **–575.897108** | **0.195335** | **70.2** | **0.7746** | **0.7504** | $C_1$ | $^2A$ | –575.945504 | –575.896163 | **103.845** |

| | | | | | | | | | | |
|---|---|---|---|---|---|---|---|---|---|---|
| 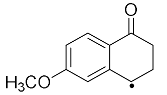 | −575.945438 | 0.194919 | 68.0 | 0.7906 | 0.7511 | | | −575.993984 | −575.944494 | 104.161 |
| 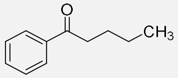 | **−502.498104** | **0.224924** | **34.2** | **0.0000** | **0.0000** | $C_S$ | $^1A'$ | **−502.549087** | **−502.497160** | **109.289** |
| **30** | −502.556800 | 0.225605 | 31.5 | 0.0000 | 0.0000 | | | −502.607810 | −502.555855 | 109.348 |
| 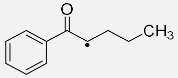 | **−501.858369** | **0.212004** | **34.7** | **0.7657** | **0.7501** | $C_1$ | $^2A$ | **−501.909564** | **−501.857425** | **109.735** |
| | −501.915126 | 0.211826 | 23.2 | 0.7652 | 0.7500 | | | −501.966779 | −501.914182 | 110.698 |
| 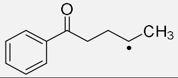 | **−501.846544** | **0.210122** | **36.0** | **0.7550** | **0.7500** | $C_1$ | $^2A$ | **−501.899206** | **−501.845600** | **112.824** |
| | −501.903328 | 0.209988 | 28.2 | 0.7547 | 0.7500 | | | −501.956297 | −501.902384 | 113.471 |
| 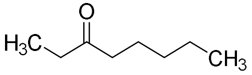 | **−389.395499** | **0.228315** | **39.4** | **0.0000** | **0.0000** | $C_1$ | $^1A$ | **−389.445485** | **−389.394555** | **107.192** |
| **32** | −389.469521 | 0.228102 | 43.4 | 0.0000 | 0.0000 | | | −389.519310 | −389.468577 | 106.778 |
| 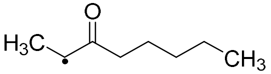 | **−388.755805** | **0.214506** | **40.1** | **0.7645** | **0.7500** | $C_S$ | $^2A''$ | **−388.806720** | **−388.754861** | **109.145** |
| | −388.827419 | 0.214406 | 36.2 | 0.7640 | 0.7500 | | | −388.878451 | −388.826475 | 109.393 |
| 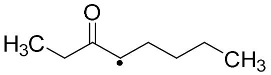 | **−388.755561** | **0.215069** | **35.0** | **0.7637** | **0.7500** | $C_1$ | $^2A$ | **−388.806434** | **−388.754617** | **109.059** |
| | −388.826713 | 0.214959 | 27.2 | 0.7640 | 0.7500 | | | −388.877226 | −388.825769 | 108.302 |
| 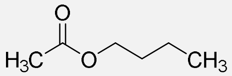 | **−386.074813** | **0.176571** | **50.0** | **0.0000** | **0.0000** | Cs | $^1A'$ | **−386.121034** | **−386.073869** | **99.266** |
| **1** | −386.131028 | 0.176195 | 40.2 | 0.0000 | 0.0000 | | | −386.177183 | −386.130084 | 99.130 |
| 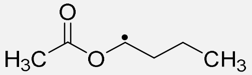 | **−385.425064** | **0.161892** | **47.8** | **0.7543** | **0.7500** | $C_1$ | $^2A$ | **−385.472587** | **−385.424119** | **102.009** |
| | −385.478082 | 0.161751 | 53.8 | 0.7543 | 0.7500 | | | −385.524741 | −385.477138 | 100.189 |
| 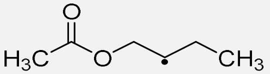 | **−385.421388** | **0.161388** | **25.7** | **0.7548** | **0.7500** | $C_1$ | $^2A$ | **−385.470309** | **−385.420444** | **104.950** |
| | −385.473897 | 0.161201 | 38.5 | 0.7545 | 0.7500 | | | −385.521552 | −385.472953 | 102.285 |
| 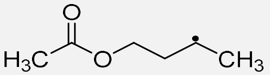 | **−385.423502** | **0.161334** | **37.2** | **0.7549** | **0.7500** | $C_1$ | $^2A$ | **−385.472116** | **−385.422558** | **104.304** |
| | −385.476023 | 0.161079 | 41.3 | 0.7546 | 0.7500 | | | −385.523810 | −385.475079 | 102.563 |
| 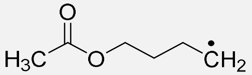 | **−385.418124** | **0.161490** | **48.2** | **0.7550** | **0.7500** | Cs | $^2A'$ | **−385.466018** | **−385.417180** | **102.788** |
| | −385.469614 | 0.161212 | 45.0 | 0.7544 | 0.7500 | | | −385.517304 | −385.468669 | 102.361 |
| | **−425.348231** | **0.205204** | **44.1** | **0.0000** | **0.0000** | Cs | $^1A'$ | **−425.397913** | **−425.347287** | **106.551** |

| | | | | | | | | | | |
|---|---|---|---|---|---|---|---|---|---|---|
| 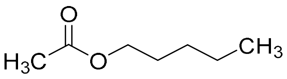 **6** | –425.413378 | 0.205071 | 38.1 | 0.0000 | 0.0000 | | | –425.463514 | –425.412434 | 107.508 |
| 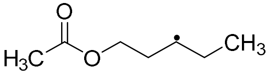 | **–424.696544** | **0.190131** | **30.5** | **0.7548** | **0.7500** | $C_1$ | $^2A$ | **–424.748935** | **–424.695600** | **112.254** |
| | –424.758073 | 0.189953 | 40.3 | 0.7546 | 0.7500 | | | –424.808959 | –424.757129 | 109.087 |
| 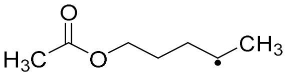 | **–424.696855** | **0.189962** | **38.7** | **0.7549** | **0.7500** | $C_1$ | $^2A$ | **–424.748848** | **–424.695911** | **111.415** |
| | –424.758592 | 0.189644 | 36.3 | 0.7546 | 0.7500 | | | –424.810045 | –424.757648 | 110.278 |
| 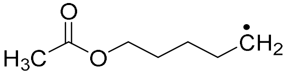 | **–424.691791** | **0.190190** | **44.4** | **0.7550** | **0.7500** | Cs | $^2A'$ | **–424.743090** | **–424.690847** | **109.954** |
| | –424.753167 | 0.189692 | 41.4 | 0.7544 | 0.7500 | | | –424.803869 | –424.752223 | 108.699 |
| 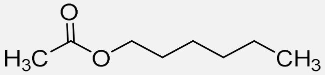 **7** | **–464.621567** | **0.233756** | **32.5** | **0.0000** | **0.0000** | Cs | $^1A'$ | **–464.675291** | **–464.620623** | **115.057** |
| | –464.696154 | 0.233530 | 31.9 | 0.0000 | 0.0000 | | | –464.749265 | –464.695210 | 113.770 |
| 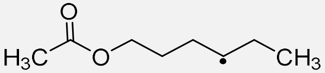 | **–463.969844** | **0.218740** | **30.7** | **0.7548** | **0.7500** | $C_1$ | $^2A$ | **–464.025621** | **–464.968900** | **119.378** |
| | –464.040625 | 0.218412 | 27.3 | 0.7546 | 0.7500 | | | –464.095416 | –464.039681 | 117.304 |
| 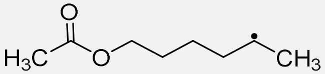 | **–463.970334** | **0.218594** | **32.9** | **0.7549** | **0.7500** | $C_1$ | $^2A$ | **–464.025927** | **–463.969390** | **118.992** |
| | –464.041287 | 0.218269 | 33.4 | 0.7546 | 0.7500 | | | –464.096237 | –464.040342 | 117.640 |
| 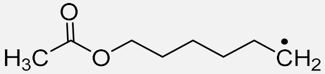 | **–463.965259** | **0.218681** | **32.4** | **0.7550** | **0.7500** | Cs | $^2A'$ | **–464.020575** | **–463.964315** | **118.409** |
| | –464.035077 | 0.218553 | 32.2 | 0.7544 | 0.7500 | | | –464.089788 | –464.034133 | 117.137 |
| 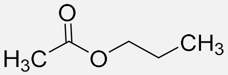 **11** | **–346.801619** | **0.147948** | **67.0** | **0.0000** | **0.0000** | Cs | $^1A'$ | **–346.844106** | **–346.800674** | **91.409** |
| | –346.848643 | 0.147436 | 55.0 | 0.0000 | 0.0000 | | | –346.891675 | –346.847699 | 92.554 |
| 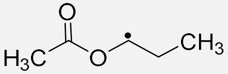 | **–346.151703** | **0.133272** | **55.8** | **0.7543** | **0.7500** | $C_1$ | $^2A$ | **–346.195578** | **–346.150759** | **94.330** |
| | –346.195735 | 0.132720 | 49.5 | 0.7543 | 0.7500 | | | –346.239848 | –346.194791 | 94.830 |
| 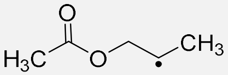 | **–346.150127** | **0.133336** | **43.3** | **0.7550** | **0.7500** | $C_1$ | $^2A$ | **–346.194287** | **–346.149183** | **94.930** |
| | –346.193223 | 0.132660 | 29.6 | 0.7545 | 0.7500 | | | –346.238455 | –346.192279 | 97.187 |
| 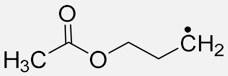 | **–346.145351** | **0.132547** | **53.8** | **0.7549** | **0.7500** | $C_1$ | $^2A$ | **–346.189909** | **–346.144407** | **95.766** |
| | –346.188030 | 0.132028 | 35.9 | 0.7544 | 0.7500 | | | –346.233001 | –346.187086 | 96.638 |
| | –619.217611 | **0.292142** | –1550.3 | **0.7564** | **0.7500** | $C_1$ | $^2A$ | –619.266352 | –619.198972 | **141.872** |

| | | | | | | | | | | |
|---|---|---|---|---|---|---|---|---|---|---|
| 1 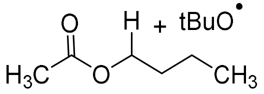 TS | −618.909637 | 0.296302 | −1601.5 | 0.7590 | 0.7500 | | | −618.955288 | −618.891665 | 133.906 |
| 1 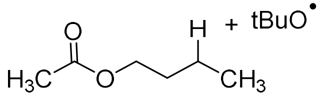 TS | −619.215532 | **0.291899** | −1512.4 | **0.7567** | **0.7500** | $C_1$ | $^2A$ | −619.264669 | −619.196803 | **142.836** |
| | −618.910175 | 0.295677 | −1392.9 | 0.7587 | 0.7500 | | | −618.957276 | −618.891941 | 137.510 |
| 11 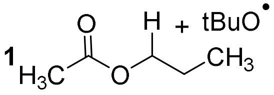 TS | **−579.619860** | **0.266904** | −1613.0 | **0.7590** | **0.7500** | $C_1$ | $^2A$ | **−579.680319** | **−579.618916** | **129.234** |
| | −579.711729 | 0.267173 | −1634.9 | 0.7581 | 0.7500 | | | −579.772142 | −579.710784 | 129.139 |
| 11 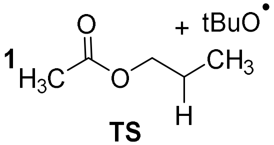 TS | **−579.617804** | **0.266964** | −1479.2 | **0.7588** | **0.7500** | $C_1$ | $^2A$ | **−579.678648** | **−579.616860** | **130.045** |
| | −579.709903 | 0.266620 | −1611.6 | 0.7582 | 0.7500 | | | −579.771220 | −579.708959 | 131.039 |
| 11 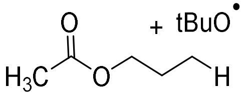 TS | **−579.613030** | **0.266959** | −1607.8 | **0.7591** | **0.7500** | $C_1$ | $^2A$ | **−579.674482** | **−579.612085** | **131.325** |
| | −579.704020 | 0.267037 | −1755.5 | 0.7583 | 0.7500 | | | −579.764973 | −579.703076 | 130.274 |
| 32 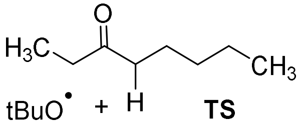 TS | −622.213161 | **0.346864** | −1565.3 | **0.7605** | **0.7500** | $C_1$ | $^2A$ | −622.281546 | −622.212217 | **145.915** |
| | −622.333107 | 0.347865 | −1630.3 | 0.7603 | 0.7500 | | | −622.399679 | −622.332163 | 142.100 |
| 32 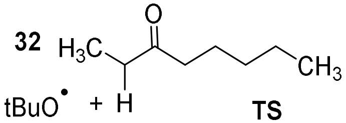 TS | −622.212722 | **0.347034** | −1468.7 | **0.7603** | **0.7500** | $C_1$ | $^2A$ | −622.281317 | −622.211778 | **146.356** |
| | −622.332791 | 0.347505 | −1539.2 | 0.7602 | 0.7500 | | | −622.400139 | −622.331847 | 143.734 |
| 24 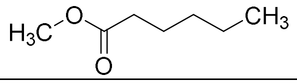 | −425.345041 | **0.205598** | 41.92 | **0.0000** | **0.0000** | **Cs** | **$^1A'$** | −425.394187 | −425.344096 | **105.424** |
| 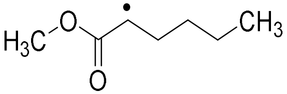 | −424.702165 | **0.192310** | 43.32 | **0.7579** | **0.7500** | **$C_1$** | **$^2A$** | −424.751629 | −425.751221 | **106.092** |

| | | | | | | | | | | |
|---|---|---|---|---|---|---|---|---|---|---|
| 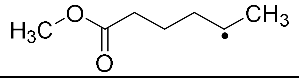 | −424.693792 | **0.190195** | 34.83 | **0.7549** | **0.7500** | **$C_1$** | **$^{2}A$** | −424.745654 | −424.692848 | **111.140** |
| 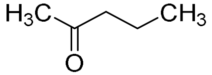 | −271.573532 | **0.141848** | 39.25 | **0.0000** | **0.0000** | **$C_1$** | **$^{1}A$** | −271.614049 | −271.572588 | **87.263** |
| 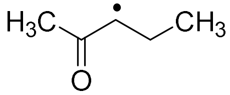 | −270.933831 | **0.128745** | 36.56 | **0.7644** | **0.7500** | **$C_1$** | **$^{2}A$** | −270.975042 | −270.932887 | **88.722** |

# 13. Cartesian coordinates at (U)M06-2X/6-311G(d,p) level of theory.

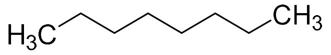

| | | | |
|---|---|---|---|
| C | 0.25827486 | 4.46637023 | 0.00000000 |
| H | 0.90080439 | 4.52539406 | 0.88230800 |
| H | 0.90080439 | 4.52539406 | -0.88230800 |
| H | -0.39241151 | 5.34284207 | 0.00000000 |
| C | -0.54524551 | 3.16823348 | 0.00000000 |
| H | -1.20112535 | 3.13997741 | 0.87676100 |
| H | -1.20112535 | 3.13997741 | -0.87676100 |
| C | 0.34440632 | 1.92689679 | 0.00000000 |
| H | 1.00200718 | 1.95377224 | 0.87744700 |
| H | 1.00200718 | 1.95377224 | -0.87744700 |
| C | -0.44520109 | 0.62022058 | 0.00000000 |
| H | -1.10252106 | 0.59425779 | -0.87751600 |
| H | -1.10252106 | 0.59425779 | 0.87751600 |
| C | 0.44520109 | -0.62022058 | 0.00000000 |
| H | 1.10252106 | -0.59425779 | 0.87751600 |
| H | 1.10252106 | -0.59425779 | -0.87751600 |
| C | -0.34440632 | -1.92689679 | 0.00000000 |
| H | -1.00200718 | -1.95377224 | -0.87744700 |
| H | -1.00200718 | -1.95377224 | 0.87744700 |
| C | 0.54524551 | -3.16823348 | 0.00000000 |
| H | 1.20112535 | -3.13997741 | 0.87676100 |
| H | 1.20112535 | -3.13997741 | -0.87676100 |
| C | -0.25827486 | -4.46637023 | 0.00000000 |
| H | -0.90080439 | -4.52539406 | -0.88230800 |
| H | 0.39241151 | -5.34284207 | 0.00000000 |
| H | -0.90080439 | -4.52539406 | 0.88230800 |

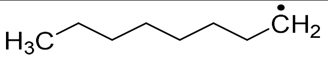

| | | | |
|---|---|---|---|
| C | 0.26523606 | 4.39159908 | 0.00000000 |
| H | 0.90795040 | 4.44830351 | 0.88231300 |
| H | 0.90795040 | 4.44830351 | -0.88231300 |
| H | -0.38225671 | 5.27041783 | 0.00000000 |
| C | -0.54331004 | 3.09656953 | 0.00000000 |
| H | -1.19928895 | 3.07085219 | 0.87673700 |
| H | -1.19928895 | 3.07085219 | -0.87673700 |
| C | 0.34165512 | 1.85190440 | 0.00000000 |
| H | 0.99930235 | 1.87612840 | 0.87745300 |
| H | 0.99930235 | 1.87612840 | -0.87745300 |
| C | -0.45351624 | 0.54842776 | 0.00000000 |
| H | -1.11077084 | 0.52499688 | -0.87751400 |
| H | -1.11077084 | 0.52499688 | 0.87751400 |
| C | 0.43255804 | -0.69510869 | 0.00000000 |
| H | 1.08971306 | -0.67248164 | 0.87767700 |
| H | 1.08971306 | -0.67248164 | -0.87767700 |
| C | -0.36476669 | -1.99727119 | 0.00000000 |
| H | -1.01968200 | -2.02955347 | -0.87766700 |
| H | -1.01968200 | -2.02955347 | 0.87766700 |
| C | 0.53786688 | -3.24702356 | 0.00000000 |
| H | 1.18913246 | -3.20131589 | 0.87956400 |
| H | 1.18913246 | -3.20131589 | -0.87956400 |
| C | -0.23542218 | -4.51992042 | 0.00000000 |
| H | -0.60613094 | -4.93966964 | -0.92575800 |
| H | -0.60613094 | -4.93966964 | 0.92575800 |

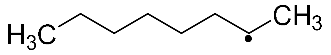

| | | | |
|---|---|---|---|
| C | -4.41685212 | 0.27852606 | 0.05813720 |
| H | -4.45851310 | 0.85894101 | 0.98341824 |
| H | -4.48358013 | 0.98082110 | -0.77679476 |
| H | -5.29808313 | -0.36490093 | 0.02614018 |
| C | -3.12505013 | -0.53168595 | -0.01650886 |
| H | -3.08918612 | -1.24654800 | 0.81254710 |
| H | -3.11443415 | -1.12579890 | -0.93661989 |
| C | -1.87711612 | 0.34767103 | 0.02611517 |
| H | -1.88530610 | 0.94249498 | 0.94753720 |
| H | -1.91211412 | 1.06501808 | -0.80285379 |
| C | -0.57696413 | -0.44892898 | -0.04972989 |
| H | -0.56935815 | -1.04283393 | -0.97177093 |
| H | -0.54337412 | -1.16657602 | 0.77892107 |
| C | 0.66880788 | 0.43167401 | -0.00578086 |
| H | 0.66842090 | 1.02318996 | 0.91657017 |
| H | 0.63649788 | 1.14906205 | -0.83422482 |
| C | 1.97055987 | -0.36573200 | -0.08626293 |
| H | 1.95999085 | -0.97128595 | -1.00899596 |
| H | 2.00566688 | -1.09611604 | 0.73405103 |
| C | 3.19377288 | 0.48594999 | -0.04532890 |
| H | 3.12203689 | 1.50517501 | -0.40965384 |
| C | 4.53962088 | -0.12973704 | 0.12566105 |
| H | 4.85044386 | -0.68561099 | -0.77168999 |
| H | 5.30855389 | 0.61822194 | 0.32466008 |
| H | 4.54166388 | -0.84967608 | 0.95094701 |

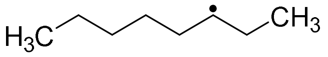

| | | | |
|---|---|---|---|
| C | 4.43057592 | -0.17998291 | 0.10184005 |
| H | 4.47192990 | -0.81467420 | 0.99078084 |
| H | 4.54871595 | -0.82566461 | -0.77218816 |
| H | 5.28463592 | 0.49902409 | 0.13481830 |
| C | 3.10856192 | 0.58104010 | 0.03952828 |
| H | 3.02057389 | 1.24043381 | 0.90958350 |
| H | 3.09761393 | 1.23071540 | -0.84219550 |
| C | 1.89776793 | -0.34845989 | -0.00845307 |
| H | 1.90778791 | -1.00024119 | 0.87369571 |
| H | 1.98385295 | -1.00924559 | -0.87976329 |
| C | 0.56918492 | 0.39979512 | -0.06975485 |
| H | 0.55820794 | 1.04968542 | -0.95268563 |
| H | 0.47788689 | 1.05864883 | 0.80098037 |
| C | -0.64273607 | -0.53130587 | -0.11962620 |
| H | -0.63443909 | -1.19269017 | 0.75744758 |
| H | -0.53753004 | -1.20364458 | -0.98843942 |
| C | -1.94453807 | 0.19240414 | -0.18469399 |
| H | -1.96887507 | 1.16764230 | -0.66374566 |
| C | -3.23611207 | -0.51969495 | 0.03679974 |
| H | -3.15301409 | -1.15266425 | 0.92933253 |
| H | -3.42808005 | -1.21768967 | -0.79428550 |
| C | -4.42520809 | 0.42978300 | 0.17989703 |
| H | -4.54119007 | 1.03876930 | -0.71990677 |
| H | -5.35661408 | -0.11611507 | 0.33911182 |
| H | -4.27644111 | 1.10623771 | 1.02423726 |

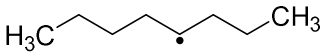

| | | | |
|---|---|---|---|
| C | 4.41580910 | 0.39090122 | 0.15668382 |
| H | 4.50841609 | 1.01655472 | -0.73478154 |
| H | 4.36457207 | 1.05461671 | 1.02358244 |
| H | 5.32611311 | -0.20511470 | 0.24397916 |
| C | 3.17188212 | -0.49025085 | 0.07401830 |
| H | 3.25322214 | -1.16433834 | -0.78553932 |
| H | 3.11203212 | -1.12846635 | 0.96217566 |
| C | 1.88562610 | 0.32297905 | -0.04627718 |
| H | 1.94239410 | 0.96218555 | -0.93537954 |
| H | 1.79596507 | 0.99637153 | 0.81362544 |
| C | 0.63070212 | -0.54585303 | -0.13347770 |
| H | 0.57331713 | -1.19808153 | 0.74870167 |
| H | 0.73314915 | -1.23156851 | -0.99212031 |
| C | -0.62966590 | 0.24164687 | -0.25245816 |
| H | -0.58422191 | 1.21878661 | -0.72592871 |
| C | -1.96343188 | -0.40436907 | -0.09127081 |
| H | -1.94751288 | -1.06615857 | 0.78560257 |
| H | -2.17165986 | -1.06799956 | -0.94830344 |
| C | -3.11291691 | 0.59531798 | 0.04468661 |
| H | -3.12651591 | 1.24281848 | -0.83821476 |
| H | -2.91731893 | 1.24505847 | 0.90324924 |
| C | -4.46767889 | -0.08842996 | 0.20491598 |
| H | -4.48083789 | -0.72155845 | 1.09586534 |
| H | -5.27569591 | 0.63923908 | 0.29945456 |
| H | -4.68737287 | -0.72399845 | -0.65689366 |

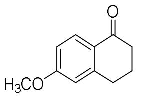

| | | | |
|---|---|---|---|
| C | -1.98038295 | -0.23530584 | -0.01840493 |
| C | -0.96804499 | -1.19523988 | -0.08443983 |
| C | 0.36669003 | -0.82327593 | -0.09697888 |
| C | 0.69536507 | 0.54303807 | -0.03892603 |
| C | -0.32097390 | 1.49354811 | 0.03211787 |
| C | -1.65606791 | 1.12423816 | 0.04104992 |
| H | 1.10611696 | -2.79301291 | 0.28951733 |
| H | -1.25905302 | -2.23888288 | -0.11874471 |
| C | 1.45323299 | -1.86981297 | -0.17950677 |
| C | 2.11053409 | 1.00980502 | -0.05261509 |
| H | -0.03566386 | 2.53798011 | 0.07838775 |
| H | -2.42368288 | 1.88338319 | 0.09587684 |
| C | 3.18042905 | -0.05970003 | -0.17193198 |
| C | 2.75042101 | -1.38140395 | 0.46237017 |
| H | 3.35966404 | -0.20954915 | -1.24446097 |
| H | 2.59169702 | -1.23783882 | 1.53580715 |
| O | -3.24349897 | -0.71689280 | -0.01099087 |
| C | -4.30674394 | 0.21228825 | 0.06482904 |
| H | -4.30371992 | 0.88719015 | -0.79627604 |
| H | -5.21996896 | -0.37749172 | 0.05957711 |
| H | -4.25522191 | 0.79674035 | 0.98828397 |
| O | 2.39571813 | 2.18342002 | 0.01044478 |
| H | 4.09817307 | 0.34162599 | 0.25885597 |
| H | 3.53685898 | -2.13000698 | 0.34760024 |
| H | 1.64029598 | -2.10142909 | -1.23543675 |

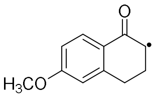

| | | | |
|---|---|---|---|
| C | -1.94359810 | -0.23208616 | -0.01279119 |
| C | -0.93283307 | -1.19166714 | -0.12196711 |
| C | 0.39931392 | -0.81603310 | -0.14442104 |
| C | 0.73091988 | 0.54671191 | -0.05234406 |
| C | -0.27988715 | 1.49601589 | 0.04471886 |
| C | -1.61687514 | 1.12394285 | 0.06802279 |
| H | -1.22515004 | -2.23315715 | -0.19146510 |
| C | 1.49504396 | -1.83833807 | -0.32715495 |
| C | 2.15386587 | 0.99289395 | -0.05423899 |
| H | 0.00548682 | 2.53978990 | 0.10359985 |
| H | -2.38304317 | 1.88211483 | 0.15028573 |
| C | 3.15114989 | -0.03078202 | 0.13998710 |
| C | 2.77233191 | -1.44320702 | 0.42344411 |
| H | 4.18303587 | 0.29729301 | 0.16747815 |
| O | -3.20763309 | -0.71497020 | 0.00583475 |
| C | -4.26793612 | 0.21388278 | 0.11118066 |
| H | -4.27477009 | 0.90387976 | -0.73800836 |
| H | -5.18245710 | -0.37401125 | 0.10756362 |
| H | -4.20380419 | 0.78268380 | 1.04374965 |
| O | 2.46381184 | 2.17466096 | -0.17769600 |
| H | 3.59224794 | -2.12106600 | 0.17748117 |
| H | 1.72394803 | -1.91436810 | -1.39596794 |
| H | 1.15005697 | -2.82123908 | -0.00127995 |
| H | 2.59604385 | -1.54744900 | 1.50483610 |

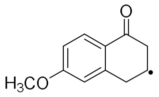

| | | | |
|---|---|---|---|
| C | 1.95489801 | -0.23043188 | 0.01204201 |
| C | 0.95246805 | -1.20124093 | 0.03940098 |
| C | -0.38647296 | -0.84379799 | 0.03666096 |
| C | -0.73092002 | 0.51761500 | 0.00792599 |
| C | 0.27729093 | 1.48041205 | -0.02575997 |
| C | 1.61581995 | 1.12683911 | -0.02229796 |
| H | 1.25362510 | -2.24264091 | 0.05330896 |
| C | -1.44950991 | -1.92453203 | 0.06331092 |
| C | -2.14649504 | 0.97848394 | 0.02073298 |
| H | -0.01890011 | 2.52241403 | -0.05276395 |
| H | 2.37520392 | 1.89574614 | -0.04716593 |
| C | -3.21732200 | -0.09174312 | 0.19918594 |
| C | -2.79156993 | -1.41409908 | -0.33136809 |
| H | -3.39601901 | -0.14304215 | 1.28835093 |
| O | 3.22318603 | -0.69766282 | 0.01496902 |
| C | 4.27693299 | 0.24468022 | -0.02530594 |
| H | 4.25376295 | 0.90084920 | 0.84983008 |
| H | 5.19653102 | -0.33506573 | -0.01900694 |
| H | 4.23227998 | 0.84801924 | -0.93684492 |
| O | -2.44276109 | 2.14578992 | -0.07069700 |
| H | -4.13810701 | 0.27734685 | -0.25222307 |
| H | -3.51071589 | -2.07591411 | -0.79268511 |
| H | -1.48231991 | -2.33445206 | 1.08781291 |
| H | -1.13946087 | -2.75138900 | -0.57995609 |

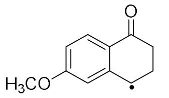


| | | | |
|---|---|---|---|
| C | 1.96877400 | -0.25180109 | 0.01846998 |
| C | 0.97304798 | -1.21903707 | 0.04056295 |
| C | -0.38743401 | -0.85898203 | 0.00556298 |
| C | -0.71829598 | 0.52787898 | -0.02602096 |
| C | 0.29352005 | 1.47569495 | -0.06235593 |
| C | 1.63360004 | 1.11070892 | -0.04145795 |
| H | 1.26886295 | -2.26105408 | 0.06433990 |
| C | -1.40534404 | -1.83829201 | -0.04804906 |
| C | -2.13252796 | 0.97400401 | 0.03156107 |
| H | 0.00754807 | 2.52070396 | -0.09333688 |
| H | 2.39731906 | 1.87506890 | -0.06280092 |
| C | -3.15025399 | -0.09562698 | 0.38292503 |
| C | -2.83159903 | -1.45078196 | -0.25730203 |
| H | -3.11917498 | -0.19992703 | 1.47407903 |
| H | -3.02901003 | -1.39117791 | -1.33823403 |
| O | 3.24022599 | -0.71532613 | 0.05047395 |
| C | 4.29268202 | 0.22788885 | 0.01481999 |
| H | 4.25460604 | 0.89704781 | 0.87963502 |
| H | 5.21271300 | -0.35050918 | 0.04597095 |
| H | 4.26475802 | 0.81780789 | -0.90623899 |
| O | -2.45343594 | 2.12997002 | -0.12700887 |
| H | -4.13915398 | 0.27055606 | 0.10840906 |
| H | -3.50208404 | -2.22024596 | 0.13098094 |
| H | -1.12771906 | -2.88534901 | -0.04282111 |

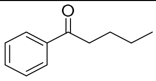


| | | | |
|---|---|---|---|
| C | -1.30890941 | 0.18215060 | 0.00000000 |
| C | -2.32333293 | 1.14157028 | 0.00000000 |
| C | -3.65440233 | 0.75310959 | 0.00000000 |
| C | -3.98411969 | -0.60020430 | 0.00000000 |
| C | -2.97991242 | -1.56150164 | 0.00000000 |
| C | -1.64510971 | -1.17216507 | 0.00000000 |
| H | -2.03929934 | 2.18679825 | 0.00000000 |
| H | -4.43720355 | 1.50195755 | 0.00000000 |
| H | -5.02406833 | -0.90464524 | 0.00000000 |
| H | -3.23547626 | -2.61414531 | 0.00000000 |
| H | -0.87113033 | -1.93017715 | 0.00000000 |
| C | 0.11568835 | 0.66253026 | 0.00000000 |
| C | 1.22803570 | -0.37015567 | 0.00000000 |
| H | 1.09589961 | -1.01831862 | 0.87482000 |
| H | 1.09589961 | -1.01831862 | -0.87482000 |
| C | 2.61554333 | 0.25596124 | 0.00000000 |
| H | 2.71531378 | 0.90804698 | -0.87264700 |
| H | 2.71531378 | 0.90804698 | 0.87264700 |
| C | 3.72732775 | -0.79034893 | 0.00000000 |
| H | 3.61790632 | -1.43768578 | -0.87706900 |
| H | 3.61790632 | -1.43768578 | 0.87706900 |
| C | 5.11640307 | -0.15713184 | 0.00000000 |
| H | 5.25494856 | 0.47286889 | 0.88210700 |
| H | 5.90298185 | -0.91382655 | 0.00000000 |
| H | 5.25494856 | 0.47286889 | -0.88210700 |
| O | 0.36159865 | 1.84516605 | 0.00000000 |

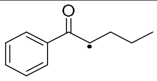


| | | | |
|---|---|---|---|
| C | 1.06123604 | 0.14699199 | -0.17806596 |
| C | 1.88944598 | 1.13052517 | 0.36531780 |
| C | 3.21927397 | 0.85225029 | 0.64521497 |
| C | 3.73948402 | -0.41005078 | 0.37191337 |
| C | 2.92536408 | -1.38985397 | -0.18474339 |
| C | 1.59024709 | -1.11403408 | -0.45674156 |
| H | 1.46580594 | 2.10882022 | 0.55616148 |
| H | 3.85335792 | 1.61896843 | 1.07386978 |
| H | 4.77883801 | -0.62758669 | 0.58737951 |
| H | 3.33055012 | -2.36858802 | -0.41152707 |
| H | 0.97449114 | -1.88174523 | -0.90968837 |
| C | -0.36555095 | 0.51772386 | -0.46508417 |
| C | -1.35440291 | -0.51813222 | -0.65942692 |
| H | -1.08825789 | -1.55630917 | -0.50905360 |
| C | -2.76402790 | -0.14573634 | -0.95210812 |
| H | -2.76526688 | 0.70007945 | -1.64406337 |
| H | -3.28240485 | -0.98626050 | -1.42165191 |
| C | -3.52809599 | 0.27554901 | 0.32319970 |
| H | -4.53361798 | 0.58722390 | 0.02859454 |
| H | -3.02821604 | 1.15205915 | 0.74144647 |
| C | -3.61295403 | -0.83922568 | 1.36229503 |
| H | -2.62059105 | -1.11876255 | 1.72515317 |
| H | -4.20457509 | -0.52776844 | 2.22475390 |
| H | -4.08060598 | -1.73348482 | 0.94067726 |
| O | -0.69995298 | 1.69966384 | -0.50158454 |

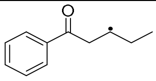


gamma radical

| | | | |
|---|---|---|---|
| C | -1.17360397 | 0.21718797 | -0.01528394 |
| C | -2.26834695 | 1.06347301 | 0.17164498 |
| C | -3.55510996 | 0.54751806 | 0.19966401 |
| C | -3.75937600 | -0.82115195 | 0.04098912 |
| C | -2.67452503 | -1.67014700 | -0.14548080 |
| C | -1.38415901 | -1.15308204 | -0.17374484 |
| H | -2.08155791 | 2.12349802 | 0.29256589 |
| H | -4.40079394 | 1.20872909 | 0.34511995 |
| H | -4.76458401 | -1.22520192 | 0.06266615 |
| H | -2.83261306 | -2.73462200 | -0.26894172 |
| H | -0.54642203 | -1.82415708 | -0.32023078 |
| C | 0.19797405 | 0.83231392 | -0.03587299 |
| C | 1.39612702 | -0.07773013 | -0.23858290 |
| H | 1.39556499 | -0.83222207 | 0.55669716 |
| H | 1.26559601 | -0.62715020 | -1.17783686 |
| C | 2.72438005 | 0.69287283 | -0.24896796 |
| H | 2.68234407 | 1.45066977 | -1.03354102 |
| H | 2.80506306 | 1.23600790 | 0.69958900 |
| C | 3.90037402 | -0.20703522 | -0.43249588 |
| H | 4.31871202 | -0.33262232 | -1.42397986 |
| C | 4.30357598 | -1.15580315 | 0.64490520 |
| H | 3.71965596 | -2.08821513 | 0.62201727 |
| H | 5.35374398 | -1.44267119 | 0.55999023 |
| H | 4.14957299 | -0.71212406 | 1.63368917 |
| O | 0.33648208 | 2.02369793 | 0.10644392 |

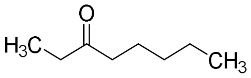


| | | | |
|---|---|---|---|
| C | -3.87757909 | 1.10301377 | -0.38403088 |
| H | -3.38803315 | 2.07352081 | -0.27081497 |
| H | -3.98912414 | 0.91000469 | -1.45423986 |
| H | -4.87685408 | 1.17853973 | 0.04875117 |
| C | -3.05691899 | 0.00272187 | 0.28409616 |
| H | -2.97858694 | 0.20112894 | 1.35803014 |
| H | -3.57422293 | -0.95767317 | 0.18258825 |
| C | -1.65209401 | -0.11648008 | -0.30222391 |
| H | -1.13213607 | 0.83998197 | -0.18727900 |
| H | -1.72595205 | -0.31396315 | -1.37927489 |
| C | -0.82724990 | -1.21856898 | 0.36020612 |
| H | -1.34555284 | -2.17633502 | 0.25179421 |
| H | -0.74540286 | -1.01172590 | 1.43084911 |
| C | 0.57653208 | -1.33707792 | -0.22194194 |
| H | 0.55030403 | -1.43337299 | -1.31361893 |
| H | 1.07751716 | -2.24207586 | 0.14328109 |
| C | 1.48347502 | -0.17298483 | 0.14520194 |
| C | 2.78913897 | -0.06864480 | -0.62259214 |
| H | 3.25923204 | -1.05808677 | -0.63480210 |
| H | 2.52856591 | 0.14406612 | -1.66628514 |
| C | 3.72299493 | 0.99403830 | -0.06255226 |
| H | 3.23427786 | 1.96838727 | -0.06243230 |
| H | 3.99006100 | 0.76554739 | 0.97005275 |
| H | 4.63782489 | 1.05900633 | -0.65276931 |
| O | 1.19103601 | 0.61711820 | 1.00739890 |

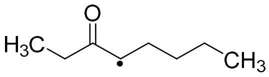


| | | | |
|---|---|---|---|
| C | 4.02229295 | 0.93574003 | 0.44485016 |
| H | 3.59629591 | 1.94197101 | 0.43919833 |
| H | 4.14439698 | 0.63168486 | 1.48761011 |
| H | 5.01426794 | 0.98837214 | -0.00745484 |
| C | 3.11697798 | -0.03932389 | -0.30355998 |
| H | 3.02642796 | 0.26792628 | -1.35042893 |
| H | 3.57032203 | -1.03639487 | -0.30900715 |
| C | 1.72259000 | -0.12193705 | 0.31253303 |
| H | 1.25419795 | 0.86696693 | 0.30523919 |
| H | 1.80067402 | -0.43577021 | 1.35954597 |
| C | 0.80342603 | -1.09569496 | -0.45283112 |
| H | 1.26506607 | -2.08653194 | -0.47743428 |
| H | 0.70473500 | -0.73030180 | -1.47899606 |
| C | -0.54657496 | -1.16515612 | 0.16244989 |
| H | -0.78146492 | -1.93499925 | 0.89031677 |
| C | -1.54089001 | -0.15591611 | -0.13523293 |
| C | -2.89342199 | -0.26905728 | 0.55220907 |
| H | -3.31216296 | -1.25368026 | 0.31627992 |
| C | -3.84713505 | 0.84503174 | 0.14739626 |
| H | -4.01508006 | 0.83255491 | -0.93000674 |
| H | -4.80730204 | 0.73799662 | 0.65376626 |
| H | -3.42538009 | 1.81930872 | 0.39627241 |
| O | -1.29183906 | 0.75729202 | -0.91206679 |
| H | -2.72387098 | -0.26955745 | 1.63474707 |

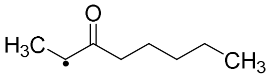


| | | | |
|---|---|---|---|
| C | -4.59215175 | 0.24365687 | 0.00000000 |
| H | -4.67765657 | 0.88313190 | 0.88242800 |
| H | -4.67765657 | 0.88313190 | -0.88242800 |
| H | -5.44127139 | -0.44222736 | 0.00000000 |
| C | -3.26300353 | -0.50715079 | 0.00000000 |
| H | -3.20702882 | -1.16120480 | 0.87648100 |
| H | -3.20702882 | -1.16120480 | -0.87648100 |
| C | -2.05843637 | 0.43166581 | 0.00000000 |
| H | -2.11346080 | 1.08771946 | 0.87765000 |
| H | -2.11346080 | 1.08771946 | -0.87765000 |
| C | -0.72612574 | -0.31258812 | 0.00000000 |
| H | -0.66324468 | -0.96981182 | -0.87241900 |
| H | -0.66324468 | -0.96981182 | 0.87241900 |
| C | 0.46937241 | 0.62918504 | 0.00000000 |
| H | 0.44011394 | 1.29121138 | 0.87424800 |
| H | 0.44011394 | 1.29121138 | -0.87424800 |
| C | 1.80364929 | -0.10144221 | 0.00000000 |
| C | 3.00357969 | 0.70820808 | 0.00000000 |
| H | 2.89933839 | 1.78832131 | 0.00000000 |
| C | 4.35466369 | 0.09835674 | 0.00000000 |
| H | 4.92740709 | 0.41634759 | -0.87762500 |
| H | 4.92740709 | 0.41634759 | 0.87762500 |
| H | 4.27275177 | -0.98760070 | 0.00000000 |
| O | 1.86345434 | -1.32407866 | 0.00000000 |

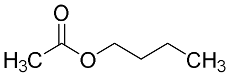


| | | | |
|---|---|---|---|
| C | 0.14290143 | 0.43913161 | 0.00000000 |
| H | 0.08168438 | 1.08226474 | 0.88213000 |
| H | 0.08168438 | 1.08226474 | -0.88213000 |
| C | 1.40613291 | -0.39471630 | 0.00000000 |
| H | 1.40212700 | -1.04609204 | -0.87953300 |
| H | 1.40212700 | -1.04609204 | 0.87953300 |
| C | 2.66126377 | 0.47549346 | 0.00000000 |
| H | 2.64890147 | 1.13105471 | 0.87698000 |
| H | 2.64890147 | 1.13105471 | -0.87698000 |
| C | 3.94024199 | -0.35792514 | 0.00000000 |
| H | 3.98467675 | -1.00084037 | -0.88250400 |
| H | 3.98467675 | -1.00084037 | 0.88250400 |
| C | -2.18660873 | 0.11735853 | 0.00000000 |
| O | -2.34900658 | 1.30554222 | 0.00000000 |
| O | -0.97347550 | -0.46142439 | 0.00000000 |
| C | -3.27813934 | -0.91795852 | 0.00000000 |
| H | -3.17719052 | -1.55359238 | -0.88041600 |
| H | -3.17719052 | -1.55359238 | 0.88041600 |
| H | -4.24460860 | -0.42159398 | 0.00000000 |
| H | 4.82931485 | 0.27476025 | 0.00000000 |

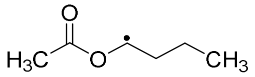


| | | | |
|---|---|---|---|
| C | -0.14968196 | 0.32699091 | -0.07372322 |
| H | -0.00705496 | 1.36403496 | 0.19925258 |
| C | -1.42131295 | -0.41221007 | 0.11427295 |
| H | -1.48102192 | -0.81578187 | 1.13787703 |
| H | -1.43297095 | -1.28248120 | -0.55239488 |
| C | -2.63977596 | 0.47170087 | -0.15332420 |
| H | -2.58348598 | 0.85511467 | -1.17616827 |
| H | -2.60351395 | 1.34288600 | 0.50852663 |
| C | -3.95222095 | -0.27865910 | 0.05314097 |
| H | -4.03454393 | -0.64536590 | 1.07937205 |
| H | -4.01594296 | -1.14123823 | -0.61488486 |
| C | 2.19043705 | 0.15021194 | 0.00696177 |
| O | 2.32290104 | 1.33641895 | 0.07520153 |
| O | 0.97697905 | -0.46194008 | -0.05412009 |
| C | 3.29244505 | -0.86899706 | -0.02824006 |
| H | 3.15974708 | -1.58267490 | 0.78548609 |
| H | 3.24290704 | -1.42324324 | -0.96659995 |
| H | 4.25022405 | -0.36392004 | 0.06044383 |
| H | -4.81272596 | 0.36261386 | -0.14409113 |

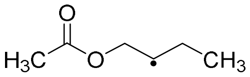


| | | | |
|---|---|---|---|
| C | 0.15071602 | 0.50562700 | -0.09742507 |
| H | 0.11975307 | 1.13399809 | 0.80010387 |
| H | -0.01413895 | 1.17956092 | -0.95122713 |
| C | 1.42619698 | -0.24200208 | -0.20061001 |
| H | 1.40240993 | -1.20938213 | -0.69002291 |
| C | 2.72505302 | 0.46725187 | -0.02554910 |
| H | 2.65232907 | 1.15942796 | 0.82202583 |
| H | 2.93358804 | 1.10084177 | -0.90218017 |
| C | 3.89525998 | -0.49417117 | 0.18482998 |
| H | 3.99720393 | -1.16753626 | -0.66933095 |
| H | 3.73564896 | -1.10447007 | 1.07580104 |
| C | -2.15732199 | 0.08845813 | 0.03507700 |
| O | -2.36419693 | 1.26879215 | 0.08370189 |
| O | -0.92528102 | -0.44123194 | -0.05385096 |
| C | -3.20597905 | -0.98950681 | 0.06428613 |
| H | -3.14083909 | -1.58485791 | -0.84718082 |
| H | -3.02132207 | -1.65484074 | 0.90831019 |
| H | -4.18826303 | -0.53291575 | 0.14843009 |
| H | 4.83590401 | 0.04575080 | 0.30280991 |

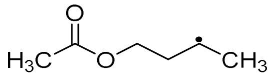


| | | | |
|---|---|---|---|
| C | 0.18924506 | 0.42026503 | -0.01023093 |
| H | 0.17038110 | 1.00782696 | 0.91056312 |
| H | 0.11702301 | 1.11559310 | -0.85066887 |
| C | 1.43882106 | -0.43367595 | -0.10082306 |
| H | 1.36411102 | -1.06000087 | -1.00367811 |
| H | 1.46263211 | -1.12905201 | 0.74585388 |
| C | 2.67847905 | 0.39486007 | -0.12458506 |
| H | 2.64072902 | 1.36244711 | -0.61344998 |
| C | 4.00466307 | -0.22170194 | 0.15586682 |
| H | 4.32494104 | -0.88619387 | -0.66022625 |
| H | 3.96997813 | -0.83646801 | 1.06105778 |
| C | -2.14353494 | 0.13286701 | 0.02590517 |
| O | -2.28664895 | 1.32166200 | 0.08842927 |
| O | -0.93944093 | -0.46276498 | -0.03263294 |
| C | -3.25046393 | -0.88549700 | 0.00228714 |
| H | -3.17501097 | -1.48526293 | -0.90536691 |
| H | -3.14350888 | -1.55861007 | 0.85373108 |
| H | -4.20901393 | -0.37551802 | 0.04136023 |
| H | 4.78320107 | 0.53135906 | 0.28393284 |

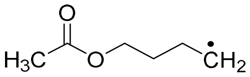


| | | | |
|---|---|---|---|
| C | 0.21222125 | 0.42403733 | 0.00000000 |
| H | 0.15649049 | 1.06757177 | 0.88226600 |
| H | 0.15649049 | 1.06757177 | -0.88226600 |
| C | 1.46961179 | -0.41974349 | 0.00000000 |
| H | 1.46961098 | -1.06859412 | -0.87995100 |
| H | 1.46961098 | -1.06859412 | 0.87995100 |
| C | 2.73430093 | 0.46052929 | 0.00000000 |
| H | 2.70656172 | 1.11225179 | 0.87972300 |
| H | 2.70656172 | 1.11225179 | -0.87972300 |
| C | 3.98824903 | -0.34409979 | 0.00000000 |
| H | 4.39984281 | -0.72116078 | -0.92648300 |
| H | 4.39984281 | -0.72116078 | 0.92648300 |
| C | -2.11887557 | 0.11930292 | 0.00000000 |
| O | -2.27144745 | 1.30863826 | 0.00000000 |
| O | -0.90949041 | -0.46869985 | 0.00000000 |
| C | -3.21783654 | -0.90783781 | 0.00000000 |
| H | -3.12146207 | -1.54421583 | -0.88039200 |
| H | -3.12146207 | -1.54421583 | 0.88039200 |
| H | -4.18061033 | -0.40434359 | 0.00000000 |

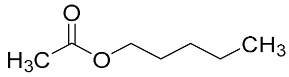


| | | | |
|---|---|---|---|
| C | 0.38481340 | 0.43676505 | 0.00000000 |
| H | 1.02996314 | 0.47035822 | 0.88214600 |
| H | 1.02996314 | 0.47035822 | -0.88214600 |
| C | -0.50281081 | -0.78930346 | 0.00000000 |
| H | -1.15302143 | -0.75656555 | -0.87966400 |
| H | -1.15302143 | -0.75656555 | 0.87966400 |
| C | 0.31325352 | -2.07971933 | 0.00000000 |
| H | 0.97017961 | -2.09894625 | 0.87772700 |
| H | 0.97017961 | -2.09894625 | -0.87772700 |
| C | -0.56132826 | -3.33181670 | 0.00000000 |
| H | -1.21692105 | -3.31137751 | -0.87686600 |
| H | -1.21692105 | -3.31137751 | 0.87686600 |
| C | 0.16329163 | 2.77805046 | 0.00000000 |
| O | 1.35737024 | 2.88937929 | 0.00000000 |

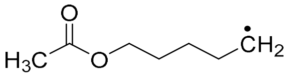


| | | | |
|---|---|---|---|
| C | -0.36946343 | 0.39283408 | 0.00000000 |
| H | -0.40542492 | 1.03768134 | 0.88217300 |
| H | -0.40542492 | 1.03768134 | -0.88217300 |
| C | 0.86030231 | -0.48986458 | 0.00000000 |
| H | 0.83097125 | -1.14000167 | -0.87983400 |
| H | 0.83097125 | -1.14000167 | 0.87983400 |
| C | 2.14594499 | 0.33404033 | 0.00000000 |
| H | 2.17034296 | 0.98841977 | 0.87795600 |
| H | 2.17034296 | 0.98841977 | -0.87795600 |
| C | 3.40685046 | -0.55259135 | 0.00000000 |
| H | 3.36958532 | -1.20393331 | -0.87966200 |
| H | 3.36958532 | -1.20393331 | 0.87966200 |
| C | -2.70953267 | 0.16126469 | 0.00000000 |
| O | -2.82524788 | 1.35481812 | 0.00000000 |

| | | | |
|---|---|---|---|
| O | -0.46695568 | 1.59089191 | 0.00000000 |
| C | -0.82419275 | 3.91304922 | 0.00000000 |
| H | -1.46363501 | 3.83952773 | -0.88038200 |
| H | -1.46363501 | 3.83952773 | 0.88038200 |
| H | -0.28675148 | 4.85729927 | 0.00000000 |
| C | 0.26063207 | -4.61799264 | 0.00000000 |
| H | -0.37873186 | -5.50250914 | 0.00000000 |
| H | 0.90354177 | -4.66857432 | -0.88242000 |
| H | 0.90354177 | -4.66857432 | 0.88242000 |

| | | | |
|---|---|---|---|
| O | -1.51951611 | -0.46424904 | 0.00000000 |
| C | -3.84044127 | -0.83078198 | 0.00000000 |
| H | -3.76436733 | -1.46988508 | -0.88040800 |
| H | -3.76436733 | -1.46988508 | 0.88040800 |
| H | -4.78679685 | -0.29706447 | 0.00000000 |
| C | 4.66826731 | 0.23925305 | 0.00000000 |
| H | 5.08556404 | 0.61151218 | -0.92606700 |
| H | 5.08556404 | 0.61151218 | 0.92606700 |

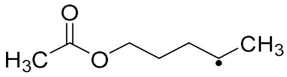


| | | | |
|---|---|---|---|
| C | -0.39534895 | 0.38967196 | -0.03109508 |
| H | -0.42078897 | 1.06239104 | 0.83054986 |
| H | -0.44685894 | 1.00621988 | -0.93271014 |
| C | 0.83679006 | -0.48895202 | -0.02118498 |
| H | 0.79786509 | -1.16619110 | -0.87955592 |
| H | 0.82961705 | -1.10918594 | 0.87931107 |
| C | 2.12132205 | 0.33800499 | -0.07041203 |
| H | 2.14965303 | 1.03178107 | 0.78087190 |
| H | 2.10137606 | 0.98132391 | -0.96668009 |
| C | 3.35550506 | -0.49928200 | -0.06819194 |
| H | 3.29696708 | -1.49943003 | -0.48345785 |
| C | -2.73397995 | 0.15160594 | 0.01223090 |
| O | -2.85383896 | 1.34426494 | -0.02336921 |
| O | -1.54203694 | -0.47005704 | 0.01323897 |
| C | -3.86119194 | -0.84338707 | 0.06125196 |
| H | -3.79560991 | -1.51089114 | -0.79867998 |
| H | -3.76978294 | -1.45289398 | 0.96098202 |
| H | -4.80943294 | -0.31303808 | 0.05776290 |
| C | 4.69136305 | 0.12826004 | 0.13175003 |
| H | 5.47059606 | -0.61724394 | 0.29580911 |
| H | 4.99342606 | 0.72923896 | -0.73892302 |
| H | 4.68322403 | 0.80872511 | 0.98966897 |

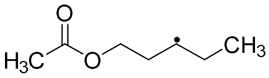


| | | | |
|---|---|---|---|
| C | -0.38122491 | 0.32868287 | -0.05779603 |
| H | -0.33716392 | 0.95131702 | 0.83874187 |
| H | -0.42734291 | 0.99431573 | -0.92370414 |
| C | 0.80536111 | -0.61197412 | -0.14188087 |
| H | 0.66349513 | -1.26749027 | -1.01524376 |
| H | 0.80594312 | -1.27098598 | 0.73329524 |
| C | 2.09556010 | 0.13001689 | -0.23249998 |
| H | 2.11942508 | 1.05727080 | -0.79861513 |
| C | 3.39042411 | -0.53474104 | 0.09034013 |
| H | 3.61123713 | -1.30954816 | -0.66139574 |
| H | 3.29334011 | -1.07535289 | 1.04004322 |
| C | -2.72658691 | 0.20343685 | 0.04066798 |
| O | -2.78741193 | 1.40079185 | 0.05738679 |
| O | -1.56732189 | -0.47517314 | -0.01929090 |
| C | -3.90020989 | -0.73666717 | 0.08243113 |
| H | -3.89019087 | -1.37213531 | -0.80379077 |
| H | -3.81626588 | -1.38484102 | 0.95550323 |
| H | -4.82055890 | -0.16088518 | 0.12570103 |
| C | 4.56115209 | 0.44458599 | 0.16309698 |
| H | 5.49497510 | -0.06740495 | 0.40025907 |
| H | 4.69228909 | 0.95956784 | -0.79151910 |
| H | 4.38183407 | 1.20118112 | 0.92980186 |

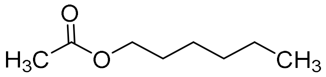


| | | | |
|---|---|---|---|
| C | 1.05739946 | -0.41967586 | 0.00000000 |
| H | 1.11617389 | -1.06301525 | 0.88214000 |
| H | 1.11617389 | -1.06301525 | -0.88214000 |
| C | -0.20267618 | 0.41902485 | 0.00000000 |
| H | -0.19567530 | 1.06993482 | -0.87968900 |
| H | -0.19567530 | 1.06993482 | 0.87968900 |
| C | -1.45963135 | -0.44794296 | 0.00000000 |
| H | -1.45182803 | -1.10476466 | 0.87783000 |
| H | -1.45182803 | -1.10476466 | -0.87783000 |
| C | -2.74474591 | 0.37649734 | 0.00000000 |
| H | -2.75385068 | 1.03393589 | -0.87758000 |
| H | -2.75385068 | 1.03393589 | 0.87758000 |
| C | 3.38816068 | -0.10713144 | 0.00000000 |
| O | 3.54581352 | -1.29598768 | 0.00000000 |
| O | 2.17742477 | 0.47649941 | 0.00000000 |
| C | 4.48401629 | 0.92363676 | 0.00000000 |
| H | 4.38573697 | 1.55975858 | -0.88037400 |
| H | 4.38573697 | 1.55975858 | 0.88037400 |
| H | 5.44835906 | 0.42312980 | 0.00000000 |
| C | -4.00727252 | -0.48247242 | 0.00000000 |
| H | -3.99555657 | -1.13860799 | 0.87683700 |
| H | -3.99555657 | -1.13860799 | -0.87683700 |
| C | -5.28419910 | 0.35423399 | 0.00000000 |
| H | -5.32705310 | 0.99781460 | -0.88239000 |

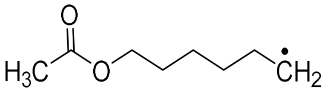


| | | | |
|---|---|---|---|
| C | 0.98497188 | -0.41303210 | 0.00000000 |
| H | 1.04062188 | -1.05659729 | 0.88217600 |
| H | 1.04062188 | -1.05659729 | -0.88217600 |
| C | -0.27139039 | 0.43152003 | 0.00000000 |
| H | -0.26179166 | 1.08225610 | -0.87970700 |
| H | -0.26179166 | 1.08225610 | 0.87970700 |
| C | -1.53144921 | -0.43092826 | 0.00000000 |
| H | -1.52676302 | -1.08754969 | 0.87797500 |
| H | -1.52676302 | -1.08754969 | -0.87797500 |
| C | -2.81210918 | 0.40062024 | 0.00000000 |
| H | -2.82695730 | 1.05554762 | -0.87781300 |
| H | -2.82695730 | 1.05554762 | 0.87781300 |
| C | 3.31689016 | -0.11162758 | 0.00000000 |
| O | 3.46831092 | -1.30123277 | 0.00000000 |
| O | 2.10882767 | 0.47798315 | 0.00000000 |
| C | 4.41775041 | 0.91369714 | 0.00000000 |
| H | 4.32262749 | 1.55022794 | -0.88041300 |
| H | 4.32262749 | 1.55022794 | 0.88041300 |
| H | 5.37960387 | 0.40842846 | 0.00000000 |
| C | -4.08295880 | -0.47137297 | 0.00000000 |
| H | -4.05352267 | -1.12341863 | 0.87962500 |
| H | -4.05352267 | -1.12341863 | -0.87962500 |
| C | -5.33605871 | 0.33374386 | 0.00000000 |

| | | | |
|---|---|---|---|
| H | -5.32705310 | 0.99781460 | 0.88239000 |
| H | -6.17646788 | -0.27435710 | 0.00000000 |

| | | | |
|---|---|---|---|
| H | -5.74950949 | 0.71045714 | -0.92602500 |
| H | -5.74950949 | 0.71045714 | 0.92602500 |

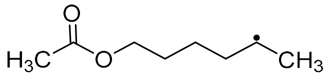

| | | | |
|---|---|---|---|
| C | 1.00570293 | 0.41723210 | -0.00600202 |
| H | 1.04813789 | 1.02754812 | -0.91214100 |
| H | 1.07180296 | 1.09323108 | 0.85080900 |
| C | -0.24838906 | -0.42896692 | 0.04256801 |
| H | -0.22444001 | -1.04671794 | 0.94566000 |
| H | -0.24854108 | -1.11200890 | -0.81249300 |
| C | -1.51004407 | 0.42980307 | 0.02773608 |
| H | -1.52571911 | 1.05226509 | -0.87352691 |
| H | -1.49700204 | 1.11741005 | 0.88119409 |
| C | -2.79053606 | -0.40374495 | 0.07875911 |
| H | -2.76326201 | -1.04141797 | 0.97891809 |
| H | -2.80739508 | -1.10323993 | -0.76809190 |
| C | 3.33821493 | 0.11856613 | -0.02316011 |
| O | 3.48831192 | 1.30754713 | -0.06647209 |
| O | 2.13109494 | -0.47177888 | 0.01204892 |
| C | 4.44018595 | -0.90538985 | -0.00088518 |
| H | 4.35473899 | -1.51293988 | 0.90072781 |
| H | 4.33657192 | -1.57034784 | -0.85907819 |
| H | 5.40149994 | -0.39981084 | -0.02748220 |
| C | -4.03298007 | 0.42052504 | 0.06834418 |
| H | -3.98860906 | 1.41985003 | 0.48792620 |
| C | -5.36203107 | -0.21707198 | -0.14643679 |
| H | -5.66889803 | -0.82133100 | 0.72035821 |
| H | -5.33930609 | -0.89663896 | -1.00481580 |
| H | -6.14557508 | 0.52228702 | -0.31811874 |

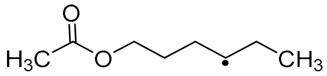

| | | | |
|---|---|---|---|
| C | -1.02456390 | 0.44536009 | -0.02878997 |
| H | -1.07063387 | 1.07134015 | 0.86653199 |
| H | -1.12872889 | 1.10240704 | -0.89665501 |
| C | 0.25498108 | -0.36080495 | -0.08524293 |
| H | 0.23599805 | -0.99641801 | -0.97550789 |
| H | 0.30158907 | -1.02375390 | 0.78304711 |
| C | 1.49004810 | 0.53959501 | -0.11654700 |
| H | 1.49733013 | 1.19143807 | 0.76735996 |
| H | 1.41513211 | 1.22262396 | -0.97975104 |
| C | 2.76789808 | -0.22648303 | -0.17864596 |
| H | 2.76203805 | -1.20061906 | -0.65984890 |
| C | -3.34471991 | 0.07140416 | 0.04298108 |
| O | -3.53254287 | 1.25544817 | 0.07166001 |
| O | -2.11976793 | -0.47992688 | -0.00906690 |
| C | -4.41265494 | -0.98800080 | 0.05935316 |
| H | -4.32659497 | -1.60565586 | -0.83531880 |
| H | -4.26891095 | -1.63655675 | 0.92427120 |
| H | -5.38922593 | -0.51352977 | 0.09969414 |
| C | 4.07873910 | 0.44698695 | 0.04796798 |
| H | 4.29140311 | 1.14258189 | -0.77991207 |
| C | 5.23903107 | -0.53773208 | 0.18822503 |
| H | 5.33890905 | -1.14545514 | -0.71427593 |
| H | 5.06970206 | -1.21320402 | 1.02938408 |
| H | 6.18526309 | -0.01944909 | 0.35126399 |
| H | 4.01266413 | 1.07812801 | 0.94316394 |

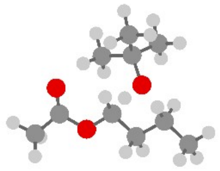

TS (1-α + tBuO radical)

| | | | |
|---|---|---|---|
| C | -0.36615367 | -1.07809304 | -0.38058004 |
| H | -0.29824454 | -1.56188501 | -1.35705304 |
| H | -0.29783089 | 0.12129398 | -0.65305002 |
| C | -1.66701666 | -1.27690830 | 0.35191991 |
| H | -1.64160381 | -0.70758230 | 1.28676391 |
| H | -1.75597247 | -2.33603932 | 0.62521589 |
| C | -2.86504371 | -0.84340351 | -0.49094714 |
| H | -2.87641257 | -1.41608250 | -1.42418514 |
| H | -2.73791590 | 0.20697651 | -0.76525812 |
| C | -4.18379671 | -1.04111878 | 0.25114381 |
| H | -4.20160586 | -0.45482779 | 1.17343481 |
| H | -4.32971152 | -2.09046781 | 0.52041879 |
| C | 1.88920845 | -1.75693261 | -0.16064096 |
| O | 2.03740250 | -1.75542156 | -1.34684295 |
| O | 0.71666836 | -1.43875784 | 0.44547600 |
| C | 2.94007347 | -2.07393842 | 0.86417208 |
| H | 3.18247627 | -1.16183138 | 1.41394910 |
| H | 2.55753458 | -2.80148050 | 1.57980406 |
| H | 3.82782356 | -2.45119524 | 0.36407711 |
| H | -5.03346374 | -0.73056693 | -0.35910322 |
| O | -0.46102413 | 1.40848095 | -0.84769401 |
| C | 0.38872970 | 2.11445310 | 0.02426503 |
| C | 0.04383743 | 3.58466904 | -0.25983396 |
| H | -1.00832762 | 3.77280884 | -0.04173200 |

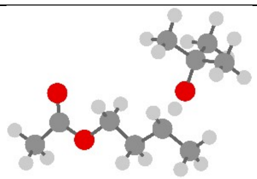

TS (1-γ + tBuO radical)

| | | | |
|---|---|---|---|
| C | -1.28681285 | 0.44867807 | -0.23073211 |
| H | -1.21519584 | 0.21016905 | -1.29643011 |
| H | -0.78578187 | -0.34520793 | 0.32606491 |
| C | -0.66668282 | 1.79400306 | 0.08375087 |
| H | -0.74403582 | 1.98300009 | 1.15876186 |
| H | -1.22925179 | 2.58589707 | -0.42603115 |
| C | 0.78707319 | 1.83668002 | -0.34200512 |
| H | 0.91113519 | 1.63186400 | -1.40978512 |
| H | 1.29518516 | 0.87718402 | 0.22487390 |
| C | 1.56766921 | 3.04658801 | 0.12046186 |
| H | 1.53818221 | 3.12879003 | 1.20922986 |
| H | 1.14114324 | 3.96503502 | -0.29772916 |
| C | -3.35532087 | -0.64103488 | -0.04317011 |
| O | -2.86617989 | -1.63589289 | -0.50143209 |
| O | -2.66965185 | 0.49954111 | 0.14367388 |
| C | -4.78560687 | -0.49030483 | 0.39756788 |
| H | -5.24588485 | 0.34717417 | -0.12761714 |
| H | -5.32395589 | -1.41143482 | 0.19189089 |
| H | -4.81283987 | -0.26710981 | 1.46502888 |
| H | 2.61291521 | 2.98876198 | -0.18808813 |
| O | 1.72150613 | -0.13941898 | 0.92647592 |
| C | 2.45676012 | -0.97800501 | 0.06604094 |
| C | 2.99104909 | -2.06923300 | 1.00658796 |
| H | 2.16015407 | -2.58863498 | 1.48598697 |

| | | | |
|---|---|---|---|
| H | 0.66138428 | 4.23287015 | 0.36668307 |
| H | 0.23291743 | 3.81660009 | -1.30889895 |
| C | 1.85603476 | 1.84522539 | -0.31130791 |
| H | 2.10179796 | 0.79754643 | -0.12641091 |
| H | 2.04101077 | 2.05426644 | -1.36608390 |
| H | 2.51788862 | 2.46527251 | 0.29871412 |
| C | 0.08120771 | 1.77164003 | 1.48355402 |
| H | -0.98051133 | 1.93098282 | 1.68581098 |
| H | 0.32588590 | 0.72621307 | 1.68815501 |
| H | 0.66602256 | 2.39915813 | 2.16076705 |

| | | | |
|---|---|---|---|
| H | 3.58069807 | -2.78976603 | 0.43522398 |
| H | 3.61986309 | -1.62285900 | 1.77819996 |
| C | 3.61925214 | -0.21185805 | -0.56778807 |
| H | 3.24153316 | 0.56385695 | -1.24004808 |
| H | 4.21732015 | 0.26547795 | 0.21100293 |
| H | 4.25988013 | -0.88046407 | -1.14799405 |
| C | 1.55657311 | -1.59896201 | -1.00384406 |
| H | 2.11317410 | -2.31779403 | -1.61025104 |
| H | 0.70986410 | -2.11135898 | -0.54175305 |
| H | 1.16678214 | -0.82572301 | -1.67149907 |

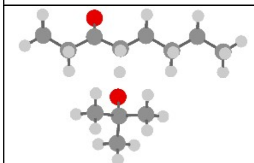

TS (32-α + tBuO radical)

| | | | |
|---|---|---|---|
| C | 4.84591594 | -0.50099982 | -0.04878119 |
| H | 4.92469794 | -0.20090786 | -1.09711120 |
| H | 4.77058212 | 0.40923921 | 0.55194679 |
| H | 5.77318788 | -1.00740396 | 0.22520378 |
| C | 3.63027181 | -1.39763162 | 0.16956589 |
| H | 3.73665364 | -2.31624465 | -0.41740309 |
| H | 3.58108581 | -1.70614659 | 1.21918490 |
| C | 2.32198790 | -0.70827142 | -0.20929506 |
| H | 2.36818190 | -0.40233945 | -1.26258407 |
| H | 2.20784908 | 0.20918861 | 0.37836992 |
| C | 1.10256177 | -1.59505822 | 0.02005501 |
| H | 1.03204078 | -1.87362118 | 1.07527502 |
| H | 1.21536560 | -2.53898525 | -0.52870197 |
| C | -0.19970814 | -0.95461302 | -0.39918094 |
| H | -0.22953514 | -0.63977104 | -1.44769295 |
| H | -0.31917195 | 0.10714501 | 0.21516604 |
| C | -1.44032425 | -1.71703181 | -0.00545086 |
| C | -2.77480816 | -1.07672061 | -0.32797682 |
| H | -2.80729998 | -0.14103859 | 0.24190716 |
| H | -2.77487216 | -0.78163664 | -1.38328983 |
| C | -3.95602729 | -1.97542041 | 0.00542626 |
| H | -3.93681628 | -2.25406339 | 1.05925127 |
| H | -4.89922522 | -1.47040727 | -0.20675871 |
| H | -3.91701646 | -2.89805943 | -0.57537272 |
| O | -1.35908239 | -2.77231381 | 0.57451916 |
| O | -0.47647974 | 1.21094705 | 0.90480902 |
| C | -0.50658460 | 2.33837004 | 0.06314699 |
| C | -0.68592437 | 3.49958409 | 1.05708597 |
| H | 0.14998767 | 3.52464498 | 1.75686493 |
| H | -0.72398824 | 4.44339408 | 0.50829495 |
| H | -1.61292736 | 3.37320025 | 1.61755902 |
| C | -1.69334165 | 2.28784720 | -0.90007495 |
| H | -1.59446082 | 1.44277717 | -1.58656193 |
| H | -2.62825964 | 2.17794637 | -0.34708690 |
| H | -1.74447253 | 3.20302220 | -1.49464597 |
| C | 0.80726639 | 2.50170481 | -0.70104707 |
| H | 1.64747843 | 2.53328569 | -0.00515311 |
| H | 0.95787123 | 1.66303577 | -1.38560706 |
| H | 0.79984851 | 3.42412480 | -1.28651910 |

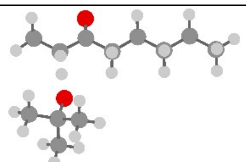

TS (32-α′ + tBuO radical)

| | | | |
|---|---|---|---|
| C | 5.62569913 | -1.58454088 | 0.10504602 |
| H | 5.66146312 | -1.71831590 | 1.18944502 |
| H | 5.22540415 | -2.50450988 | -0.32913700 |
| H | 6.65011613 | -1.46392385 | -0.25196597 |
| C | 4.75587911 | -0.38612989 | -0.26584197 |
| H | 5.18210409 | 0.52765411 | 0.16164405 |
| H | 4.75865312 | -0.24830587 | -1.35217497 |
| C | 3.31438711 | -0.53728093 | 0.21442801 |
| H | 3.31085810 | -0.68774495 | 1.30133501 |
| H | 2.88166213 | -1.44576793 | -0.22263701 |
| C | 2.44190409 | 0.66458606 | -0.13682998 |
| H | 2.44455009 | 0.82751008 | -1.21862298 |
| H | 2.86268006 | 1.57366606 | 0.30375704 |
| C | 1.00497708 | 0.49782902 | 0.33450000 |
| H | 0.96293508 | 0.32321100 | 1.41727700 |
| H | 0.53283711 | -0.37924298 | -0.12653502 |
| C | 0.12372506 | 1.68658601 | 0.00959701 |
| C | -1.31229994 | 1.59142897 | 0.45452500 |
| H | -1.70480691 | 0.63395198 | -0.19406303 |
| H | -1.38890294 | 1.24993396 | 1.49026099 |
| C | -2.18106096 | 2.77652596 | 0.11327001 |
| H | -2.10161395 | 3.00889298 | -0.94878299 |
| H | -3.22603896 | 2.58093594 | 0.35853599 |
| H | -1.85346498 | 3.66114896 | 0.66650203 |
| O | 0.52466205 | 2.64527303 | -0.60367897 |
| O | -2.07994088 | -0.41644602 | -0.89527305 |
| C | -2.81005588 | -1.28685905 | -0.06751807 |
| C | -3.32257484 | -2.35337404 | -1.05122510 |
| H | -3.89609584 | -3.10513606 | -0.50425512 |
| H | -3.96131285 | -1.89126204 | -1.80470509 |
| H | -2.48251183 | -2.83843201 | -1.54991710 |
| C | -3.99099690 | -0.56100108 | 0.57946393 |
| H | -3.63545892 | 0.18604391 | 1.29431994 |
| H | -4.57959890 | -0.05427108 | -0.18766807 |
| H | -4.63384489 | -1.26382710 | 1.11440891 |
| C | -1.90572688 | -1.93039805 | 0.98444493 |
| H | -2.45881887 | -2.65791607 | 1.58341791 |
| H | -1.06972186 | -2.43857102 | 0.49908193 |
| H | -1.50197790 | -1.17057105 | 1.65893595 |

t-BuO radical

| | | | |
|---|---|---|---|
| O | 0.25452869 | 1.42793431 | 0.00000000 |
| C | -0.02293952 | 0.07416465 | 0.00000000 |
| C | 1.38081428 | -0.57316826 | 0.00000000 |

t-BuOH

| | | | |
|---|---|---|---|
| O | 0.02843714 | 1.44289483 | 0.00000000 |
| C | -0.00584016 | 0.01564898 | 0.00000000 |
| C | 0.68183849 | -0.51340500 | 1.25786500 |

| | | | |
|---|---|---|---|
| H | 1.93597646 | -0.27273798 | 0.88897000 |
| H | 1.26061986 | -1.65842175 | 0.00000000 |
| H | 1.93597646 | -0.27273798 | -0.88897000 |
| C | -0.78712532 | -0.31551670 | -1.26860900 |
| H | -1.75420109 | 0.19099936 | -1.29168000 |
| H | -0.21935642 | -0.01720527 | -2.15111800 |
| H | -0.96171599 | -1.39347145 | -1.30232700 |
| C | -0.78712532 | -0.31551670 | 1.26860900 |
| H | -0.21935642 | -0.01720527 | 2.15111800 |
| H | -1.75420109 | 0.19099936 | 1.29168000 |
| H | -0.96171599 | -1.39347145 | 1.30232700 |

| | | | |
|---|---|---|---|
| H | 0.20367444 | -0.09541100 | 2.14559800 |
| H | 0.63046402 | -1.60361157 | 1.30883500 |
| H | 1.73759107 | -0.22506074 | 1.26710000 |
| C | 0.68183849 | -0.51340500 | -1.25786500 |
| H | 0.20367444 | -0.09541100 | -2.14559800 |
| H | 1.73759107 | -0.22506074 | -1.26710000 |
| H | 0.63046402 | -1.60361157 | -1.30883500 |
| C | -1.48524318 | -0.34397207 | 0.00000000 |
| H | -1.96784762 | 0.07441867 | 0.88505900 |
| H | -1.96784762 | 0.07441867 | -0.88505900 |
| H | -1.62129742 | -1.42737224 | 0.00000000 |
| H | 0.95047467 | 1.71434143 | 0.00000000 |

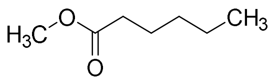

| | | | |
|---|---|---|---|
| C | 0.00000000 | -0.77765500 | 0.00000000 |
| H | -0.40857700 | -1.29603800 | 0.87326300 |
| H | -0.40857700 | -1.29603800 | -0.87326300 |
| C | -0.37921400 | 0.69657700 | 0.00000000 |
| H | 0.06603200 | 1.18188700 | -0.87351900 |
| H | 0.06603200 | 1.18188700 | 0.87351900 |
| C | -1.89071700 | 0.90659200 | 0.00000000 |
| H | -2.32978000 | 0.41642200 | 0.87764400 |
| H | -2.32978000 | 0.41642200 | -0.87764400 |
| C | -2.28317100 | 2.38237300 | 0.00000000 |
| H | -1.84364200 | 2.86981300 | -0.87656100 |
| H | -1.84364200 | 2.86981300 | 0.87656100 |
| C | 3.19271100 | -2.59911600 | 0.00000000 |
| H | 3.67041500 | -2.18133800 | -0.88671400 |
| H | 3.67041500 | -2.18133800 | 0.88671400 |
| H | 3.26476500 | -3.68324000 | 0.00000000 |
| C | -3.79595300 | 2.58650600 | 0.00000000 |
| H | -4.25027000 | 2.12858400 | 0.88243700 |
| H | -4.25027000 | 2.12858400 | -0.88243700 |
| H | -4.05810600 | 3.64602200 | 0.00000000 |
| C | 1.49297400 | -0.99194400 | 0.00000000 |
| O | 2.32440300 | -0.12652000 | 0.00000000 |
| O | 1.79624700 | -2.30116100 | 0.00000000 |

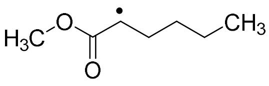

| | | | |
|---|---|---|---|
| C | 0.60973600 | -1.16029900 | -0.19535700 |
| H | 0.90080000 | -1.90177800 | -0.92746600 |
| C | -0.74646300 | -1.12739800 | 0.40625600 |
| H | -0.66452100 | -0.82110400 | 1.45384100 |
| H | -1.20036600 | -2.12108200 | 0.37235900 |
| C | -1.66573200 | -0.11886400 | -0.31276900 |
| H | -1.73544800 | -0.38045200 | -1.37438300 |
| H | -1.20298200 | 0.87087500 | -0.25271600 |
| C | -3.06371500 | -0.07475200 | 0.29895700 |
| H | -2.98103100 | 0.18085500 | 1.36032500 |
| H | -3.51142200 | -1.07329100 | 0.25244200 |
| C | 3.70613800 | 0.73811400 | -0.22166400 |
| H | 3.33318000 | 1.72354200 | -0.50401000 |
| H | 3.94339300 | 0.74586400 | 0.84298300 |
| H | 4.58350000 | 0.47822700 | -0.80782500 |
| C | -3.97059100 | 0.93110600 | -0.40546300 |
| H | -4.08517400 | 0.67825100 | -1.46260300 |
| H | -3.55042500 | 1.93814300 | -0.34774600 |
| H | -4.96529000 | 0.95577600 | 0.04321600 |
| C | 1.55844300 | -0.11385600 | 0.13496700 |
| O | 1.33644700 | 0.79956400 | 0.89954400 |
| O | 2.73466400 | -0.26433000 | -0.50929200 |

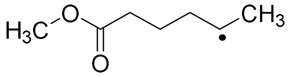

| | | | |
|---|---|---|---|
| C | 0.43144000 | -0.62225900 | 0.04531700 |
| H | 0.43249000 | -1.23699400 | 0.95137500 |
| H | 0.41929500 | -1.32552700 | -0.79317600 |
| C | -0.76954400 | 0.31140200 | 0.00903200 |
| H | -0.73652300 | 0.91260000 | -0.90339600 |
| H | -0.70195500 | 1.01784900 | 0.84102000 |
| C | -2.09399800 | -0.44686400 | 0.07967700 |
| H | -2.10895400 | -1.06035300 | 0.99723200 |
| H | -2.15417200 | -1.16845500 | -0.74725900 |
| C | -3.28501100 | 0.44945600 | 0.04324100 |
| H | -3.17137700 | 1.46857600 | 0.39606000 |
| C | 4.07050700 | -0.11374200 | -0.04103300 |
| H | 4.17611100 | 0.49165000 | -0.94175100 |
| H | 4.21075500 | 0.52552300 | 0.83111400 |
| H | 4.78970200 | -0.92815900 | -0.03952800 |
| C | -4.65275300 | -0.11807100 | -0.11724500 |
| H | -4.97814500 | -0.65840400 | 0.78433500 |
| H | -4.68613100 | -0.84063000 | -0.93961600 |
| H | -5.39471100 | 0.65678200 | -0.31491000 |

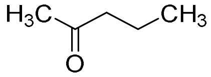

| | | | |
|---|---|---|---|
| C | 1.12955107 | 0.14542607 | 0.01326787 |
| C | -0.22522287 | -0.53997503 | 0.03419813 |
| C | -1.39665696 | 0.42963686 | -0.03816495 |
| H | -0.25699189 | -1.26112920 | -0.79211773 |
| H | -0.27675873 | -1.14495286 | 0.94867625 |
| C | -2.74126689 | -0.29094425 | 0.00248931 |
| H | -1.31825594 | 1.13995002 | 0.78798290 |
| H | -1.30998409 | 1.02296969 | -0.95178708 |
| H | -3.57084596 | 0.41601768 | -0.05012475 |
| H | -2.84107691 | -0.98597442 | -0.83542255 |
| H | -2.85089176 | -0.86622308 | 0.92562243 |
| C | 2.34148914 | -0.76173983 | -0.02080307 |
| H | 2.37343810 | -1.28918402 | -0.97803897 |
| H | 3.24615111 | -0.16890573 | 0.09703473 |
| H | 2.27986328 | -1.51822368 | 0.76473408 |
| O | 1.23474897 | 1.34515408 | 0.01718962 |

| | | | |
|---|---|---|---|
| C | 1.74333900 | 0.12058900 | 0.00050500 |
| O | 1.87180700 | 1.31337700 | -0.02549500 |
| O | 2.78316100 | -0.73056700 | -0.00431400 |

| | | | |
|---|---|---|---|
| C | -0.95874100 | 0.13079100 | -0.07966600 |
| C | 0.12821200 | -0.81252000 | -0.23688900 |
| C | 1.49014800 | -0.33624400 | -0.59532100 |
| H | -0.04985800 | -1.86182900 | -0.02516700 |
| C | 2.23593600 | 0.19070000 | 0.64587200 |
| H | 2.06344500 | -1.14198500 | -1.05845400 |
| H | 1.39569000 | 0.48224100 | -1.31285800 |
| H | 3.23660900 | 0.53029300 | 0.37350500 |
| H | 1.68769600 | 1.03139000 | 1.07253100 |
| H | 2.33252000 | -0.58686000 | 1.40639800 |
| C | -2.31520200 | -0.41110600 | 0.32439100 |
| H | -2.26033900 | -0.86126600 | 1.31878200 |
| H | -3.03670000 | 0.40306100 | 0.33515300 |
| H | -2.64315500 | -1.18682400 | -0.37130700 |
| O | -0.77600300 | 1.32775600 | -0.26111400 |

| | | | |
|---|---|---|---|
| C | -2.76180743 | 0.76709455 | 0.00000000 |
| H | -2.71260284 | 1.40884871 | 0.88039500 |
| H | -2.71260284 | 1.40884871 | -0.88039500 |
| H | -3.68492088 | 0.19409904 | 0.00000000 |
| O | -0.42758245 | 0.49797509 | 0.00000000 |
| C | -1.59022903 | -0.17667813 | 0.00000000 |
| O | -1.65610741 | -1.37406730 | 0.00000000 |
| C | 0.75737746 | -0.30959075 | 0.00000000 |
| H | 0.74895956 | -0.95582054 | 0.88213300 |
| H | 0.74895956 | -0.95582054 | -0.88213300 |
| C | 1.95059630 | 0.62235743 | 0.00000000 |
| H | 1.89174066 | 1.26935941 | -0.87895600 |
| H | 1.89174066 | 1.26935941 | 0.87895600 |
| C | 3.26137212 | -0.16032964 | 0.00000000 |
| H | 3.33727365 | -0.79971262 | -0.88281900 |
| H | 4.11984121 | 0.51216792 | 0.00000000 |
| H | 3.33727365 | -0.79971262 | 0.88281900 |

| | | | |
|---|---|---|---|
| C | -2.74692500 | -0.74796989 | 0.03171597 |
| H | -2.66783199 | -1.45644188 | -0.79345904 |
| H | -2.72668799 | -1.31837590 | 0.96143896 |
| H | -3.67009300 | -0.17987290 | -0.04130802 |
| O | -0.40946200 | -0.49382488 | 0.04418696 |
| C | -1.58072600 | 0.19723412 | 0.00178597 |
| O | -1.63554501 | 1.39039012 | -0.04864701 |
| C | 0.76649200 | 0.21923713 | 0.06538896 |
| H | 0.69094699 | 1.26718313 | -0.19284803 |
| C | 1.98499400 | -0.60029686 | -0.14505605 |
| H | 1.95252701 | -1.46491287 | 0.52616794 |
| H | 1.99145300 | -1.01059085 | -1.16600705 |
| C | 3.25369300 | 0.21673315 | 0.09323195 |
| H | 3.27984000 | 0.60010814 | 1.11487096 |
| H | 4.14556600 | -0.38938285 | -0.06850406 |
| H | 3.29916799 | 1.07013816 | -0.58707204 |

| | | | |
|---|---|---|---|
| C | -2.38230823 | -0.85874999 | -0.06202359 |
| H | -2.00890031 | -1.86625802 | -0.24928561 |
| H | -2.83632409 | -0.85340993 | 0.92943147 |
| H | -3.11351533 | -0.57958899 | -0.81565750 |
| O | -0.31389007 | -0.19633906 | 0.83152007 |
| C | -1.23546819 | 0.11551395 | -0.09540181 |
| O | -1.13938125 | 1.05271991 | -0.83849986 |
| C | 0.84602798 | 0.68119089 | 0.87306786 |
| H | 0.49766604 | 1.71340491 | 0.89996787 |
| H | 1.31564211 | 0.42168290 | 1.82417881 |
| C | 1.76458780 | 0.44390279 | -0.26880526 |
| H | 1.76728972 | 1.15204676 | -1.08639029 |
| C | 2.39636071 | -0.89309825 | -0.44303530 |
| H | 2.66798783 | -1.32995422 | 0.52244868 |
| H | 1.71002460 | -1.60228523 | -0.92615816 |
| H | 3.29109962 | -0.83924132 | -1.06550843 |

| | | | |
|---|---|---|---|
| C | -2.68960595 | -0.78115899 | 0.03228802 |
| H | -2.64472494 | -1.44183998 | -0.83425298 |
| H | -2.62277193 | -1.40242399 | 0.92608602 |
| H | -3.61853496 | -0.21768001 | 0.03095703 |
| O | -0.35806495 | -0.48922994 | 0.00250100 |
| C | -1.52826397 | 0.17403704 | -0.00395999 |
| O | -1.60503799 | 1.37004504 | -0.03465399 |
| C | 0.81678403 | 0.33007309 | -0.03534501 |
| H | 0.80321801 | 0.94153509 | -0.94042401 |
| H | 0.81140702 | 1.00610809 | 0.82382299 |
| C | 2.01857005 | -0.59681788 | -0.00508802 |
| H | 1.92744107 | -1.23913089 | 0.88366098 |
| H | 1.97963906 | -1.26599688 | -0.86886902 |
| C | 3.29710703 | 0.16587514 | 0.01697497 |
| H | 3.37537302 | 1.08383415 | 0.58554697 |
| H | 4.20623004 | -0.26297684 | -0.37852404 |

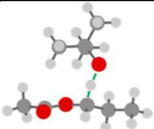

TS (11-α + tBuO radical)

| | | | |
|---|---|---|---|
| C | -2.94991379 | -1.67290028 | 0.76349692 |
| H | -3.59533369 | -2.34810136 | 0.20845991 |
| H | -2.19039673 | -2.24216018 | 1.30391795 |
| H | -3.52151788 | -1.10267535 | 1.49548790 |
| O | -1.56222302 | 0.20227490 | 0.48074495 |
| C | -2.26433588 | -0.74236520 | -0.19497306 |
| O | -2.29358783 | -0.81039521 | -1.38833506 |
| C | -0.82111712 | 1.13010599 | -0.27706903 |
| H | 0.27745795 | 0.65067712 | -0.56769300 |
| H | -1.28540711 | 1.31037892 | -1.24875105 |
| C | -0.53321730 | 2.35978903 | 0.54412597 |
| H | -1.48740736 | 2.80159391 | 0.85291894 |
| H | -0.00893529 | 2.05893510 | 1.45558199 |

| | | | |
|---|---|---|---|
| C | 0.30217360 | 3.36465713 | -0.24513301 |
| H | -0.22361642 | 3.68618206 | -1.14740203 |
| H | 0.51271447 | 4.25089816 | 0.35455099 |
| H | 1.24975567 | 2.91408625 | -0.54418098 |
| O | 1.54562200 | 0.36900829 | -0.74647796 |
| C | 1.86963912 | -0.77140667 | 0.01253306 |
| C | 3.37997315 | -0.93405848 | -0.21904189 |
| H | 3.91173602 | -0.05136541 | 0.13893612 |
| H | 3.73947424 | -1.81328543 | 0.32106712 |
| H | 3.58350419 | -1.06125946 | -1.28309889 |
| C | 1.12298329 | -1.99856777 | -0.51058295 |
| H | 0.04467727 | -1.86417490 | -0.40272099 |
| H | 1.33655634 | -2.13960075 | -1.57140495 |
| H | 1.41518339 | -2.89926573 | 0.03528306 |
| C | 1.58832704 | -0.53673770 | 1.49842505 |
| H | 1.93786013 | -1.38121765 | 2.09750707 |
| H | 2.10218992 | 0.36712837 | 1.83366906 |
| H | 0.51594302 | -0.41497383 | 1.66973002 |

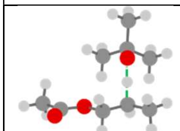

TS (11-β + tBuO radical)

| | | | |
|---|---|---|---|
| C | 2.98501408 | -1.97216082 | -0.48349429 |
| H | 3.91017007 | -2.39920586 | -0.10600331 |
| H | 2.13470507 | -2.59248086 | -0.19603439 |
| H | 3.00517111 | -1.92183167 | -1.57273428 |
| O | 1.68230406 | -0.02072784 | -0.38653607 |
| C | 2.80822405 | -0.59023789 | 0.08237589 |
| O | 3.54901702 | -0.04319599 | 0.84893498 |
| C | 1.38889004 | 1.28627710 | 0.11672009 |
| H | 1.39001301 | 1.26437495 | 1.20975909 |
| H | 2.17688405 | 1.97833014 | -0.20449480 |
| C | 0.04586905 | 1.69118216 | -0.42337090 |
| H | -0.03822491 | 1.48731730 | -1.49298293 |
| H | -0.75224096 | 0.94043508 | 0.13626298 |
| C | -0.39540897 | 3.08127810 | -0.02582773 |
| H | 0.27392904 | 3.84175316 | -0.44200461 |
| H | -1.40456696 | 3.28495915 | -0.38557774 |
| H | -0.39714100 | 3.18922096 | 1.06115228 |
| O | -1.56893898 | 0.28162398 | 0.88798486 |
| C | -2.22131095 | -0.66628892 | 0.07693072 |
| C | -3.20009797 | -1.34978205 | 1.04399660 |
| H | -3.89850299 | -0.61741811 | 1.45146367 |
| H | -3.76040595 | -2.12285799 | 0.51270648 |
| H | -2.65237600 | -1.80926616 | 1.86797255 |
| C | -2.99226792 | 0.02595522 | -1.04870022 |
| H | -3.66393993 | 0.77837316 | -0.63073114 |
| H | -2.29958990 | 0.52096932 | -1.73447413 |
| H | -3.58058889 | -0.69460671 | -1.62194233 |
| C | -1.22394592 | -1.68425084 | -0.47701338 |
| H | -0.67939295 | -2.14840195 | 0.34828557 |
| H | -1.73595590 | -2.46311877 | -1.04790950 |
| H | -0.49679591 | -1.18997875 | -1.12548430 |

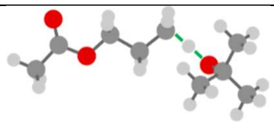

TS (11-γ + tBuO radical)

| | | | |
|---|---|---|---|
| C | -4.99392795 | -1.30868085 | -0.02037055 |
| H | -4.91814796 | -1.70788916 | -1.03244343 |
| H | -4.64962897 | -2.08236864 | 0.66694469 |
| H | -6.02094595 | -1.03317976 | 0.20316636 |
| O | -2.83228694 | -0.40000693 | -0.16721483 |
| C | -4.11355693 | -0.09630882 | 0.10860608 |
| O | -4.47613391 | 1.00460428 | 0.41488473 |
| C | -1.90582491 | 0.68967008 | -0.07630216 |
| H | -2.20891990 | 1.47716787 | -0.77146741 |
| H | -1.94128491 | 1.10687439 | 0.93375671 |
| C | -0.52899492 | 0.14298994 | -0.41111698 |
| H | -0.29277794 | -0.66822984 | 0.28265727 |
| H | -0.55088893 | -0.28795837 | -1.41497885 |
| C | 0.52415610 | 1.22454495 | -0.33329232 |
| H | 0.69493110 | 1.62228626 | 0.66806556 |
| H | 1.59245409 | 0.67962682 | -0.68735414 |
| H | 0.40664912 | 2.02588872 | -1.06398556 |
| O | 2.57847408 | -0.01743431 | -1.03908992 |
| C | 3.41256807 | -0.21215698 | 0.08049014 |
| C | 2.67280205 | -0.96606562 | 1.18655737 |
| H | 3.34692504 | -1.21180138 | 2.01048645 |
| H | 1.85737206 | -0.35676748 | 1.58538018 |
| H | 2.25126703 | -1.89064374 | 0.78672666 |
| C | 3.96216109 | 1.12192417 | 0.58706273 |
| H | 4.68039909 | 0.96915140 | 1.39623878 |
| H | 4.45656011 | 1.65127790 | -0.22949343 |
| H | 3.15165910 | 1.75062230 | 0.96436353 |
| C | 4.55299005 | -1.07664318 | -0.47691559 |
| H | 5.27701305 | -1.28564994 | 0.31416948 |
| H | 4.15708204 | -2.01898929 | -0.85820730 |
| H | 5.05562107 | -0.55236444 | -1.29097775 |
